# Effects of a Low-Carbohydrate Dietary Intervention on Hemoglobin A_1c_: A Randomized Clinical Trial

**DOI:** 10.1001/jamanetworkopen.2022.38645

**Published:** 2022-10-26

**Authors:** Kirsten S. Dorans, Lydia A. Bazzano, Lu Qi, Hua He, Jing Chen, Lawrence J. Appel, Chung-Shiuan Chen, Ming-Hui Hsieh, Frank B. Hu, Katherine T. Mills, Bernadette T. Nguyen, Matthew J. O’Brien, Jonathan M. Samet, Gabriel I. Uwaifo, Jiang He

**Affiliations:** 1 Department of Epidemiology, Tulane University School of Public Health and Tropical Medicine, New Orleans, Louisiana; 2 Tulane University Translational Science Institute, New Orleans, Louisiana; 3 Department of Medicine, Tulane University School of Medicine, New Orleans, Louisiana; 4 Welch Center for Prevention, Epidemiology, and Clinical Research, Johns Hopkins University Bloomberg School of Public Health, Baltimore, Maryland; 5 Department of Nutrition, Harvard T.H. Chan School of Public Health, Boston, Massachusetts; 6 Channing Division of Network Medicine, Brigham and Women’s Hospital and Harvard Medical School, Boston, Massachusetts; 7 Department of Epidemiology, Harvard T.H. Chan School of Public Health, Boston, Massachusetts; 8 Division of General Internal Medicine and Geriatrics, Department of Medicine, Northwestern University Feinberg School of Medicine, Chicago, Illinois; 9 Institute of Public Health and Medicine, Northwestern University Feinberg School of Medicine, Chicago, Illinois; 10 Department of Preventive Medicine, Northwestern University Feinberg School of Medicine, Chicago, Illinois; 11 Colorado School of Public Health, Aurora; 12 Department of Endocrinology, Diabetes, Metabolism, and Weight Management, Ochsner Medical Center, New Orleans, Louisiana

## Abstract

**Importance:**

Low-carbohydrate diets decrease hemoglobin A_1c_ (HbA_1c_) among patients with type 2 diabetes at least as much as low-fat diets. However, evidence on the effects of low-carbohydrate diets on HbA_1c_ among individuals with HbA_1c_ in the range of prediabetes to diabetes not treated by diabetes medications is limited.

**Objective:**

To study the effect of a behavioral intervention promoting a low-carbohydrate diet compared with usual diet on 6-month changes in HbA_1c_ among individuals with elevated untreated HbA_1c_.

**Design, Setting, and Participants:**

This 6-month randomized clinical trial with 2 parallel groups was conducted from September 2018 to June 2021 at an academic medical center in New Orleans, Louisiana. Laboratory analysts were blinded to assignment. Participants were aged 40 to 70 years with untreated HbA_1c_ of 6.0% to 6.9% (42-52 mmol/mol). Data analysis was performed from November 2021 to September 2022.

**Interventions:**

Participants were randomized to a low-carbohydrate diet intervention (target <40 net grams of carbohydrates during the first 3 months; <60 net grams for months 3 to 6) or usual diet. The low-carbohydrate diet group received dietary counseling.

**Main Outcomes and Measures:**

Six-month change in HbA_1c_ was the primary outcome. Outcomes were measured at 0, 3, and 6 months.

**Results:**

Of 2722 prescreened participants, 962 underwent screening, and 150 were enrolled (mean [SD] age, 58.9 [7.9] years; 108 women [72%]; 88 Black participants [59%]) and randomized to either the low-carbohydrate diet intervention (75 participants) or usual diet (75 participants) group. Six-month data were collected on 142 participants (95%). Mean (SD) HbA_1c_ was 6.16% (0.30%) at baseline. Compared with the usual diet group, the low-carbohydrate diet intervention group had significantly greater 6-month reductions in HbA_1c_ (net difference, –0.23%; 95% CI, –0.32% to –0.14%; *P* < .001), fasting plasma glucose (–10.3 mg/dL; 95% CI, –15.6 to –4.9 mg/dL; *P* < .001), and body weight (–5.9 kg; 95% CI, –7.4 to –4.4 kg; *P* < .001).

**Conclusions and Relevance:**

In this randomized clinical trial, a low-carbohydrate dietary intervention led to improvements in glycemia in individuals with elevated HbA_1c_ not taking glucose-lowering medication, but the study was unable to evaluate its effects independently of weight loss. This diet, if sustained, might be a useful dietary approach for preventing and treating type 2 diabetes, but more research is needed.

**Trial Registration:**

ClinicalTrials.gov Identifier: NCT03675360

## Key Points

**Question:**

What is the effect of a dietary intervention promoting a low-carbohydrate diet compared with usual diet on 6-month change in hemoglobin A_1c_ among adults with untreated hemoglobin A_1c_ of 6.0% to 6.9%?

**Findings:**

In this randomized clinical trial that included 150 adults, the low-carbohydrate diet intervention significantly reduced hemoglobin A_1c_ by 0.23% compared with usual diet over 6 months.

**Meaning:**

These findings suggest that a low-carbohydrate diet, if sustained, might be a useful dietary approach for preventing and treating type 2 diabetes, but more research is needed.

## Teaser

This randomized clinical trial examines the effect of a behavioral intervention promoting a low-carbohydrate diet compared with usual diet on 6-month changes in hemoglobin A_1c_ (HbA_1c_) among individuals with elevated untreated HbA_1c_.

## Introduction

Given its increasing prevalence and high disease burden,^1,2^ prevention of type 2 diabetes (T2D) is a major public health priority. Individuals with prediabetes have elevated risk of T2D and other cardiometabolic diseases.^3,4^ Much evidence supports the crucial role of diet in preventing T2D,^5,6,7,8,9^ with most dietary interventions focused on reducing caloric and total fat intake.

Evidence suggests low-to-moderate carbohydrate diets (<45% energy from carbohydrates) are at least as effective as low-fat diets at promoting weight loss and improving cardiovascular risk factors.^10,11^ In meta-analyses^12,13^ of trials among individuals with T2D, larger carbohydrate restriction corresponded with larger hemoglobin A_1c_ (HbA_1c_) reductions.

If sustained, interventions that lower HbA_1c_ in the short-term may lead to T2D prevention, as was observed in the Diabetes Prevention Program (DPP) trial.^5^ To our knowledge, only 2 pilot trials^14,15,16^ have assessed glycemic effects of low-carbohydrate diets in participants with prediabetes. One study^14,15^ only included 4 people with prediabetes, and the other^16^ studied only a moderately low-carbohydrate intervention (≤130 g/d). Given the benefits of low-carbohydrate diets for weight loss in general populations and for glycemic control for diabetes, studying effects of these diets on glycemic biomarkers is warranted among individuals with untreated prediabetes and diabetes.

In this randomized clinical trial, we tested effects of a behavioral intervention promoting a healthy, low-carbohydrate diet compared with usual diet on HbA_1c_ and metabolic risk factors among adults with untreated HbA_1c_ of 6.0% to 6.9%. The low-carbohydrate diet promoted was characterized by components thought to improve cardiometabolic health: unsaturated fat and protein, high-fiber foods, and minimal refined carbohydrates.^17^ The HbA_1c_ range was chosen as the lower bound aligns with the World Health Organization lower cutoff point for prediabetes and the upper bound with the less than 7.0% American Diabetes Association HbA_1c_ target.^3,18^

## Methods

### Setting and Participants

Adults aged 40 to 70 years with untreated HbA_1c_ of 6.0% to 6.9% (42-52 mmol/mol) were recruited from the greater New Orleans, Louisiana, area primarily by mass mailing. Major exclusion criteria included using glucose-lowering medications and type 1 diabetes. The study protocol has been published elsewhere^19^ and is available in Supplement 1.

Recruitment occurred from September 2018 through December 2020, with follow-up through June 2021. The study was approved by the Tulane University institutional review board and follows the Consolidated Standards of Reporting Trials (CONSORT) reporting guideline for trial studies.^20^ Trained staff obtained written informed consent from participants at the screening visit.

### Study Design and Intervention

**Figure 1.**
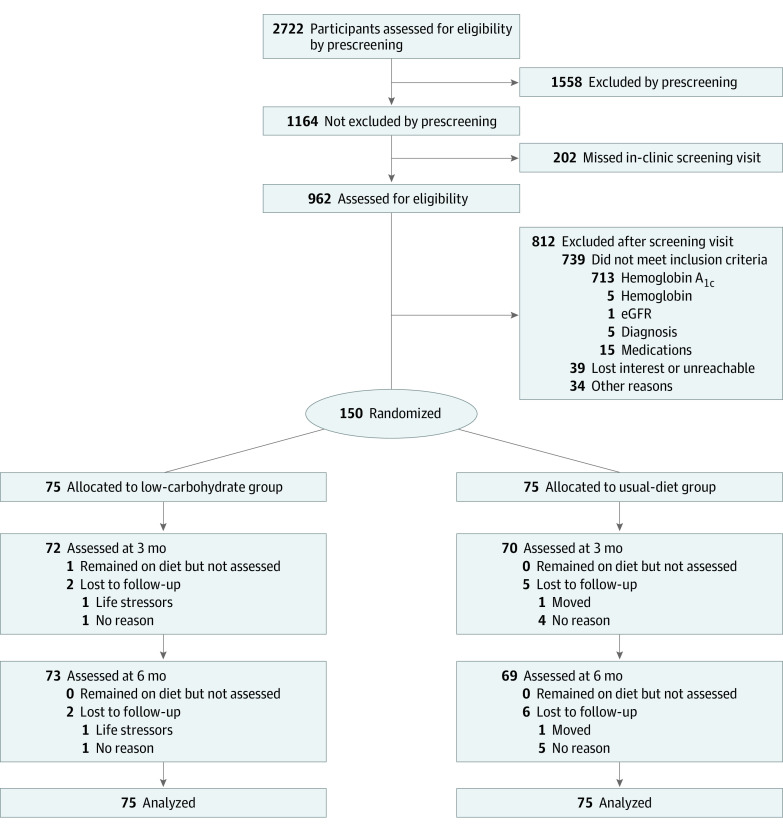
Study Flow Diagram eGFR indicates estimated glomerular filtration rate.

This was a 6-month, parallel-group, randomized controlled superiority trial (Figure 1). Participants were allocated to a group by randomized sequence generated by SAS statistical software version 9.4 (SAS Institute) before the study started by staff not otherwise involved in the study. After ascertaining eligibility, staff obtained randomization assignment in either a sealed envelope from or by telephoning the methodology unit at the time of randomization. Randomization was stratified by sex, using random block group size of 4 and 6.

#### Low-Carbohydrate Dietary Intervention Group

Participants received behavioral counseling and key supplemental food. Phase 1 (Go Low) lasted for the first 3 months (target <40 g net carbohydrates per day). During phase 2 (Keep it Low; months 4 through 6), net carbohydrate goal was less than 60 g (participants were instructed to choose the lowest feasible target). The goal of the 2-phased intervention was to allow participants to see how the less than 40 g target felt and, then, if they chose to, see how they responded to small increases in carbohydrate consumption.^21^ The less than 40 g target was chosen as this and lower targets have been shown to be safe and lead to cardiometabolic benefits in other studies, and less than 50 g per day can induce nutritional ketosis.^21,22,23^ There was no explicit weight loss goal.

Participants received a handbook with dietary guidelines and recipes.^22^ The interventionist instructed participants to reduce net carbohydrate intake and emphasized consuming key foods. Additional intervention details are described in eAppendix in Supplement 2.

The Go Low phase involved weekly individual sessions for the first 4 weeks, followed by 4 small group sessions held every other week and 4 telephone follow-ups. During Keep it Low, participants attended 3 monthly group sessions and had 3 telephone follow-ups. Starting in March 2020, most sessions were moved to videoconference.

#### Usual Diet Group 

At randomization, participants received written information with standard dietary advice^24^; they did not receive ongoing recommendations. Participants were offered optional monthly educational sessions on topics unrelated to diet.

#### Physical Activity

At baseline, participants in both groups received the same written information on standard physical activity. The recommendations were from the US Department of Health and Human Services.^25^

### Data Collection

Data collection at baseline, 3-month, and 6-month visits included standard questionnaires.^26^ Participants self-reported demographic information via questionnaire. Questionnaires specified race and ethnicity choices, but participants could choose “other” and self-specify their race. Race and ethnicity were assessed in this study to allow us to evaluate the distribution of race and ethnicity in the trial and to allow for potential subgroup analyses by race and ethnicity. Height was measured using stadiometer and weight by calibrated digital scale. Waist circumference was measured at 1 cm above the top of the navel. Three blood pressure (BP) measurements were obtained with an automated BP monitor after participants rested quietly for at least 5 minutes. The mean of 3 BP measurements was used.

Blood samples after overnight fast were collected and stored at −80 °C. Laboratory analysts were blinded to participant assignment. HbA_1c_ was measured at a local laboratory to assess eligibility. For outcome analyses, HbA_1c_ was measured at the Diabetes Diagnostic Laboratory (ion-exchange method; Tosoh G8 HPLC Analyzer). All other biomarkers were measured at Tulane University.^19^ The pooled cohort equations was used to estimate 10-year risk of atherosclerotic cardiovascular disease (ASCVD).^27^

We obtained two 24-hour dietary recalls from participants at baseline, 3 months, and 6 months. For each time point, 1 recall reflected weekday and the other weekend day consumption. The Nutrition Data System for Research software version 2020 (Nutrition Coordinating Center) was used to collect recalls and calculate dietary nutrient intake. We also measured ketones in spot urine collected at baseline and follow-up visits; 24-hour glucose levels were measured using a continuous glucose monitor (Abbott FreeStyle Libre Pro) over 14 days before the 6-month visit in a subgroup of 59 participants.^28^

### Power and Sample Size

On the basis of the net 6-month reduction observed in the lifestyle vs control group in the DPP trial, we deemed a 0.17% net reduction in HbA_1c_ over 6 months as clinically meaningful.^5^ Pilot data suggested a 0.35% SD of 6-month net change in HbA_1c_. Therefore, with 150 participants, we had 80% statistical power at 2-sided significance level of *P* < .05 to detect a 0.17% net reduction in HbA_1c_ with 95% follow-up.

### Statistical Analysis

Data analysis was performed from November 2021 to September 2022 using SAS statistical software version 9.4 (SAS Institute) and R statistical software version 4.1.0 (R Project for Statistical Computing). Data were analyzed using intention to treat. For the primary outcome (net 6-month change in HbA_1c_), we used linear mixed effects models with HbA_1c_ as the dependent variable. This model included a random intercept with an unstructured covariance matrix, indicator variables for time and study group, and group by time interaction terms. We used the same approach for secondary outcomes and exploratory outcomes. In post hoc analyses, we used a χ^2^ test to compare percentage of participants with HbA_1c_ less than 6.0% at 6 months between groups. To examine urinary ketones and reported symptoms, we used generalized estimating equations with exchangeable covariance matrix and logit link.

A 2-sided *P* < .05 was considered significant. We made no adjustment for multiple comparisons for secondary or exploratory outcomes. For subgroup analyses, we adjusted for multiple comparisons using the Bonferroni correction; a 2-sided *P* = .017 (0.05/3 subgroup analyses) was considered significant. Supplemental methods describing sensitivity, subgroup, and continuous glucose monitor analyses are included in the eAppendix in Supplement 2.

## Results

Of 2722 prescreened participants, 962 underwent screening. A total of 150 participants (mean [SD] age, 58.9 [7.9] years; 108 women [72%]; 88 Black [59%]) were enrolled (Table 1). The mean (SD) HbA_1c_ was 6.16% (0.30%) at baseline, and 130 patients (87%) had untreated HbA_1c _less than 6.5%. The mean (SD) body mass index (calculated as weight in kilograms divided by height in meters squared) was 35.9 (6.7). Most characteristics were similar by group, though there was a higher proportion of White participants and fasting plasma glucose was higher in the low-carbohydrate diet intervention than usual diet group (Table 1). A total of 142 participants (95%) completed 6-month data collection.

**Table 1.** Baseline Characteristics of Trial Participants

Characteristic	Participants, No. (%)		
	Low-carbohydrate diet (n = 75)	Usual diet (n = 75)	Total (N = 150)
Age, mean (SD), y	59.3 (7.0)	58.6 (8.8)	58.9 (7.9)
Sex			
Female	54 (72)	54 (72)	108 (72)
Male	21 (28)	21 (28)	42 (28)
Race and ethnicity			
Black	39 (52)	49 (65)	88 (59)
Hispanic	7 (9)	4 (5)	11 (7)
White	37 (49)	24 (32)	61 (41)
Other^a^	4 (5)	2 (3)	6 (4)
College degree or higher education	48 (64)	57 (76)	105 (70)
Body weight, mean (SD), kg	102.6 (21.8)	96.4 (19.6)	99.5 (20.9)
Body mass index, mean (SD)^b^	36.6 (7.2)	35.3 (6.0)	35.9 (6.7)
Waist circumference, mean (SD), cm	115.9 (14.8)	111.8 (15.0)	113.9 (15.0)
Systolic blood pressure, mean (SD), mm Hg	123.4 (12.8)	125.2 (15.4)	124.3 (14.1)
Diastolic blood pressure, mean (SD), mm Hg	75.9 (8.1)	77.9 (11.0)	76.9 (9.7)
Hemoglobin A_1c_, mean (SD), %	6.17 (0.31)	6.14 (0.30)	6.16 (0.30)
Hemoglobin A_1c_ <6.5%	65 (87)	65 (87)	130 (87)
Fasting plasma glucose, mean (SD), mg/dL	108.3 (18.1)	101.2 (13.3)	104.7 (16.2)
Insulin, mean (SD), μIU/L	30.9 (13.8)	29.3 (16.3)	30.1 (15.1)
Homeostasis model assessment of insulin resistance, mean (SD)	8.4 (4.2)	7.4 (4.3)	7.9 (4.3)
Total cholesterol, mean (SD), mg/dL	173.9 (28.7)	183.8 (35.2)	178.8 (32.4)
HDL cholesterol, mean (SD), mg/dL	49.6 (12.5)	50.9 (12.2)	50.3 (12.3)
Low-density lipoprotein cholesterol, mean (SD), mg/dL	101.7 (25.3)	110.3 (31.3)	106.0 (28.7)
Total-to-HDL cholesterol ratio, mean (SD)	3.7 (1.0)	3.8 (0.9)	3.7 (1.0)
Triglyceride level, mean (SD), mg/dL	123.7 (61.3)	115.5 (55.2)	119.6 (58.3)
Antihypertensive medication use	47 (63)	51 (68)	98 (65)
Lipid-lowering medication use	27 (36)	27 (36)	54 (36)
Current smoker	7 (9)	2 (3)	9 (6)
Physical activity level, median (IQR), metabolic equivalent-h/wk	19.6 (8.3-39.0)	14.9 (8.3-37.2)	17.6 (8.3-37.7)
10-y Atherosclerotic cardiovascular disease risk score, mean (SD), %	7.7 (4.9)	8.4 (6.7)	8.0 (5.9)

Abbreviation: HDL, high-density lipoprotein.

SI conversion factors: To convert glucose to mmol/L, multiply by 0.0555; insulin to pmol/L, multiply by 6.945; total cholesterol to mmol/L, multiply by 0.0259; HDL cholesterol to mmol/L, multiply by 0.0259; low-density lipoprotein cholesterol to mmol/L, multiply by 0.0259; triglycerides to mmol/L, multiply by 0.0113.

^a^ Other includes Asian, Native Hawaiian or Pacific Islander, Native American or Alaska Native, or participant-specified.

^b^ Body mass index is calculated as weight in kilograms divided by height in meters squared.

### Dietary Intake and Physical Activity

At baseline, reported dietary compositions were similar between groups (Table 2). During follow-up, total calorie intake was lower in the intervention than usual diet group (net difference in change at 3 months, –389 kcal; 95% CI, –613 to –166 kcal; *P* < .001; net difference in change at 6 months, –456 kcal; 95% CI, –689 to –224 kcal; *P* < .001). Intakes of total and net carbohydrates, added sugars, and sugar-sweetened beverages were lower in the intervention group at follow-up, as was daily glycemic load; percentages of calories from protein and monounsaturated and polyunsaturated fats were higher at follow-up, though total intake for monounsaturated and polyunsaturated fats was higher only at 3 months. At 3 months, 9 participants (13%) in the intervention group and 2 participants (3%) in the usual diet group had detectable urinary ketones. At 6 months, 4 participants (6%) in the intervention group and 3 participants (4%) in the usual diet group had detectable ketones. At 6 months, median (IQR) physical activity was 27.0 (9.8-71.9) metabolic equivalent hours per week in the intervention group and 15.7 (7.7-32.3) metabolic equivalent hours per week in the usual diet group (eTable 1 in Supplement 2).

**Table 2.** Daily Dietary Composition in the Low-Carbohydrate and Usual Diet Groups Over the Course of the Study^a^

Variable	Mean (SD)					
	Baseline		3 mo		6 mo	
	Low-carbohydrate (n = 75)	Usual diet (n = 75)	Low-carbohydrate (n = 73)	Usual diet (n = 69)	Low-carbohydrate (n = 73)	Usual diet (n = 69)
Total calories, kcal	1890 (775)	1789 (812)	1447 (507)	1701 (729)	1439 (500)	1757 (645)
Total carbohydrates, g	207 (98)	190 (86)	89 (50)	177 (73)	96 (52)	187 (73)
Total fiber, g	17 (12)	16 (9)	20 (13)	14 (7)	18 (12)	17 (9)
Net carbohydrates, g^a^	190 (91)	174 (81)	66 (49)	162 (70)	75 (50)	170 (68)
Protein, g	81 (45)	76 (38)	90 (37)	74 (48)	85 (33)	74 (34)
Total fat, g	81 (40)	79 (41)	84 (36)	73 (34)	82 (37)	77 (36)
SFA, g	28 (16)	24 (13)	25 (12)	24 (13)	25 (13)	24 (12)
MUFA, g	29 (16)	29 (19)	31 (16)	26 (12)	30 (14)	29 (15)
PUFA, g	18 (9)	18 (11)	21 (10)	16 (9)	20 (11)	18 (10)
Carbohydrates, % kcal	44 (10)	42 (9)	23 (11)	42 (11)	25 (12)	42 (9)
Protein, % kcal	17 (5)	18 (5)	25 (6)	18 (6)	24 (7)	17 (6)
Fat, % kcal	37 (8)	38 (8)	50 (9)	37 (10)	48 (10)	38 (8)
SFA, % kcal	12 (4)	12 (3)	15 (4)	12 (4)	15 (4)	12 (4)
MUFA, % kcal	13 (4)	14 (4)	18 (5)	13 (4)	18 (5)	14 (4)
PUFA, % kcal	8 (3)	9 (3)	12 (5)	8 (3)	12 (4)	9 (3)
Glycemic load, median (IQR), glucose reference	107 (81-138)	96 (69-136)	26 (16-49)	91 (68-115)	34 (18-52)	95 (77-124)
Added sugars, median (IQR), g	64 (28-80)	43 (24-80)	9 (4-18)	40 (23-59)	11 (5-31)	45 (22-66)
Sugar-sweetened beverages, median (IQR), servings	0.38 (0.00-1.48)	0.38 (0.00-1.25)	0.00	0.00 (0.00-0.75)	0.00	0 (0-1.00)

Abbreviations: MUFA, monounsaturated fatty acid; PUFA, polyunsaturated fatty acid; SFA, saturated fatty acid.

^a^ Calculated as total carbohydrates minus fiber.

### Primary Outcome and Secondary Outcomes

**Figure 2.**
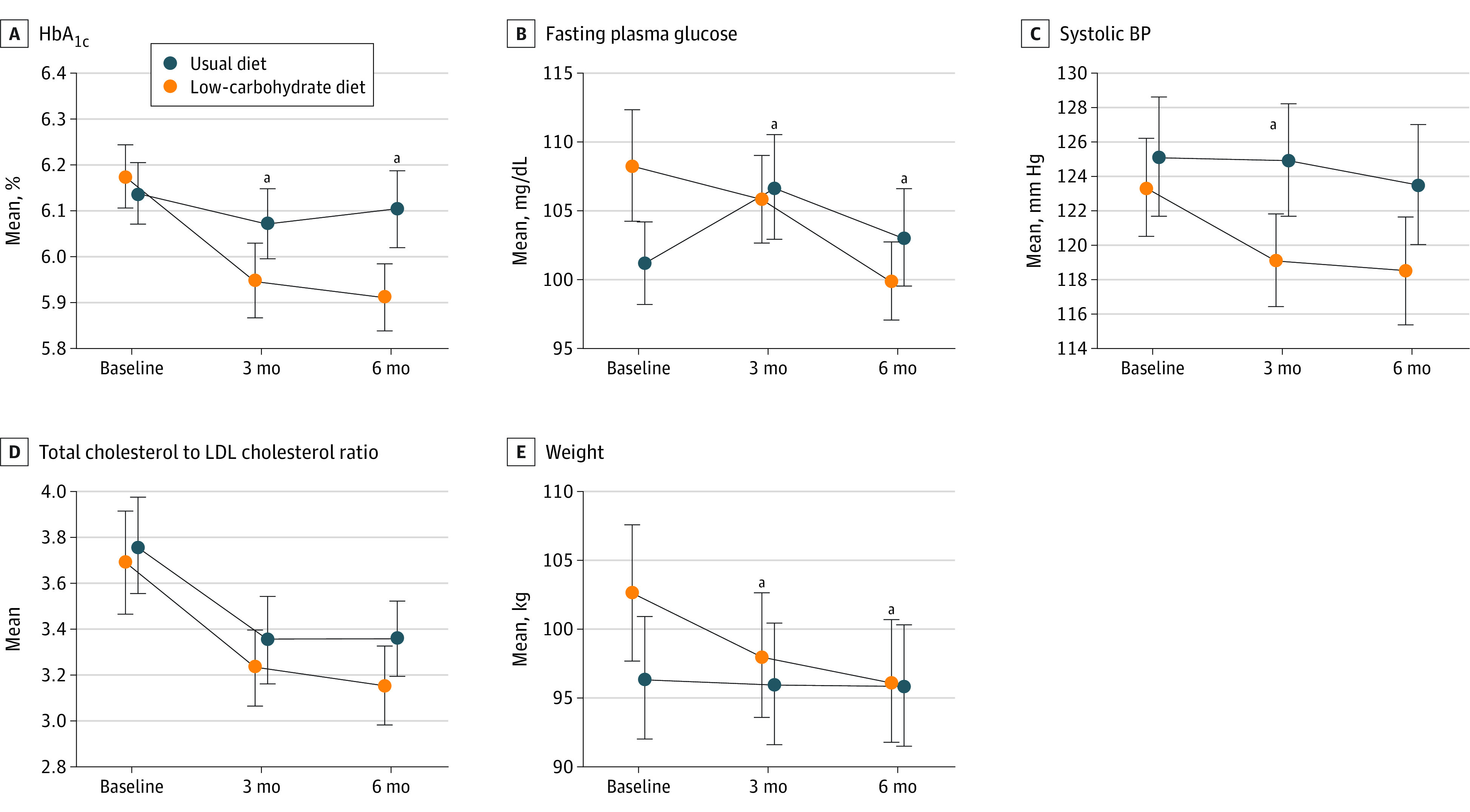
Mean Estimated Primary and Secondary Outcomes ^a^*P* < .05 for between-group net change from baseline.

For the primary outcome, participants in the low-carbohydrate diet intervention group had larger decrease in HbA_1c_ from baseline to 6 months (–0.26%; 95% CI, –0.33% to –0.19%) than those in the usual diet group (–0.04%; 95% CI, –0.10% to –0.02%) with a net difference in change of –0.23% (95% CI, –0.32% to –0.14%; *P* < .001) (Table 3 and Figure 2). The decrease in HbA_1c_ from baseline to 3 months was larger in the low-carbohydrate diet intervention than usual diet group. There was significantly greater 6-month decrease in fasting plasma glucose (net difference in change, –10.3 mg/dL; 95% CI, –15.6 to –4.9 mg/dL; *P* < .001; to convert to mmol/L, multiply by 0.0555) and body weight (net difference in change, –5.9 kg; 95% CI, –7.4 to –4.4 kg; *P* < .001) in the intervention than usual diet group (Table 3 and Figure 2). The decrease in systolic BP from baseline to 3 months was significantly greater only in the intervention than usual diet group. There was no significant net difference in change in total-to-high-density lipoprotein (HDL) cholesterol between groups.

**Table 3.** Change in Metabolic Risk Factors From Baseline, Within and Between Groups

Variable	Change from baseline (95% CI)		Difference in change from baseline, intervention vs control group (95% CI)	*P* value
	Low-carbohydrate diet (n = 75)	Usual diet (n = 75)		
Primary and secondary outcomes				
Hemoglobin A_1c_, %				
3 mo	–0.23 (–0.31 to –0.15)	–0.07 (–0.11 to –0.02)	–0.16 (–0.25 to –0.07)	<.001
6 mo^a^	–0.26 (–0.33 to –0.19)	–0.04 (–0.10 to 0.02)	–0.23 (–0.32 to –0.14)	<.001
Fasting plasma glucose, mg/dL				
3 mo	–2.4 (–6.3 to 1.5)	5.6 (1.3 to 9.8)	–8.0 (–13.7 to –2.2)	.007
6 mo	–8.4 (–12.3 to –4.5)	1.9 (–1.8 to 5.5)	–10.3 (–15.6 to –4.9)	.001
Systolic blood pressure, mm Hg				
3 mo	–4.2 (–6.7 to –1.7)	–0.2 (–2.6 to 2.2)	–4.0 (–7.5 to –0.5)	.03
6 mo	–4.9 (–7.9 to –1.8)	–1.6 (–4.4 to 1.2)	–3.2 (–7.3 to 0.9)	.12
Total-to-HDL cholesterol, mg/dL				
3 mo	–0.46 (–0.59 to –0.33)	–0.41 (–0.52 to –0.30)	–0.05 (–0.22 to 0.12)	.57
6 mo	–0.54 (–0.67 to –0.40)	–0.41 (–0.52 to –0.29)	–0.13 (–0.31 to 0.05)	.15
Body weight, kg				
3 mo	–4.6 (–5.5 to –3.6)	–0.4 (–0.9 to 0.1)	–4.1 (–5.2 to –3.1)	<.001
6 mo	–6.4 (–7.8 to –5.1)	–0.5 (–1.2 to 0.2)	–5.9 (–7.4 to –4.4)	<.001
Exploratory outcomes				
Fasting insulin, μIU/L				
3 mo	–4.1 (–6.9 to –1.3)	1.5 (–1.0 to 4.0)	–5.6 (–9.4 to –1.8)	.004
6 mo	–4.0 (–7.2 to –0.8)	2.3 (–0.5 to 5.0)	–6.2 (–10.5 to –2.0)	.004
Homeostasis model assessment of insulin resistance				
3 mo	–1.2 (–2.1 to –0.2)	0.9 (0 to 1.8)	–2.0 (–3.3 to –0.7)	.002
6 mo	–1.6 (–2.6 to –0.7)	0.8 (–0.2 to 1.7)	–2.4 (–3.7 to –1.1)	<.001
Diastolic blood pressure, mm Hg				
3 mo	–3.2 (–4.8 to –1.7)	–0.1 (–1.9 to 1.8)	–3.2 (–5.6 to –0.8)	.01
6 mo	–3.2 (–5.2 to –1.2)	–0.6 (–2.3 to 1.1)	–2.6 (–5.2 to 0)	.05
Waist circumference, cm				
3 mo	–3.0 (–4.3 to –1.7)	–0.5 (–1.2 to 0.3)	–2.5 (–4.0 to –1.0)	.001
6 mo	–5.2 (–6.9 to –3.5)	–0.5 (–1.6 to 0.5)	–4.7 (–6.7 to –2.6)	<.001
10-y Atherosclerotic cardiovascular disease risk, %				
3 mo	–1.2 (–2.1 to –0.4)	–0.4 (–1.0 to 0.1)	–0.8 (–1.8 to 0.2)	.11
6 mo	–1.7 (–2.5 to –0.9)	–0.9 (–1.6 to –0.2)	–0.8 (–1.8 to 0.3)	.14
HDL, mg/dL^b^				
3 mo	3.1 (1.2 to 4.9)	3.0 (1.6 to 4.4)	0.1 (–2.2 to 2.4)	.94
6 mo	5.2 (3.5 to 7.0)	3.4 (1.7 to 5.1)	1.9 (–0.5 to 4.3)	.13
Low-density lipoprotein, mg/dL^b^				
3 mo	5.8 (2.4 to 9.3)	2.9 (–1.6 to 7.5)	2.9 (–2.8 to 8.6)	.32
6 mo	3.2 (–0.8 to 7.1)	4.2 (–0.6 to 9.0)	–1.1 (–7.3 to 5.2)	.74

Abbreviation: HDL, high-density lipoprotein.

SI conversion factors: To convert glucose to mmol/L, multiply by 0.0555; insulin to pmol/L, multiply by 6.945; total cholesterol to mmol/L, multiply by 0.0259; HDL cholesterol to mmol/L, multiply by 0.0259; low-density lipoprotein cholesterol to mmol/L, multiply by 0.0259; triglycerides to mmol/L, multiply by 0.0113.

^a^ Primary outcome is 6-month change in hemoglobin A_1c_.

^b^ Refers to post hoc exploratory outcomes.

### Exploratory Outcomes

Six-month decreases in fasting insulin, homeostasis model assessment of insulin resistance (HOMA-IR), and waist circumference were significantly greater in the intervention than usual diet group (Table 3). There were no significant net differences in 6-month changes for diastolic BP, HDL, low-density lipoprotein, or estimated 10-year ASCVD risk. Mean 24-hour glucose was –7.0 mg/dL lower (95% CI, –13.8 to –0.1 mg/dL) in the intervention than usual diet group at 6 months, as was mean night-time glucose (–9.7 mg/dL; 95% CI, –17.7 to –1.7 mg/dL) (eTable 2 in Supplement 2). Glycemic variability assessed by SD and coefficient of variation did not differ by group. The intervention group spent 9.8% more time (95% CI, 1.6% to 18.0%) in the range of 70 to 120 mg/dL than the usual diet group (eTable 2 in Supplement 2). Percentage of time in the ranges of 70 to 140 mg/dL or 70 to 180 mg/dL did not differ by group. At 6 months, 39 participants (53%) in the intervention group and 22 participants (32%) in the usual diet group had HbA_1c_ less than 6.0% (χ^2^_1_ = 6.72; *P* = .01).

### Sensitivity Analyses

In models adjusting for baseline outcome and selected baseline covariates, patterns were similar, though some findings were attenuated (eTable 3 in Supplement 2). The 6-month adjusted net difference in HbA_1c_ was –0.17% (95% CI, –0.25% to –0.09%), and the adjusted net difference in fasting plasma glucose was –5.4 mg/dL (95% CI, –9.9 to –0.9 mg/dL). Multiple imputation yielded similar results to main analyses (eTable 4 in Supplement 2).

### Subgroup Analyses

Directions of effects were consistent among subgroups. There were significant interactions in 6-month net difference in HbA_1c_ by race and by sex (eTable 5 in Supplement 2). The 6-month net difference in HbA_1c_ change was –0.10% in Black participants and –0.36% in White participants (*P* for interaction = .003). The 6-month net difference in HbA_1c_ was –0.14% in women and –0.41% in men (*P* for interaction = .006). There was no interaction by enrollment before March 2020 vs July 2020 or later (*P* for interaction = .24) (eTable 5 in Supplement 2).

### Adverse Events and Symptoms

Numbers of adverse events were similar between groups (eTable 6 in Supplement 2). Significantly more participants in the intervention group reported muscle cramps at 3 months (35% [95% CI, 11%-46%] vs 19% [11%-29%] of participants; *P* = .03) and 6 months (34% [95% CI, 11%-46%] vs 19% [11%-30%]; *P* = .04) (eTable 7 in Supplement 2).

## Discussion

In this randomized clinical trial, a behavioral intervention promoting a healthy low-carbohydrate diet led to greater 6-month reductions in HbA_1c_ than usual diet among adults with untreated HbA_1c_ of 6.0% to 6.9%. Although the mean observed HbA_1c_ reduction of 0.23% was modest, this reduction was similar to 6-month HbA_1c_ reduction of 0.17% in the lifestyle compared with control group in the DPP trial,^5^ which promoted a low-fat, low-calorie diet, moderate activity, and weight loss and yielded a 58% T2D risk reduction over an average of 2.8 years. Compared with usual diet, the low-carbohydrate diet intervention also led to greater 6-month reductions in fasting plasma glucose, body weight, fasting insulin, HOMA-IR, and waist circumference. Because of the study design, we are unable to determine the effects of reducing carbohydrate intake independently of caloric restriction and weight loss. We found beneficial direction of effects on 6-month HbA_1c_ change in subgroups, although the effect size appeared larger in White than Black participants and in men than women.

In contrast to prior work^14,15^ on low-carbohydrate diet interventions and HbA_1c_, a substantial proportion of participants in the current study (87%) had untreated HbA_1c _less than 6.5%. Unlike most prior studies in T2D, no participants were taking glucose-lowering medications at randomization, making it feasible to assess the intervention’s effect on HbA_1c_ without confounding by medications. If patients taking glucose-lowering medications alter their diet, health care professionals should monitor and adjust medications to reduce hypoglycemia risk.^29,30^

Compared with the usual diet group, the low-carbohydrate diet intervention group had 6-month HbA_1c_ decrease of 0.23%. Although this is a modest decrease for people with T2D, it is similar to that observed in the DPP trial, which had clinically meaningful reductions in T2D risk.^5^ Similar to prior trials^11,13^ in individuals with and without T2D, the intervention led to a 6-month decrease in body weight, waist circumference, fasting insulin, and HOMA-IR. Prior work^11,22^ has also observed improvements in BP, lipids, and estimated 10-year ASCVD risk among individuals on a low-carbohydrate diet. Within the low-carbohydrate diet intervention group, there was significant improvement in systolic BP, total-to-HDL cholesterol, diastolic BP, 10-year ASCVD risk, and HDL over 6 months, but this improvement did not significantly differ from the usual diet group, potentially because of lack of power.

There were significant decreases in caloric intake in the low-carbohydrate diet intervention group during follow-up, aligning with large observed weight loss. With this study design, we are unable to conduct an isocaloric comparison between the low-carbohydrate and usual diet groups or to determine effects on HbA_1c_ independently of weight loss. Few participants had detectable urinary ketones, suggesting ketosis was unlikely to account for the findings. Prior research^31^ of a similar intervention suggests that satiety may be better preserved in a low-carbohydrate than low-fat diet. Participants in this study had a mean body mass index of 35.9 at baseline; if glycemic reductions were primarily mediated by weight loss, the magnitude of glycemic effects may be smaller in populations with lower body mass index.^32^ Future research should explore contributions of weight loss as a mediator to dietary-induced reductions in glycemia in populations with different metabolic characteristics.^32^

The continuous glucose monitor finding of significantly lower mean 24-hour glucose and higher percentage of time in the range of 70 to 120 mg/dL in the low-carbohydrate diet intervention than usual diet group at 6 months is consistent with the primary outcome findings. These novel exploratory findings should be investigated further in future research.

Directions of results were consistent in Black and White participants, though the effect size was larger among White participants. There is evidence that at similar levels of glucose-based markers, HbA_1c_ may be higher among Black than White individuals.^33,34^ If Black participants had lower average glucose than White participants at the same HbA_1c_ level, that could potentially explain the smaller effect size. The effect size was larger in men than women. Prior work has found evidence of a greater effect of weight loss interventions in men than women.^35^ These post hoc analyses should be interpreted with caution.

### Strengths and Limitations

Our study has strengths. First, more than half of participants were Black, a group underrepresented in much dietary intervention research. We observed beneficial directions of effects in Black and White participants. Second, there were high follow-up rates and adherence. Third, trained and certified staff collected all data and followed quality control protocols. Fourth, laboratory personnel measuring outcomes were blinded to study allocation. Fifth, the findings were robust in sensitivity analyses and in continuous glucose monitor analyses. The novel continuous glucose monitor findings highlight the utility of this technology for obtaining a more complete picture of glycemia in response to dietary interventions in people with untreated HbA_1c_ in this range.

Our study also has limitations. First, self-report of dietary intake is subject to potential recall issues. We addressed this by collecting 24-hour recalls at each time point, reflecting both weekend and weekday intake, and by measuring urinary ketones, a more objective adherence measure.^36^ Second, participants in the low-carbohydrate group had frequent interventionist interactions, whereas the usual diet group did not. To increase study engagement, participants in the usual diet group were offered monthly sessions on topics unrelated to diet or health, though the more frequent interactions in the intervention group could have affected adherence and results. Third, we observed large calorie reduction and weight loss in the intervention group and were unable to evaluate the intervention’s effect on HbA_1c_ independently of calorie restriction and weight loss. Fourth, we were unable to evaluate longer-term intervention adherence or effects and did not formally collect information on participants’ long-term diet adherence plans. Fifth, because of the limited sample size and follow-up, we were unable to evaluate T2D progression, though in post hoc analyses the proportion of participants with HbA_1c _less than 6.0% at 6 months differed by group. Sixth, we did not collect data on body composition. Seventh, as this is an efficacy trial of a moderately intensive intervention with supplemental food, the findings may not be generalizable to settings in which intensive dietary counseling is not accessible; future research should explore low-carbohydrate diet effectiveness in lowering glycemic outcomes in such settings.

## Conclusions

In this 6-month randomized clinical trial, a low-carbohydrate diet intervention led to larger reductions in HbA_1c_ than usual diet among adults with elevated untreated HbA_1c_ (6.0–6.9%), though we were unable to assess its effects independently of weight loss. This dietary approach may be an option for people with or at high risk of T2D to improve glycemic and other markers and should be studied further and over longer time periods in other populations and settings.

## Notes

Supplement 1.
Trial Protocol

Supplement 2. **eAppendix.** Supplemental Methods
eReferences
**eTable 1.** Self-Reported Median Physical Activity Levels Over Time by Assigned Arm **eTable 2.** Mean Differences in Continuous Glucose Monitor Outcomes at 6 Months, by Assigned Arm, Among Those With at Least 10 Days of Full 24-h Data **eTable 3.** Change in Metabolic Risk Factors From Baseline, Within and Between Arms, After Adjusting for Baseline Level of Outcome, Race and Baseline Weight, Waist Circumference, Fasting Glucose, LDL Cholesterol, Total Cholesterol, and Physical Activity **eTable 4.** Change in Metabolic Risk Factors From Baseline, Within and Between Arms, by Assigned Arm After Multiple Imputation **eTable 5.** Subgroup Analyses of Change in Metabolic Risk Factors From Baseline, Within and Between Arms **eTable 6.** Total Number of Times Any Adverse Event Was Reported **eTable 7.** Symptoms Reported by Participants: Percent of Participants in Each Arm (95% CI) Reporting Symptoms at 3 Months and 6 Months

Supplement 3.
Data Sharing Statement
