# Supplementary material for: Effects of a Low-Carbohydrate Dietary Intervention on Hemoglobin A1c: A Randomized Clinical Trial
Source: JAMA Netw Open. 2022 Oct 26;5(10):e2238645. doi: 10.1001/jamanetworkopen.2022.38645 (PMC9606840; doi:10.1001/jamanetworkopen.2022.38645)
Supplement: Supplement 1. — Trial Protocol [file jamanetwopen-e2238645-s001.pdf]

Supplemental File: Protocol

**Version**	**Date**	**Change**
V1.1 (Pre-Enrollment)	August 20, 2018	
V1.2	October 8, 2018	Addition of supplemental visit for participants who are prescribed diabetes medication during the study period, in order to collect fasting blood sample prior to starting medication.
V1.3	December 17, 2018	Additional recruitment approaches included.
V1.4	March 18, 2019	Additional recruitment approaches included.
V1.5	May 5, 2019	Added 3rd stool sample collection to 3 month visit.
V1.6	August 20, 2019	Added language to protocol to allow for follow-up blood work to be completed at a local LabCorp for subjects who move during study participation.
V1.7	October 14, 2019	Additional recruitment approaches included (to allow emailing recruitment letter).
V1.8	November 14, 2019	Adding continuous glucose monitor data collection to 6-month visit.
V1.9	December 3, 2019	Adding additional exclusion criteria for those participating in the continuous glucose monitoring data collection at 6 months (added prior to enrolment of any participants in that data collection)
V2.0	February 10, 2020	Increase in sample size of total subjects from 112 to 126, due to external advisory committee feedback regarding concerns with having sufficient statistical power. This change was approved of by the Data and Safety Monitoring Board.
V2.1	June 26, 2020	Added exclusion criteria of COVID-19 ICU stay in the past 3 months, as COVID-19 may affect blood glucose levels.
V2.2	September 14, 2020	Increase in sample size of total subjects from 126 to 150, due to concerns related to statistical power. This change was approved of by the Data and Safety Monitoring Board.

A Clinical Trial of Low-Carbohydrate Dietary Pattern on Glycemic Outcomes

PROTOCOL

PROTOCOL VERSION 1.1

Dated: August 20, 2018

New Orleans, LA

Table of Contents

1. Overview 5
2. Background and Significance 6
2.A. Type 2 Diabetes Mellitus is a major global public health challenge 6
2.B. Prediabetes is a major risk factor for cardiometabolic diseases 6
2.C. Role of lifestyle in preventing diabetes 6
2.D. Low-to-moderate carbohydrate diets, weight loss, and cardiometabolic diseases 7
2.E. Mediterranean-style diet is associated with reduced risk of T2D and improved glycemic control 8
2.F. Evidence for low-carbohydrate diets that emphasize Mediterranean-style dietary components 8
2.G. Evidence for specific food groups and risk of T2D 8
2.H. Gut microbiota, diet and T2D 9
2.I. Clinical and public health significance of the proposed study 9
2.J. Innovation 9
3. Preliminary Studies 10
3.A. Summary of aggregate experience of the research team 10
3.B. Low-carbohydrate behavioral dietary intervention trial experience 10
3.C. Other dietary intervention trial experience 11
4. Study Design and Organization 11
4.A. Overview of trial design 11
4.B. Study outcomes 12
4.B.1. Primary outcome 12
4.B.2. Secondary outcomes 12
4.C Study Timeline 12
6. Study Participants 13
6.A. Eligibility criteria 13
7. Recruitment and Randomization 13
7.A. Recruitment 13
7.A.1. Mass mailing 14
7.A.2. Database searches and targeted mailing 14
7.A.3. Referrals 14
7.B. Randomization 14
7.C. Masking 14
7.D. Safety Considerations 15

8. Dietary Intervention Program 15
8.A. Low-carbohydrate dietary intervention group 15
8.B. Usual Diet Group 16
8.C. Physical Activity 16
8.D. Dietary Adherence 16
9. Safety of Dietary Intervention 16
10. Data Collection 17
10.A. Study visits 17
10.A.1. Screening/baseline visits 18
10.A.2. Follow-up visits and final visit 18
10.B Study measurements 19
10.B.1. Questionnaires 19
10.B.2. Dietary measurements 19
10.B.3. Assessment and management of adverse effects 19
10.B.4. BP and anthropometric measures 19
10.B.5. Blood samples 19
10.B.6. Urine collection 20
10.B.7. Stool Specimen Collection 20
11. Quality Assurance and Quality Control 20
11.A. Manual of procedures 20
11.B. Training and certification 20
12. Sample Size and Statistical Power 21
13. Data Management and Analysis 22
13.A. Data management 22
13.B. Data analysis plan 22
13.B.1. Exploratory data analysis 22
13.B.2. Primary,secondary, and exploratory outcomes 22
15. Anticipated Challenges 22
16. Protection of Human Subjects 23
16.A. Protection of human subjects from research risks 23
16.A.1. Risks to the subjects 23
16.A.2. Adequacy of protection against risks 24
16.A.3. Potential benefits of the proposed research to human subjects and others 25

16.A.4. Importance of knowledge to be gained 26
16.A.5. Remuneration 26
16.B. Data safety and monitoring plan 26
Bibliography and References Cited 28

1. Overview

The increasing prevalence of type 2 diabetes mellitus (T2D), a major cause of morbidity and mortality worldwide, is a critical public health concern. In the United States, an estimated 33.9% of adults have prediabetes (impaired fasting glucose, elevated hemoglobin A1c (HbA1c), or impaired glucose tolerance). Individuals with prediabetes are at elevated risk of T2D and cardiovascular disease (CVD), with 5–10% developing clinical T2D each year.

In the short-term, among patients with T2D, low-to-moderate carbohydrate diets (energy percentage below 45) have a greater glucose-lowering effect than do high-carbohydrate diets, with larger glucose-lowering effect the greater the carbohydrate reduction. Additionally, compared with low-fat diets, Mediterranean diets that are high in unsaturated fats (particularly *cis*-monounsaturated fats) reduce risk of T2D and CVD and improve glycemic control among diabetics. However, compared with usual diets, the effect of a behavioral intervention promoting a low-carbohydrate/high-unsaturated fat and protein dietary pattern that emphasizes consumption of non-starchy vegetables, plant-based oils, fish, poultry, and mixed nuts on glucose homeostasis, and other metabolic risk factors among individuals with prediabetes or early-stage untreated T2D is not well understood.

Our *long-term goal* is to develop and implement dietary intervention(s) that reduce risk of T2D. The *overall goal of this randomized controlled trial is to study the effect of a behavioral intervention promoting a low-carbohydrate/high-unsaturated fat and high-protein dietary pattern (initial target <40 g digestible carbohydrates, final target <60 g digestible carbohydrates) compared with a usual diet on metabolic risk factors among individuals with prediabetes or early-stage untreated diabetes* (HbA1c 6.0–6.9%). Specifically, we will test the following hypotheses:

Primary hypothesis: Compared with usual diet, over a 6-month period, a healthy low-carbohydrate/high unsaturated fat and high-protein dietary pattern will lead to *a larger decrease of HbA1c, by at least 0.17%.*

Secondary hypothesis: Over a 6-month period, a healthy low-carbohydrate/high-unsaturated fat and high-protein dietary pattern will lead to *larger improvements in the following major secondary outcomes*: fasting glucose, systolic blood pressure (BP), total-to-HDL-cholesterol ratio, and body weight.

Exploratory outcomes: We will also study the effect of the invention on the following outcomes: insulin and homeostasis model assessment of insulin resistance (HOMA-IR); diastolic BP; waist circumference; and estimated CVD risk.

To achieve these study aims, we will recruit 150 adults with prediabetes or untreated diabetes (HbA1c 6.0–6.9%) in the Greater New Orleans, LA area. Using a stratified, blocked randomization scheme, participants will be assigned to either a behavioral modification intervention aimed at promoting a healthy low-carbohydrate dietary pattern or to usual diet. The intervention group will have an intensive phase for 3 months, followed by a maintenance phase in months 4 through 6. The *primary outcome will be difference between the active intervention and control groups in the change in HbA1c from baseline to 6 months of follow-up. We expect 85% power to detect a difference of at least 0.17% in change of HbA1c.* Changes in other metabolic risk factors will be studied concurrently. Metabolic risk factors will be assessed at baseline, 3 months, and 6 months of follow-up.

There is much interest regarding dietary approaches to prevent T2D and its complications. The results from this study will help to determine whether, compared with usual diet, a behavioral intervention aimed at promoting a healthy low-carbohydrate dietary pattern improves metabolic risk factors.

2. Background and Significance

2.A. Type 2 Diabetes Mellitus is a major global public health challenge

Diabetes is a strong and independent risk factor for CVD morbidity and mortality. A meta-analysis of 102 prospective studies (698,782 participants) estimated diabetes leads to an approximately 2-fold increased risk in vascular diseases, including coronary heart disease (CHD) and major stroke subtypes, and deaths due to other vascular causes.[1] Among participants with diabetes, CHD risk is about 50% higher among those with fasting blood glucose ≥7 mmol/L compared with those with fasting blood glucose <7 mmol/L.[1] In addition to increasing CVD risk, diabetes can cause diabetic retinopathy,[2] chronic kidney disease,[3] lower limb amputation,[4] and other complications. Most individuals with diabetes have T2D.[5] The US Centers for Disease Control and Prevention (CDC) estimated that 30.2 million adults ≥18 years (12.2%) had diabetes in 2015, with 7.2 million (23.8%) not being aware of or reporting diabetes.[5] Diagnosed diabetes prevalence among US adults has increased substantially over time, from 2.54% in 1980 to 7.40% in 2015.[6] Diabetes prevalence is also high globally, with an estimated 2014 age-standardized prevalence of 9.0% in men and 7.9% in women.[7] Diabetes accounts for an increasing portion of US healthcare expenditures. Total cost of diagnosed diabetes in the US was estimated to be $245 billion in 2012, 41% higher than in 2007.[8] Diabetes is associated with much higher lifetime medical expenses, with higher expenses for those diagnosed at younger ages.[9]

2.B. Prediabetes is a major risk factor for cardiometabolic diseases

Prediabetes, defined by the American Diabetes Association (ADA) as impaired fasting glucose (IFG), impaired glucose tolerance (IGT), or elevated HbA1c, is also referred to as "intermediate hyperglycemia"[10] and "high-risk state of developing diabetes".[11] The ADA defines prediabetes as having: fasting plasma glucose of 100–125 mg/dL (IFG); 2-hour post-load glucose in the 75-g oral glucose tolerance test (OGTT) of 140–199 mg/dL (IGT); or HbA1c 5.7–6.4%.[12] Prediabetes itself is not a disease; for all three tests, "risk is continuous, extending below the lower limit of the range and becoming disproportionately greater at the higher end of the range".[12] The CDC estimated that in 2015, 33.9% of US adults had prediabetes based on fasting glucose or HbA1c.[5] Among those with prediabetes, only 11.9% reported having being told this by a medical professional.[5] Approximately 5–10% of individuals with prediabetes develop diabetes within one year, depending on how prediabetes is defined.[13] In a meta-analysis, annual diabetes incidence was 15–19% among individuals with both IFG and IGT, while it was 4–6% in those with isolated IFG and 6–9% in those with isolated IGT.[13]

A recent meta-analysis (1,611,339 participants from 53 studies) reported that prediabetes as defined by the ADA is associated with higher risk of composite CVD events and CHD.[14] IFG and IGT were also positively associated with increased risk of stroke and all-cause mortality.[14] Prediabetes is linked with increased risk of other conditions typically thought of as diabetes complications, such as neuropathies and nephropathies.[15] Importantly, it is possible for individuals with prediabetes to revert to normoglycemia, as observed in observational studies[13] and lifestyle or drug intervention trials.[16,17] In the Diabetes Prevention Program (DPP), reversion to normoglycemia was associated with lower long-term T2D risk[18] and lower predicted CVD risk.[19]

2.C. Role of lifestyle in preventing diabetes

Much evidence from observational studies and clinical trials supports the critical role of diet and other lifestyle factors in preventing T2D. Globally, lifestyle transitions towards reduced physical activity, increased consumption of refined carbohydrates, coupled with resulting changes in body composition, has led to increases in diabetes prevalence and severity.[20] Body

mass index (BMI), waist circumference, and waist-to-height ratio are predictors of T2D.[21] The DPP, which combined calorie restriction and exercise to promote weight loss, substantially reduced risk of T2D (by 58%) among individuals with elevated FPG and IGT;[22] a benefit that persisted in 10-year follow-up analyses.[16] Other trials of lifestyle modification focused on diet and exercise have also shown benefits in study populations in China,[23] Finland,[24] and India.[25]

2.D. Low-to-moderate carbohydrate diets, weight loss, and cardiometabolic diseases

Low-to-moderate carbohydrate diets (<45% of energy from carbohydrates) are popular dietary approaches for losing weight. Weight loss is a key component of the DPP lifestyle intervention[26] and ADA lifestyle recommendations.[12,27] Meta-analyses from our group[28] and others support that low-to-moderate carbohydrate diets are at least as effective as low-fat diets at promoting weight loss. For instance, a meta-analysis reported a 2-kg (95% CI, 3.1–0.9) larger weight loss in low-carbohydrate diet (<120 g carbohydrates/day) arms compared with low-fat diet (<30% energy from fat) arms.[29] Estimated 10-year CVD risk was lower in low-carbohydrate than low-fat arms.[29] This aligns with research published by our team; in a 12-month trial of participants with obesity, a low-carbohydrate diet (target <40 g carbohydrates/day) was more effective than a low-fat diet (target <30% energy from fat, <7% saturated fat) for weight loss and CVD risk factor reduction.[30]

Low-to-moderate carbohydrate diets may also be beneficial among patients with T2D. Low-carbohydrate diets have come in and out of favor as treatment of diabetes.[31] In a meta-analysis (1376 patients;10 trials), after 3 or 6 months of the intervention, low-to-moderate carbohydrate diets (<45% energy from carbohydrates) led to a 0.34% lower HbA1c (95% CI, 0.06–0.63%) compared with high-carbohydrate diets.[32] Compared with high-carbohydrate diets, there was a greater reduction in HbA1c the greater the reported carbohydrate restriction.[32] There was no benefit of the low-to-moderate carbohydrate diets on HbA1c at 12 months or longer, and no difference between interventions on BMI, body weight, low-density lipoprotein (LDL) cholesterol or quality of life.[32] However, results may have been affected by dietary adherence or glucose medication. There was evidence of diabetes medication reduction in the low-carbohydrate arms in some studies,[33–37] with evidence of medication reduction and improved blood glucose stability persisting for up to 2 years.[38] Hypoglycemia (though not after medication adjustment) was observed in three subjects taking insulin or a sulfonylurea out of twelve subjects in the low-carbohydrate arm of another study.[39] In a subset of participants with T2D (n=46) from a large weight loss trial, compared with those randomized to a low-fat diet and orlistat, those randomized to a low-carbohydrate diet for 48 weeks had improved HbA1c (0.8% lower, 95% CI, -1.5, -0.02%) and a reduction in antiglycemic medications, despite similar weight loss effects of the interventions.[40]

Though low-to-moderate carbohydrate diets have been studied among overweight and obese participants (primarily focused on weight loss), less is understood about the effects of such diets on glycemic outcomes specifically among individuals with prediabetes. A study among participants with IGT observed a reduction at 12 months in weight, HbA1c, and other glycemic outcomes among individuals who participated in a 7-day in-hospital education program, though this was not randomized.[41] A randomized pilot trial of overweight or obese individuals with T2D or prediabetes found that, over 3 months and at 12 months, a very low-carbohydrate diet improved HbA1c, led to higher medication discontinuation, and more weight loss compared with a low-fat diet.[34,37] However, only 4 individuals had prediabetes. Given benefits of low-to-moderate carbohydrate diets for weight loss in general populations and for glycemic control among patients with T2D, further study of these diets are warranted among individuals with prediabetes who are at elevated cardiometabolic disease risk.

2.E. Mediterranean-style diet is associated with reduced risk of T2D and improved glycemic control

Though there is no one "Mediterranean diet", the Mediterranean dietary pattern tends to have the following characteristics: high consumption of vegetables, fruits, breads and cereals, legumes, nuts and seeds; low-to-moderate consumption of dairy products, fish, poultry and eggs; little consumption of red meat and sweets; and red wine in moderation.[42] Olive oil is an important source of fat. In many observational studies, adherence to the Mediterranean-style dietary pattern is associated with reduced risk of CVD[43] and T2D.[44,45] Evidence from randomized trials also supports the benefits of this dietary pattern. In several trials, participants randomized to Mediterranean diets had substantially lower risk of CVD than those randomized to low-fat control diets.[46,47] In the three-arm randomized PREDIMED trial of participants at high risk of CVD, after 4.1 years of follow-up, those randomized to a traditional Mediterranean diet supplemented with either olive oil or nuts had a 30% reduced risk of T2D compared to those in the low-fat control diet group.[48] In all PREDIMED arms, average carbohydrate intake was relatively low (39.7–43.7% at end of trial).[47] Two trials have found a higher rate of remission from metabolic syndrome among individuals randomized to a Mediterranean diet compared with those randomized to control diets (prudent or low-fat) during 2–5 years of follow-up.[49,50] The Mediterranean pattern may also be beneficial among individuals with T2D. Meta-analyses have reported that, among patients with T2D, those randomized to a Mediterranean diet had lower HbA1c levels and CVD risk factors, and more significant weight loss, compared with those randomized to control diets.[51]

2.F. Evidence for low-carbohydrate diets that emphasize Mediterranean-style dietary components

Many low-carbohydrate diets involve increased consumption of food items, such as red meat,[52] that are associated with increased cardiometabolic disease risk. However, low-carbohydrate dietary approaches can focus on substituting carbohydrates with foods common in Mediterranean-style diets, such as olive oil and nuts, which may improve cardiometabolic health. In a 4-year Italian study of individuals with newly diagnosed T2D, those randomized to a low-carbohydrate Mediterranean diet (<50% carbohydrates; consumed ~44%) had more favorable changes in glycemic control, higher rates of T2D remission, and delayed need for antiglycemic drugs compared with participants randomized to a low-fat diet (<30% fat, <10% saturated fat).[53,54] A 12-month Israeli study assessed low-carbohydrate Mediterranean (35% carbohydrates), traditional Mediterranean, and ADA (both 50–55% carbohydrates) diets in 259 overweight participants with T2D.[55] Most CVD risk factors improved in all groups; there was only HDL improvement in the low-carbohydrate Mediterranean diet and this diet was superior to the other diets in improving glycemic control.[55] In a 2-year weight-loss study of moderately obese participants, low-carbohydrate and Mediterranean diets were superior to low-fat diet for weight loss.[56] Participants in the low-carbohydrate arm were counseled to choose vegetarian sources of fat and protein and to avoid trans fat. At 6 years after study commencement, despite weight regain, there were still long-term benefits, particularly for the low-carbohydrate and Mediterranean diets.[57] In whole, evidence supports that low-carbohydrate Mediterranean-style diets are feasible and may have metabolic benefits.

2.G. Evidence for specific food groups and risk of T2D

Prospective cohort studies have found consumption of specific foods and beverages is associated with diabetes risk. Meta-analyses report higher consumption of processed and unprocessed red meat,[58] white rice (particularly in Asian populations),[59] and sugar-sweetened beverages[60,61] are associated with higher T2D risk. Consumption of green leafy vegetables,[62,63] yogurt,[64] whole grains,[65] decaffeinated and total coffee,[66] and light-to-moderate alcohol

consumption[67] are associated with reduced risk. Consumption of fruits, particularly blueberries, grapes, and apples, are associated with reduced risk; fruit juice is associated with higher risk.[68]

2.H. Gut microbiota, diet and T2D

There is increasing evidence for the importance of gut microbiota in risk of type 2 diabetes and other cardiometabolic diseases.[75] Additionally, dietary modifications, including changes in carbohydrate consumption, can lead to substantial changes in gut microbiome over short periods of time.[76,77] Less is known about longer-term changes to the gut microbiota during the adoption of a low-carbohydrate diet and how these changes may influence cardiometabolic risk. We plan to collect stool specimens at baseline and at 6 months, for potential future studies on how this intervention affects the gut microbiome.

2.I. Clinical and public health significance of the proposed study

Prediabetes, or intermediate hyperglycemia, is a marker of high risk of developing T2D and other complications. Due to increasing prevalence of T2D and associated morbidity and mortality globally, a better understanding of dietary approaches to delay progression to T2D or to promote reversion to normoglycemia is a high public health priority. This study proposes to assess the effect of a behavioral intervention promoting a low-carbohydrate/high-unsaturated fat dietary pattern (initial target <40 g digestible carbohydrates, final target <60 g digestible carbohydrates) on HbA1c, a marker of glycemic control, and other metabolic risk factors. Building from prior findings from clinical and observational studies, we will develop a dietary intervention that promotes the consumption of non-starchy vegetables, plant-based oils, fish, poultry, and mixed nuts, that are thought to reduced risk of T2D or CVD. Participants assigned to the intervention will be trained in behavioral techniques that will provide capacity to maintain dietary changes long after study completion. Our outcome measures will assess the effect of this dietary intervention in a “real-life” setting as opposed to dietary modifications achieved in metabolic ward studies or through food prepared in research kitchens. Findings from this study may provide evidence in support of promoting alternative dietary styles among individuals with high T2D and CVD risk and could potentially be used to inform dietary interventions used in programs like DPP.

2.J. Innovation

The status quo as it pertains to dietary approaches for reducing risk of T2D is focused on reducing caloric intake and total fat intake. However, recent evidence suggests that the types of fat, rather than the quantity of fats may be more important, with evidence supporting that a Mediterranean-style diet that does not restrict fat intake reduces risk of developing both T2D[48] and CVD[47]. Moreover, in the short-term, low-carbohydrate diets, which are popular weight loss diets, have a greater glucose-lowering effect than do high-carbohydrate diets among patients with T2D.[32] However, the effect of low-carbohydrate diets that emphasize consumption of non-starchy vegetables, plant-based oils, fish, poultry, and mixed nuts on T2D prevention is unclear, particularly compared with higher-carbohydrate diets more commonly consumed in the US. *The proposed research is innovative, in our opinion, because it represents a substantial departure from the status quo by assessing the combined effect of a low-carbohydrate, high unsaturated fat and protein diet on cardiometabolic risk factors among adults with prediabetes or early-stage untreated diabetes.* We expect the results from this study have the potential to lead to new horizons for developing and implementing dietary approaches (other than the most commonly used reduced fat diet) that will substantially reduce risk of cardiometabolic disease among adults with T2D or at high risk of T2D. The proposed intervention dietary approach promotes consumption of dietary components thought to reduce risk of cardiometabolic disease and the behavior change intervention has expected applicability in clinical practice.

3. Preliminary Studies

3.A. Summary of aggregate experience of the research team

Dr. Kirsten Dorans is trained in cardiovascular disease epidemiology, with strong training in epidemiologic methods and biostatistics. Her publication record includes studies of behavioral risk factors and risk of chronic disease. She has published on the relationships between dietary intake and chronic heart failure[78,79] and on physical activity and incident multiple sclerosis[80]. She served as lead author, conducting data management, analysis, drafting, and revision of two of these manuscripts. In graduate school, she focused on studying links between outdoor air pollution and chronic diseases and has published several papers in this field. The COBRE program will provide an excellent opportunity for the JFI to transition from air pollution research to clinical and translational research focused on nutrition and diabetes. Building off of her training in observational epidemiology, this program will involve training and practical experience that will allow her to broaden her expertise and to develop practical skills for carrying out clinical research.

The mentorship team has much experience and prior successful collaborations in key areas related to this study. Dr. Jiang He is an internationally renowned expert in cardiometabolic disease clinical and epidemiological research. He has served as PI for many epidemiological studies and clinical trials, including clinical and observational studies focused on dietary consumption and chronic diseases[81–86] and has conducted extensive global research on diabetes.[87,88] Dr. Bazzano has collaborated extensively with Dr. He for more than 15 years in research related to diet and chronic disease. She is a clinical investigator and has expertise in nutritional epidemiology and clinical research. Dr. Bazzano was the PI of a highly successful clinical trial comparing the effects of low-carbohydrate and low-fat diets on weight and cardiovascular risk factors among individuals with obesity, which was a subproject within the Tulane COBRE in Hypertension and Renal Biology.[30] Dr. He served as Dr. Bazzano's mentor for this clinical trial. They have collaborated on much other work related to dietary and chronic disease, including a meta-analysis on low-carbohydrate diets.[28] Dr. Bazzano has also published many articles on diet and risk of diabetes[89,90] and on diabetes in the Bogalusa Heart Study.[91–93]

3.B. Low-carbohydrate behavioral dietary intervention trial experience

In the study on macronutrient composition of diet and risk factors for cardiovascular disease (MACRO) study, 148 individuals without clinical cardiovascular disease or diabetes and with obesity were randomly assigned to a low-carbohydrate or a low-fat behavioral dietary intervention for 12 months. In the low-carbohydrate arm, daily dietary intake of carbohydrates decreased from an average of 242 g at baseline to 97 g, 93 g, and 127 g at 3, 6, and 12 months, respectively. At 12 months, participants in the low-carbohydrate intervention group had greater decreases in weight (-3.5 kg; 95% CI, -5.6 to -1.4 kg) and fat mass (-1.5%; 95% CI, -2.6% to -0.4%) and greater improvement in several cardiovascular risk factors.[30] This suggests that restricting carbohydrates may be an option for weight loss and reduction of cardiovascular risk factors. Further analyses found that the low-carbohydrate dietary intervention led to similar or greater improvement in inflammation, adipocyte dysfunction, and endothelial dysfunction compared with the low-fat dietary intervention[94] and that the low-fat diet reduced peptide YY more than the low-carbohydrate diet, suggesting satiety may be better preserved on low-carbohydrate diets.[95] No serious adverse events were reported in this study and the number of participants with symptoms were similar between the two diets, with the exception of more participants having headaches on the low-fat diet at 3 months (18 vs 6 participants, $p = 0.03$).[30] Dr. Bazzano served as PI for this study and Dr. He served as her mentor. Dr. Bazzano

mentored one of her PhD students in conducting analyses and writing and editing manuscripts related to this project.

3.C. Other dietary intervention trial experience

Dr. He has also led or been involved with many dietary intervention trials, including both behavioral change and feeding study interventions. In the Protein and Blood Pressure (ProBP) study, 352 adults with prehypertension or stage 1 hypertension in New Orleans, LA and Jackson, MS were assigned to take, in a random order, 40 g/d soy protein, milk protein, or carbohydrate supplementation each for 8 weeks. Compared with the high-glycemic index refined carbohydrate, both soy protein and milk protein supplementation were associated with a -2.0 mm Hg (95% CI -3.2 to -0.7 mm Hg) and -2.3 mm Hg (-3.7 to -1.0 mm Hg) net change in in systolic blood pressure.[86] These findings suggest that partial replacement of carbohydrate with soy or milk protein might be an important part of nutrition intervention strategies to prevent and treat hypertension. Additionally, soy protein supplementation led to reduction in plasma E-selectin compared with carbohydrate and milk protein and in plasma leptin compared with carbohydrate.[96] Soy protein also led to improvement in lipid profile compared with carbohydrate and milk protein.[97] Dr. He was PI for this project.

In the Fiber Intervention on Blood Pressure (FIBRE) study, 110 participants ages 30–65 years with untreated pre-hypertension or stage-1 hypertension and serum cholesterol level < 240 mg/dl were randomly assigned to 8-g/day of water soluble fiber from oat bran or a control intervention (double-blind RCT). Over the 12-week study period, net changes in systolic and diastolic blood pressure were -1.8 mm Hg (-4.3 to 0.8) and -1.2 mm Hg (-3.0 to 0.5 mm Hg), respectively.[84] Net changes in total, HDL-, and LDL-cholesterol were -2.40 mg/dL (-10.60 to 5.81 mg/d:), -1.66 mg/dL (-4.55 to 1.22 mg/dL), and -1.33 mg/dl (-8.33 to 5.68 mg/dL), respectively.[98] Dr He was PI for this study.

Dr. He is currently serving as a mentor for Dr. Katherine Mills' COBRE project (Effect of Dietary Sodium Reduction in Kidney Disease Patients with Albuminuria; SUPER). In this project, participants with chronic kidney disease and albuminuria are being randomized to a behavioral modification program designed to reduce dietary sodium intake to ≤2,300 mg/day or to usual care and test the effect of this intervention on change in albumin-to-creatinine ratio from baseline to 24-weeks.

4. Study Design and Organization

4.A. Overview of trial design

Figure 1. Study Design

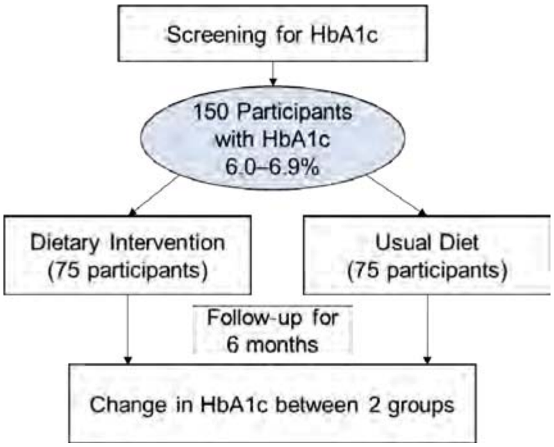

We propose to conduct a ***randomized controlled trial*** aimed at testing the effect of a behavioral intervention that promotes ***reduced carbohydrate intake and increased consumption of heart-healthy unsaturated fats and protein*** in patients with HbA1c 6.0–6.9% (***Figure 1***). We plan to recruit 150 individuals with elevated HbA1c and randomly assign them to a moderately intensive intervention on diet pattern,[99] using well-established methods for behavioral modification, or to usual diet. We will compare the difference between the active intervention and control groups for change in HbA1c from baseline to 6 months of follow-up (primary outcome). We will assess four major secondary outcomes: fasting plasma glucose, systolic BP, total-to-HDL cholesterol ratio, and body weight. In addition, we will look at several other exploratory outcomes: insulin and HOMA-IR, diastolic BP, waist circumference, and

estimated CVD risk, as estimated by the 2013 ACC/AHA risk equation. The main trial components are summarized in ***Table 1***.

Table 1. Study Overview

Study Design	Randomized controlled trial
Study Participants	150 participants with elevated HbA1c (6.0–6.9%)
Intervention Group (n=75)	Behavioral modification to reduce carbohydrate consumption and promote consumption of foods such as non-starchy vegetables, plant-based unsaturated oils, nuts and seeds, avocados, eggs, seafood, poultry, lean meat and tofu. We will recommend moderate consumption of cheese, unsweetened Greek yogurt, and low-carb milk. If consuming higher-carbohydrate items, recommend legumes, fruits, (limited) whole grains. Target <40 g net carbohydrates per day for first 3 months and will have a goal of <40 net g carbohydrates per day; <60 g net carbohydrates per day for months 4 onwards.
Usual Diet Group (n=75)	No dietary intervention. To encourage continued participation in the trial, we will offer optional monthly educational sessions on unrelated topics, such as retirement planning or health insurance
Outcome Measures	Primary: HbA1c; Secondary: fasting plasma glucose, systolic BP, total-to-HDL-cholesterol ratio, and body weight

4.B. Study outcomes

4.B.1. Primary outcome: The *primary outcome will be the difference between the intervention group and the usual diet group for change in HbA1c* from baseline to 6 months. Changes in HbA1c will be calculated from blood samples taken at baseline and 6 months.
4.B.2. Secondary outcomes: Secondary outcomes will include changes from baseline to 6 months of *fasting plasma glucose, systolic BP, total-to-HDL cholesterol ratio, and body weight.,*
4.B.3. Other outcomes: We will also study the effect of the invention on the following outcomes: insulin and homeostasis model assessment of insulin resistance (HOMA-IR); diastolic BP; waist circumference; total-to-HDL-cholesterol ratio; and estimated CVD risk.

4.C Study Timeline

Table 2 shows the timeline, which will be followed in the implementation of study-related activities, such as publications and submission of an R01 grant application.

Table 2. Study Timeline

	Year 1		Year 2		Year 3	
Develop MOP, intervention program, study forms, and pilot testing	X	X				
Certification and training of staff	X					
Recruitment		X	X			
Intervention and data collection, measurement, entry, cleaning		X	X	X	X	
Data quality control and analysis						X
Publications	X	X	X	X	X	X
R01 grant submissions, resubmissions				X	X	X

The first 6 months will focus on development of manual of operations (MOP), intervention program, study forms, study protocol, Institutional Review Board (IRB) approval, and pilot testing of the intervention program. It will also involve staff training and certification. During the

last half of year 1, recruitment, intervention, and data collection will commence. During the last 3 months of year 3, data quality control and analysis will be carried out. Throughout the study period, the JFI will publish peer-reviewed manuscripts from ongoing Tulane University research projects.

6. Study Participants

6.A. Eligibility criteria

Inclusion criteria

- Men or women ages 40 to 70 years: aim to recruit 50% women
- Any race or ethnicity: aim to recruit 40% ethnic minorities (i.e., African American and Hispanic), close to population in the greater New Orleans area[100]
- HbA1c 6.0–6.9%
- Willing and able to provide informed consent

Exclusion criteria

- Diagnosed type 1 diabetes mellitus based on patient self-report
- Use of agents affecting glycemic control (medications for diabetes, oral glucocorticoids) within the past three months prior to enrollment based on patient self-report
- Medical condition in which low-carbohydrate diet may not be advised
    - Estimated glomerular filtration rate (eGFR) ≤45 mL/min/1.73 m^2, which is close to the 5th percentile of eGFR among non-diseased individuals of 70 years of age[101]
    - Self-report of liver disease due to hepatitis or alcohol; osteoporosis; untreated thyroid disease; gout; or cancer (other than non-melanoma skin cancer) requiring treatment in the past year, unless prognosis is excellent
- Factors that may affect HbA1c:
    - Hemoglobin <11 mg/dL (cutpoint for moderate-to-severe anemia, which could lead to falsely elevated or lowered HbA1c)[102]
    - Recent blood donation or blood transfusion (self-report, past 4 months)
    - Human Immunodeficiency Virus (self-report)[103]
- Allergies to nuts
- For women, current pregnancy, breastfeeding, or plans to become pregnant during the study
- Consumption of ≥21 alcoholic drinks per week or consumption of ≥6 drinks per occasion
- Current or planned residence making it difficult to meet trial requirements (due to distance from study site and/or challenges regularly traveling to site)
- Current participation in another lifestyle intervention trial or a pharmaceutical trial
- Participation of another household member in the study; employees or persons living with employees of the study
- Other concerns regarding ability to meet trial requirements, at the discretion of the principal investigator or study coordinator

7. Recruitment and Randomization

7.A. Recruitment

We will recruit study participants from the greater New Orleans area. The initial focus of our recruitment will be mass mailing of individuals in the greater New Orleans area and recruitment through placing flyers and brochures in the community. If community-based recruitment is not successful or cost-effective, we will recruit out-patients from the Ochsner Health System and Tulane Medical Center (TMC), from which our group has prior experience recruiting patients.

For this recruitment, we will focus on electronic searches of medical records and laboratory databases. We will also carry out targeted solicitation of referrals from physicians who care for patients likely to meet our inclusion and exclusion criteria.

In addition to this recruitment plan, we have several contingency plans: (1) extending recruitment to other medical centers in the New Orleans area, including the Veterans Affairs Medical Center (VAMC), East Jefferson General Hospital, and West Jefferson Medical Center, with whom Tulane has well-established relationships; (2) TV/radio advertisements. We have ample experience with each of these approaches.

Based on U.S. Census Bureau 2016 population estimates, the metro New Orleans area population was approximately 35% African American or black and 9% Hispanic.[100] Given our prior experience and population distribution in this region, we plan to recruit a distribution of study participants with 40% ethnic minorities.

7.A.1. Mass mailing: Mass mailing individuals at high risk for prediabetes or T2D, i.e. individuals who are obese or overweight.

7.A.2. Database searches and targeted mailing: We will request IRB approval for a waiver for HIPAA authorization to conduct database searching and hospital chart reviews for identification of potentially eligible study participants. After identifying a potentially eligible patient, we will send this individual an invitation letter signed by the investigators and/or the individual's personal physician. Individuals who express interest in the study will have a pre-screening telephone interview for confirmation of potential eligibility. Those still interested and potentially eligible for enrollment will be scheduled for a visit at the COBRE study clinic.

7.A.3. Referrals: Potential participants referred by their physician will have a pre-screening telephone interview to confirm potential eligibility. Those who are still interested and potentially eligible for the study will be scheduled for a screening visit.

7.B. Randomization

Once eligibility has been confirmed, the study coordinator will ensure that all key variables have been entered into the trial-specific database. Additionally, the study coordinator will ensure any outliers have been recognized and reconciled, and will ensure receipt of informed consent prior to enrolling the volunteer in the study as a trial participant. Eligible participants will be *randomly assigned to the behavioral intervention or the usual diet group in a 1:1 allocation ratio*. Randomization will be stratified by sex, *using a random block group size (4, 6) to maintain balance among groups over time*. A statistician from the Methodology/Biostatistics Unit within the COBRE Clinical Research Core, who is not directly involved in the study and is not located at the clinic, will conduct the randomization. After ascertaining eligibility, clinical personnel will telephone the Methodology/Biostatistics Unit to obtain a randomization assignment. All randomization assignments will be created by a computer program and only accessible to the Staff at the Methodology/Biostatistics Unit. Once randomization assignment has been made, trial participants will be considered to be officially randomized into the study and every effort will be made to obtain complete follow-up information.

7.C. Masking

Clinical and laboratory staff members who assess outcomes will be blinded to the intervention assignment of participants. As this is a dietary intervention trial, study participants, and study staff members who conduct the intervention cannot be blinded to the intervention assignment. Participants will be instructed to avoid sharing knowledge of their treatment assignment with data collection staff members. We will have intervention visits and data collection study visits on different days and in separate locations. Data collectors will also use

verbal cues and visual props to remind participants not to disclose treatment assignment. Investigators will be blinded to intervention assignment.

7.D. Safety Considerations

Participants with HbA1c >7.9% will be referred to a clinician for follow-up[104], though we will continue to collect data on these participants.

8. Dietary Intervention Program

8.A. Low-carbohydrate dietary intervention group

Participants randomized to the low-carbohydrate intervention group will receive counseling aimed at reducing carbohydrate consumption. Phase I (*Go Low*) will occur for the first 3 months, with a goal of <40 g digestible carbohydrates per day. Key recommended foods will include: non-starchy vegetables, fish, poultry, lean meat, eggs, olive oil and other plant-based unsaturated oils, unsweetened/unsalted nuts and seeds, nut butters, and avocados. We will recommend moderate consumption of cheese and unsweetened Greek yogurt and low-carbohydrate milk. During Phase I, we will recommend limiting or avoiding other dairy, fruits, legumes, beans, and grains. Phase II (*Keep it Low*) will run from months 4 through 6. We will recommend that participants add 5 grams of carbohydrates per day and maintain this carbohydrate intake level for a week or two at a time, for a maximum total of <60 g net carbohydrates. As they add back carbohydrates, we will have them monitor their satisfaction with their diet and energy levels. We will recommend participants add back in foods like fruit (preferably berries), legumes, beans, dairy, and (limited) whole grains, while still aiming to keep total carbohydrate intake to <60 net grams.

Materials: We will develop a handbook that will be given to participants. This will contain diet guidelines, recipes, meal planners, shopping lists, and guides for counting carbohydrates and reading nutrition labels.

Meals: Participants will select their own menus and prepare or buy their meals according to the guidelines. We will provide participants with supplemental low-carbohydrate foods, such as nuts, olive oil, and other products, to help facilitate their compliance.

Counseling: Sessions and materials will focus on instructing participants to reduce digestible carbohydrate consumption by increasing consumption of protein and fat, with a particular emphasis on increased consumption of heart-healthy unsaturated fats. After randomization, participants will begin the 3-month *Go Low* phase consisting of individual counseling sessions each week for the first four weeks, followed by 4 group sessions held every other week with phone follow-ups occurring in between group sessions. During the *Keep it Low* phase, months 4 through 6, participants will attend 3 monthly group sessions and have 3 telephone follow-ups. There will be a total of 11 in-person sessions and 7 telephone follow-ups over the full study period. Sessions will be approximately one hour and phone calls will be no longer than thirty minutes. These sessions will consist of diet instruction and supportive counseling, addressing results from 24-hour dietary recalls, teaching self-monitoring and individual diet goal changes, and provide suggestions for replacing usual foods containing carbohydrates. Study materials will be modified from materials used in the successful MACRO study. The JFI will work closely with Drs. He and Bazzano to develop the intervention based on the MACRO study.

Carbohydrate goal: In the MACRO study, carried out among individuals from the same study region, a behavioral intervention aimed at reducing digestible carbohydrate intake to <40 g/day led to reductions in weight and improvements in cardiovascular risk factors among individuals with obesity.[30] Additionally, restriction of carbohydrate intake to <50g/day is sufficient to induce

ketosis.[105] Our rationale for having a multi-phased intervention is to allow participants to see how they feel on the more restrictive diet, and see how they respond to small increases in carbohydrate consumption. A similar technique was applied in the DIETFITS published study.[106] The higher target of 60 net grams may be more sustainable long-term and also allows for consumption of healthy higher-carbohydrate foods, such as berries, legumes, beans, and limited whole grains.

8.B. Usual Diet Group

We will provide participants randomized to the usual diet group will receive written information with standard dietary advice. Participants randomized to the usual diet group will not receive ongoing dietary recommendations. Moreover, they will continue to receive clinical care recommendations from their physician(s). To encourage continued participation in the study and minimize the difference in participant contact with study staff, participants will be offered optional monthly educational sessions on topics unrelated to the study intervention, such as retirement planning or health insurance options.

In another effort to encourage continued participation, patients randomized to the usual diet group will be offered a copy of all available intervention materials, as well as an optional one-hour counseling session with the interventionist following their final study visit.

8.C. Physical Activity

At baseline, both groups will receive written information in study materials with standard physical activity recommendations.

8.D. Dietary Adherence

Strategies for measuring compliance: For the low-carbohydrate diet, adherence will be assessed through measurement of urinary ketones at 3-month visit and 6-month TV. Study staff will make all reasonable effort to maintain adherence of participants to the visit schedule and diet intervention.

Strategies for improving compliance:

- At the screening visit, we will ask participants who does most of the cooking in their households and how often they eat outside of the home. Results from the screening visit will help to select a group of cooperative study participants.
- Visits will be scheduled at the participants' convenience and to minimize waiting time and trips to the clinic; reminder calls will also be made prior to clinic visits.
- We will hire enthusiastic staff who have good interpersonal skills.
- Participants will receive $20 gift cards for use at food stores or retailers when they complete each study visit, for a maximum of $60 over the study period.
- Participants will receive $25 gift cards when they return the stool specimens, for a maximum of $50 over the study period.
- Participants will be provided with recipes, shopping lists, and food samples for the intervention diet.

9. Safety of Dietary Intervention

On the low-carbohydrate diet, participants will be encouraged to primarily focus on replacing carbohydrates (particularly highly refined carbohydrates) with foods such as non-starchy vegetables, olive oil and other plant-based unsaturated oils, nuts and seeds, avocados, eggs, seafood, tofu, poultry, and lean meat. Consumption of more unsaturated fats, protein, and fiber will be recommended. Participants will not be encouraged to increase consumption of saturated fats and will be advised to avoid consumption of trans fats.

In animal studies, a high-protein diet has decreased renal function[107] In the MACRO study, on average, participants tended to increase consumption of fats, rather than protein.[30] The safety and tolerability of low-carbohydrate diets have previously been assessed in many studies, including MACRO.[30] A number of studies, including some in individuals with T2D, have found no clinically significant increases in estimated glomerular filtration rates,[33,38–40] microalbuminuria,[108] albumin excretion rate,[33,38] urinary albumin,[38] serum creatinine,[38,40,108–113] albumin,[40] or uric acid levels.[109–112] A small study of a very low-carbohydrate diet found significant increase in uric acid excretion, but no significant change in 24-hour urine creatinine clearance.[112]

Study staff will carefully monitor participants' BP, and HbA1c more often than what would occur in routine healthcare visits. A Data Safety and Monitoring Board (DSMB) consisting of an independent group of experts in areas such as biostatistics, nutrition medicine, general internal medicine, and endocrinology has been formed. The board will regularly review trial progress, including unblinded interim results, and can recommend that the trial prematurely end if participants are at risk or if it becomes clear that the trial will not be able to yield statistically significant results.

10. Data Collection

10.A. Study visits

After the initial pre-screening interview, in which data on medications and medical conditions will be collected, there will be one screening visit, one randomization visit, a follow-up study visit at 3 months and a termination visit (TV) at 6 months. ***Table 3*** summarizes study procedures and data collected at each study visit.

Table 3. Study visit schedule and data collection

	Pre-screen	SV	RZ	3 month	6 month TV
Pre-screen eligibility	X				
Informed consent		X			
Compliance form		X			
Eligibility confirmation			X		
Randomization			X		
Demographics		X			
Medical history questionnaire		X	X	X	X
Medications		X	X	X	X
Lifestyle (alcohol, cigarette smoking)		X	X	X	X
Physical activity			X	X	X
24-hour diet recall*			X	X	X
3 BP measurements		X	X	X	X
Body weight			X	X	X
Height			X		
Waist circumference			X	X	X
Dipstick ketone test			X	X	X
Blood samples		X	X	X	X

Stool specimen	X		X
Side-effects assessment	X	X	X
*Two 24-hour dietary recalls will be collected; one will cover a weekend and the other a weekday.			

10.A.1. Screening/baseline visits:

Participants will be pre-screened by phone or in-person by study staff to confirm eligibility for the Screening Visit. An approved questionnaire will be used to ensure the patient is eligible to attend the in-clinic screening visit, and the patient will be scheduled thereafter.

At the screening visit (SV), participants will provide informed consent for the screening and main study. Study staff will schedule the SV and randomization (RZ) visit at least 2 days apart. If a participant is eligible based on the screening visit, he or she should be scheduled for the RZ once a sufficient group of eligible individuals has been recruited. Ideally, participants should be scheduled for the RZ within two to three weeks of the screening visit.

At the screening visit, we will collect information on inclusion/exclusion criteria, medication use, demographics, medical history, and lifestyle risk factors. At this visit, we will also have participants complete a compliance form, to assess their ability to complete the study and attend all intervention and study visits. We will also measure blood pressure, and take blood samples. Women will be asked if they are postmenopausal or if they had surgical sterilization (such as a tubal ligation). If not, they will be tested for pregnancy through a spot urine test; pregnant women will be excluded from the study. Blood samples will be collected for measurement of HbA1c, and, if not available from medical records in the past 3 months, serum creatinine and hemoglobin. These blood samples will be used for excluding individuals with HbA1c <6.0 or >6.9, eGFR ≤45 mL/min/1.73 m², or hemoglobin <11 mg/dL.

Before the start of the intervention, participants will complete two 24-hour dietary recalls. At the RZ, participants will learn about the randomization process. After requirements for eligibility have been satisfied, participants will formally be enrolled into the study and randomized to either the active intervention or control group. We will collect fasting blood samples for later measurement of glucose, insulin and lipids and other potential future measurements. At this visit, we will measure blood pressure, body weight, height, and waist circumference. We will collect information on medical history, lifestyle risk factors, and physical activity. A spot urine sample will be collected to measure ketones and participants will be asked to collect a stool specimen for potential use in future microbiome studies.

10.A.2. Follow-up visits and final visit: A follow-up visit will occur at 3 months after randomization. A final visit will occur at 6-months post-randomization. These visits will aim to be scheduled as close as possible to the 3-, or 6-months after randomization, with a goal of being within two weeks of these follow-up periods. At each visit, study staff will conduct dietary recalls and anthropometric measures, take 3 BP measurements, collect a fasting blood sample to measure HbA1c. We will also collect samples for later measurement of glucose, insulin, lipid profile, and other potential future measurements. A spot urine sample will be collected to measure ketones. At the 6-month TV, participants will be asked to collect a stool specimen for potential use in future microbiome studies. At study termination, we will mail participants and/or their physicians a summary of trial results and a record of participant BP, anthropometric measures, and laboratory data from the trial period. Throughout the study period, we will provide participants with their blood pressure and weight.

10.B Study measurements

Measurements obtained for this study will be based on those used in other clinical trials and epidemiologic studies. We are familiar with strengths and limitations of such measurements and the importance of staff training and certification and use of stringent quality control procedures.

10.B.1. Questionnaires: Study staff will administer a *medical history questionnaire* at the SV, RZ, 3-month visit and 6-month TV to assess medical conditions that would necessitate changes in therapy or medical management beyond this study's scope. At the SV, *demographic factors* will be collected. At the SV, RZ, 3-month and 6-month TV, we will also assess *health behaviors*, including smoking, alcohol use, and administer *medication tracking questionnaires*. At RZ, 3-month, and 6-month TV, we will collect physical activity data.

10.B.2. Dietary measurements: Two 24-hour dietary recalls will be obtained from participants at baseline. At or close to both the 3-month and 6-month TV, two more 24-hour dietary recalls will be collected. The second sets of two recalls will be collected as closely as possible to 3- or 6-month dates after randomization, with a goal of being within 2 weeks of the respective follow-up period. For each time point, one recall must reflect week day consumption, while the other must reflect weekend day consumption and the two recalls should be at least 2 days apart from each other. We will use computer software (Nutrition Data System for Research [NDS-R]) and food models used in previous and ongoing studies. Recalls will be conducted by a study staff member who has been certified in the use of NDS-R. Food composition tables of the NDS-R will be used to calculate dietary nutrient intakes. The baseline dietary assessment will be done to characterize individual dietary intake of carbohydrates and other macronutrients. During the intervention, repeated measurements will be used to monitor short-term changes in mean dietary nutrient intake between the intervention and usual diet groups.

10.B.3. Assessment and management of adverse effects: At RZ, 3-month visit, and 6-month TV, we will administer *symptom questionnaires*, which will consist of a checklist to identify participant complaints. Prior studies of low-carbohydrate dietary interventions suggest that participants may experience a range of adverse effects, including constipation, halitosis, fatigue, headache, thirst, polyuria, and nausea. Severe adverse events will be promptly reported to the DSMB. Management of adverse effects will be based on the protection and safety of the participant. If severe adverse effects are reported by a participant, carbohydrate intake will be examined and altered in order to mitigate the adverse effect. In any extreme cases, participants will be instructed to stop the intervention. As this is a behavioral intervention, adverse effects should be rare.

10.B.4. BP and anthropometric measures: BP will be measured 3 times at the screening visit and at each follow-up visit. To minimize random error, averages of the measurements at the screening and randomization visits will be taken. BP will be measured by a standard automated blood pressure measurement device (OMRON HEM-907 XL Professional Digital Blood Pressure Monitor) and performed by experienced staff, trained and certified in BP measurement. Prior to BP measurements, participants will be asked to rest quietly in a seated position with their feet supported for at least 5 minutes; staff members will use an appropriate cuff size over a bare arm. Trained and certified staff will measure weight using a calibrated balance beam scale at the screening visits, and follow-up visits. Weight and height will be measured at the RZ, and weight will also be measured at the subsequent visits.

10.B.5. Blood samples: For SV, participants will not need to fast. For RZ, 3-month visit, and 6-month TV, participants will be instructed to fast for 12 hours prior to blood sample collection. At SV, blood samples will be collected to measure HbA1c, hemoglobin, and serum creatinine. At RZ, fasting blood samples will be collected and stored as serum/plasma, whole blood aliquots and buffy coats at -80°C for future analyses, including measurement of glucose, insulin and lipids, and potential future metabolomics or genetic analyses. At 3-month visit and 6-month TV, we will collect fasting blood samples to measure HbA1c and will also store additional

serum/plasma, whole blood aliquots and buffy coats at -80°C for future analyses, including measurement of glucose, insulin and lipids, and potential future metabolomics or genetic analyses. Details of the blood sample collection are shown in ***Table 4.*** HOMA-IR will be calculated from fasting glucose and insulin.

Table 4. Study visit schedule for specimen collection

	SV	RV	3 month	6 month TV
HbA1c	X		X	X
Serum creatinine	X			
Hemoglobin	X			
Storage of aliquots and buffy coats (for measurement of glucose, insulin, lipids, other potential future measurements)		X	X	X
Urine sample for pregnancy	X			
Urine sample for ketones		X	X	X
Stool sample		X		X

10.B.6. Urine collection: Study staff will collect spot urine specimens at RZ, interim visit, and the final visit to measure ketones.

10.B.7. Stool Specimen Collection: Stool samples will be collected from all eligible and willing trial participants during the RZ and 6-month TV, following the protocol set forth by the Human Microbiome Project.[114,115] Participants will be given a stool collection kit, which includes the specimen collection container, a shipper for home collection, and a form to record the timing and pertinent information related to specimen collection. Participants will be instructed to return the kit to the clinic within two days of collection (in-person or via shipping), at which time stool samples are stored at -80°C until analyses. Kits and instructions for collection will be reviewed by study staff with the participant at the required visits.

11. Quality Assurance and Quality Control

Throughout the study, we will implement rigorous quality assurance (QA) and quality control (QC) measures. We describe some of these approaches here.

11.A. Manual of procedures

We will develop a manual of procedures (MOP) based on the MOP successfully used in the MACRO behavior change clinical trial. The MOP will provide instructions and describe procedures for staff training and certification, participant recruitment, intervention counseling, study form completion and procedures, data entry, safety measures (e.g., methods for emergency participant/primary care physician contact).

11.B. Training and certification

Trial personnel will all be required to participate in a training session before the start of any trial procedures or measurements. Training will include particular attention to core trial

elements, such as recruitment, retention, intervention, completeness and quality of data acquisition (e.g., blinding procedures for the data collectors), and data entry. Separately, intervention staff will meet and receive detailed instruction on trial approaches to group and individual behavior change counseling and procedures that ensure data collectors are blinded to treatment assignment. Throughout the trial, periodic re-training/certification sessions will be conducted to ensure all trial-related procedures are being applied in a standard manner. Investigators will also monitor all aspects of study performance to ensure study goal achievement, timeliness and completeness of study visits, completeness and quality of data collection, intervention adherence, and adequacy of the procedures separating data collection from intervention. Study staff and investigators will pay particularly careful attention to the quality of all laboratory measurements, particularly key exposure, outcome, and intervention monitoring variables.

12. Sample Size and Statistical Power

The primary outcome will be the difference in the change of HbA1c between the intervention and usual diet groups from baseline to 6 months of follow-up. Based on estimated SD for change in HbA1c from a medical nutrition therapy intervention among individuals with IFG or HbA1c 5.7–6.4%[116] and of weight loss programs among individuals with HbA1c 5.7–6.4% or prior gestational diabetes,[117] assuming an SD of 0.31 for change in HbA1c, we would need 120 participants to have 85% power to detect an approximate difference in change of HbA1c of 0.17% between the intervention and usual diet groups.

In a follow-up analysis of the DPP, a 0.17% decrement in HbA1c at 6 months was associated with an 18% lower risk of incident T2D, adjusting for treatment arm, age, sex, race/ethnicity, household income, and baseline HbA1c.[71] In a pooled analysis from the Atherosclerosis Risk in Communities and Framingham Heart Studies, a 0.17% higher HbA1c was associated with an 18% higher risk of T2D over 20 years, a 35% higher risk over 8 years, and a 15% higher risk after 8 years, after adjusting for age, sex, fasting glucose, triglycerides, HDL, family history of T2D, SBP, and BMI.[69] Additionally, in EPIC-Norfork, a difference of HbA1c of 0.17% was associated with approximately a 6% higher risk of CHD among those free of CHD, stroke, diabetes, and with HbA1c <7.0%.[72]

Table 5. Minimum detectable differences

	SD	Detectable Difference
HbA1c, %	0.31	0.17
Fasting glucose, mg/dL	12.5	6.9
Fasting insulin, mU/mL	7.0	3.9
HOMA-IR	2.0	1.1
SBP (mmHg)	4.2	2.3
DBP (mmHg)	3.0	1.7
Total/HDL ratio	1.2	0.66
Body weight (kg)	6.6	3.7
Waist circumference (cm)	8.0	4.4
Estimated CVD risk (%)*	2.7	1.6

*10-y CVD risk, among estimated 85% or 102 participants free of CVD

In the DPP study, HbA1c was 0.18% lower among those in the lifestyle intervention arm than in the control arm at 1-year of follow-up; HbA1c at study commencement was 5.9% and levels increased in the control arm and decreased in the behavioral intervention arm.[22] In a small behavioral intervention study (n=34) of a very low-carbohydrate ketogenic diet compared with a moderate carbohydrate, calorie-restricted, low-fat diet among individuals with type 2 diabetes or prediabetes (HbA1c >6.0%) and elevated body weight (BMI >25), mean HbA1c decreased from 6.6% to 6.1% in the very low-carbohydrate group and from 6.9% to 6.7% in the moderate carbohydrate group over a 12-month period; additionally, the very low-carbohydrate group had greater reductions in glucose-lowering medications.[37]

Detectable differences are listed for HbA1c and secondary outcomes in ***Table 5***, based on data from prior studies.[30,53,56,118] These were calculated for unpaired t-tests, power of 85%, two-sided alpha of 0.05, and assuming 80% follow-up of 150 participants, which would yield an estimated 120 participants.

13. Data Management and Analysis

13.A. Data management

Personnel will double-enter all study data. Prior to entering data, the study coordinator will conduct quality control on all study forms, study measures, and laboratory data. Data will be cleaned and logically checked.

13.B. Data analysis plan

The data will be analyzed using *intention to treat*; this involves comparing the primary and secondary outcomes between participants based on randomization assignment.

13.B.1. Exploratory data analysis: We will express group data for variables with a normal distribution as mean ± standard deviation and for variables not normally distributed as median and interquartile ranges (or will log-transform the variables to obtain a normal distribution). We will compare means and percentages of baseline variables across the intervention and usual diet group to assess similarity of these groups at randomization.

13.B.2. Primary, secondary, and exploratory outcomes: We will test the primary study hypothesis that there is a greater reduction in HbA1c from baseline to 6 months in the intervention group than in the usual diet group. To do this, we will use a mixed-effects linear regression model, to test the null hypothesis that there is no difference in change between baseline and 6-month HbA1c for the intervention group compared with the usual diet group (Specific Aim 1). To do this, we will use the following model:

$$E(Y_{i,j}|T) = b_0 + b_1G_i + b_2P_j + b_3T + b_4G_iT$$

where $E(Y_{i,j}|T)$ is expected HbA1c for the jth participant (P) nested in the ith intervention group (G) at time T (where T=0 for baseline and 3 months and T=1 for 6 months). Rejection of the null hypothesis (b_1=0 and b_4=0) indicates there is a significant difference in change of HbA1c from baseline to 6 months between the intervention and control groups. In this model, participants are assumed to be random effects and intervention group, time, and the interaction terms are fixed effects.

We will use a similar approach to test the difference in the change between baseline and 6-month values of secondary outcomes (Specific Aim 2) and exploratory outcomes. We will also explore the use of mixed effects models to assess overall net change in HbA1c (by treating time as a linear variable) and other outcomes over the full study period. For each outcome, we will test modeling assumptions and will consider transformation of outcomes, if needed, to meet modeling assumptions. As the mixed-effects model assumes missing at random, we will also perform sensitivity analyses using Markov-chain Monte Carlo techniques to impute missing values of the outcomes, using covariates for this imputation and will compare results from this analysis with our primary findings.

15. Anticipated Challenges

Recruitment: Many clinical trials have challenges recruiting sufficient numbers of volunteers who meet the trial inclusion and exclusion criteria and demonstrate a high level of adherence to trial protocol requirements. Fortunately, the investigators are very experienced with clinical trial recruitment, and have several contingency options that can be used to meet unanticipated shortfall in recruitment.

Intervention: The investigators are aware of and understand challenges related to achieving and maintaining compliance with a behavioral intervention. The investigators have much experience and success in behavioral interventions, with recent success of a behavioral intervention aimed at reducing carbohydrate intake among participants from the New Orleans area. The JFI will collaborate with Drs. He and Bazzano to develop the intervention program based on the MACRO study and will assess potential barriers to implementation and intervention adherence (such as financial concerns and range of dietary patterns of study participants) during the planning and pilot phase of the study.

Adherence: Though adherence and follow-up are also main challenges related to clinical trials, the investigators have an outstanding track record for studies that have recruited participants for both clinical and epidemiology studies in the Greater New Orleans area.

16. Protection of Human Subjects

This Human Subjects Research meets the definition of a clinical trial. All investigators will be required to complete the CITI (Collaborative Institutional Training Initiative) program in the ethical use of human participants in research.

16.A. Protection of human subjects from research risks

16.A.1. Risks to the subjects

Human subject involvement and characteristics: We expect to enroll 150 participants, with an equal male to female ratio. We expect to enroll approximately 30 participants who are African-Americans, and another 10 with mixed racial backgrounds, and a few Asian participants given the demographics of the greater New Orleans area. Overall, given the demographics of greater New Orleans, we estimate approximately 10% participants of Hispanic or Latino ethnicity, 50% of them with African-American racial background.

Inclusion criteria

- Men or women ages 40 to 70 years: aim to recruit 50% women
- Any race or ethnicity: aim to recruit 40% ethnic minorities (i.e., African American and Hispanic), close to population in the greater New Orleans area[100]
- HbA1c 6.0–6.9%
- Willing and able to provide informed consent

Exclusion criteria

- Diagnosed type 1 diabetes mellitus based on patient self-report
- Use of agents affecting glycemic control (medications for diabetes, oral glucocorticoids) within the past three months prior to enrollment based on patient self-report
- Medical condition in which low-carbohydrate diet may not be advised
    - Estimated glomerular filtration rate (eGFR) ≤45 mL/min/1.73 m², which is close to the 5th percentile of eGFR among non-diseased individuals of 70 years of age[101]
    - Self-report of liver disease due to hepatitis or alcohol; osteoporosis; untreated thyroid disease; gout; or cancer (other than non-melanoma skin cancer) requiring treatment in the past year, unless prognosis is excellent
- Factors that may affect HbA1c:
    - Hemoglobin <11 mg/dL (cutpoint for moderate-to-severe anemia, which could lead to falsely elevated or lowered HbA1c)[102]

  - Recent blood donation or blood transfusion (self-report, past 4 months)
  - Human Immunodeficiency Virus (self-report)[103]
- Allergies to nuts
- For women, current pregnancy, breastfeeding, or plans to become pregnant during the study
- Consumption of ≥21 alcoholic drinks per week or consumption of ≥6 drinks per occasion
- Current or planned residence making it difficult to meet trial requirements (due to distance from study site and/or challenges regularly traveling to site)
- Current participation in another lifestyle intervention trial or a pharmaceutical trial
- Participation of another household member in the study; employees or persons living with employees of the study
- Other concerns regarding ability to meet trial requirements, at the discretion of the principal investigator or study coordinator

Sources of materials: Medical information data will be collected from the participant and, after written authorization for the disclosure of protected health information, from participants' medical records. Blood and urine samples will be collected to measure HbA1c, glucose, insulin, lipids, creatinine, ketones, and test for pregnancy. Blood pressure, height, weight, waist circumference and other physical measurements will be obtained.

Potential Risks: Risks associated with venipuncture, the diet intervention, and collection of confidential medical information are the main risks associated with this study. There is a small risk of bruising and a rare chance of local infection associated with standard venipuncture to collect blood samples. The dietary intervention has minimal risks. Reduced carbohydrate consumption may lead to adverse effects, including constipation, halitosis, fatigue, headache, thirst, polyuria, and nausea. At RZ, 3- , and 6-month visits, we will administer *symptom questionnaires*, which will consist of a checklist to identify participant complaints. Adverse effects of the low-carbohydrate diet will be assessed at counseling sessions. Severe adverse events will be promptly reported to the DSMB. Management of adverse effects will be based on the protection and safety of the participant. If severe adverse effects are reported by a participant, carbohydrate intake will be examined and altered in order to mitigate the adverse effect. In any extreme cases, participants will be instructed to stop the intervention. Furthermore, there is a potential risk of unauthorized disclosure of medical information. This risk will be minimized by using a strict protocol for data processing and by the appropriate training of all study personnel. Protection against risks is described in more detail below.

16.A.2. Adequacy of protection against risks

Recruitment and Informed Consent: The proposed study will be conducted following strict guidelines for the protection of the rights of human volunteers. The informed consent document will be signed by all participants at the screening/baseline visit. The informed consent form will be developed in the first year of the study and approved by the IRB. The consent form will clearly state the purpose, eligibility criteria, and protocol of the study, the potential benefits and risks of participation, and the subject's right to refuse to participate or withdraw. Prior to signing the informed consent, research staff will review the details of the consent form orally with the participant, and answer any questions that the participant has concerning participation in the study. In the case of potential participants with low or no literacy, the informed consent form will be read to the participant in its entirety and any questions will be answered prior to the

participant signing or marking the form. Specifically, the following must be accomplished during the informed consent process:

- The participant must be informed that participation in the study is **voluntary** and that refusal to participate will involve no penalty or loss of benefits.
- The participant must be informed of the **purpose** of the study and that it involves **research**.
- The participant must be informed of any **alternative procedures,** if applicable.
- The participant must be informed of any reasonably foreseeable **risks**.
- The participant must be informed of any **benefits** from the research.
- An outline of safeguards to protect participant **confidentiality** will be included, as well as an indication of the participant's right to withdraw without penalty. This should be balanced with a discussion of the effect that withdrawals will have on the study, and the responsibility a participant has, within limits, to continue in the study if he or she decides to enroll.
- The participant must be informed of his or her right to have **questions answered** at any time and **whom to contact** for answers or in the event of research-related injury.
- The participant must be informed as to whether or not **compensation** is offered for participation in the study and/or in the event of a medical injury.
- The participant must be informed that he/she will be notified of any significant **changes** in the protocol that might affect their willingness to continue in the study.

Written authorization for the disclosure of protected health information will be obtained during the consent process on a separate form (HIPPA authorization), if applicable.

Protection against Risk: The protocol and informed consent will be approved by the Institutional Review Boards at Tulane University Health Sciences Center and any other participating institution for recruitment. The study participants will be given contact information for the Study Coordinator and Study PI to answer questions or to report complaints. Patients can withdraw from the study at any time without any effect on their medical care. Standard techniques will be used by trained, certified phlebotomists or study nurses for blood sample collection at the study clinics where emergency medical equipment is available. Confidentiality of all data collected during the study will be maintained through the use of unique, encrypted study identification (ID numbers, rather than patient names, in the study database). These encrypted ID numbers will not be mathematical derivation of the subject's medical record number or any other patient identifier. No information will be disclosed in an individually identifiable form in any type of presentation or publication. There will be password-required access to the computerized file linking the study numbers to patient identifiers at the study clinics. Password-access will be restricted to pertinent research staff only. All study data will be kept in locked file cabinets in locked rooms, accessible only by study staff with permission. All data transmissions will use HIPPA-compliant password protected system and will be secured through a virtual private network.

16.A.3. Potential benefits of the proposed research to human subjects and others

Study participants may have improved glycemic outcomes, blood pressure, lipid levels, may lose weight and reduce waist circumference, and may have improvement in other risk factors for CVD and development and progression of T2D. Participants will receive multiple blood pressure, weight, waist circumference, and blood tests. If vital signs or lab values are clinically abnormal, participants will be referred to their primary care physician, or if they do not have a primary care physician, one will be assigned through the Tulane University Community Health Center, which is a part of the core clinical facilities. At the end of the study, we will provide the

participants' primary care physician with a copy of all the blood pressure, weight, and laboratory results.

16.A.4. Importance of knowledge to be gained

Information from this study may contribute to reducing the onset of T2D and the burden of T2D and cardiovascular disease in Louisiana and the general US population. The study has potential for public health impact; results from this study may provide evidence in support of promoting alternative dietary styles (than the most commonly used low-fat diet) among individuals with high T2D risk or with T2D.

16.A.5. Remuneration

For screening visit 2, the two follow-up visits, and the final visits, participants will receive $20 gift cards for use at food stores or retailers (after completing each study visit), for a maximum of $60 over the study period. Participants who return a stool sample will receive $25 gift cards for each stool sample that they return, for a total of $50 for two stool samples. Participants will also receive free parking for each intervention and study visit.

16.B. Data safety and monitoring plan

A DSMB, which consists of 3 experts who are not otherwise affiliated with the COBRE program, has been formed. Study investigators for this study will prepare accurate and timely data tables and reports for the DSMB meetings. The members of the DSMB will serve in an individual capacity and provide their expertise and recommendations. The primary responsibilities of the DSMB are: 1) periodically review and evaluate the accumulated study data for participant safety, study conduct and progress, and, when appropriate, efficacy, and 2) make recommendations to the NIH concerning the continuation, modification, or termination of the trial. The DSMB considers study-specific data, as well as relevant background knowledge, about the disease, test agent, and patient population under study.

The DSMB is responsible for defining its deliberative processes, including event triggers that would call for an unscheduled review, stopping guidelines, unmasking (unblinding) and voting procedures prior to initiating any data review. The DSMB is also responsible for maintaining the confidentiality of its internal discussions and activities, as well as the contents of reports provided to it.

The DSMB will review each protocol for any major concerns prior to implementation. During the trial, the DSMB should review cumulative study data to evaluate safety, study conduct, and scientific validity and integrity of the trial. As part of this responsibility, DSMB members must be satisfied that the timeliness, completeness, and accuracy of the data submitted to them for review are sufficient for evaluation of the safety and welfare of study participants. The DSMB should also assess the performance of overall study operations and any other relevant issues, as necessary.

Items reviewed by the DSMB include:

- Interim/cumulative data for evidence of study-related adverse events;
- Interim/cumulative data for evidence of efficacy according to pre-established statistical guidelines, if appropriate;
- Data quality, completeness, and timeliness;
- Adequacy of compliance with goals for recruitment and retention, including those related to the participation of women and minorities;
- Adherence to the protocol;

- Factors that might affect the study outcome or compromise the confidentiality of the trial data (such as protocol violations, unmasking, etc.); and,
- Factors external to the study, such as scientific or therapeutic developments, that may impact participant safety or the ethics of the study.

The DSMB will conclude each review with their recommendations to the COBRE program and NIH/NIGMS as to whether the study should continue without change or be modified or terminated. Recommendations regarding modification of the design and conduct of the study could include:

- Modifications of the study protocol based upon review of the safety data;
- Suspension or early termination of the study or of one or more study arms because of serious concerns about subjects' safety, inadequate performance or rate of enrollment;
- Suspension or early termination of the study or of one or more study arms because study objectives have not been obtained according to pre-established statistical guidelines; and,
- Corrective actions regarding a study when the performance appears unsatisfactory or suspicious.

Confidentiality must always be maintained during all phases of DSMB review and deliberation. Usually, only voting members of the DSMB should have access to interim analyses of outcome data by treatment group. Exceptions may be made when the DSMB deems it appropriate. The reason and to whom the exceptions for access to interim analyses is granted will be documented in the Closed Session Report. DSMB members must maintain strict confidentiality concerning all privileged trial results ever provided to them. The DSMB should review data only by masked study group (such as X vs. Y rather than experimental vs. control) unless or until the DSMB determines that the identities of the groups are necessary for their decision-making. Whenever masked data are presented to the DSMB, the key to the group coding must be available for immediate unmasking.

The DSMB will meet twice per year. The junior faculty investigator will attend these meetings and will be responsible for preparing and presenting up-to-date statistical reports on the progress of their trials. These reports will include data on recruitment, randomization, adherence, as well as statistical tests and special analyses requested by the DSMB. The Administrative Core will be responsible for organizing the annual in-person meeting and semiannual conference calls. They will put together data and materials submitted by junior faculty investigators and deliver them to DSMB members. The COBRE investigators will meet with the DSMB in the Open Session and not be present for the Closed Session.

Bibliography and References Cited

1. The Emerging Risk Factors Collaboration. Diabetes mellitus, fasting blood glucose concentration, and risk of vascular disease: a collaborative meta-analysis of 102 prospective studies. *Lancet*. 2010;375(9733):2215-2222. doi:10.1016/S0140-6736(10)60484-9.
2. Flaxman SR, Bourne RRA, Resnikoff S, et al. Global causes of blindness and distance vision impairment 1990-2020: a systematic review and meta-analysis. *Lancet Glob Heal*. October 2017. doi:10.1016/S2214-109X(17)30393-5.
3. United States Renal Data System. *2016 USRDS Annual Data Report: Epidemiology of Kidney Disease in the United States*. Bethesda, MD: National Institutes of Health, National Institute of Diabetes and Digestive and Kidney Diseases; 2016. https://www.usrds.org/2016/view/Default.aspx.
4. Narres M, Kvitkina T, Claessen H, et al. Incidence of lower extremity amputations in the diabetic compared with the non-diabetic population: A systematic review. *PLoS One*. 2017;12(8):e0182081. doi:10.1371/journal.pone.0182081.
5. Centers for Disease Control and Prevention. *National Diabetes Statistics Report, 2017*. Atlanta, GA: Centers for Disease Control and Prevention, U.S. Dept of Health and Human Services; 2017.
6. Centers for Disease Control and Prevention. *Long-Term Trends in Diabetes*. Atlanta, GA: Centers for Disease Control and Prevention, U.S. Dept of Health and Human Services; 2017.
7. NCD Risk Factor Collaboration (NCD-RisC). Worldwide trends in diabetes since 1980: A pooled analysis of 751 population-based studies with 4.4 million participants. *Lancet*. 2016;387(10027):1513-1530. doi:10.1016/S0140-6736(16)00618-8.
8. American Diabetes Association. Economic costs of diabetes in the U.S. in 2012. *Diabetes Care*. 2013;36(4):1033-1046. doi:10.2337/dc12-2625.
9. Zhuo X, Zhang P, Barker L, Albright A, Thompson TJ, Gregg E. The lifetime cost of diabetes and its implications for diabetes prevention. *Diabetes Care*. 2014;37(9):2557-2564. doi:10.2337/dc13-2484.
10. WHO, International Diabetes Foundation. *Definition and Diagnosis of Diabetes Mellitus and Intermediate Hyperglycaemia: Report of a WHO/IDF Consultation*. Geneva: Geneva: World Health Organization; 2006.
11. The International Expert Committee. International Expert Committee Report on the Role of the A1C Assay in the Diagnosis of Diabetes. *Diabetes Care*. 2009;32(7):1327-1334. doi:10.2337/dc09-9033.
12. American Diabetes Association. Standards of Medical Care in Diabetes - 2017. *Diabetes Care*. 2017;40(Supplement 1):S33-S43. doi:10.2337/dc16-S003.
13. Gerstein HC, Santaguida P, Raina P, et al. Annual incidence and relative risk of diabetes in people with various categories of dysglycemia: A systematic overview and meta-analysis of prospective studies. *Diabetes Res Clin Pract*. 2007;78(3):305-312. doi:10.1016/j.diabres.2007.05.004.
14. Huang Y, Cai X, Mai W, Li M, Hu Y. Association between prediabetes and risk of cardiovascular disease and all cause mortality: systematic review and meta-analysis. *BMJ*. 2016;355:i5953. doi:10.1136/bmj.i5953.
15. Tabák AG, Herder C, Rathmann W, Brunner EJ, Kivimäki M. Prediabetes: A high-risk state for diabetes development. *Lancet*. 2012;379(9833):2279-2290. doi:10.1016/S0140-

6736(12)60283-9.

16. Diabetes Prevention Program Research Group. 10-year follow-up of diabetes incidence and weight loss in the Diabetes Prevention Program Outcomes Study. *Lancet*. 2009;374(9702):1677-1686. doi:10.1016/S0140-6736(09)61457-4.

17. The DREAM (Diabetes REduction Assessment with ramipril and rosiglitazone Medication) Trial Investigators. Effect of rosiglitazone on the frequency of diabetes in patients with impaired glucose tolerance or impaired fasting glucose: a randomised controlled trial. *Lancet*. 2006;368(9541):1096-1105. doi:10.1016/S0140-6736(06)69420-8.

18. Perreault L, Pan Q, Mather KJ, Watson KE, Hamman RF, Kahn SE. Effect of regression from prediabetes to normal glucose regulation on long-term reduction in diabetes risk: Results from the Diabetes Prevention Program Outcomes Study. *Lancet*. 2012;379(9833):2243-2251. doi:10.1016/S0140-6736(12)60525-X.

19. Perreault L, Temprosa M, Mather KJ, et al. Regression from prediabetes to normal glucose regulation is associated with reduction in cardiovascular risk: Results from the diabetes prevention program outcomes study. *Diabetes Care*. 2014;37(9):2622-2631. doi:10.2337/dc14-0656.

20. Popkin BM. Nutrition Transition and the Global Diabetes Epidemic. *Curr Diab Rep*. 2015;15(9). doi:10.1007/s11892-015-0631-4.

21. Vazquez G, Duval S, Jacobs DRJ, Silventoinen K. Comparison of body mass index, waist circumference, and waist/hip ratio in predicting incident diabetes: a meta-analysis. *Epidemiol Rev*. 2007;29:115-128. doi:10.1093/epirev/mxm008.

22. Knowler WC, Barrett-Connor E, Fowler SE, et al. Reduction in the incidence of type 2 diabetes with lifestyle intervention or metformin. *N Engl J Med*. 2002;346(6):393-403. doi:10.1056/NEJMoa012512.

23. Pan XR, Li GW, Hu YH, et al. Effects of diet and exercise in preventing NIDDM in people with impaired glucose tolerance: The Da Qing IGT and diabetes study. *Diabetes Care*. 1997;20(4):537-544. doi:10.2337/diacare.20.4.537.

24. Tuomilehto J, Indstrom J, Eriksson J, Valle T, Hamalainine E, Uusitupa M. Prevention of Type 2 Diabetes Mellitus By Changes in Lifestyle Among Subjects With Impaired Glucose Tolerance. *N Engl J Med*. 2001;344(18):1343-1350. doi:10.1056/NEJM200105033441801.

25. Ramachandran A, Snehalatha C, Mary S, Mukesh B, Bhaskar AD, Vijay V. The Indian Diabetes Prevention Programme shows that lifestyle modification and metformin prevent type 2 diabetes in Asian Indian subjects with impaired glucose tolerance (IDPP-1). *Diabetologia*. 2006;49(2):289-297. doi:10.1007/s00125-005-0097-z.

26. The Diabetes Preventio Program Research Group. The Diabetes Prevention Program. Design and methods for a clinical trial in the prevention of type 2 diabetes. *Diabetes Care*. 1999;22(4):623-634. doi:10.2337/diacare.22.4.623 10.2337/diabetes.48.4.699.

27. Evert AB, Boucher JL, Cypress M, et al. Nutrition therapy recommendations for the management of adults with diabetes. *Diabetes Care*. 2013;36(11):3821-3842. doi:10.2337/dc13-2042.

28. Hu T, Mills KT, Yao L, et al. Effects of low-carbohydrate diets versus low-fat diets on metabolic risk factors: A meta-analysis of randomized controlled clinical trials. *Am J Epidemiol*. 2012;176(SUPPL. 7). doi:10.1093/aje/kws264.

29. Sackner-Bernstein J, Kanter D, Kaul S. Dietary Intervention for Overweight and Obese Adults: Comparison of Low-Carbohydrate and Low-Fat Diets. A Meta-Analysis. *PLoS*

One. 2015;10(10):e0139817. doi:10.1371/journal.pone.0139817.

30. Bazzano LA, Hu T, Reynolds K, et al. Effects of low-carbohydrate and low-fat diets: A randomized trial. *Ann Intern Med*. 2014;161(5):309-318. doi:10.7326/M14-0180.
31. Sawyer L, Gale EAM. Diet, delusion and diabetes. *Diabetologia*. 2009;52(1):1-7. doi:10.1007/s00125-008-1203-9.
32. Snorgaard O, Poulsen GM, Andersen HK, Astrup A. Systematic review and meta-analysis of dietary carbohydrate restriction in patients with type 2 diabetes. *BMJ Open Diabetes Res Care*. 2017;5(1):e000354. doi:10.1136/bmjdrc-2016-000354.
33. Larsen RN, Mann NJ, Maclean E, Shaw JE. The effect of high-protein, low-carbohydrate diets in the treatment of type 2 diabetes: A 12 month randomised controlled trial. *Diabetologia*. 2011;54(4):731-740. doi:10.1007/s00125-010-2027-y.
34. Saslow LR, Kim S, Daubenmier JJ, et al. A randomized pilot trial of a moderate carbohydrate diet compared to a very low carbohydrate diet in overweight or obese individuals with type 2 diabetes mellitus or prediabetes. *PLoS One*. 2014;9(4). doi:10.1371/journal.pone.0091027.
35. Tay J, Luscombe-Marsh ND, Thompson CH, et al. A very low-carbohydrate, low-saturated fat diet for type 2 diabetes management: A randomized trial. *Diabetes Care*. 2014;37(11):2909-2918. doi:10.2337/dc14-0845.
36. Davis NJ, Tomuta N, Schechter C, et al. Comparative Study of the Effects of a 1-Year Dietary Intervention of a Low- Carbohydrate Diet Versus a Low-Fat Diet on Weight and Glycemic Control in Type 2. *Diabetes Care*. 2009;32(7):1147-1152. doi:10.2337/dc08-2108.Clinical.
37. Saslow LR, Daubenmier JJ, Moskowitz JT, et al. Twelve-month outcomes of a randomized trial of a moderate-carbohydrate versus very low-carbohydrate diet in overweight adults with type 2 diabetes mellitus or prediabetes. *Nutr Diabetes*. 2017;7(12):304. doi:10.1038/s41387-017-0006-9.
38. Tay J, Thompson CH, Luscombe-Marsh ND, et al. Effects of an energy-restricted low-carbohydrate, high unsaturated fat/low saturated fat diet versus a high carbohydrate, low fat diet in type 2 diabetes: a 2 year randomized clinical trial. *Diabetes, Obes Metab*. 2017:n/a-n/a. doi:10.1111/dom.13164.
39. Yamada Y, Uchida J, Izumi H, et al. A non-calorie-restricted low-carbohydrate diet is effective as an alternative therapy for patients with type 2 diabetes. *Intern Med*. 2014;53(1):13-19. doi:http://dx.doi.org/10.2169/internalmedicine.53.0861.
40. Mayer SB, Jeffreys AS, Olsen MK, Mcduffie JR, Feinglos MN, Yancy WS. Two diets with different haemoglobin A1c and antiglycaemic medication effects despite similar weight loss in type 2 diabetes. *Diabetes, Obes Metab*. 2014;16(1):90-93. doi:10.1111/dom.12191.
41. Maekawa S, Kawahara T, Nomura R, et al. Retrospective study on the efficacy of a low-carbohydrate diet for impaired glucose tolerance. *Diabetes Metab Syndr Obes*. 2014;7:195-201. doi:10.2147/DMSO.S62681.
42. Davis C, Bryan J, Hodgson J, Murphy K. Definition of the mediterranean diet: A literature review. *Nutrients*. 2015;7(11):9139-9153. doi:10.3390/nu7115459.
43. Sofi F, Macchi C, Abbate R, Gensini GF, Casini A. Mediterranean diet and health status: an updated meta-analysis and a proposal for a literature-based adherence score. *Public Health Nutr*. 2014;17(12):2769-2782. doi:10.1017/S1368980013003169.
44. Jannasch F, Kröger J, Schulze MB. Dietary Patterns and Type 2 Diabetes: A Systematic Literature Review and Meta-Analysis of Prospective Studies. *J Nutr*. 2017;147(6):1174-

1182. doi:10.3945/jn.116.242552.

45. Schwingshackl L, Missbach B, König J, Hoffmann G. Adherence to a Mediterranean diet and risk of diabetes: a systematic review and meta-analysis. *Public Health Nutr*. 2014;18(7):1292–1299. doi:10.1017/S1368980014001542.

46. de Lorgeril M, Salen P, Martin J-L, Monjaud I, Delaye J, Mamelle N. Mediterranean Diet, Traditional Risk Factors, and the Rate of Cardiovascular Complications After Myocardial Infarction : Final Report of the Lyon Diet Heart Study. *Circulation*. 1999;99(6):779-785. doi:10.1161/01.CIR.99.6.779.

47. Estruch R, Ros E, Salas-Salvadó J, et al. Primary Prevention of Cardiovascular Disease with a Mediterranean Diet. *N Engl J Med*. 2013;368(14):1279-1290. doi:10.1056/NEJMoa1200303.

48. Salas-Salvadó J, Bulló M, Estruch R, et al. Prevention of diabetes with mediterranean diets: A subgroup analysis of a randomized trial. *Ann Intern Med*. 2014;160(1):1-10. doi:10.7326/M13-1725.

49. Esposito K, Marfella R, Ciotola M, et al. Effect of a Mediterranean-Style Diet on Endothelial Dysfunction and Markers of Vascular Inflammation in the Metabolic Syndrome. *JAMA*. 2004;292(12):1440. doi:10.1001/jama.292.12.1440.

50. Babio N, Toledo E, Estruch R, et al. Mediterranean diets and metabolic syndrome status in the PREDIMED randomized trial. *CMAJ*. 2014;186(17):E649-E657. doi:10.1503/cmaj.140764.

51. Esposito K, Maiorino MI, Bellastella G, Chiodini P, Panagiotakos D, Giugliano D. A journey into a Mediterranean diet and type 2 diabetes: a systematic review with meta-analyses. *BMJ Open*. 2015;5(8):e008222. doi:10.1136/bmjopen-2015-008222.

52. Micha R, Michas G, Mozaffarian D. Unprocessed red and processed meats and risk of coronary artery disease and type 2 diabetes--an updated review of the evidence. *Curr Atheroscler Rep*. 2012;14(6):515-524. doi:10.1007/s11883-012-0282-8.

53. Esposito K, Maiorino MI, Ciotola M, et al. Effects of a Mediterranean-style diet on the need for antihyperglycemic drug therapy in patients with newly diagnosed type 2 diabetes: A randomized trial. *Ann Intern Med*. 2009;151(5):306-314. doi:10.1097/OGX.0b013e3181e5a159.

54. Esposito K, Maiorino MI, Petrizzo M, Bellastella G, Giugliano D. The effects of a Mediterranean diet on the need for diabetes drugs and remission of newly diagnosed type 2 diabetes: Follow-up of a randomized trial. *Diabetes Care*. 2014;37(7):1824-1830. doi:10.2337/dc13-2899.

55. Elhayany A, Lustman A, Abel R, Attal-Singer J, Vinker S. A low carbohydrate Mediterranean diet improves cardiovascular risk factors and diabetes control among overweight patients with type 2 diabetes mellitus: A 1-year prospective randomized intervention study. *Diabetes, Obes Metab*. 2010;12(3):204-209. doi:10.1111/j.1463-1326.2009.01151.x.

56. Shai I, Schwarzfuchs D, Henkin Y, et al. Weight Loss with a Low-Carbohydrate, Mediterranean, or Low-Fat Diet. *N Engl J Med*. 2008;359(3):229-241. doi:10.1056/NEJMoa0708681.

57. Schwarzfuchs D, Golan R, Shai I. Four-Year Follow-up after Two-Year Dietary Interventions. *N Engl J Med*. 2012;367(14):1373-1374. doi:10.1056/NEJMc1204792.

58. Pan A, Sun Q, Bernstein AM, et al. Red meat consumption and risk of type 2 diabetes: 3 Cohorts of US adults and an updated meta-analysis. *Am J Clin Nutr*. 2011;94(4):1088-1096. doi:10.3945/ajcn.111.018978.

59. Hu EA, Pan A, Malik V, Sun Q. White rice consumption and risk of type 2 diabetes: meta-analysis and systematic review. *BMJ*. 2012;344:e1454. doi:10.1136/bmj.e1454.

60. Wang M, Yu M, Fang L, Hu R-Y. Association between sugar-sweetened beverages and type 2 diabetes: A meta-analysis. *J Diabetes Investig*. 2015;6(3):360-366. doi:10.1111/jdi.12309.

61. Imamura F, O'Connor L, Ye Z, et al. Consumption of sugar sweetened beverages, artificially sweetened beverages, and fruit juice and incidence of type 2 diabetes: systematic review, meta-analysis, and estimation of population attributable fraction. *BMJ*. 2015:h3576. doi:10.1136/bmj.h3576.

62. Carter P, Gray LJ, Troughton J, Khunti K, Davies MJ. Fruit and vegetable intake and incidence of type 2 diabetes mellitus: systematic review and meta-analysis. *BMJ*. 2010;341:c4229. doi:10.1136/bmj.c4229.

63. Cooper AJ, Forouhi NG, Ye Z, et al. Fruit and vegetable intake and type 2 diabetes: EPIC-InterAct prospective study and meta-analysis. *Eur J Clin Nutr*. 2012;66(10):1082-1092. doi:10.1038/ejcn.2012.85.

64. Chen M, Sun Q, Giovannucci E, et al. Dairy consumption and risk of type 2 diabetes: 3 cohorts of US adults and an updated meta-analysis. *BMC Med*. 2014;12(1):215. doi:10.1186/s12916-014-0215-1.

65. Aune D, Norat T, Romundstad P, Vatten LJ. Whole grain and refined grain consumption and the risk of type 2 diabetes: A systematic review and dose-response meta-analysis of cohort studies. *Eur J Epidemiol*. 2013;28(11):845-858. doi:10.1007/s10654-013-9852-5.

66. Ding M, Bhupathiraju SN, Chen M, van Dam RM, Hu FB. Caffeinated and decaffeinated coffee consumption and risk of type 2 diabetes: a systematic review and a dose-response meta-analysis. *Diabetes Care*. 2014;37(2):569-586. doi:10.2337/dc13-1203.

67. Li X-H, Yu F-F, Zhou Y-H, He J. Association between alcohol consumption and the risk of incident type 2 diabetes: a systematic review and dose-response meta-analysis. *Am J Clin Nutr*. 2016;103(3):818-829. doi:10.3945/ajcn.115.114389.

68. Muraki I, Imamura F, Manson JE, et al. Fruit consumption and risk of type 2 diabetes: results from three prospective longitudinal cohort studies. *BMJ*. 2013;347(aug28 1):f5001-f5001. doi:10.1136/bmj.f5001.

69. Leong A, Daya N, Porneala B, et al. Prediction of Type 2 Diabetes by Hemoglobin A1c in Two Community-Based Cohorts. *Diabetes Care*. 2017;41(1):dc170607. doi:10.2337/dc17-0607.

70. Zhang X, Gregg EW, Williamson DF, et al. A1C level and future risk of diabetes: A systematic review. *Diabetes Care*. 2010. doi:10.2337/dc09-1939.

71. Maruthur NM, Ma Y, Delahanty LM, et al. Early response to preventive strategies in the diabetes prevention program. *J Gen Intern Med*. 2013;28(12):1629-1636. doi:10.1007/s11606-013-2548-4.

72. Khaw K-T, Wareham N, Bingham S, Luben R, Welch A, Day N. Association of hemoglobin A1c with cardiovascular disease and mortality in adults: the European prospective investigation into cancer in Norfolk. *Ann Intern Med*. 2004;141(6):413-420. doi:10.7326/0003-4819-141-6-200409210-00006.

73. Sarwar N, Aspelund T, Eiriksdottir G, et al. Markers of dysglycaemia and risk of coronary heart disease in people without diabetes: Reykjavik prospective study and systematic review. *PLoS Med*. 2010;7(5). doi:10.1371/journal.pmed.1000278.

74. Stratton IM, Adler AI, Neil HAW, et al. Association of glycaemia with macrovascular and prospective observational study. *Br Med J*. 2000:405-412.

doi:10.1136/bmj.321.7258.405.

75. Upadhyaya S, Banerjee G. Type 2 diabetes and gut microbiome: At the intersection of known and unknown. *Gut Microbes*. 2015;6(2):85-92. doi:10.1080/19490976.2015.1024918.

76. David LA, Maurice CF, Carmody RN, et al. Diet rapidly and reproducibly alters the human gut microbiome. *Nature*. 2014;505(7484):559-563. doi:10.1038/nature12820.

77. Duncan SH, Belenguer A, Holtrop G, Johnstone AM, Flint HJ, Lobley GE. Reduced dietary intake of carbohydrates by obese subjects results in decreased concentrations of butyrate and butyrate-producing bacteria in feces. *Appl Environ Microbiol*. 2007;73(4):1073-1078. doi:10.1128/AEM.02340-06.

78. Dorans KS, Mostofsky E, Levitan EB, Håkansson N, Wolk A, Mittleman MA. Alcohol and Incident Heart Failure among Middle-Aged and Elderly Men: Cohort of Swedish Men. *Circ Hear Fail*. 2015;8(3):422-427. doi:10.1161/CIRCHEARTFAILURE.114.001787.

79. Steinhaus DA, Mostofsky E, Levitan EB, et al. Chocolate intake and incidence of heart failure: Findings from the Cohort of Swedish Men. *Am Heart J*. 2017;183:18-23. doi:10.1016/j.ahj.2016.10.002.

80. Dorans KS, Massa J, Chitnis T, Ascherio A, Munger KL. Physical activity and the incidence of multiple sclerosis. *Neurology*. 2016;87(17):1770-1776. doi:10.1212/WNL.0000000000003260.

81. Mills KT, Chen J, Yang W, et al. Sodium Excretion and the Risk of Cardiovascular Disease in Patients With Chronic Kidney Disease. *JAMA*. 2016;315(20):2200. doi:10.1001/jama.2016.4447.

82. Chen J, Gu D, Huang J, et al. Metabolic syndrome and salt sensitivity of blood pressure in non-diabetic people in China: a dietary intervention study. *Lancet*. 2009;373(9666):829-835. doi:10.1016/S0140-6736(09)60144-6.

83. Li C, He J, Chen J, et al. Genome-Wide Gene-Sodium Interaction Analyses on Blood Pressure: The Genetic Epidemiology Network of Salt-Sensitivity Study. *Hypertension*. 2016;68(2):348-355. doi:10.1161/HYPERTENSIONAHA.115.06765.

84. He J, Streiffer RH, Muntner P, Krousel-Wood MA, Whelton PK. Effect of dietary fiber intake on blood pressure: a randomized, double-blind, placebo-controlled trial. *J Hypertens*. 2004;22(1):73-80. doi:10.1097/01.hjh.0000098164.70956.54.

85. He J, Ogden LG, Vupputuri S, Bazzano LA, Loria C, Whelton PK. Dietary sodium intake and subsequent risk of cardiovascular disease in overweight adults. *JAMA*. 1999;282(21):2027-2034. doi:10.1001/jama.282.21.2027.

86. He J, Wofford MR, Reynolds K, et al. Effect of dietary protein supplementation on blood pressure a randomized, controlled trial. *Circulation*. 2011;124(5):589-595. doi:10.1161/CIRCULATIONAHA.110.009159.

87. Thompson AM, Zhang Y, Tong W, et al. Association of inflammation and endothelial dysfunction with metabolic syndrome, prediabetes and diabetes in adults from Inner Mongolia, China. *BMC Endocr Disord*. 2011;11(1):16. doi:10.1186/1472-6823-11-16.

88. Irazola V, Rubinstein A, Bazzano L, et al. Prevalence, awareness, treatment and control of diabetes and impaired fasting glucose in the Southern Cone of Latin America. *PLoS One*. 2017;12(9). doi:10.1371/journal.pone.0183953.

89. Bazzano LA, Li TY, Joshipura KJ, Hu FB. Intake of fruit, vegetables, and fruit juices and risk of diabetes in women. *Diabetes Care*. 2008;31(7):1311-1317. doi:10.2337/dc08-0080.

90. Bazzano LA, Serdula M, Liu S. Prevention of Type 2 Diabetes by Diet and Lifestyle Modification. *J Am Coll Nutr*. 2005;24(5):310-319. doi:10.1080/07315724.2005.10719479.

91. Pollock BD, Chen W, Harville EW, et al. Differential sex effects of systolic blood pressure and low-density lipoprotein cholesterol on type 2 diabetes: Life course data from the Bogalusa Heart Study. *Journal of Diabetes*. 2017.

92. Zhang T, Li Y, Zhang H, et al. Insulin-sensitive adiposity is associated with a relatively lower risk of diabetes than insulin-resistant adiposity: the Bogalusa Heart Study. *Endocrine*. 2016;54(1):93-100. doi:10.1007/s12020-016-0948-z.

93. Pollock BD, Hu T, Chen W, et al. Utility of existing diabetes risk prediction tools for young black and white adults: Evidence from the Bogalusa Heart Study. *J Diabetes Complications*. 2016;31(1):86-93. doi:10.1016/j.jdiacomp.2016.07.025.

94. Hu T, Yao L, Reynolds K, et al. The effects of a low-carbohydrate diet vs. a low-fat diet on novel cardiovascular risk factors: A randomized controlled trial. *Nutrients*. 2015;7(9):7978-7994. doi:10.3390/nu7095377.

95. Hu T, Yao L, Reynolds K, et al. The effects of a low-carbohydrate diet on appetite: A randomized controlled trial. *Nutr Metab Cardiovasc Dis*. 2016;26(6):476-488. doi:10.1016/j.numecd.2015.11.011.

96. Rebholz CM, Reynolds K, Wofford MR, et al. Effect of soybean protein on novel cardiovascular disease risk factors: A randomized controlled trial. *Eur J Clin Nutr*. 2013;67(1):58-63. doi:10.1038/ejcn.2012.186.

97. Wofford MR, Rebholz CM, Reynolds K, et al. Effect of soy and milk protein supplementation on serum lipid levels: A randomized controlled trial. *Eur J Clin Nutr*. 2012;66(4):419-425. doi:10.1038/ejcn.2011.168.

98. Chen J, He J, Wildman RP, Reynolds K, Streiffer RH, Whelton PK. A randomized controlled trial of dietary fiber intake on serum lipids. *Eur J Clin Nutr*. 2006;60(1):62-68. doi:10.1038/sj.ejcn.1602268.

99. Overweight and Obesity Expert Panel. *Managing Overweight and Obesity in Adults: Systematic Evidence Review From the Obesity Expert Panel, 2013.*; 2013.

100. The Data Center. *Who Lives in New Orleans and Metro Parishes Now?*; 2017. https://www.datacenterresearch.org/data-resources/who-lives-in-new-orleans-now/.

101. Wetzels JFM, Kiemeney LALM, Swinkels DW, Willems HL, Heijer M Den. Age- and gender-specific reference values of estimated GFR in Caucasians: The Nijmegen Biomedical Study. *Kidney Int*. 2007;72(5):632-637. doi:10.1038/sj.ki.5002374.

102. Radin MS. Pitfalls in hemoglobin A1c measurement: When results may be misleading. *J Gen Intern Med*. 2014;29(2):388-394. doi:10.1007/s11606-013-2595-x.

103. Kim PS, Woods C, Georgoff P, et al. A1C underestimates glycemia in HIV infection. *Diabetes Care*. 2009;32(9):1591-1593. doi:10.2337/dc09-0177.

104. Qaseem A, Wilt TJ, Kansagara D, Horwitch C, Barry MJ, Forciea MA. Hemoglobin A1c Targets for Glycemic Control With Pharmacologic Therapy for Nonpregnant Adults With Type 2 Diabetes Mellitus: A Guidance Statement Update From the American College of Physicians. *Ann Intern Med*. 2018;168(8):569-576. doi:10.7326/M17-0939.

105. Sumithran P, Prendergast LA, Delbridge E, et al. Ketosis and appetite-mediating nutrients and hormones after weight loss. *Eur J Clin Nutr*. 2013;67(7):759-764. doi:10.1038/ejcn.2013.90.

106. Gardner CD, Trepanowski JF, Gobbo LCD, et al. Effect of low-fat VS low-carbohydrate

diet on 12-month weight loss in overweight adults and the association with genotype pattern or insulin secretion the DIETFITS randomized clinical trial. *JAMA - J Am Med Assoc*. 2018;319(7):667-679. doi:10.1001/jama.2018.0245.

107. Brenner BM, Meyer TW, Hostetter TH. Dietary protein intake and the progressive nature of kidney disease: the role of hemodynamically mediated glomerular injury in the pathogenesis of progressive glomerular sclerosis in aging, renal ablation, and intrinsic renal disease. *N Engl J Med*. 1982;307(11):652-659. doi:10.1056/NEJM198209093071104.

108. Sato J, Kanazawa A, Makita S, et al. A randomized controlled trial of 130 g/day low-carbohydrate diet in type 2 diabetes with poor glycemic control. *Clin Nutr*. 2017;36(4):992-1000. doi:10.1016/j.clnu.2016.07.003.

109. Yancy WS, Foy M, Chalecki AM, et al. A low-carbohydrate, ketogenic diet to treat type 2 diabetes. *Nutr Metab (Lond)*. 2005;2(1):34. doi:10.1186/1743-7075-2-34.

110. Boden G, Sargrad K, Homko C, Mozzoli M, Stein TP. Effect of a low-carbohydrate diet on appetite, blood glucose levels, and insulin resistance in obese patients with type 2 diabetes. *Ann Intern Med*. 2005;142(6). doi:10.1016/S0084-3741(08)70336-6.

111. Stern L, Iqbal N, Seshadri P. The Effects of Low-Carbohydrate versus Conventional Weight Loss Diets in Severely Obese Adults : One-Year Follow-up of a randomized control trial. *Ann Intern …*. 2004;140(May 2001):769-777. doi:10.1016/j.accreview.2004.07.103.

112. Westman EC, Yancy WS, Edman JS, Tomlin KF, Perkins CE. Effect of 6-month adherence to a very low carbohydrate diet program. *Am J Med*. 2002;113(1):30-36. doi:10.1016/S0002-9343(02)01129-4.

113. Moosheer SM, Waldschütz W, Itariu BK, Brath H, Stulnig TM. A protein-enriched low glycemic index diet with omega-3 polyunsaturated fatty acid supplementation exerts beneficial effects on metabolic control in type 2 diabetes. *Prim Care Diabetes*. 2014;8(4):308-314. doi:10.1016/j.pcd.2014.02.004.

114. Aagaard K, Petrosino J, Keitel W, et al. The Human Microbiome Project strategy for comprehensive sampling of the human microbiome and why it matters. *FASEB J*. 2013;27(3):1012-1022. doi:10.1096/fj.12-220806.

115. McInnes P, Cutting M. Manual of Procedures for Human Microbiome Project. http://www.hmpdacc.org/doc/HMP_MOP_Version12_0_072910.pdf.

116. Parker AR, Byham-Gray L, Denmark R, Winkle PJ. The Effect of Medical Nutrition Therapy by a Registered Dietitian Nutritionist in Patients with Prediabetes Participating in a Randomized Controlled Clinical Research Trial. *J Acad Nutr Diet*. 2014;114(11):1739-1748. doi:10.1016/j.jand.2014.07.020.

117. Marrero DG, Palmer KNB, Phillips EO, Miller-Kovach K, Foster GD, Saha CK. Comparison of commercial and self-initiated weight loss programs in people with prediabetes: A randomized control trial. *Am J Public Health*. 2016;106(5):949-956. doi:10.2105/AJPH.2015.303035.

118. Cole RE, Boyer KM, Spanbauer SM, Sprague D, Bingham M. Effectiveness of Prediabetes Nutrition Shared Medical Appointments. *Diabetes Educ*. 2013;39(3):344-353. doi:10.1177/0145721713484812.

119. Brownlee M, Hirsch IB. Glycemic variability: a hemoglobin A1c-independent risk factor for diabetic complications. *JAMA*. 2006;295(14):1707-1708. doi:10.1001/jama.295.14.1707.

120. Smith-Palmer J, Brändle M, Trevisan R, Orsini Federici M, Liabat S, Valentine W. Assessment of the association between glycemic variability and diabetes-related

complications in type 1 and type 2 diabetes. *Diabetes Res Clin Pract*. 2014;105(3):273-284. doi:10.1016/j.diabres.2014.06.007.

121. Nalysnyk L, Hernandez-Medina M, Krishnarajah G. Glycaemic variability and complications in patients with diabetes mellitus: Evidence from a systematic review of the literature. *Diabetes, Obes Metab*. 2010;12(4):288-298. doi:10.1111/j.1463-1326.2009.01160.x.

122. J. T, N.D. L-M, C.H. T, et al. Comparison of low- and high-carbohydrate diets for type 2 diabetes management: A randomized trial. *Am J Clin Nutr*. 2015;102(4):780-790. doi:10.3945/ajcn.115.112581.1.

123. Service FJ, Molnar GD, Rosevear JW, Ackerman E, Gatewood LC, Taylor WF. Mean amplitude of glycemic excursions, a measure of diabetic instability. *Diabetes*. 1970;19(9):644-655. doi:10.2337/diab.19.9.644.

124. Monnier L, Colette C, Owens DR. The application of simple metrics in the assessment of glycaemic variability. *Diabetes and Metabolism*. 2018.

125. Monnier L, Mas E, Ginet C, et al. Activation of oxidative stress by acute glucose fluctuations compared with sustained chronic hyperglycemia in patients with type 2 diabetes. *J Am Med Assoc*. 2006;295(14):1681-1687. doi:10.1001/jama.295.14.1681.

126. Torimoto K, Okada Y, Mori H, Tanaka Y. Relationship between fluctuations in glucose levels measured by continuous glucose monitoring and vascular endothelial dysfunction in type 2 diabetes mellitus. *Cardiovasc Diabetol*. 2013;12(1). doi:10.1186/1475-2840-12-1.

127. Su G, Mi S, Tao H, et al. Association of glycemic variability and the presence and severity of coronary artery disease in patients with type 2 diabetes. *Cardiovasc Diabetol*. 2011;10. doi:10.1186/1475-2840-10-19.

128. Diabetes Prevention Program Research Group. Reduction in the Incidence of Type 2 Diabetes With Lifestyle Intervention or Metformin. *N Engl J Med*. 2002;346(6):393-403. doi:10.1056/NEJMoa012512.

129. Hostalek U, Gwilt M, Hildemann S. Therapeutic Use of Metformin in Prediabetes and Diabetes Prevention. *Drugs*. 2015;75(10):1071-1094. doi:10.1007/s40265-015-0416-8.

130. Bailey T, Bode BW, Christiansen MP, Klaff LJ, Alva S. The Performance and Usability of a Factory-Calibrated Flash Glucose Monitoring System. *Diabetes Technol Ther*. 2015;17(11):787-794. doi:10.1089/dia.2014.0378.

131. Færch K, Amadid H, Nielsen LB, et al. Protocol for a randomised controlled trial of the effect of dapagliflozin, metformin and exercise on glycaemic variability, body composition and cardiovascular risk in prediabetes (the PRE-D Trial). *BMJ Open*. 2017;7(5). doi:10.1136/bmjopen-2016-013802.

132. Hanefeld M, Sulk S, Helbig M, Thomas A, Köhler C. Differences in glycemic variability between normoglycemic and prediabetic subjects. *J Diabetes Sci Technol*. 2014;8(2):286-290. doi:10.1177/1932296814522739.

133. Rodriguez-Segade S, Rodriguez J, Camiña F, et al. Continuous glucose monitoring is more sensitive than HbA1c and fasting glucose in detecting dysglycaemia in a Spanish population without diabetes. *Diabetes Res Clin Pract*. 2018;142:100-109. doi:10.1016/j.diabres.2018.05.026.

134. Le Floch JP, Escuyer P, Baudin E, Baudon D, Perlemuter L. Blood glucose area under the curve. Methodological aspects. *Diabetes Care*. 1990;13(2):172-175. doi:10.2337/diacare.13.2.172.

135. Vogelzang M, van der Horst ICC, Nijsten MWN. Hyperglycaemic index as a tool to

assess glucose control. *Crit Care*. 2004;8(3):R122-R127. doi:10.1186/cc2840.

Low-Carbohydrate Dietary Pattern on Glycemic Outcomes Trial

PROTOCOL

PROTOCOL VERSION 1.2

Dated: October 8, 2018

New Orleans, LA

Table of Contents

1. Overview ... 5
2. Background and Significance ... 6
2.A. Type 2 Diabetes Mellitus is a major global public health challenge ... 6
2.B. Prediabetes is a major risk factor for cardiometabolic diseases ... 6
2.C. Role of lifestyle in preventing diabetes ... 6
2.D. Low-to-moderate carbohydrate diets, weight loss, and cardiometabolic diseases 7
2.E. Mediterranean-style diet is associated with reduced risk of T2D and improved glycemic control ... 8
2.F. Evidence for low-carbohydrate diets that emphasize Mediterranean-style dietary components ... 8
2.G. Evidence for specific food groups and risk of T2D ... 8
2.H. Gut microbiota, diet and T2D ... 9
2.I. Clinical and public health significance of the proposed study ... 9
2.J. Innovation ... 9
3. Preliminary Studies ... 10
3.A. Summary of aggregate experience of the research team ... 10
3.B. Low-carbohydrate behavioral dietary intervention trial experience ... 10
3.C. Other dietary intervention trial experience ... 11
4. Study Design and Organization ... 11
4.A. Overview of trial design ... 11
4.B. Study outcomes ... 12
4.B.1. Primary outcome ... 12
4.B.2. Secondary outcomes ... 12
4.C Study Timeline ... 12
6. Study Participants ... 13
6.A. Eligibility criteria ... 13
7. Recruitment and Randomization ... 13
7.A. Recruitment ... 13
7.A.1. Mass mailing ... 14
7.A.2. Database searches and targeted mailing ... 14
7.A.3. Referrals ... 14
7.B. Randomization ... 14
7.C. Masking ... 14
7.D. Safety Considerations ... 15

8. Dietary Intervention Program 15
8.A. Low-carbohydrate dietary intervention group 15
8.B. Usual Diet Group 16
8.C. Physical Activity 16
8.D. Dietary Adherence 16
9. Safety of Dietary Intervention 16
10. Data Collection 17
10.A. Study visits 17
10.A.1. Screening/baseline visits 18
10.A.2. Follow-up visits and final visit 18
10.B Study measurements 19
10.B.1. Questionnaires 19
10.B.2. Dietary measurements 19
10.B.3. Assessment and management of adverse effects 19
10.B.4. BP and anthropometric measures 19
10.B.5. Blood samples 19
10.B.6. Urine collection 20
10.B.7. Stool Specimen Collection 20
11. Quality Assurance and Quality Control 20
11.A. Manual of procedures 20
11.B. Training and certification 21
12. Sample Size and Statistical Power 21
13. Data Management and Analysis 22
13.A. Data management 22
13.B. Data analysis plan 22
13.B.1. Exploratory data analysis 22
13.B.2. Primary,secondary, and exploratory outcomes 22
15. Anticipated Challenges 23
16. Protection of Human Subjects 23
16.A. Protection of human subjects from research risks 23
16.A.1. Risks to the subjects 23
16.A.2. Adequacy of protection against risks 24
16.A.3. Potential benefits of the proposed research to human subjects and others 26

16.A.4. Importance of knowledge to be gained 26
16.A.5. Remuneration 26
16.B. Data safety and monitoring plan 26
Bibliography and References Cited 28

1. Overview

The increasing prevalence of type 2 diabetes mellitus (T2D), a major cause of morbidity and mortality worldwide, is a critical public health concern. In the United States, an estimated 33.9% of adults have prediabetes (impaired fasting glucose, elevated hemoglobin A1c (HbA1c), or impaired glucose tolerance). Individuals with prediabetes are at elevated risk of T2D and cardiovascular disease (CVD), with 5–10% developing clinical T2D each year.

In the short-term, among patients with T2D, low-to-moderate carbohydrate diets (energy percentage below 45) have a greater glucose-lowering effect than do high-carbohydrate diets, with larger glucose-lowering effect the greater the carbohydrate reduction. Additionally, compared with low-fat diets, Mediterranean diets that are high in unsaturated fats (particularly *cis*-monounsaturated fats) reduce risk of T2D and CVD and improve glycemic control among diabetics. However, compared with usual diets, the effect of a behavioral intervention promoting a low-carbohydrate/high-unsaturated fat and protein dietary pattern that emphasizes consumption of non-starchy vegetables, plant-based oils, fish, poultry, and mixed nuts on glucose homeostasis, and other metabolic risk factors among individuals with prediabetes or early-stage untreated T2D is not well understood.

Our *long-term goal* is to develop and implement dietary intervention(s) that reduce risk of T2D. The *overall goal of this randomized controlled trial is to study the effect of a behavioral intervention promoting a low-carbohydrate/high-unsaturated fat and high-protein dietary pattern (initial target <40 g digestible carbohydrates, final target <60 g digestible carbohydrates) compared with a usual diet on metabolic risk factors among individuals with prediabetes or early-stage untreated diabetes* (HbA1c 6.0–6.9%). Specifically, we will test the following hypotheses:

Primary hypothesis: Compared with usual diet, over a 6-month period, a healthy low-carbohydrate/high unsaturated fat and high-protein dietary pattern will lead to *a larger decrease of HbA1c, by at least 0.17%.*

Secondary hypothesis: Over a 6-month period, a healthy low-carbohydrate/high-unsaturated fat and high-protein dietary pattern will lead to *larger improvements in the following major secondary outcomes*: fasting glucose, systolic blood pressure (BP), total-to-HDL-cholesterol ratio, and body weight.

Exploratory outcomes: We will also study the effect of the intervention on the following outcomes: insulin and homeostasis model assessment of insulin resistance (HOMA-IR); diastolic BP; waist circumference; and estimated CVD risk.

To achieve these study aims, we will recruit 150 adults with prediabetes or untreated diabetes (HbA1c 6.0–6.9%) in the Greater New Orleans, LA area. Using a stratified, blocked randomization scheme, participants will be assigned to either a behavioral modification intervention aimed at promoting a healthy low-carbohydrate dietary pattern or to usual diet. The intervention group will have an intensive phase for 3 months, followed by a maintenance phase in months 4 through 6. The *primary outcome will be difference between the active intervention and control groups in the change in HbA1c from baseline to 6 months of follow-up. We expect 85% power to detect a difference of at least 0.17% in change of HbA1c.* Changes in other metabolic risk factors will be studied concurrently. Metabolic risk factors will be assessed at baseline, 3 months, and 6 months of follow-up.

There is much interest regarding dietary approaches to prevent T2D and its complications. The results from this study will help to determine whether, compared with usual diet, a behavioral intervention aimed at promoting a healthy low-carbohydrate dietary pattern improves metabolic risk factors.

2. Background and Significance

2.A. Type 2 Diabetes Mellitus is a major global public health challenge

Diabetes is a strong and independent risk factor for CVD morbidity and mortality. A meta-analysis of 102 prospective studies (698,782 participants) estimated diabetes leads to an approximately 2-fold increased risk in vascular diseases, including coronary heart disease (CHD) and major stroke subtypes, and deaths due to other vascular causes.[1] Among participants with diabetes, CHD risk is about 50% higher among those with fasting blood glucose ≥7 mmol/L compared with those with fasting blood glucose <7 mmol/L.[1] In addition to increasing CVD risk, diabetes can cause diabetic retinopathy,[2] chronic kidney disease,[3] lower limb amputation,[4] and other complications. Most individuals with diabetes have T2D.[5] The US Centers for Disease Control and Prevention (CDC) estimated that 30.2 million adults ≥18 years (12.2%) had diabetes in 2015, with 7.2 million (23.8%) not being aware of or reporting diabetes.[5] Diagnosed diabetes prevalence among US adults has increased substantially over time, from 2.54% in 1980 to 7.40% in 2015.[6] Diabetes prevalence is also high globally, with an estimated 2014 age-standardized prevalence of 9.0% in men and 7.9% in women.[7] Diabetes accounts for an increasing portion of US healthcare expenditures. Total cost of diagnosed diabetes in the US was estimated to be $245 billion in 2012, 41% higher than in 2007.[8] Diabetes is associated with much higher lifetime medical expenses, with higher expenses for those diagnosed at younger ages.[9]

2.B. Prediabetes is a major risk factor for cardiometabolic diseases

Prediabetes, defined by the American Diabetes Association (ADA) as impaired fasting glucose (IFG), impaired glucose tolerance (IGT), or elevated HbA1c, is also referred to as “intermediate hyperglycemia”[10] and “high-risk state of developing diabetes”.[11] The ADA defines prediabetes as having: fasting plasma glucose of 100–125 mg/dL (IFG); 2-hour post-load glucose in the 75-g oral glucose tolerance test (OGTT) of 140–199 mg/dL (IGT); or HbA1c 5.7–6.4%.[12] Prediabetes itself is not a disease; for all three tests, “risk is continuous, extending below the lower limit of the range and becoming disproportionately greater at the higher end of the range”.[12] The CDC estimated that in 2015, 33.9% of US adults had prediabetes based on fasting glucose or HbA1c.[5] Among those with prediabetes, only 11.9% reported having being told this by a medical professional.[5] Approximately 5–10% of individuals with prediabetes develop diabetes within one year, depending on how prediabetes is defined.[13] In a meta-analysis, annual diabetes incidence was 15–19% among individuals with both IFG and IGT, while it was 4–6% in those with isolated IFG and 6–9% in those with isolated IGT.[13]

A recent meta-analysis (1,611,339 participants from 53 studies) reported that prediabetes as defined by the ADA is associated with higher risk of composite CVD events and CHD.[14] IFG and IGT were also positively associated with increased risk of stroke and all-cause mortality.[14] Prediabetes is linked with increased risk of other conditions typically thought of as diabetes complications, such as neuropathies and nephropathies.[15] Importantly, it is possible for individuals with prediabetes to revert to normoglycemia, as observed in observational studies[13] and lifestyle or drug intervention trials.[16,17] In the Diabetes Prevention Program (DPP), reversion to normoglycemia was associated with lower long-term T2D risk[18] and lower predicted CVD risk.[19]

2.C. Role of lifestyle in preventing diabetes

Much evidence from observational studies and clinical trials supports the critical role of diet and other lifestyle factors in preventing T2D. Globally, lifestyle transitions towards reduced physical activity, increased consumption of refined carbohydrates, coupled with resulting changes in body composition, has led to increases in diabetes prevalence and severity.[20] Body

mass index (BMI), waist circumference, and waist-to-height ratio are predictors of T2D.[21] The DPP, which combined calorie restriction and exercise to promote weight loss, substantially reduced risk of T2D (by 58%) among individuals with elevated FPG and IGT;[22] a benefit that persisted in 10-year follow-up analyses.[16] Other trials of lifestyle modification focused on diet and exercise have also shown benefits in study populations in China,[23] Finland,[24] and India.[25]

2.D. Low-to-moderate carbohydrate diets, weight loss, and cardiometabolic diseases

Low-to-moderate carbohydrate diets (<45% of energy from carbohydrates) are popular dietary approaches for losing weight. Weight loss is a key component of the DPP lifestyle intervention[26] and ADA lifestyle recommendations.[12,27] Meta-analyses from our group[28] and others support that low-to-moderate carbohydrate diets are at least as effective as low-fat diets at promoting weight loss. For instance, a meta-analysis reported a 2-kg (95% CI, 3.1–0.9) larger weight loss in low-carbohydrate diet (<120 g carbohydrates/day) arms compared with low-fat diet (<30% energy from fat) arms.[29] Estimated 10-year CVD risk was lower in low-carbohydrate than low-fat arms.[29] This aligns with research published by our team; in a 12-month trial of participants with obesity, a low-carbohydrate diet (target <40 g carbohydrates/day) was more effective than a low-fat diet (target <30% energy from fat, <7% saturated fat) for weight loss and CVD risk factor reduction.[30]

Low-to-moderate carbohydrate diets may also be beneficial among patients with T2D. Low-carbohydrate diets have come in and out of favor as treatment of diabetes.[31] In a meta-analysis (1376 patients;10 trials), after 3 or 6 months of the intervention, low-to-moderate carbohydrate diets (<45% energy from carbohydrates) led to a 0.34% lower HbA1c (95% CI, 0.06–0.63%) compared with high-carbohydrate diets.[32] Compared with high-carbohydrate diets, there was a greater reduction in HbA1c the greater the reported carbohydrate restriction.[32] There was no benefit of the low-to-moderate carbohydrate diets on HbA1c at 12 months or longer, and no difference between interventions on BMI, body weight, low-density lipoprotein (LDL) cholesterol or quality of life.[32] However, results may have been affected by dietary adherence or glucose medication. There was evidence of diabetes medication reduction in the low-carbohydrate arms in some studies,[33–37] with evidence of medication reduction and improved blood glucose stability persisting for up to 2 years.[38] Hypoglycemia (though not after medication adjustment) was observed in three subjects taking insulin or a sulfonylurea out of twelve subjects in the low-carbohydrate arm of another study.[39] In a subset of participants with T2D (n=46) from a large weight loss trial, compared with those randomized to a low-fat diet and orlistat, those randomized to a low-carbohydrate diet for 48 weeks had improved HbA1c (0.8% lower, 95% CI, -1.5, -0.02%) and a reduction in antiglycemic medications, despite similar weight loss effects of the interventions.[40]

Though low-to-moderate carbohydrate diets have been studied among overweight and obese participants (primarily focused on weight loss), less is understood about the effects of such diets on glycemic outcomes specifically among individuals with prediabetes. A study among participants with IGT observed a reduction at 12 months in weight, HbA1c, and other glycemic outcomes among individuals who participated in a 7-day in-hospital education program, though this was not randomized.[41] A randomized pilot trial of overweight or obese individuals with T2D or prediabetes found that, over 3 months and at 12 months, a very low-carbohydrate diet improved HbA1c, led to higher medication discontinuation, and more weight loss compared with a low-fat diet.[34,37] However, only 4 individuals had prediabetes. Given benefits of low-to-moderate carbohydrate diets for weight loss in general populations and for glycemic control among patients with T2D, further study of these diets are warranted among individuals with prediabetes who are at elevated cardiometabolic disease risk.

2.E. Mediterranean-style diet is associated with reduced risk of T2D and improved glycemic control

Though there is no one “Mediterranean diet”, the Mediterranean dietary pattern tends to have the following characteristics: high consumption of vegetables, fruits, breads and cereals, legumes, nuts and seeds; low-to-moderate consumption of dairy products, fish, poultry and eggs; little consumption of red meat and sweets; and red wine in moderation.[42] Olive oil is an important source of fat. In many observational studies, adherence to the Mediterranean-style dietary pattern is associated with reduced risk of CVD[43] and T2D.[44,45] Evidence from randomized trials also supports the benefits of this dietary pattern. In several trials, participants randomized to Mediterranean diets had substantially lower risk of CVD than those randomized to low-fat control diets.[46,47] In the three-arm randomized PREDIMED trial of participants at high risk of CVD, after 4.1 years of follow-up, those randomized to a traditional Mediterranean diet supplemented with either olive oil or nuts had a 30% reduced risk of T2D compared to those in the low-fat control diet group.[48] In all PREDIMED arms, average carbohydrate intake was relatively low (39.7–43.7% at end of trial).[47] Two trials have found a higher rate of remission from metabolic syndrome among individuals randomized to a Mediterranean diet compared with those randomized to control diets (prudent or low-fat) during 2–5 years of follow-up.[49,50] The Mediterranean pattern may also be beneficial among individuals with T2D. Meta-analyses have reported that, among patients with T2D, those randomized to a Mediterranean diet had lower HbA1c levels and CVD risk factors, and more significant weight loss, compared with those randomized to control diets.[51]

2.F. Evidence for low-carbohydrate diets that emphasize Mediterranean-style dietary components

Many low-carbohydrate diets involve increased consumption of food items, such as red meat,[52] that are associated with increased cardiometabolic disease risk. However, low-carbohydrate dietary approaches can focus on substituting carbohydrates with foods common in Mediterranean-style diets, such as olive oil and nuts, which may improve cardiometabolic health. In a 4-year Italian study of individuals with newly diagnosed T2D, those randomized to a low-carbohydrate Mediterranean diet (<50% carbohydrates; consumed ~44%) had more favorable changes in glycemic control, higher rates of T2D remission, and delayed need for antiglycemic drugs compared with participants randomized to a low-fat diet (<30% fat, <10% saturated fat).[53,54] A 12-month Israeli study assessed low-carbohydrate Mediterranean (35% carbohydrates), traditional Mediterranean, and ADA (both 50–55% carbohydrates) diets in 259 overweight participants with T2D.[55] Most CVD risk factors improved in all groups; there was only HDL improvement in the low-carbohydrate Mediterranean diet and this diet was superior to the other diets in improving glycemic control.[55] In a 2-year weight-loss study of moderately obese participants, low-carbohydrate and Mediterranean diets were superior to low-fat diet for weight loss.[56] Participants in the low-carbohydrate arm were counseled to choose vegetarian sources of fat and protein and to avoid trans fat. At 6 years after study commencement, despite weight regain, there were still long-term benefits, particularly for the low-carbohydrate and Mediterranean diets.[57] In whole, evidence supports that low-carbohydrate Mediterranean-style diets are feasible and may have metabolic benefits.

2.G. Evidence for specific food groups and risk of T2D

Prospective cohort studies have found consumption of specific foods and beverages is associated with diabetes risk. Meta-analyses report higher consumption of processed and unprocessed red meat,[58] white rice (particularly in Asian populations),[59] and sugar-sweetened beverages[60,61] are associated with higher T2D risk. Consumption of green leafy vegetables,[62,63] yogurt,[64] whole grains,[65] decaffeinated and total coffee,[66] and light-to-moderate alcohol

consumption[67] are associated with reduced risk. Consumption of fruits, particularly blueberries, grapes, and apples, are associated with reduced risk; fruit juice is associated with higher risk.[68]

2.H. Gut microbiota, diet and T2D

There is increasing evidence for the importance of gut microbiota in risk of type 2 diabetes and other cardiometabolic diseases.[75] Additionally, dietary modifications, including changes in carbohydrate consumption, can lead to substantial changes in gut microbiome over short periods of time.[76,77] Less is known about longer-term changes to the gut microbiota during the adoption of a low-carbohydrate diet and how these changes may influence cardiometabolic risk. We plan to collect stool specimens at baseline and at 6 months, for potential future studies on how this intervention affects the gut microbiome.

2.I. Clinical and public health significance of the proposed study

Prediabetes, or intermediate hyperglycemia, is a marker of high risk of developing T2D and other complications. Due to increasing prevalence of T2D and associated morbidity and mortality globally, a better understanding of dietary approaches to delay progression to T2D or to promote reversion to normoglycemia is a high public health priority. This study proposes to assess the effect of a behavioral intervention promoting a low-carbohydrate/high-unsaturated fat dietary pattern (initial target <40 g digestible carbohydrates, final target <60 g digestible carbohydrates) on HbA1c, a marker of glycemic control, and other metabolic risk factors. Building from prior findings from clinical and observational studies, we will develop a dietary intervention that promotes the consumption of non-starchy vegetables, plant-based oils, fish, poultry, and mixed nuts, that are thought to reduced risk of T2D or CVD. Participants assigned to the intervention will be trained in behavioral techniques that will provide capacity to maintain dietary changes long after study completion. Our outcome measures will assess the effect of this dietary intervention in a “real-life” setting as opposed to dietary modifications achieved in metabolic ward studies or through food prepared in research kitchens. Findings from this study may provide evidence in support of promoting alternative dietary styles among individuals with high T2D and CVD risk and could potentially be used to inform dietary interventions used in programs like DPP.

2.J. Innovation

The status quo as it pertains to dietary approaches for reducing risk of T2D is focused on reducing caloric intake and total fat intake. However, recent evidence suggests that the types of fat, rather than the quantity of fats may be more important, with evidence supporting that a Mediterranean-style diet that does not restrict fat intake reduces risk of developing both T2D[48] and CVD[47]. Moreover, in the short-term, low-carbohydrate diets, which are popular weight loss diets, have a greater glucose-lowering effect than do high-carbohydrate diets among patients with T2D.[32] However, the effect of low-carbohydrate diets that emphasize consumption of non-starchy vegetables, plant-based oils, fish, poultry, and mixed nuts on T2D prevention is unclear, particularly compared with higher-carbohydrate diets more commonly consumed in the US. *The proposed research is innovative, in our opinion, because it represents a substantial departure from the status quo by assessing the combined effect of a low-carbohydrate, high unsaturated fat and protein diet on cardiometabolic risk factors among adults with prediabetes or early-stage untreated diabetes.* We expect the results from this study have the potential to lead to new horizons for developing and implementing dietary approaches (other than the most commonly used reduced fat diet) that will substantially reduce risk of cardiometabolic disease among adults with T2D or at high risk of T2D. The proposed intervention dietary approach promotes consumption of dietary components thought to reduce risk of cardiometabolic disease and the behavior change intervention has expected applicability in clinical practice.

3. Preliminary Studies

3.A. Summary of aggregate experience of the research team

Dr. Kirsten Dorans is trained in cardiovascular disease epidemiology, with strong training in epidemiologic methods and biostatistics. Her publication record includes studies of behavioral risk factors and risk of chronic disease. She has published on the relationships between dietary intake and chronic heart failure[78,79] and on physical activity and incident multiple sclerosis[80]. She served as lead author, conducting data management, analysis, drafting, and revision of two of these manuscripts. In graduate school, she focused on studying links between outdoor air pollution and chronic diseases and has published several papers in this field. The COBRE program will provide an excellent opportunity for the JFI to transition from air pollution research to clinical and translational research focused on nutrition and diabetes. Building off of her training in observational epidemiology, this program will involve training and practical experience that will allow her to broaden her expertise and to develop practical skills for carrying out clinical research.

The mentorship team has much experience and prior successful collaborations in key areas related to this study. Dr. Jiang He is an internationally renowned expert in cardiometabolic disease clinical and epidemiological research. He has served as PI for many epidemiological studies and clinical trials, including clinical and observational studies focused on dietary consumption and chronic diseases[81–86] and has conducted extensive global research on diabetes.[87,88] Dr. Bazzano has collaborated extensively with Dr. He for more than 15 years in research related to diet and chronic disease. She is a clinical investigator and has expertise in nutritional epidemiology and clinical research. Dr. Bazzano was the PI of a highly successful clinical trial comparing the effects of low-carbohydrate and low-fat diets on weight and cardiovascular risk factors among individuals with obesity, which was a subproject within the Tulane COBRE in Hypertension and Renal Biology.[30] Dr. He served as Dr. Bazzano's mentor for this clinical trial. They have collaborated on much other work related to dietary and chronic disease, including a meta-analysis on low-carbohydrate diets.[28] Dr. Bazzano has also published many articles on diet and risk of diabetes[89,90] and on diabetes in the Bogalusa Heart Study.[91–93]

3.B. Low-carbohydrate behavioral dietary intervention trial experience

In the study on macronutrient composition of diet and risk factors for cardiovascular disease (MACRO) study, 148 individuals without clinical cardiovascular disease or diabetes and with obesity were randomly assigned to a low-carbohydrate or a low-fat behavioral dietary intervention for 12 months. In the low-carbohydrate arm, daily dietary intake of carbohydrates decreased from an average of 242 g at baseline to 97 g, 93 g, and 127 g at 3, 6, and 12 months, respectively. At 12 months, participants in the low-carbohydrate intervention group had greater decreases in weight (-3.5 kg; 95% CI, -5.6 to -1.4 kg) and fat mass (-1.5%; 95% CI, -2.6% to -0.4%) and greater improvement in several cardiovascular risk factors.[30] This suggests that restricting carbohydrates may be an option for weight loss and reduction of cardiovascular risk factors. Further analyses found that the low-carbohydrate dietary intervention led to similar or greater improvement in inflammation, adipocyte dysfunction, and endothelial dysfunction compared with the low-fat dietary intervention[94] and that the low-fat diet reduced peptide YY more than the low-carbohydrate diet, suggesting satiety may be better preserved on low-carbohydrate diets.[95] No serious adverse events were reported in this study and the number of participants with symptoms were similar between the two diets, with the exception of more participants having headaches on the low-fat diet at 3 months (18 vs 6 participants, $p = 0.03$).[30] Dr. Bazzano served as PI for this study and Dr. He served as her mentor. Dr. Bazzano

mentored one of her PhD students in conducting analyses and writing and editing manuscripts related to this project.

3.C. Other dietary intervention trial experience

Dr. He has also led or been involved with many dietary intervention trials, including both behavioral change and feeding study interventions. In the Protein and Blood Pressure (ProBP) study, 352 adults with prehypertension or stage 1 hypertension in New Orleans, LA and Jackson, MS were assigned to take, in a random order, 40 g/d soy protein, milk protein, or carbohydrate supplementation each for 8 weeks. Compared with the high-glycemic index refined carbohydrate, both soy protein and milk protein supplementation were associated with a -2.0 mm Hg (95% CI -3.2 to -0.7 mm Hg) and -2.3 mm Hg (-3.7 to -1.0 mm Hg) net change in systolic blood pressure.[86] These findings suggest that partial replacement of carbohydrate with soy or milk protein might be an important part of nutrition intervention strategies to prevent and treat hypertension. Additionally, soy protein supplementation led to reduction in plasma E-selectin compared with carbohydrate and milk protein and in plasma leptin compared with carbohydrate.[96] Soy protein also led to improvement in lipid profile compared with carbohydrate and milk protein.[97] Dr. He was PI for this project.

In the Fiber Intervention on Blood Pressure (FIBRE) study, 110 participants ages 30–65 years with untreated pre-hypertension or stage-1 hypertension and serum cholesterol level < 240 mg/dl were randomly assigned to 8-g/day of water soluble fiber from oat bran or a control intervention (double-blind RCT). Over the 12-week study period, net changes in systolic and diastolic blood pressure were -1.8 mm Hg (-4.3 to 0.8) and -1.2 mm Hg (-3.0 to 0.5 mm Hg), respectively.[84] Net changes in total, HDL-, and LDL-cholesterol were -2.40 mg/dL (-10.60 to 5.81 mg/d:), -1.66 mg/dL (-4.55 to 1.22 mg/dL), and -1.33 mg/dl (-8.33 to 5.68 mg/dL), respectively.[98] Dr He was PI for this study.

Dr. He is currently serving as a mentor for Dr. Katherine Mills' COBRE project (Effect of Dietary Sodium Reduction in Kidney Disease Patients with Albuminuria; SUPER). In this project, participants with chronic kidney disease and albuminuria are being randomized to a behavioral modification program designed to reduce dietary sodium intake to ≤2,300 mg/day or to usual care and test the effect of this intervention on change in albumin-to-creatinine ratio from baseline to 24-weeks.

4. Study Design and Organization

4.A. Overview of trial design

We propose to conduct a ***randomized controlled trial*** aimed at testing the effect of a behavioral intervention that promotes ***reduced carbohydrate intake and increased consumption of heart-healthy unsaturated fats and protein*** in patients with HbA1c 6.0–6.9% (***Figure 1***). We plan to recruit 150 individuals with elevated HbA1c and randomly assign them to a moderately intensive intervention on diet pattern,[99] using well-established methods for behavioral modification, or to usual diet. We will compare the difference between the active intervention and control groups for change in HbA1c from baseline to 6 months of follow-up (primary outcome). We will assess four major secondary outcomes: fasting plasma glucose, systolic BP, total-to-HDL cholesterol ratio, and body weight. In addition, we will look at several other exploratory outcomes: insulin and HOMA-IR, diastolic BP, waist circumference, and

Figure 1. Study Design

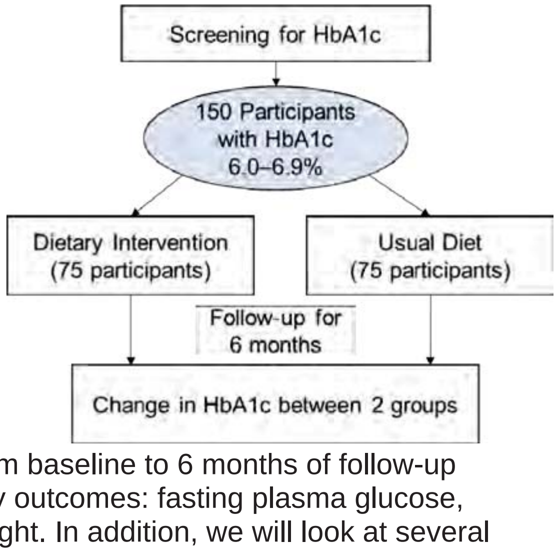

estimated CVD risk, as estimated by the 2013 ACC/AHA risk equation. The main trial components are summarized in ***Table 1***.

Table 1. Study Overview

Study Design	Randomized controlled trial
Study Participants	150 participants with elevated HbA1c (6.0–6.9%)
Intervention Group (n=75)	Behavioral modification to reduce carbohydrate consumption and promote consumption of foods such as non-starchy vegetables, plant-based unsaturated oils, nuts and seeds, avocados, eggs, seafood, poultry, lean meat and tofu. We will recommend moderate consumption of cheese, unsweetened Greek yogurt, and low-carb milk. If consuming higher-carbohydrate items, recommend legumes, fruits, (limited) whole grains. Target <40 g net carbohydrates per day for first 3 months and will have a goal of <40 net g carbohydrates per day; <60 g net carbohydrates per day for months 4 onwards.
Usual Diet Group (n=75)	No dietary intervention. To encourage continued participation in the trial, we will offer optional monthly educational sessions on unrelated topics, such as retirement planning or health insurance
Outcome Measures	Primary: HbA1c; Secondary: fasting plasma glucose, systolic BP, total-to-HDL-cholesterol ratio, and body weight

4.B. Study outcomes

4.B.1. Primary outcome: The *primary outcome will be the difference between the intervention group and the usual diet group for change in HbA1c* from baseline to 6 months. Changes in HbA1c will be calculated from blood samples taken at baseline and 6 months.
4.B.2. Secondary outcomes: Secondary outcomes will include changes from baseline to 6 months of *fasting plasma glucose, systolic BP, total-to-HDL cholesterol ratio, and body weight.,*
4.B.3. Other outcomes: We will also study the effect of the invention on the following outcomes: insulin and homeostasis model assessment of insulin resistance (HOMA-IR); diastolic BP; waist circumference; total-to-HDL-cholesterol ratio; and estimated CVD risk.

4.C Study Timeline

Table 2 shows the timeline, which will be followed in the implementation of study-related activities, such as publications and submission of an R01 grant application.

Table 2. Study Timeline

	Year 1		Year 2		Year 3	
Develop MOP, intervention program, study forms, and pilot testing	X	X				
Certification and training of staff	X					
Recruitment		X	X			
Intervention and data collection, measurement, entry, cleaning		X	X	X	X	
Data quality control and analysis						X
Publications	X	X	X	X	X	X
R01 grant submissions, resubmissions				X	X	X

The first 6 months will focus on development of manual of operations (MOP), intervention program, study forms, study protocol, Institutional Review Board (IRB) approval, and pilot testing of the intervention program. It will also involve staff training and certification. During the

last half of year 1, recruitment, intervention, and data collection will commence. During the last 3 months of year 3, data quality control and analysis will be carried out. Throughout the study period, the JFI will publish peer-reviewed manuscripts from ongoing Tulane University research projects.

6. Study Participants

6.A. Eligibility criteria

Inclusion criteria

- Men or women ages 40 to 70 years: aim to recruit 50% women
- Any race or ethnicity: aim to recruit 40% ethnic minorities (i.e., African American and Hispanic), close to population in the greater New Orleans area[100]
- HbA1c 6.0–6.9%
- Willing and able to provide informed consent

Exclusion criteria

- Diagnosed type 1 diabetes mellitus based on patient self-report
- Use of agents affecting glycemic control (medications for diabetes, oral glucocorticoids) within the past three months prior to enrollment based on patient self-report
- Medical condition in which low-carbohydrate diet may not be advised
  - Estimated glomerular filtration rate (eGFR) ≤45 mL/min/1.73 m^2, which is close to the 5th percentile of eGFR among non-diseased individuals of 70 years of age[101]
  - Self-report of liver disease due to hepatitis or alcohol; osteoporosis; untreated thyroid disease; gout; or cancer (other than non-melanoma skin cancer) requiring treatment in the past year, unless prognosis is excellent
- Factors that may affect HbA1c:
  - Hemoglobin <11 mg/dL (cutpoint for moderate-to-severe anemia, which could lead to falsely elevated or lowered HbA1c)[102]
  - Recent blood donation or blood transfusion (self-report, past 4 months)
  - Human Immunodeficiency Virus (self-report)[103]
- Allergies to nuts
- For women, current pregnancy, breastfeeding, or plans to become pregnant during the study
- Consumption of ≥21 alcoholic drinks per week or consumption of ≥6 drinks per occasion
- Current or planned residence making it difficult to meet trial requirements (due to distance from study site and/or challenges regularly traveling to site)
- Current participation in another lifestyle intervention trial or a pharmaceutical trial
- Participation of another household member in the study; employees or persons living with employees of the study
- Other concerns regarding ability to meet trial requirements, at the discretion of the principal investigator or study coordinator

7. Recruitment and Randomization

7.A. Recruitment

We will recruit study participants from the greater New Orleans area. The initial focus of our recruitment will be mass mailing of individuals in the greater New Orleans area and recruitment through placing flyers and brochures in the community. If community-based recruitment is not successful or cost-effective, we will recruit out-patients from the Ochsner Health System and Tulane Medical Center (TMC), from which our group has prior experience recruiting patients.

For this recruitment, we will focus on electronic searches of medical records and laboratory databases. We will also carry out targeted solicitation of referrals from physicians who care for patients likely to meet our inclusion and exclusion criteria.

In addition to this recruitment plan, we have several contingency plans: (1) extending recruitment to other medical centers in the New Orleans area, including the Veterans Affairs Medical Center (VAMC), East Jefferson General Hospital, and West Jefferson Medical Center, with whom Tulane has well-established relationships; (2) TV/radio advertisements. We have ample experience with each of these approaches.

Based on U.S. Census Bureau 2016 population estimates, the metro New Orleans area population was approximately 35% African American or black and 9% Hispanic.[100] Given our prior experience and population distribution in this region, we plan to recruit a distribution of study participants with 40% ethnic minorities.

7.A.1. Mass mailing: Mass mailing individuals at high risk for prediabetes or T2D, i.e. individuals who are obese or overweight.

7.A.2. Database searches and targeted mailing: We will request IRB approval for a waiver for HIPAA authorization to conduct database searching and hospital chart reviews for identification of potentially eligible study participants. After identifying a potentially eligible patient, we will send this individual an invitation letter signed by the investigators and/or the individual's personal physician. Individuals who express interest in the study will have a pre-screening telephone interview for confirmation of potential eligibility. Those still interested and potentially eligible for enrollment will be scheduled for a visit at the COBRE study clinic.

7.A.3. Referrals: Potential participants referred by their physician will have a pre-screening telephone interview to confirm potential eligibility. Those who are still interested and potentially eligible for the study will be scheduled for a screening visit.

7.B. Randomization

Once eligibility has been confirmed, the study coordinator will ensure that all key variables have been entered into the trial-specific database. Additionally, the study coordinator will ensure any outliers have been recognized and reconciled, and will ensure receipt of informed consent prior to enrolling the volunteer in the study as a trial participant. Eligible participants will be *randomly assigned to the behavioral intervention or the usual diet group in a 1:1 allocation ratio*. Randomization will be stratified by sex, *using a random block group size (4, 6) to maintain balance among groups over time*. A statistician from the Methodology/Biostatistics Unit within the COBRE Clinical Research Core, who is not directly involved in the study and is not located at the clinic, will conduct the randomization. After ascertaining eligibility, clinical personnel will telephone the Methodology/Biostatistics Unit to obtain a randomization assignment. All randomization assignments will be created by a computer program and only accessible to the Staff at the Methodology/Biostatistics Unit. Once randomization assignment has been made, trial participants will be considered to be officially randomized into the study and every effort will be made to obtain complete follow-up information.

7.C. Masking

Clinical and laboratory staff members who assess outcomes will be blinded to the intervention assignment of participants. As this is a dietary intervention trial, study participants, and study staff members who conduct the intervention cannot be blinded to the intervention assignment. Participants will be instructed to avoid sharing knowledge of their treatment assignment with data collection staff members. We will have intervention visits and data collection study visits on different days and in separate locations. Data collectors will also use

verbal cues and visual props to remind participants not to disclose treatment assignment. Investigators will be blinded to intervention assignment.

7.D. Safety Considerations

Participants with HbA1c >7.9% will be referred to a clinician for follow-up[104], though we will continue to collect data on these participants.

8. Dietary Intervention Program

8.A. Low-carbohydrate dietary intervention group

Participants randomized to the low-carbohydrate intervention group will receive counseling aimed at reducing carbohydrate consumption. Phase I (*Go Low*) will occur for the first 3 months, with a goal of <40 g digestible carbohydrates per day. Key recommended foods will include: non-starchy vegetables, fish, poultry, lean meat, eggs, olive oil and other plant-based unsaturated oils, unsweetened/unsalted nuts and seeds, nut butters, and avocados. We will recommend moderate consumption of cheese and unsweetened Greek yogurt and low-carbohydrate milk. During Phase I, we will recommend limiting or avoiding other dairy, fruits, legumes, beans, and grains. Phase II (*Keep it Low*) will run from months 4 through 6. We will recommend that participants add 5 grams of carbohydrates per day and maintain this carbohydrate intake level for a week or two at a time, for a maximum total of <60 g net carbohydrates. As they add back carbohydrates, we will have them monitor their satisfaction with their diet and energy levels. We will recommend participants add back in foods like fruit (preferably berries), legumes, beans, dairy, and (limited) whole grains, while still aiming to keep total carbohydrate intake to <60 net grams.

Materials: We will develop a handbook that will be given to participants. This will contain diet guidelines, recipes, meal planners, shopping lists, and guides for counting carbohydrates and reading nutrition labels.

Meals: Participants will select their own menus and prepare or buy their meals according to the guidelines. We will provide participants with supplemental low-carbohydrate foods, such as nuts, olive oil, and other products, to help facilitate their compliance.

Counseling: Sessions and materials will focus on instructing participants to reduce digestible carbohydrate consumption by increasing consumption of protein and fat, with a particular emphasis on increased consumption of heart-healthy unsaturated fats. After randomization, participants will begin the 3-month *Go Low* phase consisting of individual counseling sessions each week for the first four weeks, followed by 4 group sessions held every other week with phone follow-ups occurring in between group sessions. During the *Keep it Low* phase, months 4 through 6, participants will attend 3 monthly group sessions and have 3 telephone follow-ups. There will be a total of 11 in-person sessions and 7 telephone follow-ups over the full study period. Sessions will be approximately one hour and phone calls will be no longer than thirty minutes. These sessions will consist of diet instruction and supportive counseling, addressing results from 24-hour dietary recalls, teaching self-monitoring and individual diet goal changes, and provide suggestions for replacing usual foods containing carbohydrates. Study materials will be modified from materials used in the successful MACRO study. The JFI will work closely with Drs. He and Bazzano to develop the intervention based on the MACRO study.
Carbohydrate goal: In the MACRO study, carried out among individuals from the same study region, a behavioral intervention aimed at reducing digestible carbohydrate intake to <40 g/day led to reductions in weight and improvements in cardiovascular risk factors among individuals with obesity.[30] Additionally, restriction of carbohydrate intake to <50g/day is sufficient to induce

ketosis.[105] Our rationale for having a multi-phased intervention is to allow participants to see how they feel on the more restrictive diet, and see how they respond to small increases in carbohydrate consumption. A similar technique was applied in the DIETFITS published study.[106] The higher target of 60 net grams may be more sustainable long-term and also allows for consumption of healthy higher-carbohydrate foods, such as berries, legumes, beans, and limited whole grains.

8.B. Usual Diet Group

We will provide participants randomized to the usual diet group written information with standard dietary advice. Participants randomized to the usual diet group will not receive ongoing dietary recommendations. Moreover, they will continue to receive clinical care recommendations from their physician(s). To encourage continued participation in the study and minimize the difference in participant contact with study staff, participants will be offered optional monthly educational sessions on topics unrelated to the study intervention, such as retirement planning or health insurance options.

In another effort to encourage continued participation, patients randomized to the usual diet group will be offered a copy of all available intervention materials, as well as an optional one-hour counseling session with the interventionist following their final study visit.

8.C. Physical Activity

At baseline, both groups will receive written information in study materials with standard physical activity recommendations.

8.D. Dietary Adherence

Strategies for measuring compliance: For the low-carbohydrate diet, adherence will be assessed through measurement of urinary ketones at 3-month visit and 6-month TV. Study staff will make all reasonable effort to maintain adherence of participants to the visit schedule and diet intervention.

Strategies for improving compliance:

- At the screening visit, we will ask participants who does most of the cooking in their households and how often they eat outside of the home. Results from the screening visit will help to select a group of cooperative study participants.
- Visits will be scheduled at the participants' convenience and to minimize waiting time and trips to the clinic; reminder calls will also be made prior to clinic visits.
- We will hire enthusiastic staff who have good interpersonal skills.
- Participants will receive $40 gift cards for use at food stores or retailers when they complete each study visit, for a maximum of $120 over the study period.
- Participants will receive $25 gift cards when they return the stool specimens, for a maximum of $50 over the study period.
- Participants will be offered small gifts (such as pens or magnets with study logo) to encourage compliance and adherence to the study protocol.
- Participants will be provided with recipes, shopping lists, and food samples for the intervention diet.

9. Safety of Dietary Intervention

On the low-carbohydrate diet, participants will be encouraged to primarily focus on replacing carbohydrates (particularly highly refined carbohydrates) with foods such as non-starchy vegetables, olive oil and other plant-based unsaturated oils, nuts and seeds, avocados, eggs, seafood, tofu, poultry, and lean meat. Consumption of more unsaturated fats, protein, and fiber

will be recommended. Participants will not be encouraged to increase consumption of saturated fats and will be advised to avoid consumption of trans fats.

In animal studies, a high-protein diet has decreased renal function[107] In the MACRO study, on average, participants tended to increase consumption of fats, rather than protein.[30] The safety and tolerability of low-carbohydrate diets have previously been assessed in many studies, including MACRO.[30] A number of studies, including some in individuals with T2D, have found no clinically significant increases in estimated glomerular filtration rates,[33,38–40] microalbuminuria,[108] albumin excretion rate,[33,38] urinary albumin,[38] serum creatinine,[38,40,108–113] albumin,[40] or uric acid levels.[109–112] A small study of a very low-carbohydrate diet found significant increase in uric acid excretion, but no significant change in 24-hour urine creatinine clearance.[112]

Study staff will carefully monitor participants' BP, and HbA1c more often than what would occur in routine healthcare visits. A Data Safety and Monitoring Board (DSMB) consisting of an independent group of experts in areas such as biostatistics, nutrition medicine, general internal medicine, and endocrinology has been formed. The board will regularly review trial progress, including unblinded interim results, and can recommend that the trial prematurely end if participants are at risk or if it becomes clear that the trial will not be able to yield statistically significant results.

10. Data Collection

10.A. Study visits

After the initial pre-screening interview, in which data on medications and medical conditions will be collected, there will be one screening visit, one randomization visit, a follow-up study visit at 3 months and a termination visit (TV) at 6 months. ***Table 3*** summarizes study procedures and data collected at each study visit.

Table 3. Study visit schedule and data collection

	**Pre-screen**	**SV**	**RZ**	**3 month**	**6 month TV**
Pre-screen eligibility	X				
Informed consent		X			
Compliance form		X			
Eligibility confirmation			X		
Randomization			X		
Demographics		X			
Medical history questionnaire			X	X	X
Medications		X	X	X	X
Lifestyle (alcohol, cigarette smoking)			X	X	X
Physical activity			X	X	X
24-hour diet recall*			X	X	X
3 BP measurements		X	X	X	X
Body weight			X	X	X
Height			X		
Waist circumference			X	X	X

Dipstick ketone test		X	X	X
Blood samples	X	X	X	X
Stool specimen		X		X
Side-effects assessment		X	X	X

*Two 24-hour dietary recalls will be collected; one will cover a weekend and the other a weekday.

10.A.1. Screening/baseline visits:

Participants will be pre-screened by phone or in-person by study staff to confirm eligibility for the Screening Visit. An approved questionnaire will be used to ensure the patient is eligible to attend the in-clinic screening visit, and the patient will be scheduled thereafter.

At the screening visit (SV), participants will provide informed consent for the screening and main study. Study staff will schedule the SV and randomization (RZ) visit at least 2 days apart. If a participant is eligible based on the screening visit, he or she should be scheduled for the RZ once a sufficient group of eligible individuals has been recruited. Ideally, participants should be scheduled for the RZ within two to three weeks of the screening visit.

At the screening visit, we will collect information on inclusion/exclusion criteria, medication use, and demographics. At this visit, we will also have participants complete a compliance form, to assess their ability to complete the study and attend all intervention and study visits. We will also measure blood pressure, and take blood samples. Women will be asked if they are postmenopausal or if they had surgical sterilization (such as a tubal ligation). If not, they will be tested for pregnancy through a spot urine test; pregnant women will be excluded from the study. Blood samples will be collected for measurement of HbA1c, and, if not available from medical records in the past 3 months, serum creatinine and hemoglobin. These blood samples will be used for excluding individuals with HbA1c <6.0 or >6.9, eGFR ≤45 mL/min/1.73 m^2, or hemoglobin <11 mg/dL.

Before the start of the intervention, participants will complete two 24-hour dietary recalls. At the RZ, participants will learn about the randomization process. After requirements for eligibility have been satisfied, participants will formally be enrolled into the study and randomized to either the active intervention or control group. We will collect fasting blood samples for later measurement of glucose, insulin and lipids and other potential future measurements. At this visit, we will measure blood pressure, body weight, height, and waist circumference. We will collect information on medical history, lifestyle risk factors, and physical activity. A spot urine sample will be collected to measure ketones and participants will be asked to collect a stool specimen for potential use in future microbiome studies.

10.A.2. Follow-up visits and final visit: A follow-up visit will occur at 3 months after randomization. A final visit will occur at 6-months post-randomization. These visits will aim to be scheduled as close as possible to the 3-, or 6-months after randomization, with a goal of being within two weeks of these follow-up periods. At each visit, study staff will conduct dietary recalls and anthropometric measures, take 3 BP measurements, collect a fasting blood sample to measure HbA1c. We will also collect samples for later measurement of glucose, insulin, lipid profile, and other potential future measurements. A spot urine sample will be collected to measure ketones. At the 6-month TV, participants will be asked to collect a stool specimen for potential use in future microbiome studies. At study termination, we will mail participants and/or their physicians a summary of trial results and a record of participant BP, anthropometric measures, and laboratory data from the trial period. Throughout the study period, we will

provide participants with their blood pressure and weight. We will request that, if possible, a participant who is prescribed a diabetes medication during the course of the study comes into the clinic for an additional separate supplemental visit for a fasting blood sample collection prior to starting the medication.

10.B Study measurements

Measurements obtained for this study will be based on those used in other clinical trials and epidemiologic studies. We are familiar with strengths and limitations of such measurements and the importance of staff training and certification and use of stringent quality control procedures.

10.B.1. Questionnaires: Study staff will administer a *medical history questionnaire* at the RZ, 3-month visit and 6-month TV to assess medical conditions that would necessitate changes in therapy or medical management beyond this study's scope. At the SV, *demographic factors* will be collected. At the SV, RZ, 3-month and 6-month TV, we will also administer *medication tracking questionnaires*. At RZ, 3-month, and 6-month TV, we will collect health behavior and physical activity data.

10.B.2. Dietary measurements: Two 24-hour dietary recalls will be obtained from participants at baseline. At or close to both the 3-month and 6-month TV, two more 24-hour dietary recalls will be collected. The second sets of two recalls will be collected as closely as possible to 3- or 6-month dates after randomization, with a goal of being within 2 weeks of the respective follow-up period. For each time point, one recall will ideally reflect week day consumption, while the other will ideally reflect weekend day consumption and the two recalls should be at least 2 days apart from each other. We will use computer software (Nutrition Data System for Research [NDS-R]) and food models used in previous and ongoing studies. Recalls will be conducted by a study staff member who has been certified in the use of NDS-R. Food composition tables of the NDS-R will be used to calculate dietary nutrient intakes. The baseline dietary assessment will be done to characterize individual dietary intake of carbohydrates and other macronutrients. During the intervention, repeated measurements will be used to monitor short-term changes in mean dietary nutrient intake between the intervention and usual diet groups.

10.B.3. Assessment and management of adverse effects: At RZ, 3-month visit, and 6-month TV, we will administer *symptom questionnaires*, which will consist of a checklist to identify participant complaints. Prior studies of low-carbohydrate dietary interventions suggest that participants may experience a range of adverse effects, including constipation, halitosis, fatigue, headache, thirst, polyuria, and nausea. Severe adverse events will be promptly reported to the DSMB. Management of adverse effects will be based on the protection and safety of the participant. If severe adverse effects are reported by a participant, carbohydrate intake will be examined and altered in order to mitigate the adverse effect. In any extreme cases, participants will be instructed to stop the intervention. As this is a behavioral intervention, adverse effects should be rare.

10.B.4. BP and anthropometric measures: BP will be measured 3 times at the screening visit and at each follow-up visit. To minimize random error, averages of the measurements at the screening and randomization visits will be taken. BP will be measured by a standard automated blood pressure measurement device (OMRON HEM-907 XL Professional Digital Blood Pressure Monitor) and performed by experienced staff, trained and certified in BP measurement. Prior to BP measurements, participants will be asked to rest quietly in a seated position with their feet supported for at least 5 minutes; staff members will use an appropriate cuff size over a bare arm. Trained and certified staff will measure weight using a calibrated balance beam scale at the screening visits, and follow-up visits. Weight and height will be measured at the RZ, and weight will also be measured at the subsequent visits.

10.B.5. Blood samples: For SV, participants will not need to fast. For RZ, 3-month visit, and 6-month TV, participants will be instructed to fast for 12 hours prior to blood sample collection. At

SV, blood samples will be collected to measure HbA1c, hemoglobin, and serum creatinine. At RZ, fasting blood samples will be collected and stored as serum/plasma, whole blood aliquots and buffy coats at -80°C for future analyses, including measurement of glucose, insulin and lipids, and potential future metabolomics or genetic analyses. At 3-month visit and 6-month TV, we will collect fasting blood samples to measure HbA1c and will also store additional serum/plasma, whole blood aliquots and buffy coats at -80°C for future analyses, including measurement of glucose, insulin and lipids, and potential future metabolomics or genetic analyses. Details of the blood sample collection are shown in ***Table 4*****.** HOMA-IR will be calculated from fasting glucose and insulin.

Table 4. Study visit schedule for specimen collection

	SV	RZ	3 month	6 month TV
HbA1c	X		X	X
Serum creatinine	X			
Hemoglobin	X			
Storage of aliquots and buffy coats (for measurement of glucose, insulin, lipids, other potential future measurements)		X	X	X
Urine sample for pregnancy	X			
Urine sample for ketones		X	X	X
Stool sample		X		X

10.B.6. Urine collection: Study staff will collect spot urine specimens at RZ, interim visit, and the final visit to measure ketones.

10.B.7. Stool Specimen Collection: Stool samples will be collected from all eligible and willing trial participants during the RZ and 6-month TV, following the protocol set forth by the Human Microbiome Project.[114,115] Participants will be given a stool collection kit, which includes the specimen collection container, a shipper for home collection, and a form to record the timing and pertinent information related to specimen collection. Participants will be instructed to return the kit to the clinic within two days of collection (in-person or via shipping), at which time stool samples are stored at -80°C until analyses. Kits and instructions for collection will be reviewed by study staff with the participant at the required visits.

11. Quality Assurance and Quality Control

Throughout the study, we will implement rigorous quality assurance (QA) and quality control (QC) measures. We describe some of these approaches here.

11.A. Manual of procedures

We will develop a manual of procedures (MOP) based on the MOP successfully used in the MACRO behavior change clinical trial. The MOP will provide instructions and describe procedures for staff training and certification, participant recruitment, intervention counseling,

study form completion and procedures, data entry, safety measures (e.g., methods for emergency participant/primary care physician contact).

11.B. Training and certification

Trial personnel will all be required to participate in a training session before the start of any trial procedures or measurements. Training will include particular attention to core trial elements, such as recruitment, retention, intervention, completeness and quality of data acquisition (e.g., blinding procedures for the data collectors), and data entry. Separately, intervention staff will meet and receive detailed instruction on trial approaches to group and individual behavior change counseling and procedures that ensure data collectors are blinded to treatment assignment. Throughout the trial, periodic re-training/certification sessions will be conducted to ensure all trial-related procedures are being applied in a standard manner. Investigators will also monitor all aspects of study performance to ensure study goal achievement, timeliness and completeness of study visits, completeness and quality of data collection, intervention adherence, and adequacy of the procedures separating data collection from intervention. Study staff and investigators will pay particularly careful attention to the quality of all laboratory measurements, particularly key exposure, outcome, and intervention monitoring variables.

12. Sample Size and Statistical Power

The primary outcome will be the difference in the change of HbA1c between the intervention and usual diet groups from baseline to 6 months of follow-up. Based on estimated SD for change in HbA1c from a medical nutrition therapy intervention among individuals with IFG or HbA1c 5.7–6.4%[116] and of weight loss programs among individuals with HbA1c 5.7–6.4% or prior gestational diabetes,[117] assuming an SD of 0.31 for change in HbA1c, we would need 120 participants to have 85% power to detect an approximate difference in change of HbA1c of 0.17% between the intervention and usual diet groups.

In a follow-up analysis of the DPP, a 0.17% decrement in HbA1c at 6 months was associated with an 18% lower risk of incident T2D, adjusting for treatment arm, age, sex, race/ethnicity, household income, and baseline HbA1c.[71] In a pooled analysis from the Atherosclerosis Risk in Communities and Framingham Heart Studies, a 0.17% higher HbA1c was associated with an 18% higher risk of T2D over 20 years, a 35% higher risk over 8 years, and a 15% higher risk after 8 years, after adjusting for age, sex, fasting glucose, triglycerides, HDL, family history of T2D, SBP, and BMI.[69] Additionally, in EPIC-Norfork, a difference of HbA1c of 0.17% was associated with approximately a 6% higher risk of CHD among those free of CHD, stroke, diabetes, and with HbA1c <7.0%.[72]

In the DPP study, HbA1c was 0.18% lower among those in the lifestyle intervention arm than in the control arm at 1-year of follow-up; HbA1c at study commencement was 5.9% and levels increased in the control arm and decreased in the behavioral intervention arm.[22] In a small behavioral intervention study (n=34) of a very low-carbohydrate ketogenic diet compared with a moderate carbohydrate, calorie-restricted, low-fat diet among individuals

Table 5. Minimum detectable differences

	SD	Detectable Difference
HbA1c, %	0.31	0.17
Fasting glucose, mg/dL	12.5	6.9
Fasting insulin, mU/mL	7.0	3.9
HOMA-IR	2.0	1.1
SBP (mmHg)	4.2	2.3
DBP (mmHg)	3.0	1.7
Total/HDL ratio	1.2	0.66
Body weight (kg)	6.6	3.7
Waist circumference (cm)	8.0	4.4
Estimated CVD risk (%)*	2.7	1.6

*10-y CVD risk, among estimated 85% or 102 participants free of CVD

with type 2 diabetes or prediabetes (HbA1c >6.0%) and elevated body weight (BMI >25), mean HbA1c decreased from 6.6% to 6.1% in the very low-carbohydrate group and from 6.9% to 6.7% in the moderate carbohydrate group over a 12-month period; additionally, the very low-carbohydrate group had greater reductions in glucose-lowering medications.[37]

Detectable differences are listed for HbA1c and secondary outcomes in ***Table 5***, based on data from prior studies.[30,53,56,118] These were calculated for unpaired t-tests, power of 85%, two-sided alpha of 0.05, and assuming 80% follow-up of 150 participants, which would yield an estimated 120 participants.

13. Data Management and Analysis

13.A. Data management

Personnel will double-enter all study data. Prior to entering data, the study coordinator will conduct quality control on all study forms, study measures, and laboratory data. Data will be cleaned and logically checked.

13.B. Data analysis plan

The data will be analyzed using *intention to treat*; this involves comparing the primary and secondary outcomes between participants based on randomization assignment.

13.B.1. Exploratory data analysis: We will express group data for variables with a normal distribution as mean ± standard deviation and for variables not normally distributed as median and interquartile ranges (or will log-transform the variables to obtain a normal distribution). We will compare means and percentages of baseline variables across the intervention and usual diet group to assess similarity of these groups at randomization.

13.B.2. Primary, secondary, and exploratory outcomes: We will test the primary study hypothesis that there is a greater reduction in HbA1c from baseline to 6 months in the intervention group than in the usual diet group. To do this, we will use a mixed-effects linear regression model, to test the null hypothesis that there is no difference in change between baseline and 6-month HbA1c for the intervention group compared with the usual diet group (Specific Aim 1). To do this, we will use the following model:

$$Y_{i,j}|T = b_0 + b_1G_i + b_2T + b_3G_iT_6 \quad + \ _{0i,j} + \ _{i,j,}$$

where $Y_{i,j,k}$ is HbA1c for the jth participant (P) nested in the ith intervention group (G) at time T_k (where T_k=0 for baseline, 1 for 3 months, and 2 for 6 months). This model includes a random intercept ($a_{0i,j}$) and an error term $e_{i,j,k}$. The interaction term ($b_3G_iT_{6\text{-months}}$) allows for an interaction between group and $T_{6\text{-months}}$, which is equal to 0 for baseline and 3-months and 1 for 6-months. Rejection of the null hypothesis ($b_1+2*b_3+b_4=0$) indicates there is a significant difference in change of HbA1c from baseline to 6 months between the intervention and control groups. In this model, participants are assumed to be random effects and intervention group, time, and the interaction terms are fixed effects.

We will use a similar approach to test the difference in the change between baseline and 6-month values of secondary outcomes (Specific Aim 2) and exploratory outcomes. We will also explore the use of mixed effects models to assess overall net change in HbA1c (by treating time as a linear variable) and other outcomes over the full study period. For each outcome, we will test modeling assumptions and will consider transformation of outcomes, if needed, to meet modeling assumptions. As the mixed-effects model assumes missing at random, we will also perform sensitivity analyses using Markov-chain Monte Carlo techniques to impute missing values of the outcomes, using covariates for this imputation and will compare results from this analysis with our primary findings.

15. Anticipated Challenges

Recruitment: Many clinical trials have challenges recruiting sufficient numbers of volunteers who meet the trial inclusion and exclusion criteria and demonstrate a high level of adherence to trial protocol requirements. Fortunately, the investigators are very experienced with clinical trial recruitment, and have several contingency options that can be used to meet unanticipated shortfall in recruitment.

Intervention: The investigators are aware of and understand challenges related to achieving and maintaining compliance with a behavioral intervention. The investigators have much experience and success in behavioral interventions, with recent success of a behavioral intervention aimed at reducing carbohydrate intake among participants from the New Orleans area. The JFI will collaborate with Drs. He and Bazzano to develop the intervention program based on the MACRO study and will assess potential barriers to implementation and intervention adherence (such as financial concerns and range of dietary patterns of study participants) during the planning and pilot phase of the study.

Adherence: Though adherence and follow-up are also main challenges related to clinical trials, the investigators have an outstanding track record for studies that have recruited participants for both clinical and epidemiology studies in the Greater New Orleans area. To encourage adherence, small gifts (such as pens or magnets with study logo) will be offered to all study participants.

16. Protection of Human Subjects

This Human Subjects Research meets the definition of a clinical trial. All investigators will be required to complete the CITI (Collaborative Institutional Training Initiative) program in the ethical use of human participants in research.

16.A. Protection of human subjects from research risks

16.A.1. Risks to the subjects

Human subject involvement and characteristics: We expect to enroll 150 participants, with an equal male to female ratio. We expect to enroll approximately 30 participants who are African-Americans, and another 10 with mixed racial backgrounds, and a few Asian participants given the demographics of the greater New Orleans area. Overall, given the demographics of greater New Orleans, we estimate approximately 10% participants of Hispanic or Latino ethnicity, 50% of them with African-American racial background.

Inclusion criteria

- Men or women ages 40 to 70 years: aim to recruit 50% women
- Any race or ethnicity: aim to recruit 40% ethnic minorities (i.e., African American and Hispanic), close to population in the greater New Orleans area[100]
- HbA1c 6.0–6.9%
- Willing and able to provide informed consent

Exclusion criteria

- Diagnosed type 1 diabetes mellitus based on patient self-report
- Use of agents affecting glycemic control (medications for diabetes, oral glucocorticoids) within the past three months prior to enrollment based on patient self-report
- Medical condition in which low-carbohydrate diet may not be advised

  - Estimated glomerular filtration rate (eGFR) ≤45 mL/min/1.73 m^2, which is close to the 5th percentile of eGFR among non-diseased individuals of 70 years of age[101]
  - Self-report of liver disease due to hepatitis or alcohol; osteoporosis; untreated thyroid disease; gout; or cancer (other than non-melanoma skin cancer) requiring treatment in the past year, unless prognosis is excellent
- Factors that may affect HbA1c:
  - Hemoglobin <11 mg/dL (cutpoint for moderate-to-severe anemia, which could lead to falsely elevated or lowered HbA1c)[102]
  - Recent blood donation or blood transfusion (self-report, past 4 months)
  - Human Immunodeficiency Virus (self-report)[103]
- Allergies to nuts
- For women, current pregnancy, breastfeeding, or plans to become pregnant during the study
- Consumption of ≥21 alcoholic drinks per week or consumption of ≥6 drinks per occasion
- Current or planned residence making it difficult to meet trial requirements (due to distance from study site and/or challenges regularly traveling to site)
- Current participation in another lifestyle intervention trial or a pharmaceutical trial
- Participation of another household member in the study; employees or persons living with employees of the study
- Other concerns regarding ability to meet trial requirements, at the discretion of the principal investigator or study coordinator

Sources of materials: Medical information data will be collected from the participant and, after written authorization for the disclosure of protected health information, from participants' medical records. Blood and urine samples will be collected to measure HbA1c, glucose, insulin, lipids, creatinine, ketones, and test for pregnancy. Blood pressure, height, weight, waist circumference and other physical measurements will be obtained.

Potential Risks: Risks associated with venipuncture, the diet intervention, and collection of confidential medical information are the main risks associated with this study. There is a small risk of bruising and a rare chance of local infection associated with standard venipuncture to collect blood samples. The dietary intervention has minimal risks. Reduced carbohydrate consumption may lead to adverse effects, including constipation, halitosis, fatigue, headache, thirst, polyuria, and nausea. At RZ, 3- , and 6-month visits, we will administer *symptom questionnaires*, which will consist of a checklist to identify participant complaints. Adverse effects of the low-carbohydrate diet will be assessed at counseling sessions. Severe adverse events will be promptly reported to the DSMB. Management of adverse effects will be based on the protection and safety of the participant. If severe adverse effects are reported by a participant, carbohydrate intake will be examined and altered in order to mitigate the adverse effect. In any extreme cases, participants will be instructed to stop the intervention. Furthermore, there is a potential risk of unauthorized disclosure of medical information. This risk will be minimized by using a strict protocol for data processing and by the appropriate training of all study personnel. Protection against risks is described in more detail below.

16.A.2. Adequacy of protection against risks

Recruitment and Informed Consent: The proposed study will be conducted following strict guidelines for the protection of the rights of human volunteers. The informed consent document

will be signed by all participants at the screening/baseline visit. The informed consent form will be developed in the first year of the study and approved by the IRB. The consent form will clearly state the purpose, eligibility criteria, and protocol of the study, the potential benefits and risks of participation, and the subject's right to refuse to participate or withdraw. Prior to signing the informed consent, research staff will review the details of the consent form orally with the participant, and answer any questions that the participant has concerning participation in the study. In the case of potential participants with low or no literacy, the informed consent form will be read to the participant in its entirety and any questions will be answered prior to the participant signing or marking the form. Specifically, the following must be accomplished during the informed consent process:

- The participant must be informed that participation in the study is **voluntary** and that refusal to participate will involve no penalty or loss of benefits.
- The participant must be informed of the **purpose** of the study and that it involves **research**.
- The participant must be informed of any **alternative procedures,** if applicable.
- The participant must be informed of any reasonably foreseeable **risks**.
- The participant must be informed of any **benefits** from the research.
- An outline of safeguards to protect participant **confidentiality** will be included, as well as an indication of the participant’s right to withdraw without penalty. This should be balanced with a discussion of the effect that withdrawals will have on the study, and the responsibility a participant has, within limits, to continue in the study if he or she decides to enroll.
- The participant must be informed of his or her right to have **questions answered** at any time and **whom to contact** for answers or in the event of research-related injury.
- The participant must be informed as to whether or not **compensation** is offered for participation in the study and/or in the event of a medical injury.
- The participant must be informed that he/she will be notified of any significant **changes** in the protocol that might affect their willingness to continue in the study.

Written authorization for the disclosure of protected health information will be obtained during the consent process on a separate form (HIPPA authorization), if applicable.

Protection against Risk: The protocol and informed consent will be approved by the Institutional Review Boards at Tulane University Health Sciences Center and any other participating institution for recruitment. The study participants will be given contact information for the Study Coordinator and Study PI to answer questions or to report complaints. Patients can withdraw from the study at any time without any effect on their medical care. Standard techniques will be used by trained, certified phlebotomists or study nurses for blood sample collection at the study clinics where emergency medical equipment is available. Confidentiality of all data collected during the study will be maintained through the use of unique, encrypted study identification (ID numbers, rather than patient names, in the study database). These encrypted ID numbers will not be mathematical derivation of the subject’s medical record number or any other patient identifier. No information will be disclosed in an individually identifiable form in any type of presentation or publication. There will be password-required access to the computerized file linking the study numbers to patient identifiers at the study clinics. Password-access will be restricted to pertinent research staff only. All study data will be kept in locked file cabinets in locked rooms, accessible only by study staff with permission. All data transmissions will use HIPPA-compliant password protected system and will be secured through a virtual private network.

16.A.3. Potential benefits of the proposed research to human subjects and others

Study participants may have improved glycemic outcomes, blood pressure, lipid levels, may lose weight and reduce waist circumference, and may have improvement in other risk factors for CVD and development and progression of T2D. Participants will receive multiple blood pressure, weight, waist circumference, and blood tests. If vital signs or lab values are clinically abnormal, participants will be referred to their primary care physician, or if they do not have a primary care physician, one will be assigned through the Tulane University Community Health Center, which is a part of the core clinical facilities. At the end of the study, we will provide the participants' primary care physician with a copy of all the blood pressure, weight, and laboratory results.

16.A.4. Importance of knowledge to be gained

Information from this study may contribute to reducing the onset of T2D and the burden of T2D and cardiovascular disease in Louisiana and the general US population. The study has potential for public health impact; results from this study may provide evidence in support of promoting alternative dietary styles (than the most commonly used low-fat diet) among individuals with high T2D risk or with T2D.

16.A.5. Remuneration

For screening visit 2, the two follow-up visits, and the final visits, participants will receive $20 gift cards for use at food stores or retailers (after completing each study visit), for a maximum of $60 over the study period. Participants who return a stool sample will receive $25 gift cards for each stool sample that they return, for a total of $50 for two stool samples. Participants will also receive free parking for each intervention and study visit.

16.B. Data safety and monitoring plan

A DSMB, which consists of 3 experts who are not otherwise affiliated with the COBRE program, has been formed. Study investigators for this study will prepare accurate and timely data tables and reports for the DSMB meetings. The members of the DSMB will serve in an individual capacity and provide their expertise and recommendations. The primary responsibilities of the DSMB are: 1) periodically review and evaluate the accumulated study data for participant safety, study conduct and progress, and, when appropriate, efficacy, and 2) make recommendations to the NIH concerning the continuation, modification, or termination of the trial. The DSMB considers study-specific data, as well as relevant background knowledge, about the disease, test agent, and patient population under study.

The DSMB is responsible for defining its deliberative processes, including event triggers that would call for an unscheduled review, stopping guidelines, unmasking (unblinding) and voting procedures prior to initiating any data review. The DSMB is also responsible for maintaining the confidentiality of its internal discussions and activities, as well as the contents of reports provided to it.

The DSMB will review each protocol for any major concerns prior to implementation. During the trial, the DSMB should review cumulative study data to evaluate safety, study conduct, and scientific validity and integrity of the trial. As part of this responsibility, DSMB members must be satisfied that the timeliness, completeness, and accuracy of the data submitted to them for review are sufficient for evaluation of the safety and welfare of study participants. The DSMB should also assess the performance of overall study operations and any other relevant issues, as necessary.

Items reviewed by the DSMB include:

- Interim/cumulative data for evidence of study-related adverse events;
- Interim/cumulative data for evidence of efficacy according to pre-established statistical guidelines, if appropriate;
- Data quality, completeness, and timeliness;
- Adequacy of compliance with goals for recruitment and retention, including those related to the participation of women and minorities;
- Adherence to the protocol;
- Factors that might affect the study outcome or compromise the confidentiality of the trial data (such as protocol violations, unmasking, etc.); and,
- Factors external to the study, such as scientific or therapeutic developments, that may impact participant safety or the ethics of the study.

The DSMB will conclude each review with their recommendations to the COBRE program and NIH/NIGMS as to whether the study should continue without change or be modified or terminated. Recommendations regarding modification of the design and conduct of the study could include:

- Modifications of the study protocol based upon review of the safety data;
- Suspension or early termination of the study or of one or more study arms because of serious concerns about subjects' safety, inadequate performance or rate of enrollment;
- Suspension or early termination of the study or of one or more study arms because study objectives have not been obtained according to pre-established statistical guidelines; and,
- Corrective actions regarding a study when the performance appears unsatisfactory or suspicious**.**

Confidentiality must always be maintained during all phases of DSMB review and deliberation. Usually, only voting members of the DSMB should have access to interim analyses of outcome data by treatment group. Exceptions may be made when the DSMB deems it appropriate. The reason and to whom the exceptions for access to interim analyses is granted will be documented in the Closed Session Report. DSMB members must maintain strict confidentiality concerning all privileged trial results ever provided to them. The DSMB should review data only by masked study group (such as X vs. Y rather than experimental vs. control) unless or until the DSMB determines that the identities of the groups are necessary for their decision-making. Whenever masked data are presented to the DSMB, the key to the group coding must be available for immediate unmasking.

The DSMB will meet twice per year. The junior faculty investigator will attend these meetings and will be responsible for preparing and presenting up-to-date statistical reports on the progress of their trials. These reports will include data on recruitment, randomization, adherence, as well as statistical tests and special analyses requested by the DSMB. The Administrative Core will be responsible for organizing the annual in-person meeting and semiannual conference calls. They will put together data and materials submitted by junior faculty investigators and deliver them to DSMB members. The COBRE investigators will meet with the DSMB in the Open Session and not be present for the Closed Session.

Bibliography and References Cited

1. The Emerging Risk Factors Collaboration. Diabetes mellitus, fasting blood glucose concentration, and risk of vascular disease: a collaborative meta-analysis of 102 prospective studies. *Lancet*. 2010;375(9733):2215-2222. doi:10.1016/S0140-6736(10)60484-9.
2. Flaxman SR, Bourne RRA, Resnikoff S, et al. Global causes of blindness and distance vision impairment 1990-2020: a systematic review and meta-analysis. *Lancet Glob Heal*. October 2017. doi:10.1016/S2214-109X(17)30393-5.
3. United States Renal Data System. *2016 USRDS Annual Data Report: Epidemiology of Kidney Disease in the United States*. Bethesda, MD: National Institutes of Health, National Institute of Diabetes and Digestive and Kidney Diseases; 2016. https://www.usrds.org/2016/view/Default.aspx.
4. Narres M, Kvitkina T, Claessen H, et al. Incidence of lower extremity amputations in the diabetic compared with the non-diabetic population: A systematic review. *PLoS One*. 2017;12(8):e0182081. doi:10.1371/journal.pone.0182081.
5. Centers for Disease Control and Prevention. *National Diabetes Statistics Report, 2017*. Atlanta, GA: Centers for Disease Control and Prevention, U.S. Dept of Health and Human Services; 2017.
6. Centers for Disease Control and Prevention. *Long-Term Trends in Diabetes*. Atlanta, GA: Centers for Disease Control and Prevention, U.S. Dept of Health and Human Services; 2017.
7. NCD Risk Factor Collaboration (NCD-RisC). Worldwide trends in diabetes since 1980: A pooled analysis of 751 population-based studies with 4.4 million participants. *Lancet*. 2016;387(10027):1513-1530. doi:10.1016/S0140-6736(16)00618-8.
8. American Diabetes Association. Economic costs of diabetes in the U.S. in 2012. *Diabetes Care*. 2013;36(4):1033-1046. doi:10.2337/dc12-2625.
9. Zhuo X, Zhang P, Barker L, Albright A, Thompson TJ, Gregg E. The lifetime cost of diabetes and its implications for diabetes prevention. *Diabetes Care*. 2014;37(9):2557-2564. doi:10.2337/dc13-2484.
10. WHO, International Diabetes Foundation. *Definition and Diagnosis of Diabetes Mellitus and Intermediate Hyperglycaemia: Report of a WHO/IDF Consultation*. Geneva: Geneva: World Health Organization; 2006.
11. The International Expert Committee. International Expert Committee Report on the Role of the A1C Assay in the Diagnosis of Diabetes. *Diabetes Care*. 2009;32(7):1327-1334. doi:10.2337/dc09-9033.
12. American Diabetes Association. Standards of Medical Care in Diabetes - 2017. *Diabetes Care*. 2017;40(Supplement 1):S33-S43. doi:10.2337/dc16-S003.
13. Gerstein HC, Santaguida P, Raina P, et al. Annual incidence and relative risk of diabetes in people with various categories of dysglycemia: A systematic overview and meta-analysis of prospective studies. *Diabetes Res Clin Pract*. 2007;78(3):305-312. doi:10.1016/j.diabres.2007.05.004.
14. Huang Y, Cai X, Mai W, Li M, Hu Y. Association between prediabetes and risk of cardiovascular disease and all cause mortality: systematic review and meta-analysis. *BMJ*. 2016;355:i5953. doi:10.1136/bmj.i5953.
15. Tabák AG, Herder C, Rathmann W, Brunner EJ, Kivimäki M. Prediabetes: A high-risk state for diabetes development. *Lancet*. 2012;379(9833):2279-2290. doi:10.1016/S0140-

6736(12)60283-9.
16. Diabetes Prevention Program Research Group. 10-year follow-up of diabetes incidence and weight loss in the Diabetes Prevention Program Outcomes Study. *Lancet*. 2009;374(9702):1677-1686. doi:10.1016/S0140-6736(09)61457-4.
17. The DREAM (Diabetes REduction Assessment with ramipril and rosiglitazone Medication) Trial Investigators. Effect of rosiglitazone on the frequency of diabetes in patients with impaired glucose tolerance or impaired fasting glucose: a randomised controlled trial. *Lancet*. 2006;368(9541):1096-1105. doi:10.1016/S0140-6736(06)69420-8.
18. Perreault L, Pan Q, Mather KJ, Watson KE, Hamman RF, Kahn SE. Effect of regression from prediabetes to normal glucose regulation on long-term reduction in diabetes risk: Results from the Diabetes Prevention Program Outcomes Study. *Lancet*. 2012;379(9833):2243-2251. doi:10.1016/S0140-6736(12)60525-X.
19. Perreault L, Temprosa M, Mather KJ, et al. Regression from prediabetes to normal glucose regulation is associated with reduction in cardiovascular risk: Results from the diabetes prevention program outcomes study. *Diabetes Care*. 2014;37(9):2622-2631. doi:10.2337/dc14-0656.
20. Popkin BM. Nutrition Transition and the Global Diabetes Epidemic. *Curr Diab Rep*. 2015;15(9). doi:10.1007/s11892-015-0631-4.
21. Vazquez G, Duval S, Jacobs DRJ, Silventoinen K. Comparison of body mass index, waist circumference, and waist/hip ratio in predicting incident diabetes: a meta-analysis. *Epidemiol Rev*. 2007;29:115-128. doi:10.1093/epirev/mxm008.
22. Knowler WC, Barrett-Connor E, Fowler SE, et al. Reduction in the incidence of type 2 diabetes with lifestyle intervention or metformin. *N Engl J Med*. 2002;346(6):393-403. doi:10.1056/NEJMoa012512.
23. Pan XR, Li GW, Hu YH, et al. Effects of diet and exercise in preventing NIDDM in people with impaired glucose tolerance: The Da Qing IGT and diabetes study. *Diabetes Care*. 1997;20(4):537-544. doi:10.2337/diacare.20.4.537.
24. Tuomilehto J, Indstrom J, Eriksson J, Valle T, Hamalainine E, Uusitupa M. Prevention of Type 2 Diabetes Mellitus By Changes in Lifestyle Among Subjects With Impaired Glucose Tolerance. *N Engl J Med*. 2001;344(18):1343-1350. doi:10.1056/NEJM200105033441801.
25. Ramachandran A, Snehalatha C, Mary S, Mukesh B, Bhaskar AD, Vijay V. The Indian Diabetes Prevention Programme shows that lifestyle modification and metformin prevent type 2 diabetes in Asian Indian subjects with impaired glucose tolerance (IDPP-1). *Diabetologia*. 2006;49(2):289-297. doi:10.1007/s00125-005-0097-z.
26. The Diabetes Preventio Program Research Group. The Diabetes Prevention Program. Design and methods for a clinical trial in the prevention of type 2 diabetes. *Diabetes Care*. 1999;22(4):623-634. doi:10.2337/diacare.22.4.623 10.2337/diabetes.48.4.699.
27. Evert AB, Boucher JL, Cypress M, et al. Nutrition therapy recommendations for the management of adults with diabetes. *Diabetes Care*. 2013;36(11):3821-3842. doi:10.2337/dc13-2042.
28. Hu T, Mills KT, Yao L, et al. Effects of low-carbohydrate diets versus low-fat diets on metabolic risk factors: A meta-analysis of randomized controlled clinical trials. *Am J Epidemiol*. 2012;176(SUPPL. 7). doi:10.1093/aje/kws264.
29. Sackner-Bernstein J, Kanter D, Kaul S. Dietary Intervention for Overweight and Obese Adults: Comparison of Low-Carbohydrate and Low-Fat Diets. A Meta-Analysis. *PLoS*

One. 2015;10(10):e0139817. doi:10.1371/journal.pone.0139817.

30. Bazzano LA, Hu T, Reynolds K, et al. Effects of low-carbohydrate and low-fat diets: A randomized trial. *Ann Intern Med*. 2014;161(5):309-318. doi:10.7326/M14-0180.
31. Sawyer L, Gale EAM. Diet, delusion and diabetes. *Diabetologia*. 2009;52(1):1-7. doi:10.1007/s00125-008-1203-9.
32. Snorgaard O, Poulsen GM, Andersen HK, Astrup A. Systematic review and meta-analysis of dietary carbohydrate restriction in patients with type 2 diabetes. *BMJ Open Diabetes Res Care*. 2017;5(1):e000354. doi:10.1136/bmjdrc-2016-000354.
33. Larsen RN, Mann NJ, Maclean E, Shaw JE. The effect of high-protein, low-carbohydrate diets in the treatment of type 2 diabetes: A 12 month randomised controlled trial. *Diabetologia*. 2011;54(4):731-740. doi:10.1007/s00125-010-2027-y.
34. Saslow LR, Kim S, Daubenmier JJ, et al. A randomized pilot trial of a moderate carbohydrate diet compared to a very low carbohydrate diet in overweight or obese individuals with type 2 diabetes mellitus or prediabetes. *PLoS One*. 2014;9(4). doi:10.1371/journal.pone.0091027.
35. Tay J, Luscombe-Marsh ND, Thompson CH, et al. A very low-carbohydrate, low-saturated fat diet for type 2 diabetes management: A randomized trial. *Diabetes Care*. 2014;37(11):2909-2918. doi:10.2337/dc14-0845.
36. Davis NJ, Tomuta N, Schechter C, et al. Comparative Study of the Effects of a 1-Year Dietary Intervention of a Low- Carbohydrate Diet Versus a Low-Fat Diet on Weight and Glycemic Control in Type 2. *Diabetes Care*. 2009;32(7):1147-1152. doi:10.2337/dc08-2108.Clinical.
37. Saslow LR, Daubenmier JJ, Moskowitz JT, et al. Twelve-month outcomes of a randomized trial of a moderate-carbohydrate versus very low-carbohydrate diet in overweight adults with type 2 diabetes mellitus or prediabetes. *Nutr Diabetes*. 2017;7(12):304. doi:10.1038/s41387-017-0006-9.
38. Tay J, Thompson CH, Luscombe-Marsh ND, et al. Effects of an energy-restricted low-carbohydrate, high unsaturated fat/low saturated fat diet versus a high carbohydrate, low fat diet in type 2 diabetes: a 2 year randomized clinical trial. *Diabetes, Obes Metab*. 2017:n/a-n/a. doi:10.1111/dom.13164.
39. Yamada Y, Uchida J, Izumi H, et al. A non-calorie-restricted low-carbohydrate diet is effective as an alternative therapy for patients with type 2 diabetes. *Intern Med*. 2014;53(1):13-19. doi:http://dx.doi.org/10.2169/internalmedicine.53.0861.
40. Mayer SB, Jeffreys AS, Olsen MK, Mcduffie JR, Feinglos MN, Yancy WS. Two diets with different haemoglobin A1c and antiglycaemic medication effects despite similar weight loss in type 2 diabetes. *Diabetes, Obes Metab*. 2014;16(1):90-93. doi:10.1111/dom.12191.
41. Maekawa S, Kawahara T, Nomura R, et al. Retrospective study on the efficacy of a low-carbohydrate diet for impaired glucose tolerance. *Diabetes Metab Syndr Obes*. 2014;7:195-201. doi:10.2147/DMSO.S62681.
42. Davis C, Bryan J, Hodgson J, Murphy K. Definition of the mediterranean diet: A literature review. *Nutrients*. 2015;7(11):9139-9153. doi:10.3390/nu7115459.
43. Sofi F, Macchi C, Abbate R, Gensini GF, Casini A. Mediterranean diet and health status: an updated meta-analysis and a proposal for a literature-based adherence score. *Public Health Nutr*. 2014;17(12):2769-2782. doi:10.1017/S1368980013003169.
44. Jannasch F, Kröger J, Schulze MB. Dietary Patterns and Type 2 Diabetes: A Systematic Literature Review and Meta-Analysis of Prospective Studies. *J Nutr*. 2017;147(6):1174-

1182. doi:10.3945/jn.116.242552.

45. Schwingshackl L, Missbach B, König J, Hoffmann G. Adherence to a Mediterranean diet and risk of diabetes: a systematic review and meta-analysis. *Public Health Nutr*. 2014;18(7):1292–1299. doi:10.1017/S1368980014001542.

46. de Lorgeril M, Salen P, Martin J-L, Monjaud I, Delaye J, Mamelle N. Mediterranean Diet, Traditional Risk Factors, and the Rate of Cardiovascular Complications After Myocardial Infarction : Final Report of the Lyon Diet Heart Study. *Circulation*. 1999;99(6):779-785. doi:10.1161/01.CIR.99.6.779.

47. Estruch R, Ros E, Salas-Salvadó J, et al. Primary Prevention of Cardiovascular Disease with a Mediterranean Diet. *N Engl J Med*. 2013;368(14):1279-1290. doi:10.1056/NEJMoa1200303.

48. Salas-Salvadó J, Bulló M, Estruch R, et al. Prevention of diabetes with mediterranean diets: A subgroup analysis of a randomized trial. *Ann Intern Med*. 2014;160(1):1-10. doi:10.7326/M13-1725.

49. Esposito K, Marfella R, Ciotola M, et al. Effect of a Mediterranean-Style Diet on Endothelial Dysfunction and Markers of Vascular Inflammation in the Metabolic Syndrome. *JAMA*. 2004;292(12):1440. doi:10.1001/jama.292.12.1440.

50. Babio N, Toledo E, Estruch R, et al. Mediterranean diets and metabolic syndrome status in the PREDIMED randomized trial. *CMAJ*. 2014;186(17):E649-E657. doi:10.1503/cmaj.140764.

51. Esposito K, Maiorino MI, Bellastella G, Chiodini P, Panagiotakos D, Giugliano D. A journey into a Mediterranean diet and type 2 diabetes: a systematic review with meta-analyses. *BMJ Open*. 2015;5(8):e008222. doi:10.1136/bmjopen-2015-008222.

52. Micha R, Michas G, Mozaffarian D. Unprocessed red and processed meats and risk of coronary artery disease and type 2 diabetes--an updated review of the evidence. *Curr Atheroscler Rep*. 2012;14(6):515-524. doi:10.1007/s11883-012-0282-8.

53. Esposito K, Maiorino MI, Ciotola M, et al. Effects of a Mediterranean-style diet on the need for antihyperglycemic drug therapy in patients with newly diagnosed type 2 diabetes: A randomized trial. *Ann Intern Med*. 2009;151(5):306-314. doi:10.1097/OGX.0b013e3181e5a159.

54. Esposito K, Maiorino MI, Petrizzo M, Bellastella G, Giugliano D. The effects of a Mediterranean diet on the need for diabetes drugs and remission of newly diagnosed type 2 diabetes: Follow-up of a randomized trial. *Diabetes Care*. 2014;37(7):1824-1830. doi:10.2337/dc13-2899.

55. Elhayany A, Lustman A, Abel R, Attal-Singer J, Vinker S. A low carbohydrate Mediterranean diet improves cardiovascular risk factors and diabetes control among overweight patients with type 2 diabetes mellitus: A 1-year prospective randomized intervention study. *Diabetes, Obes Metab*. 2010;12(3):204-209. doi:10.1111/j.1463-1326.2009.01151.x.

56. Shai I, Schwarzfuchs D, Henkin Y, et al. Weight Loss with a Low-Carbohydrate, Mediterranean, or Low-Fat Diet. *N Engl J Med*. 2008;359(3):229-241. doi:10.1056/NEJMoa0708681.

57. Schwarzfuchs D, Golan R, Shai I. Four-Year Follow-up after Two-Year Dietary Interventions. *N Engl J Med*. 2012;367(14):1373-1374. doi:10.1056/NEJMc1204792.

58. Pan A, Sun Q, Bernstein AM, et al. Red meat consumption and risk of type 2 diabetes: 3 Cohorts of US adults and an updated meta-analysis. *Am J Clin Nutr*. 2011;94(4):1088-1096. doi:10.3945/ajcn.111.018978.

59. Hu EA, Pan A, Malik V, Sun Q. White rice consumption and risk of type 2 diabetes: meta-analysis and systematic review. *BMJ*. 2012;344:e1454. doi:10.1136/bmj.e1454.

60. Wang M, Yu M, Fang L, Hu R-Y. Association between sugar-sweetened beverages and type 2 diabetes: A meta-analysis. *J Diabetes Investig*. 2015;6(3):360-366. doi:10.1111/jdi.12309.

61. Imamura F, O'Connor L, Ye Z, et al. Consumption of sugar sweetened beverages, artificially sweetened beverages, and fruit juice and incidence of type 2 diabetes: systematic review, meta-analysis, and estimation of population attributable fraction. *BMJ*. 2015:h3576. doi:10.1136/bmj.h3576.

62. Carter P, Gray LJ, Troughton J, Khunti K, Davies MJ. Fruit and vegetable intake and incidence of type 2 diabetes mellitus: systematic review and meta-analysis. *BMJ*. 2010;341:c4229. doi:10.1136/bmj.c4229.

63. Cooper AJ, Forouhi NG, Ye Z, et al. Fruit and vegetable intake and type 2 diabetes: EPIC-InterAct prospective study and meta-analysis. *Eur J Clin Nutr*. 2012;66(10):1082-1092. doi:10.1038/ejcn.2012.85.

64. Chen M, Sun Q, Giovannucci E, et al. Dairy consumption and risk of type 2 diabetes: 3 cohorts of US adults and an updated meta-analysis. *BMC Med*. 2014;12(1):215. doi:10.1186/s12916-014-0215-1.

65. Aune D, Norat T, Romundstad P, Vatten LJ. Whole grain and refined grain consumption and the risk of type 2 diabetes: A systematic review and dose-response meta-analysis of cohort studies. *Eur J Epidemiol*. 2013;28(11):845-858. doi:10.1007/s10654-013-9852-5.

66. Ding M, Bhupathiraju SN, Chen M, van Dam RM, Hu FB. Caffeinated and decaffeinated coffee consumption and risk of type 2 diabetes: a systematic review and a dose-response meta-analysis. *Diabetes Care*. 2014;37(2):569-586. doi:10.2337/dc13-1203.

67. Li X-H, Yu F-F, Zhou Y-H, He J. Association between alcohol consumption and the risk of incident type 2 diabetes: a systematic review and dose-response meta-analysis. *Am J Clin Nutr*. 2016;103(3):818-829. doi:10.3945/ajcn.115.114389.

68. Muraki I, Imamura F, Manson JE, et al. Fruit consumption and risk of type 2 diabetes: results from three prospective longitudinal cohort studies. *BMJ*. 2013;347(aug28 1):f5001-f5001. doi:10.1136/bmj.f5001.

69. Leong A, Daya N, Porneala B, et al. Prediction of Type 2 Diabetes by Hemoglobin A1c in Two Community-Based Cohorts. *Diabetes Care*. 2017;41(1):dc170607. doi:10.2337/dc17-0607.

70. Zhang X, Gregg EW, Williamson DF, et al. A1C level and future risk of diabetes: A systematic review. *Diabetes Care*. 2010. doi:10.2337/dc09-1939.

71. Maruthur NM, Ma Y, Delahanty LM, et al. Early response to preventive strategies in the diabetes prevention program. *J Gen Intern Med*. 2013;28(12):1629-1636. doi:10.1007/s11606-013-2548-4.

72. Khaw K-T, Wareham N, Bingham S, Luben R, Welch A, Day N. Association of hemoglobin A1c with cardiovascular disease and mortality in adults: the European prospective investigation into cancer in Norfolk. *Ann Intern Med*. 2004;141(6):413-420. doi:10.7326/0003-4819-141-6-200409210-00006.

73. Sarwar N, Aspelund T, Eiriksdottir G, et al. Markers of dysglycaemia and risk of coronary heart disease in people without diabetes: Reykjavik prospective study and systematic review. *PLoS Med*. 2010;7(5). doi:10.1371/journal.pmed.1000278.

74. Stratton IM, Adler AI, Neil HAW, et al. Association of glycaemia with macrovascular and prospective observational study. *Br Med J*. 2000:405-412.

doi:10.1136/bmj.321.7258.405.

75. Upadhyaya S, Banerjee G. Type 2 diabetes and gut microbiome: At the intersection of known and unknown. *Gut Microbes*. 2015;6(2):85-92. doi:10.1080/19490976.2015.1024918.

76. David LA, Maurice CF, Carmody RN, et al. Diet rapidly and reproducibly alters the human gut microbiome. *Nature*. 2014;505(7484):559-563. doi:10.1038/nature12820.

77. Duncan SH, Belenguer A, Holtrop G, Johnstone AM, Flint HJ, Lobley GE. Reduced dietary intake of carbohydrates by obese subjects results in decreased concentrations of butyrate and butyrate-producing bacteria in feces. *Appl Environ Microbiol*. 2007;73(4):1073-1078. doi:10.1128/AEM.02340-06.

78. Dorans KS, Mostofsky E, Levitan EB, Håkansson N, Wolk A, Mittleman MA. Alcohol and Incident Heart Failure among Middle-Aged and Elderly Men: Cohort of Swedish Men. *Circ Hear Fail*. 2015;8(3):422-427. doi:10.1161/CIRCHEARTFAILURE.114.001787.

79. Steinhaus DA, Mostofsky E, Levitan EB, et al. Chocolate intake and incidence of heart failure: Findings from the Cohort of Swedish Men. *Am Heart J*. 2017;183:18-23. doi:10.1016/j.ahj.2016.10.002.

80. Dorans KS, Massa J, Chitnis T, Ascherio A, Munger KL. Physical activity and the incidence of multiple sclerosis. *Neurology*. 2016;87(17):1770-1776. doi:10.1212/WNL.0000000000003260.

81. Mills KT, Chen J, Yang W, et al. Sodium Excretion and the Risk of Cardiovascular Disease in Patients With Chronic Kidney Disease. *JAMA*. 2016;315(20):2200. doi:10.1001/jama.2016.4447.

82. Chen J, Gu D, Huang J, et al. Metabolic syndrome and salt sensitivity of blood pressure in non-diabetic people in China: a dietary intervention study. *Lancet*. 2009;373(9666):829-835. doi:10.1016/S0140-6736(09)60144-6.

83. Li C, He J, Chen J, et al. Genome-Wide Gene-Sodium Interaction Analyses on Blood Pressure: The Genetic Epidemiology Network of Salt-Sensitivity Study. *Hypertension*. 2016;68(2):348-355. doi:10.1161/HYPERTENSIONAHA.115.06765.

84. He J, Streiffer RH, Muntner P, Krousel-Wood MA, Whelton PK. Effect of dietary fiber intake on blood pressure: a randomized, double-blind, placebo-controlled trial. *J Hypertens*. 2004;22(1):73-80. doi:10.1097/01.hjh.0000098164.70956.54.

85. He J, Ogden LG, Vupputuri S, Bazzano LA, Loria C, Whelton PK. Dietary sodium intake and subsequent risk of cardiovascular disease in overweight adults. *JAMA*. 1999;282(21):2027-2034. doi:10.1001/jama.282.21.2027.

86. He J, Wofford MR, Reynolds K, et al. Effect of dietary protein supplementation on blood pressure a randomized, controlled trial. *Circulation*. 2011;124(5):589-595. doi:10.1161/CIRCULATIONAHA.110.009159.

87. Thompson AM, Zhang Y, Tong W, et al. Association of inflammation and endothelial dysfunction with metabolic syndrome, prediabetes and diabetes in adults from Inner Mongolia, China. *BMC Endocr Disord*. 2011;11(1):16. doi:10.1186/1472-6823-11-16.

88. Irazola V, Rubinstein A, Bazzano L, et al. Prevalence, awareness, treatment and control of diabetes and impaired fasting glucose in the Southern Cone of Latin America. *PLoS One*. 2017;12(9). doi:10.1371/journal.pone.0183953.

89. Bazzano LA, Li TY, Joshipura KJ, Hu FB. Intake of fruit, vegetables, and fruit juices and risk of diabetes in women. *Diabetes Care*. 2008;31(7):1311-1317. doi:10.2337/dc08-0080.

90. Bazzano LA, Serdula M, Liu S. Prevention of Type 2 Diabetes by Diet and Lifestyle Modification. *J Am Coll Nutr*. 2005;24(5):310-319. doi:10.1080/07315724.2005.10719479.
91. Pollock BD, Chen W, Harville EW, et al. Differential sex effects of systolic blood pressure and low-density lipoprotein cholesterol on type 2 diabetes: Life course data from the Bogalusa Heart Study. *Journal of Diabetes*. 2017.
92. Zhang T, Li Y, Zhang H, et al. Insulin-sensitive adiposity is associated with a relatively lower risk of diabetes than insulin-resistant adiposity: the Bogalusa Heart Study. *Endocrine*. 2016;54(1):93-100. doi:10.1007/s12020-016-0948-z.
93. Pollock BD, Hu T, Chen W, et al. Utility of existing diabetes risk prediction tools for young black and white adults: Evidence from the Bogalusa Heart Study. *J Diabetes Complications*. 2016;31(1):86-93. doi:10.1016/j.jdiacomp.2016.07.025.
94. Hu T, Yao L, Reynolds K, et al. The effects of a low-carbohydrate diet vs. a low-fat diet on novel cardiovascular risk factors: A randomized controlled trial. *Nutrients*. 2015;7(9):7978-7994. doi:10.3390/nu7095377.
95. Hu T, Yao L, Reynolds K, et al. The effects of a low-carbohydrate diet on appetite: A randomized controlled trial. *Nutr Metab Cardiovasc Dis*. 2016;26(6):476-488. doi:10.1016/j.numecd.2015.11.011.
96. Rebholz CM, Reynolds K, Wofford MR, et al. Effect of soybean protein on novel cardiovascular disease risk factors: A randomized controlled trial. *Eur J Clin Nutr*. 2013;67(1):58-63. doi:10.1038/ejcn.2012.186.
97. Wofford MR, Rebholz CM, Reynolds K, et al. Effect of soy and milk protein supplementation on serum lipid levels: A randomized controlled trial. *Eur J Clin Nutr*. 2012;66(4):419-425. doi:10.1038/ejcn.2011.168.
98. Chen J, He J, Wildman RP, Reynolds K, Streiffer RH, Whelton PK. A randomized controlled trial of dietary fiber intake on serum lipids. *Eur J Clin Nutr*. 2006;60(1):62-68. doi:10.1038/sj.ejcn.1602268.
99. Overweight and Obesity Expert Panel. *Managing Overweight and Obesity in Adults: Systematic Evidence Review From the Obesity Expert Panel, 2013.*; 2013.
100. The Data Center. *Who Lives in New Orleans and Metro Parishes Now?*; 2017. https://www.datacenterresearch.org/data-resources/who-lives-in-new-orleans-now/.
101. Wetzels JFM, Kiemeney LALM, Swinkels DW, Willems HL, Heijer M Den. Age- and gender-specific reference values of estimated GFR in Caucasians: The Nijmegen Biomedical Study. *Kidney Int*. 2007;72(5):632-637. doi:10.1038/sj.ki.5002374.
102. Radin MS. Pitfalls in hemoglobin A1c measurement: When results may be misleading. *J Gen Intern Med*. 2014;29(2):388-394. doi:10.1007/s11606-013-2595-x.
103. Kim PS, Woods C, Georgoff P, et al. A1C underestimates glycemia in HIV infection. *Diabetes Care*. 2009;32(9):1591-1593. doi:10.2337/dc09-0177.
104. Qaseem A, Wilt TJ, Kansagara D, Horwitch C, Barry MJ, Forciea MA. Hemoglobin A1c Targets for Glycemic Control With Pharmacologic Therapy for Nonpregnant Adults With Type 2 Diabetes Mellitus: A Guidance Statement Update From the American College of Physicians. *Ann Intern Med*. 2018;168(8):569-576. doi:10.7326/M17-0939.
105. Sumithran P, Prendergast LA, Delbridge E, et al. Ketosis and appetite-mediating nutrients and hormones after weight loss. *Eur J Clin Nutr*. 2013;67(7):759-764. doi:10.1038/ejcn.2013.90.
106. Gardner CD, Trepanowski JF, Gobbo LCD, et al. Effect of low-fat VS low-carbohydrate

diet on 12-month weight loss in overweight adults and the association with genotype pattern or insulin secretion the DIETFITS randomized clinical trial. *JAMA - J Am Med Assoc*. 2018;319(7):667-679. doi:10.1001/jama.2018.0245.

107. Brenner BM, Meyer TW, Hostetter TH. Dietary protein intake and the progressive nature of kidney disease: the role of hemodynamically mediated glomerular injury in the pathogenesis of progressive glomerular sclerosis in aging, renal ablation, and intrinsic renal disease. *N Engl J Med*. 1982;307(11):652-659. doi:10.1056/NEJM198209093071104.

108. Sato J, Kanazawa A, Makita S, et al. A randomized controlled trial of 130 g/day low-carbohydrate diet in type 2 diabetes with poor glycemic control. *Clin Nutr*. 2017;36(4):992-1000. doi:10.1016/j.clnu.2016.07.003.

109. Yancy WS, Foy M, Chalecki AM, et al. A low-carbohydrate, ketogenic diet to treat type 2 diabetes. *Nutr Metab (Lond)*. 2005;2(1):34. doi:10.1186/1743-7075-2-34.

110. Boden G, Sargrad K, Homko C, Mozzoli M, Stein TP. Effect of a low-carbohydrate diet on appetite, blood glucose levels, and insulin resistance in obese patients with type 2 diabetes. *Ann Intern Med*. 2005;142(6). doi:10.1016/S0084-3741(08)70336-6.

111. Stern L, Iqbal N, Seshadri P. The Effects of Low-Carbohydrate versus Conventional Weight Loss Diets in Severely Obese Adults : One-Year Follow-up of a randomized control trial. *Ann Intern …*. 2004;140(May 2001):769-777. doi:10.1016/j.accreview.2004.07.103.

112. Westman EC, Yancy WS, Edman JS, Tomlin KF, Perkins CE. Effect of 6-month adherence to a very low carbohydrate diet program. *Am J Med*. 2002;113(1):30-36. doi:10.1016/S0002-9343(02)01129-4.

113. Moosheer SM, Waldschütz W, Itariu BK, Brath H, Stulnig TM. A protein-enriched low glycemic index diet with omega-3 polyunsaturated fatty acid supplementation exerts beneficial effects on metabolic control in type 2 diabetes. *Prim Care Diabetes*. 2014;8(4):308-314. doi:10.1016/j.pcd.2014.02.004.

114. Aagaard K, Petrosino J, Keitel W, et al. The Human Microbiome Project strategy for comprehensive sampling of the human microbiome and why it matters. *FASEB J*. 2013;27(3):1012-1022. doi:10.1096/fj.12-220806.

115. McInnes P, Cutting M. Manual of Procedures for Human Microbiome Project. http://www.hmpdacc.org/doc/HMP_MOP_Version12_0_072910.pdf.

116. Parker AR, Byham-Gray L, Denmark R, Winkle PJ. The Effect of Medical Nutrition Therapy by a Registered Dietitian Nutritionist in Patients with Prediabetes Participating in a Randomized Controlled Clinical Research Trial. *J Acad Nutr Diet*. 2014;114(11):1739-1748. doi:10.1016/j.jand.2014.07.020.

117. Marrero DG, Palmer KNB, Phillips EO, Miller-Kovach K, Foster GD, Saha CK. Comparison of commercial and self-initiated weight loss programs in people with prediabetes: A randomized control trial. *Am J Public Health*. 2016;106(5):949-956. doi:10.2105/AJPH.2015.303035.

118. Cole RE, Boyer KM, Spanbauer SM, Sprague D, Bingham M. Effectiveness of Prediabetes Nutrition Shared Medical Appointments. *Diabetes Educ*. 2013;39(3):344-353. doi:10.1177/0145721713484812.

119. Brownlee M, Hirsch IB. Glycemic variability: a hemoglobin A1c-independent risk factor for diabetic complications. *JAMA*. 2006;295(14):1707-1708. doi:10.1001/jama.295.14.1707.

120. Smith-Palmer J, Brändle M, Trevisan R, Orsini Federici M, Liabat S, Valentine W. Assessment of the association between glycemic variability and diabetes-related

complications in type 1 and type 2 diabetes. *Diabetes Res Clin Pract*. 2014;105(3):273-284. doi:10.1016/j.diabres.2014.06.007.

121. Nalysnyk L, Hernandez-Medina M, Krishnarajah G. Glycaemic variability and complications in patients with diabetes mellitus: Evidence from a systematic review of the literature. *Diabetes, Obes Metab*. 2010;12(4):288-298. doi:10.1111/j.1463-1326.2009.01160.x.
122. J. T, N.D. L-M, C.H. T, et al. Comparison of low- and high-carbohydrate diets for type 2 diabetes management: A randomized trial. *Am J Clin Nutr*. 2015;102(4):780-790. doi:10.3945/ajcn.115.112581.1.
123. Service FJ, Molnar GD, Rosevear JW, Ackerman E, Gatewood LC, Taylor WF. Mean amplitude of glycemic excursions, a measure of diabetic instability. *Diabetes*. 1970;19(9):644-655. doi:10.2337/diab.19.9.644.
124. Monnier L, Colette C, Owens DR. The application of simple metrics in the assessment of glycaemic variability. *Diabetes and Metabolism*. 2018.
125. Monnier L, Mas E, Ginet C, et al. Activation of oxidative stress by acute glucose fluctuations compared with sustained chronic hyperglycemia in patients with type 2 diabetes. *J Am Med Assoc*. 2006;295(14):1681-1687. doi:10.1001/jama.295.14.1681.
126. Torimoto K, Okada Y, Mori H, Tanaka Y. Relationship between fluctuations in glucose levels measured by continuous glucose monitoring and vascular endothelial dysfunction in type 2 diabetes mellitus. *Cardiovasc Diabetol*. 2013;12(1). doi:10.1186/1475-2840-12-1.
127. Su G, Mi S, Tao H, et al. Association of glycemic variability and the presence and severity of coronary artery disease in patients with type 2 diabetes. *Cardiovasc Diabetol*. 2011;10. doi:10.1186/1475-2840-10-19.
128. Diabetes Prevention Program Research Group. Reduction in the Incidence of Type 2 Diabetes With Lifestyle Intervention or Metformin. *N Engl J Med*. 2002;346(6):393-403. doi:10.1056/NEJMoa012512.
129. Hostalek U, Gwilt M, Hildemann S. Therapeutic Use of Metformin in Prediabetes and Diabetes Prevention. *Drugs*. 2015;75(10):1071-1094. doi:10.1007/s40265-015-0416-8.
130. Bailey T, Bode BW, Christiansen MP, Klaff LJ, Alva S. The Performance and Usability of a Factory-Calibrated Flash Glucose Monitoring System. *Diabetes Technol Ther*. 2015;17(11):787-794. doi:10.1089/dia.2014.0378.
131. Færch K, Amadid H, Nielsen LB, et al. Protocol for a randomised controlled trial of the effect of dapagliflozin, metformin and exercise on glycaemic variability, body composition and cardiovascular risk in prediabetes (the PRE-D Trial). *BMJ Open*. 2017;7(5). doi:10.1136/bmjopen-2016-013802.
132. Hanefeld M, Sulk S, Helbig M, Thomas A, Köhler C. Differences in glycemic variability between normoglycemic and prediabetic subjects. *J Diabetes Sci Technol*. 2014;8(2):286-290. doi:10.1177/1932296814522739.
133. Rodriguez-Segade S, Rodriguez J, Camiña F, et al. Continuous glucose monitoring is more sensitive than HbA1c and fasting glucose in detecting dysglycaemia in a Spanish population without diabetes. *Diabetes Res Clin Pract*. 2018;142:100-109. doi:10.1016/j.diabres.2018.05.026.
134. Le Floch JP, Escuyer P, Baudin E, Baudon D, Perlemuter L. Blood glucose area under the curve. Methodological aspects. *Diabetes Care*. 1990;13(2):172-175. doi:10.2337/diacare.13.2.172.
135. Vogelzang M, van der Horst ICC, Nijsten MWN. Hyperglycaemic index as a tool to

assess glucose control. *Crit Care*. 2004;8(3):R122-R127. doi:10.1186/cc2840.

Low-Carbohydrate Dietary Pattern on Glycemic Outcomes Trial

PROTOCOL

PROTOCOL VERSION 1.3

Dated: December 17, 2018

New Orleans, LA

Table of Contents

1. Overview 5
2. Background and Significance 6
2.A. Type 2 Diabetes Mellitus is a major global public health challenge 6
2.B. Prediabetes is a major risk factor for cardiometabolic diseases 6
2.C. Role of lifestyle in preventing diabetes 6
2.D. Low-to-moderate carbohydrate diets, weight loss, and cardiometabolic diseases 7
2.E. Mediterranean-style diet is associated with reduced risk of T2D and improved glycemic control 8
2.F. Evidence for low-carbohydrate diets that emphasize Mediterranean-style dietary components 8
2.G. Evidence for specific food groups and risk of T2D 8
2.H. Gut microbiota, diet and T2D 9
2.I. Clinical and public health significance of the proposed study 9
2.J. Innovation 9
3. Preliminary Studies 10
3.A. Summary of aggregate experience of the research team 10
3.B. Low-carbohydrate behavioral dietary intervention trial experience 10
3.C. Other dietary intervention trial experience 11
4. Study Design and Organization 11
4.A. Overview of trial design 11
4.B. Study outcomes 12
4.B.1. Primary outcome 12
4.B.2. Secondary outcomes 12
4.C Study Timeline 12
6. Study Participants 13
6.A. Eligibility criteria 13
7. Recruitment and Randomization 13
7.A. Recruitment 13
7.A.1. Mass mailing, placement of brochures/flyers in community, TV/radio/billboard advertisements 14
7.A.2. Database searches and targeted mailing 14
7.A.3. Referrals and Local Partnerships 14
7.B. Randomization 14
7.C. Masking 15

7.D. Safety Considerations ... 15
8. Dietary Intervention Program ... 15
8.A. Low-carbohydrate dietary intervention group ... 15
8.B. Usual Diet Group ... 16
8.C. Physical Activity ... 16
8.D. Dietary Adherence ... 17
9. Safety of Dietary Intervention ... 17
10. Data Collection ... 18
10.A. Study visits ... 18
10.A.1. Screening/baseline visits ... 18
10.A.2. Follow-up visits and final visit ... 19
10.B Study measurements ... 19
10.B.1. Questionnaires ... 19
10.B.2. Dietary measurements ... 19
10.B.3. Assessment and management of adverse effects ... 20
10.B.4. BP and anthropometric measures ... 20
10.B.5. Blood samples ... 20
10.B.6. Urine collection ... 21
10.B.7. Stool Specimen Collection ... 21
11. Quality Assurance and Quality Control ... 21
11.A. Manual of procedures ... 21
11.B. Training and certification ... 21
12. Sample Size and Statistical Power ... 22
13. Data Management and Analysis ... 23
13.A. Data management ... 23
13.B. Data analysis plan ... 23
13.B.1. Exploratory data analysis ... 23
13.B.2. Primary, secondary, and exploratory outcomes ... 23
15. Anticipated Challenges ... 23
16. Protection of Human Subjects ... 24
16.A. Protection of human subjects from research risks ... 24
16.A.1. Risks to the subjects ... 24
16.A.2. Adequacy of protection against risks ... 25

16.A.3. Potential benefits of the proposed research to human subjects and others... 26
16.A.4. Importance of knowledge to be gained... 27
16.A.5. Remuneration... 27
16.B. Data safety and monitoring plan... 27
Bibliography and References Cited... 29

1. Overview

The increasing prevalence of type 2 diabetes mellitus (T2D), a major cause of morbidity and mortality worldwide, is a critical public health concern. In the United States, an estimated 33.9% of adults have prediabetes (impaired fasting glucose, elevated hemoglobin A1c (HbA1c), or impaired glucose tolerance). Individuals with prediabetes are at elevated risk of T2D and cardiovascular disease (CVD), with 5–10% developing clinical T2D each year.

In the short-term, among patients with T2D, low-to-moderate carbohydrate diets (energy percentage below 45) have a greater glucose-lowering effect than do high-carbohydrate diets, with larger glucose-lowering effect the greater the carbohydrate reduction. Additionally, compared with low-fat diets, Mediterranean diets that are high in unsaturated fats (particularly *cis*-monounsaturated fats) reduce risk of T2D and CVD and improve glycemic control among diabetics. However, compared with usual diets, the effect of a behavioral intervention promoting a low-carbohydrate/high-unsaturated fat and protein dietary pattern that emphasizes consumption of non-starchy vegetables, plant-based oils, fish, poultry, and mixed nuts on glucose homeostasis, and other metabolic risk factors among individuals with prediabetes or early-stage untreated T2D is not well understood.

Our *long-term goal* is to develop and implement dietary intervention(s) that reduce risk of T2D. The *overall goal of this randomized controlled trial is to study the effect of a behavioral intervention promoting a low-carbohydrate/high-unsaturated fat and high-protein dietary pattern (initial target <40 g digestible carbohydrates, final target <60 g digestible carbohydrates) compared with a usual diet on metabolic risk factors among individuals with prediabetes or early-stage untreated diabetes* (HbA1c 6.0–6.9%). Specifically, we will test the following hypotheses:

Primary hypothesis: Compared with usual diet, over a 6-month period, a healthy low-carbohydrate/high unsaturated fat and high-protein dietary pattern will lead to *a larger decrease of HbA1c, by at least 0.15%.*

Secondary hypothesis: Over a 6-month period, a healthy low-carbohydrate/high-unsaturated fat and high-protein dietary pattern will lead to *larger improvements in the following major secondary outcomes*: fasting glucose, systolic blood pressure (BP), total-to-HDL-cholesterol ratio, and body weight.

Exploratory outcomes: We will also study the effect of the intervention on the following outcomes: insulin and homeostasis model assessment of insulin resistance (HOMA-IR); diastolic BP; waist circumference; and estimated CVD risk.

To achieve these study aims, we will recruit 112 adults with prediabetes or untreated diabetes (HbA1c 6.0–6.9%) in the Greater New Orleans, LA area. Using a stratified, blocked randomization scheme, participants will be assigned to either a behavioral modification intervention aimed at promoting a healthy low-carbohydrate dietary pattern or to usual diet. The intervention group will have an intensive phase for 3 months, followed by a maintenance phase in months 4 through 6. The *primary outcome will be difference between the active intervention and control groups in the change in HbA1c from baseline to 6 months of follow-up. We expect 85% power to detect a difference of at least 0.15% in change of HbA1c.* Changes in other metabolic risk factors will be studied concurrently. Metabolic risk factors will be assessed at baseline, 3 months, and 6 months of follow-up.

There is much interest regarding dietary approaches to prevent T2D and its complications. The results from this study will help to determine whether, compared with usual diet, a behavioral intervention aimed at promoting a healthy low-carbohydrate dietary pattern improves metabolic risk factors.

2. Background and Significance

2.A. Type 2 Diabetes Mellitus is a major global public health challenge

Diabetes is a strong and independent risk factor for CVD morbidity and mortality. A meta-analysis of 102 prospective studies (698,782 participants) estimated diabetes leads to an approximately 2-fold increased risk in vascular diseases, including coronary heart disease (CHD) and major stroke subtypes, and deaths due to other vascular causes.[1] Among participants with diabetes, CHD risk is about 50% higher among those with fasting blood glucose ≥7 mmol/L compared with those with fasting blood glucose <7 mmol/L.[1] In addition to increasing CVD risk, diabetes can cause diabetic retinopathy,[2] chronic kidney disease,[3] lower limb amputation,[4] and other complications. Most individuals with diabetes have T2D.[5] The US Centers for Disease Control and Prevention (CDC) estimated that 30.2 million adults ≥18 years (12.2%) had diabetes in 2015, with 7.2 million (23.8%) not being aware of or reporting diabetes.[5] Diagnosed diabetes prevalence among US adults has increased substantially over time, from 2.54% in 1980 to 7.40% in 2015.[6] Diabetes prevalence is also high globally, with an estimated 2014 age-standardized prevalence of 9.0% in men and 7.9% in women.[7] Diabetes accounts for an increasing portion of US healthcare expenditures. Total cost of diagnosed diabetes in the US was estimated to be $245 billion in 2012, 41% higher than in 2007.[8] Diabetes is associated with much higher lifetime medical expenses, with higher expenses for those diagnosed at younger ages.[9]

2.B. Prediabetes is a major risk factor for cardiometabolic diseases

Prediabetes, defined by the American Diabetes Association (ADA) as impaired fasting glucose (IFG), impaired glucose tolerance (IGT), or elevated HbA1c, is also referred to as "intermediate hyperglycemia"[10] and "high-risk state of developing diabetes".[11] The ADA defines prediabetes as having: fasting plasma glucose of 100–125 mg/dL (IFG); 2-hour post-load glucose in the 75-g oral glucose tolerance test (OGTT) of 140–199 mg/dL (IGT); or HbA1c 5.7–6.4%.[12] Prediabetes itself is not a disease; for all three tests, "risk is continuous, extending below the lower limit of the range and becoming disproportionately greater at the higher end of the range".[12] The CDC estimated that in 2015, 33.9% of US adults had prediabetes based on fasting glucose or HbA1c.[5] Among those with prediabetes, only 11.9% reported having being told this by a medical professional.[5] Approximately 5–10% of individuals with prediabetes develop diabetes within one year, depending on how prediabetes is defined.[13] In a meta-analysis, annual diabetes incidence was 15–19% among individuals with both IFG and IGT, while it was 4–6% in those with isolated IFG and 6–9% in those with isolated IGT.[13]

A recent meta-analysis (1,611,339 participants from 53 studies) reported that prediabetes as defined by the ADA is associated with higher risk of composite CVD events and CHD.[14] IFG and IGT were also positively associated with increased risk of stroke and all-cause mortality.[14] Prediabetes is linked with increased risk of other conditions typically thought of as diabetes complications, such as neuropathies and nephropathies.[15] Importantly, it is possible for individuals with prediabetes to revert to normoglycemia, as observed in observational studies[13] and lifestyle or drug intervention trials.[16,17] In the Diabetes Prevention Program (DPP), reversion to normoglycemia was associated with lower long-term T2D risk[18] and lower predicted CVD risk.[19]

2.C. Role of lifestyle in preventing diabetes

Much evidence from observational studies and clinical trials supports the critical role of diet and other lifestyle factors in preventing T2D. Globally, lifestyle transitions towards reduced physical activity, increased consumption of refined carbohydrates, coupled with resulting changes in body composition, has led to increases in diabetes prevalence and severity.[20] Body

mass index (BMI), waist circumference, and waist-to-height ratio are predictors of T2D.[21] The DPP, which combined calorie restriction and exercise to promote weight loss, substantially reduced risk of T2D (by 58%) among individuals with elevated FPG and IGT;[22] a benefit that persisted in 10-year follow-up analyses.[16] Other trials of lifestyle modification focused on diet and exercise have also shown benefits in study populations in China,[23] Finland,[24] and India.[25]

2.D. Low-to-moderate carbohydrate diets, weight loss, and cardiometabolic diseases

Low-to-moderate carbohydrate diets (<45% of energy from carbohydrates) are popular dietary approaches for losing weight. Weight loss is a key component of the DPP lifestyle intervention[26] and ADA lifestyle recommendations.[12,27] Meta-analyses from our group[28] and others support that low-to-moderate carbohydrate diets are at least as effective as low-fat diets at promoting weight loss. For instance, a meta-analysis reported a 2-kg (95% CI, 3.1–0.9) larger weight loss in low-carbohydrate diet (<120 g carbohydrates/day) arms compared with low-fat diet (<30% energy from fat) arms.[29] Estimated 10-year CVD risk was lower in low-carbohydrate than low-fat arms.[29] This aligns with research published by our team; in a 12-month trial of participants with obesity, a low-carbohydrate diet (target <40 g carbohydrates/day) was more effective than a low-fat diet (target <30% energy from fat, <7% saturated fat) for weight loss and CVD risk factor reduction.[30]

Low-to-moderate carbohydrate diets may also be beneficial among patients with T2D. Low-carbohydrate diets have come in and out of favor as treatment of diabetes.[31] In a meta-analysis (1376 patients;10 trials), after 3 or 6 months of the intervention, low-to-moderate carbohydrate diets (<45% energy from carbohydrates) led to a 0.34% lower HbA1c (95% CI, 0.06–0.63%) compared with high-carbohydrate diets.[32] Compared with high-carbohydrate diets, there was a greater reduction in HbA1c the greater the reported carbohydrate restriction.[32] There was no benefit of the low-to-moderate carbohydrate diets on HbA1c at 12 months or longer, and no difference between interventions on BMI, body weight, low-density lipoprotein (LDL) cholesterol or quality of life.[32] However, results may have been affected by dietary adherence or glucose medication. There was evidence of diabetes medication reduction in the low-carbohydrate arms in some studies,[33–37] with evidence of medication reduction and improved blood glucose stability persisting for up to 2 years.[38] Hypoglycemia (though not after medication adjustment) was observed in three subjects taking insulin or a sulfonylurea out of twelve subjects in the low-carbohydrate arm of another study.[39] In a subset of participants with T2D (n=46) from a large weight loss trial, compared with those randomized to a low-fat diet and orlistat, those randomized to a low-carbohydrate diet for 48 weeks had improved HbA1c (0.8% lower, 95% CI, -1.5, -0.02%) and a reduction in antiglycemic medications, despite similar weight loss effects of the interventions.[40]

Though low-to-moderate carbohydrate diets have been studied among overweight and obese participants (primarily focused on weight loss), less is understood about the effects of such diets on glycemic outcomes specifically among individuals with prediabetes. A study among participants with IGT observed a reduction at 12 months in weight, HbA1c, and other glycemic outcomes among individuals who participated in a 7-day in-hospital education program, though this was not randomized.[41] A randomized pilot trial of overweight or obese individuals with T2D or prediabetes found that, over 3 months and at 12 months, a very low-carbohydrate diet improved HbA1c, led to higher medication discontinuation, and more weight loss compared with a low-fat diet.[34,37] However, only 4 individuals had prediabetes. Given benefits of low-to-moderate carbohydrate diets for weight loss in general populations and for glycemic control among patients with T2D, further study of these diets are warranted among individuals with prediabetes who are at elevated cardiometabolic disease risk.

2.E. Mediterranean-style diet is associated with reduced risk of T2D and improved glycemic control

Though there is no one "Mediterranean diet", the Mediterranean dietary pattern tends to have the following characteristics: high consumption of vegetables, fruits, breads and cereals, legumes, nuts and seeds; low-to-moderate consumption of dairy products, fish, poultry and eggs; little consumption of red meat and sweets; and red wine in moderation.[42] Olive oil is an important source of fat. In many observational studies, adherence to the Mediterranean-style dietary pattern is associated with reduced risk of CVD[43] and T2D.[44,45] Evidence from randomized trials also supports the benefits of this dietary pattern. In several trials, participants randomized to Mediterranean diets had substantially lower risk of CVD than those randomized to low-fat control diets.[46,47] In the three-arm randomized PREDIMED trial of participants at high risk of CVD, after 4.1 years of follow-up, those randomized to a traditional Mediterranean diet supplemented with either olive oil or nuts had a 30% reduced risk of T2D compared to those in the low-fat control diet group.[48] In all PREDIMED arms, average carbohydrate intake was relatively low (39.7–43.7% at end of trial).[47] Two trials have found a higher rate of remission from metabolic syndrome among individuals randomized to a Mediterranean diet compared with those randomized to control diets (prudent or low-fat) during 2–5 years of follow-up.[49,50] The Mediterranean pattern may also be beneficial among individuals with T2D. Meta-analyses have reported that, among patients with T2D, those randomized to a Mediterranean diet had lower HbA1c levels and CVD risk factors, and more significant weight loss, compared with those randomized to control diets.[51]

2.F. Evidence for low-carbohydrate diets that emphasize Mediterranean-style dietary components

Many low-carbohydrate diets involve increased consumption of food items, such as red meat,[52] that are associated with increased cardiometabolic disease risk. However, low-carbohydrate dietary approaches can focus on substituting carbohydrates with foods common in Mediterranean-style diets, such as olive oil and nuts, which may improve cardiometabolic health. In a 4-year Italian study of individuals with newly diagnosed T2D, those randomized to a low-carbohydrate Mediterranean diet (<50% carbohydrates; consumed ~44%) had more favorable changes in glycemic control, higher rates of T2D remission, and delayed need for antiglycemic drugs compared with participants randomized to a low-fat diet (<30% fat, <10% saturated fat).[53,54] A 12-month Israeli study assessed low-carbohydrate Mediterranean (35% carbohydrates), traditional Mediterranean, and ADA (both 50–55% carbohydrates) diets in 259 overweight participants with T2D.[55] Most CVD risk factors improved in all groups; there was only HDL improvement in the low-carbohydrate Mediterranean diet and this diet was superior to the other diets in improving glycemic control.[55] In a 2-year weight-loss study of moderately obese participants, low-carbohydrate and Mediterranean diets were superior to low-fat diet for weight loss.[56] Participants in the low-carbohydrate arm were counseled to choose vegetarian sources of fat and protein and to avoid trans fat. At 6 years after study commencement, despite weight regain, there were still long-term benefits, particularly for the low-carbohydrate and Mediterranean diets.[57] In whole, evidence supports that low-carbohydrate Mediterranean-style diets are feasible and may have metabolic benefits.

2.G. Evidence for specific food groups and risk of T2D

Prospective cohort studies have found consumption of specific foods and beverages is associated with diabetes risk. Meta-analyses report higher consumption of processed and unprocessed red meat,[58] white rice (particularly in Asian populations),[59] and sugar-sweetened beverages[60,61] are associated with higher T2D risk. Consumption of green leafy vegetables,[62,63] yogurt,[64] whole grains,[65] decaffeinated and total coffee,[66] and light-to-moderate alcohol

consumption[67] are associated with reduced risk. Consumption of fruits, particularly blueberries, grapes, and apples, are associated with reduced risk; fruit juice is associated with higher risk.[68]

2.H. Gut microbiota, diet and T2D

There is increasing evidence for the importance of gut microbiota in risk of type 2 diabetes and other cardiometabolic diseases.[69] Additionally, dietary modifications, including changes in carbohydrate consumption, can lead to substantial changes in gut microbiome over short periods of time.[70,71] Less is known about longer-term changes to the gut microbiota during the adoption of a low-carbohydrate diet and how these changes may influence cardiometabolic risk. We plan to collect stool specimens at baseline and at 6 months, for potential future studies on how this intervention affects the gut microbiome.

2.I. Clinical and public health significance of the proposed study

Prediabetes, or intermediate hyperglycemia, is a marker of high risk of developing T2D and other complications. Due to increasing prevalence of T2D and associated morbidity and mortality globally, a better understanding of dietary approaches to delay progression to T2D or to promote reversion to normoglycemia is a high public health priority. This study proposes to assess the effect of a behavioral intervention promoting a low-carbohydrate/high-unsaturated fat dietary pattern (initial target <40 g digestible carbohydrates, final target <60 g digestible carbohydrates) on HbA1c, a marker of glycemic control, and other metabolic risk factors. Building from prior findings from clinical and observational studies, we will develop a dietary intervention that promotes the consumption of non-starchy vegetables, plant-based oils, fish, poultry, and mixed nuts, that are thought to reduced risk of T2D or CVD. Participants assigned to the intervention will be trained in behavioral techniques that will provide capacity to maintain dietary changes long after study completion. Our outcome measures will assess the effect of this dietary intervention in a “real-life” setting as opposed to dietary modifications achieved in metabolic ward studies or through food prepared in research kitchens. Findings from this study may provide evidence in support of promoting alternative dietary styles among individuals with high T2D and CVD risk and could potentially be used to inform dietary interventions used in programs like DPP.

2.J. Innovation

The status quo as it pertains to dietary approaches for reducing risk of T2D is focused on reducing caloric intake and total fat intake. However, recent evidence suggests that the types of fat, rather than the quantity of fats may be more important, with evidence supporting that a Mediterranean-style diet that does not restrict fat intake reduces risk of developing both T2D[48] and CVD[47]. Moreover, in the short-term, low-carbohydrate diets, which are popular weight loss diets, have a greater glucose-lowering effect than do high-carbohydrate diets among patients with T2D.[32] However, the effect of low-carbohydrate diets that emphasize consumption of non-starchy vegetables, plant-based oils, fish, poultry, and mixed nuts on T2D prevention is unclear, particularly compared with higher-carbohydrate diets more commonly consumed in the US. *The proposed research is innovative, in our opinion, because it represents a substantial departure from the status quo by assessing the combined effect of a low-carbohydrate, high unsaturated fat and protein diet on cardiometabolic risk factors among adults with prediabetes or early-stage untreated diabetes.* We expect the results from this study have the potential to lead to new horizons for developing and implementing dietary approaches (other than the most commonly used reduced fat diet) that will substantially reduce risk of cardiometabolic disease among adults with T2D or at high risk of T2D. The proposed intervention dietary approach promotes consumption of dietary components thought to reduce risk of cardiometabolic disease and the behavior change intervention has expected applicability in clinical practice.

3. Preliminary Studies

3.A. Summary of aggregate experience of the research team

Dr. Kirsten Dorans is trained in cardiovascular disease epidemiology, with strong training in epidemiologic methods and biostatistics. Her publication record includes studies of behavioral risk factors and risk of chronic disease. She has published on the relationships between dietary intake and chronic heart failure[72,73] and on physical activity and incident multiple sclerosis[74]. She served as lead author, conducting data management, analysis, drafting, and revision of two of these manuscripts. In graduate school, she focused on studying links between outdoor air pollution and chronic diseases and has published several papers in this field. The COBRE program will provide an excellent opportunity for the JFI to transition from air pollution research to clinical and translational research focused on nutrition and diabetes. Building off of her training in observational epidemiology, this program will involve training and practical experience that will allow her to broaden her expertise and to develop practical skills for carrying out clinical research.

The mentorship team has much experience and prior successful collaborations in key areas related to this study. Dr. Jiang He is an internationally renowned expert in cardiometabolic disease clinical and epidemiological research. He has served as PI for many epidemiological studies and clinical trials, including clinical and observational studies focused on dietary consumption and chronic diseases[75–80] and has conducted extensive global research on diabetes.[81,82] Dr. Bazzano has collaborated extensively with Dr. He for more than 15 years in research related to diet and chronic disease. She is a clinical investigator and has expertise in nutritional epidemiology and clinical research. Dr. Bazzano was the PI of a highly successful clinical trial comparing the effects of low-carbohydrate and low-fat diets on weight and cardiovascular risk factors among individuals with obesity, which was a subproject within the Tulane COBRE in Hypertension and Renal Biology.[30] Dr. He served as Dr. Bazzano's mentor for this clinical trial. They have collaborated on much other work related to dietary and chronic disease, including a meta-analysis on low-carbohydrate diets.[28] Dr. Bazzano has also published many articles on diet and risk of diabetes[83,84] and on diabetes in the Bogalusa Heart Study.[85–87]

3.B. Low-carbohydrate behavioral dietary intervention trial experience

In the study on macronutrient composition of diet and risk factors for cardiovascular disease (MACRO) study, 148 individuals without clinical cardiovascular disease or diabetes and with obesity were randomly assigned to a low-carbohydrate or a low-fat behavioral dietary intervention for 12 months. In the low-carbohydrate arm, daily dietary intake of carbohydrates decreased from an average of 242 g at baseline to 97 g, 93 g, and 127 g at 3, 6, and 12 months, respectively. At 12 months, participants in the low-carbohydrate intervention group had greater decreases in weight (-3.5 kg; 95% CI, -5.6 to -1.4 kg) and fat mass (-1.5%; 95% CI, -2.6% to -0.4%) and greater improvement in several cardiovascular risk factors.[30] This suggests that restricting carbohydrates may be an option for weight loss and reduction of cardiovascular risk factors. Further analyses found that the low-carbohydrate dietary intervention led to similar or greater improvement in inflammation, adipocyte dysfunction, and endothelial dysfunction compared with the low-fat dietary intervention[88] and that the low-fat diet reduced peptide YY more than the low-carbohydrate diet, suggesting satiety may be better preserved on low-carbohydrate diets.[89] No serious adverse events were reported in this study and the number of participants with symptoms were similar between the two diets, with the exception of more participants having headaches on the low-fat diet at 3 months (18 vs 6 participants, $p = 0.03$).[30] Dr. Bazzano served as PI for this study and Dr. He served as her mentor. Dr. Bazzano

mentored one of her PhD students in conducting analyses and writing and editing manuscripts related to this project.

3.C. Other dietary intervention trial experience

Dr. He has also led or been involved with many dietary intervention trials, including both behavioral change and feeding study interventions. In the Protein and Blood Pressure (ProBP) study, 352 adults with prehypertension or stage 1 hypertension in New Orleans, LA and Jackson, MS were assigned to take, in a random order, 40 g/d soy protein, milk protein, or carbohydrate supplementation each for 8 weeks. Compared with the high-glycemic index refined carbohydrate, both soy protein and milk protein supplementation were associated with a -2.0 mm Hg (95% CI -3.2 to -0.7 mm Hg) and -2.3 mm Hg (-3.7 to -1.0 mm Hg) net change in systolic blood pressure.[80] These findings suggest that partial replacement of carbohydrate with soy or milk protein might be an important part of nutrition intervention strategies to prevent and treat hypertension. Additionally, soy protein supplementation led to reduction in plasma E-selectin compared with carbohydrate and milk protein and in plasma leptin compared with carbohydrate.[90] Soy protein also led to improvement in lipid profile compared with carbohydrate and milk protein.[91] Dr. He was PI for this project.

In the Fiber Intervention on Blood Pressure (FIBRE) study, 110 participants ages 30–65 years with untreated pre-hypertension or stage-1 hypertension and serum cholesterol level < 240 mg/dl were randomly assigned to 8-g/day of water soluble fiber from oat bran or a control intervention (double-blind RCT). Over the 12-week study period, net changes in systolic and diastolic blood pressure were -1.8 mm Hg (-4.3 to 0.8) and -1.2 mm Hg (-3.0 to 0.5 mm Hg), respectively.[78] Net changes in total, HDL-, and LDL-cholesterol were -2.40 mg/dL (-10.60 to 5.81 mg/d:), -1.66 mg/dL (-4.55 to 1.22 mg/dL), and -1.33 mg/dl (-8.33 to 5.68 mg/dL), respectively.[92] Dr He was PI for this study.

Dr. He is currently serving as a mentor for Dr. Katherine Mills' COBRE project (Effect of Dietary Sodium Reduction in Kidney Disease Patients with Albuminuria; SUPER). In this project, participants with chronic kidney disease and albuminuria are being randomized to a behavioral modification program designed to reduce dietary sodium intake to ≤2,300 mg/day or to usual care and test the effect of this intervention on change in albumin-to-creatinine ratio from baseline to 24-weeks.

4. Study Design and Organization

4.A. Overview of trial design

We propose to conduct a ***randomized controlled trial*** aimed at testing the effect of a behavioral intervention that promotes *reduced carbohydrate intake and increased consumption of heart-healthy unsaturated fats and protein* in patients with HbA1c 6.0–6.9% (***Figure 1***). We plan to recruit 112 individuals with elevated HbA1c and randomly assign them to a moderately intensive intervention on diet pattern,[93] using well-established methods for behavioral modification, or to usual diet. We will compare the difference between the active intervention and control groups for change in HbA1c from baseline to 6 months of follow-up (primary outcome). We will assess four major secondary outcomes: fasting plasma glucose, systolic BP, total-to-HDL cholesterol ratio, and body weight. In

Figure 1. Study Design

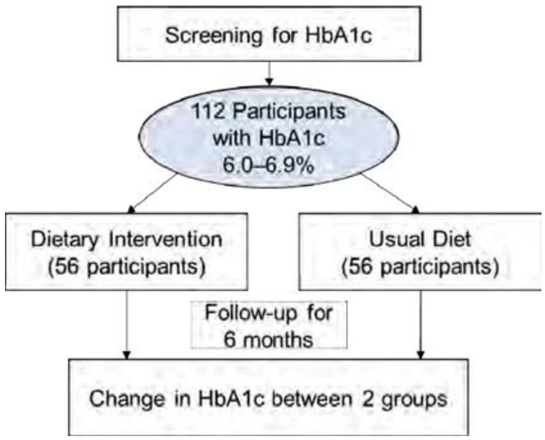

addition, we will look at several other exploratory outcomes: insulin and HOMA-IR, diastolic BP, waist circumference, and estimated CVD risk, as estimated by the 2013 ACC/AHA risk equation. The main trial components are summarized in ***Table 1***.

Table 1. Study Overview

Study Design	Randomized controlled trial
Study Participants	112 participants with elevated HbA1c (6.0–6.9%)
Intervention Group (n=56)	Behavioral modification to reduce carbohydrate consumption and promote consumption of foods such as non-starchy vegetables, plant-based unsaturated oils, nuts and seeds, avocados, eggs, seafood, poultry, lean meat and tofu. We will recommend moderate consumption of cheese, unsweetened Greek yogurt, and low-carb milk. If consuming higher-carbohydrate items, recommend legumes, fruits, (limited) whole grains. Target <40 g net carbohydrates per day for first 3 months and will have a goal of <40 net g carbohydrates per day; <60 g net carbohydrates per day for months 4 onwards.
Usual Diet Group (n=56)	No dietary intervention. To encourage continued participation in the trial, we will offer optional monthly educational sessions on unrelated topics, such as retirement planning or health insurance
Outcome Measures	Primary: HbA1c; Secondary: fasting plasma glucose, systolic BP, total-to-HDL-cholesterol ratio, and body weight

4.B. Study outcomes

4.B.1. Primary outcome: The *primary outcome will be the difference between the intervention group and the usual diet group for change in HbA1c* from baseline to 6 months. Changes in HbA1c will be calculated from blood samples taken at baseline and 6 months.
4.B.2. Secondary outcomes: Secondary outcomes will include changes from baseline to 6 months of *fasting plasma glucose, systolic BP, total-to-HDL cholesterol ratio, and body weight.,*
4.B.3. Other outcomes: We will also study the effect of the intervention on the following outcomes: insulin and homeostasis model assessment of insulin resistance (HOMA-IR); diastolic BP; waist circumference; total-to-HDL-cholesterol ratio; and estimated CVD risk.

4.C Study Timeline

Table 2 shows the timeline, which will be followed in the implementation of study-related activities, such as publications and submission of an R01 grant application.

Table 2. Study Timeline

	Year 1		Year 2		Year 3	
Develop MOP, intervention program, study forms, and pilot testing	X	X				
Certification and training of staff	X					
Recruitment		X	X			
Intervention and data collection, measurement, entry, cleaning		X	X	X	X	
Data quality control and analysis						X
Publications	X	X	X	X	X	X
R01 grant submissions, resubmissions				X	X	X

The first 6 months will focus on development of manual of operations (MOP), intervention program, study forms, study protocol, Institutional Review Board (IRB) approval, and pilot

testing of the intervention program. It will also involve staff training and certification. During the last half of year 1, recruitment, intervention, and data collection will commence. During the last 3 months of year 3, data quality control and analysis will be carried out. Throughout the study period, the JFI will publish peer-reviewed manuscripts from ongoing Tulane University research projects.

6. Study Participants

6.A. Eligibility criteria

Inclusion criteria

- Men or women ages 40 to 70 years: aim to recruit 50% women
- Any race or ethnicity: aim to recruit 40% ethnic minorities (i.e., African American and Hispanic), close to population in the greater New Orleans area[94]
- HbA1c 6.0–6.9%
- Willing and able to provide informed consent

Exclusion criteria

- Diagnosed type 1 diabetes mellitus based on patient self-report
- Use of agents affecting glycemic control (medications for diabetes, oral glucocorticoids) within the past three months prior to enrollment based on patient self-report
- Medical condition in which low-carbohydrate diet may not be advised
    - Estimated glomerular filtration rate (eGFR) ≤45 mL/min/1.73 m², which is close to the 5th percentile of eGFR among non-diseased individuals of 70 years of age[95]
    - Self-report of liver disease due to hepatitis or alcohol; osteoporosis; untreated thyroid disease; gout; or cancer (other than non-melanoma skin cancer) requiring treatment in the past year, unless prognosis is excellent
- Factors that may affect HbA1c:
    - Hemoglobin <11 mg/dL (cutpoint for moderate-to-severe anemia, which could lead to falsely elevated or lowered HbA1c)[96]
    - Recent blood donation or blood transfusion (self-report, past 4 months)
    - Human Immunodeficiency Virus (self-report)[97]
- Allergies to nuts
- For women, current pregnancy, breastfeeding, or plans to become pregnant during the study
- Consumption of ≥21 alcoholic drinks per week or consumption of ≥6 drinks per occasion
- Current or planned residence making it difficult to meet trial requirements (due to distance from study site and/or challenges regularly traveling to site)
- Current participation in another lifestyle intervention trial or a pharmaceutical trial
- Participation of another household member in the study; employees or persons living with employees of the study
- Other concerns regarding ability to meet trial requirements, at the discretion of the principal investigator or study coordinator

7. Recruitment and Randomization

7.A. Recruitment

We will recruit study participants from the greater New Orleans area. The initial focus of our recruitment will be mass mailing of individuals in the greater New Orleans area and recruitment through placing flyers and brochures in the community. If community-based recruitment is not successful or cost-effective, we will recruit out-patients from the Ochsner Health System and

Tulane Medical Center (TMC), from which our group has prior experience recruiting patients. For this recruitment, we will focus on electronic searches of medical records and laboratory databases. We will also carry out targeted solicitation of referrals from physicians who care for patients likely to meet our inclusion and exclusion criteria.

In addition to this recruitment plan, we have several contingency plans: (1) extending recruitment to other medical centers in the New Orleans area, including the Veterans Affairs Medical Center (VAMC), East Jefferson General Hospital, and West Jefferson Medical Center, with whom Tulane has well-established relationships; (2) TV/radio advertisements; (3) arrange a news story about the study in local TV newscasts, radio talk programs, and/or print media; and (4) conduct outreach at local community events and churches. We have ample experience with each of these approaches.

Based on U.S. Census Bureau 2016 population estimates, the metro New Orleans area population was approximately 35% African American or black and 9% Hispanic.[94] Given our prior experience and population distribution in this region, we plan to recruit a distribution of study participants with 40% ethnic minorities.

7.A.1. Mass mailing, placement of brochures/flyers in community, TV/radio/billboard advertisements: Community recruitment approaches include: mass mailing individuals who live in the New Orleans area; brochures/flyers in the community; TV, radio, poster, or billboard advertisements in the New Orleans area. Interested individuals will be pre-screened; those at elevated risk for prediabetes or T2D, i.e. individuals who are obese or overweight and/or have a sufficiently high score from the American Diabetes Association's Diabetes Risk Assessment Questionnaire will be invited into the clinic for a screening visit.

7.A.2. Database searches and targeted mailing: We will request IRB approval for a waiver for HIPAA authorization to conduct database searching and hospital chart reviews for identification of potentially eligible study participants. After identifying a potentially eligible patient, we will send this individual an invitation letter signed by the investigators and/or the individual's personal physician. Individuals who express interest in the study will have a pre-screening telephone interview for confirmation of potential eligibility. Those still interested and potentially eligible for enrollment will be scheduled for a visit at the COBRE study clinic.

7.A.3. Referrals and Local Partnerships: In addition to local physician referrals, we will explore holding outreach community events in the New Orleans area. We may also identify potentially interested volunteers from a Tulane volunteer recruitment registry. We will also consider recruiting potentially eligible individuals who were screened or participated in other clinical studies in our clinic and have expressed interest in being contacted about future research projects. Potential participants referred by their physician or community partners will have a pre-screening telephone interview to confirm potential eligibility. Those who are still interested and potentially eligible for the study will be scheduled for a screening visit.

7.B. Randomization

Once eligibility has been confirmed, the study coordinator will ensure that all key variables have been entered into the trial-specific database. Additionally, the study coordinator will ensure any outliers have been recognized and reconciled, and will ensure receipt of informed consent prior to enrolling the volunteer in the study as a trial participant. Eligible participants will be *randomly assigned to the behavioral intervention or the usual diet group in a 1:1 allocation ratio*. Randomization will be stratified by sex, *using a random block group size (4, 6) to maintain balance among groups over time*. A statistician from the Methodology/Biostatistics Unit within the COBRE Clinical Research Core, who is not directly involved in the study and is not located at the clinic, will conduct the randomization. After ascertaining eligibility, clinical personnel will telephone the Methodology/Biostatistics Unit to obtain a randomization assignment. All

randomization assignments will be created by a computer program and only accessible to the Staff at the Methodology/Biostatistics Unit. Once randomization assignment has been made, trial participants will be considered to be officially randomized into the study and every effort will be made to obtain complete follow-up information.

7.C. Masking

Clinical and laboratory staff members who assess outcomes will be blinded to the intervention assignment of participants. As this is a dietary intervention trial, study participants, and study staff members who conduct the intervention cannot be blinded to the intervention assignment. Participants will be instructed to avoid sharing knowledge of their treatment assignment with data collection staff members. We will have intervention visits and data collection study visits on different days and in separate locations. Data collectors will also use verbal cues and visual props to remind participants not to disclose treatment assignment. Investigators will be blinded to intervention assignment.

7.D. Safety Considerations

Participants with HbA1c >7.9% will be referred to a clinician for follow-up[98], though we will continue to collect data on these participants.

8. Dietary Intervention Program

8.A. Low-carbohydrate dietary intervention group

Participants randomized to the low-carbohydrate intervention group will receive counseling aimed at reducing carbohydrate consumption. Phase I (*Go Low*) will occur for the first 3 months, with a goal of <40 g digestible carbohydrates per day. Key recommended foods will include: non-starchy vegetables, fish, poultry, lean meat, eggs, olive oil and other plant-based unsaturated oils, unsweetened/unsalted nuts and seeds, nut butters, and avocados. We will recommend moderate consumption of cheese and unsweetened Greek yogurt and low-carbohydrate milk. During Phase I, we will recommend limiting or avoiding other dairy, fruits, legumes, beans, and grains. Phase II (*Keep it Low*) will run from months 4 through 6. The net carbohydrate goal for this phase will be <40 g to <60 g, with the objective to choose a target that is as low as possible (e.g., as close to <40 g as possible). At 3 months, we expect different participants will have different levels of intake, with some consuming <40 g of net carbohydrates and others consuming levels higher than 60 g net carbohydrates per day. We have designed the intervention to accommodate various scenarios, with the goal of achieving as low of an intake as possible. For participants who consume >60 g per day, we will recommend they aim to either reduce or maintain carbohydrate intake.

At a brief check-in at 3-months, if participants are satisfied with their current intake, we will instruct them to stay at this intake level (though will recommend they try to reduce intake, if possible, if they are eating >60 net grams of carbohydrates or have not yet reached the study goal of <40 net grams of carbohydrates). If they prefer to make modifications, we will provide guidance on how to modify carbohydrate intake (either decrease or increase). If they choose to add back carbohydrates, we will recommend that participants add 5 grams of carbohydrates for two weeks at a time (e.g., from 40 to 45 g for 2 weeks), for a maximum total of <60 g net carbohydrates. If they choose to further reduce carbohydrate intake, we will provide guidance on this as well. As they modify carbohydrate intake, we will have them monitor their satisfaction with their diet and energy levels. If adding back carbohydrates, we will recommend participants

add back in foods like fruit (preferably berries), legumes, beans, and dairy, while still aiming to keep total carbohydrate intake as low as possible (e.g., as close to <40 net grams as able).

Materials: We will develop a handbook that will be given to participants. This will contain diet guidelines, recipes, meal planners, shopping lists, and guides for counting carbohydrates and reading nutrition labels.

Meals: Participants will select their own menus and prepare or buy their meals according to the guidelines. We will provide participants with supplemental low-carbohydrate foods, such as nuts, olive oil, and other products, to help facilitate their compliance.

Counseling: Sessions and materials will focus on instructing participants to reduce digestible carbohydrate consumption by increasing consumption of protein and fat, with a particular emphasis on increased consumption of heart-healthy unsaturated fats. After randomization, participants will begin the 3-month *Go Low* phase consisting of individual counseling sessions each week for the first four weeks, followed by 4 group sessions held every other week with phone follow-ups occurring in between group sessions. During the *Keep it Low* phase, months 4 through 6, participants will attend 3 monthly group sessions and have 3 telephone follow-ups. There will be a total of 11 in-person sessions and 7 telephone follow-ups over the full study period. Sessions will be approximately one hour and phone calls will be no longer than thirty minutes. These sessions will consist of diet instruction and supportive counseling, addressing results from 24-hour dietary recalls, teaching self-monitoring and individual diet goal changes, and provide suggestions for replacing usual foods containing carbohydrates. Study materials will be modified from materials used in the successful MACRO study. The JFI will work closely with Drs. He and Bazzano to develop the intervention based on the MACRO study.
Carbohydrate goal: In the MACRO study, carried out among individuals from the same study region, a behavioral intervention aimed at reducing digestible carbohydrate intake to <40 g/day led to reductions in weight and improvements in cardiovascular risk factors among individuals with obesity.[30] Additionally, restriction of carbohydrate intake to <50g/day is sufficient to induce ketosis.[99] Our rationale for having a multi-phased intervention is to allow participants to see how they feel on the more restrictive diet, and see how they respond to small increases in carbohydrate consumption. A similar technique was applied in the DIETFITS published study.[100] The higher target of 60 net grams may be more sustainable long-term and also allows for consumption of healthy higher-carbohydrate foods, such as berries, legumes, beans, and limited whole grains.

8.B. Usual Diet Group

We will provide participants randomized to the usual diet group written information with standard dietary advice. Participants randomized to the usual diet group will not receive ongoing dietary recommendations. Moreover, they will continue to receive clinical care recommendations from their physician(s). To encourage continued participation in the study and minimize the difference in participant contact with study staff, participants will be offered optional monthly educational sessions on topics unrelated to the study intervention, such as retirement planning or health insurance options.

In another effort to encourage continued participation, patients randomized to the usual diet group will be offered a copy of all available intervention materials, as well as an optional one-hour counseling session with the interventionist following their final study visit.

8.C. Physical Activity

At baseline, both groups will receive written information in study materials with standard physical activity recommendations.

8.D. Dietary Adherence

Strategies for measuring compliance: For the low-carbohydrate diet, adherence will be assessed through measurement of urinary ketones at 3-month visit and 6-month TV. Study staff will make all reasonable effort to maintain adherence of participants to the visit schedule and diet intervention.

Strategies for improving compliance:

- At the screening visit, we will ask participants who does most of the cooking in their households and how often they eat outside of the home. Results from the screening visit will help to select a group of cooperative study participants.
- Visits will be scheduled at the participants' convenience and to minimize waiting time and trips to the clinic; reminder calls, texts or emails (chosen based on participant preference) will also be made prior to clinic visits.
- We will hire enthusiastic staff who have good interpersonal skills.
- Participants will receive $40 gift cards for use at food stores or retailers when they complete each study visit, for a maximum of $120 over the study period.
- Participants will receive $25 gift cards when they return the stool specimens, for a maximum of $50 over the study period.
- Participants will be offered small gifts (such as pens or magnets with study logo) to encourage compliance and adherence to the study protocol.
- Participants will be provided with recipes, shopping lists, and food samples for the intervention diet.

9. Safety of Dietary Intervention

On the low-carbohydrate diet, participants will be encouraged to primarily focus on replacing carbohydrates (particularly highly refined carbohydrates) with foods such as non-starchy vegetables, olive oil and other plant-based unsaturated oils, nuts and seeds, avocados, eggs, seafood, tofu, poultry, and lean meat. Consumption of more unsaturated fats, protein, and fiber will be recommended. Participants will not be encouraged to increase consumption of saturated fats and will be advised to avoid consumption of trans fats.

In animal studies, a high-protein diet has decreased renal function[101] In the MACRO study, on average, participants tended to increase consumption of fats, rather than protein.[30] The safety and tolerability of low-carbohydrate diets have previously been assessed in many studies, including MACRO.[30] A number of studies, including some in individuals with T2D, have found no clinically significant increases in estimated glomerular filtration rates,[33,38–40] microalbuminuria,[102] albumin excretion rate,[33,38] urinary albumin,[38] serum creatinine,[38,40,102–107] albumin,[40] or uric acid levels.[103–106] A small study of a very low-carbohydrate diet found significant increase in uric acid excretion, but no significant change in 24-hour urine creatinine clearance.[106]

Study staff will carefully monitor participants' BP, and HbA1c more often than what would occur in routine healthcare visits. A Data Safety and Monitoring Board (DSMB) consisting of an independent group of experts in areas such as biostatistics, nutrition medicine, general internal medicine, and endocrinology has been formed. The board will regularly review trial progress, including unblinded interim results, and can recommend that the trial prematurely end if participants are at risk or if it becomes clear that the trial will not be able to yield statistically significant results.

10. Data Collection

10.A. Study visits

After the initial pre-screening interview, in which data on medications and medical conditions will be collected, there will be one screening visit, one randomization visit, a follow-up study visit at 3 months and a termination visit (TV) at 6 months. ***Table 3*** summarizes study procedures and data collected at each study visit.

Table 3. Study visit schedule and data collection

	**Pre-screen**	**SV**	**RZ**	**3 month**	**6 month TV**
Pre-screen eligibility	X				
Informed consent		X			
Compliance form		X			
Eligibility confirmation			X		
Randomization			X		
Demographics		X			
Medical history questionnaire			X	X	X
Medications		X	X	X	X
Lifestyle (alcohol, cigarette smoking)			X	X	X
Physical activity			X	X	X
24-hour diet recall*			X	X	X
3 BP measurements		X	X	X	X
Body weight			X	X	X
Height			X		
Waist circumference			X	X	X
Dipstick ketone test			X	X	X
Blood samples		X	X	X	X
Stool specimen			X		X
Side-effects assessment			X	X	X

*Two 24-hour dietary recalls will be collected; one should cover a weekend and the other a weekday.

10.A.1. Screening/baseline visits:

Participants will be pre-screened by phone or in-person by study staff to confirm eligibility for the Screening Visit. An approved questionnaire will be used to ensure the patient is eligible to attend the in-clinic screening visit, and the patient will be scheduled thereafter.

At the screening visit (SV), participants will provide informed consent for the screening and main study. Study staff will schedule the SV and randomization (RZ) visit at least 2 days apart. If a participant is eligible based on the screening visit, he or she should be scheduled for the RZ once a sufficient group of eligible individuals has been recruited. Participants should be scheduled for the RZ at least 2 days and no more than two weeks after the screening visit.

At the screening visit, we will collect information on inclusion/exclusion criteria, medication use, and demographics. At this visit, we will also have participants complete a compliance form, to assess their ability to complete the study and attend all intervention and study visits. We will also measure blood pressure, and take blood samples. Women will be asked if they are postmenopausal or if they had surgical sterilization (such as a tubal ligation). If not, they will be tested for pregnancy through a spot urine test; pregnant women will be excluded from the study. Blood samples will be collected for measurement of HbA1c, and, if not available from medical records in the past 3 months, serum creatinine and hemoglobin. These blood samples will be used for excluding individuals with HbA1c <6.0 or >6.9, eGFR ≤45 mL/min/1.73 m^2, or hemoglobin <11 mg/dL.

Before the start of the intervention, participants will complete two 24-hour dietary recalls. At least one (and potentially both) 24-h recalls will be collected by phone. At the RZ, participants will learn about the randomization process. After requirements for eligibility have been satisfied, participants will formally be enrolled into the study and randomized to either the active intervention or control group. We will collect fasting blood samples for later measurement of glucose, insulin and lipids and other potential future measurements. At this visit, we will measure blood pressure, body weight, height, and waist circumference. We will collect information on medical history, lifestyle risk factors, and physical activity. A spot urine sample will be collected to measure ketones and participants will be asked to collect a stool specimen for potential use in future microbiome studies.

10.A.2. Follow-up visits and final visit: A follow-up visit will occur at 3 months after randomization. A final visit will occur at 6-months post-randomization. These visits should be scheduled as close as possible to the 3-, or 6-months after randomization. At each visit, study staff will conduct dietary recalls and anthropometric measures, take 3 BP measurements, collect a fasting blood sample to measure HbA1c. At least one (and potentially both) 24-h recalls will be collected by phone. We will also collect samples for later measurement of glucose, insulin, lipid profile, and other potential future measurements. A spot urine sample will be collected to measure ketones. At the 6-month TV, participants will be asked to collect a stool specimen for potential use in future microbiome studies. At study termination, we will mail participants and/or their physicians a summary of trial results and a record of participant BP, anthropometric measures, and laboratory data from the trial period. Throughout the study period, we will provide participants with their blood pressure and weight. We will request that, if possible, a participant who is prescribed a diabetes medication during the course of the study comes into the clinic for an additional separate supplemental visit for a fasting blood sample collection prior to starting the medication.

10.B Study measurements

Measurements obtained for this study will be based on those used in other clinical trials and epidemiologic studies. We are familiar with strengths and limitations of such measurements and the importance of staff training and certification and use of stringent quality control procedures.

10.B.1. Questionnaires: Study staff will administer a *medical history questionnaire* at the RZ, 3-month visit and 6-month TV to assess medical conditions that would necessitate changes in therapy or medical management beyond this study's scope. At the SV, *demographic factors* will be collected. At the SV, RZ, 3-month and 6-month TV, we will also administer *medication tracking questionnaires*. At RZ, 3-month, and 6-month TV, we will collect health behavior and physical activity data.

10.B.2. Dietary measurements: Two 24-hour dietary recalls will be obtained from participants at baseline. At or close to both the 3-month and 6-month TV, two more 24-hour dietary recalls

will be collected. The second sets of two recalls will be collected as closely as possible to 3- or 6-month dates after randomization, with a goal of being within 2 weeks of the respective follow-up period. For each time point, one recall should reflect week day consumption, while the other should reflect weekend day consumption and the two recalls should be at least 2 days apart from each other. We will use computer software (Nutrition Data System for Research [NDS-R]) and food models used in previous and ongoing studies. Recalls will be conducted by a study staff member who has been certified in the use of NDS-R. Food composition tables of the NDS-R will be used to calculate dietary nutrient intakes. The baseline dietary assessment will be done to characterize individual dietary intake of carbohydrates and other macronutrients. During the intervention, repeated measurements will be used to monitor short-term changes in mean dietary nutrient intake between the intervention and usual diet groups.

10.B.3. Assessment and management of adverse effects: At RZ, 3-month visit, and 6-month TV, we will administer *symptom questionnaires*, which will consist of a checklist to identify participant complaints. Prior studies of low-carbohydrate dietary interventions suggest that participants may experience a range of adverse effects, including constipation, halitosis, fatigue, headache, thirst, polyuria, and nausea. Severe adverse events will be promptly reported to the DSMB. Management of adverse effects will be based on the protection and safety of the participant. If severe adverse effects are reported by a participant, carbohydrate intake will be examined and altered in order to mitigate the adverse effect. In any extreme cases, participants will be instructed to stop the intervention. As this is a behavioral intervention, adverse effects should be rare.

10.B.4. BP and anthropometric measures: BP will be measured 3 times at the screening visit and at each follow-up visit. To minimize random error, averages of the measurements at the screening and randomization visits will be taken. BP will be measured by a standard automated blood pressure measurement device (OMRON HEM-907 XL Professional Digital Blood Pressure Monitor) and performed by experienced staff, trained and certified in BP measurement. Prior to BP measurements, participants will be asked to rest quietly in a seated position with their feet supported for at least 5 minutes; staff members will use an appropriate cuff size over a bare arm. Trained and certified staff will measure weight using a calibrated balance beam scale at the screening visits, and follow-up visits. Weight and height will be measured at the RZ, and weight will also be measured at the subsequent visits.

10.B.5. Blood samples: For SV, participants will not need to fast. For RZ, 3-month visit, and 6-month TV, participants will be instructed to fast for 12 hours prior to blood sample collection. At SV, blood samples will be collected to measure HbA1c, hemoglobin, and serum creatinine. At RZ, fasting blood samples will be collected and stored as serum/plasma, whole blood aliquots and buffy coats at -80°C for future analyses, including measurement of glucose, insulin and lipids, and potential future metabolomics or genetic analyses. At 3-month visit and 6-month TV, we will collect fasting blood samples to measure HbA1c and will also store additional serum/plasma, whole blood aliquots and buffy coats at -80°C for future analyses, including measurement of glucose, insulin and lipids, and potential future metabolomics or genetic analyses. Details of the blood sample collection are shown in ***Table 4***. HOMA-IR will be calculated from fasting glucose and insulin.

Table 4. Study visit schedule for specimen collection

	SV	RZ	3 month	6 month TV
HbA1c	X		X	X
Serum creatinine	X			
Hemoglobin	X			

Storage of aliquots and buffy coats (for measurement of glucose, insulin, lipids, other potential future measurements)		X	X	X
Urine sample for pregnancy	X			
Urine sample for ketones		X	X	X
Stool sample		X		X

10.B.6. Urine collection: Study staff will collect spot urine specimens at RZ, interim visit, and the final visit to measure ketones.

10.B.7. Stool Specimen Collection: Stool samples will be collected from all eligible and willing trial participants during the RZ and 6-month TV, following the protocol set forth by the Human Microbiome Project.[108,109] Participants will be given a stool collection kit, which includes the specimen collection container, a shipper for home collection, and a form to record the timing and pertinent information related to specimen collection. Participants will be instructed to return the kit to the clinic within two days of collection (in-person or via shipping), at which time stool samples are stored at -80°C until analyses. Kits and instructions for collection will be reviewed by study staff with the participant at the required visits.

11. Quality Assurance and Quality Control

Throughout the study, we will implement rigorous quality assurance (QA) and quality control (QC) measures. We describe some of these approaches here.

11.A. Manual of procedures

We will develop a manual of procedures (MOP) based on the MOP successfully used in the MACRO behavior change clinical trial. The MOP will provide instructions and describe procedures for staff training and certification, participant recruitment, intervention counseling, study form completion and procedures, data entry, safety measures (e.g., methods for emergency participant/primary care physician contact).

11.B. Training and certification

Trial personnel will all be required to participate in a training session before the start of any trial procedures or measurements. Training will include particular attention to core trial elements, such as recruitment, retention, intervention, completeness and quality of data acquisition (e.g., blinding procedures for the data collectors), and data entry. Separately, intervention staff will meet and receive detailed instruction on trial approaches to group and individual behavior change counseling and procedures that ensure data collectors are blinded to treatment assignment. Throughout the trial, periodic re-training/certification sessions will be conducted to ensure all trial-related procedures are being applied in a standard manner. Investigators will also monitor all aspects of study performance to ensure study goal achievement, timeliness and completeness of study visits, completeness and quality of data collection, intervention adherence, and adequacy of the procedures separating data collection from intervention. Study staff and investigators will pay particularly careful attention to the quality of all laboratory measurements, particularly key exposure, outcome, and intervention monitoring variables.

12. Sample Size and Statistical Power

The primary outcome will be the difference in the change of HbA1c between the intervention and usual diet groups from baseline to 6 months of follow-up. In the United States National Health and Nutrition Examination Survey, when sub-setting to the range of HbA1c of 6.0–6.9% among individuals who do not report use of diabetes medication, the cross-sectional standard deviation is 0.24% using data from 2000 through 2016 (n=4117; https://wwwn.cdc.gov/nchs/nhanes/). Studies with 6-month follow-up among individuals with HbA1c 5.7 to 6.4% or other characteristic(s) (e.g, elevated fasting glucose or metabolic syndrome) that put them at elevated risk of type 2 diabetes, the within-person correlation between baseline and follow-up HbA1c was estimated to be between 0.5 and 0.84.[110–113] As all participants in our study will be within a specific range of HbA1c at baseline, we expect this may lead to smaller within-person correlation of HbA1c over time than studies that do not restrict to a narrow range of HbA1c at baseline. Assuming a within-person correlation of 0.5 and an SD of 0.24% for change in HbA1c, we would need to have 90 individuals to have 85% power to detect an approximate difference in change of HbA1c of 0.15% between the intervention and usual diet groups.

In a follow-up analysis of the DPP, a 0.15% decrement in HbA1c at 6 months was associated with a 16% lower risk of incident T2D, adjusting for treatment arm, age, sex, race/ethnicity, household income, and baseline HbA1c.[115] In a pooled analysis from the Atherosclerosis Risk in Communities and Framingham Heart Studies, a 0.15% higher HbA1c was associated with an 16% higher risk of T2D over 20 years, a 30% higher risk over 8 years, and a 13% higher risk after 8 years, after adjusting for age, sex, fasting glucose, triglycerides, HDL, family history of T2D, SBP, and BMI.[116] Additionally, in EPIC-Norfork, a difference of HbA1c of 0.15% was associated with approximately a 5% higher risk of CHD among those free of CHD, stroke, diabetes, and with HbA1c <7.0%.[117]

Table 5. Minimum detectable differences

	SD	Detectable Difference
HbA1c, %	0.24	0.15
Fasting glucose, mg/dL	12.5	8.0
Fasting insulin, mU/mL	7.0	4.5
HOMA-IR	2.0	1.3
SBP (mmHg)	4.2	2.7
DBP (mmHg)	3.0	1.9
Total/HDL ratio	1.2	0.77
Body weight (kg)	6.6	4.2
Waist circumference (cm)	8.0	5.1
Estimated CVD risk (%)*	6.7	4.8

*10-y CVD risk, among estimated 85% or 76 participants free of CVD

In the DPP study, HbA1c was 0.15% lower among those in the lifestyle intervention arm than in the control arm at 6-months follow-up; HbA1c at study commencement was 5.9% and levels increased in the control arm and decreased in the behavioral intervention arm.[22] In a small behavioral intervention study (n=34) of a very low-carbohydrate ketogenic diet compared with a moderate carbohydrate, calorie-restricted, low-fat diet among individuals with type 2 diabetes or prediabetes (HbA1c >6.0%) and elevated body weight (BMI >25), mean HbA1c decreased from 6.6% to 6.1% in the very low-carbohydrate group and from 6.9% to 6.7% in the moderate carbohydrate group over a 12-month period; additionally, the very low-carbohydrate group had greater reductions in glucose-lowering medications.[37]

Detectable differences are listed for HbA1c and secondary outcomes in ***Table 5***, based on data from prior studies.[30,53,56,118] These were calculated for unpaired t-tests, power of 85%, two-sided alpha of 0.05, and assuming 80% follow-up of 112 participants, which would yield an estimated 90 participants.

13. Data Management and Analysis

13.A. Data management

Personnel will double-enter all study data. Prior to entering data, the study coordinator will conduct quality control on all study forms, study measures, and laboratory data. Data will be cleaned and logically checked.

13.B. Data analysis plan

The data will be analyzed using *intention to treat*; this involves comparing the primary and secondary outcomes between participants based on randomization assignment.

13.B.1. Exploratory data analysis: We will express group data for variables with a normal distribution as mean ± standard deviation and for variables not normally distributed as median and interquartile ranges (or will log-transform the variables to obtain a normal distribution). We will compare means and percentages of baseline variables across the intervention and usual diet group to assess similarity of these groups at randomization.

13.B.2. Primary, secondary, and exploratory outcomes: We will test the primary study hypothesis that there is a greater reduction in HbA1c from baseline to 6 months in the intervention group than in the usual diet group. To do this, we will use a mixed-effects linear regression model, to test the null hypothesis that there is no difference in change between baseline and 6-month HbA1c for the intervention group compared with the usual diet group (Specific Aim 1). To do this, we will use the following model:

$$Y_{i,j}|T = b_0 + b_1G_i + b_2T + b_3G_iT_6 \qquad + {}_{0i,j} + {}_{i,j,}$$

where $Y_{i,j,k}$ is HbA1c for the jth participant (P) nested in the ith intervention group (G) at time T_k (where T_k=0 for baseline, 1 for 3 months, and 2 for 6 months). This model includes a random intercept ($a_{0i,j}$) and an error term $e_{i,j,k}$. The interaction term ($b_3G_iT_{6\text{-months}}$) allows for an interaction between group and $T_{6\text{-months}}$, which is equal to 0 for baseline and 3-months and 1 for 6-months. Rejection of the null hypothesis ($b_1+2*b_3+b_4=0$) indicates there is a significant difference in change of HbA1c from baseline to 6 months between the intervention and control groups. In this model, participants are assumed to be random effects and intervention group, time, and the interaction terms are fixed effects.

We will use a similar approach to test the difference in the change between baseline and 6-month values of secondary outcomes (Specific Aim 2) and exploratory outcomes. In exploratory analyses, we will also test the difference in the change between baseline and 3-month values of outcomes (HbA1c, fasting glucose, systolic blood pressure, total-to-HDL cholesterol, body weight, insulin, HOMA-IR, diastolic blood pressure, waist circumference, and estimated CVD risk). We will also explore the use of mixed effects models to assess overall net change in HbA1c (by treating time as a linear variable) and other outcomes over the full study period. For each outcome, we will test modeling assumptions and will consider transformation of outcomes, if needed, to meet modeling assumptions. As the mixed-effects model assumes missing at random, we will also perform sensitivity analyses using Markov-chain Monte Carlo techniques to impute missing values of the outcomes, using covariates for this imputation and will compare results from this analysis with our primary findings.

15. Anticipated Challenges

Recruitment: Many clinical trials have challenges recruiting sufficient numbers of volunteers who meet the trial inclusion and exclusion criteria and demonstrate a high level of adherence to trial protocol requirements. Fortunately, the investigators are very experienced with clinical trial recruitment, and have several contingency options that can be used to meet unanticipated shortfall in recruitment.

Intervention: The investigators are aware of and understand challenges related to achieving and maintaining compliance with a behavioral intervention. The investigators have much experience and success in behavioral interventions, with recent success of a behavioral intervention aimed at reducing carbohydrate intake among participants from the New Orleans area. The JFI will collaborate with Drs. He and Bazzano to develop the intervention program based on the MACRO study and will assess potential barriers to implementation and intervention adherence (such as financial concerns and range of dietary patterns of study participants) during the planning and pilot phase of the study.

Adherence: Though adherence and follow-up are also main challenges related to clinical trials, the investigators have an outstanding track record for studies that have recruited participants for both clinical and epidemiology studies in the Greater New Orleans area. To encourage adherence, small gifts (such as pens or magnets with study logo) will be offered to all study participants.

16. Protection of Human Subjects

This Human Subjects Research meets the definition of a clinical trial. All investigators will be required to complete the CITI (Collaborative Institutional Training Initiative) program in the ethical use of human participants in research.

16.A. Protection of human subjects from research risks

16.A.1. Risks to the subjects

Human subject involvement and characteristics: We expect to enroll 112 participants, with an equal male to female ratio. We expect to enroll approximately 30 participants who are African-Americans, and another 10 with mixed racial backgrounds, and a few Asian participants given the demographics of the greater New Orleans area. Overall, given the demographics of greater New Orleans, we estimate approximately 10% participants of Hispanic or Latino ethnicity, 50% of them with African-American racial background.

Inclusion criteria

- Men or women ages 40 to 70 years: aim to recruit 50% women
- Any race or ethnicity: aim to recruit 40% ethnic minorities (i.e., African American and Hispanic), close to population in the greater New Orleans area[94]
- HbA1c 6.0–6.9%
- Willing and able to provide informed consent

Exclusion criteria

- Diagnosed type 1 diabetes mellitus based on patient self-report
- Use of agents affecting glycemic control (medications for diabetes, oral glucocorticoids) within the past three months prior to enrollment based on patient self-report
- Medical condition in which low-carbohydrate diet may not be advised
  - Estimated glomerular filtration rate (eGFR) ≤45 mL/min/1.73 m^2, which is close to the 5th percentile of eGFR among non-diseased individuals of 70 years of age[95]
  - Self-report of liver disease due to hepatitis or alcohol; osteoporosis; untreated thyroid disease; gout; or cancer (other than non-melanoma skin cancer) requiring treatment in the past year, unless prognosis is excellent
- Factors that may affect HbA1c:

    - Hemoglobin <11 mg/dL (cutpoint for moderate-to-severe anemia, which could lead to falsely elevated or lowered HbA1c)[96]
    - Recent blood donation or blood transfusion (self-report, past 4 months)
    - Human Immunodeficiency Virus (self-report)[97]
- Allergies to nuts
- For women, current pregnancy, breastfeeding, or plans to become pregnant during the study
- Consumption of ≥21 alcoholic drinks per week or consumption of ≥6 drinks per occasion
- Current or planned residence making it difficult to meet trial requirements (due to distance from study site and/or challenges regularly traveling to site)
- Current participation in another lifestyle intervention trial or a pharmaceutical trial
- Participation of another household member in the study; employees or persons living with employees of the study
- Other concerns regarding ability to meet trial requirements, at the discretion of the principal investigator or study coordinator

Sources of materials: Medical information data will be collected from the participant and, after written authorization for the disclosure of protected health information, from participants' medical records. Blood and urine samples will be collected to measure HbA1c, glucose, insulin, lipids, creatinine, ketones, and test for pregnancy. Blood pressure, height, weight, waist circumference and other physical measurements will be obtained.

Potential Risks: Risks associated with venipuncture, the diet intervention, and collection of confidential medical information are the main risks associated with this study. There is a small risk of bruising and a rare chance of local infection associated with standard venipuncture to collect blood samples. The dietary intervention has minimal risks. Reduced carbohydrate consumption may lead to adverse effects, including constipation, halitosis, fatigue, headache, thirst, polyuria, and nausea. At RZ, 3- , and 6-month visits, we will administer *symptom questionnaires*, which will consist of a checklist to identify participant complaints. Adverse effects of the low-carbohydrate diet will be assessed at counseling sessions. Severe adverse events will be promptly reported to the DSMB. Management of adverse effects will be based on the protection and safety of the participant. If severe adverse effects are reported by a participant, carbohydrate intake will be examined and altered in order to mitigate the adverse effect. In any extreme cases, participants will be instructed to stop the intervention. Furthermore, there is a potential risk of unauthorized disclosure of medical information. This risk will be minimized by using a strict protocol for data processing and by the appropriate training of all study personnel. Protection against risks is described in more detail below.

16.A.2. Adequacy of protection against risks

Recruitment and Informed Consent: The proposed study will be conducted following strict guidelines for the protection of the rights of human volunteers. The informed consent document will be signed by all participants at the screening/baseline visit. The informed consent form will be developed in the first year of the study and approved by the IRB. The consent form will clearly state the purpose, eligibility criteria, and protocol of the study, the potential benefits and risks of participation, and the subject's right to refuse to participate or withdraw. Prior to signing the informed consent, research staff will review the details of the consent form orally with the participant, and answer any questions that the participant has concerning participation in the

study. In the case of potential participants with low or no literacy, the informed consent form will be read to the participant in its entirety and any questions will be answered prior to the participant signing or marking the form. Specifically, the following must be accomplished during the informed consent process:

- The participant must be informed that participation in the study is **voluntary** and that refusal to participate will involve no penalty or loss of benefits.
- The participant must be informed of the **purpose** of the study and that it involves **research**.
- The participant must be informed of any **alternative procedures,** if applicable.
- The participant must be informed of any reasonably foreseeable **risks**.
- The participant must be informed of any **benefits** from the research.
- An outline of safeguards to protect participant **confidentiality** will be included, as well as an indication of the participant's right to withdraw without penalty. This should be balanced with a discussion of the effect that withdrawals will have on the study, and the responsibility a participant has, within limits, to continue in the study if he or she decides to enroll.
- The participant must be informed of his or her right to have **questions answered** at any time and **whom to contact** for answers or in the event of research-related injury.
- The participant must be informed as to whether or not **compensation** is offered for participation in the study and/or in the event of a medical injury.
- The participant must be informed that he/she will be notified of any significant **changes** in the protocol that might affect their willingness to continue in the study.

Written authorization for the disclosure of protected health information will be obtained during the consent process on a separate form (HIPPA authorization), if applicable.

Protection against Risk: The protocol and informed consent will be approved by the Institutional Review Boards at Tulane University Health Sciences Center and any other participating institution for recruitment. The study participants will be given contact information for the Study Coordinator and Study PI to answer questions or to report complaints. Patients can withdraw from the study at any time without any effect on their medical care. Standard techniques will be used by trained, certified phlebotomists or study nurses for blood sample collection at the study clinics where emergency medical equipment is available. Confidentiality of all data collected during the study will be maintained through the use of unique, encrypted study identification (ID numbers, rather than patient names, in the study database). These encrypted ID numbers will not be mathematical derivation of the subject's medical record number or any other patient identifier. No information will be disclosed in an individually identifiable form in any type of presentation or publication. There will be password-required access to the computerized file linking the study numbers to patient identifiers at the study clinics. Password-access will be restricted to pertinent research staff only. All study data will be kept in locked file cabinets in locked rooms, accessible only by study staff with permission. All data transmissions will use HIPPA-compliant password protected system and will be secured through a virtual private network.

16.A.3. Potential benefits of the proposed research to human subjects and others

Study participants may have improved glycemic outcomes, blood pressure, lipid levels, may lose weight and reduce waist circumference, and may have improvement in other risk factors for CVD and development and progression of T2D. Participants will receive multiple blood pressure, weight, waist circumference, and blood tests. If vital signs or lab values are clinically abnormal, participants will be referred to their primary care physician, or if they do not have a

primary care physician, one will be assigned through the Tulane University Community Health Center, which is a part of the core clinical facilities. At the end of the study, we will provide the participants' primary care physician with a copy of all the blood pressure, weight, and laboratory results.

16.A.4. Importance of knowledge to be gained

Information from this study may contribute to reducing the onset of T2D and the burden of T2D and cardiovascular disease in Louisiana and the general US population. The study has potential for public health impact; results from this study may provide evidence in support of promoting alternative dietary styles (than the most commonly used low-fat diet) among individuals with high T2D risk or with T2D.

16.A.5. Remuneration

For screening visit 2, the two follow-up visits, and the final visits, participants will receive $20 gift cards for use at food stores or retailers (after completing each study visit), for a maximum of $60 over the study period. Participants who return a stool sample will receive $25 gift cards for each stool sample that they return, for a total of $50 for two stool samples. Participants will also receive free parking for each intervention and study visit.

16.B. Data safety and monitoring plan

A DSMB, which consists of 3 experts who are not otherwise affiliated with the COBRE program, has been formed. Study investigators for this study will prepare accurate and timely data tables and reports for the DSMB meetings. The members of the DSMB will serve in an individual capacity and provide their expertise and recommendations. The primary responsibilities of the DSMB are: 1) periodically review and evaluate the accumulated study data for participant safety, study conduct and progress, and, when appropriate, efficacy, and 2) make recommendations to the NIH concerning the continuation, modification, or termination of the trial. The DSMB considers study-specific data, as well as relevant background knowledge, about the disease, test agent, and patient population under study.

The DSMB is responsible for defining its deliberative processes, including event triggers that would call for an unscheduled review, stopping guidelines, unmasking (unblinding) and voting procedures prior to initiating any data review. The DSMB is also responsible for maintaining the confidentiality of its internal discussions and activities, as well as the contents of reports provided to it.

The DSMB will review each protocol for any major concerns prior to implementation. During the trial, the DSMB should review cumulative study data to evaluate safety, study conduct, and scientific validity and integrity of the trial. As part of this responsibility, DSMB members must be satisfied that the timeliness, completeness, and accuracy of the data submitted to them for review are sufficient for evaluation of the safety and welfare of study participants. The DSMB should also assess the performance of overall study operations and any other relevant issues, as necessary.

Items reviewed by the DSMB include:

- Interim/cumulative data for evidence of study-related adverse events;
- Interim/cumulative data for evidence of efficacy according to pre-established statistical guidelines, if appropriate;
- Data quality, completeness, and timeliness;

- Adequacy of compliance with goals for recruitment and retention, including those related to the participation of women and minorities;
- Adherence to the protocol;
- Factors that might affect the study outcome or compromise the confidentiality of the trial data (such as protocol violations, unmasking, etc.); and,
- Factors external to the study, such as scientific or therapeutic developments, that may impact participant safety or the ethics of the study.

The DSMB will conclude each review with their recommendations to the COBRE program and NIH/NIGMS as to whether the study should continue without change or be modified or terminated. Recommendations regarding modification of the design and conduct of the study could include:

- Modifications of the study protocol based upon review of the safety data;
- Suspension or early termination of the study or of one or more study arms because of serious concerns about subjects' safety, inadequate performance or rate of enrollment;
- Suspension or early termination of the study or of one or more study arms because study objectives have not been obtained according to pre-established statistical guidelines; and,
- Corrective actions regarding a study when the performance appears unsatisfactory or suspicious.

Confidentiality must always be maintained during all phases of DSMB review and deliberation. Usually, only voting members of the DSMB should have access to interim analyses of outcome data by treatment group. Exceptions may be made when the DSMB deems it appropriate. The reason and to whom the exceptions for access to interim analyses is granted will be documented in the Closed Session Report. DSMB members must maintain strict confidentiality concerning all privileged trial results ever provided to them. The DSMB should review data only by masked study group (such as X vs. Y rather than experimental vs. control) unless or until the DSMB determines that the identities of the groups are necessary for their decision-making. Whenever masked data are presented to the DSMB, the key to the group coding must be available for immediate unmasking.

The DSMB will meet twice per year. The junior faculty investigator will attend these meetings and will be responsible for preparing and presenting up-to-date statistical reports on the progress of their trials. These reports will include data on recruitment, randomization, adherence, as well as statistical tests and special analyses requested by the DSMB. The Administrative Core will be responsible for organizing the annual in-person meeting and semiannual conference calls. They will put together data and materials submitted by junior faculty investigators and deliver them to DSMB members. The COBRE investigators will meet with the DSMB in the Open Session and not be present for the Closed Session.

Bibliography and References Cited

1. The Emerging Risk Factors Collaboration. Diabetes mellitus, fasting blood glucose concentration, and risk of vascular disease: a collaborative meta-analysis of 102 prospective studies. *Lancet*. 2010;375(9733):2215-2222. doi:10.1016/S0140-6736(10)60484-9.
2. Flaxman SR, Bourne RRA, Resnikoff S, et al. Global causes of blindness and distance vision impairment 1990-2020: a systematic review and meta-analysis. *Lancet Glob Heal*. October 2017. doi:10.1016/S2214-109X(17)30393-5.
3. United States Renal Data System. *2016 USRDS Annual Data Report: Epidemiology of Kidney Disease in the United States*. Bethesda, MD: National Institutes of Health, National Institute of Diabetes and Digestive and Kidney Diseases; 2016. https://www.usrds.org/2016/view/Default.aspx.
4. Narres M, Kvitkina T, Claessen H, et al. Incidence of lower extremity amputations in the diabetic compared with the non-diabetic population: A systematic review. *PLoS One*. 2017;12(8):e0182081. doi:10.1371/journal.pone.0182081.
5. Centers for Disease Control and Prevention. *National Diabetes Statistics Report, 2017*. Atlanta, GA: Centers for Disease Control and Prevention, U.S. Dept of Health and Human Services; 2017.
6. Centers for Disease Control and Prevention. *Long-Term Trends in Diabetes*. Atlanta, GA: Centers for Disease Control and Prevention, U.S. Dept of Health and Human Services; 2017.
7. NCD Risk Factor Collaboration (NCD-RisC). Worldwide trends in diabetes since 1980: A pooled analysis of 751 population-based studies with 4.4 million participants. *Lancet*. 2016;387(10027):1513-1530. doi:10.1016/S0140-6736(16)00618-8.
8. American Diabetes Association. Economic costs of diabetes in the U.S. in 2012. *Diabetes Care*. 2013;36(4):1033-1046. doi:10.2337/dc12-2625.
9. Zhuo X, Zhang P, Barker L, Albright A, Thompson TJ, Gregg E. The lifetime cost of diabetes and its implications for diabetes prevention. *Diabetes Care*. 2014;37(9):2557-2564. doi:10.2337/dc13-2484.
10. WHO, International Diabetes Foundation. *Definition and Diagnosis of Diabetes Mellitus and Intermediate Hyperglycaemia: Report of a WHO/IDF Consultation*. Geneva: Geneva: World Health Organization; 2006.
11. The International Expert Committee. International Expert Committee Report on the Role of the A1C Assay in the Diagnosis of Diabetes. *Diabetes Care*. 2009;32(7):1327-1334. doi:10.2337/dc09-9033.
12. American Diabetes Association. Standards of Medical Care in Diabetes - 2017. *Diabetes Care*. 2017;40(Supplement 1):S33-S43. doi:10.2337/dc16-S003.
13. Gerstein HC, Santaguida P, Raina P, et al. Annual incidence and relative risk of diabetes in people with various categories of dysglycemia: A systematic overview and meta-analysis of prospective studies. *Diabetes Res Clin Pract*. 2007;78(3):305-312. doi:10.1016/j.diabres.2007.05.004.
14. Huang Y, Cai X, Mai W, Li M, Hu Y. Association between prediabetes and risk of cardiovascular disease and all cause mortality: systematic review and meta-analysis. *BMJ*. 2016;355:i5953. doi:10.1136/bmj.i5953.
15. Tabák AG, Herder C, Rathmann W, Brunner EJ, Kivimäki M. Prediabetes: A high-risk state for diabetes development. *Lancet*. 2012;379(9833):2279-2290. doi:10.1016/S0140-

6736(12)60283-9.
16. Diabetes Prevention Program Research Group. 10-year follow-up of diabetes incidence and weight loss in the Diabetes Prevention Program Outcomes Study. *Lancet*. 2009;374(9702):1677-1686. doi:10.1016/S0140-6736(09)61457-4.
17. The DREAM (Diabetes REduction Assessment with ramipril and rosiglitazone Medication) Trial Investigators. Effect of rosiglitazone on the frequency of diabetes in patients with impaired glucose tolerance or impaired fasting glucose: a randomised controlled trial. *Lancet*. 2006;368(9541):1096-1105. doi:10.1016/S0140-6736(06)69420-8.
18. Perreault L, Pan Q, Mather KJ, Watson KE, Hamman RF, Kahn SE. Effect of regression from prediabetes to normal glucose regulation on long-term reduction in diabetes risk: Results from the Diabetes Prevention Program Outcomes Study. *Lancet*. 2012;379(9833):2243-2251. doi:10.1016/S0140-6736(12)60525-X.
19. Perreault L, Temprosa M, Mather KJ, et al. Regression from prediabetes to normal glucose regulation is associated with reduction in cardiovascular risk: Results from the diabetes prevention program outcomes study. *Diabetes Care*. 2014;37(9):2622-2631. doi:10.2337/dc14-0656.
20. Popkin BM. Nutrition Transition and the Global Diabetes Epidemic. *Curr Diab Rep*. 2015;15(9). doi:10.1007/s11892-015-0631-4.
21. Vazquez G, Duval S, Jacobs DRJ, Silventoinen K. Comparison of body mass index, waist circumference, and waist/hip ratio in predicting incident diabetes: a meta-analysis. *Epidemiol Rev*. 2007;29:115-128. doi:10.1093/epirev/mxm008.
22. Knowler WC, Barrett-Connor E, Fowler SE, et al. Reduction in the incidence of type 2 diabetes with lifestyle intervention or metformin. *N Engl J Med*. 2002;346(6):393-403. doi:10.1056/NEJMoa012512.
23. Pan XR, Li GW, Hu YH, et al. Effects of diet and exercise in preventing NIDDM in people with impaired glucose tolerance: The Da Qing IGT and diabetes study. *Diabetes Care*. 1997;20(4):537-544. doi:10.2337/diacare.20.4.537.
24. Tuomilehto J, Indstrom J, Eriksson J, Valle T, Hamalainine E, Uusitupa M. Prevention of Type 2 Diabetes Mellitus By Changes in Lifestyle Among Subjects With Impaired Glucose Tolerance. *N Engl J Med*. 2001;344(18):1343-1350. doi:10.1056/NEJM200105033441801.
25. Ramachandran A, Snehalatha C, Mary S, Mukesh B, Bhaskar AD, Vijay V. The Indian Diabetes Prevention Programme shows that lifestyle modification and metformin prevent type 2 diabetes in Asian Indian subjects with impaired glucose tolerance (IDPP-1). *Diabetologia*. 2006;49(2):289-297. doi:10.1007/s00125-005-0097-z.
26. The Diabetes Preventio Program Research Group. The Diabetes Prevention Program. Design and methods for a clinical trial in the prevention of type 2 diabetes. *Diabetes Care*. 1999;22(4):623-634. doi:10.2337/diacare.22.4.623 10.2337/diabetes.48.4.699.
27. Evert AB, Boucher JL, Cypress M, et al. Nutrition therapy recommendations for the management of adults with diabetes. *Diabetes Care*. 2013;36(11):3821-3842. doi:10.2337/dc13-2042.
28. Hu T, Mills KT, Yao L, et al. Effects of low-carbohydrate diets versus low-fat diets on metabolic risk factors: A meta-analysis of randomized controlled clinical trials. *Am J Epidemiol*. 2012;176(SUPPL. 7). doi:10.1093/aje/kws264.
29. Sackner-Bernstein J, Kanter D, Kaul S. Dietary Intervention for Overweight and Obese Adults: Comparison of Low-Carbohydrate and Low-Fat Diets. A Meta-Analysis. *PLoS*

One. 2015;10(10):e0139817. doi:10.1371/journal.pone.0139817.

30. Bazzano LA, Hu T, Reynolds K, et al. Effects of low-carbohydrate and low-fat diets: A randomized trial. *Ann Intern Med*. 2014;161(5):309-318. doi:10.7326/M14-0180.
31. Sawyer L, Gale EAM. Diet, delusion and diabetes. *Diabetologia*. 2009;52(1):1-7. doi:10.1007/s00125-008-1203-9.
32. Snorgaard O, Poulsen GM, Andersen HK, Astrup A. Systematic review and meta-analysis of dietary carbohydrate restriction in patients with type 2 diabetes. *BMJ Open Diabetes Res Care*. 2017;5(1):e000354. doi:10.1136/bmjdrc-2016-000354.
33. Larsen RN, Mann NJ, Maclean E, Shaw JE. The effect of high-protein, low-carbohydrate diets in the treatment of type 2 diabetes: A 12 month randomised controlled trial. *Diabetologia*. 2011;54(4):731-740. doi:10.1007/s00125-010-2027-y.
34. Saslow LR, Kim S, Daubenmier JJ, et al. A randomized pilot trial of a moderate carbohydrate diet compared to a very low carbohydrate diet in overweight or obese individuals with type 2 diabetes mellitus or prediabetes. *PLoS One*. 2014;9(4). doi:10.1371/journal.pone.0091027.
35. Tay J, Luscombe-Marsh ND, Thompson CH, et al. A very low-carbohydrate, low-saturated fat diet for type 2 diabetes management: A randomized trial. *Diabetes Care*. 2014;37(11):2909-2918. doi:10.2337/dc14-0845.
36. Davis NJ, Tomuta N, Schechter C, et al. Comparative Study of the Effects of a 1-Year Dietary Intervention of a Low- Carbohydrate Diet Versus a Low-Fat Diet on Weight and Glycemic Control in Type 2. *Diabetes Care*. 2009;32(7):1147-1152. doi:10.2337/dc08-2108.Clinical.
37. Saslow LR, Daubenmier JJ, Moskowitz JT, et al. Twelve-month outcomes of a randomized trial of a moderate-carbohydrate versus very low-carbohydrate diet in overweight adults with type 2 diabetes mellitus or prediabetes. *Nutr Diabetes*. 2017;7(12):304. doi:10.1038/s41387-017-0006-9.
38. Tay J, Thompson CH, Luscombe-Marsh ND, et al. Effects of an energy-restricted low-carbohydrate, high unsaturated fat/low saturated fat diet versus a high carbohydrate, low fat diet in type 2 diabetes: a 2 year randomized clinical trial. *Diabetes, Obes Metab*. 2017:n/a-n/a. doi:10.1111/dom.13164.
39. Yamada Y, Uchida J, Izumi H, et al. A non-calorie-restricted low-carbohydrate diet is effective as an alternative therapy for patients with type 2 diabetes. *Intern Med*. 2014;53(1):13-19. doi:http://dx.doi.org/10.2169/internalmedicine.53.0861.
40. Mayer SB, Jeffreys AS, Olsen MK, Mcduffie JR, Feinglos MN, Yancy WS. Two diets with different haemoglobin A1c and antiglycaemic medication effects despite similar weight loss in type 2 diabetes. *Diabetes, Obes Metab*. 2014;16(1):90-93. doi:10.1111/dom.12191.
41. Maekawa S, Kawahara T, Nomura R, et al. Retrospective study on the efficacy of a low-carbohydrate diet for impaired glucose tolerance. *Diabetes Metab Syndr Obes*. 2014;7:195-201. doi:10.2147/DMSO.S62681.
42. Davis C, Bryan J, Hodgson J, Murphy K. Definition of the mediterranean diet: A literature review. *Nutrients*. 2015;7(11):9139-9153. doi:10.3390/nu7115459.
43. Sofi F, Macchi C, Abbate R, Gensini GF, Casini A. Mediterranean diet and health status: an updated meta-analysis and a proposal for a literature-based adherence score. *Public Health Nutr*. 2014;17(12):2769-2782. doi:10.1017/S1368980013003169.
44. Jannasch F, Kröger J, Schulze MB. Dietary Patterns and Type 2 Diabetes: A Systematic Literature Review and Meta-Analysis of Prospective Studies. *J Nutr*. 2017;147(6):1174-

1182. doi:10.3945/jn.116.242552.

45. Schwingshackl L, Missbach B, König J, Hoffmann G. Adherence to a Mediterranean diet and risk of diabetes: a systematic review and meta-analysis. *Public Health Nutr*. 2014;18(7):1292–1299. doi:10.1017/S1368980014001542.

46. de Lorgeril M, Salen P, Martin J-L, Monjaud I, Delaye J, Mamelle N. Mediterranean Diet, Traditional Risk Factors, and the Rate of Cardiovascular Complications After Myocardial Infarction : Final Report of the Lyon Diet Heart Study. *Circulation*. 1999;99(6):779-785. doi:10.1161/01.CIR.99.6.779.

47. Estruch R, Ros E, Salas-Salvadó J, et al. Primary Prevention of Cardiovascular Disease with a Mediterranean Diet. *N Engl J Med*. 2013;368(14):1279-1290. doi:10.1056/NEJMoa1200303.

48. Salas-Salvadó J, Bulló M, Estruch R, et al. Prevention of diabetes with mediterranean diets: A subgroup analysis of a randomized trial. *Ann Intern Med*. 2014;160(1):1-10. doi:10.7326/M13-1725.

49. Esposito K, Marfella R, Ciotola M, et al. Effect of a Mediterranean-Style Diet on Endothelial Dysfunction and Markers of Vascular Inflammation in the Metabolic Syndrome. *JAMA*. 2004;292(12):1440. doi:10.1001/jama.292.12.1440.

50. Babio N, Toledo E, Estruch R, et al. Mediterranean diets and metabolic syndrome status in the PREDIMED randomized trial. *CMAJ*. 2014;186(17):E649-E657. doi:10.1503/cmaj.140764.

51. Esposito K, Maiorino MI, Bellastella G, Chiodini P, Panagiotakos D, Giugliano D. A journey into a Mediterranean diet and type 2 diabetes: a systematic review with meta-analyses. *BMJ Open*. 2015;5(8):e008222. doi:10.1136/bmjopen-2015-008222.

52. Micha R, Michas G, Mozaffarian D. Unprocessed red and processed meats and risk of coronary artery disease and type 2 diabetes--an updated review of the evidence. *Curr Atheroscler Rep*. 2012;14(6):515-524. doi:10.1007/s11883-012-0282-8.

53. Esposito K, Maiorino MI, Ciotola M, et al. Effects of a Mediterranean-style diet on the need for antihyperglycemic drug therapy in patients with newly diagnosed type 2 diabetes: A randomized trial. *Ann Intern Med*. 2009;151(5):306-314. doi:10.1097/OGX.0b013e3181e5a159.

54. Esposito K, Maiorino MI, Petrizzo M, Bellastella G, Giugliano D. The effects of a Mediterranean diet on the need for diabetes drugs and remission of newly diagnosed type 2 diabetes: Follow-up of a randomized trial. *Diabetes Care*. 2014;37(7):1824-1830. doi:10.2337/dc13-2899.

55. Elhayany A, Lustman A, Abel R, Attal-Singer J, Vinker S. A low carbohydrate Mediterranean diet improves cardiovascular risk factors and diabetes control among overweight patients with type 2 diabetes mellitus: A 1-year prospective randomized intervention study. *Diabetes, Obes Metab*. 2010;12(3):204-209. doi:10.1111/j.1463-1326.2009.01151.x.

56. Shai I, Schwarzfuchs D, Henkin Y, et al. Weight Loss with a Low-Carbohydrate, Mediterranean, or Low-Fat Diet. *N Engl J Med*. 2008;359(3):229-241. doi:10.1056/NEJMoa0708681.

57. Schwarzfuchs D, Golan R, Shai I. Four-Year Follow-up after Two-Year Dietary Interventions. *N Engl J Med*. 2012;367(14):1373-1374. doi:10.1056/NEJMc1204792.

58. Pan A, Sun Q, Bernstein AM, et al. Red meat consumption and risk of type 2 diabetes: 3 Cohorts of US adults and an updated meta-analysis. *Am J Clin Nutr*. 2011;94(4):1088-1096. doi:10.3945/ajcn.111.018978.

59. Hu EA, Pan A, Malik V, Sun Q. White rice consumption and risk of type 2 diabetes: meta-analysis and systematic review. *BMJ*. 2012;344:e1454. doi:10.1136/bmj.e1454.
60. Wang M, Yu M, Fang L, Hu R-Y. Association between sugar-sweetened beverages and type 2 diabetes: A meta-analysis. *J Diabetes Investig*. 2015;6(3):360-366. doi:10.1111/jdi.12309.
61. Imamura F, O'Connor L, Ye Z, et al. Consumption of sugar sweetened beverages, artificially sweetened beverages, and fruit juice and incidence of type 2 diabetes: systematic review, meta-analysis, and estimation of population attributable fraction. *BMJ*. 2015:h3576. doi:10.1136/bmj.h3576.
62. Carter P, Gray LJ, Troughton J, Khunti K, Davies MJ. Fruit and vegetable intake and incidence of type 2 diabetes mellitus: systematic review and meta-analysis. *BMJ*. 2010;341:c4229. doi:10.1136/bmj.c4229.
63. Cooper AJ, Forouhi NG, Ye Z, et al. Fruit and vegetable intake and type 2 diabetes: EPIC-InterAct prospective study and meta-analysis. *Eur J Clin Nutr*. 2012;66(10):1082-1092. doi:10.1038/ejcn.2012.85.
64. Chen M, Sun Q, Giovannucci E, et al. Dairy consumption and risk of type 2 diabetes: 3 cohorts of US adults and an updated meta-analysis. *BMC Med*. 2014;12(1):215. doi:10.1186/s12916-014-0215-1.
65. Aune D, Norat T, Romundstad P, Vatten LJ. Whole grain and refined grain consumption and the risk of type 2 diabetes: A systematic review and dose-response meta-analysis of cohort studies. *Eur J Epidemiol*. 2013;28(11):845-858. doi:10.1007/s10654-013-9852-5.
66. Ding M, Bhupathiraju SN, Chen M, van Dam RM, Hu FB. Caffeinated and decaffeinated coffee consumption and risk of type 2 diabetes: a systematic review and a dose-response meta-analysis. *Diabetes Care*. 2014;37(2):569-586. doi:10.2337/dc13-1203.
67. Li X-H, Yu F-F, Zhou Y-H, He J. Association between alcohol consumption and the risk of incident type 2 diabetes: a systematic review and dose-response meta-analysis. *Am J Clin Nutr*. 2016;103(3):818-829. doi:10.3945/ajcn.115.114389.
68. Muraki I, Imamura F, Manson JE, et al. Fruit consumption and risk of type 2 diabetes: results from three prospective longitudinal cohort studies. *BMJ*. 2013;347(aug28 1):f5001-f5001. doi:10.1136/bmj.f5001.
69. Upadhyaya S, Banerjee G. Type 2 diabetes and gut microbiome: At the intersection of known and unknown. *Gut Microbes*. 2015;6(2):85-92. doi:10.1080/19490976.2015.1024918.
70. David LA, Maurice CF, Carmody RN, et al. Diet rapidly and reproducibly alters the human gut microbiome. *Nature*. 2014;505(7484):559-563. doi:10.1038/nature12820.
71. Duncan SH, Belenguer A, Holtrop G, Johnstone AM, Flint HJ, Lobley GE. Reduced dietary intake of carbohydrates by obese subjects results in decreased concentrations of butyrate and butyrate-producing bacteria in feces. *Appl Environ Microbiol*. 2007;73(4):1073-1078. doi:10.1128/AEM.02340-06.
72. Dorans KS, Mostofsky E, Levitan EB, Håkansson N, Wolk A, Mittleman MA. Alcohol and Incident Heart Failure among Middle-Aged and Elderly Men: Cohort of Swedish Men. *Circ Hear Fail*. 2015;8(3):422-427. doi:10.1161/CIRCHEARTFAILURE.114.001787.
73. Steinhaus DA, Mostofsky E, Levitan EB, et al. Chocolate intake and incidence of heart failure: Findings from the Cohort of Swedish Men. *Am Heart J*. 2017;183:18-23. doi:10.1016/j.ahj.2016.10.002.
74. Dorans KS, Massa J, Chitnis T, Ascherio A, Munger KL. Physical activity and the incidence of multiple sclerosis. *Neurology*. 2016;87(17):1770-1776.

doi:10.1212/WNL.0000000000003260.

75. Mills KT, Chen J, Yang W, et al. Sodium Excretion and the Risk of Cardiovascular Disease in Patients With Chronic Kidney Disease. *JAMA*. 2016;315(20):2200. doi:10.1001/jama.2016.4447.

76. Chen J, Gu D, Huang J, et al. Metabolic syndrome and salt sensitivity of blood pressure in non-diabetic people in China: a dietary intervention study. *Lancet*. 2009;373(9666):829-835. doi:10.1016/S0140-6736(09)60144-6.

77. Li C, He J, Chen J, et al. Genome-Wide Gene-Sodium Interaction Analyses on Blood Pressure: The Genetic Epidemiology Network of Salt-Sensitivity Study. *Hypertension*. 2016;68(2):348-355. doi:10.1161/HYPERTENSIONAHA.115.06765.

78. He J, Streiffer RH, Muntner P, Krousel-Wood MA, Whelton PK. Effect of dietary fiber intake on blood pressure: a randomized, double-blind, placebo-controlled trial. *J Hypertens*. 2004;22(1):73-80. doi:10.1097/01.hjh.0000098164.70956.54.

79. He J, Ogden LG, Vupputuri S, Bazzano LA, Loria C, Whelton PK. Dietary sodium intake and subsequent risk of cardiovascular disease in overweight adults. *JAMA*. 1999;282(21):2027-2034. doi:10.1001/jama.282.21.2027.

80. He J, Wofford MR, Reynolds K, et al. Effect of dietary protein supplementation on blood pressure a randomized, controlled trial. *Circulation*. 2011;124(5):589-595. doi:10.1161/CIRCULATIONAHA.110.009159.

81. Thompson AM, Zhang Y, Tong W, et al. Association of inflammation and endothelial dysfunction with metabolic syndrome, prediabetes and diabetes in adults from Inner Mongolia, China. *BMC Endocr Disord*. 2011;11(1):16. doi:10.1186/1472-6823-11-16.

82. Irazola V, Rubinstein A, Bazzano L, et al. Prevalence, awareness, treatment and control of diabetes and impaired fasting glucose in the Southern Cone of Latin America. *PLoS One*. 2017;12(9). doi:10.1371/journal.pone.0183953.

83. Bazzano LA, Li TY, Joshipura KJ, Hu FB. Intake of fruit, vegetables, and fruit juices and risk of diabetes in women. *Diabetes Care*. 2008;31(7):1311-1317. doi:10.2337/dc08-0080.

84. Bazzano LA, Serdula M, Liu S. Prevention of Type 2 Diabetes by Diet and Lifestyle Modification. *J Am Coll Nutr*. 2005;24(5):310-319. doi:10.1080/07315724.2005.10719479.

85. Pollock BD, Chen W, Harville EW, et al. Differential sex effects of systolic blood pressure and low-density lipoprotein cholesterol on type 2 diabetes: Life course data from the Bogalusa Heart Study. *Journal of Diabetes*. 2017.

86. Zhang T, Li Y, Zhang H, et al. Insulin-sensitive adiposity is associated with a relatively lower risk of diabetes than insulin-resistant adiposity: the Bogalusa Heart Study. *Endocrine*. 2016;54(1):93-100. doi:10.1007/s12020-016-0948-z.

87. Pollock BD, Hu T, Chen W, et al. Utility of existing diabetes risk prediction tools for young black and white adults: Evidence from the Bogalusa Heart Study. *J Diabetes Complications*. 2016;31(1):86-93. doi:10.1016/j.jdiacomp.2016.07.025.

88. Hu T, Yao L, Reynolds K, et al. The effects of a low-carbohydrate diet vs. a low-fat diet on novel cardiovascular risk factors: A randomized controlled trial. *Nutrients*. 2015;7(9):7978-7994. doi:10.3390/nu7095377.

89. Hu T, Yao L, Reynolds K, et al. The effects of a low-carbohydrate diet on appetite: A randomized controlled trial. *Nutr Metab Cardiovasc Dis*. 2016;26(6):476-488. doi:10.1016/j.numecd.2015.11.011.

90. Rebholz CM, Reynolds K, Wofford MR, et al. Effect of soybean protein on novel cardiovascular disease risk factors: A randomized controlled trial. *Eur J Clin Nutr*. 2013;67(1):58-63. doi:10.1038/ejcn.2012.186.
91. Wofford MR, Rebholz CM, Reynolds K, et al. Effect of soy and milk protein supplementation on serum lipid levels: A randomized controlled trial. *Eur J Clin Nutr*. 2012;66(4):419-425. doi:10.1038/ejcn.2011.168.
92. Chen J, He J, Wildman RP, Reynolds K, Streiffer RH, Whelton PK. A randomized controlled trial of dietary fiber intake on serum lipids. *Eur J Clin Nutr*. 2006;60(1):62-68. doi:10.1038/sj.ejcn.1602268.
93. Overweight and Obesity Expert Panel. *Managing Overweight and Obesity in Adults: Systematic Evidence Review From the Obesity Expert Panel, 2013*.; 2013.
94. The Data Center. *Who Lives in New Orleans and Metro Parishes Now?*; 2017. https://www.datacenterresearch.org/data-resources/who-lives-in-new-orleans-now/.
95. Wetzels JFM, Kiemeney LALM, Swinkels DW, Willems HL, Heijer M Den. Age- and gender-specific reference values of estimated GFR in Caucasians: The Nijmegen Biomedical Study. *Kidney Int*. 2007;72(5):632-637. doi:10.1038/sj.ki.5002374.
96. Radin MS. Pitfalls in hemoglobin A1c measurement: When results may be misleading. *J Gen Intern Med*. 2014;29(2):388-394. doi:10.1007/s11606-013-2595-x.
97. Kim PS, Woods C, Georgoff P, et al. A1C underestimates glycemia in HIV infection. *Diabetes Care*. 2009;32(9):1591-1593. doi:10.2337/dc09-0177.
98. Qaseem A, Wilt TJ, Kansagara D, Horwitch C, Barry MJ, Forciea MA. Hemoglobin A1c Targets for Glycemic Control With Pharmacologic Therapy for Nonpregnant Adults With Type 2 Diabetes Mellitus: A Guidance Statement Update From the American College of Physicians. *Ann Intern Med*. 2018;168(8):569-576. doi:10.7326/M17-0939.
99. Sumithran P, Prendergast LA, Delbridge E, et al. Ketosis and appetite-mediating nutrients and hormones after weight loss. *Eur J Clin Nutr*. 2013;67(7):759-764. doi:10.1038/ejcn.2013.90.
100. Gardner CD, Trepanowski JF, Gobbo LCD, et al. Effect of low-fat VS low-carbohydrate diet on 12-month weight loss in overweight adults and the association with genotype pattern or insulin secretion the DIETFITS randomized clinical trial. *JAMA - J Am Med Assoc*. 2018;319(7):667-679. doi:10.1001/jama.2018.0245.
101. Brenner BM, Meyer TW, Hostetter TH. Dietary protein intake and the progressive nature of kidney disease: the role of hemodynamically mediated glomerular injury in the pathogenesis of progressive glomerular sclerosis in aging, renal ablation, and intrinsic renal disease. *N Engl J Med*. 1982;307(11):652-659. doi:10.1056/NEJM198209093071104.
102. Sato J, Kanazawa A, Makita S, et al. A randomized controlled trial of 130 g/day low-carbohydrate diet in type 2 diabetes with poor glycemic control. *Clin Nutr*. 2017;36(4):992-1000. doi:10.1016/j.clnu.2016.07.003.
103. Yancy WS, Foy M, Chalecki AM, et al. A low-carbohydrate, ketogenic diet to treat type 2 diabetes. *Nutr Metab (Lond)*. 2005;2(1):34. doi:10.1186/1743-7075-2-34.
104. Boden G, Sargrad K, Homko C, Mozzoli M, Stein TP. Effect of a low-carbohydrate diet on appetite, blood glucose levels, and insulin resistance in obese patients with type 2 diabetes. *Ann Intern Med*. 2005;142(6). doi:10.1016/S0084-3741(08)70336-6.
105. Stern L, Iqbal N, Seshadri P. The Effects of Low-Carbohydrate versus Conventional Weight Loss Diets in Severely Obese Adults : One-Year Follow-up of a randomized control trial. *Ann Intern …*. 2004;140(May 2001):769-777.

doi:10.1016/j.accreview.2004.07.103.

106. Westman EC, Yancy WS, Edman JS, Tomlin KF, Perkins CE. Effect of 6-month adherence to a very low carbohydrate diet program. *Am J Med*. 2002;113(1):30-36. doi:10.1016/S0002-9343(02)01129-4.

107. Moosheer SM, Waldschütz W, Itariu BK, Brath H, Stulnig TM. A protein-enriched low glycemic index diet with omega-3 polyunsaturated fatty acid supplementation exerts beneficial effects on metabolic control in type 2 diabetes. *Prim Care Diabetes*. 2014;8(4):308-314. doi:10.1016/j.pcd.2014.02.004.

108. Aagaard K, Petrosino J, Keitel W, et al. The Human Microbiome Project strategy for comprehensive sampling of the human microbiome and why it matters. *FASEB J*. 2013;27(3):1012-1022. doi:10.1096/fj.12-220806.

109. McInnes P, Cutting M. Manual of Procedures for Human Microbiome Project. http://www.hmpdacc.org/doc/HMP_MOP_Version12_0_072910.pdf.

110. Marrero DG, Palmer KNB, Phillips EO, Miller-Kovach K, Foster GD, Saha CK. Comparison of commercial and self-initiated weight loss programs in people with prediabetes: A randomized control trial. *Am J Public Health*. 2016;106(5):949-956. doi:10.2105/AJPH.2015.303035.

111. Barengolts E, Manickam B, Eisenberg Y, Akbar A, Kukreja S, Ciubotaru I. EFFECT OF HIGH-DOSE VITAMIN D REPLETION ON GLYCEMIC CONTROL IN AFRICAN-AMERICAN MALES WITH PREDIABETES AND HYPOVITAMINOSIS D. *Endocr Pract*. 2015. doi:10.4158/EP14548.OR.

112. Block G, Azar KMJ, Romanelli RJ, et al. Diabetes prevention and weight loss with a fully automated behavioral intervention by email, web, and mobile phone: A randomized controlled trial among persons with prediabetes. *J Med Internet Res*. 2015. doi:10.2196/jmir.4897.

113. Kramer MK, Molenaar DM, Arena VC, et al. Improving employee health: Evaluation of a worksite lifestyle change program to decrease risk factors for diabetes and cardiovascular disease. *J Occup Environ Med*. 2015. doi:10.1097/JOM.0000000000000350.

114. Parker AR, Byham-Gray L, Denmark R, Winkle PJ. The Effect of Medical Nutrition Therapy by a Registered Dietitian Nutritionist in Patients with Prediabetes Participating in a Randomized Controlled Clinical Research Trial. *J Acad Nutr Diet*. 2014;114(11):1739-1748. doi:10.1016/j.jand.2014.07.020.

115. Maruthur NM, Ma Y, Delahanty LM, et al. Early response to preventive strategies in the diabetes prevention program. *J Gen Intern Med*. 2013;28(12):1629-1636. doi:10.1007/s11606-013-2548-4.

116. Leong A, Daya N, Porneala B, et al. Prediction of Type 2 Diabetes by Hemoglobin A1c in Two Community-Based Cohorts. *Diabetes Care*. 2017;41(1):dc170607. doi:10.2337/dc17-0607.

117. Khaw K-T, Wareham N, Bingham S, Luben R, Welch A, Day N. Association of hemoglobin A1c with cardiovascular disease and mortality in adults: the European prospective investigation into cancer in Norfolk. *Ann Intern Med*. 2004;141(6):413-420. doi:10.7326/0003-4819-141-6-200409210-00006.

118. Cole RE, Boyer KM, Spanbauer SM, Sprague D, Bingham M. Effectiveness of Prediabetes Nutrition Shared Medical Appointments. *Diabetes Educ*. 2013;39(3):344-353. doi:10.1177/0145721713484812.

Low-Carbohydrate Dietary Pattern on Glycemic Outcomes Trial

PROTOCOL

PROTOCOL VERSION 1.4

Dated: March 18, 2019

New Orleans, LA

Table of Contents

1. Overview 5
2. Background and Significance 6
2.A. Type 2 Diabetes Mellitus is a major global public health challenge 6
2.B. Prediabetes is a major risk factor for cardiometabolic diseases 6
2.C. Role of lifestyle in preventing diabetes 6
2.D. Low-to-moderate carbohydrate diets, weight loss, and cardiometabolic diseases 7
2.E. Mediterranean-style diet is associated with reduced risk of T2D and improved glycemic control 8
2.F. Evidence for low-carbohydrate diets that emphasize Mediterranean-style dietary components 8
2.G. Evidence for specific food groups and risk of T2D 8
2.H. Gut microbiota, diet and T2D 9
2.I. Clinical and public health significance of the proposed study 9
2.J. Innovation 9
3. Preliminary Studies 10
3.A. Summary of aggregate experience of the research team 10
3.B. Low-carbohydrate behavioral dietary intervention trial experience 10
3.C. Other dietary intervention trial experience 11
4. Study Design and Organization 11
4.A. Overview of trial design 11
4.B. Study outcomes 12
4.B.1. Primary outcome 12
4.B.2. Secondary outcomes 12
4.C Study Timeline 12
6. Study Participants 13
6.A. Eligibility criteria 13
7. Recruitment and Randomization 13
7.A. Recruitment 13
7.A.1. Mass mailing, placement of brochures/flyers in community, TV/radio/billboard advertisements 14
7.A.2. Database searches and targeted mailing 14
7.A.3. Referrals and Local Partnerships 14
7.B. Randomization 14
7.C. Masking 15

7.D. Safety Considerations 15
8. Dietary Intervention Program 15
8.A. Low-carbohydrate dietary intervention group 15
8.B. Usual Diet Group 16
8.C. Physical Activity 16
8.D. Dietary Adherence 17
9. Safety of Dietary Intervention 17
10. Data Collection 18
10.A. Study visits 18
10.A.1. Screening/baseline visits 18
10.A.2. Follow-up visits and final visit 19
10.B Study measurements 19
10.B.1. Questionnaires 19
10.B.2. Dietary measurements 20
10.B.3. Assessment and management of adverse effects 20
10.B.4. BP and anthropometric measures 20
10.B.5. Blood samples 20
10.B.6. Urine collection 21
10.B.7. Stool Specimen Collection 21
11. Quality Assurance and Quality Control 21
11.A. Manual of procedures 21
11.B. Training and certification 21
12. Sample Size and Statistical Power 22
13. Data Management and Analysis 23
13.A. Data management 23
13.B. Data analysis plan 23
13.B.1. Exploratory data analysis 23
13.B.2. Primary, secondary, and exploratory outcomes 23
15. Anticipated Challenges 24
16. Protection of Human Subjects 24
16.A. Protection of human subjects from research risks 24
16.A.1. Risks to the subjects 24
16.A.2. Adequacy of protection against risks 25

16.A.3. Potential benefits of the proposed research to human subjects and others... 27
16.A.4. Importance of knowledge to be gained ... 27
16.A.5. Remuneration ... 27
16.B. Data safety and monitoring plan ... 27
Bibliography and References Cited ... 29

1. Overview

The increasing prevalence of type 2 diabetes mellitus (T2D), a major cause of morbidity and mortality worldwide, is a critical public health concern. In the United States, an estimated 33.9% of adults have prediabetes (impaired fasting glucose, elevated hemoglobin A1c (HbA1c), or impaired glucose tolerance). Individuals with prediabetes are at elevated risk of T2D and cardiovascular disease (CVD), with 5–10% developing clinical T2D each year.

In the short-term, among patients with T2D, low-to-moderate carbohydrate diets (energy percentage below 45) have a greater glucose-lowering effect than do high-carbohydrate diets, with larger glucose-lowering effect the greater the carbohydrate reduction. Additionally, compared with low-fat diets, Mediterranean diets that are high in unsaturated fats (particularly *cis*-monounsaturated fats) reduce risk of T2D and CVD and improve glycemic control among diabetics. However, compared with usual diets, the effect of a behavioral intervention promoting a low-carbohydrate/high-unsaturated fat and protein dietary pattern that emphasizes consumption of non-starchy vegetables, plant-based oils, fish, poultry, and mixed nuts on glucose homeostasis, and other metabolic risk factors among individuals with prediabetes or early-stage untreated T2D is not well understood.

Our *long-term goal* is to develop and implement dietary intervention(s) that reduce risk of T2D. The *overall goal of this randomized controlled trial is to study the effect of a behavioral intervention promoting a low-carbohydrate/high-unsaturated fat and high-protein dietary pattern (initial target <40 g digestible carbohydrates, final target <60 g digestible carbohydrates) compared with a usual diet on metabolic risk factors among individuals with prediabetes or early-stage untreated diabetes* (HbA1c 6.0–6.9%). Specifically, we will test the following hypotheses:

Primary hypothesis: Compared with usual diet, over a 6-month period, a healthy low-carbohydrate/high unsaturated fat and high-protein dietary pattern will lead to *a larger decrease of HbA1c, by at least 0.15%.*

Secondary hypothesis: Over a 6-month period, a healthy low-carbohydrate/high-unsaturated fat and high-protein dietary pattern will lead to *larger improvements in the following major secondary outcomes*: fasting glucose, systolic blood pressure (BP), total-to-HDL-cholesterol ratio, and body weight.

Exploratory outcomes: We will also study the effect of the intervention on the following outcomes: insulin and homeostasis model assessment of insulin resistance (HOMA-IR); diastolic BP; waist circumference; and estimated CVD risk.

To achieve these study aims, we will recruit 112 adults with prediabetes or untreated diabetes (HbA1c 6.0–6.9%) in the Greater New Orleans, LA area. Using a stratified, blocked randomization scheme, participants will be assigned to either a behavioral modification intervention aimed at promoting a healthy low-carbohydrate dietary pattern or to usual diet. The intervention group will have an intensive phase for 3 months, followed by a maintenance phase in months 4 through 6. The *primary outcome will be difference between the active intervention and control groups in the change in HbA1c from baseline to 6 months of follow-up. We expect 85% power to detect a difference of at least 0.15% in change of HbA1c.* Changes in other metabolic risk factors will be studied concurrently. Metabolic risk factors will be assessed at baseline, 3 months, and 6 months of follow-up.

There is much interest regarding dietary approaches to prevent T2D and its complications. The results from this study will help to determine whether, compared with usual diet, a behavioral intervention aimed at promoting a healthy low-carbohydrate dietary pattern improves metabolic risk factors.

2. Background and Significance

2.A. Type 2 Diabetes Mellitus is a major global public health challenge

Diabetes is a strong and independent risk factor for CVD morbidity and mortality. A meta-analysis of 102 prospective studies (698,782 participants) estimated diabetes leads to an approximately 2-fold increased risk in vascular diseases, including coronary heart disease (CHD) and major stroke subtypes, and deaths due to other vascular causes.[1] Among participants with diabetes, CHD risk is about 50% higher among those with fasting blood glucose ≥7 mmol/L compared with those with fasting blood glucose <7 mmol/L.[1] In addition to increasing CVD risk, diabetes can cause diabetic retinopathy,[2] chronic kidney disease,[3] lower limb amputation,[4] and other complications. Most individuals with diabetes have T2D.[5] The US Centers for Disease Control and Prevention (CDC) estimated that 30.2 million adults ≥18 years (12.2%) had diabetes in 2015, with 7.2 million (23.8%) not being aware of or reporting diabetes.[5] Diagnosed diabetes prevalence among US adults has increased substantially over time, from 2.54% in 1980 to 7.40% in 2015.[6] Diabetes prevalence is also high globally, with an estimated 2014 age-standardized prevalence of 9.0% in men and 7.9% in women.[7] Diabetes accounts for an increasing portion of US healthcare expenditures. Total cost of diagnosed diabetes in the US was estimated to be $245 billion in 2012, 41% higher than in 2007.[8] Diabetes is associated with much higher lifetime medical expenses, with higher expenses for those diagnosed at younger ages.[9]

2.B. Prediabetes is a major risk factor for cardiometabolic diseases

Prediabetes, defined by the American Diabetes Association (ADA) as impaired fasting glucose (IFG), impaired glucose tolerance (IGT), or elevated HbA1c, is also referred to as “intermediate hyperglycemia”[10] and “high-risk state of developing diabetes”.[11] The ADA defines prediabetes as having: fasting plasma glucose of 100–125 mg/dL (IFG); 2-hour post-load glucose in the 75-g oral glucose tolerance test (OGTT) of 140–199 mg/dL (IGT); or HbA1c 5.7–6.4%.[12] Prediabetes itself is not a disease; for all three tests, “risk is continuous, extending below the lower limit of the range and becoming disproportionately greater at the higher end of the range”.[12] The CDC estimated that in 2015, 33.9% of US adults had prediabetes based on fasting glucose or HbA1c.[5] Among those with prediabetes, only 11.9% reported having being told this by a medical professional.[5] Approximately 5–10% of individuals with prediabetes develop diabetes within one year, depending on how prediabetes is defined.[13] In a meta-analysis, annual diabetes incidence was 15–19% among individuals with both IFG and IGT, while it was 4–6% in those with isolated IFG and 6–9% in those with isolated IGT.[13]

A recent meta-analysis (1,611,339 participants from 53 studies) reported that prediabetes as defined by the ADA is associated with higher risk of composite CVD events and CHD.[14] IFG and IGT were also positively associated with increased risk of stroke and all-cause mortality.[14] Prediabetes is linked with increased risk of other conditions typically thought of as diabetes complications, such as neuropathies and nephropathies.[15] Importantly, it is possible for individuals with prediabetes to revert to normoglycemia, as observed in observational studies[13] and lifestyle or drug intervention trials.[16,17] In the Diabetes Prevention Program (DPP), reversion to normoglycemia was associated with lower long-term T2D risk[18] and lower predicted CVD risk.[19]

2.C. Role of lifestyle in preventing diabetes

Much evidence from observational studies and clinical trials supports the critical role of diet and other lifestyle factors in preventing T2D. Globally, lifestyle transitions towards reduced physical activity, increased consumption of refined carbohydrates, coupled with resulting changes in body composition, has led to increases in diabetes prevalence and severity.[20] Body

mass index (BMI), waist circumference, and waist-to-height ratio are predictors of T2D.[21] The DPP, which combined calorie restriction and exercise to promote weight loss, substantially reduced risk of T2D (by 58%) among individuals with elevated FPG and IGT;[22] a benefit that persisted in 10-year follow-up analyses.[16] Other trials of lifestyle modification focused on diet and exercise have also shown benefits in study populations in China,[23] Finland,[24] and India.[25]

2.D. Low-to-moderate carbohydrate diets, weight loss, and cardiometabolic diseases

Low-to-moderate carbohydrate diets (<45% of energy from carbohydrates) are popular dietary approaches for losing weight. Weight loss is a key component of the DPP lifestyle intervention[26] and ADA lifestyle recommendations.[12,27] Meta-analyses from our group[28] and others support that low-to-moderate carbohydrate diets are at least as effective as low-fat diets at promoting weight loss. For instance, a meta-analysis reported a 2-kg (95% CI, 3.1–0.9) larger weight loss in low-carbohydrate diet (<120 g carbohydrates/day) arms compared with low-fat diet (<30% energy from fat) arms.[29] Estimated 10-year CVD risk was lower in low-carbohydrate than low-fat arms.[29] This aligns with research published by our team; in a 12-month trial of participants with obesity, a low-carbohydrate diet (target <40 g carbohydrates/day) was more effective than a low-fat diet (target <30% energy from fat, <7% saturated fat) for weight loss and CVD risk factor reduction.[30]

Low-to-moderate carbohydrate diets may also be beneficial among patients with T2D. Low-carbohydrate diets have come in and out of favor as treatment of diabetes.[31] In a meta-analysis (1376 patients;10 trials), after 3 or 6 months of the intervention, low-to-moderate carbohydrate diets (<45% energy from carbohydrates) led to a 0.34% lower HbA1c (95% CI, 0.06–0.63%) compared with high-carbohydrate diets.[32] Compared with high-carbohydrate diets, there was a greater reduction in HbA1c the greater the reported carbohydrate restriction.[32] There was no benefit of the low-to-moderate carbohydrate diets on HbA1c at 12 months or longer, and no difference between interventions on BMI, body weight, low-density lipoprotein (LDL) cholesterol or quality of life.[32] However, results may have been affected by dietary adherence or glucose medication. There was evidence of diabetes medication reduction in the low-carbohydrate arms in some studies,[33–37] with evidence of medication reduction and improved blood glucose stability persisting for up to 2 years.[38] Hypoglycemia (though not after medication adjustment) was observed in three subjects taking insulin or a sulfonylurea out of twelve subjects in the low-carbohydrate arm of another study.[39] In a subset of participants with T2D (n=46) from a large weight loss trial, compared with those randomized to a low-fat diet and orlistat, those randomized to a low-carbohydrate diet for 48 weeks had improved HbA1c (0.8% lower, 95% CI, -1.5, -0.02%) and a reduction in antiglycemic medications, despite similar weight loss effects of the interventions.[40]

Though low-to-moderate carbohydrate diets have been studied among overweight and obese participants (primarily focused on weight loss), less is understood about the effects of such diets on glycemic outcomes specifically among individuals with prediabetes. A study among participants with IGT observed a reduction at 12 months in weight, HbA1c, and other glycemic outcomes among individuals who participated in a 7-day in-hospital education program, though this was not randomized.[41] A randomized pilot trial of overweight or obese individuals with T2D or prediabetes found that, over 3 months and at 12 months, a very low-carbohydrate diet improved HbA1c, led to higher medication discontinuation, and more weight loss compared with a low-fat diet.[34,37] However, only 4 individuals had prediabetes. Given benefits of low-to-moderate carbohydrate diets for weight loss in general populations and for glycemic control among patients with T2D, further study of these diets are warranted among individuals with prediabetes who are at elevated cardiometabolic disease risk.

2.E. Mediterranean-style diet is associated with reduced risk of T2D and improved glycemic control

Though there is no one "Mediterranean diet", the Mediterranean dietary pattern tends to have the following characteristics: high consumption of vegetables, fruits, breads and cereals, legumes, nuts and seeds; low-to-moderate consumption of dairy products, fish, poultry and eggs; little consumption of red meat and sweets; and red wine in moderation.[42] Olive oil is an important source of fat. In many observational studies, adherence to the Mediterranean-style dietary pattern is associated with reduced risk of CVD[43] and T2D.[44,45] Evidence from randomized trials also supports the benefits of this dietary pattern. In several trials, participants randomized to Mediterranean diets had substantially lower risk of CVD than those randomized to low-fat control diets.[46,47] In the three-arm randomized PREDIMED trial of participants at high risk of CVD, after 4.1 years of follow-up, those randomized to a traditional Mediterranean diet supplemented with either olive oil or nuts had a 30% reduced risk of T2D compared to those in the low-fat control diet group.[48] In all PREDIMED arms, average carbohydrate intake was relatively low (39.7–43.7% at end of trial).[47] Two trials have found a higher rate of remission from metabolic syndrome among individuals randomized to a Mediterranean diet compared with those randomized to control diets (prudent or low-fat) during 2–5 years of follow-up.[49,50] The Mediterranean pattern may also be beneficial among individuals with T2D. Meta-analyses have reported that, among patients with T2D, those randomized to a Mediterranean diet had lower HbA1c levels and CVD risk factors, and more significant weight loss, compared with those randomized to control diets.[51]

2.F. Evidence for low-carbohydrate diets that emphasize Mediterranean-style dietary components

Many low-carbohydrate diets involve increased consumption of food items, such as red meat,[52] that are associated with increased cardiometabolic disease risk. However, low-carbohydrate dietary approaches can focus on substituting carbohydrates with foods common in Mediterranean-style diets, such as olive oil and nuts, which may improve cardiometabolic health. In a 4-year Italian study of individuals with newly diagnosed T2D, those randomized to a low-carbohydrate Mediterranean diet (<50% carbohydrates; consumed ~44%) had more favorable changes in glycemic control, higher rates of T2D remission, and delayed need for antiglycemic drugs compared with participants randomized to a low-fat diet (<30% fat, <10% saturated fat).[53,54] A 12-month Israeli study assessed low-carbohydrate Mediterranean (35% carbohydrates), traditional Mediterranean, and ADA (both 50–55% carbohydrates) diets in 259 overweight participants with T2D.[55] Most CVD risk factors improved in all groups; there was only HDL improvement in the low-carbohydrate Mediterranean diet and this diet was superior to the other diets in improving glycemic control.[55] In a 2-year weight-loss study of moderately obese participants, low-carbohydrate and Mediterranean diets were superior to low-fat diet for weight loss.[56] Participants in the low-carbohydrate arm were counseled to choose vegetarian sources of fat and protein and to avoid trans fat. At 6 years after study commencement, despite weight regain, there were still long-term benefits, particularly for the low-carbohydrate and Mediterranean diets.[57] In whole, evidence supports that low-carbohydrate Mediterranean-style diets are feasible and may have metabolic benefits.

2.G. Evidence for specific food groups and risk of T2D

Prospective cohort studies have found consumption of specific foods and beverages is associated with diabetes risk. Meta-analyses report higher consumption of processed and unprocessed red meat,[58] white rice (particularly in Asian populations),[59] and sugar-sweetened beverages[60,61] are associated with higher T2D risk. Consumption of green leafy vegetables,[62,63] yogurt,[64] whole grains,[65] decaffeinated and total coffee,[66] and light-to-moderate alcohol

consumption[67] are associated with reduced risk. Consumption of fruits, particularly blueberries, grapes, and apples, are associated with reduced risk; fruit juice is associated with higher risk.[68]

2.H. Gut microbiota, diet and T2D

There is increasing evidence for the importance of gut microbiota in risk of type 2 diabetes and other cardiometabolic diseases.[69] Additionally, dietary modifications, including changes in carbohydrate consumption, can lead to substantial changes in gut microbiome over short periods of time.[70,71] Less is known about longer-term changes to the gut microbiota during the adoption of a low-carbohydrate diet and how these changes may influence cardiometabolic risk. We plan to collect stool specimens at baseline and at 6 months, for potential future studies on how this intervention affects the gut microbiome.

2.I. Clinical and public health significance of the proposed study

Prediabetes, or intermediate hyperglycemia, is a marker of high risk of developing T2D and other complications. Due to increasing prevalence of T2D and associated morbidity and mortality globally, a better understanding of dietary approaches to delay progression to T2D or to promote reversion to normoglycemia is a high public health priority. This study proposes to assess the effect of a behavioral intervention promoting a low-carbohydrate/high-unsaturated fat dietary pattern (initial target <40 g digestible carbohydrates, final target <60 g digestible carbohydrates) on HbA1c, a marker of glycemic control, and other metabolic risk factors. Building from prior findings from clinical and observational studies, we will develop a dietary intervention that promotes the consumption of non-starchy vegetables, plant-based oils, fish, poultry, and mixed nuts, that are thought to reduced risk of T2D or CVD. Participants assigned to the intervention will be trained in behavioral techniques that will provide capacity to maintain dietary changes long after study completion. Our outcome measures will assess the effect of this dietary intervention in a "real-life" setting as opposed to dietary modifications achieved in metabolic ward studies or through food prepared in research kitchens. Findings from this study may provide evidence in support of promoting alternative dietary styles among individuals with high T2D and CVD risk and could potentially be used to inform dietary interventions used in programs like DPP.

2.J. Innovation

The status quo as it pertains to dietary approaches for reducing risk of T2D is focused on reducing caloric intake and total fat intake. However, recent evidence suggests that the types of fat, rather than the quantity of fats may be more important, with evidence supporting that a Mediterranean-style diet that does not restrict fat intake reduces risk of developing both T2D[48] and CVD[47]. Moreover, in the short-term, low-carbohydrate diets, which are popular weight loss diets, have a greater glucose-lowering effect than do high-carbohydrate diets among patients with T2D.[32] However, the effect of low-carbohydrate diets that emphasize consumption of non-starchy vegetables, plant-based oils, fish, poultry, and mixed nuts on T2D prevention is unclear, particularly compared with higher-carbohydrate diets more commonly consumed in the US. *The proposed research is innovative, in our opinion, because it represents a substantial departure from the status quo by assessing the combined effect of a low-carbohydrate, high unsaturated fat and protein diet on cardiometabolic risk factors among adults with prediabetes or early-stage untreated diabetes.* We expect the results from this study have the potential to lead to new horizons for developing and implementing dietary approaches (other than the most commonly used reduced fat diet) that will substantially reduce risk of cardiometabolic disease among adults with T2D or at high risk of T2D. The proposed intervention dietary approach promotes consumption of dietary components thought to reduce risk of cardiometabolic disease and the behavior change intervention has expected applicability in clinical practice.

3. Preliminary Studies

3.A. Summary of aggregate experience of the research team

Dr. Kirsten Dorans is trained in cardiovascular disease epidemiology, with strong training in epidemiologic methods and biostatistics. Her publication record includes studies of behavioral risk factors and risk of chronic disease. She has published on the relationships between dietary intake and chronic heart failure[72,73] and on physical activity and incident multiple sclerosis[74]. She served as lead author, conducting data management, analysis, drafting, and revision of two of these manuscripts. In graduate school, she focused on studying links between outdoor air pollution and chronic diseases and has published several papers in this field. The COBRE program will provide an excellent opportunity for the JFI to transition from air pollution research to clinical and translational research focused on nutrition and diabetes. Building off of her training in observational epidemiology, this program will involve training and practical experience that will allow her to broaden her expertise and to develop practical skills for carrying out clinical research.

The mentorship team has much experience and prior successful collaborations in key areas related to this study. Dr. Jiang He is an internationally renowned expert in cardiometabolic disease clinical and epidemiological research. He has served as PI for many epidemiological studies and clinical trials, including clinical and observational studies focused on dietary consumption and chronic diseases[75–80] and has conducted extensive global research on diabetes.[81,82] Dr. Bazzano has collaborated extensively with Dr. He for more than 15 years in research related to diet and chronic disease. She is a clinical investigator and has expertise in nutritional epidemiology and clinical research. Dr. Bazzano was the PI of a highly successful clinical trial comparing the effects of low-carbohydrate and low-fat diets on weight and cardiovascular risk factors among individuals with obesity, which was a subproject within the Tulane COBRE in Hypertension and Renal Biology.[30] Dr. He served as Dr. Bazzano's mentor for this clinical trial. They have collaborated on much other work related to dietary and chronic disease, including a meta-analysis on low-carbohydrate diets.[28] Dr. Bazzano has also published many articles on diet and risk of diabetes[83,84] and on diabetes in the Bogalusa Heart Study.[85–87]

3.B. Low-carbohydrate behavioral dietary intervention trial experience

In the study on macronutrient composition of diet and risk factors for cardiovascular disease (MACRO) study, 148 individuals without clinical cardiovascular disease or diabetes and with obesity were randomly assigned to a low-carbohydrate or a low-fat behavioral dietary intervention for 12 months. In the low-carbohydrate arm, daily dietary intake of carbohydrates decreased from an average of 242 g at baseline to 97 g, 93 g, and 127 g at 3, 6, and 12 months, respectively. At 12 months, participants in the low-carbohydrate intervention group had greater decreases in weight (-3.5 kg; 95% CI, -5.6 to -1.4 kg) and fat mass (-1.5%; 95% CI, -2.6% to -0.4%) and greater improvement in several cardiovascular risk factors.[30] This suggests that restricting carbohydrates may be an option for weight loss and reduction of cardiovascular risk factors. Further analyses found that the low-carbohydrate dietary intervention led to similar or greater improvement in inflammation, adipocyte dysfunction, and endothelial dysfunction compared with the low-fat dietary intervention[88] and that the low-fat diet reduced peptide YY more than the low-carbohydrate diet, suggesting satiety may be better preserved on low-carbohydrate diets.[89] No serious adverse events were reported in this study and the number of participants with symptoms were similar between the two diets, with the exception of more participants having headaches on the low-fat diet at 3 months (18 vs 6 participants, $p = 0.03$).[30] Dr. Bazzano served as PI for this study and Dr. He served as her mentor. Dr. Bazzano

mentored one of her PhD students in conducting analyses and writing and editing manuscripts related to this project.

3.C. Other dietary intervention trial experience

Dr. He has also led or been involved with many dietary intervention trials, including both behavioral change and feeding study interventions. In the Protein and Blood Pressure (ProBP) study, 352 adults with prehypertension or stage 1 hypertension in New Orleans, LA and Jackson, MS were assigned to take, in a random order, 40 g/d soy protein, milk protein, or carbohydrate supplementation each for 8 weeks. Compared with the high-glycemic index refined carbohydrate, both soy protein and milk protein supplementation were associated with a -2.0 mm Hg (95% CI -3.2 to -0.7 mm Hg) and -2.3 mm Hg (-3.7 to -1.0 mm Hg) net change in systolic blood pressure.[80] These findings suggest that partial replacement of carbohydrate with soy or milk protein might be an important part of nutrition intervention strategies to prevent and treat hypertension. Additionally, soy protein supplementation led to reduction in plasma E-selectin compared with carbohydrate and milk protein and in plasma leptin compared with carbohydrate.[90] Soy protein also led to improvement in lipid profile compared with carbohydrate and milk protein.[91] Dr. He was PI for this project.

In the Fiber Intervention on Blood Pressure (FIBRE) study, 110 participants ages 30–65 years with untreated pre-hypertension or stage-1 hypertension and serum cholesterol level < 240 mg/dl were randomly assigned to 8-g/day of water soluble fiber from oat bran or a control intervention (double-blind RCT). Over the 12-week study period, net changes in systolic and diastolic blood pressure were -1.8 mm Hg (-4.3 to 0.8) and -1.2 mm Hg (-3.0 to 0.5 mm Hg), respectively.[78] Net changes in total, HDL-, and LDL-cholesterol were -2.40 mg/dL (-10.60 to 5.81 mg/d:), -1.66 mg/dL (-4.55 to 1.22 mg/dL), and -1.33 mg/dl (-8.33 to 5.68 mg/dL), respectively.[92] Dr He was PI for this study.

Dr. He is currently serving as a mentor for Dr. Katherine Mills' COBRE project (Effect of Dietary Sodium Reduction in Kidney Disease Patients with Albuminuria; SUPER). In this project, participants with chronic kidney disease and albuminuria are being randomized to a behavioral modification program designed to reduce dietary sodium intake to ≤2,300 mg/day or to usual care and test the effect of this intervention on change in albumin-to-creatinine ratio from baseline to 24-weeks.

4. Study Design and Organization

4.A. Overview of trial design

We propose to conduct a ***randomized controlled trial*** aimed at testing the effect of a behavioral intervention that promotes ***reduced carbohydrate intake and increased consumption of heart-healthy unsaturated fats and protein*** in patients with HbA1c 6.0–6.9% (*Figure 1*). We plan to recruit 112 individuals with elevated HbA1c and randomly assign them to a moderately intensive intervention on diet pattern,[93] using well-established methods for behavioral modification, or to usual diet. We will compare the difference between the active intervention and control groups for change in HbA1c from baseline to 6 months of follow-up (primary outcome). We will assess four major secondary outcomes: fasting plasma glucose, systolic BP, total-to-HDL cholesterol ratio, and body weight. In

Figure 1. Study Design

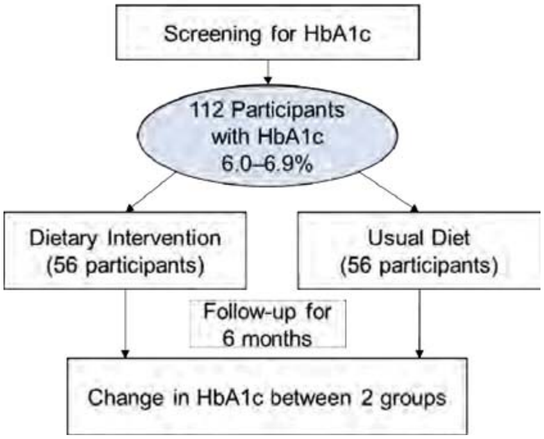

addition, we will look at several other exploratory outcomes: insulin and HOMA-IR, diastolic BP, waist circumference, and estimated CVD risk, as estimated by the 2013 ACC/AHA risk equation. The main trial components are summarized in *Table 1*.

Table 1. Study Overview

Study Design	Randomized controlled trial
Study Participants	112 participants with elevated HbA1c (6.0–6.9%)
Intervention Group (n=56)	Behavioral modification to reduce carbohydrate consumption and promote consumption of foods such as non-starchy vegetables, plant-based unsaturated oils, nuts and seeds, avocados, eggs, seafood, poultry, lean meat and tofu. We will recommend moderate consumption of cheese, unsweetened Greek yogurt, and low-carb milk. If consuming higher-carbohydrate items, recommend legumes, fruits, (limited) whole grains. Target <40 g net carbohydrates per day for first 3 months and will have a goal of <40 net g carbohydrates per day; <60 g net carbohydrates per day for months 4 onwards.
Usual Diet Group (n=56)	No dietary intervention. To encourage continued participation in the trial, we will offer optional monthly educational sessions on unrelated topics, such as retirement planning or health insurance
Outcome Measures	Primary: HbA1c; Secondary: fasting plasma glucose, systolic BP, total-to-HDL-cholesterol ratio, and body weight

4.B. Study outcomes

4.B.1. Primary outcome: The *primary outcome will be the difference between the intervention group and the usual diet group for change in HbA1c* from baseline to 6 months. Changes in HbA1c will be calculated from blood samples taken at baseline and 6 months.
4.B.2. Secondary outcomes: Secondary outcomes will include changes from baseline to 6 months of *fasting plasma glucose, systolic BP, total-to-HDL cholesterol ratio, and body weight.,*
4.B.3. Other outcomes: We will also study the effect of the intervention on the following outcomes: insulin and homeostasis model assessment of insulin resistance (HOMA-IR); diastolic BP; waist circumference; total-to-HDL-cholesterol ratio; and estimated CVD risk.

4.C Study Timeline

Table 2 shows the timeline, which will be followed in the implementation of study-related activities, such as publications and submission of an R01 grant application.

Table 2. Study Timeline

	Year 1		Year 2		Year 3	
Develop MOP, intervention program, study forms, and pilot testing	X	X				
Certification and training of staff	X					
Recruitment		X	X			
Intervention and data collection, measurement, entry, cleaning		X	X	X	X	
Data quality control and analysis						X
Publications	X	X	X	X	X	X
R01 grant submissions, resubmissions				X	X	X

The first 6 months will focus on development of manual of operations (MOP), intervention program, study forms, study protocol, Institutional Review Board (IRB) approval, and pilot

testing of the intervention program. It will also involve staff training and certification. During the last half of year 1, recruitment, intervention, and data collection will commence. During the last 3 months of year 3, data quality control and analysis will be carried out. Throughout the study period, the JFI will publish peer-reviewed manuscripts from ongoing Tulane University research projects.

6. Study Participants

6.A. Eligibility criteria

Inclusion criteria

- Men or women ages 40 to 70 years: aim to recruit 50% women
- Any race or ethnicity: aim to recruit 40% ethnic minorities (i.e., African American and Hispanic), close to population in the greater New Orleans area[94]
- HbA1c 6.0–6.9%
- Willing and able to provide informed consent

Exclusion criteria

- Diagnosed type 1 diabetes mellitus based on patient self-report
- Use of agents affecting glycemic control (medications for diabetes, oral glucocorticoids) within the past three months prior to enrollment based on patient self-report
- Medical condition in which low-carbohydrate diet may not be advised
    - Estimated glomerular filtration rate (eGFR) ≤45 mL/min/1.73 m², which is close to the 5th percentile of eGFR among non-diseased individuals of 70 years of age[95]
    - Self-report of liver disease due to hepatitis or alcohol; osteoporosis; untreated thyroid disease; gout; or cancer (other than non-melanoma skin cancer) requiring treatment in the past year, unless prognosis is excellent
- Factors that may affect HbA1c:
    - Hemoglobin <11 mg/dL (cutpoint for moderate-to-severe anemia, which could lead to falsely elevated or lowered HbA1c)[96]
    - Recent blood donation or blood transfusion (self-report, past 4 months)
    - Human Immunodeficiency Virus (self-report)[97]
- Allergies to nuts
- For women, current pregnancy, breastfeeding, or plans to become pregnant during the study
- Consumption of ≥21 alcoholic drinks per week or consumption of ≥6 drinks per occasion
- Current or planned residence making it difficult to meet trial requirements (due to distance from study site and/or challenges regularly traveling to site)
- Current participation in another lifestyle intervention trial or a pharmaceutical trial
- Participation of another household member in the study; employees or persons living with employees of the study
- Other concerns regarding ability to meet trial requirements, at the discretion of the principal investigator or study coordinator

7. Recruitment and Randomization

7.A. Recruitment

We will recruit study participants from the greater New Orleans area. The initial focus of our recruitment will be mass mailing of individuals in the greater New Orleans area and recruitment through placing flyers and brochures in the community. If community-based recruitment is not successful or cost-effective, we will recruit out-patients from the Ochsner Health System and

Tulane Medical Center (TMC), from which our group has prior experience recruiting patients. For this recruitment, we will focus on electronic searches of medical records and laboratory databases. We will also carry out targeted solicitation of referrals from physicians who care for patients likely to meet our inclusion and exclusion criteria.

In addition to this recruitment plan, we have several contingency plans: (1) extending recruitment to other medical centers in the New Orleans area, including the Veterans Affairs Medical Center (VAMC), East Jefferson General Hospital, and West Jefferson Medical Center, with whom Tulane has well-established relationships; (2) TV/radio advertisements; (3) arrange a news story about the study in local TV newscasts, radio talk programs, and/or print media; and (4) conduct outreach at local community events and churches. We have ample experience with each of these approaches.

Based on U.S. Census Bureau 2016 population estimates, the metro New Orleans area population was approximately 35% African American or black and 9% Hispanic.[94] Given our prior experience and population distribution in this region, we plan to recruit a distribution of study participants with 40% ethnic minorities.

7.A.1. Mass mailing, placement of brochures/flyers in community, TV/radio/billboard advertisements: Community recruitment approaches include: mass mailing individuals who live in the New Orleans area; brochures/flyers in the community; emailing the brochure; TV, radio, poster, or billboard advertisements in the New Orleans area. Interested individuals will be pre-screened; those at elevated risk for prediabetes or T2D, i.e. individuals who are obese or overweight and/or have a sufficiently high score from the American Diabetes Association's Diabetes Risk Assessment Questionnaire will be invited into the clinic for a screening visit.

7.A.2. Database searches and targeted mailing: We will request IRB approval for a waiver for HIPAA authorization to conduct database searching and hospital chart reviews for identification of potentially eligible study participants. After identifying a potentially eligible patient, we will send this individual an invitation letter signed by the investigators and/or the individual's personal physician. Individuals who express interest in the study will have a pre-screening telephone interview for confirmation of potential eligibility. Those still interested and potentially eligible for enrollment will be scheduled for a visit at the COBRE study clinic.

7.A.3. Referrals and Local Partnerships: In addition to local physician referrals, we will explore holding outreach community events in the New Orleans area. Local community partners may post information about the study on their social media platforms. We may also identify potentially interested volunteers from a Tulane volunteer recruitment registry. We will also consider recruiting potentially eligible individuals who were screened or participated in other clinical studies in our clinic and have expressed interest in being contacted about future research projects. Potential participants referred by their physician or community partners will have a pre-screening telephone interview to confirm potential eligibility. Those who are still interested and potentially eligible for the study will be scheduled for a screening visit.

7.B. Randomization

Once eligibility has been confirmed, the study coordinator will ensure that all key variables have been entered into the trial-specific database. Additionally, the study coordinator will ensure any outliers have been recognized and reconciled, and will ensure receipt of informed consent prior to enrolling the volunteer in the study as a trial participant. Eligible participants will be *randomly assigned to the behavioral intervention or the usual diet group in a 1:1 allocation ratio*. Randomization will be stratified by sex, *using a random block group size (4, 6) to maintain balance among groups over time*. A statistician from the Methodology/Biostatistics Unit within the COBRE Clinical Research Core, who is not directly involved in the study and is not located at the clinic, will conduct the randomization. After ascertaining eligibility, clinical personnel will

telephone the Methodology/Biostatistics Unit to obtain a randomization assignment. All randomization assignments will be created by a computer program and only accessible to the Staff at the Methodology/Biostatistics Unit. Once randomization assignment has been made, trial participants will be considered to be officially randomized into the study and every effort will be made to obtain complete follow-up information.

7.C. Masking

Clinical and laboratory staff members who assess outcomes will be blinded to the intervention assignment of participants. As this is a dietary intervention trial, study participants, and study staff members who conduct the intervention cannot be blinded to the intervention assignment. Participants will be instructed to avoid sharing knowledge of their treatment assignment with data collection staff members. We will have intervention visits and data collection study visits on different days and in separate locations. Data collectors will also use verbal cues and visual props to remind participants not to disclose treatment assignment. Investigators will be blinded to intervention assignment.

7.D. Safety Considerations

Participants with HbA1c >7.9% will be referred to a clinician for follow-up[98], though we will continue to collect data on these participants.

8. Dietary Intervention Program

8.A. Low-carbohydrate dietary intervention group

Participants randomized to the low-carbohydrate intervention group will receive counseling aimed at reducing carbohydrate consumption. Phase I (*Go Low*) will occur for the first 3 months, with a goal of <40 g digestible carbohydrates per day. Key recommended foods will include: non-starchy vegetables, fish, poultry, lean meat, eggs, olive oil and other plant-based unsaturated oils, unsweetened/unsalted nuts and seeds, nut butters, and avocados. We will recommend moderate consumption of cheese and unsweetened Greek yogurt and low-carbohydrate milk. During Phase I, we will recommend limiting or avoiding other dairy, fruits, legumes, beans, and grains. Phase II (*Keep it Low*) will run from months 4 through 6. The net carbohydrate goal for this phase will be <40 g to <60 g, with the objective to choose a target that is as low as possible (e.g., as close to <40 g as possible). At 3 months, we expect different participants will have different levels of intake, with some consuming <40 g of net carbohydrates and others consuming levels higher than 60 g net carbohydrates per day. We have designed the intervention to accommodate various scenarios, with the goal of achieving as low of an intake as possible. For participants who consume >60 g per day, we will recommend they aim to either reduce or maintain carbohydrate intake.

At a brief check-in at 3-months, if participants are satisfied with their current intake, we will instruct them to stay at this intake level (though will recommend they try to reduce intake, if possible, if they are eating >60 net grams of carbohydrates or have not yet reached the study goal of <40 net grams of carbohydrates). If they prefer to make modifications, we will provide guidance on how to modify carbohydrate intake (either decrease or increase). If they choose to add back carbohydrates, we will recommend that participants add 5 grams of carbohydrates for two weeks at a time (e.g., from 40 to 45 g for 2 weeks), for a maximum total of <60 g net carbohydrates. If they choose to further reduce carbohydrate intake, we will provide guidance on this as well. As they modify carbohydrate intake, we will have them monitor their satisfaction with their diet and energy levels. If adding back carbohydrates, we will recommend participants

add back in foods like fruit (preferably berries), legumes, beans, and dairy, while still aiming to keep total carbohydrate intake as low as possible (e.g., as close to <40 net grams as able).

Materials: We will develop a handbook that will be given to participants. This will contain diet guidelines, recipes, meal planners, shopping lists, and guides for counting carbohydrates and reading nutrition labels.

Meals: Participants will select their own menus and prepare or buy their meals according to the guidelines. We will provide participants with supplemental low-carbohydrate foods, such as nuts, olive oil, and other products, to help facilitate their compliance.

Counseling: Sessions and materials will focus on instructing participants to reduce digestible carbohydrate consumption by increasing consumption of protein and fat, with a particular emphasis on increased consumption of heart-healthy unsaturated fats. After randomization, participants will begin the 3-month *Go Low* phase consisting of individual counseling sessions each week for the first four weeks, followed by 4 group sessions held every other week with phone follow-ups occurring in between group sessions. During the *Keep it Low* phase, months 4 through 6, participants will attend 3 monthly group sessions and have 3 telephone follow-ups. There will be a total of 11 in-person sessions and 7 telephone follow-ups over the full study period. Sessions will be approximately one hour and phone calls will be no longer than thirty minutes. These sessions will consist of diet instruction and supportive counseling, addressing results from 24-hour dietary recalls, teaching self-monitoring and individual diet goal changes, and provide suggestions for replacing usual foods containing carbohydrates. Study materials will be modified from materials used in the successful MACRO study. The JFI will work closely with Drs. He and Bazzano to develop the intervention based on the MACRO study.
Carbohydrate goal: In the MACRO study, carried out among individuals from the same study region, a behavioral intervention aimed at reducing digestible carbohydrate intake to <40 g/day led to reductions in weight and improvements in cardiovascular risk factors among individuals with obesity.[30] Additionally, restriction of carbohydrate intake to <50g/day is sufficient to induce ketosis.[99] Our rationale for having a multi-phased intervention is to allow participants to see how they feel on the more restrictive diet, and see how they respond to small increases in carbohydrate consumption. A similar technique was applied in the DIETFITS published study.[100] The higher target of 60 net grams may be more sustainable long-term and also allows for consumption of healthy higher-carbohydrate foods, such as berries, legumes, beans, and limited whole grains.

8.B. Usual Diet Group

We will provide participants randomized to the usual diet group written information with standard dietary advice. Participants randomized to the usual diet group will not receive ongoing dietary recommendations. Moreover, they will continue to receive clinical care recommendations from their physician(s). To encourage continued participation in the study and minimize the difference in participant contact with study staff, participants will be offered optional monthly educational sessions on topics unrelated to the study intervention, such as retirement planning or health insurance options.

In another effort to encourage continued participation, patients randomized to the usual diet group will be offered a copy of all available intervention materials, as well as an optional one-hour counseling session with the interventionist following their final study visit.

8.C. Physical Activity

At baseline, both groups will receive written information in study materials with standard physical activity recommendations.

8.D. Dietary Adherence

Strategies for measuring compliance: For the low-carbohydrate diet, adherence will be assessed through measurement of urinary ketones at 3-month visit and 6-month TV. Study staff will make all reasonable effort to maintain adherence of participants to the visit schedule and diet intervention. Walnut consumption will be tracked throughout the study in the low-carbohydrate diet group.

Strategies for improving compliance:

- At the screening visit, we will ask participants who does most of the cooking in their households and how often they eat outside of the home. Results from the screening visit will help to select a group of cooperative study participants.
- Visits will be scheduled at the participants' convenience and to minimize waiting time and trips to the clinic; reminder calls, texts or emails (chosen based on participant preference) will also be made prior to clinic visits.
- We will hire enthusiastic staff who have good interpersonal skills.
- Participants will receive $40 gift cards for use at food stores or retailers when they complete each study visit, for a maximum of $120 over the study period.
- Participants will receive $25 gift cards when they return the stool specimens, for a maximum of $50 over the study period.
- Participants will be offered small gifts (such as pens or magnets with study logo) to encourage compliance and adherence to the study protocol.
- Participants will be provided with recipes, shopping lists, and food samples for the intervention diet.

9. Safety of Dietary Intervention

On the low-carbohydrate diet, participants will be encouraged to primarily focus on replacing carbohydrates (particularly highly refined carbohydrates) with foods such as non-starchy vegetables, olive oil and other plant-based unsaturated oils, nuts and seeds, avocados, eggs, seafood, tofu, poultry, and lean meat. Consumption of more unsaturated fats, protein, and fiber will be recommended. Participants will not be encouraged to increase consumption of saturated fats and will be advised to avoid consumption of trans fats.

In animal studies, a high-protein diet has decreased renal function[101] In the MACRO study, on average, participants tended to increase consumption of fats, rather than protein.[30] The safety and tolerability of low-carbohydrate diets have previously been assessed in many studies, including MACRO.[30] A number of studies, including some in individuals with T2D, have found no clinically significant increases in estimated glomerular filtration rates,[33,38–40] microalbuminuria,[102] albumin excretion rate,[33,38] urinary albumin,[38] serum creatinine,[38,40,102–107] albumin,[40] or uric acid levels.[103–106] A small study of a very low-carbohydrate diet found significant increase in uric acid excretion, but no significant change in 24-hour urine creatinine clearance.[106]

Study staff will carefully monitor participants' BP, and HbA1c more often than what would occur in routine healthcare visits. A Data Safety and Monitoring Board (DSMB) consisting of an independent group of experts in areas such as biostatistics, nutrition medicine, general internal medicine, and endocrinology has been formed. The board will regularly review trial progress, including unblinded interim results, and can recommend that the trial prematurely end if participants are at risk or if it becomes clear that the trial will not be able to yield statistically significant results.

10. Data Collection

10.A. Study visits

After the initial pre-screening interview, in which data on medications and medical conditions will be collected, there will be one screening visit, one randomization visit, a follow-up study visit at 3 months and a termination visit (TV) at 6 months. *Table 3* summarizes study procedures and data collected at each study visit.

Table 3. Study visit schedule and data collection

	Pre-screen	SV	RZ	3 month	6 month TV
Pre-screen eligibility	X				
Informed consent		X			
Compliance form		X			
Eligibility confirmation			X		
Randomization			X		
Demographics		X			
Medical history questionnaire			X	X	X
Medications		X	X	X	X
Lifestyle (alcohol, cigarette smoking)			X	X	X
Physical activity			X	X	X
24-hour diet recall*			X	X	X
3 BP measurements		X	X	X	X
Body weight			X	X	X
Height			X		
Waist circumference			X	X	X
Spot urine test			X	X	X
Blood samples		X	X	X	X
Stool specimen			X		X
Side-effects assessment			X	X	X
Nut Consumption					X

*Two 24-hour dietary recalls will be collected; one should cover a weekend and the other a weekday.

10.A.1. Screening/baseline visits:

Participants will be pre-screened by phone or in-person by study staff to confirm eligibility for the Screening Visit. An approved questionnaire will be used to ensure the patient is eligible to attend the in-clinic screening visit, and the patient will be scheduled thereafter.

At the screening visit (SV), participants will provide informed consent for the screening and main study. Study staff will schedule the SV and randomization (RZ) visit at least 2 days apart. If a participant is eligible based on the screening visit, he or she should be scheduled for the RZ

once a sufficient group of eligible individuals has been recruited. Participants should be scheduled for the RZ at least 2 days and no more than two weeks after the screening visit.

At the screening visit, we will collect information on inclusion/exclusion criteria, medication use, and demographics. At this visit, we will also have participants complete a compliance form, to assess their ability to complete the study and attend all intervention and study visits. We will also measure blood pressure, and take blood samples. Women will be asked if they are postmenopausal or if they had surgical sterilization (such as a tubal ligation). If not, they will be tested for pregnancy through a spot urine test; pregnant women will be excluded from the study. Blood samples will be collected for measurement of HbA1c, and, if not available from medical records in the past 3 months, serum creatinine and hemoglobin. These blood samples will be used for excluding individuals with HbA1c <6.0 or >6.9, eGFR ≤45 mL/min/1.73 m^2, or hemoglobin <11 mg/dL.

Before the start of the intervention, participants will complete two 24-hour dietary recalls. At least one (and potentially both) 24-h recalls will be collected by phone. At the RZ, participants will learn about the randomization process. After requirements for eligibility have been satisfied, participants will formally be enrolled into the study and randomized to either the active intervention or control group. We will collect fasting blood samples for later measurement of glucose, insulin and lipids and other potential future measurements. At this visit, we will measure blood pressure, body weight, height, and waist circumference. We will collect information on medical history, lifestyle risk factors, and physical activity. A spot urine sample will be collected to measure ketones and other markers and participants will be asked to collect a stool specimen for potential use in future microbiome studies.

10.A.2. Follow-up visits and final visit: A follow-up visit will occur at 3 months after randomization. A final visit will occur at 6-months post-randomization. These visits should be scheduled as close as possible to the 3-, or 6-months after randomization. At each visit, study staff will conduct dietary recalls and anthropometric measures, take 3 BP measurements, collect a fasting blood sample to measure HbA1c. At least one (and potentially both) 24-h recalls will be collected by phone. We will also collect samples for later measurement of glucose, insulin, lipid profile, and other potential future measurements. A spot urine sample will be collected to measure ketones and other markers. At the 6-month TV, participants will be asked to collect a stool specimen for potential use in future microbiome studies and information about participants' nut consumption over the past 6 months will be collected. At study termination, we will mail participants and/or their physicians a summary of trial results and a record of participant BP, anthropometric measures, and laboratory data from the trial period. Throughout the study period, we will provide participants with their blood pressure and weight. We will request that, if possible, a participant who is prescribed a diabetes medication during the course of the study comes into the clinic for an additional separate supplemental visit for a fasting blood sample collection prior to starting the medication.

10.B Study measurements

Measurements obtained for this study will be based on those used in other clinical trials and epidemiologic studies. We are familiar with strengths and limitations of such measurements and the importance of staff training and certification and use of stringent quality control procedures.

10.B.1. Questionnaires: Study staff will administer a *medical history questionnaire* at the RZ, 3-month visit and 6-month TV to assess medical conditions that would necessitate changes in therapy or medical management beyond this study's scope. At the SV, *demographic factors* will be collected. At the SV, RZ, 3-month and 6-month TV, we will also administer *medication*

tracking questionnaires. At RZ, 3-month, and 6-month TV, we will collect health behavior and physical activity data.

10.B.2. Dietary measurements: Two 24-hour dietary recalls will be obtained from participants at baseline. At or close to both the 3-month and 6-month TV, two more 24-hour dietary recalls will be collected. The second sets of two recalls will be collected as closely as possible to 3- or 6-month dates after randomization, with a goal of being within 2 weeks of the respective follow-up period. For each time point, one recall should reflect week day consumption, while the other should reflect weekend day consumption and the two recalls should be at least 2 days apart from each other. We will use computer software (Nutrition Data System for Research [NDS-R]) and food models used in previous and ongoing studies. Recalls will be conducted by a study staff member who has been certified in the use of NDS-R. Food composition tables of the NDS-R will be used to calculate dietary nutrient intakes. The baseline dietary assessment will be done to characterize individual dietary intake of carbohydrates and other macronutrients. During the intervention, repeated measurements will be used to monitor short-term changes in mean dietary nutrient intake between the intervention and usual diet groups.

10.B.3. Assessment and management of adverse effects: At RZ, 3-month visit, and 6-month TV, we will administer *symptom questionnaires*, which will consist of a checklist to identify participant complaints. Prior studies of low-carbohydrate dietary interventions suggest that participants may experience a range of adverse effects, including constipation, halitosis, fatigue, headache, thirst, polyuria, and nausea. Severe adverse events will be promptly reported to the DSMB. Management of adverse effects will be based on the protection and safety of the participant. If severe adverse effects are reported by a participant, carbohydrate intake will be examined and altered in order to mitigate the adverse effect. In any extreme cases, participants will be instructed to stop the intervention. As this is a behavioral intervention, adverse effects should be rare.

10.B.4. BP and anthropometric measures: BP will be measured 3 times at the screening visit and at each follow-up visit. To minimize random error, averages of the measurements at the screening and randomization visits will be taken. BP will be measured by a standard automated blood pressure measurement device (OMRON HEM-907 XL Professional Digital Blood Pressure Monitor) and performed by experienced staff, trained and certified in BP measurement. Prior to BP measurements, participants will be asked to rest quietly in a seated position with their feet supported for at least 5 minutes; staff members will use an appropriate cuff size over a bare arm. Trained and certified staff will measure weight using a calibrated balance beam scale at the screening visits, and follow-up visits. Weight and height will be measured at the RZ, and weight will also be measured at the subsequent visits.

10.B.5. Blood samples: For SV, participants will not need to fast. For RZ, 3-month visit, and 6-month TV, participants will be instructed to fast for 12 hours prior to blood sample collection. At SV, blood samples will be collected to measure HbA1c, hemoglobin, and serum creatinine. At RZ, fasting blood samples will be collected and stored as serum/plasma, whole blood aliquots and buffy coats at -80°C for future analyses, including measurement of glucose, insulin and lipids, and potential future metabolomics or genetic analyses. At 3-month visit and 6-month TV, we will collect fasting blood samples to measure HbA1c and will also store additional serum/plasma, whole blood aliquots and buffy coats at -80°C for future analyses, including measurement of glucose, insulin and lipids, and potential future metabolomics or genetic analyses. Details of the blood sample collection are shown in *Table 4*. HOMA-IR will be calculated from fasting glucose and insulin.

Table 4. Study visit schedule for specimen collection

	SV	RZ	3 month	6 month TV

HbA1c	X		X	X
Serum creatinine	X			
Hemoglobin	X			
Storage of aliquots and buffy coats (for measurement of glucose, insulin, lipids, other potential future measurements)		X	X	X
Urine sample for pregnancy	X			
Urine sample for ketones and other markers		X	X	X
Stool sample		X		X

10.B.6. Urine collection: Study staff will collect spot urine specimens at RZ, interim visit, and the final visit to measure ketones.

10.B.7. Stool Specimen Collection: Stool samples will be collected from all eligible and willing trial participants during the RZ and 6-month TV, following the protocol set forth by the Human Microbiome Project.[108,109] Participants will be given a stool collection kit, which includes the specimen collection container, a shipper for home collection, and a form to record the timing and pertinent information related to specimen collection. Participants will be instructed to return the kit to the clinic within two days of collection (in-person or via shipping), at which time stool samples are stored at -80°C until analyses. Kits and instructions for collection will be reviewed by study staff with the participant at the required visits.

11. Quality Assurance and Quality Control

Throughout the study, we will implement rigorous quality assurance (QA) and quality control (QC) measures. We describe some of these approaches here.

11.A. Manual of procedures

We will develop a manual of procedures (MOP) based on the MOP successfully used in the MACRO behavior change clinical trial. The MOP will provide instructions and describe procedures for staff training and certification, participant recruitment, intervention counseling, study form completion and procedures, data entry, safety measures (e.g., methods for emergency participant/primary care physician contact).

11.B. Training and certification

Trial personnel will all be required to participate in a training session before the start of any trial procedures or measurements. Training will include particular attention to core trial elements, such as recruitment, retention, intervention, completeness and quality of data acquisition (e.g., blinding procedures for the data collectors), and data entry. Separately, intervention staff will meet and receive detailed instruction on trial approaches to group and individual behavior change counseling and procedures that ensure data collectors are blinded to treatment assignment. Throughout the trial, periodic re-training/certification sessions will be conducted to ensure all trial-related procedures are being applied in a standard manner. Investigators will also monitor all aspects of study performance to ensure study goal

achievement, timeliness and completeness of study visits, completeness and quality of data collection, intervention adherence, and adequacy of the procedures separating data collection from intervention. Study staff and investigators will pay particularly careful attention to the quality of all laboratory measurements, particularly key exposure, outcome, and intervention monitoring variables.

12. Sample Size and Statistical Power

The primary outcome will be the difference in the change of HbA1c between the intervention and usual diet groups from baseline to 6 months of follow-up. In the United States National Health and Nutrition Examination Survey, when sub-setting to the range of HbA1c of 6.0–6.9% among individuals who do not report use of diabetes medication, the cross-sectional standard deviation is 0.24% using data from 2000 through 2016 (n=4117; https://wwwn.cdc.gov/nchs/nhanes/). In studies with 6-month follow-up among individuals with HbA1c 5.7 to 6.4% or other characteristic(s) (e.g, elevated fasting glucose or metabolic syndrome) that put them at elevated risk of type 2 diabetes, the within-person correlation between baseline and follow-up HbA1c was estimated to be between 0.5 and 0.84.[110–113] As all participants in our study will be within a specific range of HbA1c at baseline, we expect this may lead to smaller within-person correlation of HbA1c over time than studies that do not restrict to a narrow range of HbA1c at baseline. Assuming a within-person correlation of 0.5 and an SD of 0.24% for change in HbA1c, we would need to have 90 individuals to have 85% power to detect an approximate difference in change of HbA1c of 0.15% between the intervention and usual diet groups.

Table 5. Minimum detectable differences

	SD	Detectable Difference
HbA1c, %	0.24	0.15
Fasting glucose, mg/dL	12.5	8.0
Fasting insulin, mU/mL	7.0	4.5
HOMA-IR	2.0	1.3
SBP (mmHg)	4.2	2.7
DBP (mmHg)	3.0	1.9
Total/HDL ratio	1.2	0.77
Body weight (kg)	6.6	4.2
Waist circumference (cm)	8.0	5.1
Estimated CVD risk (%)*	6.7	4.8

*10-y CVD risk, among estimated 85% or 76 participants free of CVD

In a follow-up analysis of the DPP, a 0.15% decrement in HbA1c at 6 months was associated with a 16% lower risk of incident T2D, adjusting for treatment arm, age, sex, race/ethnicity, household income, and baseline HbA1c.[115] In a pooled analysis from the Atherosclerosis Risk in Communities and Framingham Heart Studies, a 0.15% higher HbA1c was associated with an 16% higher risk of T2D over 20 years, a 30% higher risk over 8 years, and a 13% higher risk after 8 years, after adjusting for age, sex, fasting glucose, triglycerides, HDL, family history of T2D, SBP, and BMI.[116] Additionally, in EPIC-Norfork, a difference of HbA1c of 0.15% was associated with approximately a 5% higher risk of CHD among those free of CHD, stroke, diabetes, and with HbA1c <7.0%.[117]

In the DPP study, HbA1c was 0.15% lower among those in the lifestyle intervention arm than in the control arm at 6-months follow-up; HbA1c at study commencement was 5.9% and levels increased in the control arm and decreased in the behavioral intervention arm.[22] In a small behavioral intervention study (n=34) of a very low-carbohydrate ketogenic diet compared with a moderate carbohydrate, calorie-restricted, low-fat diet among individuals with type 2 diabetes or prediabetes (HbA1c >6.0%) and elevated body weight (BMI >25), mean HbA1c decreased from 6.6% to 6.1% in the very low-carbohydrate group and from 6.9% to 6.7% in the moderate carbohydrate group over a 12-month period; additionally, the very low-carbohydrate group had greater reductions in glucose-lowering medications.[37]

Detectable differences are listed for HbA1c and secondary outcomes in *Table 5*, based on data from prior studies.[30,53,56,118] These were calculated for unpaired t-tests, power of 85%, two-sided alpha of 0.05, and assuming 80% follow-up of 112 participants, which would yield an estimated 90 participants.

13. Data Management and Analysis

13.A. Data management

Personnel will double-enter all study data. Prior to entering data, the study coordinator will conduct quality control on all study forms, study measures, and laboratory data. Data will be cleaned and logically checked.

13.B. Data analysis plan

The data will be analyzed using *intention to treat*; this involves comparing the primary and secondary outcomes between participants based on randomization assignment.

13.B.1. Exploratory data analysis: We will express group data for variables with a normal distribution as mean ± standard deviation and for variables not normally distributed as median and interquartile ranges (or will log-transform the variables to obtain a normal distribution). We will compare means and percentages of baseline variables across the intervention and usual diet group to assess similarity of these groups at randomization.

13.B.2. Primary, secondary, and exploratory outcomes: We will test the primary study hypothesis that there is a greater reduction in HbA1c from baseline to 6 months in the intervention group than in the usual diet group. To do this, we will use a mixed-effects linear regression model, to test the null hypothesis that there is no difference in change between baseline and 6-month HbA1c for the intervention group compared with the usual diet group (Specific Aim 1). To do this, we will use the following model:

$$Y_{i,j,k}|T = b_0 + b_1G_i + b_2T_{3\text{-months}} + b_3T_{6\text{-months}} + b_4G_iT_{3\text{-months}} + b_5G_iT_{6\text{-months}} + a_{0i,j} + e_{i,j,k}$$

where $Y_{i,j,k}$ is HbA1c for the jth participant (P) nested in the ith intervention group (G) at time T_k (baseline, 3 months, and 6 months). This model includes a random intercept ($a_{0i,j}$) and error term $e_{i,j,k}$. The interaction term ($b_5G_iT_{6\text{-months}}$) allows for an interaction between group and $T_{6\text{-months}}$, which is equal to 0 for baseline and 3-months and 1 for 6-months. Rejection of the null hypothesis (b_5=0) indicates there is a significant difference in change of HbA1c from baseline to 6 months between the intervention and control groups. In this model, participants are assumed to be random effects and intervention group, time, and the interaction terms are fixed effects.

We will use a similar approach to test the difference in the change between baseline and 6-month values of secondary outcomes (Specific Aim 2) and exploratory outcomes. In exploratory analyses, we will also test the difference in the change between baseline and 3-month values of outcomes (HbA1c, fasting glucose, systolic blood pressure, total-to-HDL cholesterol, body weight, insulin, HOMA-IR, diastolic blood pressure, waist circumference, and estimated CVD risk). We will also explore the use of mixed effects models to assess overall net change in HbA1c (by treating time as a linear variable) and other outcomes over the full study period. For each outcome, we will test modeling assumptions and will consider transformation of outcomes, if needed, to meet modeling assumptions. As the mixed-effects model assumes missing at random, we will also perform sensitivity analyses using Markov-chain Monte Carlo techniques to impute missing values of the outcomes, using covariates for this imputation and will compare results from this analysis with our primary findings.

15. Anticipated Challenges

Recruitment: Many clinical trials have challenges recruiting sufficient numbers of volunteers who meet the trial inclusion and exclusion criteria and demonstrate a high level of adherence to trial protocol requirements. Fortunately, the investigators are very experienced with clinical trial recruitment, and have several contingency options that can be used to meet unanticipated shortfall in recruitment.

Intervention: The investigators are aware of and understand challenges related to achieving and maintaining compliance with a behavioral intervention. The investigators have much experience and success in behavioral interventions, with recent success of a behavioral intervention aimed at reducing carbohydrate intake among participants from the New Orleans area. The JFI will collaborate with Drs. He and Bazzano to develop the intervention program based on the MACRO study and will assess potential barriers to implementation and intervention adherence (such as financial concerns and range of dietary patterns of study participants) during the planning and pilot phase of the study.

Adherence: Though adherence and follow-up are also main challenges related to clinical trials, the investigators have an outstanding track record for studies that have recruited participants for both clinical and epidemiology studies in the Greater New Orleans area. To encourage adherence, small gifts (such as pens or magnets with study logo) will be offered to all study participants.

16. Protection of Human Subjects

This Human Subjects Research meets the definition of a clinical trial. All investigators will be required to complete the CITI (Collaborative Institutional Training Initiative) program in the ethical use of human participants in research.

16.A. Protection of human subjects from research risks

16.A.1. Risks to the subjects

Human subject involvement and characteristics: We expect to enroll 112 participants, with an equal male to female ratio. We expect to enroll approximately 30 participants who are African-Americans, and another 10 with mixed racial backgrounds, and a few Asian participants given the demographics of the greater New Orleans area. Overall, given the demographics of greater New Orleans, we estimate approximately 10% participants of Hispanic or Latino ethnicity, 50% of them with African-American racial background.

Inclusion criteria

- Men or women ages 40 to 70 years: aim to recruit 50% women
- Any race or ethnicity: aim to recruit 40% ethnic minorities (i.e., African American and Hispanic), close to population in the greater New Orleans area[94]
- HbA1c 6.0–6.9%
- Willing and able to provide informed consent

Exclusion criteria

- Diagnosed type 1 diabetes mellitus based on patient self-report
- Use of agents affecting glycemic control (medications for diabetes, oral glucocorticoids) within the past three months prior to enrollment based on patient self-report
- Medical condition in which low-carbohydrate diet may not be advised

- Estimated glomerular filtration rate (eGFR) ≤45 mL/min/1.73 m^2, which is close to the 5th percentile of eGFR among non-diseased individuals of 70 years of age[95]
- Self-report of liver disease due to hepatitis or alcohol; osteoporosis; untreated thyroid disease; gout; or cancer (other than non-melanoma skin cancer) requiring treatment in the past year, unless prognosis is excellent

- Factors that may affect HbA1c:
  - Hemoglobin <11 mg/dL (cutpoint for moderate-to-severe anemia, which could lead to falsely elevated or lowered HbA1c)[96]
  - Recent blood donation or blood transfusion (self-report, past 4 months)
  - Human Immunodeficiency Virus (self-report)[97]
- Allergies to nuts
- For women, current pregnancy, breastfeeding, or plans to become pregnant during the study
- Consumption of ≥21 alcoholic drinks per week or consumption of ≥6 drinks per occasion
- Current or planned residence making it difficult to meet trial requirements (due to distance from study site and/or challenges regularly traveling to site)
- Current participation in another lifestyle intervention trial or a pharmaceutical trial
- Participation of another household member in the study; employees or persons living with employees of the study
- Other concerns regarding ability to meet trial requirements, at the discretion of the principal investigator or study coordinator

Sources of materials: Medical information data will be collected from the participant and, after written authorization for the disclosure of protected health information, from participants' medical records. Blood and urine samples will be collected to measure HbA1c, glucose, insulin, lipids, creatinine, ketones, and test for pregnancy. Blood pressure, height, weight, waist circumference and other physical measurements will be obtained.

Potential Risks: Risks associated with venipuncture, the diet intervention, and collection of confidential medical information are the main risks associated with this study. There is a small risk of bruising and a rare chance of local infection associated with standard venipuncture to collect blood samples. The dietary intervention has minimal risks. Reduced carbohydrate consumption may lead to adverse effects, including constipation, halitosis, fatigue, headache, thirst, polyuria, and nausea. At RZ, 3- , and 6-month visits, we will administer *symptom questionnaires*, which will consist of a checklist to identify participant complaints. Adverse effects of the low-carbohydrate diet will be assessed at counseling sessions. Severe adverse events will be promptly reported to the DSMB. Management of adverse effects will be based on the protection and safety of the participant. If severe adverse effects are reported by a participant, carbohydrate intake will be examined and altered in order to mitigate the adverse effect. In any extreme cases, participants will be instructed to stop the intervention. Furthermore, there is a potential risk of unauthorized disclosure of medical information. This risk will be minimized by using a strict protocol for data processing and by the appropriate training of all study personnel. Protection against risks is described in more detail below.

16.A.2. Adequacy of protection against risks

Recruitment and Informed Consent: The proposed study will be conducted following strict guidelines for the protection of the rights of human volunteers. The informed consent document

will be signed by all participants at the screening/baseline visit. The informed consent form will be developed in the first year of the study and approved by the IRB. The consent form will clearly state the purpose, eligibility criteria, and protocol of the study, the potential benefits and risks of participation, and the subject's right to refuse to participate or withdraw. Prior to signing the informed consent, research staff will review the details of the consent form orally with the participant, and answer any questions that the participant has concerning participation in the study. In the case of potential participants with low or no literacy, the informed consent form will be read to the participant in its entirety and any questions will be answered prior to the participant signing or marking the form. Specifically, the following must be accomplished during the informed consent process:

- The participant must be informed that participation in the study is voluntary and that refusal to participate will involve no penalty or loss of benefits.
- The participant must be informed of the purpose of the study and that it involves research.
- The participant must be informed of any alternative procedures, if applicable.
- The participant must be informed of any reasonably foreseeable risks.
- The participant must be informed of any benefits from the research.
- An outline of safeguards to protect participant confidentiality will be included, as well as an indication of the participant’s right to withdraw without penalty. This should be balanced with a discussion of the effect that withdrawals will have on the study, and the responsibility a participant has, within limits, to continue in the study if he or she decides to enroll.
- The participant must be informed of his or her right to have questions answered at any time and whom to contact for answers or in the event of research-related injury.
- The participant must be informed as to whether or not compensation is offered for participation in the study and/or in the event of a medical injury.
- The participant must be informed that he/she will be notified of any significant changes in the protocol that might affect their willingness to continue in the study.

Written authorization for the disclosure of protected health information will be obtained during the consent process on a separate form (HIPPA authorization), if applicable.

Protection against Risk: The protocol and informed consent will be approved by the Institutional Review Boards at Tulane University Health Sciences Center and any other participating institution for recruitment. The study participants will be given contact information for the Study Coordinator and Study PI to answer questions or to report complaints. Patients can withdraw from the study at any time without any effect on their medical care. Standard techniques will be used by trained, certified phlebotomists or study nurses for blood sample collection at the study clinics where emergency medical equipment is available. Confidentiality of all data collected during the study will be maintained through the use of unique, encrypted study identification (ID numbers, rather than patient names, in the study database). These encrypted ID numbers will not be mathematical derivation of the subject’s medical record number or any other patient identifier. No information will be disclosed in an individually identifiable form in any type of presentation or publication. There will be password-required access to the computerized file linking the study numbers to patient identifiers at the study clinics. Password-access will be restricted to pertinent research staff only. All study data will be kept in locked file cabinets in locked rooms, accessible only by study staff with permission. All data transmissions will use HIPPA-compliant password protected system and will be secured through a virtual private network.

16.A.3. Potential benefits of the proposed research to human subjects and others

Study participants may have improved glycemic outcomes, blood pressure, lipid levels, may lose weight and reduce waist circumference, and may have improvement in other risk factors for CVD and development and progression of T2D. Participants will receive multiple blood pressure, weight, waist circumference, and blood tests. If vital signs or lab values are clinically abnormal, participants will be referred to their primary care physician, or if they do not have a primary care physician, one will be assigned through the Tulane University Community Health Center, which is a part of the core clinical facilities. At the end of the study, we will provide the participants' primary care physician with a copy of all the blood pressure, weight, and laboratory results.

16.A.4. Importance of knowledge to be gained

Information from this study may contribute to reducing the onset of T2D and the burden of T2D and cardiovascular disease in Louisiana and the general US population. The study has potential for public health impact; results from this study may provide evidence in support of promoting alternative dietary styles (than the most commonly used low-fat diet) among individuals with high T2D risk or with T2D.

16.A.5. Remuneration

For screening visit 2, the two follow-up visits, and the final visits, participants will receive $20 gift cards for use at food stores or retailers (after completing each study visit), for a maximum of $60 over the study period. Participants who return a stool sample will receive $25 gift cards for each stool sample that they return, for a total of $50 for two stool samples. Participants will also receive free parking for each intervention and study visit.

16.B. Data safety and monitoring plan

A DSMB, which consists of 3 experts who are not otherwise affiliated with the COBRE program, has been formed. Study investigators for this study will prepare accurate and timely data tables and reports for the DSMB meetings. The members of the DSMB will serve in an individual capacity and provide their expertise and recommendations. The primary responsibilities of the DSMB are: 1) periodically review and evaluate the accumulated study data for participant safety, study conduct and progress, and, when appropriate, efficacy, and 2) make recommendations to the NIH concerning the continuation, modification, or termination of the trial. The DSMB considers study-specific data, as well as relevant background knowledge, about the disease, test agent, and patient population under study.

The DSMB is responsible for defining its deliberative processes, including event triggers that would call for an unscheduled review, stopping guidelines, unmasking (unblinding) and voting procedures prior to initiating any data review. The DSMB is also responsible for maintaining the confidentiality of its internal discussions and activities, as well as the contents of reports provided to it.

The DSMB will review each protocol for any major concerns prior to implementation. During the trial, the DSMB should review cumulative study data to evaluate safety, study conduct, and scientific validity and integrity of the trial. As part of this responsibility, DSMB members must be satisfied that the timeliness, completeness, and accuracy of the data submitted to them for review are sufficient for evaluation of the safety and welfare of study participants. The DSMB should also assess the performance of overall study operations and any other relevant issues, as necessary.

Items reviewed by the DSMB include:

- Interim/cumulative data for evidence of study-related adverse events;
- Interim/cumulative data for evidence of efficacy according to pre-established statistical guidelines, if appropriate;
- Data quality, completeness, and timeliness;
- Adequacy of compliance with goals for recruitment and retention, including those related to the participation of women and minorities;
- Adherence to the protocol;
- Factors that might affect the study outcome or compromise the confidentiality of the trial data (such as protocol violations, unmasking, etc.); and,
- Factors external to the study, such as scientific or therapeutic developments, that may impact participant safety or the ethics of the study.

The DSMB will conclude each review with their recommendations to the COBRE program and NIH/NIGMS as to whether the study should continue without change or be modified or terminated. Recommendations regarding modification of the design and conduct of the study could include:

- Modifications of the study protocol based upon review of the safety data;
- Suspension or early termination of the study or of one or more study arms because of serious concerns about subjects' safety, inadequate performance or rate of enrollment;
- Suspension or early termination of the study or of one or more study arms because study objectives have not been obtained according to pre-established statistical guidelines; and,
- Corrective actions regarding a study when the performance appears unsatisfactory or suspicious.

Confidentiality must always be maintained during all phases of DSMB review and deliberation. Usually, only voting members of the DSMB should have access to interim analyses of outcome data by treatment group. Exceptions may be made when the DSMB deems it appropriate. The reason and to whom the exceptions for access to interim analyses is granted will be documented in the Closed Session Report. DSMB members must maintain strict confidentiality concerning all privileged trial results ever provided to them. The DSMB should review data only by masked study group (such as X vs. Y rather than experimental vs. control) unless or until the DSMB determines that the identities of the groups are necessary for their decision-making. Whenever masked data are presented to the DSMB, the key to the group coding must be available for immediate unmasking.

The DSMB will meet twice per year. The junior faculty investigator will attend these meetings and will be responsible for preparing and presenting up-to-date statistical reports on the progress of their trials. These reports will include data on recruitment, randomization, adherence, as well as statistical tests and special analyses requested by the DSMB. The Administrative Core will be responsible for organizing the annual in-person meeting and semiannual conference calls. They will put together data and materials submitted by junior faculty investigators and deliver them to DSMB members. The COBRE investigators will meet with the DSMB in the Open Session and not be present for the Closed Session.

Bibliography and References Cited

1. The Emerging Risk Factors Collaboration. Diabetes mellitus, fasting blood glucose concentration, and risk of vascular disease: a collaborative meta-analysis of 102 prospective studies. *Lancet*. 2010;375(9733):2215-2222. doi:10.1016/S0140-6736(10)60484-9.
2. Flaxman SR, Bourne RRA, Resnikoff S, et al. Global causes of blindness and distance vision impairment 1990-2020: a systematic review and meta-analysis. *Lancet Glob Heal*. October 2017. doi:10.1016/S2214-109X(17)30393-5.
3. United States Renal Data System. *2016 USRDS Annual Data Report: Epidemiology of Kidney Disease in the United States*. Bethesda, MD: National Institutes of Health, National Institute of Diabetes and Digestive and Kidney Diseases; 2016. https://www.usrds.org/2016/view/Default.aspx.
4. Narres M, Kvitkina T, Claessen H, et al. Incidence of lower extremity amputations in the diabetic compared with the non-diabetic population: A systematic review. *PLoS One*. 2017;12(8):e0182081. doi:10.1371/journal.pone.0182081.
5. Centers for Disease Control and Prevention. *National Diabetes Statistics Report, 2017*. Atlanta, GA: Centers for Disease Control and Prevention, U.S. Dept of Health and Human Services; 2017.
6. Centers for Disease Control and Prevention. *Long-Term Trends in Diabetes*. Atlanta, GA: Centers for Disease Control and Prevention, U.S. Dept of Health and Human Services; 2017.
7. NCD Risk Factor Collaboration (NCD-RisC). Worldwide trends in diabetes since 1980: A pooled analysis of 751 population-based studies with 4.4 million participants. *Lancet*. 2016;387(10027):1513-1530. doi:10.1016/S0140-6736(16)00618-8.
8. American Diabetes Association. Economic costs of diabetes in the U.S. in 2012. *Diabetes Care*. 2013;36(4):1033-1046. doi:10.2337/dc12-2625.
9. Zhuo X, Zhang P, Barker L, Albright A, Thompson TJ, Gregg E. The lifetime cost of diabetes and its implications for diabetes prevention. *Diabetes Care*. 2014;37(9):2557-2564. doi:10.2337/dc13-2484.
10. WHO, International Diabetes Foundation. *Definition and Diagnosis of Diabetes Mellitus and Intermediate Hyperglycaemia: Report of a WHO/IDF Consultation*. Geneva: Geneva: World Health Organization; 2006.
11. The International Expert Committee. International Expert Committee Report on the Role of the A1C Assay in the Diagnosis of Diabetes. *Diabetes Care*. 2009;32(7):1327-1334. doi:10.2337/dc09-9033.
12. American Diabetes Association. Standards of Medical Care in Diabetes - 2017. *Diabetes Care*. 2017;40(Supplement 1):S33-S43. doi:10.2337/dc16-S003.
13. Gerstein HC, Santaguida P, Raina P, et al. Annual incidence and relative risk of diabetes in people with various categories of dysglycemia: A systematic overview and meta-analysis of prospective studies. *Diabetes Res Clin Pract*. 2007;78(3):305-312. doi:10.1016/j.diabres.2007.05.004.
14. Huang Y, Cai X, Mai W, Li M, Hu Y. Association between prediabetes and risk of cardiovascular disease and all cause mortality: systematic review and meta-analysis. *BMJ*. 2016;355:i5953. doi:10.1136/bmj.i5953.
15. Tabák AG, Herder C, Rathmann W, Brunner EJ, Kivimäki M. Prediabetes: A high-risk state for diabetes development. *Lancet*. 2012;379(9833):2279-2290. doi:10.1016/S0140-

6736(12)60283-9.

16. Diabetes Prevention Program Research Group. 10-year follow-up of diabetes incidence and weight loss in the Diabetes Prevention Program Outcomes Study. *Lancet*. 2009;374(9702):1677-1686. doi:10.1016/S0140-6736(09)61457-4.
17. The DREAM (Diabetes REduction Assessment with ramipril and rosiglitazone Medication) Trial Investigators. Effect of rosiglitazone on the frequency of diabetes in patients with impaired glucose tolerance or impaired fasting glucose: a randomised controlled trial. *Lancet*. 2006;368(9541):1096-1105. doi:10.1016/S0140-6736(06)69420-8.
18. Perreault L, Pan Q, Mather KJ, Watson KE, Hamman RF, Kahn SE. Effect of regression from prediabetes to normal glucose regulation on long-term reduction in diabetes risk: Results from the Diabetes Prevention Program Outcomes Study. *Lancet*. 2012;379(9833):2243-2251. doi:10.1016/S0140-6736(12)60525-X.
19. Perreault L, Temprosa M, Mather KJ, et al. Regression from prediabetes to normal glucose regulation is associated with reduction in cardiovascular risk: Results from the diabetes prevention program outcomes study. *Diabetes Care*. 2014;37(9):2622-2631. doi:10.2337/dc14-0656.
20. Popkin BM. Nutrition Transition and the Global Diabetes Epidemic. *Curr Diab Rep*. 2015;15(9). doi:10.1007/s11892-015-0631-4.
21. Vazquez G, Duval S, Jacobs DRJ, Silventoinen K. Comparison of body mass index, waist circumference, and waist/hip ratio in predicting incident diabetes: a meta-analysis. *Epidemiol Rev*. 2007;29:115-128. doi:10.1093/epirev/mxm008.
22. Knowler WC, Barrett-Connor E, Fowler SE, et al. Reduction in the incidence of type 2 diabetes with lifestyle intervention or metformin. *N Engl J Med*. 2002;346(6):393-403. doi:10.1056/NEJMoa012512.
23. Pan XR, Li GW, Hu YH, et al. Effects of diet and exercise in preventing NIDDM in people with impaired glucose tolerance: The Da Qing IGT and diabetes study. *Diabetes Care*. 1997;20(4):537-544. doi:10.2337/diacare.20.4.537.
24. Tuomilehto J, Indstrom J, Eriksson J, Valle T, Hamalainine E, Uusitupa M. Prevention of Type 2 Diabetes Mellitus By Changes in Lifestyle Among Subjects With Impaired Glucose Tolerance. *N Engl J Med*. 2001;344(18):1343-1350. doi:10.1056/NEJM200105033441801.
25. Ramachandran A, Snehalatha C, Mary S, Mukesh B, Bhaskar AD, Vijay V. The Indian Diabetes Prevention Programme shows that lifestyle modification and metformin prevent type 2 diabetes in Asian Indian subjects with impaired glucose tolerance (IDPP-1). *Diabetologia*. 2006;49(2):289-297. doi:10.1007/s00125-005-0097-z.
26. The Diabetes Preventio Program Research Group. The Diabetes Prevention Program. Design and methods for a clinical trial in the prevention of type 2 diabetes. *Diabetes Care*. 1999;22(4):623-634. doi:10.2337/diacare.22.4.623 10.2337/diabetes.48.4.699.
27. Evert AB, Boucher JL, Cypress M, et al. Nutrition therapy recommendations for the management of adults with diabetes. *Diabetes Care*. 2013;36(11):3821-3842. doi:10.2337/dc13-2042.
28. Hu T, Mills KT, Yao L, et al. Effects of low-carbohydrate diets versus low-fat diets on metabolic risk factors: A meta-analysis of randomized controlled clinical trials. *Am J Epidemiol*. 2012;176(SUPPL. 7). doi:10.1093/aje/kws264.
29. Sackner-Bernstein J, Kanter D, Kaul S. Dietary Intervention for Overweight and Obese Adults: Comparison of Low-Carbohydrate and Low-Fat Diets. A Meta-Analysis. *PLoS*

One. 2015;10(10):e0139817. doi:10.1371/journal.pone.0139817.

30. Bazzano LA, Hu T, Reynolds K, et al. Effects of low-carbohydrate and low-fat diets: A randomized trial. *Ann Intern Med*. 2014;161(5):309-318. doi:10.7326/M14-0180.
31. Sawyer L, Gale EAM. Diet, delusion and diabetes. *Diabetologia*. 2009;52(1):1-7. doi:10.1007/s00125-008-1203-9.
32. Snorgaard O, Poulsen GM, Andersen HK, Astrup A. Systematic review and meta-analysis of dietary carbohydrate restriction in patients with type 2 diabetes. *BMJ Open Diabetes Res Care*. 2017;5(1):e000354. doi:10.1136/bmjdrc-2016-000354.
33. Larsen RN, Mann NJ, Maclean E, Shaw JE. The effect of high-protein, low-carbohydrate diets in the treatment of type 2 diabetes: A 12 month randomised controlled trial. *Diabetologia*. 2011;54(4):731-740. doi:10.1007/s00125-010-2027-y.
34. Saslow LR, Kim S, Daubenmier JJ, et al. A randomized pilot trial of a moderate carbohydrate diet compared to a very low carbohydrate diet in overweight or obese individuals with type 2 diabetes mellitus or prediabetes. *PLoS One*. 2014;9(4). doi:10.1371/journal.pone.0091027.
35. Tay J, Luscombe-Marsh ND, Thompson CH, et al. A very low-carbohydrate, low-saturated fat diet for type 2 diabetes management: A randomized trial. *Diabetes Care*. 2014;37(11):2909-2918. doi:10.2337/dc14-0845.
36. Davis NJ, Tomuta N, Schechter C, et al. Comparative Study of the Effects of a 1-Year Dietary Intervention of a Low- Carbohydrate Diet Versus a Low-Fat Diet on Weight and Glycemic Control in Type 2. *Diabetes Care*. 2009;32(7):1147-1152. doi:10.2337/dc08-2108.Clinical.
37. Saslow LR, Daubenmier JJ, Moskowitz JT, et al. Twelve-month outcomes of a randomized trial of a moderate-carbohydrate versus very low-carbohydrate diet in overweight adults with type 2 diabetes mellitus or prediabetes. *Nutr Diabetes*. 2017;7(12):304. doi:10.1038/s41387-017-0006-9.
38. Tay J, Thompson CH, Luscombe-Marsh ND, et al. Effects of an energy-restricted low-carbohydrate, high unsaturated fat/low saturated fat diet versus a high carbohydrate, low fat diet in type 2 diabetes: a 2 year randomized clinical trial. *Diabetes, Obes Metab*. 2017:n/a-n/a. doi:10.1111/dom.13164.
39. Yamada Y, Uchida J, Izumi H, et al. A non-calorie-restricted low-carbohydrate diet is effective as an alternative therapy for patients with type 2 diabetes. *Intern Med*. 2014;53(1):13-19. doi:http://dx.doi.org/10.2169/internalmedicine.53.0861.
40. Mayer SB, Jeffreys AS, Olsen MK, Mcduffie JR, Feinglos MN, Yancy WS. Two diets with different haemoglobin A1c and antiglycaemic medication effects despite similar weight loss in type 2 diabetes. *Diabetes, Obes Metab*. 2014;16(1):90-93. doi:10.1111/dom.12191.
41. Maekawa S, Kawahara T, Nomura R, et al. Retrospective study on the efficacy of a low-carbohydrate diet for impaired glucose tolerance. *Diabetes Metab Syndr Obes*. 2014;7:195-201. doi:10.2147/DMSO.S62681.
42. Davis C, Bryan J, Hodgson J, Murphy K. Definition of the mediterranean diet: A literature review. *Nutrients*. 2015;7(11):9139-9153. doi:10.3390/nu7115459.
43. Sofi F, Macchi C, Abbate R, Gensini GF, Casini A. Mediterranean diet and health status: an updated meta-analysis and a proposal for a literature-based adherence score. *Public Health Nutr*. 2014;17(12):2769-2782. doi:10.1017/S1368980013003169.
44. Jannasch F, Kröger J, Schulze MB. Dietary Patterns and Type 2 Diabetes: A Systematic Literature Review and Meta-Analysis of Prospective Studies. *J Nutr*. 2017;147(6):1174-

1182. doi:10.3945/jn.116.242552.

45. Schwingshackl L, Missbach B, König J, Hoffmann G. Adherence to a Mediterranean diet and risk of diabetes: a systematic review and meta-analysis. *Public Health Nutr*. 2014;18(7):1292–1299. doi:10.1017/S1368980014001542.
46. de Lorgeril M, Salen P, Martin J-L, Monjaud I, Delaye J, Mamelle N. Mediterranean Diet, Traditional Risk Factors, and the Rate of Cardiovascular Complications After Myocardial Infarction : Final Report of the Lyon Diet Heart Study. *Circulation*. 1999;99(6):779-785. doi:10.1161/01.CIR.99.6.779.
47. Estruch R, Ros E, Salas-Salvadó J, et al. Primary Prevention of Cardiovascular Disease with a Mediterranean Diet. *N Engl J Med*. 2013;368(14):1279-1290. doi:10.1056/NEJMoa1200303.
48. Salas-Salvadó J, Bulló M, Estruch R, et al. Prevention of diabetes with mediterranean diets: A subgroup analysis of a randomized trial. *Ann Intern Med*. 2014;160(1):1-10. doi:10.7326/M13-1725.
49. Esposito K, Marfella R, Ciotola M, et al. Effect of a Mediterranean-Style Diet on Endothelial Dysfunction and Markers of Vascular Inflammation in the Metabolic Syndrome. *JAMA*. 2004;292(12):1440. doi:10.1001/jama.292.12.1440.
50. Babio N, Toledo E, Estruch R, et al. Mediterranean diets and metabolic syndrome status in the PREDIMED randomized trial. *CMAJ*. 2014;186(17):E649-E657. doi:10.1503/cmaj.140764.
51. Esposito K, Maiorino MI, Bellastella G, Chiodini P, Panagiotakos D, Giugliano D. A journey into a Mediterranean diet and type 2 diabetes: a systematic review with meta-analyses. *BMJ Open*. 2015;5(8):e008222. doi:10.1136/bmjopen-2015-008222.
52. Micha R, Michas G, Mozaffarian D. Unprocessed red and processed meats and risk of coronary artery disease and type 2 diabetes--an updated review of the evidence. *Curr Atheroscler Rep*. 2012;14(6):515-524. doi:10.1007/s11883-012-0282-8.
53. Esposito K, Maiorino MI, Ciotola M, et al. Effects of a Mediterranean-style diet on the need for antihyperglycemic drug therapy in patients with newly diagnosed type 2 diabetes: A randomized trial. *Ann Intern Med*. 2009;151(5):306-314. doi:10.1097/OGX.0b013e3181e5a159.
54. Esposito K, Maiorino MI, Petrizzo M, Bellastella G, Giugliano D. The effects of a Mediterranean diet on the need for diabetes drugs and remission of newly diagnosed type 2 diabetes: Follow-up of a randomized trial. *Diabetes Care*. 2014;37(7):1824-1830. doi:10.2337/dc13-2899.
55. Elhayany A, Lustman A, Abel R, Attal-Singer J, Vinker S. A low carbohydrate Mediterranean diet improves cardiovascular risk factors and diabetes control among overweight patients with type 2 diabetes mellitus: A 1-year prospective randomized intervention study. *Diabetes, Obes Metab*. 2010;12(3):204-209. doi:10.1111/j.1463-1326.2009.01151.x.
56. Shai I, Schwarzfuchs D, Henkin Y, et al. Weight Loss with a Low-Carbohydrate, Mediterranean, or Low-Fat Diet. *N Engl J Med*. 2008;359(3):229-241. doi:10.1056/NEJMoa0708681.
57. Schwarzfuchs D, Golan R, Shai I. Four-Year Follow-up after Two-Year Dietary Interventions. *N Engl J Med*. 2012;367(14):1373-1374. doi:10.1056/NEJMc1204792.
58. Pan A, Sun Q, Bernstein AM, et al. Red meat consumption and risk of type 2 diabetes: 3 Cohorts of US adults and an updated meta-analysis. *Am J Clin Nutr*. 2011;94(4):1088-1096. doi:10.3945/ajcn.111.018978.

59. Hu EA, Pan A, Malik V, Sun Q. White rice consumption and risk of type 2 diabetes: meta-analysis and systematic review. *BMJ*. 2012;344:e1454. doi:10.1136/bmj.e1454.

60. Wang M, Yu M, Fang L, Hu R-Y. Association between sugar-sweetened beverages and type 2 diabetes: A meta-analysis. *J Diabetes Investig*. 2015;6(3):360-366. doi:10.1111/jdi.12309.

61. Imamura F, O'Connor L, Ye Z, et al. Consumption of sugar sweetened beverages, artificially sweetened beverages, and fruit juice and incidence of type 2 diabetes: systematic review, meta-analysis, and estimation of population attributable fraction. *BMJ*. 2015:h3576. doi:10.1136/bmj.h3576.

62. Carter P, Gray LJ, Troughton J, Khunti K, Davies MJ. Fruit and vegetable intake and incidence of type 2 diabetes mellitus: systematic review and meta-analysis. *BMJ*. 2010;341:c4229. doi:10.1136/bmj.c4229.

63. Cooper AJ, Forouhi NG, Ye Z, et al. Fruit and vegetable intake and type 2 diabetes: EPIC-InterAct prospective study and meta-analysis. *Eur J Clin Nutr*. 2012;66(10):1082-1092. doi:10.1038/ejcn.2012.85.

64. Chen M, Sun Q, Giovannucci E, et al. Dairy consumption and risk of type 2 diabetes: 3 cohorts of US adults and an updated meta-analysis. *BMC Med*. 2014;12(1):215. doi:10.1186/s12916-014-0215-1.

65. Aune D, Norat T, Romundstad P, Vatten LJ. Whole grain and refined grain consumption and the risk of type 2 diabetes: A systematic review and dose-response meta-analysis of cohort studies. *Eur J Epidemiol*. 2013;28(11):845-858. doi:10.1007/s10654-013-9852-5.

66. Ding M, Bhupathiraju SN, Chen M, van Dam RM, Hu FB. Caffeinated and decaffeinated coffee consumption and risk of type 2 diabetes: a systematic review and a dose-response meta-analysis. *Diabetes Care*. 2014;37(2):569-586. doi:10.2337/dc13-1203.

67. Li X-H, Yu F-F, Zhou Y-H, He J. Association between alcohol consumption and the risk of incident type 2 diabetes: a systematic review and dose-response meta-analysis. *Am J Clin Nutr*. 2016;103(3):818-829. doi:10.3945/ajcn.115.114389.

68. Muraki I, Imamura F, Manson JE, et al. Fruit consumption and risk of type 2 diabetes: results from three prospective longitudinal cohort studies. *BMJ*. 2013;347(aug28 1):f5001-f5001. doi:10.1136/bmj.f5001.

69. Upadhyaya S, Banerjee G. Type 2 diabetes and gut microbiome: At the intersection of known and unknown. *Gut Microbes*. 2015;6(2):85-92. doi:10.1080/19490976.2015.1024918.

70. David LA, Maurice CF, Carmody RN, et al. Diet rapidly and reproducibly alters the human gut microbiome. *Nature*. 2014;505(7484):559-563. doi:10.1038/nature12820.

71. Duncan SH, Belenguer A, Holtrop G, Johnstone AM, Flint HJ, Lobley GE. Reduced dietary intake of carbohydrates by obese subjects results in decreased concentrations of butyrate and butyrate-producing bacteria in feces. *Appl Environ Microbiol*. 2007;73(4):1073-1078. doi:10.1128/AEM.02340-06.

72. Dorans KS, Mostofsky E, Levitan EB, Håkansson N, Wolk A, Mittleman MA. Alcohol and Incident Heart Failure among Middle-Aged and Elderly Men: Cohort of Swedish Men. *Circ Hear Fail*. 2015;8(3):422-427. doi:10.1161/CIRCHEARTFAILURE.114.001787.

73. Steinhaus DA, Mostofsky E, Levitan EB, et al. Chocolate intake and incidence of heart failure: Findings from the Cohort of Swedish Men. *Am Heart J*. 2017;183:18-23. doi:10.1016/j.ahj.2016.10.002.

74. Dorans KS, Massa J, Chitnis T, Ascherio A, Munger KL. Physical activity and the incidence of multiple sclerosis. *Neurology*. 2016;87(17):1770-1776.

doi:10.1212/WNL.0000000000003260.
75. Mills KT, Chen J, Yang W, et al. Sodium Excretion and the Risk of Cardiovascular Disease in Patients With Chronic Kidney Disease. *JAMA*. 2016;315(20):2200. doi:10.1001/jama.2016.4447.
76. Chen J, Gu D, Huang J, et al. Metabolic syndrome and salt sensitivity of blood pressure in non-diabetic people in China: a dietary intervention study. *Lancet*. 2009;373(9666):829-835. doi:10.1016/S0140-6736(09)60144-6.
77. Li C, He J, Chen J, et al. Genome-Wide Gene-Sodium Interaction Analyses on Blood Pressure: The Genetic Epidemiology Network of Salt-Sensitivity Study. *Hypertension*. 2016;68(2):348-355. doi:10.1161/HYPERTENSIONAHA.115.06765.
78. He J, Streiffer RH, Muntner P, Krousel-Wood MA, Whelton PK. Effect of dietary fiber intake on blood pressure: a randomized, double-blind, placebo-controlled trial. *J Hypertens*. 2004;22(1):73-80. doi:10.1097/01.hjh.0000098164.70956.54.
79. He J, Ogden LG, Vupputuri S, Bazzano LA, Loria C, Whelton PK. Dietary sodium intake and subsequent risk of cardiovascular disease in overweight adults. *JAMA*. 1999;282(21):2027-2034. doi:10.1001/jama.282.21.2027.
80. He J, Wofford MR, Reynolds K, et al. Effect of dietary protein supplementation on blood pressure a randomized, controlled trial. *Circulation*. 2011;124(5):589-595. doi:10.1161/CIRCULATIONAHA.110.009159.
81. Thompson AM, Zhang Y, Tong W, et al. Association of inflammation and endothelial dysfunction with metabolic syndrome, prediabetes and diabetes in adults from Inner Mongolia, China. *BMC Endocr Disord*. 2011;11(1):16. doi:10.1186/1472-6823-11-16.
82. Irazola V, Rubinstein A, Bazzano L, et al. Prevalence, awareness, treatment and control of diabetes and impaired fasting glucose in the Southern Cone of Latin America. *PLoS One*. 2017;12(9). doi:10.1371/journal.pone.0183953.
83. Bazzano LA, Li TY, Joshipura KJ, Hu FB. Intake of fruit, vegetables, and fruit juices and risk of diabetes in women. *Diabetes Care*. 2008;31(7):1311-1317. doi:10.2337/dc08-0080.
84. Bazzano LA, Serdula M, Liu S. Prevention of Type 2 Diabetes by Diet and Lifestyle Modification. *J Am Coll Nutr*. 2005;24(5):310-319. doi:10.1080/07315724.2005.10719479.
85. Pollock BD, Chen W, Harville EW, et al. Differential sex effects of systolic blood pressure and low-density lipoprotein cholesterol on type 2 diabetes: Life course data from the Bogalusa Heart Study. *Journal of Diabetes*. 2017.
86. Zhang T, Li Y, Zhang H, et al. Insulin-sensitive adiposity is associated with a relatively lower risk of diabetes than insulin-resistant adiposity: the Bogalusa Heart Study. *Endocrine*. 2016;54(1):93-100. doi:10.1007/s12020-016-0948-z.
87. Pollock BD, Hu T, Chen W, et al. Utility of existing diabetes risk prediction tools for young black and white adults: Evidence from the Bogalusa Heart Study. *J Diabetes Complications*. 2016;31(1):86-93. doi:10.1016/j.jdiacomp.2016.07.025.
88. Hu T, Yao L, Reynolds K, et al. The effects of a low-carbohydrate diet vs. a low-fat diet on novel cardiovascular risk factors: A randomized controlled trial. *Nutrients*. 2015;7(9):7978-7994. doi:10.3390/nu7095377.
89. Hu T, Yao L, Reynolds K, et al. The effects of a low-carbohydrate diet on appetite: A randomized controlled trial. *Nutr Metab Cardiovasc Dis*. 2016;26(6):476-488. doi:10.1016/j.numecd.2015.11.011.

90. Rebholz CM, Reynolds K, Wofford MR, et al. Effect of soybean protein on novel cardiovascular disease risk factors: A randomized controlled trial. *Eur J Clin Nutr*. 2013;67(1):58-63. doi:10.1038/ejcn.2012.186.

91. Wofford MR, Rebholz CM, Reynolds K, et al. Effect of soy and milk protein supplementation on serum lipid levels: A randomized controlled trial. *Eur J Clin Nutr*. 2012;66(4):419-425. doi:10.1038/ejcn.2011.168.

92. Chen J, He J, Wildman RP, Reynolds K, Streiffer RH, Whelton PK. A randomized controlled trial of dietary fiber intake on serum lipids. *Eur J Clin Nutr*. 2006;60(1):62-68. doi:10.1038/sj.ejcn.1602268.

93. Overweight and Obesity Expert Panel. *Managing Overweight and Obesity in Adults: Systematic Evidence Review From the Obesity Expert Panel, 2013*.; 2013.

94. The Data Center. *Who Lives in New Orleans and Metro Parishes Now?*; 2017. https://www.datacenterresearch.org/data-resources/who-lives-in-new-orleans-now/.

95. Wetzels JFM, Kiemeney LALM, Swinkels DW, Willems HL, Heijer M Den. Age- and gender-specific reference values of estimated GFR in Caucasians: The Nijmegen Biomedical Study. *Kidney Int*. 2007;72(5):632-637. doi:10.1038/sj.ki.5002374.

96. Radin MS. Pitfalls in hemoglobin A1c measurement: When results may be misleading. *J Gen Intern Med*. 2014;29(2):388-394. doi:10.1007/s11606-013-2595-x.

97. Kim PS, Woods C, Georgoff P, et al. A1C underestimates glycemia in HIV infection. *Diabetes Care*. 2009;32(9):1591-1593. doi:10.2337/dc09-0177.

98. Qaseem A, Wilt TJ, Kansagara D, Horwitch C, Barry MJ, Forciea MA. Hemoglobin A1c Targets for Glycemic Control With Pharmacologic Therapy for Nonpregnant Adults With Type 2 Diabetes Mellitus: A Guidance Statement Update From the American College of Physicians. *Ann Intern Med*. 2018;168(8):569-576. doi:10.7326/M17-0939.

99. Sumithran P, Prendergast LA, Delbridge E, et al. Ketosis and appetite-mediating nutrients and hormones after weight loss. *Eur J Clin Nutr*. 2013;67(7):759-764. doi:10.1038/ejcn.2013.90.

100. Gardner CD, Trepanowski JF, Gobbo LCD, et al. Effect of low-fat VS low-carbohydrate diet on 12-month weight loss in overweight adults and the association with genotype pattern or insulin secretion the DIETFITS randomized clinical trial. *JAMA - J Am Med Assoc*. 2018;319(7):667-679. doi:10.1001/jama.2018.0245.

101. Brenner BM, Meyer TW, Hostetter TH. Dietary protein intake and the progressive nature of kidney disease: the role of hemodynamically mediated glomerular injury in the pathogenesis of progressive glomerular sclerosis in aging, renal ablation, and intrinsic renal disease. *N Engl J Med*. 1982;307(11):652-659. doi:10.1056/NEJM198209093071104.

102. Sato J, Kanazawa A, Makita S, et al. A randomized controlled trial of 130 g/day low-carbohydrate diet in type 2 diabetes with poor glycemic control. *Clin Nutr*. 2017;36(4):992-1000. doi:10.1016/j.clnu.2016.07.003.

103. Yancy WS, Foy M, Chalecki AM, et al. A low-carbohydrate, ketogenic diet to treat type 2 diabetes. *Nutr Metab (Lond)*. 2005;2(1):34. doi:10.1186/1743-7075-2-34.

104. Boden G, Sargrad K, Homko C, Mozzoli M, Stein TP. Effect of a low-carbohydrate diet on appetite, blood glucose levels, and insulin resistance in obese patients with type 2 diabetes. *Ann Intern Med*. 2005;142(6). doi:10.1016/S0084-3741(08)70336-6.

105. Stern L, Iqbal N, Seshadri P. The Effects of Low-Carbohydrate versus Conventional Weight Loss Diets in Severely Obese Adults : One-Year Follow-up of a randomized control trial. *Ann Intern …*. 2004;140(May 2001):769-777.

doi:10.1016/j.accreview.2004.07.103.
106. Westman EC, Yancy WS, Edman JS, Tomlin KF, Perkins CE. Effect of 6-month adherence to a very low carbohydrate diet program. *Am J Med*. 2002;113(1):30-36. doi:10.1016/S0002-9343(02)01129-4.
107. Moosheer SM, Waldschütz W, Itariu BK, Brath H, Stulnig TM. A protein-enriched low glycemic index diet with omega-3 polyunsaturated fatty acid supplementation exerts beneficial effects on metabolic control in type 2 diabetes. *Prim Care Diabetes*. 2014;8(4):308-314. doi:10.1016/j.pcd.2014.02.004.
108. Aagaard K, Petrosino J, Keitel W, et al. The Human Microbiome Project strategy for comprehensive sampling of the human microbiome and why it matters. *FASEB J*. 2013;27(3):1012-1022. doi:10.1096/fj.12-220806.
109. McInnes P, Cutting M. Manual of Procedures for Human Microbiome Project. http://www.hmpdacc.org/doc/HMP_MOP_Version12_0_072910.pdf.
110. Marrero DG, Palmer KNB, Phillips EO, Miller-Kovach K, Foster GD, Saha CK. Comparison of commercial and self-initiated weight loss programs in people with prediabetes: A randomized control trial. *Am J Public Health*. 2016;106(5):949-956. doi:10.2105/AJPH.2015.303035.
111. Barengolts E, Manickam B, Eisenberg Y, Akbar A, Kukreja S, Ciubotaru I. EFFECT OF HIGH-DOSE VITAMIN D REPLETION ON GLYCEMIC CONTROL IN AFRICAN-AMERICAN MALES WITH PREDIABETES AND HYPOVITAMINOSIS D. *Endocr Pract*. 2015. doi:10.4158/EP14548.OR.
112. Block G, Azar KMJ, Romanelli RJ, et al. Diabetes prevention and weight loss with a fully automated behavioral intervention by email, web, and mobile phone: A randomized controlled trial among persons with prediabetes. *J Med Internet Res*. 2015. doi:10.2196/jmir.4897.
113. Kramer MK, Molenaar DM, Arena VC, et al. Improving employee health: Evaluation of a worksite lifestyle change program to decrease risk factors for diabetes and cardiovascular disease. *J Occup Environ Med*. 2015. doi:10.1097/JOM.0000000000000350.
114. Parker AR, Byham-Gray L, Denmark R, Winkle PJ. The Effect of Medical Nutrition Therapy by a Registered Dietitian Nutritionist in Patients with Prediabetes Participating in a Randomized Controlled Clinical Research Trial. *J Acad Nutr Diet*. 2014;114(11):1739-1748. doi:10.1016/j.jand.2014.07.020.
115. Maruthur NM, Ma Y, Delahanty LM, et al. Early response to preventive strategies in the diabetes prevention program. *J Gen Intern Med*. 2013;28(12):1629-1636. doi:10.1007/s11606-013-2548-4.
116. Leong A, Daya N, Porneala B, et al. Prediction of Type 2 Diabetes by Hemoglobin A1c in Two Community-Based Cohorts. *Diabetes Care*. 2017;41(1):dc170607. doi:10.2337/dc17-0607.
117. Khaw K-T, Wareham N, Bingham S, Luben R, Welch A, Day N. Association of hemoglobin A1c with cardiovascular disease and mortality in adults: the European prospective investigation into cancer in Norfolk. *Ann Intern Med*. 2004;141(6):413-420. doi:10.7326/0003-4819-141-6-200409210-00006.
118. Cole RE, Boyer KM, Spanbauer SM, Sprague D, Bingham M. Effectiveness of Prediabetes Nutrition Shared Medical Appointments. *Diabetes Educ*. 2013;39(3):344-353. doi:10.1177/0145721713484812.

Low-Carbohydrate Dietary Pattern on Glycemic Outcomes Trial

PROTOCOL

PROTOCOL VERSION 1.5

Dated: May 5, 2019

New Orleans, LA

Table of Contents

1. Overview 5
2. Background and Significance 6
2.A. Type 2 Diabetes Mellitus is a major global public health challenge 6
2.B. Prediabetes is a major risk factor for cardiometabolic diseases 6
2.C. Role of lifestyle in preventing diabetes 6
2.D. Low-to-moderate carbohydrate diets, weight loss, and cardiometabolic diseases 7
2.E. Mediterranean-style diet is associated with reduced risk of T2D and improved glycemic control 8
2.F. Evidence for low-carbohydrate diets that emphasize Mediterranean-style dietary components 8
2.G. Evidence for specific food groups and risk of T2D 8
2.H. Gut microbiota, diet and T2D 9
2.I. Clinical and public health significance of the proposed study 9
2.J. Innovation 9
3. Preliminary Studies 10
3.A. Summary of aggregate experience of the research team 10
3.B. Low-carbohydrate behavioral dietary intervention trial experience 10
3.C. Other dietary intervention trial experience 11
4. Study Design and Organization 11
4.A. Overview of trial design 11
4.B. Study outcomes 12
4.B.1. Primary outcome 12
4.B.2. Secondary outcomes 12
4.C Study Timeline 12
6. Study Participants 13
6.A. Eligibility criteria 13
7. Recruitment and Randomization 13
7.A. Recruitment 13
7.A.1. Mass mailing, placement of brochures/flyers in community, TV/radio/billboard advertisements 14
7.A.2. Database searches and targeted mailing 14
7.A.3. Referrals and Local Partnerships 14
7.B. Randomization 14
7.C. Masking 15

7.D. Safety Considerations 15
8. Dietary Intervention Program 15
8.A. Low-carbohydrate dietary intervention group 15
8.B. Usual Diet Group 16
8.C. Physical Activity 16
8.D. Dietary Adherence 17
9. Safety of Dietary Intervention 17
10. Data Collection 18
10.A. Study visits 18
10.A.1. Screening/baseline visits 18
10.A.2. Follow-up visits and final visit 19
10.B Study measurements 19
10.B.1. Questionnaires 19
10.B.2. Dietary measurements 20
10.B.3. Assessment and management of adverse effects 20
10.B.4. BP and anthropometric measures 20
10.B.5. Blood samples 20
10.B.6. Urine collection 21
10.B.7. Stool Specimen Collection 21
11. Quality Assurance and Quality Control 21
11.A. Manual of procedures 21
11.B. Training and certification 21
12. Sample Size and Statistical Power 22
13. Data Management and Analysis 23
13.A. Data management 23
13.B. Data analysis plan 23
13.B.1. Exploratory data analysis 23
13.B.2. Primary, secondary, and exploratory outcomes 23
15. Anticipated Challenges 24
16. Protection of Human Subjects 24
16.A. Protection of human subjects from research risks 24
16.A.1. Risks to the subjects 24
16.A.2. Adequacy of protection against risks 26

16.A.3. Potential benefits of the proposed research to human subjects and others... 27
16.A.4. Importance of knowledge to be gained ... 27
16.A.5. Remuneration ... 27
16.B. Data safety and monitoring plan ... 27
Bibliography and References Cited ... 29

1. Overview

The increasing prevalence of type 2 diabetes mellitus (T2D), a major cause of morbidity and mortality worldwide, is a critical public health concern. In the United States, an estimated 33.9% of adults have prediabetes (impaired fasting glucose, elevated hemoglobin A1c (HbA1c), or impaired glucose tolerance). Individuals with prediabetes are at elevated risk of T2D and cardiovascular disease (CVD), with 5–10% developing clinical T2D each year.

In the short-term, among patients with T2D, low-to-moderate carbohydrate diets (energy percentage below 45) have a greater glucose-lowering effect than do high-carbohydrate diets, with larger glucose-lowering effect the greater the carbohydrate reduction. Additionally, compared with low-fat diets, Mediterranean diets that are high in unsaturated fats (particularly *cis*-monounsaturated fats) reduce risk of T2D and CVD and improve glycemic control among diabetics. However, compared with usual diets, the effect of a behavioral intervention promoting a low-carbohydrate/high-unsaturated fat and protein dietary pattern that emphasizes consumption of non-starchy vegetables, plant-based oils, fish, poultry, and mixed nuts on glucose homeostasis, and other metabolic risk factors among individuals with prediabetes or early-stage untreated T2D is not well understood.

Our *long-term goal* is to develop and implement dietary intervention(s) that reduce risk of T2D. The *overall goal of this randomized controlled trial is to study the effect of a behavioral intervention promoting a low-carbohydrate/high-unsaturated fat and high-protein dietary pattern (initial target <40 g digestible carbohydrates, final target <60 g digestible carbohydrates) compared with a usual diet on metabolic risk factors among individuals with prediabetes or early-stage untreated diabetes* (HbA1c 6.0–6.9%). Specifically, we will test the following hypotheses:

Primary hypothesis: Compared with usual diet, over a 6-month period, a healthy low-carbohydrate/high unsaturated fat and high-protein dietary pattern will lead to *a larger decrease of HbA1c, by at least 0.15%.*

Secondary hypothesis: Over a 6-month period, a healthy low-carbohydrate/high-unsaturated fat and high-protein dietary pattern will lead to *larger improvements in the following major secondary outcomes*: fasting glucose, systolic blood pressure (BP), total-to-HDL-cholesterol ratio, and body weight.

Exploratory outcomes: We will also study the effect of the intervention on the following outcomes: insulin and homeostasis model assessment of insulin resistance (HOMA-IR); diastolic BP; waist circumference; and estimated CVD risk.

To achieve these study aims, we will recruit 112 adults with prediabetes or untreated diabetes (HbA1c 6.0–6.9%) in the Greater New Orleans, LA area. Using a stratified, blocked randomization scheme, participants will be assigned to either a behavioral modification intervention aimed at promoting a healthy low-carbohydrate dietary pattern or to usual diet. The intervention group will have an intensive phase for 3 months, followed by a maintenance phase in months 4 through 6. The *primary outcome will be difference between the active intervention and control groups in the change in HbA1c from baseline to 6 months of follow-up. We expect 85% power to detect a difference of at least 0.15% in change of HbA1c.* Changes in other metabolic risk factors will be studied concurrently. Metabolic risk factors will be assessed at baseline, 3 months, and 6 months of follow-up.

There is much interest regarding dietary approaches to prevent T2D and its complications. The results from this study will help to determine whether, compared with usual diet, a behavioral intervention aimed at promoting a healthy low-carbohydrate dietary pattern improves metabolic risk factors.

2. Background and Significance

2.A. Type 2 Diabetes Mellitus is a major global public health challenge

Diabetes is a strong and independent risk factor for CVD morbidity and mortality. A meta-analysis of 102 prospective studies (698,782 participants) estimated diabetes leads to an approximately 2-fold increased risk in vascular diseases, including coronary heart disease (CHD) and major stroke subtypes, and deaths due to other vascular causes.[1] Among participants with diabetes, CHD risk is about 50% higher among those with fasting blood glucose ≥7 mmol/L compared with those with fasting blood glucose <7 mmol/L.[1] In addition to increasing CVD risk, diabetes can cause diabetic retinopathy,[2] chronic kidney disease,[3] lower limb amputation,[4] and other complications. Most individuals with diabetes have T2D.[5] The US Centers for Disease Control and Prevention (CDC) estimated that 30.2 million adults ≥18 years (12.2%) had diabetes in 2015, with 7.2 million (23.8%) not being aware of or reporting diabetes.[5] Diagnosed diabetes prevalence among US adults has increased substantially over time, from 2.54% in 1980 to 7.40% in 2015.[6] Diabetes prevalence is also high globally, with an estimated 2014 age-standardized prevalence of 9.0% in men and 7.9% in women.[7] Diabetes accounts for an increasing portion of US healthcare expenditures. Total cost of diagnosed diabetes in the US was estimated to be $245 billion in 2012, 41% higher than in 2007.[8] Diabetes is associated with much higher lifetime medical expenses, with higher expenses for those diagnosed at younger ages.[9]

2.B. Prediabetes is a major risk factor for cardiometabolic diseases

Prediabetes, defined by the American Diabetes Association (ADA) as impaired fasting glucose (IFG), impaired glucose tolerance (IGT), or elevated HbA1c, is also referred to as “intermediate hyperglycemia”[10] and “high-risk state of developing diabetes”.[11] The ADA defines prediabetes as having: fasting plasma glucose of 100–125 mg/dL (IFG); 2-hour post-load glucose in the 75-g oral glucose tolerance test (OGTT) of 140–199 mg/dL (IGT); or HbA1c 5.7–6.4%.[12] Prediabetes itself is not a disease; for all three tests, “risk is continuous, extending below the lower limit of the range and becoming disproportionately greater at the higher end of the range”.[12] The CDC estimated that in 2015, 33.9% of US adults had prediabetes based on fasting glucose or HbA1c.[5] Among those with prediabetes, only 11.9% reported having being told this by a medical professional.[5] Approximately 5–10% of individuals with prediabetes develop diabetes within one year, depending on how prediabetes is defined.[13] In a meta-analysis, annual diabetes incidence was 15–19% among individuals with both IFG and IGT, while it was 4–6% in those with isolated IFG and 6–9% in those with isolated IGT.[13]

A recent meta-analysis (1,611,339 participants from 53 studies) reported that prediabetes as defined by the ADA is associated with higher risk of composite CVD events and CHD.[14] IFG and IGT were also positively associated with increased risk of stroke and all-cause mortality.[14] Prediabetes is linked with increased risk of other conditions typically thought of as diabetes complications, such as neuropathies and nephropathies.[15] Importantly, it is possible for individuals with prediabetes to revert to normoglycemia, as observed in observational studies[13] and lifestyle or drug intervention trials.[16,17] In the Diabetes Prevention Program (DPP), reversion to normoglycemia was associated with lower long-term T2D risk[18] and lower predicted CVD risk.[19]

2.C. Role of lifestyle in preventing diabetes

Much evidence from observational studies and clinical trials supports the critical role of diet and other lifestyle factors in preventing T2D. Globally, lifestyle transitions towards reduced physical activity, increased consumption of refined carbohydrates, coupled with resulting changes in body composition, has led to increases in diabetes prevalence and severity.[20] Body

mass index (BMI), waist circumference, and waist-to-height ratio are predictors of T2D.[21] The DPP, which combined calorie restriction and exercise to promote weight loss, substantially reduced risk of T2D (by 58%) among individuals with elevated FPG and IGT;[22] a benefit that persisted in 10-year follow-up analyses.[16] Other trials of lifestyle modification focused on diet and exercise have also shown benefits in study populations in China,[23] Finland,[24] and India.[25]

2.D. Low-to-moderate carbohydrate diets, weight loss, and cardiometabolic diseases

Low-to-moderate carbohydrate diets (<45% of energy from carbohydrates) are popular dietary approaches for losing weight. Weight loss is a key component of the DPP lifestyle intervention[26] and ADA lifestyle recommendations.[12,27] Meta-analyses from our group[28] and others support that low-to-moderate carbohydrate diets are at least as effective as low-fat diets at promoting weight loss. For instance, a meta-analysis reported a 2-kg (95% CI, 3.1–0.9) larger weight loss in low-carbohydrate diet (<120 g carbohydrates/day) arms compared with low-fat diet (<30% energy from fat) arms.[29] Estimated 10-year CVD risk was lower in low-carbohydrate than low-fat arms.[29] This aligns with research published by our team; in a 12-month trial of participants with obesity, a low-carbohydrate diet (target <40 g carbohydrates/day) was more effective than a low-fat diet (target <30% energy from fat, <7% saturated fat) for weight loss and CVD risk factor reduction.[30]

Low-to-moderate carbohydrate diets may also be beneficial among patients with T2D. Low-carbohydrate diets have come in and out of favor as treatment of diabetes.[31] In a meta-analysis (1376 patients;10 trials), after 3 or 6 months of the intervention, low-to-moderate carbohydrate diets (<45% energy from carbohydrates) led to a 0.34% lower HbA1c (95% CI, 0.06–0.63%) compared with high-carbohydrate diets.[32] Compared with high-carbohydrate diets, there was a greater reduction in HbA1c the greater the reported carbohydrate restriction.[32] There was no benefit of the low-to-moderate carbohydrate diets on HbA1c at 12 months or longer, and no difference between interventions on BMI, body weight, low-density lipoprotein (LDL) cholesterol or quality of life.[32] However, results may have been affected by dietary adherence or glucose medication. There was evidence of diabetes medication reduction in the low-carbohydrate arms in some studies,[33–37] with evidence of medication reduction and improved blood glucose stability persisting for up to 2 years.[38] Hypoglycemia (though not after medication adjustment) was observed in three subjects taking insulin or a sulfonylurea out of twelve subjects in the low-carbohydrate arm of another study.[39] In a subset of participants with T2D (n=46) from a large weight loss trial, compared with those randomized to a low-fat diet and orlistat, those randomized to a low-carbohydrate diet for 48 weeks had improved HbA1c (0.8% lower, 95% CI, -1.5, -0.02%) and a reduction in antiglycemic medications, despite similar weight loss effects of the interventions.[40]

Though low-to-moderate carbohydrate diets have been studied among overweight and obese participants (primarily focused on weight loss), less is understood about the effects of such diets on glycemic outcomes specifically among individuals with prediabetes. A study among participants with IGT observed a reduction at 12 months in weight, HbA1c, and other glycemic outcomes among individuals who participated in a 7-day in-hospital education program, though this was not randomized.[41] A randomized pilot trial of overweight or obese individuals with T2D or prediabetes found that, over 3 months and at 12 months, a very low-carbohydrate diet improved HbA1c, led to higher medication discontinuation, and more weight loss compared with a low-fat diet.[34,37] However, only 4 individuals had prediabetes. Given benefits of low-to-moderate carbohydrate diets for weight loss in general populations and for glycemic control among patients with T2D, further study of these diets are warranted among individuals with prediabetes who are at elevated cardiometabolic disease risk.

2.E. Mediterranean-style diet is associated with reduced risk of T2D and improved glycemic control

Though there is no one “Mediterranean diet”, the Mediterranean dietary pattern tends to have the following characteristics: high consumption of vegetables, fruits, breads and cereals, legumes, nuts and seeds; low-to-moderate consumption of dairy products, fish, poultry and eggs; little consumption of red meat and sweets; and red wine in moderation.[42] Olive oil is an important source of fat. In many observational studies, adherence to the Mediterranean-style dietary pattern is associated with reduced risk of CVD[43] and T2D.[44,45] Evidence from randomized trials also supports the benefits of this dietary pattern. In several trials, participants randomized to Mediterranean diets had substantially lower risk of CVD than those randomized to low-fat control diets.[46,47] In the three-arm randomized PREDIMED trial of participants at high risk of CVD, after 4.1 years of follow-up, those randomized to a traditional Mediterranean diet supplemented with either olive oil or nuts had a 30% reduced risk of T2D compared to those in the low-fat control diet group.[48] In all PREDIMED arms, average carbohydrate intake was relatively low (39.7–43.7% at end of trial).[47] Two trials have found a higher rate of remission from metabolic syndrome among individuals randomized to a Mediterranean diet compared with those randomized to control diets (prudent or low-fat) during 2–5 years of follow-up.[49,50] The Mediterranean pattern may also be beneficial among individuals with T2D. Meta-analyses have reported that, among patients with T2D, those randomized to a Mediterranean diet had lower HbA1c levels and CVD risk factors, and more significant weight loss, compared with those randomized to control diets.[51]

2.F. Evidence for low-carbohydrate diets that emphasize Mediterranean-style dietary components

Many low-carbohydrate diets involve increased consumption of food items, such as red meat,[52] that are associated with increased cardiometabolic disease risk. However, low-carbohydrate dietary approaches can focus on substituting carbohydrates with foods common in Mediterranean-style diets, such as olive oil and nuts, which may improve cardiometabolic health. In a 4-year Italian study of individuals with newly diagnosed T2D, those randomized to a low-carbohydrate Mediterranean diet (<50% carbohydrates; consumed ~44%) had more favorable changes in glycemic control, higher rates of T2D remission, and delayed need for antiglycemic drugs compared with participants randomized to a low-fat diet (<30% fat, <10% saturated fat).[53,54] A 12-month Israeli study assessed low-carbohydrate Mediterranean (35% carbohydrates), traditional Mediterranean, and ADA (both 50–55% carbohydrates) diets in 259 overweight participants with T2D.[55] Most CVD risk factors improved in all groups; there was only HDL improvement in the low-carbohydrate Mediterranean diet and this diet was superior to the other diets in improving glycemic control.[55] In a 2-year weight-loss study of moderately obese participants, low-carbohydrate and Mediterranean diets were superior to low-fat diet for weight loss.[56] Participants in the low-carbohydrate arm were counseled to choose vegetarian sources of fat and protein and to avoid trans fat. At 6 years after study commencement, despite weight regain, there were still long-term benefits, particularly for the low-carbohydrate and Mediterranean diets.[57] In whole, evidence supports that low-carbohydrate Mediterranean-style diets are feasible and may have metabolic benefits.

2.G. Evidence for specific food groups and risk of T2D

Prospective cohort studies have found consumption of specific foods and beverages is associated with diabetes risk. Meta-analyses report higher consumption of processed and unprocessed red meat,[58] white rice (particularly in Asian populations),[59] and sugar-sweetened beverages[60,61] are associated with higher T2D risk. Consumption of green leafy vegetables,[62,63] yogurt,[64] whole grains,[65] decaffeinated and total coffee,[66] and light-to-moderate alcohol

consumption[67] are associated with reduced risk. Consumption of fruits, particularly blueberries, grapes, and apples, are associated with reduced risk; fruit juice is associated with higher risk.[68]

2.H. Gut microbiota, diet and T2D

There is increasing evidence for the importance of gut microbiota in risk of type 2 diabetes and other cardiometabolic diseases.[69] Additionally, dietary modifications, including changes in carbohydrate consumption, can lead to substantial changes in gut microbiome over short periods of time.[70,71] Less is known about longer-term changes to the gut microbiota during the adoption of a low-carbohydrate diet and how these changes may influence cardiometabolic risk. We plan to collect stool specimens at baseline, 3 months, and at 6 months, for potential future studies on how this intervention affects the gut microbiome.

2.I. Clinical and public health significance of the proposed study

Prediabetes, or intermediate hyperglycemia, is a marker of high risk of developing T2D and other complications. Due to increasing prevalence of T2D and associated morbidity and mortality globally, a better understanding of dietary approaches to delay progression to T2D or to promote reversion to normoglycemia is a high public health priority. This study proposes to assess the effect of a behavioral intervention promoting a low-carbohydrate/high-unsaturated fat dietary pattern (initial target <40 g digestible carbohydrates, final target <60 g digestible carbohydrates) on HbA1c, a marker of glycemic control, and other metabolic risk factors. Building from prior findings from clinical and observational studies, we will develop a dietary intervention that promotes the consumption of non-starchy vegetables, plant-based oils, fish, poultry, and mixed nuts, that are thought to reduced risk of T2D or CVD. Participants assigned to the intervention will be trained in behavioral techniques that will provide capacity to maintain dietary changes long after study completion. Our outcome measures will assess the effect of this dietary intervention in a "real-life" setting as opposed to dietary modifications achieved in metabolic ward studies or through food prepared in research kitchens. Findings from this study may provide evidence in support of promoting alternative dietary styles among individuals with high T2D and CVD risk and could potentially be used to inform dietary interventions used in programs like DPP.

2.J. Innovation

The status quo as it pertains to dietary approaches for reducing risk of T2D is focused on reducing caloric intake and total fat intake. However, recent evidence suggests that the types of fat, rather than the quantity of fats may be more important, with evidence supporting that a Mediterranean-style diet that does not restrict fat intake reduces risk of developing both T2D[48] and CVD[47]. Moreover, in the short-term, low-carbohydrate diets, which are popular weight loss diets, have a greater glucose-lowering effect than do high-carbohydrate diets among patients with T2D.[32] However, the effect of low-carbohydrate diets that emphasize consumption of non-starchy vegetables, plant-based oils, fish, poultry, and mixed nuts on T2D prevention is unclear, particularly compared with higher-carbohydrate diets more commonly consumed in the US. *The proposed research is innovative, in our opinion, because it represents a substantial departure from the status quo by assessing the combined effect of a low-carbohydrate, high unsaturated fat and protein diet on cardiometabolic risk factors among adults with prediabetes or early-stage untreated diabetes.* We expect the results from this study have the potential to lead to new horizons for developing and implementing dietary approaches (other than the most commonly used reduced fat diet) that will substantially reduce risk of cardiometabolic disease among adults with T2D or at high risk of T2D. The proposed intervention dietary approach promotes consumption of dietary components thought to reduce risk of cardiometabolic disease and the behavior change intervention has expected applicability in clinical practice.

3. Preliminary Studies

3.A. Summary of aggregate experience of the research team

Dr. Kirsten Dorans is trained in cardiovascular disease epidemiology, with strong training in epidemiologic methods and biostatistics. Her publication record includes studies of behavioral risk factors and risk of chronic disease. She has published on the relationships between dietary intake and chronic heart failure[72,73] and on physical activity and incident multiple sclerosis[74]. She served as lead author, conducting data management, analysis, drafting, and revision of two of these manuscripts. In graduate school, she focused on studying links between outdoor air pollution and chronic diseases and has published several papers in this field. The COBRE program will provide an excellent opportunity for the JFI to transition from air pollution research to clinical and translational research focused on nutrition and diabetes. Building off of her training in observational epidemiology, this program will involve training and practical experience that will allow her to broaden her expertise and to develop practical skills for carrying out clinical research.

The mentorship team has much experience and prior successful collaborations in key areas related to this study. Dr. Jiang He is an internationally renowned expert in cardiometabolic disease clinical and epidemiological research. He has served as PI for many epidemiological studies and clinical trials, including clinical and observational studies focused on dietary consumption and chronic diseases[75–80] and has conducted extensive global research on diabetes.[81,82] Dr. Bazzano has collaborated extensively with Dr. He for more than 15 years in research related to diet and chronic disease. She is a clinical investigator and has expertise in nutritional epidemiology and clinical research. Dr. Bazzano was the PI of a highly successful clinical trial comparing the effects of low-carbohydrate and low-fat diets on weight and cardiovascular risk factors among individuals with obesity, which was a subproject within the Tulane COBRE in Hypertension and Renal Biology.[30] Dr. He served as Dr. Bazzano's mentor for this clinical trial. They have collaborated on much other work related to dietary and chronic disease, including a meta-analysis on low-carbohydrate diets.[28] Dr. Bazzano has also published many articles on diet and risk of diabetes[83,84] and on diabetes in the Bogalusa Heart Study.[85–87]

3.B. Low-carbohydrate behavioral dietary intervention trial experience

In the study on macronutrient composition of diet and risk factors for cardiovascular disease (MACRO) study, 148 individuals without clinical cardiovascular disease or diabetes and with obesity were randomly assigned to a low-carbohydrate or a low-fat behavioral dietary intervention for 12 months. In the low-carbohydrate arm, daily dietary intake of carbohydrates decreased from an average of 242 g at baseline to 97 g, 93 g, and 127 g at 3, 6, and 12 months, respectively. At 12 months, participants in the low-carbohydrate intervention group had greater decreases in weight (-3.5 kg; 95% CI, -5.6 to -1.4 kg) and fat mass (-1.5%; 95% CI, -2.6% to -0.4%) and greater improvement in several cardiovascular risk factors.[30] This suggests that restricting carbohydrates may be an option for weight loss and reduction of cardiovascular risk factors. Further analyses found that the low-carbohydrate dietary intervention led to similar or greater improvement in inflammation, adipocyte dysfunction, and endothelial dysfunction compared with the low-fat dietary intervention[88] and that the low-fat diet reduced peptide YY more than the low-carbohydrate diet, suggesting satiety may be better preserved on low-carbohydrate diets.[89] No serious adverse events were reported in this study and the number of participants with symptoms were similar between the two diets, with the exception of more participants having headaches on the low-fat diet at 3 months (18 vs 6 participants, $p = 0.03$).[30] Dr. Bazzano served as PI for this study and Dr. He served as her mentor. Dr. Bazzano

mentored one of her PhD students in conducting analyses and writing and editing manuscripts related to this project.

3.C. Other dietary intervention trial experience

Dr. He has also led or been involved with many dietary intervention trials, including both behavioral change and feeding study interventions. In the Protein and Blood Pressure (ProBP) study, 352 adults with prehypertension or stage 1 hypertension in New Orleans, LA and Jackson, MS were assigned to take, in a random order, 40 g/d soy protein, milk protein, or carbohydrate supplementation each for 8 weeks. Compared with the high-glycemic index refined carbohydrate, both soy protein and milk protein supplementation were associated with a -2.0 mm Hg (95% CI -3.2 to -0.7 mm Hg) and -2.3 mm Hg (-3.7 to -1.0 mm Hg) net change in systolic blood pressure.[80] These findings suggest that partial replacement of carbohydrate with soy or milk protein might be an important part of nutrition intervention strategies to prevent and treat hypertension. Additionally, soy protein supplementation led to reduction in plasma E-selectin compared with carbohydrate and milk protein and in plasma leptin compared with carbohydrate.[90] Soy protein also led to improvement in lipid profile compared with carbohydrate and milk protein.[91] Dr. He was PI for this project.

In the Fiber Intervention on Blood Pressure (FIBRE) study, 110 participants ages 30–65 years with untreated pre-hypertension or stage-1 hypertension and serum cholesterol level < 240 mg/dl were randomly assigned to 8-g/day of water soluble fiber from oat bran or a control intervention (double-blind RCT). Over the 12-week study period, net changes in systolic and diastolic blood pressure were -1.8 mm Hg (-4.3 to 0.8) and -1.2 mm Hg (-3.0 to 0.5 mm Hg), respectively.[78] Net changes in total, HDL-, and LDL-cholesterol were -2.40 mg/dL (-10.60 to 5.81 mg/d:), -1.66 mg/dL (-4.55 to 1.22 mg/dL), and -1.33 mg/dl (-8.33 to 5.68 mg/dL), respectively.[92] Dr He was PI for this study.

Dr. He is currently serving as a mentor for Dr. Katherine Mills' COBRE project (Effect of Dietary Sodium Reduction in Kidney Disease Patients with Albuminuria; SUPER). In this project, participants with chronic kidney disease and albuminuria are being randomized to a behavioral modification program designed to reduce dietary sodium intake to ≤2,300 mg/day or to usual care and test the effect of this intervention on change in albumin-to-creatinine ratio from baseline to 24-weeks.

4. Study Design and Organization

4.A. Overview of trial design

We propose to conduct a *randomized controlled trial* aimed at testing the effect of a behavioral intervention that promotes *reduced carbohydrate intake and increased consumption of heart-healthy unsaturated fats and protein* in patients with HbA1c 6.0–6.9% (*Figure 1*). We plan to recruit 112 individuals with elevated HbA1c and randomly assign them to a moderately intensive intervention on diet pattern,[93] using well-established methods for behavioral modification, or to usual diet. We will compare the difference between the active intervention and control groups for change in HbA1c from baseline to 6 months of follow-up (primary outcome). We will assess four major secondary outcomes: fasting plasma glucose, systolic BP, total-to-HDL cholesterol ratio, and body weight. In

Figure 1. Study Design

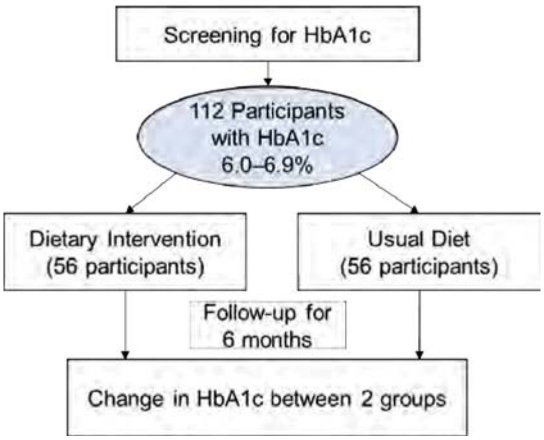

addition, we will look at several other exploratory outcomes: insulin and HOMA-IR, diastolic BP, waist circumference, and estimated CVD risk, as estimated by the 2013 ACC/AHA risk equation. The main trial components are summarized in *Table 1*.

Table 1. Study Overview

Study Design	Randomized controlled trial
Study Participants	112 participants with elevated HbA1c (6.0–6.9%)
Intervention Group (n=56)	Behavioral modification to reduce carbohydrate consumption and promote consumption of foods such as non-starchy vegetables, plant-based unsaturated oils, nuts and seeds, avocados, eggs, seafood, poultry, lean meat and tofu. We will recommend moderate consumption of cheese, unsweetened Greek yogurt, and low-carb milk. If consuming higher-carbohydrate items, recommend legumes, fruits, (limited) whole grains. Target <40 g net carbohydrates per day for first 3 months and will have a goal of <40 net g carbohydrates per day; <60 g net carbohydrates per day for months 4 onwards.
Usual Diet Group (n=56)	No dietary intervention. To encourage continued participation in the trial, we will offer optional monthly educational sessions on unrelated topics, such as retirement planning or health insurance
Outcome Measures	Primary: HbA1c; Secondary: fasting plasma glucose, systolic BP, total-to-HDL-cholesterol ratio, and body weight

4.B. Study outcomes

4.B.1. Primary outcome: The ***primary outcome will be the difference between the intervention group and the usual diet group for change in HbA1c*** from baseline to 6 months. Changes in HbA1c will be calculated from blood samples taken at baseline and 6 months.
4.B.2. Secondary outcomes: Secondary outcomes will include changes from baseline to 6 months of *fasting plasma glucose, systolic BP, total-to-HDL cholesterol ratio, and body weight.,*
4.B.3. Other outcomes: We will also study the effect of the intervention on the following outcomes: insulin and homeostasis model assessment of insulin resistance (HOMA-IR); diastolic BP; waist circumference; total-to-HDL-cholesterol ratio; and estimated CVD risk.

4.C Study Timeline

Table 2 shows the timeline, which will be followed in the implementation of study-related activities, such as publications and submission of an R01 grant application.

Table 2. Study Timeline

	Year 1		Year 2		Year 3	
Develop MOP, intervention program, study forms, and pilot testing	X	X				
Certification and training of staff	X					
Recruitment		X	X			
Intervention and data collection, measurement, entry, cleaning		X	X	X	X	
Data quality control and analysis						X
Publications	X	X	X	X	X	X
R01 grant submissions, resubmissions				X	X	X

The first 6 months will focus on development of manual of operations (MOP), intervention program, study forms, study protocol, Institutional Review Board (IRB) approval, and pilot

testing of the intervention program. It will also involve staff training and certification. During the last half of year 1, recruitment, intervention, and data collection will commence. During the last 3 months of year 3, data quality control and analysis will be carried out. Throughout the study period, the JFI will publish peer-reviewed manuscripts from ongoing Tulane University research projects.

6. Study Participants

6.A. Eligibility criteria

Inclusion criteria

- Men or women ages 40 to 70 years: aim to recruit 50% women
- Any race or ethnicity: aim to recruit 40% ethnic minorities (i.e., African American and Hispanic), close to population in the greater New Orleans area[94]
- HbA1c 6.0–6.9%
- Willing and able to provide informed consent

Exclusion criteria

- Diagnosed type 1 diabetes mellitus based on patient self-report
- Use of agents affecting glycemic control (medications for diabetes, oral glucocorticoids) within the past three months prior to enrollment based on patient self-report
- Medical condition in which low-carbohydrate diet may not be advised
    - Estimated glomerular filtration rate (eGFR) ≤45 mL/min/1.73 m^2, which is close to the 5th percentile of eGFR among non-diseased individuals of 70 years of age[95]
    - Self-report of liver disease due to hepatitis or alcohol; osteoporosis; untreated thyroid disease; gout; or cancer (other than non-melanoma skin cancer) requiring treatment in the past year, unless prognosis is excellent
- Factors that may affect HbA1c:
    - Hemoglobin <11 mg/dL (cutpoint for moderate-to-severe anemia, which could lead to falsely elevated or lowered HbA1c)[96]
    - Recent blood donation or blood transfusion (self-report, past 4 months)
    - Human Immunodeficiency Virus (self-report)[97]
- Allergies to nuts
- For women, current pregnancy, breastfeeding, or plans to become pregnant during the study
- Consumption of ≥21 alcoholic drinks per week or consumption of ≥6 drinks per occasion
- Current or planned residence making it difficult to meet trial requirements (due to distance from study site and/or challenges regularly traveling to site)
- Current participation in another lifestyle intervention trial or a pharmaceutical trial
- Participation of another household member in the study; employees or persons living with employees of the study
- Other concerns regarding ability to meet trial requirements, at the discretion of the principal investigator or study coordinator

7. Recruitment and Randomization

7.A. Recruitment

We will recruit study participants from the greater New Orleans area. The initial focus of our recruitment will be mass mailing of individuals in the greater New Orleans area and recruitment through placing flyers and brochures in the community. If community-based recruitment is not successful or cost-effective, we will recruit out-patients from the Ochsner Health System and

Tulane Medical Center (TMC), from which our group has prior experience recruiting patients. For this recruitment, we will focus on electronic searches of medical records and laboratory databases. We will also carry out targeted solicitation of referrals from physicians who care for patients likely to meet our inclusion and exclusion criteria.

In addition to this recruitment plan, we have several contingency plans: (1) extending recruitment to other medical centers in the New Orleans area, including the Veterans Affairs Medical Center (VAMC), East Jefferson General Hospital, and West Jefferson Medical Center, with whom Tulane has well-established relationships; (2) TV/radio advertisements; (3) arrange a news story about the study in local TV newscasts, radio talk programs, and/or print media; and (4) conduct outreach at local community events and churches. We have ample experience with each of these approaches.

Based on U.S. Census Bureau 2016 population estimates, the metro New Orleans area population was approximately 35% African American or black and 9% Hispanic.[94] Given our prior experience and population distribution in this region, we plan to recruit a distribution of study participants with 40% ethnic minorities.

7.A.1. Mass mailing, placement of brochures/flyers in community, TV/radio/billboard advertisements: Community recruitment approaches include: mass mailing individuals who live in the New Orleans area; brochures/flyers in the community; emailing the brochure; TV, radio, poster, or billboard advertisements in the New Orleans area. Interested individuals will be pre-screened; those at elevated risk for prediabetes or T2D, i.e. individuals who are obese or overweight and/or have a sufficiently high score from the American Diabetes Association's Diabetes Risk Assessment Questionnaire will be invited into the clinic for a screening visit.

7.A.2. Database searches and targeted mailing: We will request IRB approval for a waiver for HIPAA authorization to conduct database searching and hospital chart reviews for identification of potentially eligible study participants. After identifying a potentially eligible patient, we will send this individual an invitation letter signed by the investigators and/or the individual's personal physician. Individuals who express interest in the study will have a pre-screening telephone interview for confirmation of potential eligibility. Those still interested and potentially eligible for enrollment will be scheduled for a visit at the COBRE study clinic.

7.A.3. Referrals and Local Partnerships: In addition to local physician referrals, we will explore holding outreach community events in the New Orleans area. Local community partners may post information about the study on their social media platforms. We may also identify potentially interested volunteers from a Tulane volunteer recruitment registry. We will also consider recruiting potentially eligible individuals who were screened or participated in other clinical studies in our clinic and have expressed interest in being contacted about future research projects. Potential participants referred by their physician or community partners will have a pre-screening telephone interview to confirm potential eligibility. Those who are still interested and potentially eligible for the study will be scheduled for a screening visit.

7.B. Randomization

Once eligibility has been confirmed, the study coordinator will ensure that all key variables have been entered into the trial-specific database. Additionally, the study coordinator will ensure any outliers have been recognized and reconciled, and will ensure receipt of informed consent prior to enrolling the volunteer in the study as a trial participant. Eligible participants will be *randomly assigned to the behavioral intervention or the usual diet group in a 1:1 allocation ratio*. Randomization will be stratified by sex, *using a random block group size (4, 6) to maintain balance among groups over time*. A statistician from the Methodology/Biostatistics Unit within the COBRE Clinical Research Core, who is not directly involved in the study and is not located at the clinic, will conduct the randomization. After ascertaining eligibility, clinical personnel will

telephone the Methodology/Biostatistics Unit to obtain a randomization assignment. All randomization assignments will be created by a computer program and only accessible to the Staff at the Methodology/Biostatistics Unit. Once randomization assignment has been made, trial participants will be considered to be officially randomized into the study and every effort will be made to obtain complete follow-up information.

7.C. Masking

Clinical and laboratory staff members who assess outcomes will be blinded to the intervention assignment of participants. As this is a dietary intervention trial, study participants, and study staff members who conduct the intervention cannot be blinded to the intervention assignment. Participants will be instructed to avoid sharing knowledge of their treatment assignment with data collection staff members. We will have intervention visits and data collection study visits on different days and in separate locations. Data collectors will also use verbal cues and visual props to remind participants not to disclose treatment assignment. Investigators will be blinded to intervention assignment.

7.D. Safety Considerations

Participants with HbA1c >7.9% will be referred to a clinician for follow-up[98], though we will continue to collect data on these participants.

8. Dietary Intervention Program

8.A. Low-carbohydrate dietary intervention group

Participants randomized to the low-carbohydrate intervention group will receive counseling aimed at reducing carbohydrate consumption. Phase I (*Go Low*) will occur for the first 3 months, with a goal of <40 g digestible carbohydrates per day. Key recommended foods will include: non-starchy vegetables, fish, poultry, lean meat, eggs, olive oil and other plant-based unsaturated oils, unsweetened/unsalted nuts and seeds, nut butters, and avocados. We will recommend moderate consumption of cheese and unsweetened Greek yogurt and low-carbohydrate milk. During Phase I, we will recommend limiting or avoiding other dairy, fruits, legumes, beans, and grains. Phase II (*Keep it Low*) will run from months 4 through 6. The net carbohydrate goal for this phase will be <40 g to <60 g, with the objective to choose a target that is as low as possible (e.g., as close to <40 g as possible). At 3 months, we expect different participants will have different levels of intake, with some consuming <40 g of net carbohydrates and others consuming levels higher than 60 g net carbohydrates per day. We have designed the intervention to accommodate various scenarios, with the goal of achieving as low of an intake as possible. For participants who consume >60 g per day, we will recommend they aim to either reduce or maintain carbohydrate intake.

At a brief check-in at 3-months, if participants are satisfied with their current intake, we will instruct them to stay at this intake level (though will recommend they try to reduce intake, if possible, if they are eating >60 net grams of carbohydrates or have not yet reached the study goal of <40 net grams of carbohydrates). If they prefer to make modifications, we will provide guidance on how to modify carbohydrate intake (either decrease or increase). If they choose to add back carbohydrates, we will recommend that participants add 5 grams of carbohydrates for two weeks at a time (e.g., from 40 to 45 g for 2 weeks), for a maximum total of <60 g net carbohydrates. If they choose to further reduce carbohydrate intake, we will provide guidance on this as well. As they modify carbohydrate intake, we will have them monitor their satisfaction with their diet and energy levels. If adding back carbohydrates, we will recommend participants

add back in foods like fruit (preferably berries), legumes, beans, and dairy, while still aiming to keep total carbohydrate intake as low as possible (e.g., as close to <40 net grams as able).

Materials: We will develop a handbook that will be given to participants. This will contain diet guidelines, recipes, meal planners, shopping lists, and guides for counting carbohydrates and reading nutrition labels.

Meals: Participants will select their own menus and prepare or buy their meals according to the guidelines. We will provide participants with supplemental low-carbohydrate foods, such as nuts, olive oil, and other products, to help facilitate their compliance.

Counseling: Sessions and materials will focus on instructing participants to reduce digestible carbohydrate consumption by increasing consumption of protein and fat, with a particular emphasis on increased consumption of heart-healthy unsaturated fats. After randomization, participants will begin the 3-month *Go Low* phase consisting of individual counseling sessions each week for the first four weeks, followed by 4 group sessions held every other week with phone follow-ups occurring in between group sessions. During the *Keep it Low* phase, months 4 through 6, participants will attend 3 monthly group sessions and have 3 telephone follow-ups. There will be a total of 11 in-person sessions and 7 telephone follow-ups over the full study period. Sessions will be approximately one hour and phone calls will be no longer than thirty minutes. These sessions will consist of diet instruction and supportive counseling, addressing results from 24-hour dietary recalls, teaching self-monitoring and individual diet goal changes, and provide suggestions for replacing usual foods containing carbohydrates. Study materials will be modified from materials used in the successful MACRO study. The JFI will work closely with Drs. He and Bazzano to develop the intervention based on the MACRO study.
Carbohydrate goal: In the MACRO study, carried out among individuals from the same study region, a behavioral intervention aimed at reducing digestible carbohydrate intake to <40 g/day led to reductions in weight and improvements in cardiovascular risk factors among individuals with obesity.[30] Additionally, restriction of carbohydrate intake to <50g/day is sufficient to induce ketosis.[99] Our rationale for having a multi-phased intervention is to allow participants to see how they feel on the more restrictive diet, and see how they respond to small increases in carbohydrate consumption. A similar technique was applied in the DIETFITS published study.[100] The higher target of 60 net grams may be more sustainable long-term and also allows for consumption of healthy higher-carbohydrate foods, such as berries, legumes, beans, and limited whole grains.

8.B. Usual Diet Group

We will provide participants randomized to the usual diet group written information with standard dietary advice. Participants randomized to the usual diet group will not receive ongoing dietary recommendations. Moreover, they will continue to receive clinical care recommendations from their physician(s). To encourage continued participation in the study and minimize the difference in participant contact with study staff, participants will be offered optional monthly educational sessions on topics unrelated to the study intervention, such as retirement planning or health insurance options.

In another effort to encourage continued participation, patients randomized to the usual diet group will be offered a copy of all available intervention materials, as well as an optional one-hour counseling session with the interventionist following their final study visit.

8.C. Physical Activity

At baseline, both groups will receive written information in study materials with standard physical activity recommendations.

8.D. Dietary Adherence

Strategies for measuring compliance: For the low-carbohydrate diet, adherence will be assessed through measurement of urinary ketones at 3-month visit and 6-month TV. Study staff will make all reasonable effort to maintain adherence of participants to the visit schedule and diet intervention. Walnut consumption will be tracked throughout the study in the low-carbohydrate diet group.

Strategies for improving compliance:

- At the screening visit, we will ask participants who does most of the cooking in their households and how often they eat outside of the home. Results from the screening visit will help to select a group of cooperative study participants.
- Visits will be scheduled at the participants' convenience and to minimize waiting time and trips to the clinic; reminder calls, texts or emails (chosen based on participant preference) will also be made prior to clinic visits.
- We will hire enthusiastic staff who have good interpersonal skills.
- Participants will receive $40 gift cards for use at food stores or retailers when they complete each study visit, for a maximum of $120 over the study period.
- Participants will receive $25 gift cards when they return the stool specimens, for a maximum of $75 over the study period.
- Participants will be offered small gifts (such as pens or magnets with study logo) to encourage compliance and adherence to the study protocol.
- Participants will be provided with recipes, shopping lists, and food samples for the intervention diet.

9. Safety of Dietary Intervention

On the low-carbohydrate diet, participants will be encouraged to primarily focus on replacing carbohydrates (particularly highly refined carbohydrates) with foods such as non-starchy vegetables, olive oil and other plant-based unsaturated oils, nuts and seeds, avocados, eggs, seafood, tofu, poultry, and lean meat. Consumption of more unsaturated fats, protein, and fiber will be recommended. Participants will not be encouraged to increase consumption of saturated fats and will be advised to avoid consumption of trans fats.

In animal studies, a high-protein diet has decreased renal function[101] In the MACRO study, on average, participants tended to increase consumption of fats, rather than protein.[30] The safety and tolerability of low-carbohydrate diets have previously been assessed in many studies, including MACRO.[30] A number of studies, including some in individuals with T2D, have found no clinically significant increases in estimated glomerular filtration rates,[33,38–40] microalbuminuria,[102] albumin excretion rate,[33,38] urinary albumin,[38] serum creatinine,[38,40,102–107] albumin,[40] or uric acid levels.[103–106] A small study of a very low-carbohydrate diet found significant increase in uric acid excretion, but no significant change in 24-hour urine creatinine clearance.[106]

Study staff will carefully monitor participants' BP, and HbA1c more often than what would occur in routine healthcare visits. A Data Safety and Monitoring Board (DSMB) consisting of an independent group of experts in areas such as biostatistics, nutrition medicine, general internal medicine, and endocrinology has been formed. The board will regularly review trial progress, including unblinded interim results, and can recommend that the trial prematurely end if participants are at risk or if it becomes clear that the trial will not be able to yield statistically significant results.

10. Data Collection

10.A. Study visits

After the initial pre-screening interview, in which data on medications and medical conditions will be collected, there will be one screening visit, one randomization visit, a follow-up study visit at 3 months and a termination visit (TV) at 6 months. *Table 3* summarizes study procedures and data collected at each study visit.

Table 3. Study visit schedule and data collection

	Pre-screen	SV	RZ	3 month	6 month TV
Pre-screen eligibility	X				
Informed consent		X			
Compliance form		X			
Eligibility confirmation			X		
Randomization			X		
Demographics		X			
Medical history questionnaire			X	X	X
Medications		X	X	X	X
Lifestyle (alcohol, cigarette smoking)			X	X	X
Physical activity			X	X	X
24-hour diet recall*			X	X	X
3 BP measurements		X	X	X	X
Body weight			X	X	X
Height			X		
Waist circumference			X	X	X
Spot urine test			X	X	X
Blood samples		X	X	X	X
Stool specimen			X	X	X
Side-effects assessment			X	X	X
Nut Consumption					X

*Two 24-hour dietary recalls will be collected; one should cover a weekend and the other a weekday.

10.A.1. Screening/baseline visits:

Participants will be pre-screened by phone or in-person by study staff to confirm eligibility for the Screening Visit. An approved questionnaire will be used to ensure the patient is eligible to attend the in-clinic screening visit, and the patient will be scheduled thereafter.

At the screening visit (SV), participants will provide informed consent for the screening and main study. Study staff will schedule the SV and randomization (RZ) visit at least 2 days apart. If a participant is eligible based on the screening visit, he or she should be scheduled for the RZ

once a sufficient group of eligible individuals has been recruited. Participants should be scheduled for the RZ at least 2 days and no more than two weeks after the screening visit.

At the screening visit, we will collect information on inclusion/exclusion criteria, medication use, and demographics. At this visit, we will also have participants complete a compliance form, to assess their ability to complete the study and attend all intervention and study visits. We will also measure blood pressure, and take blood samples. Women will be asked if they are postmenopausal or if they had surgical sterilization (such as a tubal ligation). If not, they will be tested for pregnancy through a spot urine test; pregnant women will be excluded from the study. Blood samples will be collected for measurement of HbA1c, and, if not available from medical records in the past 3 months, serum creatinine and hemoglobin. These blood samples will be used for excluding individuals with HbA1c <6.0 or >6.9, eGFR ≤45 mL/min/1.73 m^2, or hemoglobin <11 mg/dL.

Before the start of the intervention, participants will complete two 24-hour dietary recalls. At least one (and potentially both) 24-h recalls will be collected by phone. At the RZ, participants will learn about the randomization process. After requirements for eligibility have been satisfied, participants will formally be enrolled into the study and randomized to either the active intervention or control group. We will collect fasting blood samples for later measurement of glucose, insulin and lipids and other potential future measurements. At this visit, we will measure blood pressure, body weight, height, and waist circumference. We will collect information on medical history, lifestyle risk factors, and physical activity. A spot urine sample will be collected to measure ketones and other markers and participants will be asked to collect a stool specimen for potential use in future microbiome studies.

10.A.2. Follow-up visits and final visit: A follow-up visit will occur at 3 months after randomization. A final visit will occur at 6-months post-randomization. These visits should be scheduled as close as possible to the 3-, or 6-months after randomization. At each visit, study staff will conduct dietary recalls and anthropometric measures, take 3 BP measurements, collect a fasting blood sample to measure HbA1c. At least one (and potentially both) 24-h recalls will be collected by phone. We will also collect samples for later measurement of glucose, insulin, lipid profile, and other potential future measurements. A spot urine sample will be collected to measure ketones and other markers. Participants will be asked to collect a stool specimen for potential use in future microbiome studies. Information about participants' nut consumption over the past 6 months will be collected at the 6-month TV. At study termination, we will mail participants and/or their physicians a summary of trial results and a record of participant BP, anthropometric measures, and laboratory data from the trial period. Throughout the study period, we will provide participants with their blood pressure and weight. We will request that, if possible, a participant who is prescribed a diabetes medication during the course of the study comes into the clinic for an additional separate supplemental visit for a fasting blood sample collection prior to starting the medication.

10.B Study measurements

Measurements obtained for this study will be based on those used in other clinical trials and epidemiologic studies. We are familiar with strengths and limitations of such measurements and the importance of staff training and certification and use of stringent quality control procedures.

10.B.1. Questionnaires: Study staff will administer a *medical history questionnaire* at the RZ, 3-month visit and 6-month TV to assess medical conditions that would necessitate changes in therapy or medical management beyond this study's scope. At the SV, *demographic factors* will be collected. At the SV, RZ, 3-month and 6-month TV, we will also administer *medication*

tracking questionnaires. At RZ, 3-month, and 6-month TV, we will collect health behavior and physical activity data.

10.B.2. Dietary measurements: Two 24-hour dietary recalls will be obtained from participants at baseline. At or close to both the 3-month and 6-month TV, two more 24-hour dietary recalls will be collected. The second sets of two recalls will be collected as closely as possible to 3- or 6-month dates after randomization, with a goal of being within 2 weeks of the respective follow-up period. For each time point, one recall should reflect week day consumption, while the other should reflect weekend day consumption and the two recalls should be at least 2 days apart from each other. We will use computer software (Nutrition Data System for Research [NDS-R]) and food models used in previous and ongoing studies. Recalls will be conducted by a study staff member who has been certified in the use of NDS-R. Food composition tables of the NDS-R will be used to calculate dietary nutrient intakes. The baseline dietary assessment will be done to characterize individual dietary intake of carbohydrates and other macronutrients. During the intervention, repeated measurements will be used to monitor short-term changes in mean dietary nutrient intake between the intervention and usual diet groups.

10.B.3. Assessment and management of adverse effects: At RZ, 3-month visit, and 6-month TV, we will administer *symptom questionnaires*, which will consist of a checklist to identify participant complaints. Prior studies of low-carbohydrate dietary interventions suggest that participants may experience a range of adverse effects, including constipation, halitosis, fatigue, headache, thirst, polyuria, and nausea. Severe adverse events will be promptly reported to the DSMB. Management of adverse effects will be based on the protection and safety of the participant. If severe adverse effects are reported by a participant, carbohydrate intake will be examined and altered in order to mitigate the adverse effect. In any extreme cases, participants will be instructed to stop the intervention. As this is a behavioral intervention, adverse effects should be rare.

10.B.4. BP and anthropometric measures: BP will be measured 3 times at the screening visit and at each follow-up visit. To minimize random error, averages of the measurements at the screening and randomization visits will be taken. BP will be measured by a standard automated blood pressure measurement device (OMRON HEM-907 XL Professional Digital Blood Pressure Monitor) and performed by experienced staff, trained and certified in BP measurement. Prior to BP measurements, participants will be asked to rest quietly in a seated position with their feet supported for at least 5 minutes; staff members will use an appropriate cuff size over a bare arm. Trained and certified staff will measure weight using a calibrated balance beam scale at the screening visits, and follow-up visits. Weight and height will be measured at the RZ, and weight will also be measured at the subsequent visits.

10.B.5. Blood samples: For SV, participants will not need to fast. For RZ, 3-month visit, and 6-month TV, participants will be instructed to fast for 12 hours prior to blood sample collection. At SV, blood samples will be collected to measure HbA1c, hemoglobin, and serum creatinine. At RZ, fasting blood samples will be collected and stored as serum/plasma, whole blood aliquots and buffy coats at -80°C for future analyses, including measurement of glucose, insulin and lipids, and potential future metabolomics or genetic analyses. At 3-month visit and 6-month TV, we will collect fasting blood samples to measure HbA1c and will also store additional serum/plasma, whole blood aliquots and buffy coats at -80°C for future analyses, including measurement of glucose, insulin and lipids, and potential future metabolomics or genetic analyses. Details of the blood sample collection are shown in *Table 4*. HOMA-IR will be calculated from fasting glucose and insulin.

Table 4. Study visit schedule for specimen collection

	SV	RZ	3 month	6 month TV
HbA1c	X		X	X
Serum creatinine	X			
Hemoglobin	X			
Storage of aliquots and buffy coats (for measurement of glucose, insulin, lipids, other potential future measurements)		X	X	X
Urine sample for pregnancy	X			
Urine sample for ketones and other markers		X	X	X
Stool sample		X	X	X

10.B.6. Urine collection: Study staff will collect spot urine specimens at RZ, interim visit, and the final visit to measure ketones.

10.B.7. Stool Specimen Collection: Stool samples will be collected from all eligible and willing trial participants during the RZ, 3-month, and 6-month TV, following the protocol set forth by the Human Microbiome Project.[108,109] Participants will be given a stool collection kit, which includes the specimen collection container, a shipper for home collection, and a form to record the timing and pertinent information related to specimen collection. Participants will be instructed to return the kit to the clinic within two days of collection (in-person or via shipping), at which time stool samples are stored at -80°C until analyses. Kits and instructions for collection will be reviewed by study staff with the participant at the required visits.

11. Quality Assurance and Quality Control

Throughout the study, we will implement rigorous quality assurance (QA) and quality control (QC) measures. We describe some of these approaches here.

11.A. Manual of procedures

We will develop a manual of procedures (MOP) based on the MOP successfully used in the MACRO behavior change clinical trial. The MOP will provide instructions and describe procedures for staff training and certification, participant recruitment, intervention counseling, study form completion and procedures, data entry, safety measures (e.g., methods for emergency participant/primary care physician contact).

11.B. Training and certification

Trial personnel will all be required to participate in a training session before the start of any trial procedures or measurements. Training will include particular attention to core trial elements, such as recruitment, retention, intervention, completeness and quality of data acquisition (e.g., blinding procedures for the data collectors), and data entry. Separately, intervention staff will meet and receive detailed instruction on trial approaches to group and individual behavior change counseling and procedures that ensure data collectors are blinded to treatment assignment. Throughout the trial, periodic re-training/certification sessions will be

conducted to ensure all trial-related procedures are being applied in a standard manner. Investigators will also monitor all aspects of study performance to ensure study goal achievement, timeliness and completeness of study visits, completeness and quality of data collection, intervention adherence, and adequacy of the procedures separating data collection from intervention. Study staff and investigators will pay particularly careful attention to the quality of all laboratory measurements, particularly key exposure, outcome, and intervention monitoring variables.

12. Sample Size and Statistical Power

The primary outcome will be the difference in the change of HbA1c between the intervention and usual diet groups from baseline to 6 months of follow-up. In the United States National Health and Nutrition Examination Survey, when sub-setting to the range of HbA1c of 6.0–6.9% among individuals who do not report use of diabetes medication, the cross-sectional standard deviation is 0.24% using data from 2000 through 2016 (n=4117; https://wwwn.cdc.gov/nchs/nhanes/). In studies with 6-month follow-up among individuals with HbA1c 5.7 to 6.4% or other characteristic(s) (e.g, elevated fasting glucose or metabolic syndrome) that put them at elevated risk of type 2 diabetes, the within-person correlation between baseline and follow-up HbA1c was estimated to be between 0.5 and 0.84.[110–113] As all participants in our study will be within a specific range of HbA1c at baseline, we expect this may lead to smaller within-person correlation of HbA1c over time than studies that do not restrict to a narrow range of HbA1c at baseline. Assuming a within-person correlation of 0.5 and an SD of 0.24% for change in HbA1c, we would need to have 90 individuals to have 85% power to detect an approximate difference in change of HbA1c of 0.15% between the intervention and usual diet groups.

In a follow-up analysis of the DPP, a 0.15% decrement in HbA1c at 6 months was associated with a 16% lower risk of incident T2D, adjusting for treatment arm, age, sex, race/ethnicity, household income, and baseline HbA1c.[115] In a pooled analysis from the Atherosclerosis Risk in Communities and Framingham Heart Studies, a 0.15% higher HbA1c was associated with an 16% higher risk of T2D over 20 years, a 30% higher risk over 8 years, and a 13% higher risk after 8 years, after adjusting for age, sex, fasting glucose, triglycerides, HDL, family history of T2D, SBP, and BMI.[116] Additionally, in EPIC-Norfork, a difference of HbA1c of 0.15% was associated with approximately a 5% higher risk of CHD among those free of CHD, stroke, diabetes, and with HbA1c <7.0%.[117]

Table 5. Minimum detectable differences

	SD	Detectable Difference
HbA1c, %	0.24	0.15
Fasting glucose, mg/dL	12.5	8.0
Fasting insulin, mU/mL	7.0	4.5
HOMA-IR	2.0	1.3
SBP (mmHg)	4.2	2.7
DBP (mmHg)	3.0	1.9
Total/HDL ratio	1.2	0.77
Body weight (kg)	6.6	4.2
Waist circumference (cm)	8.0	5.1
Estimated CVD risk (%)*	6.7	4.8

*10-y CVD risk, among estimated 85% or 76 participants free of CVD

In the DPP study, HbA1c was 0.15% lower among those in the lifestyle intervention arm than in the control arm at 6-months follow-up; HbA1c at study commencement was 5.9% and levels increased in the control arm and decreased in the behavioral intervention arm.[22] In a small behavioral intervention study (n=34) of a very low-carbohydrate ketogenic diet compared with a moderate carbohydrate, calorie-restricted, low-fat diet among individuals with type 2 diabetes or prediabetes (HbA1c >6.0%) and elevated body weight (BMI >25), mean HbA1c decreased from 6.6% to 6.1% in the very low-carbohydrate group and from 6.9% to 6.7% in the

moderate carbohydrate group over a 12-month period; additionally, the very low-carbohydrate group had greater reductions in glucose-lowering medications.[37]

Detectable differences are listed for HbA1c and secondary outcomes in *Table 5*, based on data from prior studies.[30,53,56,118] These were calculated for unpaired t-tests, power of 85%, two-sided alpha of 0.05, and assuming 80% follow-up of 112 participants, which would yield an estimated 90 participants.

13. Data Management and Analysis

13.A. Data management

Personnel will double-enter all study data. Prior to entering data, the study coordinator will conduct quality control on all study forms, study measures, and laboratory data. Data will be cleaned and logically checked.

13.B. Data analysis plan

The data will be analyzed using <u>*intention to treat*</u>; this involves comparing the primary and secondary outcomes between participants based on randomization assignment.

13.B.1. Exploratory data analysis: We will express group data for variables with a normal distribution as mean ± standard deviation and for variables not normally distributed as median and interquartile ranges (or will log-transform the variables to obtain a normal distribution). We will compare means and percentages of baseline variables across the intervention and usual diet group to assess similarity of these groups at randomization.

13.B.2. Primary, secondary, and exploratory outcomes: We will test the primary study hypothesis that there is a greater reduction in HbA1c from baseline to 6 months in the intervention group than in the usual diet group. To do this, we will use a mixed-effects linear regression model, to test the null hypothesis that there is no difference in change between baseline and 6-month HbA1c for the intervention group compared with the usual diet group (Specific Aim 1). To do this, we will use the following model:

$$Y_{i,j,k}|T = b_0 + b_1G_i + b_2T_{3\text{-months}} + b_3T_{6\text{-months}} + b_4G_iT_{3\text{-months}} + b_5G_iT_{6\text{-months}} + a_{0i,j} + e_{i,j,k}$$

where $Y_{i,j,k}$ is HbA1c for the jth participant (P) nested in the ith intervention group (G) at time T_k (baseline, 3 months, and 6 months). This model includes a random intercept ($a_{0i,j}$) and error term $e_{i,j,k}$. The interaction term ($b_5G_iT_{6\text{-months}}$) allows for an interaction between group and $T_{6\text{-months}}$, which is equal to 0 for baseline and 3-months and 1 for 6-months. Rejection of the null hypothesis (b_5=0) indicates there is a significant difference in change of HbA1c from baseline to 6 months between the intervention and control groups. In this model, participants are assumed to be random effects and intervention group, time, and the interaction terms are fixed effects.

We will use a similar approach to test the difference in the change between baseline and 6-month values of secondary outcomes (Specific Aim 2) and exploratory outcomes. In exploratory analyses, we will also test the difference in the change between baseline and 3-month values of outcomes (HbA1c, fasting glucose, systolic blood pressure, total-to-HDL cholesterol, body weight, insulin, HOMA-IR, diastolic blood pressure, waist circumference, and estimated CVD risk). We will also explore the use of mixed effects models to assess overall net change in HbA1c (by treating time as a linear variable) and other outcomes over the full study period. For each outcome, we will test modeling assumptions and will consider transformation of outcomes, if needed, to meet modeling assumptions. As the mixed-effects model assumes missing at random, we will also perform sensitivity analyses using Markov-chain Monte Carlo techniques to impute missing values of the outcomes, using covariates for this imputation and will compare results from this analysis with our primary findings.

15. Anticipated Challenges

Recruitment: Many clinical trials have challenges recruiting sufficient numbers of volunteers who meet the trial inclusion and exclusion criteria and demonstrate a high level of adherence to trial protocol requirements. Fortunately, the investigators are very experienced with clinical trial recruitment, and have several contingency options that can be used to meet unanticipated shortfall in recruitment.

Intervention: The investigators are aware of and understand challenges related to achieving and maintaining compliance with a behavioral intervention. The investigators have much experience and success in behavioral interventions, with recent success of a behavioral intervention aimed at reducing carbohydrate intake among participants from the New Orleans area. The JFI will collaborate with Drs. He and Bazzano to develop the intervention program based on the MACRO study and will assess potential barriers to implementation and intervention adherence (such as financial concerns and range of dietary patterns of study participants) during the planning and pilot phase of the study.

Adherence: Though adherence and follow-up are also main challenges related to clinical trials, the investigators have an outstanding track record for studies that have recruited participants for both clinical and epidemiology studies in the Greater New Orleans area. To encourage adherence, small gifts (such as pens or magnets with study logo) will be offered to all study participants.

16. Protection of Human Subjects

This Human Subjects Research meets the definition of a clinical trial. All investigators will be required to complete the CITI (Collaborative Institutional Training Initiative) program in the ethical use of human participants in research.

16.A. Protection of human subjects from research risks

16.A.1. Risks to the subjects

Human subject involvement and characteristics: We expect to enroll 112 participants, with an equal male to female ratio. We expect to enroll approximately 30 participants who are African-Americans, and another 10 with mixed racial backgrounds, and a few Asian participants given the demographics of the greater New Orleans area. Overall, given the demographics of greater New Orleans, we estimate approximately 10% participants of Hispanic or Latino ethnicity, 50% of them with African-American racial background.

Inclusion criteria

- Men or women ages 40 to 70 years: aim to recruit 50% women
- Any race or ethnicity: aim to recruit 40% ethnic minorities (i.e., African American and Hispanic), close to population in the greater New Orleans area[94]
- HbA1c 6.0–6.9%
- Willing and able to provide informed consent

Exclusion criteria

- Diagnosed type 1 diabetes mellitus based on patient self-report
- Use of agents affecting glycemic control (medications for diabetes, oral glucocorticoids) within the past three months prior to enrollment based on patient self-report

- Medical condition in which low-carbohydrate diet may not be advised
    - o Estimated glomerular filtration rate (eGFR) ≤45 mL/min/1.73 m², which is close to the 5th percentile of eGFR among non-diseased individuals of 70 years of age[95]
    - o Self-report of liver disease due to hepatitis or alcohol; osteoporosis; untreated thyroid disease; gout; or cancer (other than non-melanoma skin cancer) requiring treatment in the past year, unless prognosis is excellent
- Factors that may affect HbA1c:
    - o Hemoglobin <11 mg/dL (cutpoint for moderate-to-severe anemia, which could lead to falsely elevated or lowered HbA1c)[96]
    - o Recent blood donation or blood transfusion (self-report, past 4 months)
    - o Human Immunodeficiency Virus (self-report)[97]
- Allergies to nuts
- For women, current pregnancy, breastfeeding, or plans to become pregnant during the study
- Consumption of ≥21 alcoholic drinks per week or consumption of ≥6 drinks per occasion
- Current or planned residence making it difficult to meet trial requirements (due to distance from study site and/or challenges regularly traveling to site)
- Current participation in another lifestyle intervention trial or a pharmaceutical trial
- Participation of another household member in the study; employees or persons living with employees of the study
- Other concerns regarding ability to meet trial requirements, at the discretion of the principal investigator or study coordinator

Sources of materials: Medical information data will be collected from the participant and, after written authorization for the disclosure of protected health information, from participants' medical records. Blood and urine samples will be collected to measure HbA1c, glucose, insulin, lipids, creatinine, ketones, and test for pregnancy. Blood pressure, height, weight, waist circumference and other physical measurements will be obtained.

Potential Risks: Risks associated with venipuncture, the diet intervention, and collection of confidential medical information are the main risks associated with this study. There is a small risk of bruising and a rare chance of local infection associated with standard venipuncture to collect blood samples. The dietary intervention has minimal risks. Reduced carbohydrate consumption may lead to adverse effects, including constipation, halitosis, fatigue, headache, thirst, polyuria, and nausea. At RZ, 3- , and 6-month visits, we will administer *symptom questionnaires*, which will consist of a checklist to identify participant complaints. Adverse effects of the low-carbohydrate diet will be assessed at counseling sessions. Severe adverse events will be promptly reported to the DSMB. Management of adverse effects will be based on the protection and safety of the participant. If severe adverse effects are reported by a participant, carbohydrate intake will be examined and altered in order to mitigate the adverse effect. In any extreme cases, participants will be instructed to stop the intervention. Furthermore, there is a potential risk of unauthorized disclosure of medical information. This risk will be minimized by using a strict protocol for data processing and by the appropriate training of all study personnel. Protection against risks is described in more detail below.

16.A.2. Adequacy of protection against risks

Recruitment and Informed Consent: The proposed study will be conducted following strict guidelines for the protection of the rights of human volunteers. The informed consent document will be signed by all participants at the screening/baseline visit. The informed consent form will be developed in the first year of the study and approved by the IRB. The consent form will clearly state the purpose, eligibility criteria, and protocol of the study, the potential benefits and risks of participation, and the subject's right to refuse to participate or withdraw. Prior to signing the informed consent, research staff will review the details of the consent form orally with the participant, and answer any questions that the participant has concerning participation in the study. In the case of potential participants with low or no literacy, the informed consent form will be read to the participant in its entirety and any questions will be answered prior to the participant signing or marking the form. Specifically, the following must be accomplished during the informed consent process:

- The participant must be informed that participation in the study is voluntary and that refusal to participate will involve no penalty or loss of benefits.
- The participant must be informed of the purpose of the study and that it involves research.
- The participant must be informed of any alternative procedures, if applicable.
- The participant must be informed of any reasonably foreseeable risks.
- The participant must be informed of any benefits from the research.
- An outline of safeguards to protect participant confidentiality will be included, as well as an indication of the participant's right to withdraw without penalty. This should be balanced with a discussion of the effect that withdrawals will have on the study, and the responsibility a participant has, within limits, to continue in the study if he or she decides to enroll.
- The participant must be informed of his or her right to have questions answered at any time and whom to contact for answers or in the event of research-related injury.
- The participant must be informed as to whether or not compensation is offered for participation in the study and/or in the event of a medical injury.
- The participant must be informed that he/she will be notified of any significant changes in the protocol that might affect their willingness to continue in the study.

Written authorization for the disclosure of protected health information will be obtained during the consent process on a separate form (HIPPA authorization), if applicable.

Protection against Risk: The protocol and informed consent will be approved by the Institutional Review Boards at Tulane University Health Sciences Center and any other participating institution for recruitment. The study participants will be given contact information for the Study Coordinator and Study PI to answer questions or to report complaints. Patients can withdraw from the study at any time without any effect on their medical care. Standard techniques will be used by trained, certified phlebotomists or study nurses for blood sample collection at the study clinics where emergency medical equipment is available. Confidentiality of all data collected during the study will be maintained through the use of unique, encrypted study identification (ID numbers, rather than patient names, in the study database). These encrypted ID numbers will not be mathematical derivation of the subject's medical record number or any other patient identifier. No information will be disclosed in an individually identifiable form in any type of presentation or publication. There will be password-required access to the computerized file linking the study numbers to patient identifiers at the study clinics. Password-access will be restricted to pertinent research staff only. All study data will be kept in locked file cabinets in locked rooms, accessible only by study staff with permission. All

data transmissions will use HIPPA-compliant password protected system and will be secured through a virtual private network.

16.A.3. Potential benefits of the proposed research to human subjects and others

Study participants may have improved glycemic outcomes, blood pressure, lipid levels, may lose weight and reduce waist circumference, and may have improvement in other risk factors for CVD and development and progression of T2D. Participants will receive multiple blood pressure, weight, waist circumference, and blood tests. If vital signs or lab values are clinically abnormal, participants will be referred to their primary care physician, or if they do not have a primary care physician, one will be assigned through the Tulane University Community Health Center, which is a part of the core clinical facilities. At the end of the study, we will provide the participants' primary care physician with a copy of all the blood pressure, weight, and laboratory results.

16.A.4. Importance of knowledge to be gained

Information from this study may contribute to reducing the onset of T2D and the burden of T2D and cardiovascular disease in Louisiana and the general US population. The study has potential for public health impact; results from this study may provide evidence in support of promoting alternative dietary styles (than the most commonly used low-fat diet) among individuals with high T2D risk or with T2D.

16.A.5. Remuneration

For screening visit 2, the two follow-up visits, and the final visits, participants will receive $40 gift cards for use at food stores or retailers (after completing each study visit), for a maximum of $120 over the study period. Participants who return a stool sample will receive $25 gift cards for each stool sample that they return, for a total of $75 for three stool samples. Participants will also receive free parking for each intervention and study visit.

16.B. Data safety and monitoring plan

A DSMB, which consists of 3 experts who are not otherwise affiliated with the COBRE program, has been formed. Study investigators for this study will prepare accurate and timely data tables and reports for the DSMB meetings. The members of the DSMB will serve in an individual capacity and provide their expertise and recommendations. The primary responsibilities of the DSMB are: 1) periodically review and evaluate the accumulated study data for participant safety, study conduct and progress, and, when appropriate, efficacy, and 2) make recommendations to the NIH concerning the continuation, modification, or termination of the trial. The DSMB considers study-specific data, as well as relevant background knowledge, about the disease, test agent, and patient population under study.

The DSMB is responsible for defining its deliberative processes, including event triggers that would call for an unscheduled review, stopping guidelines, unmasking (unblinding) and voting procedures prior to initiating any data review. The DSMB is also responsible for maintaining the confidentiality of its internal discussions and activities, as well as the contents of reports provided to it.

The DSMB will review each protocol for any major concerns prior to implementation. During the trial, the DSMB should review cumulative study data to evaluate safety, study conduct, and scientific validity and integrity of the trial. As part of this responsibility, DSMB members must be satisfied that the timeliness, completeness, and accuracy of the data submitted to them for review are sufficient for evaluation of the safety and welfare of study participants. The DSMB

should also assess the performance of overall study operations and any other relevant issues, as necessary.

Items reviewed by the DSMB include:

- Interim/cumulative data for evidence of study-related adverse events;
- Interim/cumulative data for evidence of efficacy according to pre-established statistical guidelines, if appropriate;
- Data quality, completeness, and timeliness;
- Adequacy of compliance with goals for recruitment and retention, including those related to the participation of women and minorities;
- Adherence to the protocol;
- Factors that might affect the study outcome or compromise the confidentiality of the trial data (such as protocol violations, unmasking, etc.); and,
- Factors external to the study, such as scientific or therapeutic developments, that may impact participant safety or the ethics of the study.

The DSMB will conclude each review with their recommendations to the COBRE program and NIH/NIGMS as to whether the study should continue without change or be modified or terminated. Recommendations regarding modification of the design and conduct of the study could include:

- Modifications of the study protocol based upon review of the safety data;
- Suspension or early termination of the study or of one or more study arms because of serious concerns about subjects' safety, inadequate performance or rate of enrollment;
- Suspension or early termination of the study or of one or more study arms because study objectives have not been obtained according to pre-established statistical guidelines; and,
- Corrective actions regarding a study when the performance appears unsatisfactory or suspicious.

Confidentiality must always be maintained during all phases of DSMB review and deliberation. Usually, only voting members of the DSMB should have access to interim analyses of outcome data by treatment group. Exceptions may be made when the DSMB deems it appropriate. The reason and to whom the exceptions for access to interim analyses is granted will be documented in the Closed Session Report. DSMB members must maintain strict confidentiality concerning all privileged trial results ever provided to them. The DSMB should review data only by masked study group (such as X vs. Y rather than experimental vs. control) unless or until the DSMB determines that the identities of the groups are necessary for their decision-making. Whenever masked data are presented to the DSMB, the key to the group coding must be available for immediate unmasking.

The DSMB will meet twice per year. The junior faculty investigator will attend these meetings and will be responsible for preparing and presenting up-to-date statistical reports on the progress of their trials. These reports will include data on recruitment, randomization, adherence, as well as statistical tests and special analyses requested by the DSMB. The Administrative Core will be responsible for organizing the annual in-person meeting and semiannual conference calls. They will put together data and materials submitted by junior faculty investigators and deliver them to DSMB members. The COBRE investigators will meet with the DSMB in the Open Session and not be present for the Closed Session.

Bibliography and References Cited

1. The Emerging Risk Factors Collaboration. Diabetes mellitus, fasting blood glucose concentration, and risk of vascular disease: a collaborative meta-analysis of 102 prospective studies. *Lancet*. 2010;375(9733):2215-2222. doi:10.1016/S0140-6736(10)60484-9.
2. Flaxman SR, Bourne RRA, Resnikoff S, et al. Global causes of blindness and distance vision impairment 1990-2020: a systematic review and meta-analysis. *Lancet Glob Heal*. October 2017. doi:10.1016/S2214-109X(17)30393-5.
3. United States Renal Data System. *2016 USRDS Annual Data Report: Epidemiology of Kidney Disease in the United States*. Bethesda, MD: National Institutes of Health, National Institute of Diabetes and Digestive and Kidney Diseases; 2016. https://www.usrds.org/2016/view/Default.aspx.
4. Narres M, Kvitkina T, Claessen H, et al. Incidence of lower extremity amputations in the diabetic compared with the non-diabetic population: A systematic review. *PLoS One*. 2017;12(8):e0182081. doi:10.1371/journal.pone.0182081.
5. Centers for Disease Control and Prevention. *National Diabetes Statistics Report, 2017*. Atlanta, GA: Centers for Disease Control and Prevention, U.S. Dept of Health and Human Services; 2017.
6. Centers for Disease Control and Prevention. *Long-Term Trends in Diabetes*. Atlanta, GA: Centers for Disease Control and Prevention, U.S. Dept of Health and Human Services; 2017.
7. NCD Risk Factor Collaboration (NCD-RisC). Worldwide trends in diabetes since 1980: A pooled analysis of 751 population-based studies with 4.4 million participants. *Lancet*. 2016;387(10027):1513-1530. doi:10.1016/S0140-6736(16)00618-8.
8. American Diabetes Association. Economic costs of diabetes in the U.S. in 2012. *Diabetes Care*. 2013;36(4):1033-1046. doi:10.2337/dc12-2625.
9. Zhuo X, Zhang P, Barker L, Albright A, Thompson TJ, Gregg E. The lifetime cost of diabetes and its implications for diabetes prevention. *Diabetes Care*. 2014;37(9):2557-2564. doi:10.2337/dc13-2484.
10. WHO, International Diabetes Foundation. *Definition and Diagnosis of Diabetes Mellitus and Intermediate Hyperglycaemia: Report of a WHO/IDF Consultation*. Geneva: Geneva: World Health Organization; 2006.
11. The International Expert Committee. International Expert Committee Report on the Role of the A1C Assay in the Diagnosis of Diabetes. *Diabetes Care*. 2009;32(7):1327-1334. doi:10.2337/dc09-9033.
12. American Diabetes Association. Standards of Medical Care in Diabetes - 2017. *Diabetes Care*. 2017;40(Supplement 1):S33-S43. doi:10.2337/dc16-S003.
13. Gerstein HC, Santaguida P, Raina P, et al. Annual incidence and relative risk of diabetes in people with various categories of dysglycemia: A systematic overview and meta-analysis of prospective studies. *Diabetes Res Clin Pract*. 2007;78(3):305-312. doi:10.1016/j.diabres.2007.05.004.
14. Huang Y, Cai X, Mai W, Li M, Hu Y. Association between prediabetes and risk of cardiovascular disease and all cause mortality: systematic review and meta-analysis. *BMJ*. 2016;355:i5953. doi:10.1136/bmj.i5953.
15. Tabák AG, Herder C, Rathmann W, Brunner EJ, Kivimäki M. Prediabetes: A high-risk state for diabetes development. *Lancet*. 2012;379(9833):2279-2290. doi:10.1016/S0140-

6736(12)60283-9.
16. Diabetes Prevention Program Research Group. 10-year follow-up of diabetes incidence and weight loss in the Diabetes Prevention Program Outcomes Study. *Lancet*. 2009;374(9702):1677-1686. doi:10.1016/S0140-6736(09)61457-4.
17. The DREAM (Diabetes REduction Assessment with ramipril and rosiglitazone Medication) Trial Investigators. Effect of rosiglitazone on the frequency of diabetes in patients with impaired glucose tolerance or impaired fasting glucose: a randomised controlled trial. *Lancet*. 2006;368(9541):1096-1105. doi:10.1016/S0140-6736(06)69420-8.
18. Perreault L, Pan Q, Mather KJ, Watson KE, Hamman RF, Kahn SE. Effect of regression from prediabetes to normal glucose regulation on long-term reduction in diabetes risk: Results from the Diabetes Prevention Program Outcomes Study. *Lancet*. 2012;379(9833):2243-2251. doi:10.1016/S0140-6736(12)60525-X.
19. Perreault L, Temprosa M, Mather KJ, et al. Regression from prediabetes to normal glucose regulation is associated with reduction in cardiovascular risk: Results from the diabetes prevention program outcomes study. *Diabetes Care*. 2014;37(9):2622-2631. doi:10.2337/dc14-0656.
20. Popkin BM. Nutrition Transition and the Global Diabetes Epidemic. *Curr Diab Rep*. 2015;15(9). doi:10.1007/s11892-015-0631-4.
21. Vazquez G, Duval S, Jacobs DRJ, Silventoinen K. Comparison of body mass index, waist circumference, and waist/hip ratio in predicting incident diabetes: a meta-analysis. *Epidemiol Rev*. 2007;29:115-128. doi:10.1093/epirev/mxm008.
22. Knowler WC, Barrett-Connor E, Fowler SE, et al. Reduction in the incidence of type 2 diabetes with lifestyle intervention or metformin. *N Engl J Med*. 2002;346(6):393-403. doi:10.1056/NEJMoa012512.
23. Pan XR, Li GW, Hu YH, et al. Effects of diet and exercise in preventing NIDDM in people with impaired glucose tolerance: The Da Qing IGT and diabetes study. *Diabetes Care*. 1997;20(4):537-544. doi:10.2337/diacare.20.4.537.
24. Tuomilehto J, Indstrom J, Eriksson J, Valle T, Hamalainine E, Uusitupa M. Prevention of Type 2 Diabetes Mellitus By Changes in Lifestyle Among Subjects With Impaired Glucose Tolerance. *N Engl J Med*. 2001;344(18):1343-1350. doi:10.1056/NEJM200105033441801.
25. Ramachandran A, Snehalatha C, Mary S, Mukesh B, Bhaskar AD, Vijay V. The Indian Diabetes Prevention Programme shows that lifestyle modification and metformin prevent type 2 diabetes in Asian Indian subjects with impaired glucose tolerance (IDPP-1). *Diabetologia*. 2006;49(2):289-297. doi:10.1007/s00125-005-0097-z.
26. The Diabetes Preventio Program Research Group. The Diabetes Prevention Program. Design and methods for a clinical trial in the prevention of type 2 diabetes. *Diabetes Care*. 1999;22(4):623-634. doi:10.2337/diacare.22.4.623 10.2337/diabetes.48.4.699.
27. Evert AB, Boucher JL, Cypress M, et al. Nutrition therapy recommendations for the management of adults with diabetes. *Diabetes Care*. 2013;36(11):3821-3842. doi:10.2337/dc13-2042.
28. Hu T, Mills KT, Yao L, et al. Effects of low-carbohydrate diets versus low-fat diets on metabolic risk factors: A meta-analysis of randomized controlled clinical trials. *Am J Epidemiol*. 2012;176(SUPPL. 7). doi:10.1093/aje/kws264.
29. Sackner-Bernstein J, Kanter D, Kaul S. Dietary Intervention for Overweight and Obese Adults: Comparison of Low-Carbohydrate and Low-Fat Diets. A Meta-Analysis. *PLoS*

One. 2015;10(10):e0139817. doi:10.1371/journal.pone.0139817.

30. Bazzano LA, Hu T, Reynolds K, et al. Effects of low-carbohydrate and low-fat diets: A randomized trial. *Ann Intern Med*. 2014;161(5):309-318. doi:10.7326/M14-0180.
31. Sawyer L, Gale EAM. Diet, delusion and diabetes. *Diabetologia*. 2009;52(1):1-7. doi:10.1007/s00125-008-1203-9.
32. Snorgaard O, Poulsen GM, Andersen HK, Astrup A. Systematic review and meta-analysis of dietary carbohydrate restriction in patients with type 2 diabetes. *BMJ Open Diabetes Res Care*. 2017;5(1):e000354. doi:10.1136/bmjdrc-2016-000354.
33. Larsen RN, Mann NJ, Maclean E, Shaw JE. The effect of high-protein, low-carbohydrate diets in the treatment of type 2 diabetes: A 12 month randomised controlled trial. *Diabetologia*. 2011;54(4):731-740. doi:10.1007/s00125-010-2027-y.
34. Saslow LR, Kim S, Daubenmier JJ, et al. A randomized pilot trial of a moderate carbohydrate diet compared to a very low carbohydrate diet in overweight or obese individuals with type 2 diabetes mellitus or prediabetes. *PLoS One*. 2014;9(4). doi:10.1371/journal.pone.0091027.
35. Tay J, Luscombe-Marsh ND, Thompson CH, et al. A very low-carbohydrate, low-saturated fat diet for type 2 diabetes management: A randomized trial. *Diabetes Care*. 2014;37(11):2909-2918. doi:10.2337/dc14-0845.
36. Davis NJ, Tomuta N, Schechter C, et al. Comparative Study of the Effects of a 1-Year Dietary Intervention of a Low- Carbohydrate Diet Versus a Low-Fat Diet on Weight and Glycemic Control in Type 2. *Diabetes Care*. 2009;32(7):1147-1152. doi:10.2337/dc08-2108.Clinical.
37. Saslow LR, Daubenmier JJ, Moskowitz JT, et al. Twelve-month outcomes of a randomized trial of a moderate-carbohydrate versus very low-carbohydrate diet in overweight adults with type 2 diabetes mellitus or prediabetes. *Nutr Diabetes*. 2017;7(12):304. doi:10.1038/s41387-017-0006-9.
38. Tay J, Thompson CH, Luscombe-Marsh ND, et al. Effects of an energy-restricted low-carbohydrate, high unsaturated fat/low saturated fat diet versus a high carbohydrate, low fat diet in type 2 diabetes: a 2 year randomized clinical trial. *Diabetes, Obes Metab*. 2017:n/a-n/a. doi:10.1111/dom.13164.
39. Yamada Y, Uchida J, Izumi H, et al. A non-calorie-restricted low-carbohydrate diet is effective as an alternative therapy for patients with type 2 diabetes. *Intern Med*. 2014;53(1):13-19. doi:http://dx.doi.org/10.2169/internalmedicine.53.0861.
40. Mayer SB, Jeffreys AS, Olsen MK, Mcduffie JR, Feinglos MN, Yancy WS. Two diets with different haemoglobin A1c and antiglycaemic medication effects despite similar weight loss in type 2 diabetes. *Diabetes, Obes Metab*. 2014;16(1):90-93. doi:10.1111/dom.12191.
41. Maekawa S, Kawahara T, Nomura R, et al. Retrospective study on the efficacy of a low-carbohydrate diet for impaired glucose tolerance. *Diabetes Metab Syndr Obes*. 2014;7:195-201. doi:10.2147/DMSO.S62681.
42. Davis C, Bryan J, Hodgson J, Murphy K. Definition of the mediterranean diet: A literature review. *Nutrients*. 2015;7(11):9139-9153. doi:10.3390/nu7115459.
43. Sofi F, Macchi C, Abbate R, Gensini GF, Casini A. Mediterranean diet and health status: an updated meta-analysis and a proposal for a literature-based adherence score. *Public Health Nutr*. 2014;17(12):2769-2782. doi:10.1017/S1368980013003169.
44. Jannasch F, Kröger J, Schulze MB. Dietary Patterns and Type 2 Diabetes: A Systematic Literature Review and Meta-Analysis of Prospective Studies. *J Nutr*. 2017;147(6):1174-

1182. doi:10.3945/jn.116.242552.

45. Schwingshackl L, Missbach B, König J, Hoffmann G. Adherence to a Mediterranean diet and risk of diabetes: a systematic review and meta-analysis. *Public Health Nutr*. 2014;18(7):1292–1299. doi:10.1017/S1368980014001542.

46. de Lorgeril M, Salen P, Martin J-L, Monjaud I, Delaye J, Mamelle N. Mediterranean Diet, Traditional Risk Factors, and the Rate of Cardiovascular Complications After Myocardial Infarction : Final Report of the Lyon Diet Heart Study. *Circulation*. 1999;99(6):779-785. doi:10.1161/01.CIR.99.6.779.

47. Estruch R, Ros E, Salas-Salvadó J, et al. Primary Prevention of Cardiovascular Disease with a Mediterranean Diet. *N Engl J Med*. 2013;368(14):1279-1290. doi:10.1056/NEJMoa1200303.

48. Salas-Salvadó J, Bulló M, Estruch R, et al. Prevention of diabetes with mediterranean diets: A subgroup analysis of a randomized trial. *Ann Intern Med*. 2014;160(1):1-10. doi:10.7326/M13-1725.

49. Esposito K, Marfella R, Ciotola M, et al. Effect of a Mediterranean-Style Diet on Endothelial Dysfunction and Markers of Vascular Inflammation in the Metabolic Syndrome. *JAMA*. 2004;292(12):1440. doi:10.1001/jama.292.12.1440.

50. Babio N, Toledo E, Estruch R, et al. Mediterranean diets and metabolic syndrome status in the PREDIMED randomized trial. *CMAJ*. 2014;186(17):E649-E657. doi:10.1503/cmaj.140764.

51. Esposito K, Maiorino MI, Bellastella G, Chiodini P, Panagiotakos D, Giugliano D. A journey into a Mediterranean diet and type 2 diabetes: a systematic review with meta-analyses. *BMJ Open*. 2015;5(8):e008222. doi:10.1136/bmjopen-2015-008222.

52. Micha R, Michas G, Mozaffarian D. Unprocessed red and processed meats and risk of coronary artery disease and type 2 diabetes--an updated review of the evidence. *Curr Atheroscler Rep*. 2012;14(6):515-524. doi:10.1007/s11883-012-0282-8.

53. Esposito K, Maiorino MI, Ciotola M, et al. Effects of a Mediterranean-style diet on the need for antihyperglycemic drug therapy in patients with newly diagnosed type 2 diabetes: A randomized trial. *Ann Intern Med*. 2009;151(5):306-314. doi:10.1097/OGX.0b013e3181e5a159.

54. Esposito K, Maiorino MI, Petrizzo M, Bellastella G, Giugliano D. The effects of a Mediterranean diet on the need for diabetes drugs and remission of newly diagnosed type 2 diabetes: Follow-up of a randomized trial. *Diabetes Care*. 2014;37(7):1824-1830. doi:10.2337/dc13-2899.

55. Elhayany A, Lustman A, Abel R, Attal-Singer J, Vinker S. A low carbohydrate Mediterranean diet improves cardiovascular risk factors and diabetes control among overweight patients with type 2 diabetes mellitus: A 1-year prospective randomized intervention study. *Diabetes, Obes Metab*. 2010;12(3):204-209. doi:10.1111/j.1463-1326.2009.01151.x.

56. Shai I, Schwarzfuchs D, Henkin Y, et al. Weight Loss with a Low-Carbohydrate, Mediterranean, or Low-Fat Diet. *N Engl J Med*. 2008;359(3):229-241. doi:10.1056/NEJMoa0708681.

57. Schwarzfuchs D, Golan R, Shai I. Four-Year Follow-up after Two-Year Dietary Interventions. *N Engl J Med*. 2012;367(14):1373-1374. doi:10.1056/NEJMc1204792.

58. Pan A, Sun Q, Bernstein AM, et al. Red meat consumption and risk of type 2 diabetes: 3 Cohorts of US adults and an updated meta-analysis. *Am J Clin Nutr*. 2011;94(4):1088-1096. doi:10.3945/ajcn.111.018978.

59. Hu EA, Pan A, Malik V, Sun Q. White rice consumption and risk of type 2 diabetes: meta-analysis and systematic review. *BMJ*. 2012;344:e1454. doi:10.1136/bmj.e1454.

60. Wang M, Yu M, Fang L, Hu R-Y. Association between sugar-sweetened beverages and type 2 diabetes: A meta-analysis. *J Diabetes Investig*. 2015;6(3):360-366. doi:10.1111/jdi.12309.

61. Imamura F, O'Connor L, Ye Z, et al. Consumption of sugar sweetened beverages, artificially sweetened beverages, and fruit juice and incidence of type 2 diabetes: systematic review, meta-analysis, and estimation of population attributable fraction. *BMJ*. 2015:h3576. doi:10.1136/bmj.h3576.

62. Carter P, Gray LJ, Troughton J, Khunti K, Davies MJ. Fruit and vegetable intake and incidence of type 2 diabetes mellitus: systematic review and meta-analysis. *BMJ*. 2010;341:c4229. doi:10.1136/bmj.c4229.

63. Cooper AJ, Forouhi NG, Ye Z, et al. Fruit and vegetable intake and type 2 diabetes: EPIC-InterAct prospective study and meta-analysis. *Eur J Clin Nutr*. 2012;66(10):1082-1092. doi:10.1038/ejcn.2012.85.

64. Chen M, Sun Q, Giovannucci E, et al. Dairy consumption and risk of type 2 diabetes: 3 cohorts of US adults and an updated meta-analysis. *BMC Med*. 2014;12(1):215. doi:10.1186/s12916-014-0215-1.

65. Aune D, Norat T, Romundstad P, Vatten LJ. Whole grain and refined grain consumption and the risk of type 2 diabetes: A systematic review and dose-response meta-analysis of cohort studies. *Eur J Epidemiol*. 2013;28(11):845-858. doi:10.1007/s10654-013-9852-5.

66. Ding M, Bhupathiraju SN, Chen M, van Dam RM, Hu FB. Caffeinated and decaffeinated coffee consumption and risk of type 2 diabetes: a systematic review and a dose-response meta-analysis. *Diabetes Care*. 2014;37(2):569-586. doi:10.2337/dc13-1203.

67. Li X-H, Yu F-F, Zhou Y-H, He J. Association between alcohol consumption and the risk of incident type 2 diabetes: a systematic review and dose-response meta-analysis. *Am J Clin Nutr*. 2016;103(3):818-829. doi:10.3945/ajcn.115.114389.

68. Muraki I, Imamura F, Manson JE, et al. Fruit consumption and risk of type 2 diabetes: results from three prospective longitudinal cohort studies. *BMJ*. 2013;347(aug28 1):f5001-f5001. doi:10.1136/bmj.f5001.

69. Upadhyaya S, Banerjee G. Type 2 diabetes and gut microbiome: At the intersection of known and unknown. *Gut Microbes*. 2015;6(2):85-92. doi:10.1080/19490976.2015.1024918.

70. David LA, Maurice CF, Carmody RN, et al. Diet rapidly and reproducibly alters the human gut microbiome. *Nature*. 2014;505(7484):559-563. doi:10.1038/nature12820.

71. Duncan SH, Belenguer A, Holtrop G, Johnstone AM, Flint HJ, Lobley GE. Reduced dietary intake of carbohydrates by obese subjects results in decreased concentrations of butyrate and butyrate-producing bacteria in feces. *Appl Environ Microbiol*. 2007;73(4):1073-1078. doi:10.1128/AEM.02340-06.

72. Dorans KS, Mostofsky E, Levitan EB, Håkansson N, Wolk A, Mittleman MA. Alcohol and Incident Heart Failure among Middle-Aged and Elderly Men: Cohort of Swedish Men. *Circ Hear Fail*. 2015;8(3):422-427. doi:10.1161/CIRCHEARTFAILURE.114.001787.

73. Steinhaus DA, Mostofsky E, Levitan EB, et al. Chocolate intake and incidence of heart failure: Findings from the Cohort of Swedish Men. *Am Heart J*. 2017;183:18-23. doi:10.1016/j.ahj.2016.10.002.

74. Dorans KS, Massa J, Chitnis T, Ascherio A, Munger KL. Physical activity and the incidence of multiple sclerosis. *Neurology*. 2016;87(17):1770-1776.

doi:10.1212/WNL.0000000000003260.
75. Mills KT, Chen J, Yang W, et al. Sodium Excretion and the Risk of Cardiovascular Disease in Patients With Chronic Kidney Disease. *JAMA*. 2016;315(20):2200. doi:10.1001/jama.2016.4447.
76. Chen J, Gu D, Huang J, et al. Metabolic syndrome and salt sensitivity of blood pressure in non-diabetic people in China: a dietary intervention study. *Lancet*. 2009;373(9666):829-835. doi:10.1016/S0140-6736(09)60144-6.
77. Li C, He J, Chen J, et al. Genome-Wide Gene-Sodium Interaction Analyses on Blood Pressure: The Genetic Epidemiology Network of Salt-Sensitivity Study. *Hypertension*. 2016;68(2):348-355. doi:10.1161/HYPERTENSIONAHA.115.06765.
78. He J, Streiffer RH, Muntner P, Krousel-Wood MA, Whelton PK. Effect of dietary fiber intake on blood pressure: a randomized, double-blind, placebo-controlled trial. *J Hypertens*. 2004;22(1):73-80. doi:10.1097/01.hjh.0000098164.70956.54.
79. He J, Ogden LG, Vupputuri S, Bazzano LA, Loria C, Whelton PK. Dietary sodium intake and subsequent risk of cardiovascular disease in overweight adults. *JAMA*. 1999;282(21):2027-2034. doi:10.1001/jama.282.21.2027.
80. He J, Wofford MR, Reynolds K, et al. Effect of dietary protein supplementation on blood pressure a randomized, controlled trial. *Circulation*. 2011;124(5):589-595. doi:10.1161/CIRCULATIONAHA.110.009159.
81. Thompson AM, Zhang Y, Tong W, et al. Association of inflammation and endothelial dysfunction with metabolic syndrome, prediabetes and diabetes in adults from Inner Mongolia, China. *BMC Endocr Disord*. 2011;11(1):16. doi:10.1186/1472-6823-11-16.
82. Irazola V, Rubinstein A, Bazzano L, et al. Prevalence, awareness, treatment and control of diabetes and impaired fasting glucose in the Southern Cone of Latin America. *PLoS One*. 2017;12(9). doi:10.1371/journal.pone.0183953.
83. Bazzano LA, Li TY, Joshipura KJ, Hu FB. Intake of fruit, vegetables, and fruit juices and risk of diabetes in women. *Diabetes Care*. 2008;31(7):1311-1317. doi:10.2337/dc08-0080.
84. Bazzano LA, Serdula M, Liu S. Prevention of Type 2 Diabetes by Diet and Lifestyle Modification. *J Am Coll Nutr*. 2005;24(5):310-319. doi:10.1080/07315724.2005.10719479.
85. Pollock BD, Chen W, Harville EW, et al. Differential sex effects of systolic blood pressure and low-density lipoprotein cholesterol on type 2 diabetes: Life course data from the Bogalusa Heart Study. *Journal of Diabetes*. 2017.
86. Zhang T, Li Y, Zhang H, et al. Insulin-sensitive adiposity is associated with a relatively lower risk of diabetes than insulin-resistant adiposity: the Bogalusa Heart Study. *Endocrine*. 2016;54(1):93-100. doi:10.1007/s12020-016-0948-z.
87. Pollock BD, Hu T, Chen W, et al. Utility of existing diabetes risk prediction tools for young black and white adults: Evidence from the Bogalusa Heart Study. *J Diabetes Complications*. 2016;31(1):86-93. doi:10.1016/j.jdiacomp.2016.07.025.
88. Hu T, Yao L, Reynolds K, et al. The effects of a low-carbohydrate diet vs. a low-fat diet on novel cardiovascular risk factors: A randomized controlled trial. *Nutrients*. 2015;7(9):7978-7994. doi:10.3390/nu7095377.
89. Hu T, Yao L, Reynolds K, et al. The effects of a low-carbohydrate diet on appetite: A randomized controlled trial. *Nutr Metab Cardiovasc Dis*. 2016;26(6):476-488. doi:10.1016/j.numecd.2015.11.011.

90. Rebholz CM, Reynolds K, Wofford MR, et al. Effect of soybean protein on novel cardiovascular disease risk factors: A randomized controlled trial. *Eur J Clin Nutr*. 2013;67(1):58-63. doi:10.1038/ejcn.2012.186.

91. Wofford MR, Rebholz CM, Reynolds K, et al. Effect of soy and milk protein supplementation on serum lipid levels: A randomized controlled trial. *Eur J Clin Nutr*. 2012;66(4):419-425. doi:10.1038/ejcn.2011.168.

92. Chen J, He J, Wildman RP, Reynolds K, Streiffer RH, Whelton PK. A randomized controlled trial of dietary fiber intake on serum lipids. *Eur J Clin Nutr*. 2006;60(1):62-68. doi:10.1038/sj.ejcn.1602268.

93. Overweight and Obesity Expert Panel. *Managing Overweight and Obesity in Adults: Systematic Evidence Review From the Obesity Expert Panel, 2013*.; 2013.

94. The Data Center. *Who Lives in New Orleans and Metro Parishes Now?*; 2017. https://www.datacenterresearch.org/data-resources/who-lives-in-new-orleans-now/.

95. Wetzels JFM, Kiemeney LALM, Swinkels DW, Willems HL, Heijer M Den. Age- and gender-specific reference values of estimated GFR in Caucasians: The Nijmegen Biomedical Study. *Kidney Int*. 2007;72(5):632-637. doi:10.1038/sj.ki.5002374.

96. Radin MS. Pitfalls in hemoglobin A1c measurement: When results may be misleading. *J Gen Intern Med*. 2014;29(2):388-394. doi:10.1007/s11606-013-2595-x.

97. Kim PS, Woods C, Georgoff P, et al. A1C underestimates glycemia in HIV infection. *Diabetes Care*. 2009;32(9):1591-1593. doi:10.2337/dc09-0177.

98. Qaseem A, Wilt TJ, Kansagara D, Horwitch C, Barry MJ, Forciea MA. Hemoglobin A1c Targets for Glycemic Control With Pharmacologic Therapy for Nonpregnant Adults With Type 2 Diabetes Mellitus: A Guidance Statement Update From the American College of Physicians. *Ann Intern Med*. 2018;168(8):569-576. doi:10.7326/M17-0939.

99. Sumithran P, Prendergast LA, Delbridge E, et al. Ketosis and appetite-mediating nutrients and hormones after weight loss. *Eur J Clin Nutr*. 2013;67(7):759-764. doi:10.1038/ejcn.2013.90.

100. Gardner CD, Trepanowski JF, Gobbo LCD, et al. Effect of low-fat VS low-carbohydrate diet on 12-month weight loss in overweight adults and the association with genotype pattern or insulin secretion the DIETFITS randomized clinical trial. *JAMA - J Am Med Assoc*. 2018;319(7):667-679. doi:10.1001/jama.2018.0245.

101. Brenner BM, Meyer TW, Hostetter TH. Dietary protein intake and the progressive nature of kidney disease: the role of hemodynamically mediated glomerular injury in the pathogenesis of progressive glomerular sclerosis in aging, renal ablation, and intrinsic renal disease. *N Engl J Med*. 1982;307(11):652-659. doi:10.1056/NEJM198209093071104.

102. Sato J, Kanazawa A, Makita S, et al. A randomized controlled trial of 130 g/day low-carbohydrate diet in type 2 diabetes with poor glycemic control. *Clin Nutr*. 2017;36(4):992-1000. doi:10.1016/j.clnu.2016.07.003.

103. Yancy WS, Foy M, Chalecki AM, et al. A low-carbohydrate, ketogenic diet to treat type 2 diabetes. *Nutr Metab (Lond)*. 2005;2(1):34. doi:10.1186/1743-7075-2-34.

104. Boden G, Sargrad K, Homko C, Mozzoli M, Stein TP. Effect of a low-carbohydrate diet on appetite, blood glucose levels, and insulin resistance in obese patients with type 2 diabetes. *Ann Intern Med*. 2005;142(6). doi:10.1016/S0084-3741(08)70336-6.

105. Stern L, Iqbal N, Seshadri P. The Effects of Low-Carbohydrate versus Conventional Weight Loss Diets in Severely Obese Adults : One-Year Follow-up of a randomized control trial. *Ann Intern …*. 2004;140(May 2001):769-777.

doi:10.1016/j.accreview.2004.07.103.

106. Westman EC, Yancy WS, Edman JS, Tomlin KF, Perkins CE. Effect of 6-month adherence to a very low carbohydrate diet program. *Am J Med*. 2002;113(1):30-36. doi:10.1016/S0002-9343(02)01129-4.

107. Moosheer SM, Waldschütz W, Itariu BK, Brath H, Stulnig TM. A protein-enriched low glycemic index diet with omega-3 polyunsaturated fatty acid supplementation exerts beneficial effects on metabolic control in type 2 diabetes. *Prim Care Diabetes*. 2014;8(4):308-314. doi:10.1016/j.pcd.2014.02.004.

108. Aagaard K, Petrosino J, Keitel W, et al. The Human Microbiome Project strategy for comprehensive sampling of the human microbiome and why it matters. *FASEB J*. 2013;27(3):1012-1022. doi:10.1096/fj.12-220806.

109. McInnes P, Cutting M. Manual of Procedures for Human Microbiome Project. http://www.hmpdacc.org/doc/HMP_MOP_Version12_0_072910.pdf.

110. Marrero DG, Palmer KNB, Phillips EO, Miller-Kovach K, Foster GD, Saha CK. Comparison of commercial and self-initiated weight loss programs in people with prediabetes: A randomized control trial. *Am J Public Health*. 2016;106(5):949-956. doi:10.2105/AJPH.2015.303035.

111. Barengolts E, Manickam B, Eisenberg Y, Akbar A, Kukreja S, Ciubotaru I. EFFECT OF HIGH-DOSE VITAMIN D REPLETION ON GLYCEMIC CONTROL IN AFRICAN-AMERICAN MALES WITH PREDIABETES AND HYPOVITAMINOSIS D. *Endocr Pract*. 2015. doi:10.4158/EP14548.OR.

112. Block G, Azar KMJ, Romanelli RJ, et al. Diabetes prevention and weight loss with a fully automated behavioral intervention by email, web, and mobile phone: A randomized controlled trial among persons with prediabetes. *J Med Internet Res*. 2015. doi:10.2196/jmir.4897.

113. Kramer MK, Molenaar DM, Arena VC, et al. Improving employee health: Evaluation of a worksite lifestyle change program to decrease risk factors for diabetes and cardiovascular disease. *J Occup Environ Med*. 2015. doi:10.1097/JOM.0000000000000350.

114. Parker AR, Byham-Gray L, Denmark R, Winkle PJ. The Effect of Medical Nutrition Therapy by a Registered Dietitian Nutritionist in Patients with Prediabetes Participating in a Randomized Controlled Clinical Research Trial. *J Acad Nutr Diet*. 2014;114(11):1739-1748. doi:10.1016/j.jand.2014.07.020.

115. Maruthur NM, Ma Y, Delahanty LM, et al. Early response to preventive strategies in the diabetes prevention program. *J Gen Intern Med*. 2013;28(12):1629-1636. doi:10.1007/s11606-013-2548-4.

116. Leong A, Daya N, Porneala B, et al. Prediction of Type 2 Diabetes by Hemoglobin A1c in Two Community-Based Cohorts. *Diabetes Care*. 2017;41(1):dc170607. doi:10.2337/dc17-0607.

117. Khaw K-T, Wareham N, Bingham S, Luben R, Welch A, Day N. Association of hemoglobin A1c with cardiovascular disease and mortality in adults: the European prospective investigation into cancer in Norfolk. *Ann Intern Med*. 2004;141(6):413-420. doi:10.7326/0003-4819-141-6-200409210-00006.

118. Cole RE, Boyer KM, Spanbauer SM, Sprague D, Bingham M. Effectiveness of Prediabetes Nutrition Shared Medical Appointments. *Diabetes Educ*. 2013;39(3):344-353. doi:10.1177/0145721713484812.

Low-Carbohydrate Dietary Pattern on Glycemic Outcomes Trial

PROTOCOL

PROTOCOL VERSION 1.6

Dated: August 20, 2019

New Orleans, LA

Table of Contents

1. Overview 5
2. Background and Significance 6
2.A. Type 2 Diabetes Mellitus is a major global public health challenge 6
2.B. Prediabetes is a major risk factor for cardiometabolic diseases 6
2.C. Role of lifestyle in preventing diabetes 6
2.D. Low-to-moderate carbohydrate diets, weight loss, and cardiometabolic diseases 7
2.E. Mediterranean-style diet is associated with reduced risk of T2D and improved glycemic control 8
2.F. Evidence for low-carbohydrate diets that emphasize Mediterranean-style dietary components 8
2.G. Evidence for specific food groups and risk of T2D 8
2.H. Gut microbiota, diet and T2D 9
2.I. Clinical and public health significance of the proposed study 9
2.J. Innovation 9
3. Preliminary Studies 10
3.A. Summary of aggregate experience of the research team 10
3.B. Low-carbohydrate behavioral dietary intervention trial experience 10
3.C. Other dietary intervention trial experience 11
4. Study Design and Organization 11
4.A. Overview of trial design 11
4.B. Study outcomes 12
4.B.1. Primary outcome 12
4.B.2. Secondary outcomes 12
4.C Study Timeline 12
6. Study Participants 13
6.A. Eligibility criteria 13
7. Recruitment and Randomization 13
7.A. Recruitment 13
7.A.1. Mass mailing, placement of brochures/flyers in community, TV/radio/billboard advertisements 14
7.A.2. Database searches and targeted mailing 14
7.A.3. Referrals and Local Partnerships 14
7.B. Randomization 14
7.C. Masking 15

7.D. Safety Considerations 15
8. Dietary Intervention Program 15
8.A. Low-carbohydrate dietary intervention group 15
8.B. Usual Diet Group 16
8.C. Physical Activity 16
8.D. Dietary Adherence 17
9. Safety of Dietary Intervention 17
10. Data Collection 18
10.A. Study visits 18
10.A.1. Screening/baseline visits 18
10.A.2. Follow-up visits and final visit 19
10.B Study measurements 19
10.B.1. Questionnaires 19
10.B.2. Dietary measurements 20
10.B.3. Assessment and management of adverse effects 20
10.B.4. BP and anthropometric measures 20
10.B.5. Blood samples 20
10.B.6. Urine collection 21
10.B.7. Stool Specimen Collection 21
11. Quality Assurance and Quality Control 21
11.A. Manual of procedures 21
11.B. Training and certification 21
12. Sample Size and Statistical Power 22
13. Data Management and Analysis 23
13.A. Data management 23
13.B. Data analysis plan 23
13.B.1. Exploratory data analysis 23
13.B.2. Primary, secondary, and exploratory outcomes 23
15. Anticipated Challenges 24
16. Protection of Human Subjects 24
16.A. Protection of human subjects from research risks 24
16.A.1. Risks to the subjects 24
16.A.2. Adequacy of protection against risks 26

16.A.3. Potential benefits of the proposed research to human subjects and others... 27
16.A.4. Importance of knowledge to be gained ... 27
16.A.5. Remuneration ... 27
16.B. Data safety and monitoring plan ... 27
Bibliography and References Cited ... 30

1. Overview

The increasing prevalence of type 2 diabetes mellitus (T2D), a major cause of morbidity and mortality worldwide, is a critical public health concern. In the United States, an estimated 33.9% of adults have prediabetes (impaired fasting glucose, elevated hemoglobin A1c (HbA1c), or impaired glucose tolerance). Individuals with prediabetes are at elevated risk of T2D and cardiovascular disease (CVD), with 5–10% developing clinical T2D each year.

In the short-term, among patients with T2D, low-to-moderate carbohydrate diets (energy percentage below 45) have a greater glucose-lowering effect than do high-carbohydrate diets, with larger glucose-lowering effect the greater the carbohydrate reduction. Additionally, compared with low-fat diets, Mediterranean diets that are high in unsaturated fats (particularly *cis*-monounsaturated fats) reduce risk of T2D and CVD and improve glycemic control among diabetics. However, compared with usual diets, the effect of a behavioral intervention promoting a low-carbohydrate/high-unsaturated fat and protein dietary pattern that emphasizes consumption of non-starchy vegetables, plant-based oils, fish, poultry, and mixed nuts on glucose homeostasis, and other metabolic risk factors among individuals with prediabetes or early-stage untreated T2D is not well understood.

Our *long-term goal* is to develop and implement dietary intervention(s) that reduce risk of T2D. The *overall goal of this randomized controlled trial is to study the effect of a behavioral intervention promoting a low-carbohydrate/high-unsaturated fat and high-protein dietary pattern (initial target <40 g digestible carbohydrates, final target <60 g digestible carbohydrates) compared with a usual diet on metabolic risk factors among individuals with prediabetes or early-stage untreated diabetes* (HbA1c 6.0–6.9%). Specifically, we will test the following hypotheses:

Primary hypothesis: Compared with usual diet, over a 6-month period, a healthy low-carbohydrate/high unsaturated fat and high-protein dietary pattern will lead to *a larger decrease of HbA1c, by at least 0.15%.*

Secondary hypothesis: Over a 6-month period, a healthy low-carbohydrate/high-unsaturated fat and high-protein dietary pattern will lead to *larger improvements in the following major secondary outcomes*: fasting glucose, systolic blood pressure (BP), total-to-HDL-cholesterol ratio, and body weight.

Exploratory outcomes: We will also study the effect of the intervention on the following outcomes: insulin and homeostasis model assessment of insulin resistance (HOMA-IR); diastolic BP; waist circumference; and estimated CVD risk.

To achieve these study aims, we will recruit 112 adults with prediabetes or untreated diabetes (HbA1c 6.0–6.9%) in the Greater New Orleans, LA area. Using a stratified, blocked randomization scheme, participants will be assigned to either a behavioral modification intervention aimed at promoting a healthy low-carbohydrate dietary pattern or to usual diet. The intervention group will have an intensive phase for 3 months, followed by a maintenance phase in months 4 through 6. The *primary outcome will be difference between the active intervention and control groups in the change in HbA1c from baseline to 6 months of follow-up. We expect 85% power to detect a difference of at least 0.15% in change of HbA1c.* Changes in other metabolic risk factors will be studied concurrently. Metabolic risk factors will be assessed at baseline, 3 months, and 6 months of follow-up.

There is much interest regarding dietary approaches to prevent T2D and its complications. The results from this study will help to determine whether, compared with usual diet, a behavioral intervention aimed at promoting a healthy low-carbohydrate dietary pattern improves metabolic risk factors.

2. Background and Significance

2.A. Type 2 Diabetes Mellitus is a major global public health challenge

Diabetes is a strong and independent risk factor for CVD morbidity and mortality. A meta-analysis of 102 prospective studies (698,782 participants) estimated diabetes leads to an approximately 2-fold increased risk in vascular diseases, including coronary heart disease (CHD) and major stroke subtypes, and deaths due to other vascular causes.[1] Among participants with diabetes, CHD risk is about 50% higher among those with fasting blood glucose ≥7 mmol/L compared with those with fasting blood glucose <7 mmol/L.[1] In addition to increasing CVD risk, diabetes can cause diabetic retinopathy,[2] chronic kidney disease,[3] lower limb amputation,[4] and other complications. Most individuals with diabetes have T2D.[5] The US Centers for Disease Control and Prevention (CDC) estimated that 30.2 million adults ≥18 years (12.2%) had diabetes in 2015, with 7.2 million (23.8%) not being aware of or reporting diabetes.[5] Diagnosed diabetes prevalence among US adults has increased substantially over time, from 2.54% in 1980 to 7.40% in 2015.[6] Diabetes prevalence is also high globally, with an estimated 2014 age-standardized prevalence of 9.0% in men and 7.9% in women.[7] Diabetes accounts for an increasing portion of US healthcare expenditures. Total cost of diagnosed diabetes in the US was estimated to be $245 billion in 2012, 41% higher than in 2007.[8] Diabetes is associated with much higher lifetime medical expenses, with higher expenses for those diagnosed at younger ages.[9]

2.B. Prediabetes is a major risk factor for cardiometabolic diseases

Prediabetes, defined by the American Diabetes Association (ADA) as impaired fasting glucose (IFG), impaired glucose tolerance (IGT), or elevated HbA1c, is also referred to as "intermediate hyperglycemia"[10] and "high-risk state of developing diabetes".[11] The ADA defines prediabetes as having: fasting plasma glucose of 100–125 mg/dL (IFG); 2-hour post-load glucose in the 75-g oral glucose tolerance test (OGTT) of 140–199 mg/dL (IGT); or HbA1c 5.7–6.4%.[12] Prediabetes itself is not a disease; for all three tests, "risk is continuous, extending below the lower limit of the range and becoming disproportionately greater at the higher end of the range".[12] The CDC estimated that in 2015, 33.9% of US adults had prediabetes based on fasting glucose or HbA1c.[5] Among those with prediabetes, only 11.9% reported having being told this by a medical professional.[5] Approximately 5–10% of individuals with prediabetes develop diabetes within one year, depending on how prediabetes is defined.[13] In a meta-analysis, annual diabetes incidence was 15–19% among individuals with both IFG and IGT, while it was 4–6% in those with isolated IFG and 6–9% in those with isolated IGT.[13]

A recent meta-analysis (1,611,339 participants from 53 studies) reported that prediabetes as defined by the ADA is associated with higher risk of composite CVD events and CHD.[14] IFG and IGT were also positively associated with increased risk of stroke and all-cause mortality.[14] Prediabetes is linked with increased risk of other conditions typically thought of as diabetes complications, such as neuropathies and nephropathies.[15] Importantly, it is possible for individuals with prediabetes to revert to normoglycemia, as observed in observational studies[13] and lifestyle or drug intervention trials.[16,17] In the Diabetes Prevention Program (DPP), reversion to normoglycemia was associated with lower long-term T2D risk[18] and lower predicted CVD risk.[19]

2.C. Role of lifestyle in preventing diabetes

Much evidence from observational studies and clinical trials supports the critical role of diet and other lifestyle factors in preventing T2D. Globally, lifestyle transitions towards reduced physical activity, increased consumption of refined carbohydrates, coupled with resulting changes in body composition, has led to increases in diabetes prevalence and severity.[20] Body

mass index (BMI), waist circumference, and waist-to-height ratio are predictors of T2D.[21] The DPP, which combined calorie restriction and exercise to promote weight loss, substantially reduced risk of T2D (by 58%) among individuals with elevated FPG and IGT;[22] a benefit that persisted in 10-year follow-up analyses.[16] Other trials of lifestyle modification focused on diet and exercise have also shown benefits in study populations in China,[23] Finland,[24] and India.[25]

2.D. Low-to-moderate carbohydrate diets, weight loss, and cardiometabolic diseases

Low-to-moderate carbohydrate diets (<45% of energy from carbohydrates) are popular dietary approaches for losing weight. Weight loss is a key component of the DPP lifestyle intervention[26] and ADA lifestyle recommendations.[12,27] Meta-analyses from our group[28] and others support that low-to-moderate carbohydrate diets are at least as effective as low-fat diets at promoting weight loss. For instance, a meta-analysis reported a 2-kg (95% CI, 3.1–0.9) larger weight loss in low-carbohydrate diet (<120 g carbohydrates/day) arms compared with low-fat diet (<30% energy from fat) arms.[29] Estimated 10-year CVD risk was lower in low-carbohydrate than low-fat arms.[29] This aligns with research published by our team; in a 12-month trial of participants with obesity, a low-carbohydrate diet (target <40 g carbohydrates/day) was more effective than a low-fat diet (target <30% energy from fat, <7% saturated fat) for weight loss and CVD risk factor reduction.[30]

Low-to-moderate carbohydrate diets may also be beneficial among patients with T2D. Low-carbohydrate diets have come in and out of favor as treatment of diabetes.[31] In a meta-analysis (1376 patients;10 trials), after 3 or 6 months of the intervention, low-to-moderate carbohydrate diets (<45% energy from carbohydrates) led to a 0.34% lower HbA1c (95% CI, 0.06–0.63%) compared with high-carbohydrate diets.[32] Compared with high-carbohydrate diets, there was a greater reduction in HbA1c the greater the reported carbohydrate restriction.[32] There was no benefit of the low-to-moderate carbohydrate diets on HbA1c at 12 months or longer, and no difference between interventions on BMI, body weight, low-density lipoprotein (LDL) cholesterol or quality of life.[32] However, results may have been affected by dietary adherence or glucose medication. There was evidence of diabetes medication reduction in the low-carbohydrate arms in some studies,[33–37] with evidence of medication reduction and improved blood glucose stability persisting for up to 2 years.[38] Hypoglycemia (though not after medication adjustment) was observed in three subjects taking insulin or a sulfonylurea out of twelve subjects in the low-carbohydrate arm of another study.[39] In a subset of participants with T2D (n=46) from a large weight loss trial, compared with those randomized to a low-fat diet and orlistat, those randomized to a low-carbohydrate diet for 48 weeks had improved HbA1c (0.8% lower, 95% CI, -1.5, -0.02%) and a reduction in antiglycemic medications, despite similar weight loss effects of the interventions.[40]

Though low-to-moderate carbohydrate diets have been studied among overweight and obese participants (primarily focused on weight loss), less is understood about the effects of such diets on glycemic outcomes specifically among individuals with prediabetes. A study among participants with IGT observed a reduction at 12 months in weight, HbA1c, and other glycemic outcomes among individuals who participated in a 7-day in-hospital education program, though this was not randomized.[41] A randomized pilot trial of overweight or obese individuals with T2D or prediabetes found that, over 3 months and at 12 months, a very low-carbohydrate diet improved HbA1c, led to higher medication discontinuation, and more weight loss compared with a low-fat diet.[34,37] However, only 4 individuals had prediabetes. Given benefits of low-to-moderate carbohydrate diets for weight loss in general populations and for glycemic control among patients with T2D, further study of these diets are warranted among individuals with prediabetes who are at elevated cardiometabolic disease risk.

2.E. Mediterranean-style diet is associated with reduced risk of T2D and improved glycemic control

Though there is no one "Mediterranean diet", the Mediterranean dietary pattern tends to have the following characteristics: high consumption of vegetables, fruits, breads and cereals, legumes, nuts and seeds; low-to-moderate consumption of dairy products, fish, poultry and eggs; little consumption of red meat and sweets; and red wine in moderation.[42] Olive oil is an important source of fat. In many observational studies, adherence to the Mediterranean-style dietary pattern is associated with reduced risk of CVD[43] and T2D.[44,45] Evidence from randomized trials also supports the benefits of this dietary pattern. In several trials, participants randomized to Mediterranean diets had substantially lower risk of CVD than those randomized to low-fat control diets.[46,47] In the three-arm randomized PREDIMED trial of participants at high risk of CVD, after 4.1 years of follow-up, those randomized to a traditional Mediterranean diet supplemented with either olive oil or nuts had a 30% reduced risk of T2D compared to those in the low-fat control diet group.[48] In all PREDIMED arms, average carbohydrate intake was relatively low (39.7–43.7% at end of trial).[47] Two trials have found a higher rate of remission from metabolic syndrome among individuals randomized to a Mediterranean diet compared with those randomized to control diets (prudent or low-fat) during 2–5 years of follow-up.[49,50] The Mediterranean pattern may also be beneficial among individuals with T2D. Meta-analyses have reported that, among patients with T2D, those randomized to a Mediterranean diet had lower HbA1c levels and CVD risk factors, and more significant weight loss, compared with those randomized to control diets.[51]

2.F. Evidence for low-carbohydrate diets that emphasize Mediterranean-style dietary components

Many low-carbohydrate diets involve increased consumption of food items, such as red meat,[52] that are associated with increased cardiometabolic disease risk. However, low-carbohydrate dietary approaches can focus on substituting carbohydrates with foods common in Mediterranean-style diets, such as olive oil and nuts, which may improve cardiometabolic health. In a 4-year Italian study of individuals with newly diagnosed T2D, those randomized to a low-carbohydrate Mediterranean diet (<50% carbohydrates; consumed ~44%) had more favorable changes in glycemic control, higher rates of T2D remission, and delayed need for antiglycemic drugs compared with participants randomized to a low-fat diet (<30% fat, <10% saturated fat).[53,54] A 12-month Israeli study assessed low-carbohydrate Mediterranean (35% carbohydrates), traditional Mediterranean, and ADA (both 50–55% carbohydrates) diets in 259 overweight participants with T2D.[55] Most CVD risk factors improved in all groups; there was only HDL improvement in the low-carbohydrate Mediterranean diet and this diet was superior to the other diets in improving glycemic control.[55] In a 2-year weight-loss study of moderately obese participants, low-carbohydrate and Mediterranean diets were superior to low-fat diet for weight loss.[56] Participants in the low-carbohydrate arm were counseled to choose vegetarian sources of fat and protein and to avoid trans fat. At 6 years after study commencement, despite weight regain, there were still long-term benefits, particularly for the low-carbohydrate and Mediterranean diets.[57] In whole, evidence supports that low-carbohydrate Mediterranean-style diets are feasible and may have metabolic benefits.

2.G. Evidence for specific food groups and risk of T2D

Prospective cohort studies have found consumption of specific foods and beverages is associated with diabetes risk. Meta-analyses report higher consumption of processed and unprocessed red meat,[58] white rice (particularly in Asian populations),[59] and sugar-sweetened beverages[60,61] are associated with higher T2D risk. Consumption of green leafy vegetables,[62,63] yogurt,[64] whole grains,[65] decaffeinated and total coffee,[66] and light-to-moderate alcohol

consumption[67] are associated with reduced risk. Consumption of fruits, particularly blueberries, grapes, and apples, are associated with reduced risk; fruit juice is associated with higher risk.[68]

2.H. Gut microbiota, diet and T2D

There is increasing evidence for the importance of gut microbiota in risk of type 2 diabetes and other cardiometabolic diseases.[69] Additionally, dietary modifications, including changes in carbohydrate consumption, can lead to substantial changes in gut microbiome over short periods of time.[70,71] Less is known about longer-term changes to the gut microbiota during the adoption of a low-carbohydrate diet and how these changes may influence cardiometabolic risk. We plan to collect stool specimens at baseline, 3 months, and at 6 months, for potential future studies on how this intervention affects the gut microbiome.

2.I. Clinical and public health significance of the proposed study

Prediabetes, or intermediate hyperglycemia, is a marker of high risk of developing T2D and other complications. Due to increasing prevalence of T2D and associated morbidity and mortality globally, a better understanding of dietary approaches to delay progression to T2D or to promote reversion to normoglycemia is a high public health priority. This study proposes to assess the effect of a behavioral intervention promoting a low-carbohydrate/high-unsaturated fat dietary pattern (initial target <40 g digestible carbohydrates, final target <60 g digestible carbohydrates) on HbA1c, a marker of glycemic control, and other metabolic risk factors. Building from prior findings from clinical and observational studies, we will develop a dietary intervention that promotes the consumption of non-starchy vegetables, plant-based oils, fish, poultry, and mixed nuts, that are thought to reduced risk of T2D or CVD. Participants assigned to the intervention will be trained in behavioral techniques that will provide capacity to maintain dietary changes long after study completion. Our outcome measures will assess the effect of this dietary intervention in a “real-life” setting as opposed to dietary modifications achieved in metabolic ward studies or through food prepared in research kitchens. Findings from this study may provide evidence in support of promoting alternative dietary styles among individuals with high T2D and CVD risk and could potentially be used to inform dietary interventions used in programs like DPP.

2.J. Innovation

The status quo as it pertains to dietary approaches for reducing risk of T2D is focused on reducing caloric intake and total fat intake. However, recent evidence suggests that the types of fat, rather than the quantity of fats may be more important, with evidence supporting that a Mediterranean-style diet that does not restrict fat intake reduces risk of developing both T2D[48] and CVD[47]. Moreover, in the short-term, low-carbohydrate diets, which are popular weight loss diets, have a greater glucose-lowering effect than do high-carbohydrate diets among patients with T2D.[32] However, the effect of low-carbohydrate diets that emphasize consumption of non-starchy vegetables, plant-based oils, fish, poultry, and mixed nuts on T2D prevention is unclear, particularly compared with higher-carbohydrate diets more commonly consumed in the US. *The proposed research is innovative, in our opinion, because it represents a substantial departure from the status quo by assessing the combined effect of a low-carbohydrate, high unsaturated fat and protein diet on cardiometabolic risk factors among adults with prediabetes or early-stage untreated diabetes.* We expect the results from this study have the potential to lead to new horizons for developing and implementing dietary approaches (other than the most commonly used reduced fat diet) that will substantially reduce risk of cardiometabolic disease among adults with T2D or at high risk of T2D. The proposed intervention dietary approach promotes consumption of dietary components thought to reduce risk of cardiometabolic disease and the behavior change intervention has expected applicability in clinical practice.

3. Preliminary Studies

3.A. Summary of aggregate experience of the research team

Dr. Kirsten Dorans is trained in cardiovascular disease epidemiology, with strong training in epidemiologic methods and biostatistics. Her publication record includes studies of behavioral risk factors and risk of chronic disease. She has published on the relationships between dietary intake and chronic heart failure[72,73] and on physical activity and incident multiple sclerosis[74]. She served as lead author, conducting data management, analysis, drafting, and revision of two of these manuscripts. In graduate school, she focused on studying links between outdoor air pollution and chronic diseases and has published several papers in this field. The COBRE program will provide an excellent opportunity for the JFI to transition from air pollution research to clinical and translational research focused on nutrition and diabetes. Building off of her training in observational epidemiology, this program will involve training and practical experience that will allow her to broaden her expertise and to develop practical skills for carrying out clinical research.

The mentorship team has much experience and prior successful collaborations in key areas related to this study. Dr. Jiang He is an internationally renowned expert in cardiometabolic disease clinical and epidemiological research. He has served as PI for many epidemiological studies and clinical trials, including clinical and observational studies focused on dietary consumption and chronic diseases[75–80] and has conducted extensive global research on diabetes.[81,82] Dr. Bazzano has collaborated extensively with Dr. He for more than 15 years in research related to diet and chronic disease. She is a clinical investigator and has expertise in nutritional epidemiology and clinical research. Dr. Bazzano was the PI of a highly successful clinical trial comparing the effects of low-carbohydrate and low-fat diets on weight and cardiovascular risk factors among individuals with obesity, which was a subproject within the Tulane COBRE in Hypertension and Renal Biology.[30] Dr. He served as Dr. Bazzano's mentor for this clinical trial. They have collaborated on much other work related to dietary and chronic disease, including a meta-analysis on low-carbohydrate diets.[28] Dr. Bazzano has also published many articles on diet and risk of diabetes[83,84] and on diabetes in the Bogalusa Heart Study.[85–87]

3.B. Low-carbohydrate behavioral dietary intervention trial experience

In the study on macronutrient composition of diet and risk factors for cardiovascular disease (MACRO) study, 148 individuals without clinical cardiovascular disease or diabetes and with obesity were randomly assigned to a low-carbohydrate or a low-fat behavioral dietary intervention for 12 months. In the low-carbohydrate arm, daily dietary intake of carbohydrates decreased from an average of 242 g at baseline to 97 g, 93 g, and 127 g at 3, 6, and 12 months, respectively. At 12 months, participants in the low-carbohydrate intervention group had greater decreases in weight (-3.5 kg; 95% CI, -5.6 to -1.4 kg) and fat mass (-1.5%; 95% CI, -2.6% to -0.4%) and greater improvement in several cardiovascular risk factors.[30] This suggests that restricting carbohydrates may be an option for weight loss and reduction of cardiovascular risk factors. Further analyses found that the low-carbohydrate dietary intervention led to similar or greater improvement in inflammation, adipocyte dysfunction, and endothelial dysfunction compared with the low-fat dietary intervention[88] and that the low-fat diet reduced peptide YY more than the low-carbohydrate diet, suggesting satiety may be better preserved on low-carbohydrate diets.[89] No serious adverse events were reported in this study and the number of participants with symptoms were similar between the two diets, with the exception of more participants having headaches on the low-fat diet at 3 months (18 vs 6 participants, $p = 0.03$).[30] Dr. Bazzano served as PI for this study and Dr. He served as her mentor. Dr. Bazzano

mentored one of her PhD students in conducting analyses and writing and editing manuscripts related to this project.

3.C. Other dietary intervention trial experience

Dr. He has also led or been involved with many dietary intervention trials, including both behavioral change and feeding study interventions. In the Protein and Blood Pressure (ProBP) study, 352 adults with prehypertension or stage 1 hypertension in New Orleans, LA and Jackson, MS were assigned to take, in a random order, 40 g/d soy protein, milk protein, or carbohydrate supplementation each for 8 weeks. Compared with the high-glycemic index refined carbohydrate, both soy protein and milk protein supplementation were associated with a -2.0 mm Hg (95% CI -3.2 to -0.7 mm Hg) and -2.3 mm Hg (-3.7 to -1.0 mm Hg) net change in systolic blood pressure.[80] These findings suggest that partial replacement of carbohydrate with soy or milk protein might be an important part of nutrition intervention strategies to prevent and treat hypertension. Additionally, soy protein supplementation led to reduction in plasma E-selectin compared with carbohydrate and milk protein and in plasma leptin compared with carbohydrate.[90] Soy protein also led to improvement in lipid profile compared with carbohydrate and milk protein.[91] Dr. He was PI for this project.

In the Fiber Intervention on Blood Pressure (FIBRE) study, 110 participants ages 30–65 years with untreated pre-hypertension or stage-1 hypertension and serum cholesterol level < 240 mg/dl were randomly assigned to 8-g/day of water soluble fiber from oat bran or a control intervention (double-blind RCT). Over the 12-week study period, net changes in systolic and diastolic blood pressure were -1.8 mm Hg (-4.3 to 0.8) and -1.2 mm Hg (-3.0 to 0.5 mm Hg), respectively.[78] Net changes in total, HDL-, and LDL-cholesterol were -2.40 mg/dL (-10.60 to 5.81 mg/d:), -1.66 mg/dL (-4.55 to 1.22 mg/dL), and -1.33 mg/dl (-8.33 to 5.68 mg/dL), respectively.[92] Dr He was PI for this study.

Dr. He is currently serving as a mentor for Dr. Katherine Mills' COBRE project (Effect of Dietary Sodium Reduction in Kidney Disease Patients with Albuminuria; SUPER). In this project, participants with chronic kidney disease and albuminuria are being randomized to a behavioral modification program designed to reduce dietary sodium intake to ≤2,300 mg/day or to usual care and test the effect of this intervention on change in albumin-to-creatinine ratio from baseline to 24-weeks.

4. Study Design and Organization

4.A. Overview of trial design

We propose to conduct a *randomized controlled trial* aimed at testing the effect of a behavioral intervention that promotes *reduced carbohydrate intake and increased consumption of heart-healthy unsaturated fats and protein* in patients with HbA1c 6.0–6.9% (*Figure 1*). We plan to recruit 112 individuals with elevated HbA1c and randomly assign them to a moderately intensive intervention on diet pattern,[93] using well-established methods for behavioral modification, or to usual diet. We will compare the difference between the active intervention and control groups for change in HbA1c from baseline to 6 months of follow-up (primary outcome). We will assess four major secondary outcomes: fasting plasma glucose, systolic BP, total-to-HDL cholesterol ratio, and body weight. In

Figure 1. Study Design

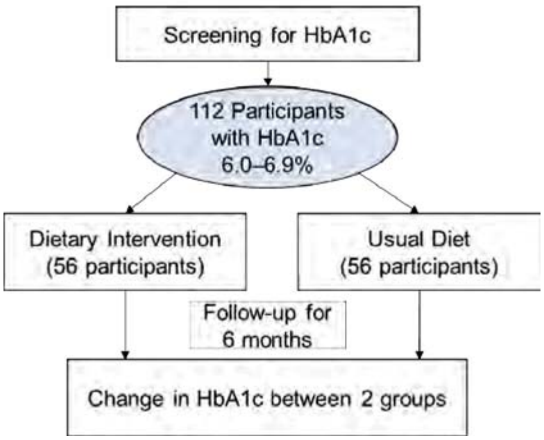

addition, we will look at several other exploratory outcomes: insulin and HOMA-IR, diastolic BP, waist circumference, and estimated CVD risk, as estimated by the 2013 ACC/AHA risk equation. The main trial components are summarized in *Table 1*.

Table 1. Study Overview

Study Design	Randomized controlled trial
Study Participants	112 participants with elevated HbA1c (6.0–6.9%)
Intervention Group (n=56)	Behavioral modification to reduce carbohydrate consumption and promote consumption of foods such as non-starchy vegetables, plant-based unsaturated oils, nuts and seeds, avocados, eggs, seafood, poultry, lean meat and tofu. We will recommend moderate consumption of cheese, unsweetened Greek yogurt, and low-carb milk. If consuming higher-carbohydrate items, recommend legumes, fruits, (limited) whole grains. Target <40 g net carbohydrates per day for first 3 months and will have a goal of <40 net g carbohydrates per day; <60 g net carbohydrates per day for months 4 onwards.
Usual Diet Group (n=56)	No dietary intervention. To encourage continued participation in the trial, we will offer optional monthly educational sessions on unrelated topics, such as retirement planning or health insurance
Outcome Measures	Primary: HbA1c; Secondary: fasting plasma glucose, systolic BP, total-to-HDL-cholesterol ratio, and body weight

4.B. Study outcomes

4.B.1. Primary outcome: The ***primary outcome will be the difference between the intervention group and the usual diet group for change in HbA1c*** from baseline to 6 months. Changes in HbA1c will be calculated from blood samples taken at baseline and 6 months.
4.B.2. Secondary outcomes: Secondary outcomes will include changes from baseline to 6 months of *fasting plasma glucose, systolic BP, total-to-HDL cholesterol ratio, and body weight.,*
4.B.3. Other outcomes: We will also study the effect of the intervention on the following outcomes: insulin and homeostasis model assessment of insulin resistance (HOMA-IR); diastolic BP; waist circumference; total-to-HDL-cholesterol ratio; and estimated CVD risk.

4.C Study Timeline

Table 2 shows the timeline, which will be followed in the implementation of study-related activities, such as publications and submission of an R01 grant application.

Table 2. Study Timeline

	Year 1		Year 2		Year 3	
Develop MOP, intervention program, study forms, and pilot testing	X	X				
Certification and training of staff	X					
Recruitment		X	X			
Intervention and data collection, measurement, entry, cleaning		X	X	X	X	
Data quality control and analysis						X
Publications	X	X	X	X	X	X
R01 grant submissions, resubmissions				X	X	X

The first 6 months will focus on development of manual of operations (MOP), intervention program, study forms, study protocol, Institutional Review Board (IRB) approval, and pilot

testing of the intervention program. It will also involve staff training and certification. During the last half of year 1, recruitment, intervention, and data collection will commence. During the last 3 months of year 3, data quality control and analysis will be carried out. Throughout the study period, the JFI will publish peer-reviewed manuscripts from ongoing Tulane University research projects.

6. Study Participants

6.A. Eligibility criteria

Inclusion criteria

- Men or women ages 40 to 70 years: aim to recruit 50% women
- Any race or ethnicity: aim to recruit 40% ethnic minorities (i.e., African American and Hispanic), close to population in the greater New Orleans area[94]
- HbA1c 6.0–6.9%
- Willing and able to provide informed consent

Exclusion criteria

- Diagnosed type 1 diabetes mellitus based on patient self-report
- Use of agents affecting glycemic control (medications for diabetes, oral glucocorticoids) within the past three months prior to enrollment based on patient self-report
- Medical condition in which low-carbohydrate diet may not be advised
    - Estimated glomerular filtration rate (eGFR) ≤45 mL/min/1.73 m^2, which is close to the 5th percentile of eGFR among non-diseased individuals of 70 years of age[95]
    - Self-report of liver disease due to hepatitis or alcohol; osteoporosis; untreated thyroid disease; gout; or cancer (other than non-melanoma skin cancer) requiring treatment in the past year, unless prognosis is excellent
- Factors that may affect HbA1c:
    - Hemoglobin <11 mg/dL (cutpoint for moderate-to-severe anemia, which could lead to falsely elevated or lowered HbA1c)[96]
    - Recent blood donation or blood transfusion (self-report, past 4 months)
    - Human Immunodeficiency Virus (self-report)[97]
- Allergies to nuts
- For women, current pregnancy, breastfeeding, or plans to become pregnant during the study
- Consumption of ≥21 alcoholic drinks per week or consumption of ≥6 drinks per occasion
- Current or planned residence making it difficult to meet trial requirements (due to distance from study site and/or challenges regularly traveling to site)
- Current participation in another lifestyle intervention trial or a pharmaceutical trial
- Participation of another household member in the study; employees or persons living with employees of the study
- Other concerns regarding ability to meet trial requirements, at the discretion of the principal investigator or study coordinator

7. Recruitment and Randomization

7.A. Recruitment

We will recruit study participants from the greater New Orleans area. The initial focus of our recruitment will be mass mailing of individuals in the greater New Orleans area and recruitment through placing flyers and brochures in the community. If community-based recruitment is not successful or cost-effective, we will recruit out-patients from the Ochsner Health System and

Tulane Medical Center (TMC), from which our group has prior experience recruiting patients. For this recruitment, we will focus on electronic searches of medical records and laboratory databases. We will also carry out targeted solicitation of referrals from physicians who care for patients likely to meet our inclusion and exclusion criteria.

In addition to this recruitment plan, we have several contingency plans: (1) extending recruitment to other medical centers in the New Orleans area, including the Veterans Affairs Medical Center (VAMC), East Jefferson General Hospital, and West Jefferson Medical Center, with whom Tulane has well-established relationships; (2) TV/radio advertisements; (3) arrange a news story about the study in local TV newscasts, radio talk programs, and/or print media; and (4) conduct outreach at local community events and churches. We have ample experience with each of these approaches.

Based on U.S. Census Bureau 2016 population estimates, the metro New Orleans area population was approximately 35% African American or black and 9% Hispanic.[94] Given our prior experience and population distribution in this region, we plan to recruit a distribution of study participants with 40% ethnic minorities.

7.A.1. Mass mailing, placement of brochures/flyers in community, TV/radio/billboard advertisements: Community recruitment approaches include: mass mailing individuals who live in the New Orleans area; brochures/flyers in the community; emailing the brochure; TV, radio, poster, or billboard advertisements in the New Orleans area. Interested individuals will be pre-screened; those at elevated risk for prediabetes or T2D, i.e. individuals who are obese or overweight and/or have a sufficiently high score from the American Diabetes Association's Diabetes Risk Assessment Questionnaire will be invited into the clinic for a screening visit.

7.A.2. Database searches and targeted mailing: We will request IRB approval for a waiver for HIPAA authorization to conduct database searching and hospital chart reviews for identification of potentially eligible study participants. After identifying a potentially eligible patient, we will send this individual an invitation letter signed by the investigators and/or the individual's personal physician. Individuals who express interest in the study will have a pre-screening telephone interview for confirmation of potential eligibility. Those still interested and potentially eligible for enrollment will be scheduled for a visit at the COBRE study clinic.

7.A.3. Referrals and Local Partnerships: In addition to local physician referrals, we will explore holding outreach community events in the New Orleans area. Local community partners may post information about the study on their social media platforms. We may also identify potentially interested volunteers from a Tulane volunteer recruitment registry. We will also consider recruiting potentially eligible individuals who were screened or participated in other clinical studies in our clinic and have expressed interest in being contacted about future research projects. Potential participants referred by their physician or community partners will have a pre-screening telephone interview to confirm potential eligibility. Those who are still interested and potentially eligible for the study will be scheduled for a screening visit.

7.B. Randomization

Once eligibility has been confirmed, the study coordinator will ensure that all key variables have been entered into the trial-specific database. Additionally, the study coordinator will ensure any outliers have been recognized and reconciled, and will ensure receipt of informed consent prior to enrolling the volunteer in the study as a trial participant. Eligible participants will be *randomly assigned to the behavioral intervention or the usual diet group in a 1:1 allocation ratio*. Randomization will be stratified by sex, *using a random block group size (4, 6) to maintain balance among groups over time*. A statistician from the Methodology/Biostatistics Unit within the COBRE Clinical Research Core, who is not directly involved in the study and is not located at the clinic, will conduct the randomization. After ascertaining eligibility, clinical personnel will

telephone the Methodology/Biostatistics Unit to obtain a randomization assignment. All randomization assignments will be created by a computer program and only accessible to the Staff at the Methodology/Biostatistics Unit. Once randomization assignment has been made, trial participants will be considered to be officially randomized into the study and every effort will be made to obtain complete follow-up information.

7.C. Masking

Clinical and laboratory staff members who assess outcomes will be blinded to the intervention assignment of participants. As this is a dietary intervention trial, study participants, and study staff members who conduct the intervention cannot be blinded to the intervention assignment. Participants will be instructed to avoid sharing knowledge of their treatment assignment with data collection staff members. We will have intervention visits and data collection study visits on different days and in separate locations. Data collectors will also use verbal cues and visual props to remind participants not to disclose treatment assignment. Investigators will be blinded to intervention assignment.

7.D. Safety Considerations

Participants with HbA1c >7.9% will be referred to a clinician for follow-up[98], though we will continue to collect data on these participants.

8. Dietary Intervention Program

8.A. Low-carbohydrate dietary intervention group

Participants randomized to the low-carbohydrate intervention group will receive counseling aimed at reducing carbohydrate consumption. Phase I (*Go Low*) will occur for the first 3 months, with a goal of <40 g digestible carbohydrates per day. Key recommended foods will include: non-starchy vegetables, fish, poultry, lean meat, eggs, olive oil and other plant-based unsaturated oils, unsweetened/unsalted nuts and seeds, nut butters, and avocados. We will recommend moderate consumption of cheese and unsweetened Greek yogurt and low-carbohydrate milk. During Phase I, we will recommend limiting or avoiding other dairy, fruits, legumes, beans, and grains. Phase II (*Keep it Low*) will run from months 4 through 6. The net carbohydrate goal for this phase will be <40 g to <60 g, with the objective to choose a target that is as low as possible (e.g., as close to <40 g as possible). At 3 months, we expect different participants will have different levels of intake, with some consuming <40 g of net carbohydrates and others consuming levels higher than 60 g net carbohydrates per day. We have designed the intervention to accommodate various scenarios, with the goal of achieving as low of an intake as possible. For participants who consume >60 g per day, we will recommend they aim to either reduce or maintain carbohydrate intake.

At a brief check-in at 3-months, if participants are satisfied with their current intake, we will instruct them to stay at this intake level (though will recommend they try to reduce intake, if possible, if they are eating >60 net grams of carbohydrates or have not yet reached the study goal of <40 net grams of carbohydrates). If they prefer to make modifications, we will provide guidance on how to modify carbohydrate intake (either decrease or increase). If they choose to add back carbohydrates, we will recommend that participants add 5 grams of carbohydrates for two weeks at a time (e.g., from 40 to 45 g for 2 weeks), for a maximum total of <60 g net carbohydrates. If they choose to further reduce carbohydrate intake, we will provide guidance on this as well. As they modify carbohydrate intake, we will have them monitor their satisfaction with their diet and energy levels. If adding back carbohydrates, we will recommend participants

add back in foods like fruit (preferably berries), legumes, beans, and dairy, while still aiming to keep total carbohydrate intake as low as possible (e.g., as close to <40 net grams as able).

Materials: We will develop a handbook that will be given to participants. This will contain diet guidelines, recipes, meal planners, shopping lists, and guides for counting carbohydrates and reading nutrition labels.

Meals: Participants will select their own menus and prepare or buy their meals according to the guidelines. We will provide participants with supplemental low-carbohydrate foods, such as nuts, olive oil, and other products, to help facilitate their compliance.

Counseling: Sessions and materials will focus on instructing participants to reduce digestible carbohydrate consumption by increasing consumption of protein and fat, with a particular emphasis on increased consumption of heart-healthy unsaturated fats. After randomization, participants will begin the 3-month *Go Low* phase consisting of individual counseling sessions each week for the first four weeks, followed by 4 group sessions held every other week with phone follow-ups occurring in between group sessions. During the *Keep it Low* phase, months 4 through 6, participants will attend 3 monthly group sessions and have 3 telephone follow-ups. There will be a total of 11 in-person sessions and 7 telephone follow-ups over the full study period. Sessions will be approximately one hour and phone calls will be no longer than thirty minutes. These sessions will consist of diet instruction and supportive counseling, addressing results from 24-hour dietary recalls, teaching self-monitoring and individual diet goal changes, and provide suggestions for replacing usual foods containing carbohydrates. Study materials will be modified from materials used in the successful MACRO study. The JFI will work closely with Drs. He and Bazzano to develop the intervention based on the MACRO study.
Carbohydrate goal: In the MACRO study, carried out among individuals from the same study region, a behavioral intervention aimed at reducing digestible carbohydrate intake to <40 g/day led to reductions in weight and improvements in cardiovascular risk factors among individuals with obesity.[30] Additionally, restriction of carbohydrate intake to <50g/day is sufficient to induce ketosis.[99] Our rationale for having a multi-phased intervention is to allow participants to see how they feel on the more restrictive diet, and see how they respond to small increases in carbohydrate consumption. A similar technique was applied in the DIETFITS published study.[100] The higher target of 60 net grams may be more sustainable long-term and also allows for consumption of healthy higher-carbohydrate foods, such as berries, legumes, beans, and limited whole grains.

8.B. Usual Diet Group

We will provide participants randomized to the usual diet group written information with standard dietary advice. Participants randomized to the usual diet group will not receive ongoing dietary recommendations. Moreover, they will continue to receive clinical care recommendations from their physician(s). To encourage continued participation in the study and minimize the difference in participant contact with study staff, participants will be offered optional monthly educational sessions on topics unrelated to the study intervention, such as retirement planning or health insurance options.

In another effort to encourage continued participation, patients randomized to the usual diet group will be offered a copy of all available intervention materials, as well as an optional one-hour counseling session with the interventionist following their final study visit.

8.C. Physical Activity

At baseline, both groups will receive written information in study materials with standard physical activity recommendations.

8.D. Dietary Adherence

Strategies for measuring compliance: For the low-carbohydrate diet, adherence will be assessed through measurement of urinary ketones at 3-month visit and 6-month TV. Study staff will make all reasonable effort to maintain adherence of participants to the visit schedule and diet intervention. Walnut consumption will be tracked throughout the study in the low-carbohydrate diet group.

Strategies for improving compliance:

- At the screening visit, we will ask participants who does most of the cooking in their households and how often they eat outside of the home. Results from the screening visit will help to select a group of cooperative study participants.
- Visits will be scheduled at the participants' convenience and to minimize waiting time and trips to the clinic; reminder calls, texts or emails (chosen based on participant preference) will also be made prior to clinic visits.
- We will hire enthusiastic staff who have good interpersonal skills.
- Participants will receive $40 gift cards for use at food stores or retailers when they complete each study visit, for a maximum of $120 over the study period.
- Participants will receive $25 gift cards when they return the stool specimens, for a maximum of $75 over the study period.
- Participants will be offered small gifts (such as pens or magnets with study logo) to encourage compliance and adherence to the study protocol.
- Participants will be provided with recipes, shopping lists, and food samples for the intervention diet.

9. Safety of Dietary Intervention

On the low-carbohydrate diet, participants will be encouraged to primarily focus on replacing carbohydrates (particularly highly refined carbohydrates) with foods such as non-starchy vegetables, olive oil and other plant-based unsaturated oils, nuts and seeds, avocados, eggs, seafood, tofu, poultry, and lean meat. Consumption of more unsaturated fats, protein, and fiber will be recommended. Participants will not be encouraged to increase consumption of saturated fats and will be advised to avoid consumption of trans fats.

In animal studies, a high-protein diet has decreased renal function[101] In the MACRO study, on average, participants tended to increase consumption of fats, rather than protein.[30] The safety and tolerability of low-carbohydrate diets have previously been assessed in many studies, including MACRO.[30] A number of studies, including some in individuals with T2D, have found no clinically significant increases in estimated glomerular filtration rates,[33,38–40] microalbuminuria,[102] albumin excretion rate,[33,38] urinary albumin,[38] serum creatinine,[38,40,102–107] albumin,[40] or uric acid levels.[103–106] A small study of a very low-carbohydrate diet found significant increase in uric acid excretion, but no significant change in 24-hour urine creatinine clearance.[106]

Study staff will carefully monitor participants' BP, and HbA1c more often than what would occur in routine healthcare visits. A Data Safety and Monitoring Board (DSMB) consisting of an independent group of experts in areas such as biostatistics, nutrition medicine, general internal medicine, and endocrinology has been formed. The board will regularly review trial progress, including unblinded interim results, and can recommend that the trial prematurely end if participants are at risk or if it becomes clear that the trial will not be able to yield statistically significant results.

10. Data Collection

10.A. Study visits

After the initial pre-screening interview, in which data on medications and medical conditions will be collected, there will be one screening visit, one randomization visit, a follow-up study visit at 3 months and a termination visit (TV) at 6 months. *Table 3* summarizes study procedures and data collected at each study visit.

Table 3. Study visit schedule and data collection

	Pre-screen	SV	RZ	3 month	6 month TV
Pre-screen eligibility	X				
Informed consent		X			
Compliance form		X			
Eligibility confirmation			X		
Randomization			X		
Demographics		X			
Medical history questionnaire			X	X	X
Medications		X	X	X	X
Lifestyle (alcohol, cigarette smoking)			X	X	X
Physical activity			X	X	X
24-hour diet recall*			X	X	X
3 BP measurements		X	X	X	X
Body weight			X	X	X
Height			X		
Waist circumference			X	X	X
Spot urine test			X	X	X
Blood samples		X	X	X	X
Stool specimen			X	X	X
Side-effects assessment			X	X	X
Nut Consumption					X

*Two 24-hour dietary recalls will be collected; one should cover a weekend and the other a weekday.

10.A.1. Screening/baseline visits:

Participants will be pre-screened by phone or in-person by study staff to confirm eligibility for the Screening Visit. An approved questionnaire will be used to ensure the patient is eligible to attend the in-clinic screening visit, and the patient will be scheduled thereafter.

At the screening visit (SV), participants will provide informed consent for the screening and main study. Study staff will schedule the SV and randomization (RZ) visit at least 2 days apart. If a participant is eligible based on the screening visit, he or she should be scheduled for the RZ

once a sufficient group of eligible individuals has been recruited. Participants should be scheduled for the RZ at least 2 days and no more than two weeks after the screening visit.

At the screening visit, we will collect information on inclusion/exclusion criteria, medication use, and demographics. At this visit, we will also have participants complete a compliance form, to assess their ability to complete the study and attend all intervention and study visits. We will also measure blood pressure, and take blood samples. Women will be asked if they are postmenopausal or if they had surgical sterilization (such as a tubal ligation). If not, they will be tested for pregnancy through a spot urine test; pregnant women will be excluded from the study. Blood samples will be collected for measurement of HbA1c, and, if not available from medical records in the past 3 months, serum creatinine and hemoglobin. These blood samples will be used for excluding individuals with HbA1c <6.0 or >6.9, eGFR ≤45 mL/min/1.73 m^2, or hemoglobin <11 mg/dL.

Before the start of the intervention, participants will complete two 24-hour dietary recalls. At least one (and potentially both) 24-h recalls will be collected by phone. At the RZ, participants will learn about the randomization process. After requirements for eligibility have been satisfied, participants will formally be enrolled into the study and randomized to either the active intervention or control group. We will collect fasting blood samples for later measurement of glucose, insulin and lipids and other potential future measurements. At this visit, we will measure blood pressure, body weight, height, and waist circumference. We will collect information on medical history, lifestyle risk factors, and physical activity. A spot urine sample will be collected to measure ketones and other markers and participants will be asked to collect a stool specimen for potential use in future microbiome studies.

10.A.2. Follow-up visits and final visit: A follow-up visit will occur at 3 months after randomization. A final visit will occur at 6-months post-randomization. These visits should be scheduled as close as possible to the 3-, or 6-months after randomization. At each visit, study staff will conduct dietary recalls and anthropometric measures, take 3 BP measurements, collect a fasting blood sample to measure HbA1c. At least one (and potentially both) 24-h recalls will be collected by phone. We will also collect samples for later measurement of glucose, insulin, lipid profile, and other potential future measurements. A spot urine sample will be collected to measure ketones and other markers. Participants will be asked to collect a stool specimen for potential use in future microbiome studies. Information about participants' nut consumption over the past 6 months will be collected at the 6-month TV. At study termination, we will mail participants and/or their physicians a summary of trial results and a record of participant BP, anthropometric measures, and laboratory data from the trial period. Throughout the study period, we will provide participants with their blood pressure and weight. We will request that, if possible, a participant who is prescribed a diabetes medication during the course of the study comes into the clinic for an additional separate supplemental visit for a fasting blood sample collection prior to starting the medication. Maintaining follow-up of all randomized participants is of utmost importance to ensure data integrity. In the event that a participant moves during the study we may request that the participant provides a fasting blood sample at a local LabCorp facility (this will be described on the informed consent form).

10.B Study measurements

Measurements obtained for this study will be based on those used in other clinical trials and epidemiologic studies. We are familiar with strengths and limitations of such measurements and the importance of staff training and certification and use of stringent quality control procedures.

10.B.1. Questionnaires: Study staff will administer a *medical history questionnaire* at the RZ, 3-month visit and 6-month TV to assess medical conditions that would necessitate changes in

therapy or medical management beyond this study's scope. At the SV, *demographic factors* will be collected. At the SV, RZ, 3-month and 6-month TV, we will also administer *medication tracking questionnaires*. At RZ, 3-month, and 6-month TV, we will collect health behavior and physical activity data.

10.B.2. Dietary measurements: Two 24-hour dietary recalls will be obtained from participants at baseline. At or close to both the 3-month and 6-month TV, two more 24-hour dietary recalls will be collected. The second sets of two recalls will be collected as closely as possible to 3- or 6-month dates after randomization, with a goal of being within 2 weeks of the respective follow-up period. For each time point, one recall should reflect week day consumption, while the other should reflect weekend day consumption and the two recalls should be at least 2 days apart from each other. We will use computer software (Nutrition Data System for Research [NDS-R]) and food models used in previous and ongoing studies. Recalls will be conducted by a study staff member who has been certified in the use of NDS-R. Food composition tables of the NDS-R will be used to calculate dietary nutrient intakes. The baseline dietary assessment will be done to characterize individual dietary intake of carbohydrates and other macronutrients. During the intervention, repeated measurements will be used to monitor short-term changes in mean dietary nutrient intake between the intervention and usual diet groups.

10.B.3. Assessment and management of adverse effects: At RZ, 3-month visit, and 6-month TV, we will administer *symptom questionnaires*, which will consist of a checklist to identify participant complaints. Prior studies of low-carbohydrate dietary interventions suggest that participants may experience a range of adverse effects, including constipation, halitosis, fatigue, headache, thirst, polyuria, and nausea. Severe adverse events will be promptly reported to the DSMB. Management of adverse effects will be based on the protection and safety of the participant. If severe adverse effects are reported by a participant, carbohydrate intake will be examined and altered in order to mitigate the adverse effect. In any extreme cases, participants will be instructed to stop the intervention. As this is a behavioral intervention, adverse effects should be rare.

10.B.4. BP and anthropometric measures: BP will be measured 3 times at the screening visit and at each follow-up visit. To minimize random error, averages of the measurements at the screening and randomization visits will be taken. BP will be measured by a standard automated blood pressure measurement device (OMRON HEM-907 XL Professional Digital Blood Pressure Monitor) and performed by experienced staff, trained and certified in BP measurement. Prior to BP measurements, participants will be asked to rest quietly in a seated position with their feet supported for at least 5 minutes; staff members will use an appropriate cuff size over a bare arm. Trained and certified staff will measure weight using a calibrated balance beam scale at the screening visits, and follow-up visits. Weight and height will be measured at the RZ, and weight will also be measured at the subsequent visits.

10.B.5. Blood samples: For SV, participants will not need to fast. For RZ, 3-month visit, and 6-month TV, participants will be instructed to fast for 12 hours prior to blood sample collection. At SV, blood samples will be collected to measure HbA1c, hemoglobin, and serum creatinine. At RZ, fasting blood samples will be collected and stored as serum/plasma, whole blood aliquots and buffy coats at -80°C for future analyses, including measurement of glucose, insulin and lipids, and potential future metabolomics or genetic analyses. At 3-month visit and 6-month TV, we will collect fasting blood samples to measure HbA1c and will also store additional serum/plasma, whole blood aliquots and buffy coats at -80°C for future analyses, including measurement of glucose, insulin and lipids, and potential future metabolomics or genetic analyses. Details of the blood sample collection are shown in *Table 4*. In the event that a participant moves, we may request that they collect a fasting blood sample at a local LabCorp and we will measure HbA1c, glucose, insulin, and lipids at the 3 and 6-month visits. HOMA-IR will be calculated from fasting glucose and insulin.

Table 4. Study visit schedule for specimen collection

	SV	RZ	3 month	6 month TV
HbA1c	X		X	X
Serum creatinine	X			
Hemoglobin	X			
Storage of aliquots and buffy coats (for measurement of glucose, insulin, lipids, other potential future measurements)		X	X	X
Urine sample for pregnancy	X			
Urine sample for ketones and other markers		X	X	X
Stool sample		X	X	X

10.B.6. Urine collection: Study staff will collect spot urine specimens at RZ, interim visit, and the final visit to measure ketones.

10.B.7. Stool Specimen Collection: Stool samples will be collected from all eligible and willing trial participants during the RZ, 3-month, and 6-month TV, following the protocol set forth by the Human Microbiome Project.[108,109] Participants will be given a stool collection kit, which includes the specimen collection container, a shipper for home collection, and a form to record the timing and pertinent information related to specimen collection. Participants will be instructed to return the kit to the clinic within two days of collection (in-person or via shipping), at which time stool samples are stored at -80°C until analyses. Kits and instructions for collection will be reviewed by study staff with the participant at the required visits.

11. Quality Assurance and Quality Control

Throughout the study, we will implement rigorous quality assurance (QA) and quality control (QC) measures. We describe some of these approaches here.

11.A. Manual of procedures

We will develop a manual of procedures (MOP) based on the MOP successfully used in the MACRO behavior change clinical trial. The MOP will provide instructions and describe procedures for staff training and certification, participant recruitment, intervention counseling, study form completion and procedures, data entry, safety measures (e.g., methods for emergency participant/primary care physician contact).

11.B. Training and certification

Trial personnel will all be required to participate in a training session before the start of any trial procedures or measurements. Training will include particular attention to core trial elements, such as recruitment, retention, intervention, completeness and quality of data

acquisition (e.g., blinding procedures for the data collectors), and data entry. Separately, intervention staff will meet and receive detailed instruction on trial approaches to group and individual behavior change counseling and procedures that ensure data collectors are blinded to treatment assignment. Throughout the trial, periodic re-training/certification sessions will be conducted to ensure all trial-related procedures are being applied in a standard manner. Investigators will also monitor all aspects of study performance to ensure study goal achievement, timeliness and completeness of study visits, completeness and quality of data collection, intervention adherence, and adequacy of the procedures separating data collection from intervention. Study staff and investigators will pay particularly careful attention to the quality of all laboratory measurements, particularly key exposure, outcome, and intervention monitoring variables.

12. Sample Size and Statistical Power

The primary outcome will be the difference in the change of HbA1c between the intervention and usual diet groups from baseline to 6 months of follow-up. In the United States National Health and Nutrition Examination Survey, when sub-setting to the range of HbA1c of 6.0–6.9% among individuals who do not report use of diabetes medication, the cross-sectional standard deviation is 0.24% using data from 2000 through 2016 (n=4117; https://wwwn.cdc.gov/nchs/nhanes/). In studies with 6-month follow-up among individuals with HbA1c 5.7 to 6.4% or other characteristic(s) (e.g, elevated fasting glucose or metabolic syndrome) that put them at elevated risk of type 2 diabetes, the within-person correlation between baseline and follow-up HbA1c was estimated to be between 0.5 and 0.84.[110–113] As all participants in our study will be within a specific range of HbA1c at baseline, we expect this may lead to smaller within-person correlation of HbA1c over time than studies that do not restrict to a narrow range of HbA1c at baseline. Assuming a within-person correlation of 0.5 and an SD of 0.24% for change in HbA1c, we would need to have 90 individuals to have 85% power to detect an approximate difference in change of HbA1c of 0.15% between the intervention and usual diet groups.

In a follow-up analysis of the DPP, a 0.15% decrement in HbA1c at 6 months was associated with a 16% lower risk of incident T2D, adjusting for treatment arm, age, sex, race/ethnicity, household income, and baseline HbA1c.[115] In a pooled analysis from the Atherosclerosis Risk in Communities and Framingham Heart Studies, a 0.15% higher HbA1c was associated with an 16% higher risk of T2D over 20 years, a 30% higher risk over 8 years, and a 13% higher risk after 8 years, after adjusting for age, sex, fasting glucose, triglycerides, HDL, family history of T2D, SBP, and BMI.[116] Additionally, in EPIC-Norfork, a difference of HbA1c of 0.15% was associated with approximately a 5% higher risk of CHD among those free of CHD, stroke, diabetes, and with HbA1c <7.0%.[117]

Table 5. Minimum detectable differences

	SD	Detectable Difference
HbA1c, %	0.24	0.15
Fasting glucose, mg/dL	12.5	8.0
Fasting insulin, mU/mL	7.0	4.5
HOMA-IR	2.0	1.3
SBP (mmHg)	4.2	2.7
DBP (mmHg)	3.0	1.9
Total/HDL ratio	1.2	0.77
Body weight (kg)	6.6	4.2
Waist circumference (cm)	8.0	5.1
Estimated CVD risk (%)*	6.7	4.8

*10-y CVD risk, among estimated 85% or 76 participants free of CVD

In the DPP study, HbA1c was 0.15% lower among those in the lifestyle intervention arm than in the control arm at 6-months follow-up; HbA1c at study commencement was 5.9% and levels increased in the control arm and decreased in the behavioral intervention arm.[22] In a small behavioral intervention study (n=34) of a very low-carbohydrate ketogenic diet compared

with a moderate carbohydrate, calorie-restricted, low-fat diet among individuals with type 2 diabetes or prediabetes (HbA1c >6.0%) and elevated body weight (BMI >25), mean HbA1c decreased from 6.6% to 6.1% in the very low-carbohydrate group and from 6.9% to 6.7% in the moderate carbohydrate group over a 12-month period; additionally, the very low-carbohydrate group had greater reductions in glucose-lowering medications.[37]

Detectable differences are listed for HbA1c and secondary outcomes in *Table 5*, based on data from prior studies.[30,53,56,118] These were calculated for unpaired t-tests, power of 85%, two-sided alpha of 0.05, and assuming 80% follow-up of 112 participants, which would yield an estimated 90 participants.

13. Data Management and Analysis

13.A. Data management

Personnel will double-enter all study data. Prior to entering data, the study coordinator will conduct quality control on all study forms, study measures, and laboratory data. Data will be cleaned and logically checked.

13.B. Data analysis plan

The data will be analyzed using *intention to treat*; this involves comparing the primary and secondary outcomes between participants based on randomization assignment.

13.B.1. Exploratory data analysis: We will express group data for variables with a normal distribution as mean ± standard deviation and for variables not normally distributed as median and interquartile ranges (or will log-transform the variables to obtain a normal distribution). We will compare means and percentages of baseline variables across the intervention and usual diet group to assess similarity of these groups at randomization.

13.B.2. Primary, secondary, and exploratory outcomes: We will test the primary study hypothesis that there is a greater reduction in HbA1c from baseline to 6 months in the intervention group than in the usual diet group. To do this, we will use a mixed-effects linear regression model, to test the null hypothesis that there is no difference in change between baseline and 6-month HbA1c for the intervention group compared with the usual diet group (Specific Aim 1). To do this, we will use the following model:

$$Y_{i,j,k}|T = b_0 + b_1G_i + b_2T_{3\text{-months}} + b_3T_{6\text{-months}} + b_4G_iT_{3\text{-months}} + b_5G_iT_{6\text{-months}} + a_{0i,j} + e_{i,j,k}$$

where $Y_{i,j,k}$ is HbA1c for the jth participant (P) nested in the ith intervention group (G) at time T_k (baseline, 3 months, and 6 months). This model includes a random intercept ($a_{0i,j}$) and error term $e_{i,j,k}$. The interaction term ($b_5G_iT_{6\text{-months}}$) allows for an interaction between group and $T_{6\text{-months}}$, which is equal to 0 for baseline and 3-months and 1 for 6-months. Rejection of the null hypothesis (b_5=0) indicates there is a significant difference in change of HbA1c from baseline to 6 months between the intervention and control groups. In this model, participants are assumed to be random effects and intervention group, time, and the interaction terms are fixed effects.

We will use a similar approach to test the difference in the change between baseline and 6-month values of secondary outcomes (Specific Aim 2) and exploratory outcomes. In exploratory analyses, we will also test the difference in the change between baseline and 3-month values of outcomes (HbA1c, fasting glucose, systolic blood pressure, total-to-HDL cholesterol, body weight, insulin, HOMA-IR, diastolic blood pressure, waist circumference, and estimated CVD risk). We will also explore the use of mixed effects models to assess overall net change in HbA1c (by treating time as a linear variable) and other outcomes over the full study period. For each outcome, we will test modeling assumptions and will consider transformation of outcomes, if needed, to meet modeling assumptions. As the mixed-effects model assumes missing at

random, we will also perform sensitivity analyses using Markov-chain Monte Carlo techniques to impute missing values of the outcomes, using covariates for this imputation and will compare results from this analysis with our primary findings.

15. Anticipated Challenges

Recruitment: Many clinical trials have challenges recruiting sufficient numbers of volunteers who meet the trial inclusion and exclusion criteria and demonstrate a high level of adherence to trial protocol requirements. Fortunately, the investigators are very experienced with clinical trial recruitment, and have several contingency options that can be used to meet unanticipated shortfall in recruitment.

Intervention: The investigators are aware of and understand challenges related to achieving and maintaining compliance with a behavioral intervention. The investigators have much experience and success in behavioral interventions, with recent success of a behavioral intervention aimed at reducing carbohydrate intake among participants from the New Orleans area. The JFI will collaborate with Drs. He and Bazzano to develop the intervention program based on the MACRO study and will assess potential barriers to implementation and intervention adherence (such as financial concerns and range of dietary patterns of study participants) during the planning and pilot phase of the study.

Adherence: Though adherence and follow-up are also main challenges related to clinical trials, the investigators have an outstanding track record for studies that have recruited participants for both clinical and epidemiology studies in the Greater New Orleans area. To encourage adherence, small gifts (such as pens or magnets with study logo) will be offered to all study participants.

16. Protection of Human Subjects

This Human Subjects Research meets the definition of a clinical trial. All investigators will be required to complete the CITI (Collaborative Institutional Training Initiative) program in the ethical use of human participants in research.

16.A. Protection of human subjects from research risks

16.A.1. Risks to the subjects

Human subject involvement and characteristics: We expect to enroll 112 participants, with an equal male to female ratio. We expect to enroll approximately 30 participants who are African-Americans, and another 10 with mixed racial backgrounds, and a few Asian participants given the demographics of the greater New Orleans area. Overall, given the demographics of greater New Orleans, we estimate approximately 10% participants of Hispanic or Latino ethnicity, 50% of them with African-American racial background.

Inclusion criteria

- Men or women ages 40 to 70 years: aim to recruit 50% women
- Any race or ethnicity: aim to recruit 40% ethnic minorities (i.e., African American and Hispanic), close to population in the greater New Orleans area[94]
- HbA1c 6.0–6.9%
- Willing and able to provide informed consent

Exclusion criteria

- Diagnosed type 1 diabetes mellitus based on patient self-report
- Use of agents affecting glycemic control (medications for diabetes, oral glucocorticoids) within the past three months prior to enrollment based on patient self-report
- Medical condition in which low-carbohydrate diet may not be advised
    - o Estimated glomerular filtration rate (eGFR) ≤45 mL/min/1.73 m², which is close to the 5th percentile of eGFR among non-diseased individuals of 70 years of age[95]
    - o Self-report of liver disease due to hepatitis or alcohol; osteoporosis; untreated thyroid disease; gout; or cancer (other than non-melanoma skin cancer) requiring treatment in the past year, unless prognosis is excellent
- Factors that may affect HbA1c:
    - o Hemoglobin <11 mg/dL (cutpoint for moderate-to-severe anemia, which could lead to falsely elevated or lowered HbA1c)[96]
    - o Recent blood donation or blood transfusion (self-report, past 4 months)
    - o Human Immunodeficiency Virus (self-report)[97]
- Allergies to nuts
- For women, current pregnancy, breastfeeding, or plans to become pregnant during the study
- Consumption of ≥21 alcoholic drinks per week or consumption of ≥6 drinks per occasion
- Current or planned residence making it difficult to meet trial requirements (due to distance from study site and/or challenges regularly traveling to site)
- Current participation in another lifestyle intervention trial or a pharmaceutical trial
- Participation of another household member in the study; employees or persons living with employees of the study
- Other concerns regarding ability to meet trial requirements, at the discretion of the principal investigator or study coordinator

Sources of materials: Medical information data will be collected from the participant and, after written authorization for the disclosure of protected health information, from participants' medical records. Blood and urine samples will be collected to measure HbA1c, glucose, insulin, lipids, creatinine, ketones, and test for pregnancy. Blood pressure, height, weight, waist circumference and other physical measurements will be obtained.

Potential Risks: Risks associated with venipuncture, the diet intervention, and collection of confidential medical information are the main risks associated with this study. There is a small risk of bruising and a rare chance of local infection associated with standard venipuncture to collect blood samples. The dietary intervention has minimal risks. Reduced carbohydrate consumption may lead to adverse effects, including constipation, halitosis, fatigue, headache, thirst, polyuria, and nausea. At RZ, 3- , and 6-month visits, we will administer *symptom questionnaires*, which will consist of a checklist to identify participant complaints. Adverse effects of the low-carbohydrate diet will be assessed at counseling sessions. Severe adverse events will be promptly reported to the DSMB. Management of adverse effects will be based on the protection and safety of the participant. If severe adverse effects are reported by a participant, carbohydrate intake will be examined and altered in order to mitigate the adverse effect. In any extreme cases, participants will be instructed to stop the intervention. Furthermore, there is a potential risk of unauthorized disclosure of medical information. This risk will be minimized by using a strict protocol for data processing and by the appropriate training of all study personnel. Protection against risks is described in more detail below.

16.A.2. Adequacy of protection against risks

Recruitment and Informed Consent: The proposed study will be conducted following strict guidelines for the protection of the rights of human volunteers. The informed consent document will be signed by all participants at the screening/baseline visit. The informed consent form will be developed in the first year of the study and approved by the IRB. The consent form will clearly state the purpose, eligibility criteria, and protocol of the study, the potential benefits and risks of participation, and the subject's right to refuse to participate or withdraw. Prior to signing the informed consent, research staff will review the details of the consent form orally with the participant, and answer any questions that the participant has concerning participation in the study. In the case of potential participants with low or no literacy, the informed consent form will be read to the participant in its entirety and any questions will be answered prior to the participant signing or marking the form. Specifically, the following must be accomplished during the informed consent process:

- The participant must be informed that participation in the study is voluntary and that refusal to participate will involve no penalty or loss of benefits.
- The participant must be informed of the purpose of the study and that it involves research.
- The participant must be informed of any alternative procedures, if applicable.
- The participant must be informed of any reasonably foreseeable risks.
- The participant must be informed of any benefits from the research.
- An outline of safeguards to protect participant confidentiality will be included, as well as an indication of the participant's right to withdraw without penalty. This should be balanced with a discussion of the effect that withdrawals will have on the study, and the responsibility a participant has, within limits, to continue in the study if he or she decides to enroll.
- The participant must be informed of his or her right to have questions answered at any time and whom to contact for answers or in the event of research-related injury.
- The participant must be informed as to whether or not compensation is offered for participation in the study and/or in the event of a medical injury.
- The participant must be informed that he/she will be notified of any significant changes in the protocol that might affect their willingness to continue in the study.

Written authorization for the disclosure of protected health information will be obtained during the consent process on a separate form (HIPPA authorization), if applicable.

Protection against Risk: The protocol and informed consent will be approved by the Institutional Review Boards at Tulane University Health Sciences Center and any other participating institution for recruitment. The study participants will be given contact information for the Study Coordinator and Study PI to answer questions or to report complaints. Patients can withdraw from the study at any time without any effect on their medical care. Standard techniques will be used by trained, certified phlebotomists or study nurses for blood sample collection at the study clinics where emergency medical equipment is available. Confidentiality of all data collected during the study will be maintained through the use of unique, encrypted study identification (ID numbers, rather than patient names, in the study database). These encrypted ID numbers will not be mathematical derivation of the subject's medical record number or any other patient identifier. No information will be disclosed in an individually identifiable form in any type of presentation or publication. There will be password-required access to the computerized file linking the study numbers to patient identifiers at the study clinics. Password-access will be restricted to pertinent research staff only. All study data will be

kept in locked file cabinets in locked rooms, accessible only by study staff with permission. All data transmissions will use HIPPA-compliant password protected system and will be secured through a virtual private network.

16.A.3. Potential benefits of the proposed research to human subjects and others

Study participants may have improved glycemic outcomes, blood pressure, lipid levels, may lose weight and reduce waist circumference, and may have improvement in other risk factors for CVD and development and progression of T2D. Participants will receive multiple blood pressure, weight, waist circumference, and blood tests. If vital signs or lab values are clinically abnormal, participants will be referred to their primary care physician, or if they do not have a primary care physician, one will be assigned through the Tulane University Community Health Center, which is a part of the core clinical facilities. At the end of the study, we will provide the participants' primary care physician with a copy of all the blood pressure, weight, and laboratory results.

16.A.4. Importance of knowledge to be gained

Information from this study may contribute to reducing the onset of T2D and the burden of T2D and cardiovascular disease in Louisiana and the general US population. The study has potential for public health impact; results from this study may provide evidence in support of promoting alternative dietary styles (than the most commonly used low-fat diet) among individuals with high T2D risk or with T2D.

16.A.5. Remuneration

For screening visit 2, the two follow-up visits, and the final visits, participants will receive $40 gift cards for use at food stores or retailers (after completing each study visit), for a maximum of $120 over the study period. Participants who return a stool sample will receive $25 gift cards for each stool sample that they return, for a total of $75 for three stool samples. Participants will also receive free parking for each intervention and study visit.

16.B. Data safety and monitoring plan

A DSMB, which consists of 3 experts who are not otherwise affiliated with the COBRE program, has been formed. Study investigators for this study will prepare accurate and timely data tables and reports for the DSMB meetings. The members of the DSMB will serve in an individual capacity and provide their expertise and recommendations. The primary responsibilities of the DSMB are: 1) periodically review and evaluate the accumulated study data for participant safety, study conduct and progress, and, when appropriate, efficacy, and 2) make recommendations to the NIH concerning the continuation, modification, or termination of the trial. The DSMB considers study-specific data, as well as relevant background knowledge, about the disease, test agent, and patient population under study.

The DSMB is responsible for defining its deliberative processes, including event triggers that would call for an unscheduled review, stopping guidelines, unmasking (unblinding) and voting procedures prior to initiating any data review. The DSMB is also responsible for maintaining the confidentiality of its internal discussions and activities, as well as the contents of reports provided to it.

The DSMB will review each protocol for any major concerns prior to implementation. During the trial, the DSMB should review cumulative study data to evaluate safety, study conduct, and scientific validity and integrity of the trial. As part of this responsibility, DSMB members must be satisfied that the timeliness, completeness, and accuracy of the data submitted to them for

review are sufficient for evaluation of the safety and welfare of study participants. The DSMB should also assess the performance of overall study operations and any other relevant issues, as necessary.

Items reviewed by the DSMB include:

- Interim/cumulative data for evidence of study-related adverse events;
- Interim/cumulative data for evidence of efficacy according to pre-established statistical guidelines, if appropriate;
- Data quality, completeness, and timeliness;
- Adequacy of compliance with goals for recruitment and retention, including those related to the participation of women and minorities;
- Adherence to the protocol;
- Factors that might affect the study outcome or compromise the confidentiality of the trial data (such as protocol violations, unmasking, etc.); and,
- Factors external to the study, such as scientific or therapeutic developments, that may impact participant safety or the ethics of the study.

The DSMB will conclude each review with their recommendations to the COBRE program and NIH/NIGMS as to whether the study should continue without change or be modified or terminated. Recommendations regarding modification of the design and conduct of the study could include:

- Modifications of the study protocol based upon review of the safety data;
- Suspension or early termination of the study or of one or more study arms because of serious concerns about subjects' safety, inadequate performance or rate of enrollment;
- Suspension or early termination of the study or of one or more study arms because study objectives have not been obtained according to pre-established statistical guidelines; and,
- Corrective actions regarding a study when the performance appears unsatisfactory or suspicious.

Confidentiality must always be maintained during all phases of DSMB review and deliberation. Usually, only voting members of the DSMB should have access to interim analyses of outcome data by treatment group. Exceptions may be made when the DSMB deems it appropriate. The reason and to whom the exceptions for access to interim analyses is granted will be documented in the Closed Session Report. DSMB members must maintain strict confidentiality concerning all privileged trial results ever provided to them. The DSMB should review data only by masked study group (such as X vs. Y rather than experimental vs. control) unless or until the DSMB determines that the identities of the groups are necessary for their decision-making. Whenever masked data are presented to the DSMB, the key to the group coding must be available for immediate unmasking.

The DSMB will meet twice per year. The junior faculty investigator will attend these meetings and will be responsible for preparing and presenting up-to-date statistical reports on the progress of their trials. These reports will include data on recruitment, randomization, adherence, as well as statistical tests and special analyses requested by the DSMB. The Administrative Core will be responsible for organizing the annual in-person meeting and semiannual conference calls. They will put together data and materials submitted by junior faculty investigators and deliver them to DSMB members. The COBRE investigators will meet with the DSMB in the Open Session and not be present for the Closed Session.

Bibliography and References Cited

1. The Emerging Risk Factors Collaboration. Diabetes mellitus, fasting blood glucose concentration, and risk of vascular disease: a collaborative meta-analysis of 102 prospective studies. *Lancet*. 2010;375(9733):2215-2222. doi:10.1016/S0140-6736(10)60484-9.
2. Flaxman SR, Bourne RRA, Resnikoff S, et al. Global causes of blindness and distance vision impairment 1990-2020: a systematic review and meta-analysis. *Lancet Glob Heal*. October 2017. doi:10.1016/S2214-109X(17)30393-5.
3. United States Renal Data System. *2016 USRDS Annual Data Report: Epidemiology of Kidney Disease in the United States*. Bethesda, MD: National Institutes of Health, National Institute of Diabetes and Digestive and Kidney Diseases; 2016. https://www.usrds.org/2016/view/Default.aspx.
4. Narres M, Kvitkina T, Claessen H, et al. Incidence of lower extremity amputations in the diabetic compared with the non-diabetic population: A systematic review. *PLoS One*. 2017;12(8):e0182081. doi:10.1371/journal.pone.0182081.
5. Centers for Disease Control and Prevention. *National Diabetes Statistics Report, 2017*. Atlanta, GA: Centers for Disease Control and Prevention, U.S. Dept of Health and Human Services; 2017.
6. Centers for Disease Control and Prevention. *Long-Term Trends in Diabetes*. Atlanta, GA: Centers for Disease Control and Prevention, U.S. Dept of Health and Human Services; 2017.
7. NCD Risk Factor Collaboration (NCD-RisC). Worldwide trends in diabetes since 1980: A pooled analysis of 751 population-based studies with 4.4 million participants. *Lancet*. 2016;387(10027):1513-1530. doi:10.1016/S0140-6736(16)00618-8.
8. American Diabetes Association. Economic costs of diabetes in the U.S. in 2012. *Diabetes Care*. 2013;36(4):1033-1046. doi:10.2337/dc12-2625.
9. Zhuo X, Zhang P, Barker L, Albright A, Thompson TJ, Gregg E. The lifetime cost of diabetes and its implications for diabetes prevention. *Diabetes Care*. 2014;37(9):2557-2564. doi:10.2337/dc13-2484.
10. WHO, International Diabetes Foundation. *Definition and Diagnosis of Diabetes Mellitus and Intermediate Hyperglycaemia: Report of a WHO/IDF Consultation*. Geneva: Geneva: World Health Organization; 2006.
11. The International Expert Committee. International Expert Committee Report on the Role of the A1C Assay in the Diagnosis of Diabetes. *Diabetes Care*. 2009;32(7):1327-1334. doi:10.2337/dc09-9033.
12. American Diabetes Association. Standards of Medical Care in Diabetes - 2017. *Diabetes Care*. 2017;40(Supplement 1):S33-S43. doi:10.2337/dc16-S003.
13. Gerstein HC, Santaguida P, Raina P, et al. Annual incidence and relative risk of diabetes in people with various categories of dysglycemia: A systematic overview and meta-analysis of prospective studies. *Diabetes Res Clin Pract*. 2007;78(3):305-312. doi:10.1016/j.diabres.2007.05.004.
14. Huang Y, Cai X, Mai W, Li M, Hu Y. Association between prediabetes and risk of cardiovascular disease and all cause mortality: systematic review and meta-analysis. *BMJ*. 2016;355:i5953. doi:10.1136/bmj.i5953.
15. Tabák AG, Herder C, Rathmann W, Brunner EJ, Kivimäki M. Prediabetes: A high-risk state for diabetes development. *Lancet*. 2012;379(9833):2279-2290. doi:10.1016/S0140-

6736(12)60283-9.

16. Diabetes Prevention Program Research Group. 10-year follow-up of diabetes incidence and weight loss in the Diabetes Prevention Program Outcomes Study. *Lancet*. 2009;374(9702):1677-1686. doi:10.1016/S0140-6736(09)61457-4.
17. The DREAM (Diabetes REduction Assessment with ramipril and rosiglitazone Medication) Trial Investigators. Effect of rosiglitazone on the frequency of diabetes in patients with impaired glucose tolerance or impaired fasting glucose: a randomised controlled trial. *Lancet*. 2006;368(9541):1096-1105. doi:10.1016/S0140-6736(06)69420-8.
18. Perreault L, Pan Q, Mather KJ, Watson KE, Hamman RF, Kahn SE. Effect of regression from prediabetes to normal glucose regulation on long-term reduction in diabetes risk: Results from the Diabetes Prevention Program Outcomes Study. *Lancet*. 2012;379(9833):2243-2251. doi:10.1016/S0140-6736(12)60525-X.
19. Perreault L, Temprosa M, Mather KJ, et al. Regression from prediabetes to normal glucose regulation is associated with reduction in cardiovascular risk: Results from the diabetes prevention program outcomes study. *Diabetes Care*. 2014;37(9):2622-2631. doi:10.2337/dc14-0656.
20. Popkin BM. Nutrition Transition and the Global Diabetes Epidemic. *Curr Diab Rep*. 2015;15(9). doi:10.1007/s11892-015-0631-4.
21. Vazquez G, Duval S, Jacobs DRJ, Silventoinen K. Comparison of body mass index, waist circumference, and waist/hip ratio in predicting incident diabetes: a meta-analysis. *Epidemiol Rev*. 2007;29:115-128. doi:10.1093/epirev/mxm008.
22. Knowler WC, Barrett-Connor E, Fowler SE, et al. Reduction in the incidence of type 2 diabetes with lifestyle intervention or metformin. *N Engl J Med*. 2002;346(6):393-403. doi:10.1056/NEJMoa012512.
23. Pan XR, Li GW, Hu YH, et al. Effects of diet and exercise in preventing NIDDM in people with impaired glucose tolerance: The Da Qing IGT and diabetes study. *Diabetes Care*. 1997;20(4):537-544. doi:10.2337/diacare.20.4.537.
24. Tuomilehto J, Indstrom J, Eriksson J, Valle T, Hamalainine E, Uusitupa M. Prevention of Type 2 Diabetes Mellitus By Changes in Lifestyle Among Subjects With Impaired Glucose Tolerance. *N Engl J Med*. 2001;344(18):1343-1350. doi:10.1056/NEJM200105033441801.
25. Ramachandran A, Snehalatha C, Mary S, Mukesh B, Bhaskar AD, Vijay V. The Indian Diabetes Prevention Programme shows that lifestyle modification and metformin prevent type 2 diabetes in Asian Indian subjects with impaired glucose tolerance (IDPP-1). *Diabetologia*. 2006;49(2):289-297. doi:10.1007/s00125-005-0097-z.
26. The Diabetes Preventio Program Research Group. The Diabetes Prevention Program. Design and methods for a clinical trial in the prevention of type 2 diabetes. *Diabetes Care*. 1999;22(4):623-634. doi:10.2337/diacare.22.4.623 10.2337/diabetes.48.4.699.
27. Evert AB, Boucher JL, Cypress M, et al. Nutrition therapy recommendations for the management of adults with diabetes. *Diabetes Care*. 2013;36(11):3821-3842. doi:10.2337/dc13-2042.
28. Hu T, Mills KT, Yao L, et al. Effects of low-carbohydrate diets versus low-fat diets on metabolic risk factors: A meta-analysis of randomized controlled clinical trials. *Am J Epidemiol*. 2012;176(SUPPL. 7). doi:10.1093/aje/kws264.
29. Sackner-Bernstein J, Kanter D, Kaul S. Dietary Intervention for Overweight and Obese Adults: Comparison of Low-Carbohydrate and Low-Fat Diets. A Meta-Analysis. *PLoS*

One. 2015;10(10):e0139817. doi:10.1371/journal.pone.0139817.

30. Bazzano LA, Hu T, Reynolds K, et al. Effects of low-carbohydrate and low-fat diets: A randomized trial. *Ann Intern Med*. 2014;161(5):309-318. doi:10.7326/M14-0180.
31. Sawyer L, Gale EAM. Diet, delusion and diabetes. *Diabetologia*. 2009;52(1):1-7. doi:10.1007/s00125-008-1203-9.
32. Snorgaard O, Poulsen GM, Andersen HK, Astrup A. Systematic review and meta-analysis of dietary carbohydrate restriction in patients with type 2 diabetes. *BMJ Open Diabetes Res Care*. 2017;5(1):e000354. doi:10.1136/bmjdrc-2016-000354.
33. Larsen RN, Mann NJ, Maclean E, Shaw JE. The effect of high-protein, low-carbohydrate diets in the treatment of type 2 diabetes: A 12 month randomised controlled trial. *Diabetologia*. 2011;54(4):731-740. doi:10.1007/s00125-010-2027-y.
34. Saslow LR, Kim S, Daubenmier JJ, et al. A randomized pilot trial of a moderate carbohydrate diet compared to a very low carbohydrate diet in overweight or obese individuals with type 2 diabetes mellitus or prediabetes. *PLoS One*. 2014;9(4). doi:10.1371/journal.pone.0091027.
35. Tay J, Luscombe-Marsh ND, Thompson CH, et al. A very low-carbohydrate, low-saturated fat diet for type 2 diabetes management: A randomized trial. *Diabetes Care*. 2014;37(11):2909-2918. doi:10.2337/dc14-0845.
36. Davis NJ, Tomuta N, Schechter C, et al. Comparative Study of the Effects of a 1-Year Dietary Intervention of a Low- Carbohydrate Diet Versus a Low-Fat Diet on Weight and Glycemic Control in Type 2. *Diabetes Care*. 2009;32(7):1147-1152. doi:10.2337/dc08-2108.Clinical.
37. Saslow LR, Daubenmier JJ, Moskowitz JT, et al. Twelve-month outcomes of a randomized trial of a moderate-carbohydrate versus very low-carbohydrate diet in overweight adults with type 2 diabetes mellitus or prediabetes. *Nutr Diabetes*. 2017;7(12):304. doi:10.1038/s41387-017-0006-9.
38. Tay J, Thompson CH, Luscombe-Marsh ND, et al. Effects of an energy-restricted low-carbohydrate, high unsaturated fat/low saturated fat diet versus a high carbohydrate, low fat diet in type 2 diabetes: a 2 year randomized clinical trial. *Diabetes, Obes Metab*. 2017:n/a-n/a. doi:10.1111/dom.13164.
39. Yamada Y, Uchida J, Izumi H, et al. A non-calorie-restricted low-carbohydrate diet is effective as an alternative therapy for patients with type 2 diabetes. *Intern Med*. 2014;53(1):13-19. doi:http://dx.doi.org/10.2169/internalmedicine.53.0861.
40. Mayer SB, Jeffreys AS, Olsen MK, Mcduffie JR, Feinglos MN, Yancy WS. Two diets with different haemoglobin A1c and antiglycaemic medication effects despite similar weight loss in type 2 diabetes. *Diabetes, Obes Metab*. 2014;16(1):90-93. doi:10.1111/dom.12191.
41. Maekawa S, Kawahara T, Nomura R, et al. Retrospective study on the efficacy of a low-carbohydrate diet for impaired glucose tolerance. *Diabetes Metab Syndr Obes*. 2014;7:195-201. doi:10.2147/DMSO.S62681.
42. Davis C, Bryan J, Hodgson J, Murphy K. Definition of the mediterranean diet: A literature review. *Nutrients*. 2015;7(11):9139-9153. doi:10.3390/nu7115459.
43. Sofi F, Macchi C, Abbate R, Gensini GF, Casini A. Mediterranean diet and health status: an updated meta-analysis and a proposal for a literature-based adherence score. *Public Health Nutr*. 2014;17(12):2769-2782. doi:10.1017/S1368980013003169.
44. Jannasch F, Kröger J, Schulze MB. Dietary Patterns and Type 2 Diabetes: A Systematic Literature Review and Meta-Analysis of Prospective Studies. *J Nutr*. 2017;147(6):1174-

1182. doi:10.3945/jn.116.242552.

45. Schwingshackl L, Missbach B, König J, Hoffmann G. Adherence to a Mediterranean diet and risk of diabetes: a systematic review and meta-analysis. *Public Health Nutr*. 2014;18(7):1292–1299. doi:10.1017/S1368980014001542.

46. de Lorgeril M, Salen P, Martin J-L, Monjaud I, Delaye J, Mamelle N. Mediterranean Diet, Traditional Risk Factors, and the Rate of Cardiovascular Complications After Myocardial Infarction : Final Report of the Lyon Diet Heart Study. *Circulation*. 1999;99(6):779-785. doi:10.1161/01.CIR.99.6.779.

47. Estruch R, Ros E, Salas-Salvadó J, et al. Primary Prevention of Cardiovascular Disease with a Mediterranean Diet. *N Engl J Med*. 2013;368(14):1279-1290. doi:10.1056/NEJMoa1200303.

48. Salas-Salvadó J, Bulló M, Estruch R, et al. Prevention of diabetes with mediterranean diets: A subgroup analysis of a randomized trial. *Ann Intern Med*. 2014;160(1):1-10. doi:10.7326/M13-1725.

49. Esposito K, Marfella R, Ciotola M, et al. Effect of a Mediterranean-Style Diet on Endothelial Dysfunction and Markers of Vascular Inflammation in the Metabolic Syndrome. *JAMA*. 2004;292(12):1440. doi:10.1001/jama.292.12.1440.

50. Babio N, Toledo E, Estruch R, et al. Mediterranean diets and metabolic syndrome status in the PREDIMED randomized trial. *CMAJ*. 2014;186(17):E649-E657. doi:10.1503/cmaj.140764.

51. Esposito K, Maiorino MI, Bellastella G, Chiodini P, Panagiotakos D, Giugliano D. A journey into a Mediterranean diet and type 2 diabetes: a systematic review with meta-analyses. *BMJ Open*. 2015;5(8):e008222. doi:10.1136/bmjopen-2015-008222.

52. Micha R, Michas G, Mozaffarian D. Unprocessed red and processed meats and risk of coronary artery disease and type 2 diabetes--an updated review of the evidence. *Curr Atheroscler Rep*. 2012;14(6):515-524. doi:10.1007/s11883-012-0282-8.

53. Esposito K, Maiorino MI, Ciotola M, et al. Effects of a Mediterranean-style diet on the need for antihyperglycemic drug therapy in patients with newly diagnosed type 2 diabetes: A randomized trial. *Ann Intern Med*. 2009;151(5):306-314. doi:10.1097/OGX.0b013e3181e5a159.

54. Esposito K, Maiorino MI, Petrizzo M, Bellastella G, Giugliano D. The effects of a Mediterranean diet on the need for diabetes drugs and remission of newly diagnosed type 2 diabetes: Follow-up of a randomized trial. *Diabetes Care*. 2014;37(7):1824-1830. doi:10.2337/dc13-2899.

55. Elhayany A, Lustman A, Abel R, Attal-Singer J, Vinker S. A low carbohydrate Mediterranean diet improves cardiovascular risk factors and diabetes control among overweight patients with type 2 diabetes mellitus: A 1-year prospective randomized intervention study. *Diabetes, Obes Metab*. 2010;12(3):204-209. doi:10.1111/j.1463-1326.2009.01151.x.

56. Shai I, Schwarzfuchs D, Henkin Y, et al. Weight Loss with a Low-Carbohydrate, Mediterranean, or Low-Fat Diet. *N Engl J Med*. 2008;359(3):229-241. doi:10.1056/NEJMoa0708681.

57. Schwarzfuchs D, Golan R, Shai I. Four-Year Follow-up after Two-Year Dietary Interventions. *N Engl J Med*. 2012;367(14):1373-1374. doi:10.1056/NEJMc1204792.

58. Pan A, Sun Q, Bernstein AM, et al. Red meat consumption and risk of type 2 diabetes: 3 Cohorts of US adults and an updated meta-analysis. *Am J Clin Nutr*. 2011;94(4):1088-1096. doi:10.3945/ajcn.111.018978.

59. Hu EA, Pan A, Malik V, Sun Q. White rice consumption and risk of type 2 diabetes: meta-analysis and systematic review. *BMJ*. 2012;344:e1454. doi:10.1136/bmj.e1454.
60. Wang M, Yu M, Fang L, Hu R-Y. Association between sugar-sweetened beverages and type 2 diabetes: A meta-analysis. *J Diabetes Investig*. 2015;6(3):360-366. doi:10.1111/jdi.12309.
61. Imamura F, O'Connor L, Ye Z, et al. Consumption of sugar sweetened beverages, artificially sweetened beverages, and fruit juice and incidence of type 2 diabetes: systematic review, meta-analysis, and estimation of population attributable fraction. *BMJ*. 2015:h3576. doi:10.1136/bmj.h3576.
62. Carter P, Gray LJ, Troughton J, Khunti K, Davies MJ. Fruit and vegetable intake and incidence of type 2 diabetes mellitus: systematic review and meta-analysis. *BMJ*. 2010;341:c4229. doi:10.1136/bmj.c4229.
63. Cooper AJ, Forouhi NG, Ye Z, et al. Fruit and vegetable intake and type 2 diabetes: EPIC-InterAct prospective study and meta-analysis. *Eur J Clin Nutr*. 2012;66(10):1082-1092. doi:10.1038/ejcn.2012.85.
64. Chen M, Sun Q, Giovannucci E, et al. Dairy consumption and risk of type 2 diabetes: 3 cohorts of US adults and an updated meta-analysis. *BMC Med*. 2014;12(1):215. doi:10.1186/s12916-014-0215-1.
65. Aune D, Norat T, Romundstad P, Vatten LJ. Whole grain and refined grain consumption and the risk of type 2 diabetes: A systematic review and dose-response meta-analysis of cohort studies. *Eur J Epidemiol*. 2013;28(11):845-858. doi:10.1007/s10654-013-9852-5.
66. Ding M, Bhupathiraju SN, Chen M, van Dam RM, Hu FB. Caffeinated and decaffeinated coffee consumption and risk of type 2 diabetes: a systematic review and a dose-response meta-analysis. *Diabetes Care*. 2014;37(2):569-586. doi:10.2337/dc13-1203.
67. Li X-H, Yu F-F, Zhou Y-H, He J. Association between alcohol consumption and the risk of incident type 2 diabetes: a systematic review and dose-response meta-analysis. *Am J Clin Nutr*. 2016;103(3):818-829. doi:10.3945/ajcn.115.114389.
68. Muraki I, Imamura F, Manson JE, et al. Fruit consumption and risk of type 2 diabetes: results from three prospective longitudinal cohort studies. *BMJ*. 2013;347(aug28 1):f5001-f5001. doi:10.1136/bmj.f5001.
69. Upadhyaya S, Banerjee G. Type 2 diabetes and gut microbiome: At the intersection of known and unknown. *Gut Microbes*. 2015;6(2):85-92. doi:10.1080/19490976.2015.1024918.
70. David LA, Maurice CF, Carmody RN, et al. Diet rapidly and reproducibly alters the human gut microbiome. *Nature*. 2014;505(7484):559-563. doi:10.1038/nature12820.
71. Duncan SH, Belenguer A, Holtrop G, Johnstone AM, Flint HJ, Lobley GE. Reduced dietary intake of carbohydrates by obese subjects results in decreased concentrations of butyrate and butyrate-producing bacteria in feces. *Appl Environ Microbiol*. 2007;73(4):1073-1078. doi:10.1128/AEM.02340-06.
72. Dorans KS, Mostofsky E, Levitan EB, Håkansson N, Wolk A, Mittleman MA. Alcohol and Incident Heart Failure among Middle-Aged and Elderly Men: Cohort of Swedish Men. *Circ Hear Fail*. 2015;8(3):422-427. doi:10.1161/CIRCHEARTFAILURE.114.001787.
73. Steinhaus DA, Mostofsky E, Levitan EB, et al. Chocolate intake and incidence of heart failure: Findings from the Cohort of Swedish Men. *Am Heart J*. 2017;183:18-23. doi:10.1016/j.ahj.2016.10.002.
74. Dorans KS, Massa J, Chitnis T, Ascherio A, Munger KL. Physical activity and the incidence of multiple sclerosis. *Neurology*. 2016;87(17):1770-1776.

doi:10.1212/WNL.0000000000003260.

75. Mills KT, Chen J, Yang W, et al. Sodium Excretion and the Risk of Cardiovascular Disease in Patients With Chronic Kidney Disease. *JAMA*. 2016;315(20):2200. doi:10.1001/jama.2016.4447.

76. Chen J, Gu D, Huang J, et al. Metabolic syndrome and salt sensitivity of blood pressure in non-diabetic people in China: a dietary intervention study. *Lancet*. 2009;373(9666):829-835. doi:10.1016/S0140-6736(09)60144-6.

77. Li C, He J, Chen J, et al. Genome-Wide Gene-Sodium Interaction Analyses on Blood Pressure: The Genetic Epidemiology Network of Salt-Sensitivity Study. *Hypertension*. 2016;68(2):348-355. doi:10.1161/HYPERTENSIONAHA.115.06765.

78. He J, Streiffer RH, Muntner P, Krousel-Wood MA, Whelton PK. Effect of dietary fiber intake on blood pressure: a randomized, double-blind, placebo-controlled trial. *J Hypertens*. 2004;22(1):73-80. doi:10.1097/01.hjh.0000098164.70956.54.

79. He J, Ogden LG, Vupputuri S, Bazzano LA, Loria C, Whelton PK. Dietary sodium intake and subsequent risk of cardiovascular disease in overweight adults. *JAMA*. 1999;282(21):2027-2034. doi:10.1001/jama.282.21.2027.

80. He J, Wofford MR, Reynolds K, et al. Effect of dietary protein supplementation on blood pressure a randomized, controlled trial. *Circulation*. 2011;124(5):589-595. doi:10.1161/CIRCULATIONAHA.110.009159.

81. Thompson AM, Zhang Y, Tong W, et al. Association of inflammation and endothelial dysfunction with metabolic syndrome, prediabetes and diabetes in adults from Inner Mongolia, China. *BMC Endocr Disord*. 2011;11(1):16. doi:10.1186/1472-6823-11-16.

82. Irazola V, Rubinstein A, Bazzano L, et al. Prevalence, awareness, treatment and control of diabetes and impaired fasting glucose in the Southern Cone of Latin America. *PLoS One*. 2017;12(9). doi:10.1371/journal.pone.0183953.

83. Bazzano LA, Li TY, Joshipura KJ, Hu FB. Intake of fruit, vegetables, and fruit juices and risk of diabetes in women. *Diabetes Care*. 2008;31(7):1311-1317. doi:10.2337/dc08-0080.

84. Bazzano LA, Serdula M, Liu S. Prevention of Type 2 Diabetes by Diet and Lifestyle Modification. *J Am Coll Nutr*. 2005;24(5):310-319. doi:10.1080/07315724.2005.10719479.

85. Pollock BD, Chen W, Harville EW, et al. Differential sex effects of systolic blood pressure and low-density lipoprotein cholesterol on type 2 diabetes: Life course data from the Bogalusa Heart Study. *Journal of Diabetes*. 2017.

86. Zhang T, Li Y, Zhang H, et al. Insulin-sensitive adiposity is associated with a relatively lower risk of diabetes than insulin-resistant adiposity: the Bogalusa Heart Study. *Endocrine*. 2016;54(1):93-100. doi:10.1007/s12020-016-0948-z.

87. Pollock BD, Hu T, Chen W, et al. Utility of existing diabetes risk prediction tools for young black and white adults: Evidence from the Bogalusa Heart Study. *J Diabetes Complications*. 2016;31(1):86-93. doi:10.1016/j.jdiacomp.2016.07.025.

88. Hu T, Yao L, Reynolds K, et al. The effects of a low-carbohydrate diet vs. a low-fat diet on novel cardiovascular risk factors: A randomized controlled trial. *Nutrients*. 2015;7(9):7978-7994. doi:10.3390/nu7095377.

89. Hu T, Yao L, Reynolds K, et al. The effects of a low-carbohydrate diet on appetite: A randomized controlled trial. *Nutr Metab Cardiovasc Dis*. 2016;26(6):476-488. doi:10.1016/j.numecd.2015.11.011.

90. Rebholz CM, Reynolds K, Wofford MR, et al. Effect of soybean protein on novel cardiovascular disease risk factors: A randomized controlled trial. *Eur J Clin Nutr*. 2013;67(1):58-63. doi:10.1038/ejcn.2012.186.

91. Wofford MR, Rebholz CM, Reynolds K, et al. Effect of soy and milk protein supplementation on serum lipid levels: A randomized controlled trial. *Eur J Clin Nutr*. 2012;66(4):419-425. doi:10.1038/ejcn.2011.168.

92. Chen J, He J, Wildman RP, Reynolds K, Streiffer RH, Whelton PK. A randomized controlled trial of dietary fiber intake on serum lipids. *Eur J Clin Nutr*. 2006;60(1):62-68. doi:10.1038/sj.ejcn.1602268.

93. Overweight and Obesity Expert Panel. *Managing Overweight and Obesity in Adults: Systematic Evidence Review From the Obesity Expert Panel, 2013*.; 2013.

94. The Data Center. *Who Lives in New Orleans and Metro Parishes Now?*; 2017. https://www.datacenterresearch.org/data-resources/who-lives-in-new-orleans-now/.

95. Wetzels JFM, Kiemeney LALM, Swinkels DW, Willems HL, Heijer M Den. Age- and gender-specific reference values of estimated GFR in Caucasians: The Nijmegen Biomedical Study. *Kidney Int*. 2007;72(5):632-637. doi:10.1038/sj.ki.5002374.

96. Radin MS. Pitfalls in hemoglobin A1c measurement: When results may be misleading. *J Gen Intern Med*. 2014;29(2):388-394. doi:10.1007/s11606-013-2595-x.

97. Kim PS, Woods C, Georgoff P, et al. A1C underestimates glycemia in HIV infection. *Diabetes Care*. 2009;32(9):1591-1593. doi:10.2337/dc09-0177.

98. Qaseem A, Wilt TJ, Kansagara D, Horwitch C, Barry MJ, Forciea MA. Hemoglobin A1c Targets for Glycemic Control With Pharmacologic Therapy for Nonpregnant Adults With Type 2 Diabetes Mellitus: A Guidance Statement Update From the American College of Physicians. *Ann Intern Med*. 2018;168(8):569-576. doi:10.7326/M17-0939.

99. Sumithran P, Prendergast LA, Delbridge E, et al. Ketosis and appetite-mediating nutrients and hormones after weight loss. *Eur J Clin Nutr*. 2013;67(7):759-764. doi:10.1038/ejcn.2013.90.

100. Gardner CD, Trepanowski JF, Gobbo LCD, et al. Effect of low-fat VS low-carbohydrate diet on 12-month weight loss in overweight adults and the association with genotype pattern or insulin secretion the DIETFITS randomized clinical trial. *JAMA - J Am Med Assoc*. 2018;319(7):667-679. doi:10.1001/jama.2018.0245.

101. Brenner BM, Meyer TW, Hostetter TH. Dietary protein intake and the progressive nature of kidney disease: the role of hemodynamically mediated glomerular injury in the pathogenesis of progressive glomerular sclerosis in aging, renal ablation, and intrinsic renal disease. *N Engl J Med*. 1982;307(11):652-659. doi:10.1056/NEJM198209093071104.

102. Sato J, Kanazawa A, Makita S, et al. A randomized controlled trial of 130 g/day low-carbohydrate diet in type 2 diabetes with poor glycemic control. *Clin Nutr*. 2017;36(4):992-1000. doi:10.1016/j.clnu.2016.07.003.

103. Yancy WS, Foy M, Chalecki AM, et al. A low-carbohydrate, ketogenic diet to treat type 2 diabetes. *Nutr Metab (Lond)*. 2005;2(1):34. doi:10.1186/1743-7075-2-34.

104. Boden G, Sargrad K, Homko C, Mozzoli M, Stein TP. Effect of a low-carbohydrate diet on appetite, blood glucose levels, and insulin resistance in obese patients with type 2 diabetes. *Ann Intern Med*. 2005;142(6). doi:10.1016/S0084-3741(08)70336-6.

105. Stern L, Iqbal N, Seshadri P. The Effects of Low-Carbohydrate versus Conventional Weight Loss Diets in Severely Obese Adults : One-Year Follow-up of a randomized control trial. *Ann Intern …*. 2004;140(May 2001):769-777.

doi:10.1016/j.accreview.2004.07.103.

106. Westman EC, Yancy WS, Edman JS, Tomlin KF, Perkins CE. Effect of 6-month adherence to a very low carbohydrate diet program. *Am J Med*. 2002;113(1):30-36. doi:10.1016/S0002-9343(02)01129-4.

107. Moosheer SM, Waldschütz W, Itariu BK, Brath H, Stulnig TM. A protein-enriched low glycemic index diet with omega-3 polyunsaturated fatty acid supplementation exerts beneficial effects on metabolic control in type 2 diabetes. *Prim Care Diabetes*. 2014;8(4):308-314. doi:10.1016/j.pcd.2014.02.004.

108. Aagaard K, Petrosino J, Keitel W, et al. The Human Microbiome Project strategy for comprehensive sampling of the human microbiome and why it matters. *FASEB J*. 2013;27(3):1012-1022. doi:10.1096/fj.12-220806.

109. McInnes P, Cutting M. Manual of Procedures for Human Microbiome Project. http://www.hmpdacc.org/doc/HMP_MOP_Version12_0_072910.pdf.

110. Marrero DG, Palmer KNB, Phillips EO, Miller-Kovach K, Foster GD, Saha CK. Comparison of commercial and self-initiated weight loss programs in people with prediabetes: A randomized control trial. *Am J Public Health*. 2016;106(5):949-956. doi:10.2105/AJPH.2015.303035.

111. Barengolts E, Manickam B, Eisenberg Y, Akbar A, Kukreja S, Ciubotaru I. EFFECT OF HIGH-DOSE VITAMIN D REPLETION ON GLYCEMIC CONTROL IN AFRICAN-AMERICAN MALES WITH PREDIABETES AND HYPOVITAMINOSIS D. *Endocr Pract*. 2015. doi:10.4158/EP14548.OR.

112. Block G, Azar KMJ, Romanelli RJ, et al. Diabetes prevention and weight loss with a fully automated behavioral intervention by email, web, and mobile phone: A randomized controlled trial among persons with prediabetes. *J Med Internet Res*. 2015. doi:10.2196/jmir.4897.

113. Kramer MK, Molenaar DM, Arena VC, et al. Improving employee health: Evaluation of a worksite lifestyle change program to decrease risk factors for diabetes and cardiovascular disease. *J Occup Environ Med*. 2015. doi:10.1097/JOM.0000000000000350.

114. Parker AR, Byham-Gray L, Denmark R, Winkle PJ. The Effect of Medical Nutrition Therapy by a Registered Dietitian Nutritionist in Patients with Prediabetes Participating in a Randomized Controlled Clinical Research Trial. *J Acad Nutr Diet*. 2014;114(11):1739-1748. doi:10.1016/j.jand.2014.07.020.

115. Maruthur NM, Ma Y, Delahanty LM, et al. Early response to preventive strategies in the diabetes prevention program. *J Gen Intern Med*. 2013;28(12):1629-1636. doi:10.1007/s11606-013-2548-4.

116. Leong A, Daya N, Porneala B, et al. Prediction of Type 2 Diabetes by Hemoglobin A1c in Two Community-Based Cohorts. *Diabetes Care*. 2017;41(1):dc170607. doi:10.2337/dc17-0607.

117. Khaw K-T, Wareham N, Bingham S, Luben R, Welch A, Day N. Association of hemoglobin A1c with cardiovascular disease and mortality in adults: the European prospective investigation into cancer in Norfolk. *Ann Intern Med*. 2004;141(6):413-420. doi:10.7326/0003-4819-141-6-200409210-00006.

118. Cole RE, Boyer KM, Spanbauer SM, Sprague D, Bingham M. Effectiveness of Prediabetes Nutrition Shared Medical Appointments. *Diabetes Educ*. 2013;39(3):344-353. doi:10.1177/0145721713484812.

Low-Carbohydrate Dietary Pattern on Glycemic Outcomes Trial

PROTOCOL

PROTOCOL VERSION 1.7

Dated: October 14, 2019

New Orleans, LA

Table of Contents

1. Overview 5
2. Background and Significance 6
2.A. Type 2 Diabetes Mellitus is a major global public health challenge 6
2.B. Prediabetes is a major risk factor for cardiometabolic diseases 6
2.C. Role of lifestyle in preventing diabetes 6
2.D. Low-to-moderate carbohydrate diets, weight loss, and cardiometabolic diseases 7
2.E. Mediterranean-style diet is associated with reduced risk of T2D and improved glycemic control 8
2.F. Evidence for low-carbohydrate diets that emphasize Mediterranean-style dietary components 8
2.G. Evidence for specific food groups and risk of T2D 8
2.H. Gut microbiota, diet and T2D 9
2.I. Clinical and public health significance of the proposed study 9
2.J. Innovation 9
3. Preliminary Studies 10
3.A. Summary of aggregate experience of the research team 10
3.B. Low-carbohydrate behavioral dietary intervention trial experience 10
3.C. Other dietary intervention trial experience 11
4. Study Design and Organization 11
4.A. Overview of trial design 11
4.B. Study outcomes 12
4.B.1. Primary outcome 12
4.B.2. Secondary outcomes 12
4.C Study Timeline 12
6. Study Participants 13
6.A. Eligibility criteria 13
7. Recruitment and Randomization 13
7.A. Recruitment 13
7.A.1. Mass mailing, placement of brochures/flyers in community, TV/radio/billboard advertisements 14
7.A.2. Database searches and targeted mailing 14
7.A.3. Referrals and Local Partnerships 14
7.B. Randomization 14
7.C. Masking 15

7.D. Safety Considerations 15
8. Dietary Intervention Program 15
8.A. Low-carbohydrate dietary intervention group 15
8.B. Usual Diet Group 16
8.C. Physical Activity 16
8.D. Dietary Adherence 17
9. Safety of Dietary Intervention 17
10. Data Collection 18
10.A. Study visits 18
10.A.1. Screening/baseline visits 18
10.A.2. Follow-up visits and final visit 19
10.B Study measurements 19
10.B.1. Questionnaires 19
10.B.2. Dietary measurements 20
10.B.3. Assessment and management of adverse effects 20
10.B.4. BP and anthropometric measures 20
10.B.5. Blood samples 20
10.B.6. Urine collection 21
10.B.7. Stool Specimen Collection 21
11. Quality Assurance and Quality Control 21
11.A. Manual of procedures 21
11.B. Training and certification 21
12. Sample Size and Statistical Power 22
13. Data Management and Analysis 23
13.A. Data management 23
13.B. Data analysis plan 23
13.B.1. Exploratory data analysis 23
13.B.2. Primary, secondary, and exploratory outcomes 23
15. Anticipated Challenges 24
16. Protection of Human Subjects 24
16.A. Protection of human subjects from research risks 24
16.A.1. Risks to the subjects 24
16.A.2. Adequacy of protection against risks 26

16.A.3. Potential benefits of the proposed research to human subjects and others... 27
16.A.4. Importance of knowledge to be gained ... 27
16.A.5. Remuneration ... 27
16.B. Data safety and monitoring plan ... 27
Bibliography and References Cited ... 30

1. Overview

The increasing prevalence of type 2 diabetes mellitus (T2D), a major cause of morbidity and mortality worldwide, is a critical public health concern. In the United States, an estimated 33.9% of adults have prediabetes (impaired fasting glucose, elevated hemoglobin A1c (HbA1c), or impaired glucose tolerance). Individuals with prediabetes are at elevated risk of T2D and cardiovascular disease (CVD), with 5–10% developing clinical T2D each year.

In the short-term, among patients with T2D, low-to-moderate carbohydrate diets (energy percentage below 45) have a greater glucose-lowering effect than do high-carbohydrate diets, with larger glucose-lowering effect the greater the carbohydrate reduction. Additionally, compared with low-fat diets, Mediterranean diets that are high in unsaturated fats (particularly *cis*-monounsaturated fats) reduce risk of T2D and CVD and improve glycemic control among diabetics. However, compared with usual diets, the effect of a behavioral intervention promoting a low-carbohydrate/high-unsaturated fat and protein dietary pattern that emphasizes consumption of non-starchy vegetables, plant-based oils, fish, poultry, and mixed nuts on glucose homeostasis, and other metabolic risk factors among individuals with prediabetes or early-stage untreated T2D is not well understood.

Our *long-term goal* is to develop and implement dietary intervention(s) that reduce risk of T2D. The *overall goal of this randomized controlled trial is to study the effect of a behavioral intervention promoting a low-carbohydrate/high-unsaturated fat and high-protein dietary pattern (initial target <40 g digestible carbohydrates, final target <60 g digestible carbohydrates) compared with a usual diet on metabolic risk factors among individuals with prediabetes or early-stage untreated diabetes* (HbA1c 6.0–6.9%). Specifically, we will test the following hypotheses:

Primary hypothesis: Compared with usual diet, over a 6-month period, a healthy low-carbohydrate/high unsaturated fat and high-protein dietary pattern will lead to *a larger decrease of HbA1c, by at least 0.15%.*

Secondary hypothesis: Over a 6-month period, a healthy low-carbohydrate/high-unsaturated fat and high-protein dietary pattern will lead to *larger improvements in the following major secondary outcomes*: fasting glucose, systolic blood pressure (BP), total-to-HDL-cholesterol ratio, and body weight.

Exploratory outcomes: We will also study the effect of the intervention on the following outcomes: insulin and homeostasis model assessment of insulin resistance (HOMA-IR); diastolic BP; waist circumference; and estimated CVD risk.

To achieve these study aims, we will recruit 112 adults with prediabetes or untreated diabetes (HbA1c 6.0–6.9%) in the Greater New Orleans, LA area. Using a stratified, blocked randomization scheme, participants will be assigned to either a behavioral modification intervention aimed at promoting a healthy low-carbohydrate dietary pattern or to usual diet. The intervention group will have an intensive phase for 3 months, followed by a maintenance phase in months 4 through 6. The *primary outcome will be difference between the active intervention and control groups in the change in HbA1c from baseline to 6 months of follow-up. We expect 85% power to detect a difference of at least 0.15% in change of HbA1c.* Changes in other metabolic risk factors will be studied concurrently. Metabolic risk factors will be assessed at baseline, 3 months, and 6 months of follow-up.

There is much interest regarding dietary approaches to prevent T2D and its complications. The results from this study will help to determine whether, compared with usual diet, a behavioral intervention aimed at promoting a healthy low-carbohydrate dietary pattern improves metabolic risk factors.

2. Background and Significance

2.A. Type 2 Diabetes Mellitus is a major global public health challenge

Diabetes is a strong and independent risk factor for CVD morbidity and mortality. A meta-analysis of 102 prospective studies (698,782 participants) estimated diabetes leads to an approximately 2-fold increased risk in vascular diseases, including coronary heart disease (CHD) and major stroke subtypes, and deaths due to other vascular causes.[1] Among participants with diabetes, CHD risk is about 50% higher among those with fasting blood glucose ≥7 mmol/L compared with those with fasting blood glucose <7 mmol/L.[1] In addition to increasing CVD risk, diabetes can cause diabetic retinopathy,[2] chronic kidney disease,[3] lower limb amputation,[4] and other complications. Most individuals with diabetes have T2D.[5] The US Centers for Disease Control and Prevention (CDC) estimated that 30.2 million adults ≥18 years (12.2%) had diabetes in 2015, with 7.2 million (23.8%) not being aware of or reporting diabetes.[5] Diagnosed diabetes prevalence among US adults has increased substantially over time, from 2.54% in 1980 to 7.40% in 2015.[6] Diabetes prevalence is also high globally, with an estimated 2014 age-standardized prevalence of 9.0% in men and 7.9% in women.[7] Diabetes accounts for an increasing portion of US healthcare expenditures. Total cost of diagnosed diabetes in the US was estimated to be $245 billion in 2012, 41% higher than in 2007.[8] Diabetes is associated with much higher lifetime medical expenses, with higher expenses for those diagnosed at younger ages.[9]

2.B. Prediabetes is a major risk factor for cardiometabolic diseases

Prediabetes, defined by the American Diabetes Association (ADA) as impaired fasting glucose (IFG), impaired glucose tolerance (IGT), or elevated HbA1c, is also referred to as “intermediate hyperglycemia”[10] and “high-risk state of developing diabetes”.[11] The ADA defines prediabetes as having: fasting plasma glucose of 100–125 mg/dL (IFG); 2-hour post-load glucose in the 75-g oral glucose tolerance test (OGTT) of 140–199 mg/dL (IGT); or HbA1c 5.7–6.4%.[12] Prediabetes itself is not a disease; for all three tests, “risk is continuous, extending below the lower limit of the range and becoming disproportionately greater at the higher end of the range”.[12] The CDC estimated that in 2015, 33.9% of US adults had prediabetes based on fasting glucose or HbA1c.[5] Among those with prediabetes, only 11.9% reported having being told this by a medical professional.[5] Approximately 5–10% of individuals with prediabetes develop diabetes within one year, depending on how prediabetes is defined.[13] In a meta-analysis, annual diabetes incidence was 15–19% among individuals with both IFG and IGT, while it was 4–6% in those with isolated IFG and 6–9% in those with isolated IGT.[13]

A recent meta-analysis (1,611,339 participants from 53 studies) reported that prediabetes as defined by the ADA is associated with higher risk of composite CVD events and CHD.[14] IFG and IGT were also positively associated with increased risk of stroke and all-cause mortality.[14] Prediabetes is linked with increased risk of other conditions typically thought of as diabetes complications, such as neuropathies and nephropathies.[15] Importantly, it is possible for individuals with prediabetes to revert to normoglycemia, as observed in observational studies[13] and lifestyle or drug intervention trials.[16,17] In the Diabetes Prevention Program (DPP), reversion to normoglycemia was associated with lower long-term T2D risk[18] and lower predicted CVD risk.[19]

2.C. Role of lifestyle in preventing diabetes

Much evidence from observational studies and clinical trials supports the critical role of diet and other lifestyle factors in preventing T2D. Globally, lifestyle transitions towards reduced physical activity, increased consumption of refined carbohydrates, coupled with resulting changes in body composition, has led to increases in diabetes prevalence and severity.[20] Body

mass index (BMI), waist circumference, and waist-to-height ratio are predictors of T2D.[21] The DPP, which combined calorie restriction and exercise to promote weight loss, substantially reduced risk of T2D (by 58%) among individuals with elevated FPG and IGT;[22] a benefit that persisted in 10-year follow-up analyses.[16] Other trials of lifestyle modification focused on diet and exercise have also shown benefits in study populations in China,[23] Finland,[24] and India.[25]

2.D. Low-to-moderate carbohydrate diets, weight loss, and cardiometabolic diseases

Low-to-moderate carbohydrate diets (<45% of energy from carbohydrates) are popular dietary approaches for losing weight. Weight loss is a key component of the DPP lifestyle intervention[26] and ADA lifestyle recommendations.[12,27] Meta-analyses from our group[28] and others support that low-to-moderate carbohydrate diets are at least as effective as low-fat diets at promoting weight loss. For instance, a meta-analysis reported a 2-kg (95% CI, 3.1–0.9) larger weight loss in low-carbohydrate diet (<120 g carbohydrates/day) arms compared with low-fat diet (<30% energy from fat) arms.[29] Estimated 10-year CVD risk was lower in low-carbohydrate than low-fat arms.[29] This aligns with research published by our team; in a 12-month trial of participants with obesity, a low-carbohydrate diet (target <40 g carbohydrates/day) was more effective than a low-fat diet (target <30% energy from fat, <7% saturated fat) for weight loss and CVD risk factor reduction.[30]

Low-to-moderate carbohydrate diets may also be beneficial among patients with T2D. Low-carbohydrate diets have come in and out of favor as treatment of diabetes.[31] In a meta-analysis (1376 patients;10 trials), after 3 or 6 months of the intervention, low-to-moderate carbohydrate diets (<45% energy from carbohydrates) led to a 0.34% lower HbA1c (95% CI, 0.06–0.63%) compared with high-carbohydrate diets.[32] Compared with high-carbohydrate diets, there was a greater reduction in HbA1c the greater the reported carbohydrate restriction.[32] There was no benefit of the low-to-moderate carbohydrate diets on HbA1c at 12 months or longer, and no difference between interventions on BMI, body weight, low-density lipoprotein (LDL) cholesterol or quality of life.[32] However, results may have been affected by dietary adherence or glucose medication. There was evidence of diabetes medication reduction in the low-carbohydrate arms in some studies,[33–37] with evidence of medication reduction and improved blood glucose stability persisting for up to 2 years.[38] Hypoglycemia (though not after medication adjustment) was observed in three subjects taking insulin or a sulfonylurea out of twelve subjects in the low-carbohydrate arm of another study.[39] In a subset of participants with T2D (n=46) from a large weight loss trial, compared with those randomized to a low-fat diet and orlistat, those randomized to a low-carbohydrate diet for 48 weeks had improved HbA1c (0.8% lower, 95% CI, -1.5, -0.02%) and a reduction in antiglycemic medications, despite similar weight loss effects of the interventions.[40]

Though low-to-moderate carbohydrate diets have been studied among overweight and obese participants (primarily focused on weight loss), less is understood about the effects of such diets on glycemic outcomes specifically among individuals with prediabetes. A study among participants with IGT observed a reduction at 12 months in weight, HbA1c, and other glycemic outcomes among individuals who participated in a 7-day in-hospital education program, though this was not randomized.[41] A randomized pilot trial of overweight or obese individuals with T2D or prediabetes found that, over 3 months and at 12 months, a very low-carbohydrate diet improved HbA1c, led to higher medication discontinuation, and more weight loss compared with a low-fat diet.[34,37] However, only 4 individuals had prediabetes. Given benefits of low-to-moderate carbohydrate diets for weight loss in general populations and for glycemic control among patients with T2D, further study of these diets are warranted among individuals with prediabetes who are at elevated cardiometabolic disease risk.

2.E. Mediterranean-style diet is associated with reduced risk of T2D and improved glycemic control

Though there is no one "Mediterranean diet", the Mediterranean dietary pattern tends to have the following characteristics: high consumption of vegetables, fruits, breads and cereals, legumes, nuts and seeds; low-to-moderate consumption of dairy products, fish, poultry and eggs; little consumption of red meat and sweets; and red wine in moderation.[42] Olive oil is an important source of fat. In many observational studies, adherence to the Mediterranean-style dietary pattern is associated with reduced risk of CVD[43] and T2D.[44,45] Evidence from randomized trials also supports the benefits of this dietary pattern. In several trials, participants randomized to Mediterranean diets had substantially lower risk of CVD than those randomized to low-fat control diets.[46,47] In the three-arm randomized PREDIMED trial of participants at high risk of CVD, after 4.1 years of follow-up, those randomized to a traditional Mediterranean diet supplemented with either olive oil or nuts had a 30% reduced risk of T2D compared to those in the low-fat control diet group.[48] In all PREDIMED arms, average carbohydrate intake was relatively low (39.7–43.7% at end of trial).[47] Two trials have found a higher rate of remission from metabolic syndrome among individuals randomized to a Mediterranean diet compared with those randomized to control diets (prudent or low-fat) during 2–5 years of follow-up.[49,50] The Mediterranean pattern may also be beneficial among individuals with T2D. Meta-analyses have reported that, among patients with T2D, those randomized to a Mediterranean diet had lower HbA1c levels and CVD risk factors, and more significant weight loss, compared with those randomized to control diets.[51]

2.F. Evidence for low-carbohydrate diets that emphasize Mediterranean-style dietary components

Many low-carbohydrate diets involve increased consumption of food items, such as red meat,[52] that are associated with increased cardiometabolic disease risk. However, low-carbohydrate dietary approaches can focus on substituting carbohydrates with foods common in Mediterranean-style diets, such as olive oil and nuts, which may improve cardiometabolic health. In a 4-year Italian study of individuals with newly diagnosed T2D, those randomized to a low-carbohydrate Mediterranean diet (<50% carbohydrates; consumed ~44%) had more favorable changes in glycemic control, higher rates of T2D remission, and delayed need for antiglycemic drugs compared with participants randomized to a low-fat diet (<30% fat, <10% saturated fat).[53,54] A 12-month Israeli study assessed low-carbohydrate Mediterranean (35% carbohydrates), traditional Mediterranean, and ADA (both 50–55% carbohydrates) diets in 259 overweight participants with T2D.[55] Most CVD risk factors improved in all groups; there was only HDL improvement in the low-carbohydrate Mediterranean diet and this diet was superior to the other diets in improving glycemic control.[55] In a 2-year weight-loss study of moderately obese participants, low-carbohydrate and Mediterranean diets were superior to low-fat diet for weight loss.[56] Participants in the low-carbohydrate arm were counseled to choose vegetarian sources of fat and protein and to avoid trans fat. At 6 years after study commencement, despite weight regain, there were still long-term benefits, particularly for the low-carbohydrate and Mediterranean diets.[57] In whole, evidence supports that low-carbohydrate Mediterranean-style diets are feasible and may have metabolic benefits.

2.G. Evidence for specific food groups and risk of T2D

Prospective cohort studies have found consumption of specific foods and beverages is associated with diabetes risk. Meta-analyses report higher consumption of processed and unprocessed red meat,[58] white rice (particularly in Asian populations),[59] and sugar-sweetened beverages[60,61] are associated with higher T2D risk. Consumption of green leafy vegetables,[62,63] yogurt,[64] whole grains,[65] decaffeinated and total coffee,[66] and light-to-moderate alcohol

consumption[67] are associated with reduced risk. Consumption of fruits, particularly blueberries, grapes, and apples, are associated with reduced risk; fruit juice is associated with higher risk.[68]

2.H. Gut microbiota, diet and T2D

There is increasing evidence for the importance of gut microbiota in risk of type 2 diabetes and other cardiometabolic diseases.[69] Additionally, dietary modifications, including changes in carbohydrate consumption, can lead to substantial changes in gut microbiome over short periods of time.[70,71] Less is known about longer-term changes to the gut microbiota during the adoption of a low-carbohydrate diet and how these changes may influence cardiometabolic risk. We plan to collect stool specimens at baseline, 3 months, and at 6 months, for potential future studies on how this intervention affects the gut microbiome.

2.I. Clinical and public health significance of the proposed study

Prediabetes, or intermediate hyperglycemia, is a marker of high risk of developing T2D and other complications. Due to increasing prevalence of T2D and associated morbidity and mortality globally, a better understanding of dietary approaches to delay progression to T2D or to promote reversion to normoglycemia is a high public health priority. This study proposes to assess the effect of a behavioral intervention promoting a low-carbohydrate/high-unsaturated fat dietary pattern (initial target <40 g digestible carbohydrates, final target <60 g digestible carbohydrates) on HbA1c, a marker of glycemic control, and other metabolic risk factors. Building from prior findings from clinical and observational studies, we will develop a dietary intervention that promotes the consumption of non-starchy vegetables, plant-based oils, fish, poultry, and mixed nuts, that are thought to reduced risk of T2D or CVD. Participants assigned to the intervention will be trained in behavioral techniques that will provide capacity to maintain dietary changes long after study completion. Our outcome measures will assess the effect of this dietary intervention in a “real-life” setting as opposed to dietary modifications achieved in metabolic ward studies or through food prepared in research kitchens. Findings from this study may provide evidence in support of promoting alternative dietary styles among individuals with high T2D and CVD risk and could potentially be used to inform dietary interventions used in programs like DPP.

2.J. Innovation

The status quo as it pertains to dietary approaches for reducing risk of T2D is focused on reducing caloric intake and total fat intake. However, recent evidence suggests that the types of fat, rather than the quantity of fats may be more important, with evidence supporting that a Mediterranean-style diet that does not restrict fat intake reduces risk of developing both T2D[48] and CVD[47]. Moreover, in the short-term, low-carbohydrate diets, which are popular weight loss diets, have a greater glucose-lowering effect than do high-carbohydrate diets among patients with T2D.[32] However, the effect of low-carbohydrate diets that emphasize consumption of non-starchy vegetables, plant-based oils, fish, poultry, and mixed nuts on T2D prevention is unclear, particularly compared with higher-carbohydrate diets more commonly consumed in the US. *The proposed research is innovative, in our opinion, because it represents a substantial departure from the status quo by assessing the combined effect of a low-carbohydrate, high unsaturated fat and protein diet on cardiometabolic risk factors among adults with prediabetes or early-stage untreated diabetes.* We expect the results from this study have the potential to lead to new horizons for developing and implementing dietary approaches (other than the most commonly used reduced fat diet) that will substantially reduce risk of cardiometabolic disease among adults with T2D or at high risk of T2D. The proposed intervention dietary approach promotes consumption of dietary components thought to reduce risk of cardiometabolic disease and the behavior change intervention has expected applicability in clinical practice.

3. Preliminary Studies

3.A. Summary of aggregate experience of the research team

Dr. Kirsten Dorans is trained in cardiovascular disease epidemiology, with strong training in epidemiologic methods and biostatistics. Her publication record includes studies of behavioral risk factors and risk of chronic disease. She has published on the relationships between dietary intake and chronic heart failure[72,73] and on physical activity and incident multiple sclerosis[74]. She served as lead author, conducting data management, analysis, drafting, and revision of two of these manuscripts. In graduate school, she focused on studying links between outdoor air pollution and chronic diseases and has published several papers in this field. The COBRE program will provide an excellent opportunity for the JFI to transition from air pollution research to clinical and translational research focused on nutrition and diabetes. Building off of her training in observational epidemiology, this program will involve training and practical experience that will allow her to broaden her expertise and to develop practical skills for carrying out clinical research.

The mentorship team has much experience and prior successful collaborations in key areas related to this study. Dr. Jiang He is an internationally renowned expert in cardiometabolic disease clinical and epidemiological research. He has served as PI for many epidemiological studies and clinical trials, including clinical and observational studies focused on dietary consumption and chronic diseases[75–80] and has conducted extensive global research on diabetes.[81,82] Dr. Bazzano has collaborated extensively with Dr. He for more than 15 years in research related to diet and chronic disease. She is a clinical investigator and has expertise in nutritional epidemiology and clinical research. Dr. Bazzano was the PI of a highly successful clinical trial comparing the effects of low-carbohydrate and low-fat diets on weight and cardiovascular risk factors among individuals with obesity, which was a subproject within the Tulane COBRE in Hypertension and Renal Biology.[30] Dr. He served as Dr. Bazzano's mentor for this clinical trial. They have collaborated on much other work related to dietary and chronic disease, including a meta-analysis on low-carbohydrate diets.[28] Dr. Bazzano has also published many articles on diet and risk of diabetes[83,84] and on diabetes in the Bogalusa Heart Study.[85–87]

3.B. Low-carbohydrate behavioral dietary intervention trial experience

In the study on macronutrient composition of diet and risk factors for cardiovascular disease (MACRO) study, 148 individuals without clinical cardiovascular disease or diabetes and with obesity were randomly assigned to a low-carbohydrate or a low-fat behavioral dietary intervention for 12 months. In the low-carbohydrate arm, daily dietary intake of carbohydrates decreased from an average of 242 g at baseline to 97 g, 93 g, and 127 g at 3, 6, and 12 months, respectively. At 12 months, participants in the low-carbohydrate intervention group had greater decreases in weight (-3.5 kg; 95% CI, -5.6 to -1.4 kg) and fat mass (-1.5%; 95% CI, -2.6% to -0.4%) and greater improvement in several cardiovascular risk factors.[30] This suggests that restricting carbohydrates may be an option for weight loss and reduction of cardiovascular risk factors. Further analyses found that the low-carbohydrate dietary intervention led to similar or greater improvement in inflammation, adipocyte dysfunction, and endothelial dysfunction compared with the low-fat dietary intervention[88] and that the low-fat diet reduced peptide YY more than the low-carbohydrate diet, suggesting satiety may be better preserved on low-carbohydrate diets.[89] No serious adverse events were reported in this study and the number of participants with symptoms were similar between the two diets, with the exception of more participants having headaches on the low-fat diet at 3 months (18 vs 6 participants, $p = 0.03$).[30] Dr. Bazzano served as PI for this study and Dr. He served as her mentor. Dr. Bazzano

mentored one of her PhD students in conducting analyses and writing and editing manuscripts related to this project.

3.C. Other dietary intervention trial experience

Dr. He has also led or been involved with many dietary intervention trials, including both behavioral change and feeding study interventions. In the Protein and Blood Pressure (ProBP) study, 352 adults with prehypertension or stage 1 hypertension in New Orleans, LA and Jackson, MS were assigned to take, in a random order, 40 g/d soy protein, milk protein, or carbohydrate supplementation each for 8 weeks. Compared with the high-glycemic index refined carbohydrate, both soy protein and milk protein supplementation were associated with a -2.0 mm Hg (95% CI -3.2 to -0.7 mm Hg) and -2.3 mm Hg (-3.7 to -1.0 mm Hg) net change in systolic blood pressure.[80] These findings suggest that partial replacement of carbohydrate with soy or milk protein might be an important part of nutrition intervention strategies to prevent and treat hypertension. Additionally, soy protein supplementation led to reduction in plasma E-selectin compared with carbohydrate and milk protein and in plasma leptin compared with carbohydrate.[90] Soy protein also led to improvement in lipid profile compared with carbohydrate and milk protein.[91] Dr. He was PI for this project.

In the Fiber Intervention on Blood Pressure (FIBRE) study, 110 participants ages 30–65 years with untreated pre-hypertension or stage-1 hypertension and serum cholesterol level < 240 mg/dl were randomly assigned to 8-g/day of water soluble fiber from oat bran or a control intervention (double-blind RCT). Over the 12-week study period, net changes in systolic and diastolic blood pressure were -1.8 mm Hg (-4.3 to 0.8) and -1.2 mm Hg (-3.0 to 0.5 mm Hg), respectively.[78] Net changes in total, HDL-, and LDL-cholesterol were -2.40 mg/dL (-10.60 to 5.81 mg/d:), -1.66 mg/dL (-4.55 to 1.22 mg/dL), and -1.33 mg/dl (-8.33 to 5.68 mg/dL), respectively.[92] Dr He was PI for this study.

Dr. He is currently serving as a mentor for Dr. Katherine Mills' COBRE project (Effect of Dietary Sodium Reduction in Kidney Disease Patients with Albuminuria; SUPER). In this project, participants with chronic kidney disease and albuminuria are being randomized to a behavioral modification program designed to reduce dietary sodium intake to ≤2,300 mg/day or to usual care and test the effect of this intervention on change in albumin-to-creatinine ratio from baseline to 24-weeks.

4. Study Design and Organization

4.A. Overview of trial design

We propose to conduct a *randomized controlled trial* aimed at testing the effect of a behavioral intervention that promotes *reduced carbohydrate intake and increased consumption of heart-healthy unsaturated fats and protein* in patients with HbA1c 6.0–6.9% (*Figure 1*). We plan to recruit 112 individuals with elevated HbA1c and randomly assign them to a moderately intensive intervention on diet pattern,[93] using well-established methods for behavioral modification, or to usual diet. We will compare the difference between the active intervention and control groups for change in HbA1c from baseline to 6 months of follow-up (primary outcome). We will assess four major secondary outcomes: fasting plasma glucose, systolic BP, total-to-HDL cholesterol ratio, and body weight. In

Figure 1. Study Design

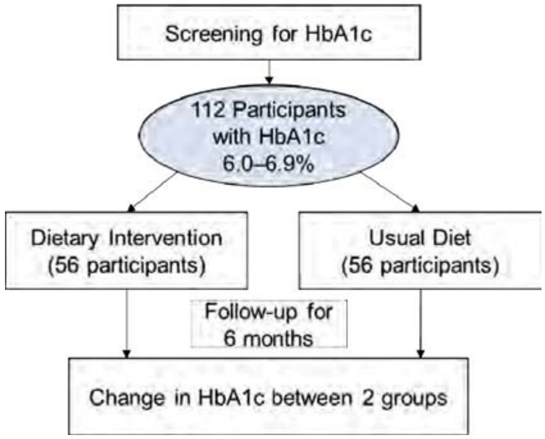

addition, we will look at several other exploratory outcomes: insulin and HOMA-IR, diastolic BP, waist circumference, and estimated CVD risk, as estimated by the 2013 ACC/AHA risk equation. The main trial components are summarized in *Table 1*.

Table 1. Study Overview

Study Design	Randomized controlled trial
Study Participants	112 participants with elevated HbA1c (6.0–6.9%)
Intervention Group (n=56)	Behavioral modification to reduce carbohydrate consumption and promote consumption of foods such as non-starchy vegetables, plant-based unsaturated oils, nuts and seeds, avocados, eggs, seafood, poultry, lean meat and tofu. We will recommend moderate consumption of cheese, unsweetened Greek yogurt, and low-carb milk. If consuming higher-carbohydrate items, recommend legumes, fruits, (limited) whole grains. Target <40 g net carbohydrates per day for first 3 months and will have a goal of <40 net g carbohydrates per day; <60 g net carbohydrates per day for months 4 onwards.
Usual Diet Group (n=56)	No dietary intervention. To encourage continued participation in the trial, we will offer optional monthly educational sessions on unrelated topics, such as retirement planning or health insurance
Outcome Measures	Primary: HbA1c; Secondary: fasting plasma glucose, systolic BP, total-to-HDL-cholesterol ratio, and body weight

4.B. Study outcomes

4.B.1. Primary outcome: The ***primary outcome will be the difference between the intervention group and the usual diet group for change in HbA1c*** from baseline to 6 months. Changes in HbA1c will be calculated from blood samples taken at baseline and 6 months.
4.B.2. Secondary outcomes: Secondary outcomes will include changes from baseline to 6 months of *fasting plasma glucose, systolic BP, total-to-HDL cholesterol ratio, and body weight.,*
4.B.3. Other outcomes: We will also study the effect of the intervention on the following outcomes: insulin and homeostasis model assessment of insulin resistance (HOMA-IR); diastolic BP; waist circumference; total-to-HDL-cholesterol ratio; and estimated CVD risk.

4.C Study Timeline

Table 2 shows the timeline, which will be followed in the implementation of study-related activities, such as publications and submission of an R01 grant application.

Table 2. Study Timeline

	Year 1		Year 2		Year 3	
Develop MOP, intervention program, study forms, and pilot testing	X	X				
Certification and training of staff	X					
Recruitment		X	X			
Intervention and data collection, measurement, entry, cleaning		X	X	X	X	
Data quality control and analysis						X
Publications	X	X	X	X	X	X
R01 grant submissions, resubmissions				X	X	X

The first 6 months will focus on development of manual of operations (MOP), intervention program, study forms, study protocol, Institutional Review Board (IRB) approval, and pilot

testing of the intervention program. It will also involve staff training and certification. During the last half of year 1, recruitment, intervention, and data collection will commence. During the last 3 months of year 3, data quality control and analysis will be carried out. Throughout the study period, the JFI will publish peer-reviewed manuscripts from ongoing Tulane University research projects.

6. Study Participants

6.A. Eligibility criteria

Inclusion criteria

- Men or women ages 40 to 70 years: aim to recruit 50% women
- Any race or ethnicity: aim to recruit 40% ethnic minorities (i.e., African American and Hispanic), close to population in the greater New Orleans area[94]
- HbA1c 6.0–6.9%
- Willing and able to provide informed consent

Exclusion criteria

- Diagnosed type 1 diabetes mellitus based on patient self-report
- Use of agents affecting glycemic control (medications for diabetes, oral glucocorticoids) within the past three months prior to enrollment based on patient self-report
- Medical condition in which low-carbohydrate diet may not be advised
    - Estimated glomerular filtration rate (eGFR) ≤45 mL/min/1.73 m^2, which is close to the 5th percentile of eGFR among non-diseased individuals of 70 years of age[95]
    - Self-report of liver disease due to hepatitis or alcohol; osteoporosis; untreated thyroid disease; gout; or cancer (other than non-melanoma skin cancer) requiring treatment in the past year, unless prognosis is excellent
- Factors that may affect HbA1c:
    - Hemoglobin <11 mg/dL (cutpoint for moderate-to-severe anemia, which could lead to falsely elevated or lowered HbA1c)[96]
    - Recent blood donation or blood transfusion (self-report, past 4 months)
    - Human Immunodeficiency Virus (self-report)[97]
- Allergies to nuts
- For women, current pregnancy, breastfeeding, or plans to become pregnant during the study
- Consumption of ≥21 alcoholic drinks per week or consumption of ≥6 drinks per occasion
- Current or planned residence making it difficult to meet trial requirements (due to distance from study site and/or challenges regularly traveling to site)
- Current participation in another lifestyle intervention trial or a pharmaceutical trial
- Participation of another household member in the study; employees or persons living with employees of the study
- Other concerns regarding ability to meet trial requirements, at the discretion of the principal investigator or study coordinator

7. Recruitment and Randomization

7.A. Recruitment

We will recruit study participants from the greater New Orleans area. The initial focus of our recruitment will be mass mailing of individuals in the greater New Orleans area and recruitment through placing flyers and brochures in the community. If community-based recruitment is not successful or cost-effective, we will recruit out-patients from the Ochsner Health System and

Tulane Medical Center (TMC), from which our group has prior experience recruiting patients. For this recruitment, we will focus on electronic searches of medical records and laboratory databases. We will also carry out targeted solicitation of referrals from physicians who care for patients likely to meet our inclusion and exclusion criteria.

In addition to this recruitment plan, we have several contingency plans: (1) extending recruitment to other medical centers in the New Orleans area, including the Veterans Affairs Medical Center (VAMC), East Jefferson General Hospital, and West Jefferson Medical Center, with whom Tulane has well-established relationships; (2) TV/radio advertisements; (3) arrange a news story about the study in local TV newscasts, radio talk programs, and/or print media; and (4) conduct outreach at local community events and churches. We have ample experience with each of these approaches.

Based on U.S. Census Bureau 2016 population estimates, the metro New Orleans area population was approximately 35% African American or black and 9% Hispanic.[94] Given our prior experience and population distribution in this region, we plan to recruit a distribution of study participants with 40% ethnic minorities.

7.A.1. Mass mailing, placement of brochures/flyers in community, TV/radio/billboard advertisements: Community recruitment approaches include: mass mailing individuals who live in the New Orleans area; brochures/flyers in the community; emailing the brochure; TV, radio, poster, or billboard advertisements in the New Orleans area. Interested individuals will be pre-screened; those at elevated risk for prediabetes or T2D, i.e. individuals who are obese or overweight and/or have a sufficiently high score from the American Diabetes Association's Diabetes Risk Assessment Questionnaire will be invited into the clinic for a screening visit.

7.A.2. Database searches and targeted mailing: We will request IRB approval for a waiver for HIPAA authorization to conduct database searching and hospital chart reviews for identification of potentially eligible study participants. After identifying a potentially eligible patient, we will send this individual an invitation letter signed by the investigators and/or the individual's personal physician and brochure via mail or email. Individuals who express interest in the study will have a pre-screening telephone interview for confirmation of potential eligibility. Those still interested and potentially eligible for enrollment will be scheduled for a visit at the COBRE study clinic.

7.A.3. Referrals and Local Partnerships: In addition to local physician referrals, we will explore holding outreach community events in the New Orleans area. Local community partners may post information about the study on their social media platforms. We may also identify potentially interested volunteers from a Tulane volunteer recruitment registry. We will also consider recruiting potentially eligible individuals who were screened or participated in other clinical studies in our clinic and have expressed interest in being contacted about future research projects. Potential participants referred by their physician or community partners will have a pre-screening telephone interview to confirm potential eligibility. Those who are still interested and potentially eligible for the study will be scheduled for a screening visit.

7.B. Randomization

Once eligibility has been confirmed, the study coordinator will ensure that all key variables have been entered into the trial-specific database. Additionally, the study coordinator will ensure any outliers have been recognized and reconciled, and will ensure receipt of informed consent prior to enrolling the volunteer in the study as a trial participant. Eligible participants will be *randomly assigned to the behavioral intervention or the usual diet group in a 1:1 allocation ratio*. Randomization will be stratified by sex, *using a random block group size (4, 6) to maintain balance among groups over time*. A statistician from the Methodology/Biostatistics Unit within the COBRE Clinical Research Core, who is not directly involved in the study and is not located

at the clinic, will conduct the randomization. After ascertaining eligibility, clinical personnel will telephone the Methodology/Biostatistics Unit to obtain a randomization assignment. All randomization assignments will be created by a computer program and only accessible to the Staff at the Methodology/Biostatistics Unit. Once randomization assignment has been made, trial participants will be considered to be officially randomized into the study and every effort will be made to obtain complete follow-up information.

7.C. Masking

Clinical and laboratory staff members who assess outcomes will be blinded to the intervention assignment of participants. As this is a dietary intervention trial, study participants, and study staff members who conduct the intervention cannot be blinded to the intervention assignment. Participants will be instructed to avoid sharing knowledge of their treatment assignment with data collection staff members. We will have intervention visits and data collection study visits on different days and in separate locations. Data collectors will also use verbal cues and visual props to remind participants not to disclose treatment assignment. Investigators will be blinded to intervention assignment.

7.D. Safety Considerations

Participants with HbA1c >7.9% will be referred to a clinician for follow-up[98], though we will continue to collect data on these participants.

8. Dietary Intervention Program

8.A. Low-carbohydrate dietary intervention group

Participants randomized to the low-carbohydrate intervention group will receive counseling aimed at reducing carbohydrate consumption. Phase I (*Go Low*) will occur for the first 3 months, with a goal of <40 g digestible carbohydrates per day. Key recommended foods will include: non-starchy vegetables, fish, poultry, lean meat, eggs, olive oil and other plant-based unsaturated oils, unsweetened/unsalted nuts and seeds, nut butters, and avocados. We will recommend moderate consumption of cheese and unsweetened Greek yogurt and low-carbohydrate milk. During Phase I, we will recommend limiting or avoiding other dairy, fruits, legumes, beans, and grains. Phase II (*Keep it Low*) will run from months 4 through 6. The net carbohydrate goal for this phase will be <40 g to <60 g, with the objective to choose a target that is as low as possible (e.g., as close to <40 g as possible). At 3 months, we expect different participants will have different levels of intake, with some consuming <40 g of net carbohydrates and others consuming levels higher than 60 g net carbohydrates per day. We have designed the intervention to accommodate various scenarios, with the goal of achieving as low of an intake as possible. For participants who consume >60 g per day, we will recommend they aim to either reduce or maintain carbohydrate intake.

At a brief check-in at 3-months, if participants are satisfied with their current intake, we will instruct them to stay at this intake level (though will recommend they try to reduce intake, if possible, if they are eating >60 net grams of carbohydrates or have not yet reached the study goal of <40 net grams of carbohydrates). If they prefer to make modifications, we will provide guidance on how to modify carbohydrate intake (either decrease or increase). If they choose to add back carbohydrates, we will recommend that participants add 5 grams of carbohydrates for two weeks at a time (e.g., from 40 to 45 g for 2 weeks), for a maximum total of <60 g net carbohydrates. If they choose to further reduce carbohydrate intake, we will provide guidance on this as well. As they modify carbohydrate intake, we will have them monitor their satisfaction

with their diet and energy levels. If adding back carbohydrates, we will recommend participants add back in foods like fruit (preferably berries), legumes, beans, and dairy, while still aiming to keep total carbohydrate intake as low as possible (e.g., as close to <40 net grams as able).

Materials: We will develop a handbook that will be given to participants. This will contain diet guidelines, recipes, meal planners, shopping lists, and guides for counting carbohydrates and reading nutrition labels.

Meals: Participants will select their own menus and prepare or buy their meals according to the guidelines. We will provide participants with supplemental low-carbohydrate foods, such as nuts, olive oil, and other products, to help facilitate their compliance.

Counseling: Sessions and materials will focus on instructing participants to reduce digestible carbohydrate consumption by increasing consumption of protein and fat, with a particular emphasis on increased consumption of heart-healthy unsaturated fats. After randomization, participants will begin the 3-month *Go Low* phase consisting of individual counseling sessions each week for the first four weeks, followed by 4 group sessions held every other week with phone follow-ups occurring in between group sessions. During the *Keep it Low* phase, months 4 through 6, participants will attend 3 monthly group sessions and have 3 telephone follow-ups. There will be a total of 11 in-person sessions and 7 telephone follow-ups over the full study period. Sessions will be approximately one hour and phone calls will be no longer than thirty minutes. These sessions will consist of diet instruction and supportive counseling, addressing results from 24-hour dietary recalls, teaching self-monitoring and individual diet goal changes, and provide suggestions for replacing usual foods containing carbohydrates. Study materials will be modified from materials used in the successful MACRO study. The JFI will work closely with Drs. He and Bazzano to develop the intervention based on the MACRO study.

Carbohydrate goal: In the MACRO study, carried out among individuals from the same study region, a behavioral intervention aimed at reducing digestible carbohydrate intake to <40 g/day led to reductions in weight and improvements in cardiovascular risk factors among individuals with obesity.[30] Additionally, restriction of carbohydrate intake to <50g/day is sufficient to induce ketosis.[99] Our rationale for having a multi-phased intervention is to allow participants to see how they feel on the more restrictive diet, and see how they respond to small increases in carbohydrate consumption. A similar technique was applied in the DIETFITS published study.[100] The higher target of 60 net grams may be more sustainable long-term and also allows for consumption of healthy higher-carbohydrate foods, such as berries, legumes, beans, and limited whole grains.

8.B. Usual Diet Group

We will provide participants randomized to the usual diet group written information with standard dietary advice. Participants randomized to the usual diet group will not receive ongoing dietary recommendations. Moreover, they will continue to receive clinical care recommendations from their physician(s). To encourage continued participation in the study and minimize the difference in participant contact with study staff, participants will be offered optional monthly educational sessions on topics unrelated to the study intervention, such as retirement planning or health insurance options.

In another effort to encourage continued participation, patients randomized to the usual diet group will be offered a copy of all available intervention materials, as well as an optional one-hour counseling session with the interventionist following their final study visit.

8.C. Physical Activity

At baseline, both groups will receive written information in study materials with standard physical activity recommendations.

8.D. Dietary Adherence

Strategies for measuring compliance: For the low-carbohydrate diet, adherence will be assessed through measurement of urinary ketones at 3-month visit and 6-month TV. Study staff will make all reasonable effort to maintain adherence of participants to the visit schedule and diet intervention. Walnut consumption will be tracked throughout the study in the low-carbohydrate diet group.

Strategies for improving compliance:

- At the screening visit, we will ask participants who does most of the cooking in their households and how often they eat outside of the home. Results from the screening visit will help to select a group of cooperative study participants.
- Visits will be scheduled at the participants' convenience and to minimize waiting time and trips to the clinic; reminder calls, texts or emails (chosen based on participant preference) will also be made prior to clinic visits.
- We will hire enthusiastic staff who have good interpersonal skills.
- Participants will receive $40 gift cards for use at food stores or retailers when they complete each study visit, for a maximum of $120 over the study period.
- Participants will receive $25 gift cards when they return the stool specimens, for a maximum of $75 over the study period.
- Participants will be offered small gifts (such as pens or magnets with study logo) to encourage compliance and adherence to the study protocol.
- Participants will be provided with recipes, shopping lists, and food samples for the intervention diet.

9. Safety of Dietary Intervention

On the low-carbohydrate diet, participants will be encouraged to primarily focus on replacing carbohydrates (particularly highly refined carbohydrates) with foods such as non-starchy vegetables, olive oil and other plant-based unsaturated oils, nuts and seeds, avocados, eggs, seafood, tofu, poultry, and lean meat. Consumption of more unsaturated fats, protein, and fiber will be recommended. Participants will not be encouraged to increase consumption of saturated fats and will be advised to avoid consumption of trans fats.

In animal studies, a high-protein diet has decreased renal function[101] In the MACRO study, on average, participants tended to increase consumption of fats, rather than protein.[30] The safety and tolerability of low-carbohydrate diets have previously been assessed in many studies, including MACRO.[30] A number of studies, including some in individuals with T2D, have found no clinically significant increases in estimated glomerular filtration rates,[33,38–40] microalbuminuria,[102] albumin excretion rate,[33,38] urinary albumin,[38] serum creatinine,[38,40,102–107] albumin,[40] or uric acid levels.[103–106] A small study of a very low-carbohydrate diet found significant increase in uric acid excretion, but no significant change in 24-hour urine creatinine clearance.[106]

Study staff will carefully monitor participants' BP, and HbA1c more often than what would occur in routine healthcare visits. A Data Safety and Monitoring Board (DSMB) consisting of an independent group of experts in areas such as biostatistics, nutrition medicine, general internal medicine, and endocrinology has been formed. The board will regularly review trial progress, including unblinded interim results, and can recommend that the trial prematurely end if participants are at risk or if it becomes clear that the trial will not be able to yield statistically significant results.

10. Data Collection

10.A. Study visits

After the initial pre-screening interview, in which data on medications and medical conditions will be collected, there will be one screening visit, one randomization visit, a follow-up study visit at 3 months and a termination visit (TV) at 6 months. *Table 3* summarizes study procedures and data collected at each study visit.

Table 3. Study visit schedule and data collection

	Pre-screen	SV	RZ	3 month	6 month TV
Pre-screen eligibility	X				
Informed consent		X			
Compliance form		X			
Eligibility confirmation			X		
Randomization			X		
Demographics		X			
Medical history questionnaire			X	X	X
Medications		X	X	X	X
Lifestyle (alcohol, cigarette smoking)			X	X	X
Physical activity			X	X	X
24-hour diet recall*			X	X	X
3 BP measurements		X	X	X	X
Body weight			X	X	X
Height			X		
Waist circumference			X	X	X
Spot urine test			X	X	X
Blood samples		X	X	X	X
Stool specimen			X	X	X
Side-effects assessment			X	X	X
Nut Consumption					X

*Two 24-hour dietary recalls will be collected; one should cover a weekend and the other a weekday.

10.A.1. Screening/baseline visits:

Participants will be pre-screened by phone or in-person by study staff to confirm eligibility for the Screening Visit. An approved questionnaire will be used to ensure the patient is eligible to attend the in-clinic screening visit, and the patient will be scheduled thereafter.

At the screening visit (SV), participants will provide informed consent for the screening and main study. Study staff will schedule the SV and randomization (RZ) visit at least 2 days apart. If a participant is eligible based on the screening visit, he or she should be scheduled for the RZ

once a sufficient group of eligible individuals has been recruited. Participants should be scheduled for the RZ at least 2 days and no more than two weeks after the screening visit.

At the screening visit, we will collect information on inclusion/exclusion criteria, medication use, and demographics. At this visit, we will also have participants complete a compliance form, to assess their ability to complete the study and attend all intervention and study visits. We will also measure blood pressure, and take blood samples. Women will be asked if they are postmenopausal or if they had surgical sterilization (such as a tubal ligation). If not, they will be tested for pregnancy through a spot urine test; pregnant women will be excluded from the study. Blood samples will be collected for measurement of HbA1c, and, if not available from medical records in the past 3 months, serum creatinine and hemoglobin. These blood samples will be used for excluding individuals with HbA1c <6.0 or >6.9, eGFR ≤45 mL/min/1.73 m^2, or hemoglobin <11 mg/dL.

Before the start of the intervention, participants will complete two 24-hour dietary recalls. At least one (and potentially both) 24-h recalls will be collected by phone. At the RZ, participants will learn about the randomization process. After requirements for eligibility have been satisfied, participants will formally be enrolled into the study and randomized to either the active intervention or control group. We will collect fasting blood samples for later measurement of glucose, insulin and lipids and other potential future measurements. At this visit, we will measure blood pressure, body weight, height, and waist circumference. We will collect information on medical history, lifestyle risk factors, and physical activity. A spot urine sample will be collected to measure ketones and other markers and participants will be asked to collect a stool specimen for potential use in future microbiome studies.

10.A.2. Follow-up visits and final visit: A follow-up visit will occur at 3 months after randomization. A final visit will occur at 6-months post-randomization. These visits should be scheduled as close as possible to the 3-, or 6-months after randomization. At each visit, study staff will conduct dietary recalls and anthropometric measures, take 3 BP measurements, collect a fasting blood sample to measure HbA1c. At least one (and potentially both) 24-h recalls will be collected by phone. We will also collect samples for later measurement of glucose, insulin, lipid profile, and other potential future measurements. A spot urine sample will be collected to measure ketones and other markers. Participants will be asked to collect a stool specimen for potential use in future microbiome studies. Information about participants' nut consumption over the past 6 months will be collected at the 6-month TV. At study termination, we will mail participants and/or their physicians a summary of trial results and a record of participant BP, anthropometric measures, and laboratory data from the trial period. Throughout the study period, we will provide participants with their blood pressure and weight. We will request that, if possible, a participant who is prescribed a diabetes medication during the course of the study comes into the clinic for an additional separate supplemental visit for a fasting blood sample collection prior to starting the medication. Maintaining follow-up of all randomized participants is of utmost importance to ensure data integrity. In the event that a participant moves during the study we may request that the participant provides a fasting blood sample at a local LabCorp facility (this will be described on the informed consent form).

10.B Study measurements

Measurements obtained for this study will be based on those used in other clinical trials and epidemiologic studies. We are familiar with strengths and limitations of such measurements and the importance of staff training and certification and use of stringent quality control procedures.

10.B.1. Questionnaires: Study staff will administer a *medical history questionnaire* at the RZ, 3-month visit and 6-month TV to assess medical conditions that would necessitate changes in

therapy or medical management beyond this study's scope. At the SV, *demographic factors* will be collected. At the SV, RZ, 3-month and 6-month TV, we will also administer *medication tracking questionnaires*. At RZ, 3-month, and 6-month TV, we will collect health behavior and physical activity data.

10.B.2. Dietary measurements: Two 24-hour dietary recalls will be obtained from participants at baseline. At or close to both the 3-month and 6-month TV, two more 24-hour dietary recalls will be collected. The second sets of two recalls will be collected as closely as possible to 3- or 6-month dates after randomization, with a goal of being within 2 weeks of the respective follow-up period. For each time point, one recall should reflect week day consumption, while the other should reflect weekend day consumption and the two recalls should be at least 2 days apart from each other. We will use computer software (Nutrition Data System for Research [NDS-R]) and food models used in previous and ongoing studies. Recalls will be conducted by a study staff member who has been certified in the use of NDS-R. Food composition tables of the NDS-R will be used to calculate dietary nutrient intakes. The baseline dietary assessment will be done to characterize individual dietary intake of carbohydrates and other macronutrients. During the intervention, repeated measurements will be used to monitor short-term changes in mean dietary nutrient intake between the intervention and usual diet groups.

10.B.3. Assessment and management of adverse effects: At RZ, 3-month visit, and 6-month TV, we will administer *symptom questionnaires*, which will consist of a checklist to identify participant complaints. Prior studies of low-carbohydrate dietary interventions suggest that participants may experience a range of adverse effects, including constipation, halitosis, fatigue, headache, thirst, polyuria, and nausea. Severe adverse events will be promptly reported to the DSMB. Management of adverse effects will be based on the protection and safety of the participant. If severe adverse effects are reported by a participant, carbohydrate intake will be examined and altered in order to mitigate the adverse effect. In any extreme cases, participants will be instructed to stop the intervention. As this is a behavioral intervention, adverse effects should be rare.

10.B.4. BP and anthropometric measures: BP will be measured 3 times at the screening visit and at each follow-up visit. To minimize random error, averages of the measurements at the screening and randomization visits will be taken. BP will be measured by a standard automated blood pressure measurement device (OMRON HEM-907 XL Professional Digital Blood Pressure Monitor) and performed by experienced staff, trained and certified in BP measurement. Prior to BP measurements, participants will be asked to rest quietly in a seated position with their feet supported for at least 5 minutes; staff members will use an appropriate cuff size over a bare arm. Trained and certified staff will measure weight using a calibrated balance beam scale at the screening visits, and follow-up visits. Weight and height will be measured at the RZ, and weight will also be measured at the subsequent visits.

10.B.5. Blood samples: For SV, participants will not need to fast. For RZ, 3-month visit, and 6-month TV, participants will be instructed to fast for 12 hours prior to blood sample collection. At SV, blood samples will be collected to measure HbA1c, hemoglobin, and serum creatinine. At RZ, fasting blood samples will be collected and stored as serum/plasma, whole blood aliquots and buffy coats at -80°C for future analyses, including measurement of glucose, insulin and lipids, and potential future metabolomics or genetic analyses. At 3-month visit and 6-month TV, we will collect fasting blood samples to measure HbA1c and will also store additional serum/plasma, whole blood aliquots and buffy coats at -80°C for future analyses, including measurement of glucose, insulin and lipids, and potential future metabolomics or genetic analyses. Details of the blood sample collection are shown in *Table 4*. In the event that a participant moves, we may request that they collect a fasting blood sample at a local LabCorp and we will measure HbA1c, glucose, insulin, and lipids at the 3 and 6-month visits. HOMA-IR will be calculated from fasting glucose and insulin.

Table 4. Study visit schedule for specimen collection

	SV	RZ	3 month	6 month TV
HbA1c	X		X	X
Serum creatinine	X			
Hemoglobin	X			
Storage of aliquots and buffy coats (for measurement of glucose, insulin, lipids, other potential future measurements)		X	X	X
Urine sample for pregnancy	X			
Urine sample for ketones and other markers		X	X	X
Stool sample		X	X	X

10.B.6. Urine collection: Study staff will collect spot urine specimens at RZ, interim visit, and the final visit to measure ketones.

10.B.7. Stool Specimen Collection: Stool samples will be collected from all eligible and willing trial participants during the RZ, 3-month, and 6-month TV, following the protocol set forth by the Human Microbiome Project.[108,109] Participants will be given a stool collection kit, which includes the specimen collection container, a shipper for home collection, and a form to record the timing and pertinent information related to specimen collection. Participants will be instructed to return the kit to the clinic within two days of collection (in-person or via shipping), at which time stool samples are stored at -80°C until analyses. Kits and instructions for collection will be reviewed by study staff with the participant at the required visits.

11. Quality Assurance and Quality Control

Throughout the study, we will implement rigorous quality assurance (QA) and quality control (QC) measures. We describe some of these approaches here.

11.A. Manual of procedures

We will develop a manual of procedures (MOP) based on the MOP successfully used in the MACRO behavior change clinical trial. The MOP will provide instructions and describe procedures for staff training and certification, participant recruitment, intervention counseling, study form completion and procedures, data entry, safety measures (e.g., methods for emergency participant/primary care physician contact).

11.B. Training and certification

Trial personnel will all be required to participate in a training session before the start of any trial procedures or measurements. Training will include particular attention to core trial elements, such as recruitment, retention, intervention, completeness and quality of data

acquisition (e.g., blinding procedures for the data collectors), and data entry. Separately, intervention staff will meet and receive detailed instruction on trial approaches to group and individual behavior change counseling and procedures that ensure data collectors are blinded to treatment assignment. Throughout the trial, periodic re-training/certification sessions will be conducted to ensure all trial-related procedures are being applied in a standard manner. Investigators will also monitor all aspects of study performance to ensure study goal achievement, timeliness and completeness of study visits, completeness and quality of data collection, intervention adherence, and adequacy of the procedures separating data collection from intervention. Study staff and investigators will pay particularly careful attention to the quality of all laboratory measurements, particularly key exposure, outcome, and intervention monitoring variables.

12. Sample Size and Statistical Power

The primary outcome will be the difference in the change of HbA1c between the intervention and usual diet groups from baseline to 6 months of follow-up. In the United States National Health and Nutrition Examination Survey, when sub-setting to the range of HbA1c of 6.0–6.9% among individuals who do not report use of diabetes medication, the cross-sectional standard deviation is 0.24% using data from 2000 through 2016 (n=4117; https://wwwn.cdc.gov/nchs/nhanes/). In studies with 6-month follow-up among individuals with HbA1c 5.7 to 6.4% or other characteristic(s) (e.g, elevated fasting glucose or metabolic syndrome) that put them at elevated risk of type 2 diabetes, the within-person correlation between baseline and follow-up HbA1c was estimated to be between 0.5 and 0.84.[110–113] As all participants in our study will be within a specific range of HbA1c at baseline, we expect this may lead to smaller within-person correlation of HbA1c over time than studies that do not restrict to a narrow range of HbA1c at baseline. Assuming a within-person correlation of 0.5 and an SD of 0.24% for change in HbA1c, we would need to have 90 individuals to have 85% power to detect an approximate difference in change of HbA1c of 0.15% between the intervention and usual diet groups.

In a follow-up analysis of the DPP, a 0.15% decrement in HbA1c at 6 months was associated with a 16% lower risk of incident T2D, adjusting for treatment arm, age, sex, race/ethnicity, household income, and baseline HbA1c.[115] In a pooled analysis from the Atherosclerosis Risk in Communities and Framingham Heart Studies, a 0.15% higher HbA1c was associated with an 16% higher risk of T2D over 20 years, a 30% higher risk over 8 years, and a 13% higher risk after 8 years, after adjusting for age, sex, fasting glucose, triglycerides, HDL, family history of T2D, SBP, and BMI.[116] Additionally, in EPIC-Norfork, a difference of HbA1c of 0.15% was associated with approximately a 5% higher risk of CHD among those free of CHD, stroke, diabetes, and with HbA1c <7.0%.[117]

Table 5. Minimum detectable differences

	SD	Detectable Difference
HbA1c, %	0.24	0.15
Fasting glucose, mg/dL	12.5	8.0
Fasting insulin, mU/mL	7.0	4.5
HOMA-IR	2.0	1.3
SBP (mmHg)	4.2	2.7
DBP (mmHg)	3.0	1.9
Total/HDL ratio	1.2	0.77
Body weight (kg)	6.6	4.2
Waist circumference (cm)	8.0	5.1
Estimated CVD risk (%)*	6.7	4.8

*10-y CVD risk, among estimated 85% or 76 participants free of CVD

In the DPP study, HbA1c was 0.15% lower among those in the lifestyle intervention arm than in the control arm at 6-months follow-up; HbA1c at study commencement was 5.9% and levels increased in the control arm and decreased in the behavioral intervention arm.[22] In a small behavioral intervention study (n=34) of a very low-carbohydrate ketogenic diet compared

with a moderate carbohydrate, calorie-restricted, low-fat diet among individuals with type 2 diabetes or prediabetes (HbA1c >6.0%) and elevated body weight (BMI >25), mean HbA1c decreased from 6.6% to 6.1% in the very low-carbohydrate group and from 6.9% to 6.7% in the moderate carbohydrate group over a 12-month period; additionally, the very low-carbohydrate group had greater reductions in glucose-lowering medications.[37]

Detectable differences are listed for HbA1c and secondary outcomes in *Table 5*, based on data from prior studies.[30,53,56,118] These were calculated for unpaired t-tests, power of 85%, two-sided alpha of 0.05, and assuming 80% follow-up of 112 participants, which would yield an estimated 90 participants.

13. Data Management and Analysis

13.A. Data management

Personnel will double-enter all study data. Prior to entering data, the study coordinator will conduct quality control on all study forms, study measures, and laboratory data. Data will be cleaned and logically checked.

13.B. Data analysis plan

The data will be analyzed using *intention to treat*; this involves comparing the primary and secondary outcomes between participants based on randomization assignment.

13.B.1. Exploratory data analysis: We will express group data for variables with a normal distribution as mean ± standard deviation and for variables not normally distributed as median and interquartile ranges (or will log-transform the variables to obtain a normal distribution). We will compare means and percentages of baseline variables across the intervention and usual diet group to assess similarity of these groups at randomization.

13.B.2. Primary, secondary, and exploratory outcomes: We will test the primary study hypothesis that there is a greater reduction in HbA1c from baseline to 6 months in the intervention group than in the usual diet group. To do this, we will use a mixed-effects linear regression model, to test the null hypothesis that there is no difference in change between baseline and 6-month HbA1c for the intervention group compared with the usual diet group (Specific Aim 1). To do this, we will use the following model:

$$Y_{i,j,k}|T = b_0 + b_1G_i + b_2T_{3\text{-months}} + b_3T_{6\text{-months}} + b_4G_iT_{3\text{-months}} + b_5G_iT_{6\text{-months}} + a_{0i,j} + e_{i,j,k}$$

where $Y_{i,j,k}$ is HbA1c for the jth participant (P) nested in the ith intervention group (G) at time T_k (baseline, 3 months, and 6 months). This model includes a random intercept ($a_{0i,j}$) and error term $e_{i,j,k}$. The interaction term ($b_5G_iT_{6\text{-months}}$) allows for an interaction between group and $T_{6\text{-months}}$, which is equal to 0 for baseline and 3-months and 1 for 6-months. Rejection of the null hypothesis (b_5=0) indicates there is a significant difference in change of HbA1c from baseline to 6 months between the intervention and control groups. In this model, participants are assumed to be random effects and intervention group, time, and the interaction terms are fixed effects.

We will use a similar approach to test the difference in the change between baseline and 6-month values of secondary outcomes (Specific Aim 2) and exploratory outcomes. In exploratory analyses, we will also test the difference in the change between baseline and 3-month values of outcomes (HbA1c, fasting glucose, systolic blood pressure, total-to-HDL cholesterol, body weight, insulin, HOMA-IR, diastolic blood pressure, waist circumference, and estimated CVD risk). We will also explore the use of mixed effects models to assess overall net change in HbA1c (by treating time as a linear variable) and other outcomes over the full study period. For each outcome, we will test modeling assumptions and will consider transformation of outcomes, if needed, to meet modeling assumptions. As the mixed-effects model assumes missing at

random, we will also perform sensitivity analyses using Markov-chain Monte Carlo techniques to impute missing values of the outcomes, using covariates for this imputation and will compare results from this analysis with our primary findings.

15. Anticipated Challenges

Recruitment: Many clinical trials have challenges recruiting sufficient numbers of volunteers who meet the trial inclusion and exclusion criteria and demonstrate a high level of adherence to trial protocol requirements. Fortunately, the investigators are very experienced with clinical trial recruitment, and have several contingency options that can be used to meet unanticipated shortfall in recruitment.

Intervention: The investigators are aware of and understand challenges related to achieving and maintaining compliance with a behavioral intervention. The investigators have much experience and success in behavioral interventions, with recent success of a behavioral intervention aimed at reducing carbohydrate intake among participants from the New Orleans area. The JFI will collaborate with Drs. He and Bazzano to develop the intervention program based on the MACRO study and will assess potential barriers to implementation and intervention adherence (such as financial concerns and range of dietary patterns of study participants) during the planning and pilot phase of the study.

Adherence: Though adherence and follow-up are also main challenges related to clinical trials, the investigators have an outstanding track record for studies that have recruited participants for both clinical and epidemiology studies in the Greater New Orleans area. To encourage adherence, small gifts (such as pens or magnets with study logo) will be offered to all study participants.

16. Protection of Human Subjects

This Human Subjects Research meets the definition of a clinical trial. All investigators will be required to complete the CITI (Collaborative Institutional Training Initiative) program in the ethical use of human participants in research.

16.A. Protection of human subjects from research risks

16.A.1. Risks to the subjects

Human subject involvement and characteristics: We expect to enroll 112 participants, with an equal male to female ratio. We expect to enroll approximately 30 participants who are African-Americans, and another 10 with mixed racial backgrounds, and a few Asian participants given the demographics of the greater New Orleans area. Overall, given the demographics of greater New Orleans, we estimate approximately 10% participants of Hispanic or Latino ethnicity, 50% of them with African-American racial background.

Inclusion criteria

- Men or women ages 40 to 70 years: aim to recruit 50% women
- Any race or ethnicity: aim to recruit 40% ethnic minorities (i.e., African American and Hispanic), close to population in the greater New Orleans area[94]
- HbA1c 6.0–6.9%
- Willing and able to provide informed consent

Exclusion criteria

- Diagnosed type 1 diabetes mellitus based on patient self-report
- Use of agents affecting glycemic control (medications for diabetes, oral glucocorticoids) within the past three months prior to enrollment based on patient self-report
- Medical condition in which low-carbohydrate diet may not be advised
    - Estimated glomerular filtration rate (eGFR) ≤45 mL/min/1.73 m², which is close to the 5th percentile of eGFR among non-diseased individuals of 70 years of age[95]
    - Self-report of liver disease due to hepatitis or alcohol; osteoporosis; untreated thyroid disease; gout; or cancer (other than non-melanoma skin cancer) requiring treatment in the past year, unless prognosis is excellent
- Factors that may affect HbA1c:
    - Hemoglobin <11 mg/dL (cutpoint for moderate-to-severe anemia, which could lead to falsely elevated or lowered HbA1c)[96]
    - Recent blood donation or blood transfusion (self-report, past 4 months)
    - Human Immunodeficiency Virus (self-report)[97]
- Allergies to nuts
- For women, current pregnancy, breastfeeding, or plans to become pregnant during the study
- Consumption of ≥21 alcoholic drinks per week or consumption of ≥6 drinks per occasion
- Current or planned residence making it difficult to meet trial requirements (due to distance from study site and/or challenges regularly traveling to site)
- Current participation in another lifestyle intervention trial or a pharmaceutical trial
- Participation of another household member in the study; employees or persons living with employees of the study
- Other concerns regarding ability to meet trial requirements, at the discretion of the principal investigator or study coordinator

Sources of materials: Medical information data will be collected from the participant and, after written authorization for the disclosure of protected health information, from participants' medical records. Blood and urine samples will be collected to measure HbA1c, glucose, insulin, lipids, creatinine, ketones, and test for pregnancy. Blood pressure, height, weight, waist circumference and other physical measurements will be obtained.

Potential Risks: Risks associated with venipuncture, the diet intervention, and collection of confidential medical information are the main risks associated with this study. There is a small risk of bruising and a rare chance of local infection associated with standard venipuncture to collect blood samples. The dietary intervention has minimal risks. Reduced carbohydrate consumption may lead to adverse effects, including constipation, halitosis, fatigue, headache, thirst, polyuria, and nausea. At RZ, 3- , and 6-month visits, we will administer *symptom questionnaires*, which will consist of a checklist to identify participant complaints. Adverse effects of the low-carbohydrate diet will be assessed at counseling sessions. Severe adverse events will be promptly reported to the DSMB. Management of adverse effects will be based on the protection and safety of the participant. If severe adverse effects are reported by a participant, carbohydrate intake will be examined and altered in order to mitigate the adverse effect. In any extreme cases, participants will be instructed to stop the intervention. Furthermore, there is a potential risk of unauthorized disclosure of medical information. This risk will be minimized by using a strict protocol for data processing and by the appropriate training of all study personnel. Protection against risks is described in more detail below.

16.A.2. Adequacy of protection against risks

Recruitment and Informed Consent: The proposed study will be conducted following strict guidelines for the protection of the rights of human volunteers. The informed consent document will be signed by all participants at the screening/baseline visit. The informed consent form will be developed in the first year of the study and approved by the IRB. The consent form will clearly state the purpose, eligibility criteria, and protocol of the study, the potential benefits and risks of participation, and the subject's right to refuse to participate or withdraw. Prior to signing the informed consent, research staff will review the details of the consent form orally with the participant, and answer any questions that the participant has concerning participation in the study. In the case of potential participants with low or no literacy, the informed consent form will be read to the participant in its entirety and any questions will be answered prior to the participant signing or marking the form. Specifically, the following must be accomplished during the informed consent process:

- The participant must be informed that participation in the study is voluntary and that refusal to participate will involve no penalty or loss of benefits.
- The participant must be informed of the purpose of the study and that it involves research.
- The participant must be informed of any alternative procedures, if applicable.
- The participant must be informed of any reasonably foreseeable risks.
- The participant must be informed of any benefits from the research.
- An outline of safeguards to protect participant confidentiality will be included, as well as an indication of the participant’s right to withdraw without penalty. This should be balanced with a discussion of the effect that withdrawals will have on the study, and the responsibility a participant has, within limits, to continue in the study if he or she decides to enroll.
- The participant must be informed of his or her right to have questions answered at any time and whom to contact for answers or in the event of research-related injury.
- The participant must be informed as to whether or not compensation is offered for participation in the study and/or in the event of a medical injury.
- The participant must be informed that he/she will be notified of any significant changes in the protocol that might affect their willingness to continue in the study.

Written authorization for the disclosure of protected health information will be obtained during the consent process on a separate form (HIPPA authorization), if applicable.

Protection against Risk: The protocol and informed consent will be approved by the Institutional Review Boards at Tulane University Health Sciences Center and any other participating institution for recruitment. The study participants will be given contact information for the Study Coordinator and Study PI to answer questions or to report complaints. Patients can withdraw from the study at any time without any effect on their medical care. Standard techniques will be used by trained, certified phlebotomists or study nurses for blood sample collection at the study clinics where emergency medical equipment is available. Confidentiality of all data collected during the study will be maintained through the use of unique, encrypted study identification (ID numbers, rather than patient names, in the study database). These encrypted ID numbers will not be mathematical derivation of the subject’s medical record number or any other patient identifier. No information will be disclosed in an individually identifiable form in any type of presentation or publication. There will be password-required access to the computerized file linking the study numbers to patient identifiers at the study clinics. Password-access will be restricted to pertinent research staff only. All study data will be

kept in locked file cabinets in locked rooms, accessible only by study staff with permission. All data transmissions will use HIPPA-compliant password protected system and will be secured through a virtual private network.

16.A.3. Potential benefits of the proposed research to human subjects and others

Study participants may have improved glycemic outcomes, blood pressure, lipid levels, may lose weight and reduce waist circumference, and may have improvement in other risk factors for CVD and development and progression of T2D. Participants will receive multiple blood pressure, weight, waist circumference, and blood tests. If vital signs or lab values are clinically abnormal, participants will be referred to their primary care physician, or if they do not have a primary care physician, one will be assigned through the Tulane University Community Health Center, which is a part of the core clinical facilities. At the end of the study, we will provide the participants' primary care physician with a copy of all the blood pressure, weight, and laboratory results.

16.A.4. Importance of knowledge to be gained

Information from this study may contribute to reducing the onset of T2D and the burden of T2D and cardiovascular disease in Louisiana and the general US population. The study has potential for public health impact; results from this study may provide evidence in support of promoting alternative dietary styles (than the most commonly used low-fat diet) among individuals with high T2D risk or with T2D.

16.A.5. Remuneration

For screening visit 2, the two follow-up visits, and the final visits, participants will receive $40 gift cards for use at food stores or retailers (after completing each study visit), for a maximum of $120 over the study period. Participants who return a stool sample will receive $25 gift cards for each stool sample that they return, for a total of $75 for three stool samples. Participants will also receive free parking for each intervention and study visit.

16.B. Data safety and monitoring plan

A DSMB, which consists of 3 experts who are not otherwise affiliated with the COBRE program, has been formed. Study investigators for this study will prepare accurate and timely data tables and reports for the DSMB meetings. The members of the DSMB will serve in an individual capacity and provide their expertise and recommendations. The primary responsibilities of the DSMB are: 1) periodically review and evaluate the accumulated study data for participant safety, study conduct and progress, and, when appropriate, efficacy, and 2) make recommendations to the NIH concerning the continuation, modification, or termination of the trial. The DSMB considers study-specific data, as well as relevant background knowledge, about the disease, test agent, and patient population under study.

The DSMB is responsible for defining its deliberative processes, including event triggers that would call for an unscheduled review, stopping guidelines, unmasking (unblinding) and voting procedures prior to initiating any data review. The DSMB is also responsible for maintaining the confidentiality of its internal discussions and activities, as well as the contents of reports provided to it.

The DSMB will review each protocol for any major concerns prior to implementation. During the trial, the DSMB should review cumulative study data to evaluate safety, study conduct, and scientific validity and integrity of the trial. As part of this responsibility, DSMB members must be satisfied that the timeliness, completeness, and accuracy of the data submitted to them for

review are sufficient for evaluation of the safety and welfare of study participants. The DSMB should also assess the performance of overall study operations and any other relevant issues, as necessary.

Items reviewed by the DSMB include:

- Interim/cumulative data for evidence of study-related adverse events;
- Interim/cumulative data for evidence of efficacy according to pre-established statistical guidelines, if appropriate;
- Data quality, completeness, and timeliness;
- Adequacy of compliance with goals for recruitment and retention, including those related to the participation of women and minorities;
- Adherence to the protocol;
- Factors that might affect the study outcome or compromise the confidentiality of the trial data (such as protocol violations, unmasking, etc.); and,
- Factors external to the study, such as scientific or therapeutic developments, that may impact participant safety or the ethics of the study.

The DSMB will conclude each review with their recommendations to the COBRE program and NIH/NIGMS as to whether the study should continue without change or be modified or terminated. Recommendations regarding modification of the design and conduct of the study could include:

- Modifications of the study protocol based upon review of the safety data;
- Suspension or early termination of the study or of one or more study arms because of serious concerns about subjects' safety, inadequate performance or rate of enrollment;
- Suspension or early termination of the study or of one or more study arms because study objectives have not been obtained according to pre-established statistical guidelines; and,
- Corrective actions regarding a study when the performance appears unsatisfactory or suspicious.

Confidentiality must always be maintained during all phases of DSMB review and deliberation. Usually, only voting members of the DSMB should have access to interim analyses of outcome data by treatment group. Exceptions may be made when the DSMB deems it appropriate. The reason and to whom the exceptions for access to interim analyses is granted will be documented in the Closed Session Report. DSMB members must maintain strict confidentiality concerning all privileged trial results ever provided to them. The DSMB should review data only by masked study group (such as X vs. Y rather than experimental vs. control) unless or until the DSMB determines that the identities of the groups are necessary for their decision-making. Whenever masked data are presented to the DSMB, the key to the group coding must be available for immediate unmasking.

The DSMB will meet twice per year. The junior faculty investigator will attend these meetings and will be responsible for preparing and presenting up-to-date statistical reports on the progress of their trials. These reports will include data on recruitment, randomization, adherence, as well as statistical tests and special analyses requested by the DSMB. The Administrative Core will be responsible for organizing the annual in-person meeting and semiannual conference calls. They will put together data and materials submitted by junior faculty investigators and deliver them to DSMB members. The COBRE investigators will meet with the DSMB in the Open Session and not be present for the Closed Session.

Bibliography and References Cited

1. The Emerging Risk Factors Collaboration. Diabetes mellitus, fasting blood glucose concentration, and risk of vascular disease: a collaborative meta-analysis of 102 prospective studies. *Lancet*. 2010;375(9733):2215-2222. doi:10.1016/S0140-6736(10)60484-9.
2. Flaxman SR, Bourne RRA, Resnikoff S, et al. Global causes of blindness and distance vision impairment 1990-2020: a systematic review and meta-analysis. *Lancet Glob Heal*. October 2017. doi:10.1016/S2214-109X(17)30393-5.
3. United States Renal Data System. *2016 USRDS Annual Data Report: Epidemiology of Kidney Disease in the United States*. Bethesda, MD: National Institutes of Health, National Institute of Diabetes and Digestive and Kidney Diseases; 2016. https://www.usrds.org/2016/view/Default.aspx.
4. Narres M, Kvitkina T, Claessen H, et al. Incidence of lower extremity amputations in the diabetic compared with the non-diabetic population: A systematic review. *PLoS One*. 2017;12(8):e0182081. doi:10.1371/journal.pone.0182081.
5. Centers for Disease Control and Prevention. *National Diabetes Statistics Report, 2017*. Atlanta, GA: Centers for Disease Control and Prevention, U.S. Dept of Health and Human Services; 2017.
6. Centers for Disease Control and Prevention. *Long-Term Trends in Diabetes*. Atlanta, GA: Centers for Disease Control and Prevention, U.S. Dept of Health and Human Services; 2017.
7. NCD Risk Factor Collaboration (NCD-RisC). Worldwide trends in diabetes since 1980: A pooled analysis of 751 population-based studies with 4.4 million participants. *Lancet*. 2016;387(10027):1513-1530. doi:10.1016/S0140-6736(16)00618-8.
8. American Diabetes Association. Economic costs of diabetes in the U.S. in 2012. *Diabetes Care*. 2013;36(4):1033-1046. doi:10.2337/dc12-2625.
9. Zhuo X, Zhang P, Barker L, Albright A, Thompson TJ, Gregg E. The lifetime cost of diabetes and its implications for diabetes prevention. *Diabetes Care*. 2014;37(9):2557-2564. doi:10.2337/dc13-2484.
10. WHO, International Diabetes Foundation. *Definition and Diagnosis of Diabetes Mellitus and Intermediate Hyperglycaemia: Report of a WHO/IDF Consultation*. Geneva: Geneva: World Health Organization; 2006.
11. The International Expert Committee. International Expert Committee Report on the Role of the A1C Assay in the Diagnosis of Diabetes. *Diabetes Care*. 2009;32(7):1327-1334. doi:10.2337/dc09-9033.
12. American Diabetes Association. Standards of Medical Care in Diabetes - 2017. *Diabetes Care*. 2017;40(Supplement 1):S33-S43. doi:10.2337/dc16-S003.
13. Gerstein HC, Santaguida P, Raina P, et al. Annual incidence and relative risk of diabetes in people with various categories of dysglycemia: A systematic overview and meta-analysis of prospective studies. *Diabetes Res Clin Pract*. 2007;78(3):305-312. doi:10.1016/j.diabres.2007.05.004.
14. Huang Y, Cai X, Mai W, Li M, Hu Y. Association between prediabetes and risk of cardiovascular disease and all cause mortality: systematic review and meta-analysis. *BMJ*. 2016;355:i5953. doi:10.1136/bmj.i5953.
15. Tabák AG, Herder C, Rathmann W, Brunner EJ, Kivimäki M. Prediabetes: A high-risk state for diabetes development. *Lancet*. 2012;379(9833):2279-2290. doi:10.1016/S0140-

6736(12)60283-9.
16. Diabetes Prevention Program Research Group. 10-year follow-up of diabetes incidence and weight loss in the Diabetes Prevention Program Outcomes Study. *Lancet*. 2009;374(9702):1677-1686. doi:10.1016/S0140-6736(09)61457-4.
17. The DREAM (Diabetes REduction Assessment with ramipril and rosiglitazone Medication) Trial Investigators. Effect of rosiglitazone on the frequency of diabetes in patients with impaired glucose tolerance or impaired fasting glucose: a randomised controlled trial. *Lancet*. 2006;368(9541):1096-1105. doi:10.1016/S0140-6736(06)69420-8.
18. Perreault L, Pan Q, Mather KJ, Watson KE, Hamman RF, Kahn SE. Effect of regression from prediabetes to normal glucose regulation on long-term reduction in diabetes risk: Results from the Diabetes Prevention Program Outcomes Study. *Lancet*. 2012;379(9833):2243-2251. doi:10.1016/S0140-6736(12)60525-X.
19. Perreault L, Temprosa M, Mather KJ, et al. Regression from prediabetes to normal glucose regulation is associated with reduction in cardiovascular risk: Results from the diabetes prevention program outcomes study. *Diabetes Care*. 2014;37(9):2622-2631. doi:10.2337/dc14-0656.
20. Popkin BM. Nutrition Transition and the Global Diabetes Epidemic. *Curr Diab Rep*. 2015;15(9). doi:10.1007/s11892-015-0631-4.
21. Vazquez G, Duval S, Jacobs DRJ, Silventoinen K. Comparison of body mass index, waist circumference, and waist/hip ratio in predicting incident diabetes: a meta-analysis. *Epidemiol Rev*. 2007;29:115-128. doi:10.1093/epirev/mxm008.
22. Knowler WC, Barrett-Connor E, Fowler SE, et al. Reduction in the incidence of type 2 diabetes with lifestyle intervention or metformin. *N Engl J Med*. 2002;346(6):393-403. doi:10.1056/NEJMoa012512.
23. Pan XR, Li GW, Hu YH, et al. Effects of diet and exercise in preventing NIDDM in people with impaired glucose tolerance: The Da Qing IGT and diabetes study. *Diabetes Care*. 1997;20(4):537-544. doi:10.2337/diacare.20.4.537.
24. Tuomilehto J, Indstrom J, Eriksson J, Valle T, Hamalainine E, Uusitupa M. Prevention of Type 2 Diabetes Mellitus By Changes in Lifestyle Among Subjects With Impaired Glucose Tolerance. *N Engl J Med*. 2001;344(18):1343-1350. doi:10.1056/NEJM200105033441801.
25. Ramachandran A, Snehalatha C, Mary S, Mukesh B, Bhaskar AD, Vijay V. The Indian Diabetes Prevention Programme shows that lifestyle modification and metformin prevent type 2 diabetes in Asian Indian subjects with impaired glucose tolerance (IDPP-1). *Diabetologia*. 2006;49(2):289-297. doi:10.1007/s00125-005-0097-z.
26. The Diabetes Preventio Program Research Group. The Diabetes Prevention Program. Design and methods for a clinical trial in the prevention of type 2 diabetes. *Diabetes Care*. 1999;22(4):623-634. doi:10.2337/diacare.22.4.623 10.2337/diabetes.48.4.699.
27. Evert AB, Boucher JL, Cypress M, et al. Nutrition therapy recommendations for the management of adults with diabetes. *Diabetes Care*. 2013;36(11):3821-3842. doi:10.2337/dc13-2042.
28. Hu T, Mills KT, Yao L, et al. Effects of low-carbohydrate diets versus low-fat diets on metabolic risk factors: A meta-analysis of randomized controlled clinical trials. *Am J Epidemiol*. 2012;176(SUPPL. 7). doi:10.1093/aje/kws264.
29. Sackner-Bernstein J, Kanter D, Kaul S. Dietary Intervention for Overweight and Obese Adults: Comparison of Low-Carbohydrate and Low-Fat Diets. A Meta-Analysis. *PLoS*

One. 2015;10(10):e0139817. doi:10.1371/journal.pone.0139817.

30. Bazzano LA, Hu T, Reynolds K, et al. Effects of low-carbohydrate and low-fat diets: A randomized trial. *Ann Intern Med*. 2014;161(5):309-318. doi:10.7326/M14-0180.
31. Sawyer L, Gale EAM. Diet, delusion and diabetes. *Diabetologia*. 2009;52(1):1-7. doi:10.1007/s00125-008-1203-9.
32. Snorgaard O, Poulsen GM, Andersen HK, Astrup A. Systematic review and meta-analysis of dietary carbohydrate restriction in patients with type 2 diabetes. *BMJ Open Diabetes Res Care*. 2017;5(1):e000354. doi:10.1136/bmjdrc-2016-000354.
33. Larsen RN, Mann NJ, Maclean E, Shaw JE. The effect of high-protein, low-carbohydrate diets in the treatment of type 2 diabetes: A 12 month randomised controlled trial. *Diabetologia*. 2011;54(4):731-740. doi:10.1007/s00125-010-2027-y.
34. Saslow LR, Kim S, Daubenmier JJ, et al. A randomized pilot trial of a moderate carbohydrate diet compared to a very low carbohydrate diet in overweight or obese individuals with type 2 diabetes mellitus or prediabetes. *PLoS One*. 2014;9(4). doi:10.1371/journal.pone.0091027.
35. Tay J, Luscombe-Marsh ND, Thompson CH, et al. A very low-carbohydrate, low-saturated fat diet for type 2 diabetes management: A randomized trial. *Diabetes Care*. 2014;37(11):2909-2918. doi:10.2337/dc14-0845.
36. Davis NJ, Tomuta N, Schechter C, et al. Comparative Study of the Effects of a 1-Year Dietary Intervention of a Low- Carbohydrate Diet Versus a Low-Fat Diet on Weight and Glycemic Control in Type 2. *Diabetes Care*. 2009;32(7):1147-1152. doi:10.2337/dc08-2108.Clinical.
37. Saslow LR, Daubenmier JJ, Moskowitz JT, et al. Twelve-month outcomes of a randomized trial of a moderate-carbohydrate versus very low-carbohydrate diet in overweight adults with type 2 diabetes mellitus or prediabetes. *Nutr Diabetes*. 2017;7(12):304. doi:10.1038/s41387-017-0006-9.
38. Tay J, Thompson CH, Luscombe-Marsh ND, et al. Effects of an energy-restricted low-carbohydrate, high unsaturated fat/low saturated fat diet versus a high carbohydrate, low fat diet in type 2 diabetes: a 2 year randomized clinical trial. *Diabetes, Obes Metab*. 2017:n/a-n/a. doi:10.1111/dom.13164.
39. Yamada Y, Uchida J, Izumi H, et al. A non-calorie-restricted low-carbohydrate diet is effective as an alternative therapy for patients with type 2 diabetes. *Intern Med*. 2014;53(1):13-19. doi:http://dx.doi.org/10.2169/internalmedicine.53.0861.
40. Mayer SB, Jeffreys AS, Olsen MK, Mcduffie JR, Feinglos MN, Yancy WS. Two diets with different haemoglobin A1c and antiglycaemic medication effects despite similar weight loss in type 2 diabetes. *Diabetes, Obes Metab*. 2014;16(1):90-93. doi:10.1111/dom.12191.
41. Maekawa S, Kawahara T, Nomura R, et al. Retrospective study on the efficacy of a low-carbohydrate diet for impaired glucose tolerance. *Diabetes Metab Syndr Obes*. 2014;7:195-201. doi:10.2147/DMSO.S62681.
42. Davis C, Bryan J, Hodgson J, Murphy K. Definition of the mediterranean diet: A literature review. *Nutrients*. 2015;7(11):9139-9153. doi:10.3390/nu7115459.
43. Sofi F, Macchi C, Abbate R, Gensini GF, Casini A. Mediterranean diet and health status: an updated meta-analysis and a proposal for a literature-based adherence score. *Public Health Nutr*. 2014;17(12):2769-2782. doi:10.1017/S1368980013003169.
44. Jannasch F, Kröger J, Schulze MB. Dietary Patterns and Type 2 Diabetes: A Systematic Literature Review and Meta-Analysis of Prospective Studies. *J Nutr*. 2017;147(6):1174-

1182. doi:10.3945/jn.116.242552.

45. Schwingshackl L, Missbach B, König J, Hoffmann G. Adherence to a Mediterranean diet and risk of diabetes: a systematic review and meta-analysis. *Public Health Nutr*. 2014;18(7):1292–1299. doi:10.1017/S1368980014001542.

46. de Lorgeril M, Salen P, Martin J-L, Monjaud I, Delaye J, Mamelle N. Mediterranean Diet, Traditional Risk Factors, and the Rate of Cardiovascular Complications After Myocardial Infarction : Final Report of the Lyon Diet Heart Study. *Circulation*. 1999;99(6):779-785. doi:10.1161/01.CIR.99.6.779.

47. Estruch R, Ros E, Salas-Salvadó J, et al. Primary Prevention of Cardiovascular Disease with a Mediterranean Diet. *N Engl J Med*. 2013;368(14):1279-1290. doi:10.1056/NEJMoa1200303.

48. Salas-Salvadó J, Bulló M, Estruch R, et al. Prevention of diabetes with mediterranean diets: A subgroup analysis of a randomized trial. *Ann Intern Med*. 2014;160(1):1-10. doi:10.7326/M13-1725.

49. Esposito K, Marfella R, Ciotola M, et al. Effect of a Mediterranean-Style Diet on Endothelial Dysfunction and Markers of Vascular Inflammation in the Metabolic Syndrome. *JAMA*. 2004;292(12):1440. doi:10.1001/jama.292.12.1440.

50. Babio N, Toledo E, Estruch R, et al. Mediterranean diets and metabolic syndrome status in the PREDIMED randomized trial. *CMAJ*. 2014;186(17):E649-E657. doi:10.1503/cmaj.140764.

51. Esposito K, Maiorino MI, Bellastella G, Chiodini P, Panagiotakos D, Giugliano D. A journey into a Mediterranean diet and type 2 diabetes: a systematic review with meta-analyses. *BMJ Open*. 2015;5(8):e008222. doi:10.1136/bmjopen-2015-008222.

52. Micha R, Michas G, Mozaffarian D. Unprocessed red and processed meats and risk of coronary artery disease and type 2 diabetes--an updated review of the evidence. *Curr Atheroscler Rep*. 2012;14(6):515-524. doi:10.1007/s11883-012-0282-8.

53. Esposito K, Maiorino MI, Ciotola M, et al. Effects of a Mediterranean-style diet on the need for antihyperglycemic drug therapy in patients with newly diagnosed type 2 diabetes: A randomized trial. *Ann Intern Med*. 2009;151(5):306-314. doi:10.1097/OGX.0b013e3181e5a159.

54. Esposito K, Maiorino MI, Petrizzo M, Bellastella G, Giugliano D. The effects of a Mediterranean diet on the need for diabetes drugs and remission of newly diagnosed type 2 diabetes: Follow-up of a randomized trial. *Diabetes Care*. 2014;37(7):1824-1830. doi:10.2337/dc13-2899.

55. Elhayany A, Lustman A, Abel R, Attal-Singer J, Vinker S. A low carbohydrate Mediterranean diet improves cardiovascular risk factors and diabetes control among overweight patients with type 2 diabetes mellitus: A 1-year prospective randomized intervention study. *Diabetes, Obes Metab*. 2010;12(3):204-209. doi:10.1111/j.1463-1326.2009.01151.x.

56. Shai I, Schwarzfuchs D, Henkin Y, et al. Weight Loss with a Low-Carbohydrate, Mediterranean, or Low-Fat Diet. *N Engl J Med*. 2008;359(3):229-241. doi:10.1056/NEJMoa0708681.

57. Schwarzfuchs D, Golan R, Shai I. Four-Year Follow-up after Two-Year Dietary Interventions. *N Engl J Med*. 2012;367(14):1373-1374. doi:10.1056/NEJMc1204792.

58. Pan A, Sun Q, Bernstein AM, et al. Red meat consumption and risk of type 2 diabetes: 3 Cohorts of US adults and an updated meta-analysis. *Am J Clin Nutr*. 2011;94(4):1088-1096. doi:10.3945/ajcn.111.018978.

59. Hu EA, Pan A, Malik V, Sun Q. White rice consumption and risk of type 2 diabetes: meta-analysis and systematic review. *BMJ*. 2012;344:e1454. doi:10.1136/bmj.e1454.
60. Wang M, Yu M, Fang L, Hu R-Y. Association between sugar-sweetened beverages and type 2 diabetes: A meta-analysis. *J Diabetes Investig*. 2015;6(3):360-366. doi:10.1111/jdi.12309.
61. Imamura F, O'Connor L, Ye Z, et al. Consumption of sugar sweetened beverages, artificially sweetened beverages, and fruit juice and incidence of type 2 diabetes: systematic review, meta-analysis, and estimation of population attributable fraction. *BMJ*. 2015:h3576. doi:10.1136/bmj.h3576.
62. Carter P, Gray LJ, Troughton J, Khunti K, Davies MJ. Fruit and vegetable intake and incidence of type 2 diabetes mellitus: systematic review and meta-analysis. *BMJ*. 2010;341:c4229. doi:10.1136/bmj.c4229.
63. Cooper AJ, Forouhi NG, Ye Z, et al. Fruit and vegetable intake and type 2 diabetes: EPIC-InterAct prospective study and meta-analysis. *Eur J Clin Nutr*. 2012;66(10):1082-1092. doi:10.1038/ejcn.2012.85.
64. Chen M, Sun Q, Giovannucci E, et al. Dairy consumption and risk of type 2 diabetes: 3 cohorts of US adults and an updated meta-analysis. *BMC Med*. 2014;12(1):215. doi:10.1186/s12916-014-0215-1.
65. Aune D, Norat T, Romundstad P, Vatten LJ. Whole grain and refined grain consumption and the risk of type 2 diabetes: A systematic review and dose-response meta-analysis of cohort studies. *Eur J Epidemiol*. 2013;28(11):845-858. doi:10.1007/s10654-013-9852-5.
66. Ding M, Bhupathiraju SN, Chen M, van Dam RM, Hu FB. Caffeinated and decaffeinated coffee consumption and risk of type 2 diabetes: a systematic review and a dose-response meta-analysis. *Diabetes Care*. 2014;37(2):569-586. doi:10.2337/dc13-1203.
67. Li X-H, Yu F-F, Zhou Y-H, He J. Association between alcohol consumption and the risk of incident type 2 diabetes: a systematic review and dose-response meta-analysis. *Am J Clin Nutr*. 2016;103(3):818-829. doi:10.3945/ajcn.115.114389.
68. Muraki I, Imamura F, Manson JE, et al. Fruit consumption and risk of type 2 diabetes: results from three prospective longitudinal cohort studies. *BMJ*. 2013;347(aug28 1):f5001-f5001. doi:10.1136/bmj.f5001.
69. Upadhyaya S, Banerjee G. Type 2 diabetes and gut microbiome: At the intersection of known and unknown. *Gut Microbes*. 2015;6(2):85-92. doi:10.1080/19490976.2015.1024918.
70. David LA, Maurice CF, Carmody RN, et al. Diet rapidly and reproducibly alters the human gut microbiome. *Nature*. 2014;505(7484):559-563. doi:10.1038/nature12820.
71. Duncan SH, Belenguer A, Holtrop G, Johnstone AM, Flint HJ, Lobley GE. Reduced dietary intake of carbohydrates by obese subjects results in decreased concentrations of butyrate and butyrate-producing bacteria in feces. *Appl Environ Microbiol*. 2007;73(4):1073-1078. doi:10.1128/AEM.02340-06.
72. Dorans KS, Mostofsky E, Levitan EB, Håkansson N, Wolk A, Mittleman MA. Alcohol and Incident Heart Failure among Middle-Aged and Elderly Men: Cohort of Swedish Men. *Circ Hear Fail*. 2015;8(3):422-427. doi:10.1161/CIRCHEARTFAILURE.114.001787.
73. Steinhaus DA, Mostofsky E, Levitan EB, et al. Chocolate intake and incidence of heart failure: Findings from the Cohort of Swedish Men. *Am Heart J*. 2017;183:18-23. doi:10.1016/j.ahj.2016.10.002.
74. Dorans KS, Massa J, Chitnis T, Ascherio A, Munger KL. Physical activity and the incidence of multiple sclerosis. *Neurology*. 2016;87(17):1770-1776.

doi:10.1212/WNL.0000000000003260.
75. Mills KT, Chen J, Yang W, et al. Sodium Excretion and the Risk of Cardiovascular Disease in Patients With Chronic Kidney Disease. *JAMA*. 2016;315(20):2200. doi:10.1001/jama.2016.4447.
76. Chen J, Gu D, Huang J, et al. Metabolic syndrome and salt sensitivity of blood pressure in non-diabetic people in China: a dietary intervention study. *Lancet*. 2009;373(9666):829-835. doi:10.1016/S0140-6736(09)60144-6.
77. Li C, He J, Chen J, et al. Genome-Wide Gene-Sodium Interaction Analyses on Blood Pressure: The Genetic Epidemiology Network of Salt-Sensitivity Study. *Hypertension*. 2016;68(2):348-355. doi:10.1161/HYPERTENSIONAHA.115.06765.
78. He J, Streiffer RH, Muntner P, Krousel-Wood MA, Whelton PK. Effect of dietary fiber intake on blood pressure: a randomized, double-blind, placebo-controlled trial. *J Hypertens*. 2004;22(1):73-80. doi:10.1097/01.hjh.0000098164.70956.54.
79. He J, Ogden LG, Vupputuri S, Bazzano LA, Loria C, Whelton PK. Dietary sodium intake and subsequent risk of cardiovascular disease in overweight adults. *JAMA*. 1999;282(21):2027-2034. doi:10.1001/jama.282.21.2027.
80. He J, Wofford MR, Reynolds K, et al. Effect of dietary protein supplementation on blood pressure a randomized, controlled trial. *Circulation*. 2011;124(5):589-595. doi:10.1161/CIRCULATIONAHA.110.009159.
81. Thompson AM, Zhang Y, Tong W, et al. Association of inflammation and endothelial dysfunction with metabolic syndrome, prediabetes and diabetes in adults from Inner Mongolia, China. *BMC Endocr Disord*. 2011;11(1):16. doi:10.1186/1472-6823-11-16.
82. Irazola V, Rubinstein A, Bazzano L, et al. Prevalence, awareness, treatment and control of diabetes and impaired fasting glucose in the Southern Cone of Latin America. *PLoS One*. 2017;12(9). doi:10.1371/journal.pone.0183953.
83. Bazzano LA, Li TY, Joshipura KJ, Hu FB. Intake of fruit, vegetables, and fruit juices and risk of diabetes in women. *Diabetes Care*. 2008;31(7):1311-1317. doi:10.2337/dc08-0080.
84. Bazzano LA, Serdula M, Liu S. Prevention of Type 2 Diabetes by Diet and Lifestyle Modification. *J Am Coll Nutr*. 2005;24(5):310-319. doi:10.1080/07315724.2005.10719479.
85. Pollock BD, Chen W, Harville EW, et al. Differential sex effects of systolic blood pressure and low-density lipoprotein cholesterol on type 2 diabetes: Life course data from the Bogalusa Heart Study. *Journal of Diabetes*. 2017.
86. Zhang T, Li Y, Zhang H, et al. Insulin-sensitive adiposity is associated with a relatively lower risk of diabetes than insulin-resistant adiposity: the Bogalusa Heart Study. *Endocrine*. 2016;54(1):93-100. doi:10.1007/s12020-016-0948-z.
87. Pollock BD, Hu T, Chen W, et al. Utility of existing diabetes risk prediction tools for young black and white adults: Evidence from the Bogalusa Heart Study. *J Diabetes Complications*. 2016;31(1):86-93. doi:10.1016/j.jdiacomp.2016.07.025.
88. Hu T, Yao L, Reynolds K, et al. The effects of a low-carbohydrate diet vs. a low-fat diet on novel cardiovascular risk factors: A randomized controlled trial. *Nutrients*. 2015;7(9):7978-7994. doi:10.3390/nu7095377.
89. Hu T, Yao L, Reynolds K, et al. The effects of a low-carbohydrate diet on appetite: A randomized controlled trial. *Nutr Metab Cardiovasc Dis*. 2016;26(6):476-488. doi:10.1016/j.numecd.2015.11.011.

90. Rebholz CM, Reynolds K, Wofford MR, et al. Effect of soybean protein on novel cardiovascular disease risk factors: A randomized controlled trial. *Eur J Clin Nutr*. 2013;67(1):58-63. doi:10.1038/ejcn.2012.186.

91. Wofford MR, Rebholz CM, Reynolds K, et al. Effect of soy and milk protein supplementation on serum lipid levels: A randomized controlled trial. *Eur J Clin Nutr*. 2012;66(4):419-425. doi:10.1038/ejcn.2011.168.

92. Chen J, He J, Wildman RP, Reynolds K, Streiffer RH, Whelton PK. A randomized controlled trial of dietary fiber intake on serum lipids. *Eur J Clin Nutr*. 2006;60(1):62-68. doi:10.1038/sj.ejcn.1602268.

93. Overweight and Obesity Expert Panel. *Managing Overweight and Obesity in Adults: Systematic Evidence Review From the Obesity Expert Panel, 2013*.; 2013.

94. The Data Center. *Who Lives in New Orleans and Metro Parishes Now?*; 2017. https://www.datacenterresearch.org/data-resources/who-lives-in-new-orleans-now/.

95. Wetzels JFM, Kiemeney LALM, Swinkels DW, Willems HL, Heijer M Den. Age- and gender-specific reference values of estimated GFR in Caucasians: The Nijmegen Biomedical Study. *Kidney Int*. 2007;72(5):632-637. doi:10.1038/sj.ki.5002374.

96. Radin MS. Pitfalls in hemoglobin A1c measurement: When results may be misleading. *J Gen Intern Med*. 2014;29(2):388-394. doi:10.1007/s11606-013-2595-x.

97. Kim PS, Woods C, Georgoff P, et al. A1C underestimates glycemia in HIV infection. *Diabetes Care*. 2009;32(9):1591-1593. doi:10.2337/dc09-0177.

98. Qaseem A, Wilt TJ, Kansagara D, Horwitch C, Barry MJ, Forciea MA. Hemoglobin A1c Targets for Glycemic Control With Pharmacologic Therapy for Nonpregnant Adults With Type 2 Diabetes Mellitus: A Guidance Statement Update From the American College of Physicians. *Ann Intern Med*. 2018;168(8):569-576. doi:10.7326/M17-0939.

99. Sumithran P, Prendergast LA, Delbridge E, et al. Ketosis and appetite-mediating nutrients and hormones after weight loss. *Eur J Clin Nutr*. 2013;67(7):759-764. doi:10.1038/ejcn.2013.90.

100. Gardner CD, Trepanowski JF, Gobbo LCD, et al. Effect of low-fat VS low-carbohydrate diet on 12-month weight loss in overweight adults and the association with genotype pattern or insulin secretion the DIETFITS randomized clinical trial. *JAMA - J Am Med Assoc*. 2018;319(7):667-679. doi:10.1001/jama.2018.0245.

101. Brenner BM, Meyer TW, Hostetter TH. Dietary protein intake and the progressive nature of kidney disease: the role of hemodynamically mediated glomerular injury in the pathogenesis of progressive glomerular sclerosis in aging, renal ablation, and intrinsic renal disease. *N Engl J Med*. 1982;307(11):652-659. doi:10.1056/NEJM198209093071104.

102. Sato J, Kanazawa A, Makita S, et al. A randomized controlled trial of 130 g/day low-carbohydrate diet in type 2 diabetes with poor glycemic control. *Clin Nutr*. 2017;36(4):992-1000. doi:10.1016/j.clnu.2016.07.003.

103. Yancy WS, Foy M, Chalecki AM, et al. A low-carbohydrate, ketogenic diet to treat type 2 diabetes. *Nutr Metab (Lond)*. 2005;2(1):34. doi:10.1186/1743-7075-2-34.

104. Boden G, Sargrad K, Homko C, Mozzoli M, Stein TP. Effect of a low-carbohydrate diet on appetite, blood glucose levels, and insulin resistance in obese patients with type 2 diabetes. *Ann Intern Med*. 2005;142(6). doi:10.1016/S0084-3741(08)70336-6.

105. Stern L, Iqbal N, Seshadri P. The Effects of Low-Carbohydrate versus Conventional Weight Loss Diets in Severely Obese Adults : One-Year Follow-up of a randomized control trial. *Ann Intern …*. 2004;140(May 2001):769-777.

doi:10.1016/j.accreview.2004.07.103.

106. Westman EC, Yancy WS, Edman JS, Tomlin KF, Perkins CE. Effect of 6-month adherence to a very low carbohydrate diet program. *Am J Med*. 2002;113(1):30-36. doi:10.1016/S0002-9343(02)01129-4.

107. Moosheer SM, Waldschütz W, Itariu BK, Brath H, Stulnig TM. A protein-enriched low glycemic index diet with omega-3 polyunsaturated fatty acid supplementation exerts beneficial effects on metabolic control in type 2 diabetes. *Prim Care Diabetes*. 2014;8(4):308-314. doi:10.1016/j.pcd.2014.02.004.

108. Aagaard K, Petrosino J, Keitel W, et al. The Human Microbiome Project strategy for comprehensive sampling of the human microbiome and why it matters. *FASEB J*. 2013;27(3):1012-1022. doi:10.1096/fj.12-220806.

109. McInnes P, Cutting M. Manual of Procedures for Human Microbiome Project. http://www.hmpdacc.org/doc/HMP_MOP_Version12_0_072910.pdf.

110. Marrero DG, Palmer KNB, Phillips EO, Miller-Kovach K, Foster GD, Saha CK. Comparison of commercial and self-initiated weight loss programs in people with prediabetes: A randomized control trial. *Am J Public Health*. 2016;106(5):949-956. doi:10.2105/AJPH.2015.303035.

111. Barengolts E, Manickam B, Eisenberg Y, Akbar A, Kukreja S, Ciubotaru I. EFFECT OF HIGH-DOSE VITAMIN D REPLETION ON GLYCEMIC CONTROL IN AFRICAN-AMERICAN MALES WITH PREDIABETES AND HYPOVITAMINOSIS D. *Endocr Pract*. 2015. doi:10.4158/EP14548.OR.

112. Block G, Azar KMJ, Romanelli RJ, et al. Diabetes prevention and weight loss with a fully automated behavioral intervention by email, web, and mobile phone: A randomized controlled trial among persons with prediabetes. *J Med Internet Res*. 2015. doi:10.2196/jmir.4897.

113. Kramer MK, Molenaar DM, Arena VC, et al. Improving employee health: Evaluation of a worksite lifestyle change program to decrease risk factors for diabetes and cardiovascular disease. *J Occup Environ Med*. 2015. doi:10.1097/JOM.0000000000000350.

114. Parker AR, Byham-Gray L, Denmark R, Winkle PJ. The Effect of Medical Nutrition Therapy by a Registered Dietitian Nutritionist in Patients with Prediabetes Participating in a Randomized Controlled Clinical Research Trial. *J Acad Nutr Diet*. 2014;114(11):1739-1748. doi:10.1016/j.jand.2014.07.020.

115. Maruthur NM, Ma Y, Delahanty LM, et al. Early response to preventive strategies in the diabetes prevention program. *J Gen Intern Med*. 2013;28(12):1629-1636. doi:10.1007/s11606-013-2548-4.

116. Leong A, Daya N, Porneala B, et al. Prediction of Type 2 Diabetes by Hemoglobin A1c in Two Community-Based Cohorts. *Diabetes Care*. 2017;41(1):dc170607. doi:10.2337/dc17-0607.

117. Khaw K-T, Wareham N, Bingham S, Luben R, Welch A, Day N. Association of hemoglobin A1c with cardiovascular disease and mortality in adults: the European prospective investigation into cancer in Norfolk. *Ann Intern Med*. 2004;141(6):413-420. doi:10.7326/0003-4819-141-6-200409210-00006.

118. Cole RE, Boyer KM, Spanbauer SM, Sprague D, Bingham M. Effectiveness of Prediabetes Nutrition Shared Medical Appointments. *Diabetes Educ*. 2013;39(3):344-353. doi:10.1177/0145721713484812.

Low-Carbohydrate Dietary Pattern on Glycemic Outcomes Trial

PROTOCOL

PROTOCOL VERSION 1.8

Dated: November 14, 2019

New Orleans, LA

Table of Contents

1. Overview 5
2. Background and Significance 6
2.A. Type 2 Diabetes Mellitus is a major global public health challenge 6
2.B. Prediabetes is a major risk factor for cardiometabolic diseases 6
2.C. Role of lifestyle in preventing diabetes 6
2.D. Low-to-moderate carbohydrate diets, weight loss, and cardiometabolic diseases 7
2.E. Mediterranean-style diet is associated with reduced risk of T2D and improved glycemic control 8
2.F. Evidence for low-carbohydrate diets that emphasize Mediterranean-style dietary components 8
2.G. Evidence for specific food groups and risk of T2D 8
2.H. Gut microbiota, diet and T2D 9
2.I. Continuous Glucose Monitoring 9
2.I. Clinical and public health significance of the proposed study 9
2.J. Innovation 10
3. Preliminary Studies 10
3.A. Summary of aggregate experience of the research team 10
3.B. Low-carbohydrate behavioral dietary intervention trial experience 11
3.C. Other dietary intervention trial experience 11
4. Study Design and Organization 12
4.A. Overview of trial design 12
4.B. Study outcomes 12
4.B.1. Primary outcome 12
4.B.2. Secondary outcomes 13
4.C Study Timeline 13
6. Study Participants 13
6.A. Eligibility criteria 13
7. Recruitment and Randomization 14
7.A. Recruitment 14
7.A.1. Mass mailing, placement of brochures/flyers in community, TV/radio/billboard advertisements 14
7.A.2. Database searches and targeted mailing 14
7.A.3. Referrals and Local Partnerships 15
7.B. Randomization 15

7.C. Masking 15
7.D. Safety Considerations 15
8. Dietary Intervention Program 15
8.A. Low-carbohydrate dietary intervention group 15
8.B. Usual Diet Group 17
8.C. Physical Activity 17
8.D. Dietary Adherence 17
9. Safety of Dietary Intervention 18
10. Data Collection 18
10.A. Study visits 18
10.A.1. Screening/baseline visits 19
10.A.2. Follow-up visits and final visit 20
10.A.3. CGM 6-month visit 20
10.B Study measurements 21
10.B.1. Questionnaires 21
10.B.2. Dietary measurements 21
10.B.3. Assessment and management of adverse effects 21
10.B.4. BP and anthropometric measures 21
10.B.5. Blood samples 21
10.B.6. Urine collection 22
10.B.7. Stool Specimen Collection 22
10.B.8. CGM at 6 months: 22
11. Quality Assurance and Quality Control 23
11.A. Manual of procedures 23
11.B. Training and certification 23
12. Sample Size and Statistical Power 24
13. Data Management and Analysis 25
13.A. Data management 25
13.B. Data analysis plan 25
13.B.1. Exploratory data analysis 25
13.B.2. Primary, secondary, and exploratory outcomes 25
15. Anticipated Challenges 25
16. Protection of Human Subjects 26

16.A. Protection of human subjects from research risks 26
16.A.1. Risks to the subjects 26
16.A.2. Adequacy of protection against risks 28
16.A.3. Potential benefits of the proposed research to human subjects and others... 29
16.A.4. Importance of knowledge to be gained 29
16.A.5. Remuneration 29
16.B. Data safety and monitoring plan 29
Bibliography and References Cited 31

1. Overview

The increasing prevalence of type 2 diabetes mellitus (T2D), a major cause of morbidity and mortality worldwide, is a critical public health concern. In the United States, an estimated 33.9% of adults have prediabetes (impaired fasting glucose, elevated hemoglobin A1c (HbA1c), or impaired glucose tolerance). Individuals with prediabetes are at elevated risk of T2D and cardiovascular disease (CVD), with 5–10% developing clinical T2D each year.

In the short-term, among patients with T2D, low-to-moderate carbohydrate diets (energy percentage below 45) have a greater glucose-lowering effect than do high-carbohydrate diets, with larger glucose-lowering effect the greater the carbohydrate reduction. Additionally, compared with low-fat diets, Mediterranean diets that are high in unsaturated fats (particularly *cis*-monounsaturated fats) reduce risk of T2D and CVD and improve glycemic control among diabetics. However, compared with usual diets, the effect of a behavioral intervention promoting a low-carbohydrate/high-unsaturated fat and protein dietary pattern that emphasizes consumption of non-starchy vegetables, plant-based oils, fish, poultry, and mixed nuts on glucose homeostasis, and other metabolic risk factors among individuals with prediabetes or early-stage untreated T2D is not well understood.

Our *long-term goal* is to develop and implement dietary intervention(s) that reduce risk of T2D. The *overall goal of this randomized controlled trial is to study the effect of a behavioral intervention promoting a low-carbohydrate/high-unsaturated fat and high-protein dietary pattern (initial target <40 g digestible carbohydrates, final target <60 g digestible carbohydrates) compared with a usual diet on metabolic risk factors among individuals with prediabetes or early-stage untreated diabetes* (HbA1c 6.0–6.9%). Specifically, we will test the following hypotheses:

Primary hypothesis: Compared with usual diet, over a 6-month period, a healthy low-carbohydrate/high unsaturated fat and high-protein dietary pattern will lead to *a larger decrease of HbA1c, by at least 0.15%.*

Secondary hypothesis: Over a 6-month period, a healthy low-carbohydrate/high-unsaturated fat and high-protein dietary pattern will lead to *larger improvements in the following major secondary outcomes*: fasting glucose, systolic blood pressure (BP), total-to-HDL-cholesterol ratio, and body weight.

Exploratory outcomes: We will also study the effect of the intervention on the following outcomes: insulin and homeostasis model assessment of insulin resistance (HOMA-IR); diastolic BP; waist circumference; and estimated CVD risk.

To achieve these study aims, we will recruit 112 adults with prediabetes or untreated diabetes (HbA1c 6.0–6.9%) in the Greater New Orleans, LA area. Using a stratified, blocked randomization scheme, participants will be assigned to either a behavioral modification intervention aimed at promoting a healthy low-carbohydrate dietary pattern or to usual diet. The intervention group will have an intensive phase for 3 months, followed by a maintenance phase in months 4 through 6. The *primary outcome will be difference between the active intervention and control groups in the change in HbA1c from baseline to 6 months of follow-up. We expect 85% power to detect a difference of at least 0.15% in change of HbA1c.* Changes in other metabolic risk factors will be studied concurrently. Metabolic risk factors will be assessed at baseline, 3 months, and 6 months of follow-up.

There is much interest regarding dietary approaches to prevent T2D and its complications. The results from this study will help to determine whether, compared with usual diet, a behavioral intervention aimed at promoting a healthy low-carbohydrate dietary pattern improves metabolic risk factors.

2. Background and Significance

2.A. Type 2 Diabetes Mellitus is a major global public health challenge

Diabetes is a strong and independent risk factor for CVD morbidity and mortality. A meta-analysis of 102 prospective studies (698,782 participants) estimated diabetes leads to an approximately 2-fold increased risk in vascular diseases, including coronary heart disease (CHD) and major stroke subtypes, and deaths due to other vascular causes.[1] Among participants with diabetes, CHD risk is about 50% higher among those with fasting blood glucose ≥7 mmol/L compared with those with fasting blood glucose <7 mmol/L.[1] In addition to increasing CVD risk, diabetes can cause diabetic retinopathy,[2] chronic kidney disease,[3] lower limb amputation,[4] and other complications. Most individuals with diabetes have T2D.[5] The US Centers for Disease Control and Prevention (CDC) estimated that 30.2 million adults ≥18 years (12.2%) had diabetes in 2015, with 7.2 million (23.8%) not being aware of or reporting diabetes.[5] Diagnosed diabetes prevalence among US adults has increased substantially over time, from 2.54% in 1980 to 7.40% in 2015.[6] Diabetes prevalence is also high globally, with an estimated 2014 age-standardized prevalence of 9.0% in men and 7.9% in women.[7] Diabetes accounts for an increasing portion of US healthcare expenditures. Total cost of diagnosed diabetes in the US was estimated to be $245 billion in 2012, 41% higher than in 2007.[8] Diabetes is associated with much higher lifetime medical expenses, with higher expenses for those diagnosed at younger ages.[9]

2.B. Prediabetes is a major risk factor for cardiometabolic diseases

Prediabetes, defined by the American Diabetes Association (ADA) as impaired fasting glucose (IFG), impaired glucose tolerance (IGT), or elevated HbA1c, is also referred to as “intermediate hyperglycemia”[10] and “high-risk state of developing diabetes”.[11] The ADA defines prediabetes as having: fasting plasma glucose of 100–125 mg/dL (IFG); 2-hour post-load glucose in the 75-g oral glucose tolerance test (OGTT) of 140–199 mg/dL (IGT); or HbA1c 5.7–6.4%.[12] Prediabetes itself is not a disease; for all three tests, “risk is continuous, extending below the lower limit of the range and becoming disproportionately greater at the higher end of the range”.[12] The CDC estimated that in 2015, 33.9% of US adults had prediabetes based on fasting glucose or HbA1c.[5] Among those with prediabetes, only 11.9% reported having being told this by a medical professional.[5] Approximately 5–10% of individuals with prediabetes develop diabetes within one year, depending on how prediabetes is defined.[13] In a meta-analysis, annual diabetes incidence was 15–19% among individuals with both IFG and IGT, while it was 4–6% in those with isolated IFG and 6–9% in those with isolated IGT.[13]

A recent meta-analysis (1,611,339 participants from 53 studies) reported that prediabetes as defined by the ADA is associated with higher risk of composite CVD events and CHD.[14] IFG and IGT were also positively associated with increased risk of stroke and all-cause mortality.[14] Prediabetes is linked with increased risk of other conditions typically thought of as diabetes complications, such as neuropathies and nephropathies.[15] Importantly, it is possible for individuals with prediabetes to revert to normoglycemia, as observed in observational studies[13] and lifestyle or drug intervention trials.[16,17] In the Diabetes Prevention Program (DPP), reversion to normoglycemia was associated with lower long-term T2D risk[18] and lower predicted CVD risk.[19]

2.C. Role of lifestyle in preventing diabetes

Much evidence from observational studies and clinical trials supports the critical role of diet and other lifestyle factors in preventing T2D. Globally, lifestyle transitions towards reduced physical activity, increased consumption of refined carbohydrates, coupled with resulting changes in body composition, has led to increases in diabetes prevalence and severity.[20] Body

mass index (BMI), waist circumference, and waist-to-height ratio are predictors of T2D.[21] The DPP, which combined calorie restriction and exercise to promote weight loss, substantially reduced risk of T2D (by 58%) among individuals with elevated FPG and IGT;[22] a benefit that persisted in 10-year follow-up analyses.[16] Other trials of lifestyle modification focused on diet and exercise have also shown benefits in study populations in China,[23] Finland,[24] and India.[25]

2.D. Low-to-moderate carbohydrate diets, weight loss, and cardiometabolic diseases

Low-to-moderate carbohydrate diets (<45% of energy from carbohydrates) are popular dietary approaches for losing weight. Weight loss is a key component of the DPP lifestyle intervention[26] and ADA lifestyle recommendations.[12,27] Meta-analyses from our group[28] and others support that low-to-moderate carbohydrate diets are at least as effective as low-fat diets at promoting weight loss. For instance, a meta-analysis reported a 2-kg (95% CI, 3.1–0.9) larger weight loss in low-carbohydrate diet (<120 g carbohydrates/day) arms compared with low-fat diet (<30% energy from fat) arms.[29] Estimated 10-year CVD risk was lower in low-carbohydrate than low-fat arms.[29] This aligns with research published by our team; in a 12-month trial of participants with obesity, a low-carbohydrate diet (target <40 g carbohydrates/day) was more effective than a low-fat diet (target <30% energy from fat, <7% saturated fat) for weight loss and CVD risk factor reduction.[30]

Low-to-moderate carbohydrate diets may also be beneficial among patients with T2D. Low-carbohydrate diets have come in and out of favor as treatment of diabetes.[31] In a meta-analysis (1376 patients;10 trials), after 3 or 6 months of the intervention, low-to-moderate carbohydrate diets (<45% energy from carbohydrates) led to a 0.34% lower HbA1c (95% CI, 0.06–0.63%) compared with high-carbohydrate diets.[32] Compared with high-carbohydrate diets, there was a greater reduction in HbA1c the greater the reported carbohydrate restriction.[32] There was no benefit of the low-to-moderate carbohydrate diets on HbA1c at 12 months or longer, and no difference between interventions on BMI, body weight, low-density lipoprotein (LDL) cholesterol or quality of life.[32] However, results may have been affected by dietary adherence or glucose medication. There was evidence of diabetes medication reduction in the low-carbohydrate arms in some studies,[33–37] with evidence of medication reduction and improved blood glucose stability persisting for up to 2 years.[38] Hypoglycemia (though not after medication adjustment) was observed in three subjects taking insulin or a sulfonylurea out of twelve subjects in the low-carbohydrate arm of another study.[39] In a subset of participants with T2D (n=46) from a large weight loss trial, compared with those randomized to a low-fat diet and orlistat, those randomized to a low-carbohydrate diet for 48 weeks had improved HbA1c (0.8% lower, 95% CI, -1.5, -0.02%) and a reduction in antiglycemic medications, despite similar weight loss effects of the interventions.[40]

Though low-to-moderate carbohydrate diets have been studied among overweight and obese participants (primarily focused on weight loss), less is understood about the effects of such diets on glycemic outcomes specifically among individuals with prediabetes. A study among participants with IGT observed a reduction at 12 months in weight, HbA1c, and other glycemic outcomes among individuals who participated in a 7-day in-hospital education program, though this was not randomized.[41] A randomized pilot trial of overweight or obese individuals with T2D or prediabetes found that, over 3 months and at 12 months, a very low-carbohydrate diet improved HbA1c, led to higher medication discontinuation, and more weight loss compared with a low-fat diet.[34,37] However, only 4 individuals had prediabetes. Given benefits of low-to-moderate carbohydrate diets for weight loss in general populations and for glycemic control among patients with T2D, further study of these diets are warranted among individuals with prediabetes who are at elevated cardiometabolic disease risk.

2.E. Mediterranean-style diet is associated with reduced risk of T2D and improved glycemic control

Though there is no one “Mediterranean diet”, the Mediterranean dietary pattern tends to have the following characteristics: high consumption of vegetables, fruits, breads and cereals, legumes, nuts and seeds; low-to-moderate consumption of dairy products, fish, poultry and eggs; little consumption of red meat and sweets; and red wine in moderation.[42] Olive oil is an important source of fat. In many observational studies, adherence to the Mediterranean-style dietary pattern is associated with reduced risk of CVD[43] and T2D.[44,45] Evidence from randomized trials also supports the benefits of this dietary pattern. In several trials, participants randomized to Mediterranean diets had substantially lower risk of CVD than those randomized to low-fat control diets.[46,47] In the three-arm randomized PREDIMED trial of participants at high risk of CVD, after 4.1 years of follow-up, those randomized to a traditional Mediterranean diet supplemented with either olive oil or nuts had a 30% reduced risk of T2D compared to those in the low-fat control diet group.[48] In all PREDIMED arms, average carbohydrate intake was relatively low (39.7–43.7% at end of trial).[47] Two trials have found a higher rate of remission from metabolic syndrome among individuals randomized to a Mediterranean diet compared with those randomized to control diets (prudent or low-fat) during 2–5 years of follow-up.[49,50] The Mediterranean pattern may also be beneficial among individuals with T2D. Meta-analyses have reported that, among patients with T2D, those randomized to a Mediterranean diet had lower HbA1c levels and CVD risk factors, and more significant weight loss, compared with those randomized to control diets.[51]

2.F. Evidence for low-carbohydrate diets that emphasize Mediterranean-style dietary components

Many low-carbohydrate diets involve increased consumption of food items, such as red meat,[52] that are associated with increased cardiometabolic disease risk. However, low-carbohydrate dietary approaches can focus on substituting carbohydrates with foods common in Mediterranean-style diets, such as olive oil and nuts, which may improve cardiometabolic health. In a 4-year Italian study of individuals with newly diagnosed T2D, those randomized to a low-carbohydrate Mediterranean diet (<50% carbohydrates; consumed ~44%) had more favorable changes in glycemic control, higher rates of T2D remission, and delayed need for antiglycemic drugs compared with participants randomized to a low-fat diet (<30% fat, <10% saturated fat).[53,54] A 12-month Israeli study assessed low-carbohydrate Mediterranean (35% carbohydrates), traditional Mediterranean, and ADA (both 50–55% carbohydrates) diets in 259 overweight participants with T2D.[55] Most CVD risk factors improved in all groups; there was only HDL improvement in the low-carbohydrate Mediterranean diet and this diet was superior to the other diets in improving glycemic control.[55] In a 2-year weight-loss study of moderately obese participants, low-carbohydrate and Mediterranean diets were superior to low-fat diet for weight loss.[56] Participants in the low-carbohydrate arm were counseled to choose vegetarian sources of fat and protein and to avoid trans fat. At 6 years after study commencement, despite weight regain, there were still long-term benefits, particularly for the low-carbohydrate and Mediterranean diets.[57] In whole, evidence supports that low-carbohydrate Mediterranean-style diets are feasible and may have metabolic benefits.

2.G. Evidence for specific food groups and risk of T2D

Prospective cohort studies have found consumption of specific foods and beverages is associated with diabetes risk. Meta-analyses report higher consumption of processed and unprocessed red meat,[58] white rice (particularly in Asian populations),[59] and sugar-sweetened beverages[60,61] are associated with higher T2D risk. Consumption of green leafy vegetables,[62,63] yogurt,[64] whole grains,[65] decaffeinated and total coffee,[66] and light-to-moderate alcohol

consumption[67] are associated with reduced risk. Consumption of fruits, particularly blueberries, grapes, and apples, are associated with reduced risk; fruit juice is associated with higher risk.[68]

2.H. Gut microbiota, diet and T2D

There is increasing evidence for the importance of gut microbiota in risk of type 2 diabetes and other cardiometabolic diseases.[69] Additionally, dietary modifications, including changes in carbohydrate consumption, can lead to substantial changes in gut microbiome over short periods of time.[70,71] Less is known about longer-term changes to the gut microbiota during the adoption of a low-carbohydrate diet and how these changes may influence cardiometabolic risk. We plan to collect stool specimens at baseline, 3 months, and at 6 months, for potential future studies on how this intervention affects the gut microbiome.

2.I. Continuous Glucose Monitoring

As HbA1c is a marker of weighted average glucose over the past 8 to 12 weeks, it does not explicitly capture short-term glycemic excursions or variability. It has been theorized that such fluctuations may have an independent role in the development of diabetes complications.[3] Though more evidence is needed, research has found relationships of higher glycemic variability with coronary artery disease (CAD) and microvascular outcomes in type 2 diabetes (T2D).[4,5] This suggests that improved control of extreme glucose excursions, particularly postprandially, may help to reduce risks of such complications.[5]

In one study of a low-calorie, low-carbohydrate diet compared with a low-calorie, low-fat behavioral dietary intervention among individuals with T2D, there was evidence that some markers of glycemic variability were further reduced in the low-carbohydrate arm compared with the low-fat arm (assessed by 48-hour continuous glucose monitoring) at 6 months, 1 year, and 2 years.[6–8] However, there is not prior work assessing how a behavioral intervention aimed at reducing carbohydrate consumption affects mean glucose, glucose exposure above the set-point of 100 mg/dL, glucose excursions, or glycemic variability as measured by continuous glucose monitoring among individuals with untreated T2D or at high risk of T2D.

Given this background, we will use continuous glucose monitors (CGM) to collect data for up to 14 days on glucose levels at the end of the 6-months study and will compare mean glucose among those in the intervention group compared with mean glucose among those in the control group. We will also measure other markers of glycemic exposure, such as glycemic variability (mean amplitude of glycemic excursions (MAGE), standard deviation of glucose, coefficient of variation) and mean 24-hour glucose during wake-time (6:00 AM to 12:00 AM), and during sleep-time (12:00 AM to 6:00 AM).

2.I. Clinical and public health significance of the proposed study

Prediabetes, or intermediate hyperglycemia, is a marker of high risk of developing T2D and other complications. Due to increasing prevalence of T2D and associated morbidity and mortality globally, a better understanding of dietary approaches to delay progression to T2D or to promote reversion to normoglycemia is a high public health priority. This study proposes to assess the effect of a behavioral intervention promoting a low-carbohydrate/high-unsaturated fat dietary pattern (initial target <40 g digestible carbohydrates, final target <60 g digestible carbohydrates) on HbA1c, a marker of glycemic control, and other metabolic risk factors. Building from prior findings from clinical and observational studies, we will develop a dietary intervention that promotes the consumption of non-starchy vegetables, plant-based oils, fish, poultry, and mixed nuts, that are thought to reduced risk of T2D or CVD. Participants assigned

to the intervention will be trained in behavioral techniques that will provide capacity to maintain dietary changes long after study completion. Our outcome measures will assess the effect of this dietary intervention in a "real-life" setting as opposed to dietary modifications achieved in metabolic ward studies or through food prepared in research kitchens. Findings from this study may provide evidence in support of promoting alternative dietary styles among individuals with high T2D and CVD risk and could potentially be used to inform dietary interventions used in programs like DPP.

2.J. Innovation

The status quo as it pertains to dietary approaches for reducing risk of T2D is focused on reducing caloric intake and total fat intake. However, recent evidence suggests that the types of fat, rather than the quantity of fats may be more important, with evidence supporting that a Mediterranean-style diet that does not restrict fat intake reduces risk of developing both T2D[48] and CVD[47]. Moreover, in the short-term, low-carbohydrate diets, which are popular weight loss diets, have a greater glucose-lowering effect than do high-carbohydrate diets among patients with T2D.[32] However, the effect of low-carbohydrate diets that emphasize consumption of non-starchy vegetables, plant-based oils, fish, poultry, and mixed nuts on T2D prevention is unclear, particularly compared with higher-carbohydrate diets more commonly consumed in the US. *The proposed research is innovative, in our opinion, because it represents a substantial departure from the status quo by assessing the combined effect of a low-carbohydrate, high unsaturated fat and protein diet on cardiometabolic risk factors among adults with prediabetes or early-stage untreated diabetes.* We expect the results from this study have the potential to lead to new horizons for developing and implementing dietary approaches (other than the most commonly used reduced fat diet) that will substantially reduce risk of cardiometabolic disease among adults with T2D or at high risk of T2D. The proposed intervention dietary approach promotes consumption of dietary components thought to reduce risk of cardiometabolic disease and the behavior change intervention has expected applicability in clinical practice.

3. Preliminary Studies

3.A. Summary of aggregate experience of the research team

Dr. Kirsten Dorans is trained in cardiovascular disease epidemiology, with strong training in epidemiologic methods and biostatistics. Her publication record includes studies of behavioral risk factors and risk of chronic disease. She has published on the relationships between dietary intake and chronic heart failure[72,73] and on physical activity and incident multiple sclerosis[74]. She served as lead author, conducting data management, analysis, drafting, and revision of two of these manuscripts. In graduate school, she focused on studying links between outdoor air pollution and chronic diseases and has published several papers in this field. The COBRE program will provide an excellent opportunity for the JFI to transition from air pollution research to clinical and translational research focused on nutrition and diabetes. Building off of her training in observational epidemiology, this program will involve training and practical experience that will allow her to broaden her expertise and to develop practical skills for carrying out clinical research.

The mentorship team has much experience and prior successful collaborations in key areas related to this study. Dr. Jiang He is an internationally renowned expert in cardiometabolic disease clinical and epidemiological research. He has served as PI for many epidemiological studies and clinical trials, including clinical and observational studies focused on dietary consumption and chronic diseases[75–80] and has conducted extensive global research on diabetes.[81,82] Dr. Bazzano has collaborated extensively with Dr. He for more than 15 years in research related to diet and chronic disease. She is a clinical investigator and has expertise in

nutritional epidemiology and clinical research. Dr. Bazzano was the PI of a highly successful clinical trial comparing the effects of low-carbohydrate and low-fat diets on weight and cardiovascular risk factors among individuals with obesity, which was a subproject within the Tulane COBRE in Hypertension and Renal Biology.[30] Dr. He served as Dr. Bazzano's mentor for this clinical trial. They have collaborated on much other work related to dietary and chronic disease, including a meta-analysis on low-carbohydrate diets.[28] Dr. Bazzano has also published many articles on diet and risk of diabetes[83,84] and on diabetes in the Bogalusa Heart Study.[85–87]

3.B. Low-carbohydrate behavioral dietary intervention trial experience

In the study on macronutrient composition of diet and risk factors for cardiovascular disease (MACRO) study, 148 individuals without clinical cardiovascular disease or diabetes and with obesity were randomly assigned to a low-carbohydrate or a low-fat behavioral dietary intervention for 12 months. In the low-carbohydrate arm, daily dietary intake of carbohydrates decreased from an average of 242 g at baseline to 97 g, 93 g, and 127 g at 3, 6, and 12 months, respectively. At 12 months, participants in the low-carbohydrate intervention group had greater decreases in weight (-3.5 kg; 95% CI, -5.6 to -1.4 kg) and fat mass (-1.5%; 95% CI, -2.6% to -0.4%) and greater improvement in several cardiovascular risk factors.[30] This suggests that restricting carbohydrates may be an option for weight loss and reduction of cardiovascular risk factors. Further analyses found that the low-carbohydrate dietary intervention led to similar or greater improvement in inflammation, adipocyte dysfunction, and endothelial dysfunction compared with the low-fat dietary intervention[88] and that the low-fat diet reduced peptide YY more than the low-carbohydrate diet, suggesting satiety may be better preserved on low-carbohydrate diets.[89] No serious adverse events were reported in this study and the number of participants with symptoms were similar between the two diets, with the exception of more participants having headaches on the low-fat diet at 3 months (18 vs 6 participants, $p = 0.03$).[30] Dr. Bazzano served as PI for this study and Dr. He served as her mentor. Dr. Bazzano mentored one of her PhD students in conducting analyses and writing and editing manuscripts related to this project.

3.C. Other dietary intervention trial experience

Dr. He has also led or been involved with many dietary intervention trials, including both behavioral change and feeding study interventions. In the Protein and Blood Pressure (ProBP) study, 352 adults with prehypertension or stage 1 hypertension in New Orleans, LA and Jackson, MS were assigned to take, in a random order, 40 g/d soy protein, milk protein, or carbohydrate supplementation each for 8 weeks. Compared with the high-glycemic index refined carbohydrate, both soy protein and milk protein supplementation were associated with a -2.0 mm Hg (95% CI -3.2 to -0.7 mm Hg) and -2.3 mm Hg (-3.7 to -1.0 mm Hg) net change in systolic blood pressure.[80] These findings suggest that partial replacement of carbohydrate with soy or milk protein might be an important part of nutrition intervention strategies to prevent and treat hypertension. Additionally, soy protein supplementation led to reduction in plasma E-selectin compared with carbohydrate and milk protein and in plasma leptin compared with carbohydrate.[90] Soy protein also led to improvement in lipid profile compared with carbohydrate and milk protein.[91] Dr. He was PI for this project.

In the Fiber Intervention on Blood Pressure (FIBRE) study, 110 participants ages 30–65 years with untreated pre-hypertension or stage-1 hypertension and serum cholesterol level < 240 mg/dl were randomly assigned to 8-g/day of water soluble fiber from oat bran or a control intervention (double-blind RCT). Over the 12-week study period, net changes in systolic and diastolic blood pressure were -1.8 mm Hg (-4.3 to 0.8) and -1.2 mm Hg (-3.0 to 0.5 mm Hg), respectively.[78] Net changes in total, HDL-, and LDL-cholesterol were -2.40 mg/dL (-10.60 to

5.81 mg/d:), -1.66 mg/dL (-4.55 to 1.22 mg/dL), and -1.33 mg/dl (-8.33 to 5.68 mg/dL), respectively.[92] Dr He was PI for this study.

Dr. He is currently serving as a mentor for Dr. Katherine Mills' COBRE project (Effect of Dietary Sodium Reduction in Kidney Disease Patients with Albuminuria; SUPER). In this project, participants with chronic kidney disease and albuminuria are being randomized to a behavioral modification program designed to reduce dietary sodium intake to ≤2,300 mg/day or to usual care and test the effect of this intervention on change in albumin-to-creatinine ratio from baseline to 24-weeks.

4. Study Design and Organization

4.A. Overview of trial design

We propose to conduct a *randomized controlled trial* aimed at testing the effect of a behavioral intervention that promotes *reduced carbohydrate intake and increased consumption of heart-healthy unsaturated fats and protein* in patients with HbA1c 6.0–6.9% (*Figure 1*). We plan to recruit 112 individuals with elevated HbA1c and randomly assign them to a moderately intensive intervention on diet pattern,[93] using well-established methods for behavioral modification, or to usual diet. We will compare the difference between the active intervention and control groups for change in HbA1c from baseline to 6 months of follow-up (primary outcome). We will assess four major secondary outcomes: fasting plasma glucose, systolic BP, total-to-HDL cholesterol ratio, and body weight. In addition, we will look at several other exploratory outcomes: insulin and HOMA-IR, diastolic BP, waist circumference, and estimated CVD risk, as estimated by the 2013 ACC/AHA risk equation. The main trial components are summarized in *Table 1*.

Table 1. Study Overview

Study Design	Randomized controlled trial
Study Participants	112 participants with elevated HbA1c (6.0–6.9%)
Intervention Group (n=56)	Behavioral modification to reduce carbohydrate consumption and promote consumption of foods such as non-starchy vegetables, plant-based unsaturated oils, nuts and seeds, avocados, eggs, seafood, poultry, lean meat and tofu. We will recommend moderate consumption of cheese, unsweetened Greek yogurt, and low-carb milk. If consuming higher-carbohydrate items, recommend legumes, fruits, (limited) whole grains. Target <40 g net carbohydrates per day for first 3 months and will have a goal of <40 net g carbohydrates per day; <60 g net carbohydrates per day for months 4 onwards.
Usual Diet Group (n=56)	No dietary intervention. To encourage continued participation in the trial, we will offer optional monthly educational sessions on unrelated topics, such as retirement planning or health insurance
Outcome Measures	Primary: HbA1c; Secondary: fasting plasma glucose, systolic BP, total-to-HDL-cholesterol ratio, and body weight

4.B. Study outcomes

4.B.1. Primary outcome: The *primary outcome will be the difference between the intervention group and the usual diet group for change in HbA1c* from baseline to 6 months. Changes in HbA1c will be calculated from blood samples taken at baseline and 6 months.

4.B.2. Secondary outcomes: Secondary outcomes will include changes from baseline to 6 months of *fasting plasma glucose, systolic BP, total-to-HDL cholesterol ratio, and body weight.,*
4.B.3. Other outcomes: We will also study the effect of the intervention on the following outcomes: insulin and homeostasis model assessment of insulin resistance (HOMA-IR); diastolic BP; waist circumference; total-to-HDL-cholesterol ratio; and estimated CVD risk.

4.C Study Timeline

Table 2 shows the timeline, which will be followed in the implementation of study-related activities, such as publications and submission of an R01 grant application.

Table 2. Study Timeline

	Year 1		Year 2		Year 3	
Develop MOP, intervention program, study forms, and pilot testing	X	X				
Certification and training of staff	X					
Recruitment		X	X			
Intervention and data collection, measurement, entry, cleaning		X	X	X	X	
Data quality control and analysis						X
Publications	X	X	X	X	X	X
R01 grant submissions, resubmissions				X	X	X

The first 6 months will focus on development of manual of operations (MOP), intervention program, study forms, study protocol, Institutional Review Board (IRB) approval, and pilot testing of the intervention program. It will also involve staff training and certification. During the last half of year 1, recruitment, intervention, and data collection will commence. During the last 3 months of year 3, data quality control and analysis will be carried out. Throughout the study period, the JFI will publish peer-reviewed manuscripts from ongoing Tulane University research projects.

6. Study Participants

6.A. Eligibility criteria

Inclusion criteria

- Men or women ages 40 to 70 years: aim to recruit 50% women
- Any race or ethnicity: aim to recruit 40% ethnic minorities (i.e., African American and Hispanic), close to population in the greater New Orleans area[94]
- HbA1c 6.0–6.9%
- Willing and able to provide informed consent

Exclusion criteria

- Diagnosed type 1 diabetes mellitus based on patient self-report
- Use of agents affecting glycemic control (medications for diabetes, oral glucocorticoids) within the past three months prior to enrollment based on patient self-report
- Medical condition in which low-carbohydrate diet may not be advised
    - o Estimated glomerular filtration rate (eGFR) ≤45 mL/min/1.73 m^2, which is close to the 5th percentile of eGFR among non-diseased individuals of 70 years of age[95]
    - o Self-report of liver disease due to hepatitis or alcohol; osteoporosis; untreated thyroid disease; gout; or cancer (other than non-melanoma skin cancer) requiring treatment in the past year, unless prognosis is excellent
- Factors that may affect HbA1c:
    - o Hemoglobin <11 mg/dL (cutpoint for moderate-to-severe anemia, which could lead to falsely elevated or lowered HbA1c)[96]

- Recent blood donation or blood transfusion (self-report, past 4 months)
- Human Immunodeficiency Virus (self-report)[97]

- Allergies to nuts
- For women, current pregnancy, breastfeeding, or plans to become pregnant during the study
- Consumption of ≥21 alcoholic drinks per week or consumption of ≥6 drinks per occasion
- For the CGM collection at the end of the study period only: known allergy to adhesives or other products involved with CGM use (e.g., skin disinfectants)
- Current or planned residence making it difficult to meet trial requirements (due to distance from study site and/or challenges regularly traveling to site)
- Current participation in another lifestyle intervention trial or a pharmaceutical trial
- Participation of another household member in the study; employees or persons living with employees of the study
- Other concerns regarding ability to meet trial requirements, at the discretion of the principal investigator or study coordinator

7. Recruitment and Randomization

7.A. Recruitment

We will recruit study participants from the greater New Orleans area. The initial focus of our recruitment will be mass mailing of individuals in the greater New Orleans area and recruitment through placing flyers and brochures in the community. If community-based recruitment is not successful or cost-effective, we will recruit out-patients from the Ochsner Health System and Tulane Medical Center (TMC), from which our group has prior experience recruiting patients. For this recruitment, we will focus on electronic searches of medical records and laboratory databases. We will also carry out targeted solicitation of referrals from physicians who care for patients likely to meet our inclusion and exclusion criteria.

In addition to this recruitment plan, we have several contingency plans: (1) extending recruitment to other medical centers in the New Orleans area, including the Veterans Affairs Medical Center (VAMC), East Jefferson General Hospital, and West Jefferson Medical Center, with whom Tulane has well-established relationships; (2) TV/radio advertisements; (3) arrange a news story about the study in local TV newscasts, radio talk programs, and/or print media; and (4) conduct outreach at local community events and churches. We have ample experience with each of these approaches.

Based on U.S. Census Bureau 2016 population estimates, the metro New Orleans area population was approximately 35% African American or black and 9% Hispanic.[94] Given our prior experience and population distribution in this region, we plan to recruit a distribution of study participants with 40% ethnic minorities.

7.A.1. Mass mailing, placement of brochures/flyers in community, TV/radio/billboard advertisements: Community recruitment approaches include: mass mailing individuals who live in the New Orleans area; brochures/flyers in the community; emailing the brochure; TV, radio, poster, or billboard advertisements in the New Orleans area. Interested individuals will be pre-screened; those at elevated risk for prediabetes or T2D, i.e. individuals who are obese or overweight and/or have a sufficiently high score from the American Diabetes Association's Diabetes Risk Assessment Questionnaire will be invited into the clinic for a screening visit.

7.A.2. Database searches and targeted mailing: We will request IRB approval for a waiver for HIPAA authorization to conduct database searching and hospital chart reviews for identification of potentially eligible study participants. After identifying a potentially eligible patient, we will

send this individual an invitation letter signed by the investigators and/or the individual's personal physician and brochure via mail or email. Individuals who express interest in the study will have a pre-screening telephone interview for confirmation of potential eligibility. Those still interested and potentially eligible for enrollment will be scheduled for a visit at the COBRE study clinic.

7.A.3. Referrals and Local Partnerships: In addition to local physician referrals, we will explore holding outreach community events in the New Orleans area. Local community partners may post information about the study on their social media platforms. We may also identify potentially interested volunteers from a Tulane volunteer recruitment registry. We will also consider recruiting potentially eligible individuals who were screened or participated in other clinical studies in our clinic and have expressed interest in being contacted about future research projects. Potential participants referred by their physician or community partners will have a pre-screening telephone interview to confirm potential eligibility. Those who are still interested and potentially eligible for the study will be scheduled for a screening visit.

7.B. Randomization

Once eligibility has been confirmed, the study coordinator will ensure that all key variables have been entered into the trial-specific database. Additionally, the study coordinator will ensure any outliers have been recognized and reconciled, and will ensure receipt of informed consent prior to enrolling the volunteer in the study as a trial participant. Eligible participants will be *randomly assigned to the behavioral intervention or the usual diet group in a 1:1 allocation ratio*. Randomization will be stratified by sex, *using a random block group size (4, 6) to maintain balance among groups over time*. A statistician from the Methodology/Biostatistics Unit within the COBRE Clinical Research Core, who is not directly involved in the study and is not located at the clinic, will conduct the randomization. After ascertaining eligibility, clinical personnel will telephone the Methodology/Biostatistics Unit to obtain a randomization assignment. All randomization assignments will be created by a computer program and only accessible to the Staff at the Methodology/Biostatistics Unit. Once randomization assignment has been made, trial participants will be considered to be officially randomized into the study and every effort will be made to obtain complete follow-up information.

7.C. Masking

Clinical and laboratory staff members who assess outcomes will be blinded to the intervention assignment of participants. As this is a dietary intervention trial, study participants, and study staff members who conduct the intervention cannot be blinded to the intervention assignment. Participants will be instructed to avoid sharing knowledge of their treatment assignment with data collection staff members. We will have intervention visits and data collection study visits on different days and in separate locations. Data collectors will also use verbal cues and visual props to remind participants not to disclose treatment assignment. Investigators will be blinded to intervention assignment.

7.D. Safety Considerations

Participants with HbA1c >7.9% will be referred to a clinician for follow-up[98], though we will continue to collect data on these participants.

8. Dietary Intervention Program

8.A. Low-carbohydrate dietary intervention group

Participants randomized to the low-carbohydrate intervention group will receive counseling

aimed at reducing carbohydrate consumption. Phase I (*Go Low*) will occur for the first 3 months, with a goal of <40 g digestible carbohydrates per day. Key recommended foods will include: non-starchy vegetables, fish, poultry, lean meat, eggs, olive oil and other plant-based unsaturated oils, unsweetened/unsalted nuts and seeds, nut butters, and avocados. We will recommend moderate consumption of cheese and unsweetened Greek yogurt and low-carbohydrate milk. During Phase I, we will recommend limiting or avoiding other dairy, fruits, legumes, beans, and grains. Phase II (*Keep it Low*) will run from months 4 through 6. The net carbohydrate goal for this phase will be <40 g to <60 g, with the objective to choose a target that is as low as possible (e.g., as close to <40 g as possible). At 3 months, we expect different participants will have different levels of intake, with some consuming <40 g of net carbohydrates and others consuming levels higher than 60 g net carbohydrates per day. We have designed the intervention to accommodate various scenarios, with the goal of achieving as low of an intake as possible. For participants who consume >60 g per day, we will recommend they aim to either reduce or maintain carbohydrate intake.

At a brief check-in at 3-months, if participants are satisfied with their current intake, we will instruct them to stay at this intake level (though will recommend they try to reduce intake, if possible, if they are eating >60 net grams of carbohydrates or have not yet reached the study goal of <40 net grams of carbohydrates). If they prefer to make modifications, we will provide guidance on how to modify carbohydrate intake (either decrease or increase). If they choose to add back carbohydrates, we will recommend that participants add 5 grams of carbohydrates for two weeks at a time (e.g., from 40 to 45 g for 2 weeks), for a maximum total of <60 g net carbohydrates. If they choose to further reduce carbohydrate intake, we will provide guidance on this as well. As they modify carbohydrate intake, we will have them monitor their satisfaction with their diet and energy levels. If adding back carbohydrates, we will recommend participants add back in foods like fruit (preferably berries), legumes, beans, and dairy, while still aiming to keep total carbohydrate intake as low as possible (e.g., as close to <40 net grams as able).

Materials: We will develop a handbook that will be given to participants. This will contain diet guidelines, recipes, meal planners, shopping lists, and guides for counting carbohydrates and reading nutrition labels.

Meals: Participants will select their own menus and prepare or buy their meals according to the guidelines. We will provide participants with supplemental low-carbohydrate foods, such as nuts, olive oil, and other products, to help facilitate their compliance.

Counseling: Sessions and materials will focus on instructing participants to reduce digestible carbohydrate consumption by increasing consumption of protein and fat, with a particular emphasis on increased consumption of heart-healthy unsaturated fats. After randomization, participants will begin the 3-month *Go Low* phase consisting of individual counseling sessions each week for the first four weeks, followed by 4 group sessions held every other week with phone follow-ups occurring in between group sessions. During the *Keep it Low* phase, months 4 through 6, participants will attend 3 monthly group sessions and have 3 telephone follow-ups. There will be a total of 11 in-person sessions and 7 telephone follow-ups over the full study period. Sessions will be approximately one hour and phone calls will be no longer than thirty minutes. These sessions will consist of diet instruction and supportive counseling, addressing results from 24-hour dietary recalls, teaching self-monitoring and individual diet goal changes, and provide suggestions for replacing usual foods containing carbohydrates. Study materials will be modified from materials used in the successful MACRO study. The JFI will work closely with Drs. He and Bazzano to develop the intervention based on the MACRO study.
Carbohydrate goal: In the MACRO study, carried out among individuals from the same study region, a behavioral intervention aimed at reducing digestible carbohydrate intake to <40 g/day led to reductions in weight and improvements in cardiovascular risk factors among individuals

with obesity.[30] Additionally, restriction of carbohydrate intake to <50g/day is sufficient to induce ketosis.[99] Our rationale for having a multi-phased intervention is to allow participants to see how they feel on the more restrictive diet, and see how they respond to small increases in carbohydrate consumption. A similar technique was applied in the DIETFITS published study.[100] The higher target of 60 net grams may be more sustainable long-term and also allows for consumption of healthy higher-carbohydrate foods, such as berries, legumes, beans, and limited whole grains.

8.B. Usual Diet Group

We will provide participants randomized to the usual diet group written information with standard dietary advice. Participants randomized to the usual diet group will not receive ongoing dietary recommendations. Moreover, they will continue to receive clinical care recommendations from their physician(s). To encourage continued participation in the study and minimize the difference in participant contact with study staff, participants will be offered optional monthly educational sessions on topics unrelated to the study intervention, such as retirement planning or health insurance options.

In another effort to encourage continued participation, patients randomized to the usual diet group will be offered a copy of all available intervention materials, as well as an optional one-hour counseling session with the interventionist following their final study visit.

8.C. Physical Activity

At baseline, both groups will receive written information in study materials with standard physical activity recommendations.

8.D. Dietary Adherence

Strategies for measuring compliance: For the low-carbohydrate diet, adherence will be assessed through measurement of urinary ketones at 3-month visit and 6-month TV. Study staff will make all reasonable effort to maintain adherence of participants to the visit schedule and diet intervention. Walnut consumption will be tracked throughout the study in the low-carbohydrate diet group.

Strategies for improving compliance:

- At the screening visit, we will ask participants who does most of the cooking in their households and how often they eat outside of the home. Results from the screening visit will help to select a group of cooperative study participants.
- Visits will be scheduled at the participants' convenience and to minimize waiting time and trips to the clinic; reminder calls, texts or emails (chosen based on participant preference) will also be made prior to clinic visits.
- We will hire enthusiastic staff who have good interpersonal skills.
- Participants will receive $40 gift cards for use at food stores or retailers when they complete each study visit, for a maximum of $120 over the study period.
- Participants will receive $25 gift cards when they return the stool specimens, for a maximum of $75 over the study period.
- Participants will receive a $25 gift card when they return the continuous glucose monitor, for a maximum of $25 over the study period.
- Participants will be offered small gifts (such as pens or magnets with study logo) to encourage compliance and adherence to the study protocol.
- Participants will be provided with recipes, shopping lists, and food samples for the intervention diet.

9. Safety of Dietary Intervention

On the low-carbohydrate diet, participants will be encouraged to primarily focus on replacing carbohydrates (particularly highly refined carbohydrates) with foods such as non-starchy vegetables, olive oil and other plant-based unsaturated oils, nuts and seeds, avocados, eggs, seafood, tofu, poultry, and lean meat. Consumption of more unsaturated fats, protein, and fiber will be recommended. Participants will not be encouraged to increase consumption of saturated fats and will be advised to avoid consumption of trans fats.

In animal studies, a high-protein diet has decreased renal function[101] In the MACRO study, on average, participants tended to increase consumption of fats, rather than protein.[30] The safety and tolerability of low-carbohydrate diets have previously been assessed in many studies, including MACRO.[30] A number of studies, including some in individuals with T2D, have found no clinically significant increases in estimated glomerular filtration rates,[33,38–40] microalbuminuria,[102] albumin excretion rate,[33,38] urinary albumin,[38] serum creatinine,[38,40,102–107] albumin,[40] or uric acid levels.[103–106] A small study of a very low-carbohydrate diet found significant increase in uric acid excretion, but no significant change in 24-hour urine creatinine clearance.[106]

Study staff will carefully monitor participants' BP, and HbA1c more often than what would occur in routine healthcare visits. A Data Safety and Monitoring Board (DSMB) consisting of an independent group of experts in areas such as biostatistics, nutrition medicine, general internal medicine, and endocrinology has been formed. The board will regularly review trial progress, including unblinded interim results, and can recommend that the trial prematurely end if participants are at risk or if it becomes clear that the trial will not be able to yield statistically significant results.

10. Data Collection

10.A. Study visits

After the initial pre-screening interview, in which data on medications and medical conditions will be collected, there will be one screening visit, one randomization visit, a follow-up study visit at 3 months and a termination visit (TV) at 6 months. *Table 3* summarizes study procedures and data collected at each study visit.

Table 3. Study visit schedule and data collection

	Pre-screen	SV	RZ	3 month	6 month TV
Pre-screen eligibility	X				
Informed consent		X			
Compliance form		X			
Eligibility confirmation			X		
Randomization			X		
Demographics		X			
Medical history questionnaire			X	X	X
Medications		X	X	X	X
Lifestyle (alcohol, cigarette smoking)			X	X	X
Physical activity			X	X	X
24-hour diet recall*			X	X	X
3 BP measurements		X	X	X	X
Body weight			X	X	X
Height			X		
Waist circumference			X	X	X
Spot urine test			X	X	X
Blood samples		X	X	X	X
Stool specimen			X	X	X
Side-effects assessment			X	X	X
Nut Consumption					X
CGM**					X

*Two 24-hour dietary recalls will be collected; one should cover a weekend and the other a weekday.
**The CGM will be worn for up to 14 days prior to the 6-month TV and returned at the TV or by mail. Participants will come in for a 6-month CGM visit (prior to the 6-month TV) to have the CGM inserted.

10.A.1. Screening/baseline visits:

Participants will be pre-screened by phone or in-person by study staff to confirm eligibility for the Screening Visit. An approved questionnaire will be used to ensure the patient is eligible to attend the in-clinic screening visit, and the patient will be scheduled thereafter.

At the screening visit (SV), participants will provide informed consent for the screening and main study. Study staff will schedule the SV and randomization (RZ) visit at least 2 days apart. If a participant is eligible based on the screening visit, he or she should be scheduled for the RZ once a sufficient group of eligible individuals has been recruited. Participants should be scheduled for the RZ at least 2 days and no more than two weeks after the screening visit.

At the screening visit, we will collect information on inclusion/exclusion criteria, medication use, and demographics. At this visit, we will also have participants complete a compliance form,

to assess their ability to complete the study and attend all intervention and study visits. We will also measure blood pressure, and take blood samples. Women will be asked if they are postmenopausal or if they had surgical sterilization (such as a tubal ligation). If not, they will be tested for pregnancy through a spot urine test; pregnant women will be excluded from the study. Blood samples will be collected for measurement of HbA1c, and, if not available from medical records in the past 3 months, serum creatinine and hemoglobin. These blood samples will be used for excluding individuals with HbA1c <6.0 or >6.9, eGFR ≤45 mL/min/1.73 m^2, or hemoglobin <11 mg/dL.

Before the start of the intervention, participants will complete two 24-hour dietary recalls. At least one (and potentially both) 24-h recalls will be collected by phone. At the RZ, participants will learn about the randomization process. After requirements for eligibility have been satisfied, participants will formally be enrolled into the study and randomized to either the active intervention or control group. We will collect fasting blood samples for later measurement of glucose, insulin and lipids and other potential future measurements. At this visit, we will measure blood pressure, body weight, height, and waist circumference. We will collect information on medical history, lifestyle risk factors, and physical activity. A spot urine sample will be collected to measure ketones and other markers and participants will be asked to collect a stool specimen for potential use in future microbiome studies.

10.A.2. Follow-up visits and final visit: A follow-up visit will occur at 3 months after randomization. A final visit will occur at 6-months post-randomization. These visits should be scheduled as close as possible to the 3-, or 6-months after randomization. At each visit, study staff will conduct dietary recalls and anthropometric measures, take 3 BP measurements, collect a fasting blood sample to measure HbA1c. At least one (and potentially both) 24-h recalls will be collected by phone. We will also collect samples for later measurement of glucose, insulin, lipid profile, and other potential future measurements. A spot urine sample will be collected to measure ketones and other markers. Participants will be asked to collect a stool specimen for potential use in future microbiome studies. Information about participants' nut consumption over the past 6 months will be collected at the 6-month TV. At study termination, we will mail participants and/or their physicians a summary of trial results and a record of participant BP, anthropometric measures, and laboratory data from the trial period. Throughout the study period, we will provide participants with their blood pressure and weight. We will request that, if possible, a participant who is prescribed a diabetes medication during the course of the study comes into the clinic for an additional separate supplemental visit for a fasting blood sample collection prior to starting the medication. Maintaining follow-up of all randomized participants is of utmost importance to ensure data integrity. In the event that a participant moves during the study we may request that the participant provides a fasting blood sample at a local LabCorp facility (this will be described on the informed consent form).

10.A.3. CGM 6-month visit: Individuals who are willing to wear the CGM prior to their 6-month termination visit will come to the research clinic 2 weeks (±1 week) before their 6-month termination visit for their CGM visit. At this time, they will have a factory-calibrated CGM inserted just under the skin of the upper arm and activated.[27] If the CGM falls off of a participant prior to 14 days of wear, we may request that the participant (if willing) returns to the clinic for the CGM to be re-inserted. The CGM will be returned at the 6-month TV, or by mail.

10.B Study measurements

Measurements obtained for this study will be based on those used in other clinical trials and epidemiologic studies. We are familiar with strengths and limitations of such measurements and the importance of staff training and certification and use of stringent quality control procedures.

10.B.1. Questionnaires: Study staff will administer a *medical history questionnaire* at the RZ, 3-month visit and 6-month TV to assess medical conditions that would necessitate changes in therapy or medical management beyond this study's scope. At the SV, *demographic factors* will be collected. At the SV, RZ, 3-month and 6-month TV, we will also administer *medication tracking questionnaires*. At RZ, 3-month, and 6-month TV, we will collect health behavior and physical activity data.

10.B.2. Dietary measurements: Two 24-hour dietary recalls will be obtained from participants at baseline. At or close to both the 3-month and 6-month TV, two more 24-hour dietary recalls will be collected. The second sets of two recalls will be collected as closely as possible to 3- or 6-month dates after randomization, with a goal of being within 2 weeks of the respective follow-up period. For each time point, one recall should reflect week day consumption, while the other should reflect weekend day consumption and the two recalls should be at least 2 days apart from each other. We will use computer software (Nutrition Data System for Research [NDS-R]) and food models used in previous and ongoing studies. Recalls will be conducted by a study staff member who has been certified in the use of NDS-R. Food composition tables of the NDS-R will be used to calculate dietary nutrient intakes. The baseline dietary assessment will be done to characterize individual dietary intake of carbohydrates and other macronutrients. During the intervention, repeated measurements will be used to monitor short-term changes in mean dietary nutrient intake between the intervention and usual diet groups.

10.B.3. Assessment and management of adverse effects: At RZ, 3-month visit, and 6-month TV, we will administer *symptom questionnaires*, which will consist of a checklist to identify participant complaints. Prior studies of low-carbohydrate dietary interventions suggest that participants may experience a range of adverse effects, including constipation, halitosis, fatigue, headache, thirst, polyuria, and nausea. Severe adverse events will be promptly reported to the DSMB. Management of adverse effects will be based on the protection and safety of the participant. If severe adverse effects are reported by a participant, carbohydrate intake will be examined and altered in order to mitigate the adverse effect. In any extreme cases, participants will be instructed to stop the intervention. As this is a behavioral intervention, adverse effects should be rare.

10.B.4. BP and anthropometric measures: BP will be measured 3 times at the screening visit and at each follow-up visit. To minimize random error, averages of the measurements at the screening and randomization visits will be taken. BP will be measured by a standard automated blood pressure measurement device (OMRON HEM-907 XL Professional Digital Blood Pressure Monitor) and performed by experienced staff, trained and certified in BP measurement. Prior to BP measurements, participants will be asked to rest quietly in a seated position with their feet supported for at least 5 minutes; staff members will use an appropriate cuff size over a bare arm. Trained and certified staff will measure weight using a calibrated balance beam scale at the screening visits, and follow-up visits. Weight and height will be measured at the RZ, and weight will also be measured at the subsequent visits.

10.B.5. Blood samples: For SV, participants will not need to fast. For RZ, 3-month visit, and 6-month TV, participants will be instructed to fast for 12 hours prior to blood sample collection. At SV, blood samples will be collected to measure HbA1c, hemoglobin, and serum creatinine. At RZ, fasting blood samples will be collected and stored as serum/plasma, whole blood aliquots and buffy coats at -80°C for future analyses, including measurement of glucose, insulin and lipids, and potential future metabolomics or genetic analyses. At 3-month visit and 6-month TV,

we will collect fasting blood samples to measure HbA1c and will also store additional serum/plasma, whole blood aliquots and buffy coats at -80°C for future analyses, including measurement of glucose, insulin and lipids, and potential future metabolomics or genetic analyses. Details of the blood sample collection are shown in *Table 4*. In the event that a participant moves, we may request that they collect a fasting blood sample at a local LabCorp and we will measure HbA1c, glucose, insulin, and lipids at the 3 and 6-month visits. HOMA-IR will be calculated from fasting glucose and insulin.

Table 4. Study visit schedule for specimen collection

	SV	RZ	3 month	6 month TV
HbA1c	X		X	X
Serum creatinine	X			
Hemoglobin	X			
Storage of aliquots and buffy coats (for measurement of glucose, insulin, lipids, other potential future measurements)		X	X	X
Urine sample for pregnancy	X			
Urine sample for ketones and other markers		X	X	X
Stool sample		X	X	X
CGM				X

10.B.6. Urine collection: Study staff will collect spot urine specimens at RZ, interim visit, and the final visit to measure ketones.

10.B.7. Stool Specimen Collection: Stool samples will be collected from all eligible and willing trial participants during the RZ, 3-month, and 6-month TV, following the protocol set forth by the Human Microbiome Project.[108,109] Participants will be given a stool collection kit, which includes the specimen collection container, a shipper for home collection, and a form to record the timing and pertinent information related to specimen collection. Participants will be instructed to return the kit to the clinic within two days of collection (in-person or via shipping), at which time stool samples are stored at -80°C until analyses. Kits and instructions for collection will be reviewed by study staff with the participant at the required visits.

10.B.8. CGM at 6 months: Approximately 6 months before their 6-month termination visit, participants willing to wear the CGM will come to the clinic to have their CGM inserted. This CGM has been found to be accurate compared with capillary blood glucose reference values over 14 days of wear.[27] This CGM records interstitial glucose every 15 minutes throughout the day for a period of up to 14 days. The sensor uses a thin, flexible filament inserted just under the skin to measure glucose and is read through near-field communication by touching the reader to the

sensor. For quality control of the CGM data, a random subset of participants who wear the CGM will be asked to wear two CGMs at the same time, one on each arm. Prior to insertion, one arm will be designated as the arm used for data collection and the other arm as a duplicate.

11. Quality Assurance and Quality Control

Throughout the study, we will implement rigorous quality assurance (QA) and quality control (QC) measures. We describe some of these approaches here.

11.A. Manual of procedures

We will develop a manual of procedures (MOP) based on the MOP successfully used in the MACRO behavior change clinical trial. The MOP will provide instructions and describe procedures for staff training and certification, participant recruitment, intervention counseling, study form completion and procedures, data entry, safety measures (e.g., methods for emergency participant/primary care physician contact).

11.B. Training and certification

Trial personnel will all be required to participate in a training session before the start of any trial procedures or measurements. Training will include particular attention to core trial elements, such as recruitment, retention, intervention, completeness and quality of data acquisition (e.g., blinding procedures for the data collectors), and data entry. Separately, intervention staff will meet and receive detailed instruction on trial approaches to group and individual behavior change counseling and procedures that ensure data collectors are blinded to treatment assignment. Throughout the trial, periodic re-training/certification sessions will be conducted to ensure all trial-related procedures are being applied in a standard manner. Investigators will also monitor all aspects of study performance to ensure study goal achievement, timeliness and completeness of study visits, completeness and quality of data collection, intervention adherence, and adequacy of the procedures separating data collection from intervention. Study staff and investigators will pay particularly careful attention to the quality of all laboratory measurements, particularly key exposure, outcome, and intervention monitoring variables.

12. Sample Size and Statistical Power

The primary outcome will be the difference in the change of HbA1c between the intervention and usual diet groups from baseline to 6 months of follow-up. In the United States National Health and Nutrition Examination Survey, when sub-setting to the range of HbA1c of 6.0–6.9% among individuals who do not report use of diabetes medication, the cross-sectional standard deviation is 0.24% using data from 2000 through 2016 (n=4117; https://wwwn.cdc.gov/nchs/nhanes/). In studies with 6-month follow-up among individuals with HbA1c 5.7 to 6.4% or other characteristic(s) (e.g, elevated fasting glucose or metabolic syndrome) that put them at elevated risk of type 2 diabetes, the within-person correlation between baseline and follow-up HbA1c was estimated to be between 0.5 and 0.84.[110–113] As all participants in our study will be within a specific range of HbA1c at baseline, we expect this may lead to smaller within-person correlation of HbA1c over time than studies that do not restrict to a narrow range of HbA1c at baseline. Assuming a within-person correlation of 0.5 and an SD of 0.24% for change in HbA1c, we would need to have 90 individuals to have 85% power to detect an approximate difference in change of HbA1c of 0.15% between the intervention and usual diet groups.

In a follow-up analysis of the DPP, a 0.15% decrement in HbA1c at 6 months was associated with a 16% lower risk of incident T2D, adjusting for treatment arm, age, sex, race/ethnicity, household income, and baseline HbA1c.[115] In a pooled analysis from the Atherosclerosis Risk in Communities and Framingham Heart Studies, a 0.15% higher HbA1c was associated with an 16% higher risk of T2D over 20 years, a 30% higher risk over 8 years, and a 13% higher risk after 8 years, after adjusting for age, sex, fasting glucose, triglycerides, HDL, family history of T2D, SBP, and BMI.[116] Additionally, in EPIC-Norfork, a difference of HbA1c of 0.15% was associated with approximately a 5% higher risk of CHD among those free of CHD, stroke, diabetes, and with HbA1c <7.0%.[117]

Table 5. Minimum detectable differences

	SD	Detectable Difference
HbA1c, %	0.24	0.15
Fasting glucose, mg/dL	12.5	8.0
Fasting insulin, mU/mL	7.0	4.5
HOMA-IR	2.0	1.3
SBP (mmHg)	4.2	2.7
DBP (mmHg)	3.0	1.9
Total/HDL ratio	1.2	0.77
Body weight (kg)	6.6	4.2
Waist circumference (cm)	8.0	5.1
Estimated CVD risk (%)*	6.7	4.8

*10-y CVD risk, among estimated 85% or 76 participants free of CVD

In the DPP study, HbA1c was 0.15% lower among those in the lifestyle intervention arm than in the control arm at 6-months follow-up; HbA1c at study commencement was 5.9% and levels increased in the control arm and decreased in the behavioral intervention arm.[22] In a small behavioral intervention study (n=34) of a very low-carbohydrate ketogenic diet compared with a moderate carbohydrate, calorie-restricted, low-fat diet among individuals with type 2 diabetes or prediabetes (HbA1c >6.0%) and elevated body weight (BMI >25), mean HbA1c decreased from 6.6% to 6.1% in the very low-carbohydrate group and from 6.9% to 6.7% in the moderate carbohydrate group over a 12-month period; additionally, the very low-carbohydrate group had greater reductions in glucose-lowering medications.[37]

Detectable differences are listed for HbA1c and secondary outcomes in *Table 5*, based on data from prior studies.[30,53,56,118] These were calculated for unpaired t-tests, power of 85%, two-sided alpha of 0.05, and assuming 80% follow-up of 112 participants, which would yield an estimated 90 participants.

13. Data Management and Analysis

13.A. Data management

Personnel will double-enter all study data. Prior to entering data, the study coordinator will conduct quality control on all study forms, study measures, and laboratory data. Data will be cleaned and logically checked.

13.B. Data analysis plan

The data will be analyzed using *intention to treat*; this involves comparing the primary and secondary outcomes between participants based on randomization assignment.

13.B.1. Exploratory data analysis: We will express group data for variables with a normal distribution as mean ± standard deviation and for variables not normally distributed as median and interquartile ranges (or will log-transform the variables to obtain a normal distribution). We will compare means and percentages of baseline variables across the intervention and usual diet group to assess similarity of these groups at randomization.

13.B.2. Primary, secondary, and exploratory outcomes: We will test the primary study hypothesis that there is a greater reduction in HbA1c from baseline to 6 months in the intervention group than in the usual diet group. To do this, we will use a mixed-effects linear regression model, to test the null hypothesis that there is no difference in change between baseline and 6-month HbA1c for the intervention group compared with the usual diet group (Specific Aim 1). To do this, we will use the following model:

$$Y_{i,j,k}|T = b_0 + b_1G_i + b_2T_{3\text{-months}} + b_3T_{6\text{-months}} + b_4G_iT_{3\text{-months}} + b_5G_iT_{6\text{-months}} + a_{0i,j} + e_{i,j,k}$$

where $Y_{i,j,k}$ is HbA1c for the jth participant (P) nested in the ith intervention group (G) at time T_k (baseline, 3 months, and 6 months). This model includes a random intercept ($a_{0i,j}$) and error term $e_{i,j,k}$. The interaction term ($b_5G_iT_{6\text{-months}}$) allows for an interaction between group and $T_{6\text{-months}}$, which is equal to 0 for baseline and 3-months and 1 for 6-months. Rejection of the null hypothesis (b_5=0) indicates there is a significant difference in change of HbA1c from baseline to 6 months between the intervention and control groups. In this model, participants are assumed to be random effects and intervention group, time, and the interaction terms are fixed effects.

We will use a similar approach to test the difference in the change between baseline and 6-month values of secondary outcomes (Specific Aim 2) and exploratory outcomes. In exploratory analyses, we will also test the difference in the change between baseline and 3-month values of outcomes (HbA1c, fasting glucose, systolic blood pressure, total-to-HDL cholesterol, body weight, insulin, HOMA-IR, diastolic blood pressure, waist circumference, and estimated CVD risk). We will also explore the use of mixed effects models to assess overall net change in HbA1c (by treating time as a linear variable) and other outcomes over the full study period. For each outcome, we will test modeling assumptions and will consider transformation of outcomes, if needed, to meet modeling assumptions. As the mixed-effects model assumes missing at random, we will also perform sensitivity analyses using Markov-chain Monte Carlo techniques to impute missing values of the outcomes, using covariates for this imputation and will compare results from this analysis with our primary findings.

15. Anticipated Challenges

Recruitment: Many clinical trials have challenges recruiting sufficient numbers of volunteers who meet the trial inclusion and exclusion criteria and demonstrate a high level of adherence to trial protocol requirements. Fortunately, the investigators are very experienced with clinical trial recruitment, and have several contingency options that can be used to meet unanticipated shortfall in recruitment.

Intervention: The investigators are aware of and understand challenges related to achieving and maintaining compliance with a behavioral intervention. The investigators have much experience and success in behavioral interventions, with recent success of a behavioral intervention aimed at reducing carbohydrate intake among participants from the New Orleans area. The JFI will collaborate with Drs. He and Bazzano to develop the intervention program based on the MACRO study and will assess potential barriers to implementation and intervention adherence (such as financial concerns and range of dietary patterns of study participants) during the planning and pilot phase of the study.

Adherence: Though adherence and follow-up are also main challenges related to clinical trials, the investigators have an outstanding track record for studies that have recruited participants for both clinical and epidemiology studies in the Greater New Orleans area. To encourage adherence, small gifts (such as pens or magnets with study logo) will be offered to all study participants.

16. Protection of Human Subjects

This Human Subjects Research meets the definition of a clinical trial. All investigators will be required to complete the CITI (Collaborative Institutional Training Initiative) program in the ethical use of human participants in research.

16.A. Protection of human subjects from research risks

16.A.1. Risks to the subjects

Human subject involvement and characteristics: We expect to enroll 112 participants, with an equal male to female ratio. We expect to enroll approximately 30 participants who are African-Americans, and another 10 with mixed racial backgrounds, and a few Asian participants given the demographics of the greater New Orleans area. Overall, given the demographics of greater New Orleans, we estimate approximately 10% participants of Hispanic or Latino ethnicity, 50% of them with African-American racial background.

Inclusion criteria

- Men or women ages 40 to 70 years: aim to recruit 50% women
- Any race or ethnicity: aim to recruit 40% ethnic minorities (i.e., African American and Hispanic), close to population in the greater New Orleans area[94]
- HbA1c 6.0–6.9%
- Willing and able to provide informed consent

Exclusion criteria

- Diagnosed type 1 diabetes mellitus based on patient self-report
- Use of agents affecting glycemic control (medications for diabetes, oral glucocorticoids) within the past three months prior to enrollment based on patient self-report
- Medical condition in which low-carbohydrate diet may not be advised
    - Estimated glomerular filtration rate (eGFR) ≤45 mL/min/1.73 m^2, which is close to the 5th percentile of eGFR among non-diseased individuals of 70 years of age[95]
    - Self-report of liver disease due to hepatitis or alcohol; osteoporosis; untreated thyroid disease; gout; or cancer (other than non-melanoma skin cancer) requiring treatment in the past year, unless prognosis is excellent
- Factors that may affect HbA1c:

- Hemoglobin <11 mg/dL (cutpoint for moderate-to-severe anemia, which could lead to falsely elevated or lowered HbA1c)[96]
- Recent blood donation or blood transfusion (self-report, past 4 months)
- Human Immunodeficiency Virus (self-report)[97]

- Allergies to nuts
- For women, current pregnancy, breastfeeding, or plans to become pregnant during the study
- Consumption of ≥21 alcoholic drinks per week or consumption of ≥6 drinks per occasion
- For the CGM collection at the end of the study period only: known allergy to adhesives or other products involved with CGM use (e.g., skin disinfectants)
- Current or planned residence making it difficult to meet trial requirements (due to distance from study site and/or challenges regularly traveling to site)
- Current participation in another lifestyle intervention trial or a pharmaceutical trial
- Participation of another household member in the study; employees or persons living with employees of the study
- Other concerns regarding ability to meet trial requirements, at the discretion of the principal investigator or study coordinator

Sources of materials: Medical information data will be collected from the participant and, after written authorization for the disclosure of protected health information, from participants' medical records. Blood and urine samples will be collected to measure HbA1c, glucose, insulin, lipids, creatinine, ketones, and test for pregnancy. Blood pressure, height, weight, waist circumference and other physical measurements will be obtained.

Potential Risks: Risks associated with venipuncture, the diet intervention, CGM insertion, and collection of confidential medical information are the main risks associated with this study. There is a small risk of bruising and a rare chance of local infection associated with standard venipuncture to collect blood samples. The risks of wearing the CGM are minimal. There is a very low risk for local infection at the skin site where the sensor is inserted into the skin. Possible symptoms associated with the CGM sensor application or the adhesive used to keep the sensor in place include skin irritation or redness, skin inflammation, pain or discomfort, bleeding, bruising, swelling, skin rash, itching, scarring or skin discoloration, or allergic reactions to the sensor needle or adhesives. Sensors may fracture in situ on rare occasions. The dietary intervention has minimal risks. Reduced carbohydrate consumption may lead to adverse effects, including constipation, halitosis, fatigue, headache, thirst, polyuria, and nausea. At RZ, 3- , and 6-month visits, we will administer *symptom questionnaires*, which will consist of a checklist to identify participant complaints. Adverse effects of the low-carbohydrate diet will be assessed at counseling sessions. Severe adverse events will be promptly reported to the DSMB. Management of adverse effects will be based on the protection and safety of the participant. If severe adverse effects are reported by a participant, carbohydrate intake will be examined and altered in order to mitigate the adverse effect. In any extreme cases, participants will be instructed to stop the intervention. Furthermore, there is a potential risk of unauthorized disclosure of medical information. This risk will be minimized by using a strict protocol for data processing and by the appropriate training of all study personnel. Protection against risks is described in more detail below.

16.A.2. Adequacy of protection against risks

Recruitment and Informed Consent: The proposed study will be conducted following strict guidelines for the protection of the rights of human volunteers. The informed consent document will be signed by all participants at the screening/baseline visit. The informed consent form will be developed in the first year of the study and approved by the IRB. The consent form will clearly state the purpose, eligibility criteria, and protocol of the study, the potential benefits and risks of participation, and the subject's right to refuse to participate or withdraw. Prior to signing the informed consent, research staff will review the details of the consent form orally with the participant, and answer any questions that the participant has concerning participation in the study. In the case of potential participants with low or no literacy, the informed consent form will be read to the participant in its entirety and any questions will be answered prior to the participant signing or marking the form. Specifically, the following must be accomplished during the informed consent process:

- The participant must be informed that participation in the study is voluntary and that refusal to participate will involve no penalty or loss of benefits.
- The participant must be informed of the purpose of the study and that it involves research.
- The participant must be informed of any alternative procedures, if applicable.
- The participant must be informed of any reasonably foreseeable risks.
- The participant must be informed of any benefits from the research.
- An outline of safeguards to protect participant confidentiality will be included, as well as an indication of the participant's right to withdraw without penalty. This should be balanced with a discussion of the effect that withdrawals will have on the study, and the responsibility a participant has, within limits, to continue in the study if he or she decides to enroll.
- The participant must be informed of his or her right to have questions answered at any time and whom to contact for answers or in the event of research-related injury.
- The participant must be informed as to whether or not compensation is offered for participation in the study and/or in the event of a medical injury.
- The participant must be informed that he/she will be notified of any significant changes in the protocol that might affect their willingness to continue in the study.

Written authorization for the disclosure of protected health information will be obtained during the consent process on a separate form (HIPPA authorization), if applicable.

Protection against Risk: The protocol and informed consent will be approved by the Institutional Review Boards at Tulane University Health Sciences Center and any other participating institution for recruitment. The study participants will be given contact information for the Study Coordinator and Study PI to answer questions or to report complaints. Patients can withdraw from the study at any time without any effect on their medical care. Standard techniques will be used by trained, certified phlebotomists or study nurses for blood sample collection at the study clinics where emergency medical equipment is available. Confidentiality of all data collected during the study will be maintained through the use of unique, encrypted study identification (ID numbers, rather than patient names, in the study database). These encrypted ID numbers will not be mathematical derivation of the subject's medical record number or any other patient identifier. No information will be disclosed in an individually identifiable form in any type of presentation or publication. There will be password-required access to the computerized file linking the study numbers to patient identifiers at the study clinics. Password-access will be restricted to pertinent research staff only. All study data will be kept in locked file cabinets in locked rooms, accessible only by study staff with permission. All

data transmissions will use HIPPA-compliant password protected system and will be secured through a virtual private network.

16.A.3. Potential benefits of the proposed research to human subjects and others

Study participants may have improved glycemic outcomes, blood pressure, lipid levels, may lose weight and reduce waist circumference, and may have improvement in other risk factors for CVD and development and progression of T2D. Participants will receive multiple blood pressure, weight, waist circumference, and blood tests. If vital signs or lab values are clinically abnormal, participants will be referred to their primary care physician, or if they do not have a primary care physician, one will be assigned through the Tulane University Community Health Center, which is a part of the core clinical facilities. At the end of the study, we will provide the participants' primary care physician with a copy of all the blood pressure, weight, laboratory results, and (if applicable) CGM results.

16.A.4. Importance of knowledge to be gained

Information from this study may contribute to reducing the onset of T2D and the burden of T2D and cardiovascular disease in Louisiana and the general US population. The study has potential for public health impact; results from this study may provide evidence in support of promoting alternative dietary styles (than the most commonly used low-fat diet) among individuals with high T2D risk or with T2D.

16.A.5. Remuneration

For screening visit 2, the two follow-up visits, and the final visits, participants will receive $40 gift cards for use at food stores or retailers (after completing each study visit), for a maximum of $120 over the study period. Participants who return a stool sample will receive $25 gift cards for each stool sample that they return, for a total of $75 for three stool samples. For the CGM 6-month study, participants will receive $25 gift cards for returning the CGM, for a maximum of $25. Participants will also receive free parking for each intervention and study visit.

16.B. Data safety and monitoring plan

A DSMB, which consists of 3 experts who are not otherwise affiliated with the COBRE program, has been formed. Study investigators for this study will prepare accurate and timely data tables and reports for the DSMB meetings. The members of the DSMB will serve in an individual capacity and provide their expertise and recommendations. The primary responsibilities of the DSMB are: 1) periodically review and evaluate the accumulated study data for participant safety, study conduct and progress, and, when appropriate, efficacy, and 2) make recommendations to the NIH concerning the continuation, modification, or termination of the trial. The DSMB considers study-specific data, as well as relevant background knowledge, about the disease, test agent, and patient population under study.

The DSMB is responsible for defining its deliberative processes, including event triggers that would call for an unscheduled review, stopping guidelines, unmasking (unblinding) and voting procedures prior to initiating any data review. The DSMB is also responsible for maintaining the confidentiality of its internal discussions and activities, as well as the contents of reports provided to it.

The DSMB will review each protocol for any major concerns prior to implementation. During the trial, the DSMB should review cumulative study data to evaluate safety, study conduct, and scientific validity and integrity of the trial. As part of this responsibility, DSMB members must be satisfied that the timeliness, completeness, and accuracy of the data submitted to them for

review are sufficient for evaluation of the safety and welfare of study participants. The DSMB should also assess the performance of overall study operations and any other relevant issues, as necessary.

Items reviewed by the DSMB include:

- Interim/cumulative data for evidence of study-related adverse events;
- Interim/cumulative data for evidence of efficacy according to pre-established statistical guidelines, if appropriate;
- Data quality, completeness, and timeliness;
- Adequacy of compliance with goals for recruitment and retention, including those related to the participation of women and minorities;
- Adherence to the protocol;
- Factors that might affect the study outcome or compromise the confidentiality of the trial data (such as protocol violations, unmasking, etc.); and,
- Factors external to the study, such as scientific or therapeutic developments, that may impact participant safety or the ethics of the study.

The DSMB will conclude each review with their recommendations to the COBRE program and NIH/NIGMS as to whether the study should continue without change or be modified or terminated. Recommendations regarding modification of the design and conduct of the study could include:

- Modifications of the study protocol based upon review of the safety data;
- Suspension or early termination of the study or of one or more study arms because of serious concerns about subjects' safety, inadequate performance or rate of enrollment;
- Suspension or early termination of the study or of one or more study arms because study objectives have not been obtained according to pre-established statistical guidelines; and,
- Corrective actions regarding a study when the performance appears unsatisfactory or suspicious.

Confidentiality must always be maintained during all phases of DSMB review and deliberation. Usually, only voting members of the DSMB should have access to interim analyses of outcome data by treatment group. Exceptions may be made when the DSMB deems it appropriate. The reason and to whom the exceptions for access to interim analyses is granted will be documented in the Closed Session Report. DSMB members must maintain strict confidentiality concerning all privileged trial results ever provided to them. The DSMB should review data only by masked study group (such as X vs. Y rather than experimental vs. control) unless or until the DSMB determines that the identities of the groups are necessary for their decision-making. Whenever masked data are presented to the DSMB, the key to the group coding must be available for immediate unmasking.

The DSMB will meet twice per year. The junior faculty investigator will attend these meetings and will be responsible for preparing and presenting up-to-date statistical reports on the progress of their trials. These reports will include data on recruitment, randomization, adherence, as well as statistical tests and special analyses requested by the DSMB. The Administrative Core will be responsible for organizing the annual in-person meeting and semiannual conference calls. They will put together data and materials submitted by junior faculty investigators and deliver them to DSMB members. The COBRE investigators will meet with the DSMB in the Open Session and not be present for the Closed Session.

Bibliography and References Cited

1. The Emerging Risk Factors Collaboration. Diabetes mellitus, fasting blood glucose concentration, and risk of vascular disease: a collaborative meta-analysis of 102 prospective studies. *Lancet*. 2010;375(9733):2215-2222. doi:10.1016/S0140-6736(10)60484-9.
2. Flaxman SR, Bourne RRA, Resnikoff S, et al. Global causes of blindness and distance vision impairment 1990-2020: a systematic review and meta-analysis. *Lancet Glob Heal*. October 2017. doi:10.1016/S2214-109X(17)30393-5.
3. United States Renal Data System. *2016 USRDS Annual Data Report: Epidemiology of Kidney Disease in the United States*. Bethesda, MD: National Institutes of Health, National Institute of Diabetes and Digestive and Kidney Diseases; 2016. https://www.usrds.org/2016/view/Default.aspx.
4. Narres M, Kvitkina T, Claessen H, et al. Incidence of lower extremity amputations in the diabetic compared with the non-diabetic population: A systematic review. *PLoS One*. 2017;12(8):e0182081. doi:10.1371/journal.pone.0182081.
5. Centers for Disease Control and Prevention. *National Diabetes Statistics Report, 2017*. Atlanta, GA: Centers for Disease Control and Prevention, U.S. Dept of Health and Human Services; 2017.
6. Centers for Disease Control and Prevention. *Long-Term Trends in Diabetes*. Atlanta, GA: Centers for Disease Control and Prevention, U.S. Dept of Health and Human Services; 2017.
7. NCD Risk Factor Collaboration (NCD-RisC). Worldwide trends in diabetes since 1980: A pooled analysis of 751 population-based studies with 4.4 million participants. *Lancet*. 2016;387(10027):1513-1530. doi:10.1016/S0140-6736(16)00618-8.
8. American Diabetes Association. Economic costs of diabetes in the U.S. in 2012. *Diabetes Care*. 2013;36(4):1033-1046. doi:10.2337/dc12-2625.
9. Zhuo X, Zhang P, Barker L, Albright A, Thompson TJ, Gregg E. The lifetime cost of diabetes and its implications for diabetes prevention. *Diabetes Care*. 2014;37(9):2557-2564. doi:10.2337/dc13-2484.
10. WHO, International Diabetes Foundation. *Definition and Diagnosis of Diabetes Mellitus and Intermediate Hyperglycaemia: Report of a WHO/IDF Consultation*. Geneva: Geneva: World Health Organization; 2006.
11. The International Expert Committee. International Expert Committee Report on the Role of the A1C Assay in the Diagnosis of Diabetes. *Diabetes Care*. 2009;32(7):1327-1334. doi:10.2337/dc09-9033.
12. American Diabetes Association. Standards of Medical Care in Diabetes - 2017. *Diabetes Care*. 2017;40(Supplement 1):S33-S43. doi:10.2337/dc16-S003.
13. Gerstein HC, Santaguida P, Raina P, et al. Annual incidence and relative risk of diabetes in people with various categories of dysglycemia: A systematic overview and meta-analysis of prospective studies. *Diabetes Res Clin Pract*. 2007;78(3):305-312. doi:10.1016/j.diabres.2007.05.004.
14. Huang Y, Cai X, Mai W, Li M, Hu Y. Association between prediabetes and risk of cardiovascular disease and all cause mortality: systematic review and meta-analysis. *BMJ*. 2016;355:i5953. doi:10.1136/bmj.i5953.
15. Tabák AG, Herder C, Rathmann W, Brunner EJ, Kivimäki M. Prediabetes: A high-risk state for diabetes development. *Lancet*. 2012;379(9833):2279-2290. doi:10.1016/S0140-

6736(12)60283-9.

16. Diabetes Prevention Program Research Group. 10-year follow-up of diabetes incidence and weight loss in the Diabetes Prevention Program Outcomes Study. *Lancet*. 2009;374(9702):1677-1686. doi:10.1016/S0140-6736(09)61457-4.
17. The DREAM (Diabetes REduction Assessment with ramipril and rosiglitazone Medication) Trial Investigators. Effect of rosiglitazone on the frequency of diabetes in patients with impaired glucose tolerance or impaired fasting glucose: a randomised controlled trial. *Lancet*. 2006;368(9541):1096-1105. doi:10.1016/S0140-6736(06)69420-8.
18. Perreault L, Pan Q, Mather KJ, Watson KE, Hamman RF, Kahn SE. Effect of regression from prediabetes to normal glucose regulation on long-term reduction in diabetes risk: Results from the Diabetes Prevention Program Outcomes Study. *Lancet*. 2012;379(9833):2243-2251. doi:10.1016/S0140-6736(12)60525-X.
19. Perreault L, Temprosa M, Mather KJ, et al. Regression from prediabetes to normal glucose regulation is associated with reduction in cardiovascular risk: Results from the diabetes prevention program outcomes study. *Diabetes Care*. 2014;37(9):2622-2631. doi:10.2337/dc14-0656.
20. Popkin BM. Nutrition Transition and the Global Diabetes Epidemic. *Curr Diab Rep*. 2015;15(9). doi:10.1007/s11892-015-0631-4.
21. Vazquez G, Duval S, Jacobs DRJ, Silventoinen K. Comparison of body mass index, waist circumference, and waist/hip ratio in predicting incident diabetes: a meta-analysis. *Epidemiol Rev*. 2007;29:115-128. doi:10.1093/epirev/mxm008.
22. Knowler WC, Barrett-Connor E, Fowler SE, et al. Reduction in the incidence of type 2 diabetes with lifestyle intervention or metformin. *N Engl J Med*. 2002;346(6):393-403. doi:10.1056/NEJMoa012512.
23. Pan XR, Li GW, Hu YH, et al. Effects of diet and exercise in preventing NIDDM in people with impaired glucose tolerance: The Da Qing IGT and diabetes study. *Diabetes Care*. 1997;20(4):537-544. doi:10.2337/diacare.20.4.537.
24. Tuomilehto J, Indstrom J, Eriksson J, Valle T, Hamalainine E, Uusitupa M. Prevention of Type 2 Diabetes Mellitus By Changes in Lifestyle Among Subjects With Impaired Glucose Tolerance. *N Engl J Med*. 2001;344(18):1343-1350. doi:10.1056/NEJM200105033441801.
25. Ramachandran A, Snehalatha C, Mary S, Mukesh B, Bhaskar AD, Vijay V. The Indian Diabetes Prevention Programme shows that lifestyle modification and metformin prevent type 2 diabetes in Asian Indian subjects with impaired glucose tolerance (IDPP-1). *Diabetologia*. 2006;49(2):289-297. doi:10.1007/s00125-005-0097-z.
26. The Diabetes Preventio Program Research Group. The Diabetes Prevention Program. Design and methods for a clinical trial in the prevention of type 2 diabetes. *Diabetes Care*. 1999;22(4):623-634. doi:10.2337/diacare.22.4.623 10.2337/diabetes.48.4.699.
27. Evert AB, Boucher JL, Cypress M, et al. Nutrition therapy recommendations for the management of adults with diabetes. *Diabetes Care*. 2013;36(11):3821-3842. doi:10.2337/dc13-2042.
28. Hu T, Mills KT, Yao L, et al. Effects of low-carbohydrate diets versus low-fat diets on metabolic risk factors: A meta-analysis of randomized controlled clinical trials. *Am J Epidemiol*. 2012;176(SUPPL. 7). doi:10.1093/aje/kws264.
29. Sackner-Bernstein J, Kanter D, Kaul S. Dietary Intervention for Overweight and Obese Adults: Comparison of Low-Carbohydrate and Low-Fat Diets. A Meta-Analysis. *PLoS*

One. 2015;10(10):e0139817. doi:10.1371/journal.pone.0139817.
30. Bazzano LA, Hu T, Reynolds K, et al. Effects of low-carbohydrate and low-fat diets: A randomized trial. *Ann Intern Med*. 2014;161(5):309-318. doi:10.7326/M14-0180.
31. Sawyer L, Gale EAM. Diet, delusion and diabetes. *Diabetologia*. 2009;52(1):1-7. doi:10.1007/s00125-008-1203-9.
32. Snorgaard O, Poulsen GM, Andersen HK, Astrup A. Systematic review and meta-analysis of dietary carbohydrate restriction in patients with type 2 diabetes. *BMJ Open Diabetes Res Care*. 2017;5(1):e000354. doi:10.1136/bmjdrc-2016-000354.
33. Larsen RN, Mann NJ, Maclean E, Shaw JE. The effect of high-protein, low-carbohydrate diets in the treatment of type 2 diabetes: A 12 month randomised controlled trial. *Diabetologia*. 2011;54(4):731-740. doi:10.1007/s00125-010-2027-y.
34. Saslow LR, Kim S, Daubenmier JJ, et al. A randomized pilot trial of a moderate carbohydrate diet compared to a very low carbohydrate diet in overweight or obese individuals with type 2 diabetes mellitus or prediabetes. *PLoS One*. 2014;9(4). doi:10.1371/journal.pone.0091027.
35. Tay J, Luscombe-Marsh ND, Thompson CH, et al. A very low-carbohydrate, low-saturated fat diet for type 2 diabetes management: A randomized trial. *Diabetes Care*. 2014;37(11):2909-2918. doi:10.2337/dc14-0845.
36. Davis NJ, Tomuta N, Schechter C, et al. Comparative Study of the Effects of a 1-Year Dietary Intervention of a Low- Carbohydrate Diet Versus a Low-Fat Diet on Weight and Glycemic Control in Type 2. *Diabetes Care*. 2009;32(7):1147-1152. doi:10.2337/dc08-2108.Clinical.
37. Saslow LR, Daubenmier JJ, Moskowitz JT, et al. Twelve-month outcomes of a randomized trial of a moderate-carbohydrate versus very low-carbohydrate diet in overweight adults with type 2 diabetes mellitus or prediabetes. *Nutr Diabetes*. 2017;7(12):304. doi:10.1038/s41387-017-0006-9.
38. Tay J, Thompson CH, Luscombe-Marsh ND, et al. Effects of an energy-restricted low-carbohydrate, high unsaturated fat/low saturated fat diet versus a high carbohydrate, low fat diet in type 2 diabetes: a 2 year randomized clinical trial. *Diabetes, Obes Metab*. 2017:n/a-n/a. doi:10.1111/dom.13164.
39. Yamada Y, Uchida J, Izumi H, et al. A non-calorie-restricted low-carbohydrate diet is effective as an alternative therapy for patients with type 2 diabetes. *Intern Med*. 2014;53(1):13-19. doi:http://dx.doi.org/10.2169/internalmedicine.53.0861.
40. Mayer SB, Jeffreys AS, Olsen MK, Mcduffie JR, Feinglos MN, Yancy WS. Two diets with different haemoglobin A1c and antiglycaemic medication effects despite similar weight loss in type 2 diabetes. *Diabetes, Obes Metab*. 2014;16(1):90-93. doi:10.1111/dom.12191.
41. Maekawa S, Kawahara T, Nomura R, et al. Retrospective study on the efficacy of a low-carbohydrate diet for impaired glucose tolerance. *Diabetes Metab Syndr Obes*. 2014;7:195-201. doi:10.2147/DMSO.S62681.
42. Davis C, Bryan J, Hodgson J, Murphy K. Definition of the mediterranean diet: A literature review. *Nutrients*. 2015;7(11):9139-9153. doi:10.3390/nu7115459.
43. Sofi F, Macchi C, Abbate R, Gensini GF, Casini A. Mediterranean diet and health status: an updated meta-analysis and a proposal for a literature-based adherence score. *Public Health Nutr*. 2014;17(12):2769-2782. doi:10.1017/S1368980013003169.
44. Jannasch F, Kröger J, Schulze MB. Dietary Patterns and Type 2 Diabetes: A Systematic Literature Review and Meta-Analysis of Prospective Studies. *J Nutr*. 2017;147(6):1174-

1182. doi:10.3945/jn.116.242552.
45. Schwingshackl L, Missbach B, König J, Hoffmann G. Adherence to a Mediterranean diet and risk of diabetes: a systematic review and meta-analysis. *Public Health Nutr*. 2014;18(7):1292–1299. doi:10.1017/S1368980014001542.
46. de Lorgeril M, Salen P, Martin J-L, Monjaud I, Delaye J, Mamelle N. Mediterranean Diet, Traditional Risk Factors, and the Rate of Cardiovascular Complications After Myocardial Infarction : Final Report of the Lyon Diet Heart Study. *Circulation*. 1999;99(6):779-785. doi:10.1161/01.CIR.99.6.779.
47. Estruch R, Ros E, Salas-Salvadó J, et al. Primary Prevention of Cardiovascular Disease with a Mediterranean Diet. *N Engl J Med*. 2013;368(14):1279-1290. doi:10.1056/NEJMoa1200303.
48. Salas-Salvadó J, Bulló M, Estruch R, et al. Prevention of diabetes with mediterranean diets: A subgroup analysis of a randomized trial. *Ann Intern Med*. 2014;160(1):1-10. doi:10.7326/M13-1725.
49. Esposito K, Marfella R, Ciotola M, et al. Effect of a Mediterranean-Style Diet on Endothelial Dysfunction and Markers of Vascular Inflammation in the Metabolic Syndrome. *JAMA*. 2004;292(12):1440. doi:10.1001/jama.292.12.1440.
50. Babio N, Toledo E, Estruch R, et al. Mediterranean diets and metabolic syndrome status in the PREDIMED randomized trial. *CMAJ*. 2014;186(17):E649-E657. doi:10.1503/cmaj.140764.
51. Esposito K, Maiorino MI, Bellastella G, Chiodini P, Panagiotakos D, Giugliano D. A journey into a Mediterranean diet and type 2 diabetes: a systematic review with meta-analyses. *BMJ Open*. 2015;5(8):e008222. doi:10.1136/bmjopen-2015-008222.
52. Micha R, Michas G, Mozaffarian D. Unprocessed red and processed meats and risk of coronary artery disease and type 2 diabetes--an updated review of the evidence. *Curr Atheroscler Rep*. 2012;14(6):515-524. doi:10.1007/s11883-012-0282-8.
53. Esposito K, Maiorino MI, Ciotola M, et al. Effects of a Mediterranean-style diet on the need for antihyperglycemic drug therapy in patients with newly diagnosed type 2 diabetes: A randomized trial. *Ann Intern Med*. 2009;151(5):306-314. doi:10.1097/OGX.0b013e3181e5a159.
54. Esposito K, Maiorino MI, Petrizzo M, Bellastella G, Giugliano D. The effects of a Mediterranean diet on the need for diabetes drugs and remission of newly diagnosed type 2 diabetes: Follow-up of a randomized trial. *Diabetes Care*. 2014;37(7):1824-1830. doi:10.2337/dc13-2899.
55. Elhayany A, Lustman A, Abel R, Attal-Singer J, Vinker S. A low carbohydrate Mediterranean diet improves cardiovascular risk factors and diabetes control among overweight patients with type 2 diabetes mellitus: A 1-year prospective randomized intervention study. *Diabetes, Obes Metab*. 2010;12(3):204-209. doi:10.1111/j.1463-1326.2009.01151.x.
56. Shai I, Schwarzfuchs D, Henkin Y, et al. Weight Loss with a Low-Carbohydrate, Mediterranean, or Low-Fat Diet. *N Engl J Med*. 2008;359(3):229-241. doi:10.1056/NEJMoa0708681.
57. Schwarzfuchs D, Golan R, Shai I. Four-Year Follow-up after Two-Year Dietary Interventions. *N Engl J Med*. 2012;367(14):1373-1374. doi:10.1056/NEJMc1204792.
58. Pan A, Sun Q, Bernstein AM, et al. Red meat consumption and risk of type 2 diabetes: 3 Cohorts of US adults and an updated meta-analysis. *Am J Clin Nutr*. 2011;94(4):1088-1096. doi:10.3945/ajcn.111.018978.

59. Hu EA, Pan A, Malik V, Sun Q. White rice consumption and risk of type 2 diabetes: meta-analysis and systematic review. *BMJ*. 2012;344:e1454. doi:10.1136/bmj.e1454.
60. Wang M, Yu M, Fang L, Hu R-Y. Association between sugar-sweetened beverages and type 2 diabetes: A meta-analysis. *J Diabetes Investig*. 2015;6(3):360-366. doi:10.1111/jdi.12309.
61. Imamura F, O'Connor L, Ye Z, et al. Consumption of sugar sweetened beverages, artificially sweetened beverages, and fruit juice and incidence of type 2 diabetes: systematic review, meta-analysis, and estimation of population attributable fraction. *BMJ*. 2015:h3576. doi:10.1136/bmj.h3576.
62. Carter P, Gray LJ, Troughton J, Khunti K, Davies MJ. Fruit and vegetable intake and incidence of type 2 diabetes mellitus: systematic review and meta-analysis. *BMJ*. 2010;341:c4229. doi:10.1136/bmj.c4229.
63. Cooper AJ, Forouhi NG, Ye Z, et al. Fruit and vegetable intake and type 2 diabetes: EPIC-InterAct prospective study and meta-analysis. *Eur J Clin Nutr*. 2012;66(10):1082-1092. doi:10.1038/ejcn.2012.85.
64. Chen M, Sun Q, Giovannucci E, et al. Dairy consumption and risk of type 2 diabetes: 3 cohorts of US adults and an updated meta-analysis. *BMC Med*. 2014;12(1):215. doi:10.1186/s12916-014-0215-1.
65. Aune D, Norat T, Romundstad P, Vatten LJ. Whole grain and refined grain consumption and the risk of type 2 diabetes: A systematic review and dose-response meta-analysis of cohort studies. *Eur J Epidemiol*. 2013;28(11):845-858. doi:10.1007/s10654-013-9852-5.
66. Ding M, Bhupathiraju SN, Chen M, van Dam RM, Hu FB. Caffeinated and decaffeinated coffee consumption and risk of type 2 diabetes: a systematic review and a dose-response meta-analysis. *Diabetes Care*. 2014;37(2):569-586. doi:10.2337/dc13-1203.
67. Li X-H, Yu F-F, Zhou Y-H, He J. Association between alcohol consumption and the risk of incident type 2 diabetes: a systematic review and dose-response meta-analysis. *Am J Clin Nutr*. 2016;103(3):818-829. doi:10.3945/ajcn.115.114389.
68. Muraki I, Imamura F, Manson JE, et al. Fruit consumption and risk of type 2 diabetes: results from three prospective longitudinal cohort studies. *BMJ*. 2013;347(aug28 1):f5001-f5001. doi:10.1136/bmj.f5001.
69. Upadhyaya S, Banerjee G. Type 2 diabetes and gut microbiome: At the intersection of known and unknown. *Gut Microbes*. 2015;6(2):85-92. doi:10.1080/19490976.2015.1024918.
70. David LA, Maurice CF, Carmody RN, et al. Diet rapidly and reproducibly alters the human gut microbiome. *Nature*. 2014;505(7484):559-563. doi:10.1038/nature12820.
71. Duncan SH, Belenguer A, Holtrop G, Johnstone AM, Flint HJ, Lobley GE. Reduced dietary intake of carbohydrates by obese subjects results in decreased concentrations of butyrate and butyrate-producing bacteria in feces. *Appl Environ Microbiol*. 2007;73(4):1073-1078. doi:10.1128/AEM.02340-06.
72. Dorans KS, Mostofsky E, Levitan EB, Håkansson N, Wolk A, Mittleman MA. Alcohol and Incident Heart Failure among Middle-Aged and Elderly Men: Cohort of Swedish Men. *Circ Hear Fail*. 2015;8(3):422-427. doi:10.1161/CIRCHEARTFAILURE.114.001787.
73. Steinhaus DA, Mostofsky E, Levitan EB, et al. Chocolate intake and incidence of heart failure: Findings from the Cohort of Swedish Men. *Am Heart J*. 2017;183:18-23. doi:10.1016/j.ahj.2016.10.002.
74. Dorans KS, Massa J, Chitnis T, Ascherio A, Munger KL. Physical activity and the incidence of multiple sclerosis. *Neurology*. 2016;87(17):1770-1776.

doi:10.1212/WNL.0000000000003260.

75. Mills KT, Chen J, Yang W, et al. Sodium Excretion and the Risk of Cardiovascular Disease in Patients With Chronic Kidney Disease. *JAMA*. 2016;315(20):2200. doi:10.1001/jama.2016.4447.

76. Chen J, Gu D, Huang J, et al. Metabolic syndrome and salt sensitivity of blood pressure in non-diabetic people in China: a dietary intervention study. *Lancet*. 2009;373(9666):829-835. doi:10.1016/S0140-6736(09)60144-6.

77. Li C, He J, Chen J, et al. Genome-Wide Gene-Sodium Interaction Analyses on Blood Pressure: The Genetic Epidemiology Network of Salt-Sensitivity Study. *Hypertension*. 2016;68(2):348-355. doi:10.1161/HYPERTENSIONAHA.115.06765.

78. He J, Streiffer RH, Muntner P, Krousel-Wood MA, Whelton PK. Effect of dietary fiber intake on blood pressure: a randomized, double-blind, placebo-controlled trial. *J Hypertens*. 2004;22(1):73-80. doi:10.1097/01.hjh.0000098164.70956.54.

79. He J, Ogden LG, Vupputuri S, Bazzano LA, Loria C, Whelton PK. Dietary sodium intake and subsequent risk of cardiovascular disease in overweight adults. *JAMA*. 1999;282(21):2027-2034. doi:10.1001/jama.282.21.2027.

80. He J, Wofford MR, Reynolds K, et al. Effect of dietary protein supplementation on blood pressure a randomized, controlled trial. *Circulation*. 2011;124(5):589-595. doi:10.1161/CIRCULATIONAHA.110.009159.

81. Thompson AM, Zhang Y, Tong W, et al. Association of inflammation and endothelial dysfunction with metabolic syndrome, prediabetes and diabetes in adults from Inner Mongolia, China. *BMC Endocr Disord*. 2011;11(1):16. doi:10.1186/1472-6823-11-16.

82. Irazola V, Rubinstein A, Bazzano L, et al. Prevalence, awareness, treatment and control of diabetes and impaired fasting glucose in the Southern Cone of Latin America. *PLoS One*. 2017;12(9). doi:10.1371/journal.pone.0183953.

83. Bazzano LA, Li TY, Joshipura KJ, Hu FB. Intake of fruit, vegetables, and fruit juices and risk of diabetes in women. *Diabetes Care*. 2008;31(7):1311-1317. doi:10.2337/dc08-0080.

84. Bazzano LA, Serdula M, Liu S. Prevention of Type 2 Diabetes by Diet and Lifestyle Modification. *J Am Coll Nutr*. 2005;24(5):310-319. doi:10.1080/07315724.2005.10719479.

85. Pollock BD, Chen W, Harville EW, et al. Differential sex effects of systolic blood pressure and low-density lipoprotein cholesterol on type 2 diabetes: Life course data from the Bogalusa Heart Study. *Journal of Diabetes*. 2017.

86. Zhang T, Li Y, Zhang H, et al. Insulin-sensitive adiposity is associated with a relatively lower risk of diabetes than insulin-resistant adiposity: the Bogalusa Heart Study. *Endocrine*. 2016;54(1):93-100. doi:10.1007/s12020-016-0948-z.

87. Pollock BD, Hu T, Chen W, et al. Utility of existing diabetes risk prediction tools for young black and white adults: Evidence from the Bogalusa Heart Study. *J Diabetes Complications*. 2016;31(1):86-93. doi:10.1016/j.jdiacomp.2016.07.025.

88. Hu T, Yao L, Reynolds K, et al. The effects of a low-carbohydrate diet vs. a low-fat diet on novel cardiovascular risk factors: A randomized controlled trial. *Nutrients*. 2015;7(9):7978-7994. doi:10.3390/nu7095377.

89. Hu T, Yao L, Reynolds K, et al. The effects of a low-carbohydrate diet on appetite: A randomized controlled trial. *Nutr Metab Cardiovasc Dis*. 2016;26(6):476-488. doi:10.1016/j.numecd.2015.11.011.

90. Rebholz CM, Reynolds K, Wofford MR, et al. Effect of soybean protein on novel cardiovascular disease risk factors: A randomized controlled trial. *Eur J Clin Nutr*. 2013;67(1):58-63. doi:10.1038/ejcn.2012.186.
91. Wofford MR, Rebholz CM, Reynolds K, et al. Effect of soy and milk protein supplementation on serum lipid levels: A randomized controlled trial. *Eur J Clin Nutr*. 2012;66(4):419-425. doi:10.1038/ejcn.2011.168.
92. Chen J, He J, Wildman RP, Reynolds K, Streiffer RH, Whelton PK. A randomized controlled trial of dietary fiber intake on serum lipids. *Eur J Clin Nutr*. 2006;60(1):62-68. doi:10.1038/sj.ejcn.1602268.
93. Overweight and Obesity Expert Panel. *Managing Overweight and Obesity in Adults: Systematic Evidence Review From the Obesity Expert Panel, 2013*.; 2013.
94. The Data Center. *Who Lives in New Orleans and Metro Parishes Now?*; 2017. https://www.datacenterresearch.org/data-resources/who-lives-in-new-orleans-now/.
95. Wetzels JFM, Kiemeney LALM, Swinkels DW, Willems HL, Heijer M Den. Age- and gender-specific reference values of estimated GFR in Caucasians: The Nijmegen Biomedical Study. *Kidney Int*. 2007;72(5):632-637. doi:10.1038/sj.ki.5002374.
96. Radin MS. Pitfalls in hemoglobin A1c measurement: When results may be misleading. *J Gen Intern Med*. 2014;29(2):388-394. doi:10.1007/s11606-013-2595-x.
97. Kim PS, Woods C, Georgoff P, et al. A1C underestimates glycemia in HIV infection. *Diabetes Care*. 2009;32(9):1591-1593. doi:10.2337/dc09-0177.
98. Qaseem A, Wilt TJ, Kansagara D, Horwitch C, Barry MJ, Forciea MA. Hemoglobin A1c Targets for Glycemic Control With Pharmacologic Therapy for Nonpregnant Adults With Type 2 Diabetes Mellitus: A Guidance Statement Update From the American College of Physicians. *Ann Intern Med*. 2018;168(8):569-576. doi:10.7326/M17-0939.
99. Sumithran P, Prendergast LA, Delbridge E, et al. Ketosis and appetite-mediating nutrients and hormones after weight loss. *Eur J Clin Nutr*. 2013;67(7):759-764. doi:10.1038/ejcn.2013.90.
100. Gardner CD, Trepanowski JF, Gobbo LCD, et al. Effect of low-fat VS low-carbohydrate diet on 12-month weight loss in overweight adults and the association with genotype pattern or insulin secretion the DIETFITS randomized clinical trial. *JAMA - J Am Med Assoc*. 2018;319(7):667-679. doi:10.1001/jama.2018.0245.
101. Brenner BM, Meyer TW, Hostetter TH. Dietary protein intake and the progressive nature of kidney disease: the role of hemodynamically mediated glomerular injury in the pathogenesis of progressive glomerular sclerosis in aging, renal ablation, and intrinsic renal disease. *N Engl J Med*. 1982;307(11):652-659. doi:10.1056/NEJM198209093071104.
102. Sato J, Kanazawa A, Makita S, et al. A randomized controlled trial of 130 g/day low-carbohydrate diet in type 2 diabetes with poor glycemic control. *Clin Nutr*. 2017;36(4):992-1000. doi:10.1016/j.clnu.2016.07.003.
103. Yancy WS, Foy M, Chalecki AM, et al. A low-carbohydrate, ketogenic diet to treat type 2 diabetes. *Nutr Metab (Lond)*. 2005;2(1):34. doi:10.1186/1743-7075-2-34.
104. Boden G, Sargrad K, Homko C, Mozzoli M, Stein TP. Effect of a low-carbohydrate diet on appetite, blood glucose levels, and insulin resistance in obese patients with type 2 diabetes. *Ann Intern Med*. 2005;142(6). doi:10.1016/S0084-3741(08)70336-6.
105. Stern L, Iqbal N, Seshadri P. The Effects of Low-Carbohydrate versus Conventional Weight Loss Diets in Severely Obese Adults : One-Year Follow-up of a randomized control trial. *Ann Intern …*. 2004;140(May 2001):769-777.

doi:10.1016/j.accreview.2004.07.103.

106. Westman EC, Yancy WS, Edman JS, Tomlin KF, Perkins CE. Effect of 6-month adherence to a very low carbohydrate diet program. *Am J Med*. 2002;113(1):30-36. doi:10.1016/S0002-9343(02)01129-4.

107. Moosheer SM, Waldschütz W, Itariu BK, Brath H, Stulnig TM. A protein-enriched low glycemic index diet with omega-3 polyunsaturated fatty acid supplementation exerts beneficial effects on metabolic control in type 2 diabetes. *Prim Care Diabetes*. 2014;8(4):308-314. doi:10.1016/j.pcd.2014.02.004.

108. Aagaard K, Petrosino J, Keitel W, et al. The Human Microbiome Project strategy for comprehensive sampling of the human microbiome and why it matters. *FASEB J*. 2013;27(3):1012-1022. doi:10.1096/fj.12-220806.

109. McInnes P, Cutting M. Manual of Procedures for Human Microbiome Project. http://www.hmpdacc.org/doc/HMP_MOP_Version12_0_072910.pdf.

110. Marrero DG, Palmer KNB, Phillips EO, Miller-Kovach K, Foster GD, Saha CK. Comparison of commercial and self-initiated weight loss programs in people with prediabetes: A randomized control trial. *Am J Public Health*. 2016;106(5):949-956. doi:10.2105/AJPH.2015.303035.

111. Barengolts E, Manickam B, Eisenberg Y, Akbar A, Kukreja S, Ciubotaru I. EFFECT OF HIGH-DOSE VITAMIN D REPLETION ON GLYCEMIC CONTROL IN AFRICAN-AMERICAN MALES WITH PREDIABETES AND HYPOVITAMINOSIS D. *Endocr Pract*. 2015. doi:10.4158/EP14548.OR.

112. Block G, Azar KMJ, Romanelli RJ, et al. Diabetes prevention and weight loss with a fully automated behavioral intervention by email, web, and mobile phone: A randomized controlled trial among persons with prediabetes. *J Med Internet Res*. 2015. doi:10.2196/jmir.4897.

113. Kramer MK, Molenaar DM, Arena VC, et al. Improving employee health: Evaluation of a worksite lifestyle change program to decrease risk factors for diabetes and cardiovascular disease. *J Occup Environ Med*. 2015. doi:10.1097/JOM.0000000000000350.

114. Parker AR, Byham-Gray L, Denmark R, Winkle PJ. The Effect of Medical Nutrition Therapy by a Registered Dietitian Nutritionist in Patients with Prediabetes Participating in a Randomized Controlled Clinical Research Trial. *J Acad Nutr Diet*. 2014;114(11):1739-1748. doi:10.1016/j.jand.2014.07.020.

115. Maruthur NM, Ma Y, Delahanty LM, et al. Early response to preventive strategies in the diabetes prevention program. *J Gen Intern Med*. 2013;28(12):1629-1636. doi:10.1007/s11606-013-2548-4.

116. Leong A, Daya N, Porneala B, et al. Prediction of Type 2 Diabetes by Hemoglobin A1c in Two Community-Based Cohorts. *Diabetes Care*. 2017;41(1):dc170607. doi:10.2337/dc17-0607.

117. Khaw K-T, Wareham N, Bingham S, Luben R, Welch A, Day N. Association of hemoglobin A1c with cardiovascular disease and mortality in adults: the European prospective investigation into cancer in Norfolk. *Ann Intern Med*. 2004;141(6):413-420. doi:10.7326/0003-4819-141-6-200409210-00006.

118. Cole RE, Boyer KM, Spanbauer SM, Sprague D, Bingham M. Effectiveness of Prediabetes Nutrition Shared Medical Appointments. *Diabetes Educ*. 2013;39(3):344-353. doi:10.1177/0145721713484812.

Low-Carbohydrate Dietary Pattern on Glycemic Outcomes Trial

PROTOCOL

PROTOCOL VERSION 1.9

Dated: December 03, 2019

New Orleans, LA

Table of Contents

1. Overview 5
2. Background and Significance 6
2.A. Type 2 Diabetes Mellitus is a major global public health challenge 6
2.B. Prediabetes is a major risk factor for cardiometabolic diseases 6
2.C. Role of lifestyle in preventing diabetes 6
2.D. Low-to-moderate carbohydrate diets, weight loss, and cardiometabolic diseases 7
2.E. Mediterranean-style diet is associated with reduced risk of T2D and improved glycemic control 8
2.F. Evidence for low-carbohydrate diets that emphasize Mediterranean-style dietary components 8
2.G. Evidence for specific food groups and risk of T2D 8
2.H. Gut microbiota, diet and T2D 9
2.I. Continuous Glucose Monitoring 9
2.I. Clinical and public health significance of the proposed study 9
2.J. Innovation 10
3. Preliminary Studies 10
3.A. Summary of aggregate experience of the research team 10
3.B. Low-carbohydrate behavioral dietary intervention trial experience 11
3.C. Other dietary intervention trial experience 11
4. Study Design and Organization 12
4.A. Overview of trial design 12
4.B. Study outcomes 12
4.B.1. Primary outcome 12
4.B.2. Secondary outcomes 13
4.C Study Timeline 13
6. Study Participants 13
6.A. Eligibility criteria 13
7. Recruitment and Randomization 14
7.A. Recruitment 14
7.A.1. Mass mailing, placement of brochures/flyers in community, TV/radio/billboard advertisements 14
7.A.2. Database searches and targeted mailing 15
7.A.3. Referrals and Local Partnerships 15
7.B. Randomization 15

7.C. Masking 15
7.D. Safety Considerations 15
8. Dietary Intervention Program 16
8.A. Low-carbohydrate dietary intervention group 16
8.B. Usual Diet Group 17
8.C. Physical Activity 17
8.D. Dietary Adherence 17
9. Safety of Dietary Intervention 18
10. Data Collection 18
10.A. Study visits 18
10.A.1. Screening/baseline visits 19
10.A.2. Follow-up visits and final visit 20
10.A.3. CGM 6-month visit 20
10.B Study measurements 21
10.B.1. Questionnaires 21
10.B.2. Dietary measurements 21
10.B.3. Assessment and management of adverse effects 21
10.B.4. BP and anthropometric measures 21
10.B.5. Blood samples 21
10.B.6. Urine collection 22
10.B.7. Stool Specimen Collection 22
10.B.8. CGM at 6 months: 22
11. Quality Assurance and Quality Control 23
11.A. Manual of procedures 23
11.B. Training and certification 23
12. Sample Size and Statistical Power 24
13. Data Management and Analysis 25
13.A. Data management 25
13.B. Data analysis plan 25
13.B.1. Exploratory data analysis 25
13.B.2. Primary, secondary, and exploratory outcomes 25
15. Anticipated Challenges 25
16. Protection of Human Subjects 26

16.A. Protection of human subjects from research risks 26
16.A.1. Risks to the subjects 26
16.A.2. Adequacy of protection against risks 28
16.A.3. Potential benefits of the proposed research to human subjects and others... 29
16.A.4. Importance of knowledge to be gained 29
16.A.5. Remuneration 29
16.B. Data safety and monitoring plan 29
Bibliography and References Cited 31

1. Overview

The increasing prevalence of type 2 diabetes mellitus (T2D), a major cause of morbidity and mortality worldwide, is a critical public health concern. In the United States, an estimated 33.9% of adults have prediabetes (impaired fasting glucose, elevated hemoglobin A1c (HbA1c), or impaired glucose tolerance). Individuals with prediabetes are at elevated risk of T2D and cardiovascular disease (CVD), with 5–10% developing clinical T2D each year.

In the short-term, among patients with T2D, low-to-moderate carbohydrate diets (energy percentage below 45) have a greater glucose-lowering effect than do high-carbohydrate diets, with larger glucose-lowering effect the greater the carbohydrate reduction. Additionally, compared with low-fat diets, Mediterranean diets that are high in unsaturated fats (particularly *cis*-monounsaturated fats) reduce risk of T2D and CVD and improve glycemic control among diabetics. However, compared with usual diets, the effect of a behavioral intervention promoting a low-carbohydrate/high-unsaturated fat and protein dietary pattern that emphasizes consumption of non-starchy vegetables, plant-based oils, fish, poultry, and mixed nuts on glucose homeostasis, and other metabolic risk factors among individuals with prediabetes or early-stage untreated T2D is not well understood.

Our *long-term goal* is to develop and implement dietary intervention(s) that reduce risk of T2D. The *overall goal of this randomized controlled trial is to study the effect of a behavioral intervention promoting a low-carbohydrate/high-unsaturated fat and high-protein dietary pattern (initial target <40 g digestible carbohydrates, final target <60 g digestible carbohydrates) compared with a usual diet on metabolic risk factors among individuals with prediabetes or early-stage untreated diabetes* (HbA1c 6.0–6.9%). Specifically, we will test the following hypotheses:

Primary hypothesis: Compared with usual diet, over a 6-month period, a healthy low-carbohydrate/high unsaturated fat and high-protein dietary pattern will lead to *a larger decrease of HbA1c, by at least 0.15%.*

Secondary hypothesis: Over a 6-month period, a healthy low-carbohydrate/high-unsaturated fat and high-protein dietary pattern will lead to *larger improvements in the following major secondary outcomes*: fasting glucose, systolic blood pressure (BP), total-to-HDL-cholesterol ratio, and body weight.

Exploratory outcomes: We will also study the effect of the intervention on the following outcomes: insulin and homeostasis model assessment of insulin resistance (HOMA-IR); diastolic BP; waist circumference; and estimated CVD risk.

To achieve these study aims, we will recruit 112 adults with prediabetes or untreated diabetes (HbA1c 6.0–6.9%) in the Greater New Orleans, LA area. Using a stratified, blocked randomization scheme, participants will be assigned to either a behavioral modification intervention aimed at promoting a healthy low-carbohydrate dietary pattern or to usual diet. The intervention group will have an intensive phase for 3 months, followed by a maintenance phase in months 4 through 6. The *primary outcome will be difference between the active intervention and control groups in the change in HbA1c from baseline to 6 months of follow-up. We expect 85% power to detect a difference of at least 0.15% in change of HbA1c.* Changes in other metabolic risk factors will be studied concurrently. Metabolic risk factors will be assessed at baseline, 3 months, and 6 months of follow-up.

There is much interest regarding dietary approaches to prevent T2D and its complications. The results from this study will help to determine whether, compared with usual diet, a behavioral intervention aimed at promoting a healthy low-carbohydrate dietary pattern improves metabolic risk factors.

2. Background and Significance

2.A. Type 2 Diabetes Mellitus is a major global public health challenge

Diabetes is a strong and independent risk factor for CVD morbidity and mortality. A meta-analysis of 102 prospective studies (698,782 participants) estimated diabetes leads to an approximately 2-fold increased risk in vascular diseases, including coronary heart disease (CHD) and major stroke subtypes, and deaths due to other vascular causes.[1] Among participants with diabetes, CHD risk is about 50% higher among those with fasting blood glucose ≥7 mmol/L compared with those with fasting blood glucose <7 mmol/L.[1] In addition to increasing CVD risk, diabetes can cause diabetic retinopathy,[2] chronic kidney disease,[3] lower limb amputation,[4] and other complications. Most individuals with diabetes have T2D.[5] The US Centers for Disease Control and Prevention (CDC) estimated that 30.2 million adults ≥18 years (12.2%) had diabetes in 2015, with 7.2 million (23.8%) not being aware of or reporting diabetes.[5] Diagnosed diabetes prevalence among US adults has increased substantially over time, from 2.54% in 1980 to 7.40% in 2015.[6] Diabetes prevalence is also high globally, with an estimated 2014 age-standardized prevalence of 9.0% in men and 7.9% in women.[7] Diabetes accounts for an increasing portion of US healthcare expenditures. Total cost of diagnosed diabetes in the US was estimated to be $245 billion in 2012, 41% higher than in 2007.[8] Diabetes is associated with much higher lifetime medical expenses, with higher expenses for those diagnosed at younger ages.[9]

2.B. Prediabetes is a major risk factor for cardiometabolic diseases

Prediabetes, defined by the American Diabetes Association (ADA) as impaired fasting glucose (IFG), impaired glucose tolerance (IGT), or elevated HbA1c, is also referred to as "intermediate hyperglycemia"[10] and "high-risk state of developing diabetes".[11] The ADA defines prediabetes as having: fasting plasma glucose of 100–125 mg/dL (IFG); 2-hour post-load glucose in the 75-g oral glucose tolerance test (OGTT) of 140–199 mg/dL (IGT); or HbA1c 5.7–6.4%.[12] Prediabetes itself is not a disease; for all three tests, "risk is continuous, extending below the lower limit of the range and becoming disproportionately greater at the higher end of the range".[12] The CDC estimated that in 2015, 33.9% of US adults had prediabetes based on fasting glucose or HbA1c.[5] Among those with prediabetes, only 11.9% reported having being told this by a medical professional.[5] Approximately 5–10% of individuals with prediabetes develop diabetes within one year, depending on how prediabetes is defined.[13] In a meta-analysis, annual diabetes incidence was 15–19% among individuals with both IFG and IGT, while it was 4–6% in those with isolated IFG and 6–9% in those with isolated IGT.[13]

A recent meta-analysis (1,611,339 participants from 53 studies) reported that prediabetes as defined by the ADA is associated with higher risk of composite CVD events and CHD.[14] IFG and IGT were also positively associated with increased risk of stroke and all-cause mortality.[14] Prediabetes is linked with increased risk of other conditions typically thought of as diabetes complications, such as neuropathies and nephropathies.[15] Importantly, it is possible for individuals with prediabetes to revert to normoglycemia, as observed in observational studies[13] and lifestyle or drug intervention trials.[16,17] In the Diabetes Prevention Program (DPP), reversion to normoglycemia was associated with lower long-term T2D risk[18] and lower predicted CVD risk.[19]

2.C. Role of lifestyle in preventing diabetes

Much evidence from observational studies and clinical trials supports the critical role of diet and other lifestyle factors in preventing T2D. Globally, lifestyle transitions towards reduced physical activity, increased consumption of refined carbohydrates, coupled with resulting changes in body composition, has led to increases in diabetes prevalence and severity.[20] Body

mass index (BMI), waist circumference, and waist-to-height ratio are predictors of T2D.[21] The DPP, which combined calorie restriction and exercise to promote weight loss, substantially reduced risk of T2D (by 58%) among individuals with elevated FPG and IGT;[22] a benefit that persisted in 10-year follow-up analyses.[16] Other trials of lifestyle modification focused on diet and exercise have also shown benefits in study populations in China,[23] Finland,[24] and India.[25]

2.D. Low-to-moderate carbohydrate diets, weight loss, and cardiometabolic diseases

Low-to-moderate carbohydrate diets (<45% of energy from carbohydrates) are popular dietary approaches for losing weight. Weight loss is a key component of the DPP lifestyle intervention[26] and ADA lifestyle recommendations.[12,27] Meta-analyses from our group[28] and others support that low-to-moderate carbohydrate diets are at least as effective as low-fat diets at promoting weight loss. For instance, a meta-analysis reported a 2-kg (95% CI, 3.1–0.9) larger weight loss in low-carbohydrate diet (<120 g carbohydrates/day) arms compared with low-fat diet (<30% energy from fat) arms.[29] Estimated 10-year CVD risk was lower in low-carbohydrate than low-fat arms.[29] This aligns with research published by our team; in a 12-month trial of participants with obesity, a low-carbohydrate diet (target <40 g carbohydrates/day) was more effective than a low-fat diet (target <30% energy from fat, <7% saturated fat) for weight loss and CVD risk factor reduction.[30]

Low-to-moderate carbohydrate diets may also be beneficial among patients with T2D. Low-carbohydrate diets have come in and out of favor as treatment of diabetes.[31] In a meta-analysis (1376 patients;10 trials), after 3 or 6 months of the intervention, low-to-moderate carbohydrate diets (<45% energy from carbohydrates) led to a 0.34% lower HbA1c (95% CI, 0.06–0.63%) compared with high-carbohydrate diets.[32] Compared with high-carbohydrate diets, there was a greater reduction in HbA1c the greater the reported carbohydrate restriction.[32] There was no benefit of the low-to-moderate carbohydrate diets on HbA1c at 12 months or longer, and no difference between interventions on BMI, body weight, low-density lipoprotein (LDL) cholesterol or quality of life.[32] However, results may have been affected by dietary adherence or glucose medication. There was evidence of diabetes medication reduction in the low-carbohydrate arms in some studies,[33–37] with evidence of medication reduction and improved blood glucose stability persisting for up to 2 years.[38] Hypoglycemia (though not after medication adjustment) was observed in three subjects taking insulin or a sulfonylurea out of twelve subjects in the low-carbohydrate arm of another study.[39] In a subset of participants with T2D (n=46) from a large weight loss trial, compared with those randomized to a low-fat diet and orlistat, those randomized to a low-carbohydrate diet for 48 weeks had improved HbA1c (0.8% lower, 95% CI, -1.5, -0.02%) and a reduction in antiglycemic medications, despite similar weight loss effects of the interventions.[40]

Though low-to-moderate carbohydrate diets have been studied among overweight and obese participants (primarily focused on weight loss), less is understood about the effects of such diets on glycemic outcomes specifically among individuals with prediabetes. A study among participants with IGT observed a reduction at 12 months in weight, HbA1c, and other glycemic outcomes among individuals who participated in a 7-day in-hospital education program, though this was not randomized.[41] A randomized pilot trial of overweight or obese individuals with T2D or prediabetes found that, over 3 months and at 12 months, a very low-carbohydrate diet improved HbA1c, led to higher medication discontinuation, and more weight loss compared with a low-fat diet.[34,37] However, only 4 individuals had prediabetes. Given benefits of low-to-moderate carbohydrate diets for weight loss in general populations and for glycemic control among patients with T2D, further study of these diets are warranted among individuals with prediabetes who are at elevated cardiometabolic disease risk.

2.E. Mediterranean-style diet is associated with reduced risk of T2D and improved glycemic control

Though there is no one "Mediterranean diet", the Mediterranean dietary pattern tends to have the following characteristics: high consumption of vegetables, fruits, breads and cereals, legumes, nuts and seeds; low-to-moderate consumption of dairy products, fish, poultry and eggs; little consumption of red meat and sweets; and red wine in moderation.[42] Olive oil is an important source of fat. In many observational studies, adherence to the Mediterranean-style dietary pattern is associated with reduced risk of CVD[43] and T2D.[44,45] Evidence from randomized trials also supports the benefits of this dietary pattern. In several trials, participants randomized to Mediterranean diets had substantially lower risk of CVD than those randomized to low-fat control diets.[46,47] In the three-arm randomized PREDIMED trial of participants at high risk of CVD, after 4.1 years of follow-up, those randomized to a traditional Mediterranean diet supplemented with either olive oil or nuts had a 30% reduced risk of T2D compared to those in the low-fat control diet group.[48] In all PREDIMED arms, average carbohydrate intake was relatively low (39.7–43.7% at end of trial).[47] Two trials have found a higher rate of remission from metabolic syndrome among individuals randomized to a Mediterranean diet compared with those randomized to control diets (prudent or low-fat) during 2–5 years of follow-up.[49,50] The Mediterranean pattern may also be beneficial among individuals with T2D. Meta-analyses have reported that, among patients with T2D, those randomized to a Mediterranean diet had lower HbA1c levels and CVD risk factors, and more significant weight loss, compared with those randomized to control diets.[51]

2.F. Evidence for low-carbohydrate diets that emphasize Mediterranean-style dietary components

Many low-carbohydrate diets involve increased consumption of food items, such as red meat,[52] that are associated with increased cardiometabolic disease risk. However, low-carbohydrate dietary approaches can focus on substituting carbohydrates with foods common in Mediterranean-style diets, such as olive oil and nuts, which may improve cardiometabolic health. In a 4-year Italian study of individuals with newly diagnosed T2D, those randomized to a low-carbohydrate Mediterranean diet (<50% carbohydrates; consumed ~44%) had more favorable changes in glycemic control, higher rates of T2D remission, and delayed need for antiglycemic drugs compared with participants randomized to a low-fat diet (<30% fat, <10% saturated fat).[53,54] A 12-month Israeli study assessed low-carbohydrate Mediterranean (35% carbohydrates), traditional Mediterranean, and ADA (both 50–55% carbohydrates) diets in 259 overweight participants with T2D.[55] Most CVD risk factors improved in all groups; there was only HDL improvement in the low-carbohydrate Mediterranean diet and this diet was superior to the other diets in improving glycemic control.[55] In a 2-year weight-loss study of moderately obese participants, low-carbohydrate and Mediterranean diets were superior to low-fat diet for weight loss.[56] Participants in the low-carbohydrate arm were counseled to choose vegetarian sources of fat and protein and to avoid trans fat. At 6 years after study commencement, despite weight regain, there were still long-term benefits, particularly for the low-carbohydrate and Mediterranean diets.[57] In whole, evidence supports that low-carbohydrate Mediterranean-style diets are feasible and may have metabolic benefits.

2.G. Evidence for specific food groups and risk of T2D

Prospective cohort studies have found consumption of specific foods and beverages is associated with diabetes risk. Meta-analyses report higher consumption of processed and unprocessed red meat,[58] white rice (particularly in Asian populations),[59] and sugar-sweetened beverages[60,61] are associated with higher T2D risk. Consumption of green leafy vegetables,[62,63] yogurt,[64] whole grains,[65] decaffeinated and total coffee,[66] and light-to-moderate alcohol

consumption[67] are associated with reduced risk. Consumption of fruits, particularly blueberries, grapes, and apples, are associated with reduced risk; fruit juice is associated with higher risk.[68]

2.H. Gut microbiota, diet and T2D

There is increasing evidence for the importance of gut microbiota in risk of type 2 diabetes and other cardiometabolic diseases.[69] Additionally, dietary modifications, including changes in carbohydrate consumption, can lead to substantial changes in gut microbiome over short periods of time.[70,71] Less is known about longer-term changes to the gut microbiota during the adoption of a low-carbohydrate diet and how these changes may influence cardiometabolic risk. We plan to collect stool specimens at baseline, 3 months, and at 6 months, for potential future studies on how this intervention affects the gut microbiome.

2.I. Continuous Glucose Monitoring

As HbA1c is a marker of weighted average glucose over the past 8 to 12 weeks, it does not explicitly capture short-term glycemic excursions or variability. It has been theorized that such fluctuations may have an independent role in the development of diabetes complications.[3] Though more evidence is needed, research has found relationships of higher glycemic variability with coronary artery disease (CAD) and microvascular outcomes in type 2 diabetes (T2D).[4,5] This suggests that improved control of extreme glucose excursions, particularly postprandially, may help to reduce risks of such complications.[5]

In one study of a low-calorie, low-carbohydrate diet compared with a low-calorie, low-fat behavioral dietary intervention among individuals with T2D, there was evidence that some markers of glycemic variability were further reduced in the low-carbohydrate arm compared with the low-fat arm (assessed by 48-hour continuous glucose monitoring) at 6 months, 1 year, and 2 years.[6–8] However, there is not prior work assessing how a behavioral intervention aimed at reducing carbohydrate consumption affects mean glucose, glucose exposure above the set-point of 100 mg/dL, glucose excursions, or glycemic variability as measured by continuous glucose monitoring among individuals with untreated T2D or at high risk of T2D.

Given this background, we will use continuous glucose monitors (CGM) to collect data for up to 14 days on glucose levels at the end of the 6-months study and will compare mean 24-h glucose among those in the intervention group compared with mean 24-h glucose among those in the control group. We will also measure other markers of glycemic exposure, such as glycemic variability (mean amplitude of glycemic excursions (MAGE), standard deviation of glucose, coefficient of variation) and mean 24-hour glucose during wake-time (6:00 AM to 12:00 AM), and during sleep-time (12:00 AM to 6:00 AM).

2.I. Clinical and public health significance of the proposed study

Prediabetes, or intermediate hyperglycemia, is a marker of high risk of developing T2D and other complications. Due to increasing prevalence of T2D and associated morbidity and mortality globally, a better understanding of dietary approaches to delay progression to T2D or to promote reversion to normoglycemia is a high public health priority. This study proposes to assess the effect of a behavioral intervention promoting a low-carbohydrate/high-unsaturated fat dietary pattern (initial target <40 g digestible carbohydrates, final target <60 g digestible carbohydrates) on HbA1c, a marker of glycemic control, and other metabolic risk factors. Building from prior findings from clinical and observational studies, we will develop a dietary intervention that promotes the consumption of non-starchy vegetables, plant-based oils, fish, poultry, and mixed nuts, that are thought to reduced risk of T2D or CVD. Participants assigned

to the intervention will be trained in behavioral techniques that will provide capacity to maintain dietary changes long after study completion. Our outcome measures will assess the effect of this dietary intervention in a “real-life” setting as opposed to dietary modifications achieved in metabolic ward studies or through food prepared in research kitchens. Findings from this study may provide evidence in support of promoting alternative dietary styles among individuals with high T2D and CVD risk and could potentially be used to inform dietary interventions used in programs like DPP.

2.J. Innovation

The status quo as it pertains to dietary approaches for reducing risk of T2D is focused on reducing caloric intake and total fat intake. However, recent evidence suggests that the types of fat, rather than the quantity of fats may be more important, with evidence supporting that a Mediterranean-style diet that does not restrict fat intake reduces risk of developing both T2D[48] and CVD[47]. Moreover, in the short-term, low-carbohydrate diets, which are popular weight loss diets, have a greater glucose-lowering effect than do high-carbohydrate diets among patients with T2D.[32] However, the effect of low-carbohydrate diets that emphasize consumption of non-starchy vegetables, plant-based oils, fish, poultry, and mixed nuts on T2D prevention is unclear, particularly compared with higher-carbohydrate diets more commonly consumed in the US. *The proposed research is innovative, in our opinion, because it represents a substantial departure from the status quo by assessing the combined effect of a low-carbohydrate, high unsaturated fat and protein diet on cardiometabolic risk factors among adults with prediabetes or early-stage untreated diabetes.* We expect the results from this study have the potential to lead to new horizons for developing and implementing dietary approaches (other than the most commonly used reduced fat diet) that will substantially reduce risk of cardiometabolic disease among adults with T2D or at high risk of T2D. The proposed intervention dietary approach promotes consumption of dietary components thought to reduce risk of cardiometabolic disease and the behavior change intervention has expected applicability in clinical practice.

3. Preliminary Studies

3.A. Summary of aggregate experience of the research team

Dr. Kirsten Dorans is trained in cardiovascular disease epidemiology, with strong training in epidemiologic methods and biostatistics. Her publication record includes studies of behavioral risk factors and risk of chronic disease. She has published on the relationships between dietary intake and chronic heart failure[72,73] and on physical activity and incident multiple sclerosis[74]. She served as lead author, conducting data management, analysis, drafting, and revision of two of these manuscripts. In graduate school, she focused on studying links between outdoor air pollution and chronic diseases and has published several papers in this field. The COBRE program will provide an excellent opportunity for the JFI to transition from air pollution research to clinical and translational research focused on nutrition and diabetes. Building off of her training in observational epidemiology, this program will involve training and practical experience that will allow her to broaden her expertise and to develop practical skills for carrying out clinical research.

The mentorship team has much experience and prior successful collaborations in key areas related to this study. Dr. Jiang He is an internationally renowned expert in cardiometabolic disease clinical and epidemiological research. He has served as PI for many epidemiological studies and clinical trials, including clinical and observational studies focused on dietary consumption and chronic diseases[75–80] and has conducted extensive global research on diabetes.[81,82] Dr. Bazzano has collaborated extensively with Dr. He for more than 15 years in research related to diet and chronic disease. She is a clinical investigator and has expertise in

nutritional epidemiology and clinical research. Dr. Bazzano was the PI of a highly successful clinical trial comparing the effects of low-carbohydrate and low-fat diets on weight and cardiovascular risk factors among individuals with obesity, which was a subproject within the Tulane COBRE in Hypertension and Renal Biology.[30] Dr. He served as Dr. Bazzano's mentor for this clinical trial. They have collaborated on much other work related to dietary and chronic disease, including a meta-analysis on low-carbohydrate diets.[28] Dr. Bazzano has also published many articles on diet and risk of diabetes[83,84] and on diabetes in the Bogalusa Heart Study.[85–87]

3.B. Low-carbohydrate behavioral dietary intervention trial experience

In the study on macronutrient composition of diet and risk factors for cardiovascular disease (MACRO) study, 148 individuals without clinical cardiovascular disease or diabetes and with obesity were randomly assigned to a low-carbohydrate or a low-fat behavioral dietary intervention for 12 months. In the low-carbohydrate arm, daily dietary intake of carbohydrates decreased from an average of 242 g at baseline to 97 g, 93 g, and 127 g at 3, 6, and 12 months, respectively. At 12 months, participants in the low-carbohydrate intervention group had greater decreases in weight (-3.5 kg; 95% CI, -5.6 to -1.4 kg) and fat mass (-1.5%; 95% CI, -2.6% to -0.4%) and greater improvement in several cardiovascular risk factors.[30] This suggests that restricting carbohydrates may be an option for weight loss and reduction of cardiovascular risk factors. Further analyses found that the low-carbohydrate dietary intervention led to similar or greater improvement in inflammation, adipocyte dysfunction, and endothelial dysfunction compared with the low-fat dietary intervention[88] and that the low-fat diet reduced peptide YY more than the low-carbohydrate diet, suggesting satiety may be better preserved on low-carbohydrate diets.[89] No serious adverse events were reported in this study and the number of participants with symptoms were similar between the two diets, with the exception of more participants having headaches on the low-fat diet at 3 months (18 vs 6 participants, $p = 0.03$).[30] Dr. Bazzano served as PI for this study and Dr. He served as her mentor. Dr. Bazzano mentored one of her PhD students in conducting analyses and writing and editing manuscripts related to this project.

3.C. Other dietary intervention trial experience

Dr. He has also led or been involved with many dietary intervention trials, including both behavioral change and feeding study interventions. In the Protein and Blood Pressure (ProBP) study, 352 adults with prehypertension or stage 1 hypertension in New Orleans, LA and Jackson, MS were assigned to take, in a random order, 40 g/d soy protein, milk protein, or carbohydrate supplementation each for 8 weeks. Compared with the high-glycemic index refined carbohydrate, both soy protein and milk protein supplementation were associated with a -2.0 mm Hg (95% CI -3.2 to -0.7 mm Hg) and -2.3 mm Hg (-3.7 to -1.0 mm Hg) net change in systolic blood pressure.[80] These findings suggest that partial replacement of carbohydrate with soy or milk protein might be an important part of nutrition intervention strategies to prevent and treat hypertension. Additionally, soy protein supplementation led to reduction in plasma E-selectin compared with carbohydrate and milk protein and in plasma leptin compared with carbohydrate.[90] Soy protein also led to improvement in lipid profile compared with carbohydrate and milk protein.[91] Dr. He was PI for this project.

In the Fiber Intervention on Blood Pressure (FIBRE) study, 110 participants ages 30–65 years with untreated pre-hypertension or stage-1 hypertension and serum cholesterol level < 240 mg/dl were randomly assigned to 8-g/day of water soluble fiber from oat bran or a control intervention (double-blind RCT). Over the 12-week study period, net changes in systolic and diastolic blood pressure were -1.8 mm Hg (-4.3 to 0.8) and -1.2 mm Hg (-3.0 to 0.5 mm Hg), respectively.[78] Net changes in total, HDL-, and LDL-cholesterol were -2.40 mg/dL (-10.60 to

5.81 mg/d:), -1.66 mg/dL (-4.55 to 1.22 mg/dL), and -1.33 mg/dl (-8.33 to 5.68 mg/dL), respectively.[92] Dr He was PI for this study.

Dr. He is currently serving as a mentor for Dr. Katherine Mills' COBRE project (Effect of Dietary Sodium Reduction in Kidney Disease Patients with Albuminuria; SUPER). In this project, participants with chronic kidney disease and albuminuria are being randomized to a behavioral modification program designed to reduce dietary sodium intake to ≤2,300 mg/day or to usual care and test the effect of this intervention on change in albumin-to-creatinine ratio from baseline to 24-weeks.

4. Study Design and Organization

4.A. Overview of trial design

We propose to conduct a *randomized controlled trial* aimed at testing the effect of a behavioral intervention that promotes *reduced carbohydrate intake and increased consumption of heart-healthy unsaturated fats and protein* in patients with HbA1c 6.0–6.9% (*Figure 1*). We plan to recruit 112 individuals with elevated HbA1c and randomly assign them to a moderately intensive intervention on diet pattern,[93] using well-established methods for behavioral modification, or to usual diet. We will compare the difference between the active intervention and control groups for change in HbA1c from baseline to 6 months of follow-up (primary outcome). We will assess four major secondary outcomes: fasting plasma glucose, systolic BP, total-to-HDL cholesterol ratio, and body weight. In addition, we will look at several other exploratory outcomes: insulin and HOMA-IR, diastolic BP, waist circumference, and estimated CVD risk, as estimated by the 2013 ACC/AHA risk equation. The main trial components are summarized in *Table 1*.

Table 1. Study Overview

Study Design	Randomized controlled trial
Study Participants	112 participants with elevated HbA1c (6.0–6.9%)
Intervention Group (n=56)	Behavioral modification to reduce carbohydrate consumption and promote consumption of foods such as non-starchy vegetables, plant-based unsaturated oils, nuts and seeds, avocados, eggs, seafood, poultry, lean meat and tofu. We will recommend moderate consumption of cheese, unsweetened Greek yogurt, and low-carb milk. If consuming higher-carbohydrate items, recommend legumes, fruits, (limited) whole grains. Target <40 g net carbohydrates per day for first 3 months and will have a goal of <40 net g carbohydrates per day; <60 g net carbohydrates per day for months 4 onwards.
Usual Diet Group (n=56)	No dietary intervention. To encourage continued participation in the trial, we will offer optional monthly educational sessions on unrelated topics, such as retirement planning or health insurance
Outcome Measures	Primary: HbA1c; Secondary: fasting plasma glucose, systolic BP, total-to-HDL-cholesterol ratio, and body weight

4.B. Study outcomes

4.B.1. Primary outcome: The *primary outcome will be the difference between the intervention group and the usual diet group for change in HbA1c* from baseline to 6 months. Changes in HbA1c will be calculated from blood samples taken at baseline and 6 months.

4.B.2. Secondary outcomes: Secondary outcomes will include changes from baseline to 6 months of *fasting plasma glucose, systolic BP, total-to-HDL cholesterol ratio, and body weight.,*
4.B.3. Other outcomes: We will also study the effect of the intervention on the following outcomes: insulin and homeostasis model assessment of insulin resistance (HOMA-IR); diastolic BP; waist circumference; total-to-HDL-cholesterol ratio; and estimated CVD risk.

4.C Study Timeline

Table 2 shows the timeline, which will be followed in the implementation of study-related activities, such as publications and submission of an R01 grant application.

Table 2. Study Timeline

	Year 1		Year 2		Year 3	
Develop MOP, intervention program, study forms, and pilot testing	X	X				
Certification and training of staff	X					
Recruitment		X	X			
Intervention and data collection, measurement, entry, cleaning		X	X	X	X	
Data quality control and analysis						X
Publications	X	X	X	X	X	X
R01 grant submissions, resubmissions				X	X	X

The first 6 months will focus on development of manual of operations (MOP), intervention program, study forms, study protocol, Institutional Review Board (IRB) approval, and pilot testing of the intervention program. It will also involve staff training and certification. During the last half of year 1, recruitment, intervention, and data collection will commence. During the last 3 months of year 3, data quality control and analysis will be carried out. Throughout the study period, the JFI will publish peer-reviewed manuscripts from ongoing Tulane University research projects.

6. Study Participants

6.A. Eligibility criteria

Inclusion criteria

- Men or women ages 40 to 70 years: aim to recruit 50% women
- Any race or ethnicity: aim to recruit 40% ethnic minorities (i.e., African American and Hispanic), close to population in the greater New Orleans area[94]
- HbA1c 6.0–6.9%
- Willing and able to provide informed consent

Exclusion criteria

- Diagnosed type 1 diabetes mellitus based on patient self-report
- Use of agents affecting glycemic control (medications for diabetes, oral glucocorticoids) within the past three months prior to enrollment based on patient self-report
- Medical condition in which low-carbohydrate diet may not be advised
    - Estimated glomerular filtration rate (eGFR) ≤45 mL/min/1.73 m^2, which is close to the 5th percentile of eGFR among non-diseased individuals of 70 years of age[95]
    - Self-report of liver disease due to hepatitis or alcohol; osteoporosis; untreated thyroid disease; gout; or cancer (other than non-melanoma skin cancer) requiring treatment in the past year, unless prognosis is excellent
- Factors that may affect HbA1c:
    - Hemoglobin <11 mg/dL (cutpoint for moderate-to-severe anemia, which could lead to falsely elevated or lowered HbA1c)[96]

    - Recent blood donation or blood transfusion (self-report, past 4 months)
    - Human Immunodeficiency Virus (self-report)[97]
- Allergies to nuts
- For women, current pregnancy, breastfeeding, or plans to become pregnant during the study
- Consumption of ≥21 alcoholic drinks per week or consumption of ≥6 drinks per occasion
- For the CGM collection at the end of the study period only: known allergy to adhesives or other products involved with CGM use (e.g., skin disinfectants), current pregnancy, currently on hemodialysis or peritoneal dialysis, or people with other implanted medical devices (e.g., a pacemaker)
- Current or planned residence making it difficult to meet trial requirements (due to distance from study site and/or challenges regularly traveling to site)
- Current participation in another lifestyle intervention trial or a pharmaceutical trial
- Participation of another household member in the study; employees or persons living with employees of the study
- Other concerns regarding ability to meet trial requirements, at the discretion of the principal investigator or study coordinator

7. Recruitment and Randomization

7.A. Recruitment

We will recruit study participants from the greater New Orleans area. The initial focus of our recruitment will be mass mailing of individuals in the greater New Orleans area and recruitment through placing flyers and brochures in the community. If community-based recruitment is not successful or cost-effective, we will recruit out-patients from the Ochsner Health System and Tulane Medical Center (TMC), from which our group has prior experience recruiting patients. For this recruitment, we will focus on electronic searches of medical records and laboratory databases. We will also carry out targeted solicitation of referrals from physicians who care for patients likely to meet our inclusion and exclusion criteria.

In addition to this recruitment plan, we have several contingency plans: (1) extending recruitment to other medical centers in the New Orleans area, including the Veterans Affairs Medical Center (VAMC), East Jefferson General Hospital, and West Jefferson Medical Center, with whom Tulane has well-established relationships; (2) TV/radio advertisements; (3) arrange a news story about the study in local TV newscasts, radio talk programs, and/or print media; and (4) conduct outreach at local community events and churches. We have ample experience with each of these approaches.

Based on U.S. Census Bureau 2016 population estimates, the metro New Orleans area population was approximately 35% African American or black and 9% Hispanic.[94] Given our prior experience and population distribution in this region, we plan to recruit a distribution of study participants with 40% ethnic minorities.

7.A.1. Mass mailing, placement of brochures/flyers in community, TV/radio/billboard advertisements: Community recruitment approaches include: mass mailing individuals who live in the New Orleans area; brochures/flyers in the community; emailing the brochure; TV, radio, poster, or billboard advertisements in the New Orleans area. Interested individuals will be pre-screened; those at elevated risk for prediabetes or T2D, i.e. individuals who are obese or overweight and/or have a sufficiently high score from the American Diabetes Association’s Diabetes Risk Assessment Questionnaire will be invited into the clinic for a screening visit.

7.A.2. Database searches and targeted mailing: We will request IRB approval for a waiver for HIPAA authorization to conduct database searching and hospital chart reviews for identification of potentially eligible study participants. After identifying a potentially eligible patient, we will send this individual an invitation letter signed by the investigators and/or the individual's personal physician and brochure via mail or email. Individuals who express interest in the study will have a pre-screening telephone interview for confirmation of potential eligibility. Those still interested and potentially eligible for enrollment will be scheduled for a visit at the COBRE study clinic.

7.A.3. Referrals and Local Partnerships: In addition to local physician referrals, we will explore holding outreach community events in the New Orleans area. Local community partners may post information about the study on their social media platforms. We may also identify potentially interested volunteers from a Tulane volunteer recruitment registry. We will also consider recruiting potentially eligible individuals who were screened or participated in other clinical studies in our clinic and have expressed interest in being contacted about future research projects. Potential participants referred by their physician or community partners will have a pre-screening telephone interview to confirm potential eligibility. Those who are still interested and potentially eligible for the study will be scheduled for a screening visit.

7.B. Randomization

Once eligibility has been confirmed, the study coordinator will ensure that all key variables have been entered into the trial-specific database. Additionally, the study coordinator will ensure any outliers have been recognized and reconciled, and will ensure receipt of informed consent prior to enrolling the volunteer in the study as a trial participant. Eligible participants will be *randomly assigned to the behavioral intervention or the usual diet group in a 1:1 allocation ratio*. Randomization will be stratified by sex, *using a random block group size (4, 6) to maintain balance among groups over time*. A statistician from the Methodology/Biostatistics Unit within the COBRE Clinical Research Core, who is not directly involved in the study and is not located at the clinic, will conduct the randomization. After ascertaining eligibility, clinical personnel will telephone the Methodology/Biostatistics Unit to obtain a randomization assignment. All randomization assignments will be created by a computer program and only accessible to the Staff at the Methodology/Biostatistics Unit. Once randomization assignment has been made, trial participants will be considered to be officially randomized into the study and every effort will be made to obtain complete follow-up information.

7.C. Masking

Clinical and laboratory staff members who assess outcomes will be blinded to the intervention assignment of participants. As this is a dietary intervention trial, study participants, and study staff members who conduct the intervention cannot be blinded to the intervention assignment. Participants will be instructed to avoid sharing knowledge of their treatment assignment with data collection staff members. We will have intervention visits and data collection study visits on different days and in separate locations. Data collectors will also use verbal cues and visual props to remind participants not to disclose treatment assignment. Investigators will be blinded to intervention assignment.

7.D. Safety Considerations

Participants with HbA1c >7.9% will be referred to a clinician for follow-up[98], though we will continue to collect data on these participants.

8. Dietary Intervention Program

8.A. Low-carbohydrate dietary intervention group

Participants randomized to the low-carbohydrate intervention group will receive counseling aimed at reducing carbohydrate consumption. Phase I (*Go Low*) will occur for the first 3 months, with a goal of <40 g digestible carbohydrates per day. Key recommended foods will include: non-starchy vegetables, fish, poultry, lean meat, eggs, olive oil and other plant-based unsaturated oils, unsweetened/unsalted nuts and seeds, nut butters, and avocados. We will recommend moderate consumption of cheese and unsweetened Greek yogurt and low-carbohydrate milk. During Phase I, we will recommend limiting or avoiding other dairy, fruits, legumes, beans, and grains. Phase II (*Keep it Low*) will run from months 4 through 6. The net carbohydrate goal for this phase will be <40 g to <60 g, with the objective to choose a target that is as low as possible (e.g., as close to <40 g as possible). At 3 months, we expect different participants will have different levels of intake, with some consuming <40 g of net carbohydrates and others consuming levels higher than 60 g net carbohydrates per day. We have designed the intervention to accommodate various scenarios, with the goal of achieving as low of an intake as possible. For participants who consume >60 g per day, we will recommend they aim to either reduce or maintain carbohydrate intake.

At a brief check-in at 3-months, if participants are satisfied with their current intake, we will instruct them to stay at this intake level (though will recommend they try to reduce intake, if possible, if they are eating >60 net grams of carbohydrates or have not yet reached the study goal of <40 net grams of carbohydrates). If they prefer to make modifications, we will provide guidance on how to modify carbohydrate intake (either decrease or increase). If they choose to add back carbohydrates, we will recommend that participants add 5 grams of carbohydrates for two weeks at a time (e.g., from 40 to 45 g for 2 weeks), for a maximum total of <60 g net carbohydrates. If they choose to further reduce carbohydrate intake, we will provide guidance on this as well. As they modify carbohydrate intake, we will have them monitor their satisfaction with their diet and energy levels. If adding back carbohydrates, we will recommend participants add back in foods like fruit (preferably berries), legumes, beans, and dairy, while still aiming to keep total carbohydrate intake as low as possible (e.g., as close to <40 net grams as able).

Materials: We will develop a handbook that will be given to participants. This will contain diet guidelines, recipes, meal planners, shopping lists, and guides for counting carbohydrates and reading nutrition labels.

Meals: Participants will select their own menus and prepare or buy their meals according to the guidelines. We will provide participants with supplemental low-carbohydrate foods, such as nuts, olive oil, and other products, to help facilitate their compliance.

Counseling: Sessions and materials will focus on instructing participants to reduce digestible carbohydrate consumption by increasing consumption of protein and fat, with a particular emphasis on increased consumption of heart-healthy unsaturated fats. After randomization, participants will begin the 3-month *Go Low* phase consisting of individual counseling sessions each week for the first four weeks, followed by 4 group sessions held every other week with phone follow-ups occurring in between group sessions. During the *Keep it Low* phase, months 4 through 6, participants will attend 3 monthly group sessions and have 3 telephone follow-ups. There will be a total of 11 in-person sessions and 7 telephone follow-ups over the full study period. Sessions will be approximately one hour and phone calls will be no longer than thirty minutes. These sessions will consist of diet instruction and supportive counseling, addressing results from 24-hour dietary recalls, teaching self-monitoring and individual diet goal changes, and provide suggestions for replacing usual foods containing carbohydrates. Study materials will be modified from materials used in the successful MACRO study. The JFI will work closely

with Drs. He and Bazzano to develop the intervention based on the MACRO study. *Carbohydrate goal*: In the MACRO study, carried out among individuals from the same study region, a behavioral intervention aimed at reducing digestible carbohydrate intake to <40 g/day led to reductions in weight and improvements in cardiovascular risk factors among individuals with obesity.[30] Additionally, restriction of carbohydrate intake to <50g/day is sufficient to induce ketosis.[99] Our rationale for having a multi-phased intervention is to allow participants to see how they feel on the more restrictive diet, and see how they respond to small increases in carbohydrate consumption. A similar technique was applied in the DIETFITS published study.[100] The higher target of 60 net grams may be more sustainable long-term and also allows for consumption of healthy higher-carbohydrate foods, such as berries, legumes, beans, and limited whole grains.

8.B. Usual Diet Group

We will provide participants randomized to the usual diet group written information with standard dietary advice. Participants randomized to the usual diet group will not receive ongoing dietary recommendations. Moreover, they will continue to receive clinical care recommendations from their physician(s). To encourage continued participation in the study and minimize the difference in participant contact with study staff, participants will be offered optional monthly educational sessions on topics unrelated to the study intervention, such as retirement planning or health insurance options.

In another effort to encourage continued participation, patients randomized to the usual diet group will be offered a copy of all available intervention materials, as well as an optional one-hour counseling session with the interventionist following their final study visit.

8.C. Physical Activity

At baseline, both groups will receive written information in study materials with standard physical activity recommendations.

8.D. Dietary Adherence

<u>Strategies for measuring compliance</u>: For the low-carbohydrate diet, adherence will be assessed through measurement of urinary ketones at 3-month visit and 6-month TV. Study staff will make all reasonable effort to maintain adherence of participants to the visit schedule and diet intervention. Walnut consumption will be tracked throughout the study in the low-carbohydrate diet group.

<u>Strategies for improving compliance</u>:

- At the screening visit, we will ask participants who does most of the cooking in their households and how often they eat outside of the home. Results from the screening visit will help to select a group of cooperative study participants.
- Visits will be scheduled at the participants' convenience and to minimize waiting time and trips to the clinic; reminder calls, texts or emails (chosen based on participant preference) will also be made prior to clinic visits.
- We will hire enthusiastic staff who have good interpersonal skills.
- Participants will receive $40 gift cards for use at food stores or retailers when they complete each study visit, for a maximum of $120 over the study period.
- Participants will receive $25 gift cards when they return the stool specimens, for a maximum of $75 over the study period.
- Participants will receive a $25 gift card when they return the continuous glucose monitor, for a maximum of $25 over the study period.
- Participants will be offered small gifts (such as pens or magnets with study logo) to encourage compliance and adherence to the study protocol.

- Participants will be provided with recipes, shopping lists, and food samples for the intervention diet.

9. Safety of Dietary Intervention

On the low-carbohydrate diet, participants will be encouraged to primarily focus on replacing carbohydrates (particularly highly refined carbohydrates) with foods such as non-starchy vegetables, olive oil and other plant-based unsaturated oils, nuts and seeds, avocados, eggs, seafood, tofu, poultry, and lean meat. Consumption of more unsaturated fats, protein, and fiber will be recommended. Participants will not be encouraged to increase consumption of saturated fats and will be advised to avoid consumption of trans fats.

In animal studies, a high-protein diet has decreased renal function[101] In the MACRO study, on average, participants tended to increase consumption of fats, rather than protein.[30] The safety and tolerability of low-carbohydrate diets have previously been assessed in many studies, including MACRO.[30] A number of studies, including some in individuals with T2D, have found no clinically significant increases in estimated glomerular filtration rates,[33,38–40] microalbuminuria,[102] albumin excretion rate,[33,38] urinary albumin,[38] serum creatinine,[38,40,102–107] albumin,[40] or uric acid levels.[103–106] A small study of a very low-carbohydrate diet found significant increase in uric acid excretion, but no significant change in 24-hour urine creatinine clearance.[106]

Study staff will carefully monitor participants' BP, and HbA1c more often than what would occur in routine healthcare visits. A Data Safety and Monitoring Board (DSMB) consisting of an independent group of experts in areas such as biostatistics, nutrition medicine, general internal medicine, and endocrinology has been formed. The board will regularly review trial progress, including unblinded interim results, and can recommend that the trial prematurely end if participants are at risk or if it becomes clear that the trial will not be able to yield statistically significant results.

10. Data Collection

10.A. Study visits

After the initial pre-screening interview, in which data on medications and medical conditions will be collected, there will be one screening visit, one randomization visit, a follow-up study visit at 3 months and a termination visit (TV) at 6 months. *Table 3* summarizes study procedures and data collected at each study visit.

Table 3. Study visit schedule and data collection

	Pre-screen	SV	RZ	3 month	6 month TV
Pre-screen eligibility	X				
Informed consent		X			
Compliance form		X			
Eligibility confirmation			X		
Randomization			X		
Demographics		X			
Medical history questionnaire			X	X	X
Medications		X	X	X	X
Lifestyle (alcohol, cigarette smoking)			X	X	X
Physical activity			X	X	X
24-hour diet recall*			X	X	X
3 BP measurements		X	X	X	X
Body weight			X	X	X
Height			X		
Waist circumference			X	X	X
Spot urine test			X	X	X
Blood samples		X	X	X	X
Stool specimen			X	X	X
Side-effects assessment			X	X	X
Nut Consumption					X
CGM**					X

*Two 24-hour dietary recalls will be collected; one should cover a weekend and the other a weekday.
**The CGM will be worn for up to 14 days prior to the 6-month TV and returned at the TV or by mail. Participants will come in for a 6-month CGM visit (prior to the 6-month TV) to have the CGM inserted.

10.A.1. Screening/baseline visits:

Participants will be pre-screened by phone or in-person by study staff to confirm eligibility for the Screening Visit. An approved questionnaire will be used to ensure the patient is eligible to attend the in-clinic screening visit, and the patient will be scheduled thereafter.

At the screening visit (SV), participants will provide informed consent for the screening and main study. Study staff will schedule the SV and randomization (RZ) visit at least 2 days apart. If a participant is eligible based on the screening visit, he or she should be scheduled for the RZ once a sufficient group of eligible individuals has been recruited. Participants should be scheduled for the RZ at least 2 days and no more than two weeks after the screening visit.

At the screening visit, we will collect information on inclusion/exclusion criteria, medication use, and demographics. At this visit, we will also have participants complete a compliance form,

to assess their ability to complete the study and attend all intervention and study visits. We will also measure blood pressure, and take blood samples. Women will be asked if they are postmenopausal or if they had surgical sterilization (such as a tubal ligation). If not, they will be tested for pregnancy through a spot urine test; pregnant women will be excluded from the study. Blood samples will be collected for measurement of HbA1c, and, if not available from medical records in the past 3 months, serum creatinine and hemoglobin. These blood samples will be used for excluding individuals with HbA1c <6.0 or >6.9, eGFR ≤45 mL/min/1.73 m^2, or hemoglobin <11 mg/dL.

Before the start of the intervention, participants will complete two 24-hour dietary recalls. At least one (and potentially both) 24-h recalls will be collected by phone. At the RZ, participants will learn about the randomization process. After requirements for eligibility have been satisfied, participants will formally be enrolled into the study and randomized to either the active intervention or control group. We will collect fasting blood samples for later measurement of glucose, insulin and lipids and other potential future measurements. At this visit, we will measure blood pressure, body weight, height, and waist circumference. We will collect information on medical history, lifestyle risk factors, and physical activity. A spot urine sample will be collected to measure ketones and other markers and participants will be asked to collect a stool specimen for potential use in future microbiome studies.

10.A.2. Follow-up visits and final visit: A follow-up visit will occur at 3 months after randomization. A final visit will occur at 6-months post-randomization. These visits should be scheduled as close as possible to the 3-, or 6-months after randomization. At each visit, study staff will conduct dietary recalls and anthropometric measures, take 3 BP measurements, collect a fasting blood sample to measure HbA1c. At least one (and potentially both) 24-h recalls will be collected by phone. We will also collect samples for later measurement of glucose, insulin, lipid profile, and other potential future measurements. A spot urine sample will be collected to measure ketones and other markers. Participants will be asked to collect a stool specimen for potential use in future microbiome studies. Information about participants' nut consumption over the past 6 months will be collected at the 6-month TV. At study termination, we will mail participants and/or their physicians a summary of trial results and a record of participant BP, anthropometric measures, and laboratory data from the trial period. Throughout the study period, we will provide participants with their blood pressure and weight. We will request that, if possible, a participant who is prescribed a diabetes medication during the course of the study comes into the clinic for an additional separate supplemental visit for a fasting blood sample collection prior to starting the medication. Maintaining follow-up of all randomized participants is of utmost importance to ensure data integrity. In the event that a participant moves during the study we may request that the participant provides a fasting blood sample at a local LabCorp facility (this will be described on the informed consent form).

10.A.3. CGM 6-month visit: Individuals who are willing to wear the CGM prior to their 6-month termination visit will come to the research clinic approximately 2 weeks before their 6-month termination visit for their CGM visit. At this time, they will have a factory-calibrated CGM inserted just under the skin of the upper arm and activated.[27] If the CGM falls off of a participant prior to 14 days of wear, we may request that the participant (if willing) returns to the clinic for the CGM to be re-inserted. The CGM will be returned at the 6-month TV, or by mail.

10.B Study measurements

Measurements obtained for this study will be based on those used in other clinical trials and epidemiologic studies. We are familiar with strengths and limitations of such measurements and the importance of staff training and certification and use of stringent quality control procedures.

10.B.1. Questionnaires: Study staff will administer a *medical history questionnaire* at the RZ, 3-month visit and 6-month TV to assess medical conditions that would necessitate changes in therapy or medical management beyond this study's scope. At the SV, *demographic factors* will be collected. At the SV, RZ, 3-month and 6-month TV, we will also administer *medication tracking questionnaires*. At RZ, 3-month, and 6-month TV, we will collect health behavior and physical activity data.

10.B.2. Dietary measurements: Two 24-hour dietary recalls will be obtained from participants at baseline. At or close to both the 3-month and 6-month TV, two more 24-hour dietary recalls will be collected. The second sets of two recalls will be collected as closely as possible to 3- or 6-month dates after randomization, with a goal of being within 2 weeks of the respective follow-up period. For each time point, one recall should reflect week day consumption, while the other should reflect weekend day consumption and the two recalls should be at least 2 days apart from each other. We will use computer software (Nutrition Data System for Research [NDS-R]) and food models used in previous and ongoing studies. Recalls will be conducted by a study staff member who has been certified in the use of NDS-R. Food composition tables of the NDS-R will be used to calculate dietary nutrient intakes. The baseline dietary assessment will be done to characterize individual dietary intake of carbohydrates and other macronutrients. During the intervention, repeated measurements will be used to monitor short-term changes in mean dietary nutrient intake between the intervention and usual diet groups.

10.B.3. Assessment and management of adverse effects: At RZ, 3-month visit, and 6-month TV, we will administer *symptom questionnaires*, which will consist of a checklist to identify participant complaints. Prior studies of low-carbohydrate dietary interventions suggest that participants may experience a range of adverse effects, including constipation, halitosis, fatigue, headache, thirst, polyuria, and nausea. Severe adverse events will be promptly reported to the DSMB. Management of adverse effects will be based on the protection and safety of the participant. If severe adverse effects are reported by a participant, carbohydrate intake will be examined and altered in order to mitigate the adverse effect. In any extreme cases, participants will be instructed to stop the intervention. As this is a behavioral intervention, adverse effects should be rare.

10.B.4. BP and anthropometric measures: BP will be measured 3 times at the screening visit and at each follow-up visit. To minimize random error, averages of the measurements at the screening and randomization visits will be taken. BP will be measured by a standard automated blood pressure measurement device (OMRON HEM-907 XL Professional Digital Blood Pressure Monitor) and performed by experienced staff, trained and certified in BP measurement. Prior to BP measurements, participants will be asked to rest quietly in a seated position with their feet supported for at least 5 minutes; staff members will use an appropriate cuff size over a bare arm. Trained and certified staff will measure weight using a calibrated balance beam scale at the screening visits, and follow-up visits. Weight and height will be measured at the RZ, and weight will also be measured at the subsequent visits.

10.B.5. Blood samples: For SV, participants will not need to fast. For RZ, 3-month visit, and 6-month TV, participants will be instructed to fast for 12 hours prior to blood sample collection. At SV, blood samples will be collected to measure HbA1c, hemoglobin, and serum creatinine. At RZ, fasting blood samples will be collected and stored as serum/plasma, whole blood aliquots and buffy coats at -80°C for future analyses, including measurement of glucose, insulin and lipids, and potential future metabolomics or genetic analyses. At 3-month visit and 6-month TV,

we will collect fasting blood samples to measure HbA1c and will also store additional serum/plasma, whole blood aliquots and buffy coats at -80°C for future analyses, including measurement of glucose, insulin and lipids, and potential future metabolomics or genetic analyses. Details of the blood sample collection are shown in *Table 4*. In the event that a participant moves, we may request that they collect a fasting blood sample at a local LabCorp and we will measure HbA1c, glucose, insulin, and lipids at the 3 and 6-month visits. HOMA-IR will be calculated from fasting glucose and insulin.

Table 4. Study visit schedule for specimen collection

	SV	RZ	3 month	6 month TV
HbA1c	X		X	X
Serum creatinine	X			
Hemoglobin	X			
Storage of aliquots and buffy coats (for measurement of glucose, insulin, lipids, other potential future measurements)		X	X	X
Urine sample for pregnancy	X			
Urine sample for ketones and other markers		X	X	X
Stool sample		X	X	X
CGM				X

10.B.6. Urine collection: Study staff will collect spot urine specimens at RZ, interim visit, and the final visit to measure ketones.

10.B.7. Stool Specimen Collection: Stool samples will be collected from all eligible and willing trial participants during the RZ, 3-month, and 6-month TV, following the protocol set forth by the Human Microbiome Project.[108,109] Participants will be given a stool collection kit, which includes the specimen collection container, a shipper for home collection, and a form to record the timing and pertinent information related to specimen collection. Participants will be instructed to return the kit to the clinic within two days of collection (in-person or via shipping), at which time stool samples are stored at -80°C until analyses. Kits and instructions for collection will be reviewed by study staff with the participant at the required visits.

10.B.8. CGM at 6 months: Approximately two weeks before their 6-month termination visit, participants willing to wear the CGM will come to the clinic to have their CGM inserted. This CGM has been found to be accurate compared with capillary blood glucose reference values over 14 days of wear.[27] This CGM records interstitial glucose every 15 minutes throughout the day for a period of up to 14 days. The sensor uses a thin, flexible filament inserted just under the skin to measure glucose and is read through near-field communication by touching the reader to the

sensor. For quality control of the CGM data, a subset of participants who wear the CGM will be asked to wear two CGMs at the same time, one on each arm. Prior to insertion, one arm will be designated as the arm used for data collection and the other arm as a duplicate.

11. Quality Assurance and Quality Control

Throughout the study, we will implement rigorous quality assurance (QA) and quality control (QC) measures. We describe some of these approaches here.

11.A. Manual of procedures

We will develop a manual of procedures (MOP) based on the MOP successfully used in the MACRO behavior change clinical trial. The MOP will provide instructions and describe procedures for staff training and certification, participant recruitment, intervention counseling, study form completion and procedures, data entry, safety measures (e.g., methods for emergency participant/primary care physician contact).

11.B. Training and certification

Trial personnel will all be required to participate in a training session before the start of any trial procedures or measurements. Training will include particular attention to core trial elements, such as recruitment, retention, intervention, completeness and quality of data acquisition (e.g., blinding procedures for the data collectors), and data entry. Separately, intervention staff will meet and receive detailed instruction on trial approaches to group and individual behavior change counseling and procedures that ensure data collectors are blinded to treatment assignment. Throughout the trial, periodic re-training/certification sessions will be conducted to ensure all trial-related procedures are being applied in a standard manner. Investigators will also monitor all aspects of study performance to ensure study goal achievement, timeliness and completeness of study visits, completeness and quality of data collection, intervention adherence, and adequacy of the procedures separating data collection from intervention. Study staff and investigators will pay particularly careful attention to the quality of all laboratory measurements, particularly key exposure, outcome, and intervention monitoring variables.

12. Sample Size and Statistical Power

The primary outcome will be the difference in the change of HbA1c between the intervention and usual diet groups from baseline to 6 months of follow-up. In the United States National Health and Nutrition Examination Survey, when sub-setting to the range of HbA1c of 6.0–6.9% among individuals who do not report use of diabetes medication, the cross-sectional standard deviation is 0.24% using data from 2000 through 2016 (n=4117; https://wwwn.cdc.gov/nchs/nhanes/). In studies with 6-month follow-up among individuals with HbA1c 5.7 to 6.4% or other characteristic(s) (e.g, elevated fasting glucose or metabolic syndrome) that put them at elevated risk of type 2 diabetes, the within-person correlation between baseline and follow-up HbA1c was estimated to be between 0.5 and 0.84.[110–113] As all participants in our study will be within a specific range of HbA1c at baseline, we expect this may lead to smaller within-person correlation of HbA1c over time than studies that do not restrict to a narrow range of HbA1c at baseline. Assuming a within-person correlation of 0.5 and an SD of 0.24% for change in HbA1c, we would need to have 90 individuals to have 85% power to detect an approximate difference in change of HbA1c of 0.15% between the intervention and usual diet groups.

In a follow-up analysis of the DPP, a 0.15% decrement in HbA1c at 6 months was associated with a 16% lower risk of incident T2D, adjusting for treatment arm, age, sex, race/ethnicity, household income, and baseline HbA1c.[115] In a pooled analysis from the Atherosclerosis Risk in Communities and Framingham Heart Studies, a 0.15% higher HbA1c was associated with an 16% higher risk of T2D over 20 years, a 30% higher risk over 8 years, and a 13% higher risk after 8 years, after adjusting for age, sex, fasting glucose, triglycerides, HDL, family history of T2D, SBP, and BMI.[116] Additionally, in EPIC-Norfork, a difference of HbA1c of 0.15% was associated with approximately a 5% higher risk of CHD among those free of CHD, stroke, diabetes, and with HbA1c <7.0%.[117]

Table 5. Minimum detectable differences

	SD	Detectable Difference
HbA1c, %	0.24	0.15
Fasting glucose, mg/dL	12.5	8.0
Fasting insulin, mU/mL	7.0	4.5
HOMA-IR	2.0	1.3
SBP (mmHg)	4.2	2.7
DBP (mmHg)	3.0	1.9
Total/HDL ratio	1.2	0.77
Body weight (kg)	6.6	4.2
Waist circumference (cm)	8.0	5.1
Estimated CVD risk (%)*	6.7	4.8

*10-y CVD risk, among estimated 85% or 76 participants free of CVD

In the DPP study, HbA1c was 0.15% lower among those in the lifestyle intervention arm than in the control arm at 6-months follow-up; HbA1c at study commencement was 5.9% and levels increased in the control arm and decreased in the behavioral intervention arm.[22] In a small behavioral intervention study (n=34) of a very low-carbohydrate ketogenic diet compared with a moderate carbohydrate, calorie-restricted, low-fat diet among individuals with type 2 diabetes or prediabetes (HbA1c >6.0%) and elevated body weight (BMI >25), mean HbA1c decreased from 6.6% to 6.1% in the very low-carbohydrate group and from 6.9% to 6.7% in the moderate carbohydrate group over a 12-month period; additionally, the very low-carbohydrate group had greater reductions in glucose-lowering medications.[37]

Detectable differences are listed for HbA1c and secondary outcomes in *Table 5*, based on data from prior studies.[30,53,56,118] These were calculated for unpaired t-tests, power of 85%, two-sided alpha of 0.05, and assuming 80% follow-up of 112 participants, which would yield an estimated 90 participants.

13. Data Management and Analysis

13.A. Data management

Personnel will double-enter all study data. Prior to entering data, the study coordinator will conduct quality control on all study forms, study measures, and laboratory data. Data will be cleaned and logically checked.

13.B. Data analysis plan

The data will be analyzed using *intention to treat*; this involves comparing the primary and secondary outcomes between participants based on randomization assignment.

13.B.1. Exploratory data analysis: We will express group data for variables with a normal distribution as mean ± standard deviation and for variables not normally distributed as median and interquartile ranges (or will log-transform the variables to obtain a normal distribution). We will compare means and percentages of baseline variables across the intervention and usual diet group to assess similarity of these groups at randomization.

13.B.2. Primary, secondary, and exploratory outcomes: We will test the primary study hypothesis that there is a greater reduction in HbA1c from baseline to 6 months in the intervention group than in the usual diet group. To do this, we will use a mixed-effects linear regression model, to test the null hypothesis that there is no difference in change between baseline and 6-month HbA1c for the intervention group compared with the usual diet group (Specific Aim 1). To do this, we will use the following model:

$$Y_{i,j,k}|T = b_0 + b_1G_i + b_2T_{3\text{-months}} + b_3T_{6\text{-months}} + b_4G_iT_{3\text{-months}} + b_5G_iT_{6\text{-months}} + a_{0i,j} + e_{i,j,k}$$

where $Y_{i,j,k}$ is HbA1c for the jth participant (P) nested in the ith intervention group (G) at time T_k (baseline, 3 months, and 6 months). This model includes a random intercept ($a_{0i,j}$) and error term $e_{i,j,k}$. The interaction term ($b_5G_iT_{6\text{-months}}$) allows for an interaction between group and $T_{6\text{-months}}$, which is equal to 0 for baseline and 3-months and 1 for 6-months. Rejection of the null hypothesis (b_5=0) indicates there is a significant difference in change of HbA1c from baseline to 6 months between the intervention and control groups. In this model, participants are assumed to be random effects and intervention group, time, and the interaction terms are fixed effects.

We will use a similar approach to test the difference in the change between baseline and 6-month values of secondary outcomes (Specific Aim 2) and exploratory outcomes. In exploratory analyses, we will also test the difference in the change between baseline and 3-month values of outcomes (HbA1c, fasting glucose, systolic blood pressure, total-to-HDL cholesterol, body weight, insulin, HOMA-IR, diastolic blood pressure, waist circumference, and estimated CVD risk). We will also explore the use of mixed effects models to assess overall net change in HbA1c (by treating time as a linear variable) and other outcomes over the full study period. For each outcome, we will test modeling assumptions and will consider transformation of outcomes, if needed, to meet modeling assumptions. As the mixed-effects model assumes missing at random, we will also perform sensitivity analyses using Markov-chain Monte Carlo techniques to impute missing values of the outcomes, using covariates for this imputation and will compare results from this analysis with our primary findings.

15. Anticipated Challenges

Recruitment: Many clinical trials have challenges recruiting sufficient numbers of volunteers who meet the trial inclusion and exclusion criteria and demonstrate a high level of adherence to trial protocol requirements. Fortunately, the investigators are very experienced with clinical trial recruitment, and have several contingency options that can be used to meet unanticipated shortfall in recruitment.

Intervention: The investigators are aware of and understand challenges related to achieving and maintaining compliance with a behavioral intervention. The investigators have much experience and success in behavioral interventions, with recent success of a behavioral intervention aimed at reducing carbohydrate intake among participants from the New Orleans area. The JFI will collaborate with Drs. He and Bazzano to develop the intervention program based on the MACRO study and will assess potential barriers to implementation and intervention adherence (such as financial concerns and range of dietary patterns of study participants) during the planning and pilot phase of the study.

Adherence: Though adherence and follow-up are also main challenges related to clinical trials, the investigators have an outstanding track record for studies that have recruited participants for both clinical and epidemiology studies in the Greater New Orleans area. To encourage adherence, small gifts (such as pens or magnets with study logo) will be offered to all study participants.

16. Protection of Human Subjects

This Human Subjects Research meets the definition of a clinical trial. All investigators will be required to complete the CITI (Collaborative Institutional Training Initiative) program in the ethical use of human participants in research.

16.A. Protection of human subjects from research risks

16.A.1. Risks to the subjects

Human subject involvement and characteristics: We expect to enroll 112 participants, with an equal male to female ratio. We expect to enroll approximately 30 participants who are African-Americans, and another 10 with mixed racial backgrounds, and a few Asian participants given the demographics of the greater New Orleans area. Overall, given the demographics of greater New Orleans, we estimate approximately 10% participants of Hispanic or Latino ethnicity, 50% of them with African-American racial background.

Inclusion criteria

- Men or women ages 40 to 70 years: aim to recruit 50% women
- Any race or ethnicity: aim to recruit 40% ethnic minorities (i.e., African American and Hispanic), close to population in the greater New Orleans area[94]
- HbA1c 6.0–6.9%
- Willing and able to provide informed consent

Exclusion criteria

- Diagnosed type 1 diabetes mellitus based on patient self-report
- Use of agents affecting glycemic control (medications for diabetes, oral glucocorticoids) within the past three months prior to enrollment based on patient self-report
- Medical condition in which low-carbohydrate diet may not be advised
    - Estimated glomerular filtration rate (eGFR) ≤45 mL/min/1.73 m^2, which is close to the 5th percentile of eGFR among non-diseased individuals of 70 years of age[95]
    - Self-report of liver disease due to hepatitis or alcohol; osteoporosis; untreated thyroid disease; gout; or cancer (other than non-melanoma skin cancer) requiring treatment in the past year, unless prognosis is excellent
- Factors that may affect HbA1c:

    - Hemoglobin <11 mg/dL (cutpoint for moderate-to-severe anemia, which could lead to falsely elevated or lowered HbA1c)[96]
    - Recent blood donation or blood transfusion (self-report, past 4 months)
    - Human Immunodeficiency Virus (self-report)[97]
- Allergies to nuts
- For women, current pregnancy, breastfeeding, or plans to become pregnant during the study
- Consumption of ≥21 alcoholic drinks per week or consumption of ≥6 drinks per occasion
- For the CGM collection at the end of the study period only: known allergy to adhesives or other products involved with CGM use (e.g., skin disinfectants), current pregnancy, currently on hemodialysis or peritoneal dialysis, or people with other implanted medical devices (e.g., a pacemaker)
- Current or planned residence making it difficult to meet trial requirements (due to distance from study site and/or challenges regularly traveling to site)
- Current participation in another lifestyle intervention trial or a pharmaceutical trial
- Participation of another household member in the study; employees or persons living with employees of the study
- Other concerns regarding ability to meet trial requirements, at the discretion of the principal investigator or study coordinator

Sources of materials: Medical information data will be collected from the participant and, after written authorization for the disclosure of protected health information, from participants' medical records. Blood and urine samples will be collected to measure HbA1c, glucose, insulin, lipids, creatinine, ketones, and test for pregnancy. Blood pressure, height, weight, waist circumference and other physical measurements will be obtained.

Potential Risks: Risks associated with venipuncture, the diet intervention, CGM insertion, and collection of confidential medical information are the main risks associated with this study. There is a small risk of bruising and a rare chance of local infection associated with standard venipuncture to collect blood samples. The risks of wearing the CGM are minimal. There is a very low risk for local infection at the skin site where the sensor is inserted into the skin. Possible symptoms associated with the CGM sensor application or the adhesive used to keep the sensor in place include skin irritation or redness, skin inflammation, pain or discomfort, bleeding, bruising, swelling, skin rash, itching, scarring or skin discoloration, or allergic reactions to the sensor needle or adhesives. Sensors may fracture in situ on rare occasions. The dietary intervention has minimal risks. Reduced carbohydrate consumption may lead to adverse effects, including constipation, halitosis, fatigue, headache, thirst, polyuria, and nausea. At RZ, 3- , and 6-month visits, we will administer *symptom questionnaires*, which will consist of a checklist to identify participant complaints. Adverse effects of the low-carbohydrate diet will be assessed at counseling sessions. Severe adverse events will be promptly reported to the DSMB. Management of adverse effects will be based on the protection and safety of the participant. If severe adverse effects are reported by a participant, carbohydrate intake will be examined and altered in order to mitigate the adverse effect. In any extreme cases, participants will be instructed to stop the intervention. Furthermore, there is a potential risk of unauthorized disclosure of medical information. This risk will be minimized by using a strict protocol for data processing and by the appropriate training of all study personnel. Protection against risks is described in more detail below.

16.A.2. Adequacy of protection against risks

Recruitment and Informed Consent: The proposed study will be conducted following strict guidelines for the protection of the rights of human volunteers. The informed consent document will be signed by all participants at the screening/baseline visit. The informed consent form will be developed in the first year of the study and approved by the IRB. The consent form will clearly state the purpose, eligibility criteria, and protocol of the study, the potential benefits and risks of participation, and the subject's right to refuse to participate or withdraw. Prior to signing the informed consent, research staff will review the details of the consent form orally with the participant, and answer any questions that the participant has concerning participation in the study. In the case of potential participants with low or no literacy, the informed consent form will be read to the participant in its entirety and any questions will be answered prior to the participant signing or marking the form. Specifically, the following must be accomplished during the informed consent process:

- The participant must be informed that participation in the study is voluntary and that refusal to participate will involve no penalty or loss of benefits.
- The participant must be informed of the purpose of the study and that it involves research.
- The participant must be informed of any alternative procedures, if applicable.
- The participant must be informed of any reasonably foreseeable risks.
- The participant must be informed of any benefits from the research.
- An outline of safeguards to protect participant confidentiality will be included, as well as an indication of the participant's right to withdraw without penalty. This should be balanced with a discussion of the effect that withdrawals will have on the study, and the responsibility a participant has, within limits, to continue in the study if he or she decides to enroll.
- The participant must be informed of his or her right to have questions answered at any time and whom to contact for answers or in the event of research-related injury.
- The participant must be informed as to whether or not compensation is offered for participation in the study and/or in the event of a medical injury.
- The participant must be informed that he/she will be notified of any significant changes in the protocol that might affect their willingness to continue in the study.

Written authorization for the disclosure of protected health information will be obtained during the consent process on a separate form (HIPPA authorization), if applicable.

Protection against Risk: The protocol and informed consent will be approved by the Institutional Review Boards at Tulane University Health Sciences Center and any other participating institution for recruitment. The study participants will be given contact information for the Study Coordinator and Study PI to answer questions or to report complaints. Patients can withdraw from the study at any time without any effect on their medical care. Standard techniques will be used by trained, certified phlebotomists or study nurses for blood sample collection at the study clinics where emergency medical equipment is available. Confidentiality of all data collected during the study will be maintained through the use of unique, encrypted study identification (ID numbers, rather than patient names, in the study database). These encrypted ID numbers will not be mathematical derivation of the subject's medical record number or any other patient identifier. No information will be disclosed in an individually identifiable form in any type of presentation or publication. There will be password-required access to the computerized file linking the study numbers to patient identifiers at the study clinics. Password-access will be restricted to pertinent research staff only. All study data will be kept in locked file cabinets in locked rooms, accessible only by study staff with permission. All

data transmissions will use HIPPA-compliant password protected system and will be secured through a virtual private network.

16.A.3. Potential benefits of the proposed research to human subjects and others

Study participants may have improved glycemic outcomes, blood pressure, lipid levels, may lose weight and reduce waist circumference, and may have improvement in other risk factors for CVD and development and progression of T2D. Participants will receive multiple blood pressure, weight, waist circumference, and blood tests. If vital signs or lab values are clinically abnormal, participants will be referred to their primary care physician, or if they do not have a primary care physician, one will be assigned through the Tulane University Community Health Center, which is a part of the core clinical facilities. At the end of the study, we will provide the participants' primary care physician with a copy of all the blood pressure, weight, laboratory results, and (if applicable) CGM results.

16.A.4. Importance of knowledge to be gained

Information from this study may contribute to reducing the onset of T2D and the burden of T2D and cardiovascular disease in Louisiana and the general US population. The study has potential for public health impact; results from this study may provide evidence in support of promoting alternative dietary styles (than the most commonly used low-fat diet) among individuals with high T2D risk or with T2D.

16.A.5. Remuneration

For screening visit 2, the two follow-up visits, and the final visits, participants will receive $40 gift cards for use at food stores or retailers (after completing each study visit), for a maximum of $120 over the study period. Participants who return a stool sample will receive $25 gift cards for each stool sample that they return, for a total of $75 for three stool samples. For the CGM 6-month study, participants will receive $25 gift cards for returning the CGM, for a maximum of $25. Participants will also receive free parking for each intervention and study visit.

16.B. Data safety and monitoring plan

A DSMB, which consists of 3 experts who are not otherwise affiliated with the COBRE program, has been formed. Study investigators for this study will prepare accurate and timely data tables and reports for the DSMB meetings. The members of the DSMB will serve in an individual capacity and provide their expertise and recommendations. The primary responsibilities of the DSMB are: 1) periodically review and evaluate the accumulated study data for participant safety, study conduct and progress, and, when appropriate, efficacy, and 2) make recommendations to the NIH concerning the continuation, modification, or termination of the trial. The DSMB considers study-specific data, as well as relevant background knowledge, about the disease, test agent, and patient population under study.

The DSMB is responsible for defining its deliberative processes, including event triggers that would call for an unscheduled review, stopping guidelines, unmasking (unblinding) and voting procedures prior to initiating any data review. The DSMB is also responsible for maintaining the confidentiality of its internal discussions and activities, as well as the contents of reports provided to it.

The DSMB will review each protocol for any major concerns prior to implementation. During the trial, the DSMB should review cumulative study data to evaluate safety, study conduct, and scientific validity and integrity of the trial. As part of this responsibility, DSMB members must be satisfied that the timeliness, completeness, and accuracy of the data submitted to them for

review are sufficient for evaluation of the safety and welfare of study participants. The DSMB should also assess the performance of overall study operations and any other relevant issues, as necessary.

Items reviewed by the DSMB include:

- Interim/cumulative data for evidence of study-related adverse events;
- Interim/cumulative data for evidence of efficacy according to pre-established statistical guidelines, if appropriate;
- Data quality, completeness, and timeliness;
- Adequacy of compliance with goals for recruitment and retention, including those related to the participation of women and minorities;
- Adherence to the protocol;
- Factors that might affect the study outcome or compromise the confidentiality of the trial data (such as protocol violations, unmasking, etc.); and,
- Factors external to the study, such as scientific or therapeutic developments, that may impact participant safety or the ethics of the study.

The DSMB will conclude each review with their recommendations to the COBRE program and NIH/NIGMS as to whether the study should continue without change or be modified or terminated. Recommendations regarding modification of the design and conduct of the study could include:

- Modifications of the study protocol based upon review of the safety data;
- Suspension or early termination of the study or of one or more study arms because of serious concerns about subjects' safety, inadequate performance or rate of enrollment;
- Suspension or early termination of the study or of one or more study arms because study objectives have not been obtained according to pre-established statistical guidelines; and,
- Corrective actions regarding a study when the performance appears unsatisfactory or suspicious.

Confidentiality must always be maintained during all phases of DSMB review and deliberation. Usually, only voting members of the DSMB should have access to interim analyses of outcome data by treatment group. Exceptions may be made when the DSMB deems it appropriate. The reason and to whom the exceptions for access to interim analyses is granted will be documented in the Closed Session Report. DSMB members must maintain strict confidentiality concerning all privileged trial results ever provided to them. The DSMB should review data only by masked study group (such as X vs. Y rather than experimental vs. control) unless or until the DSMB determines that the identities of the groups are necessary for their decision-making. Whenever masked data are presented to the DSMB, the key to the group coding must be available for immediate unmasking.

The DSMB will meet twice per year. The junior faculty investigator will attend these meetings and will be responsible for preparing and presenting up-to-date statistical reports on the progress of their trials. These reports will include data on recruitment, randomization, adherence, as well as statistical tests and special analyses requested by the DSMB. The Administrative Core will be responsible for organizing the annual in-person meeting and semiannual conference calls. They will put together data and materials submitted by junior faculty investigators and deliver them to DSMB members. The COBRE investigators will meet with the DSMB in the Open Session and not be present for the Closed Session.

Bibliography and References Cited

1. The Emerging Risk Factors Collaboration. Diabetes mellitus, fasting blood glucose concentration, and risk of vascular disease: a collaborative meta-analysis of 102 prospective studies. *Lancet*. 2010;375(9733):2215-2222. doi:10.1016/S0140-6736(10)60484-9.
2. Flaxman SR, Bourne RRA, Resnikoff S, et al. Global causes of blindness and distance vision impairment 1990-2020: a systematic review and meta-analysis. *Lancet Glob Heal*. October 2017. doi:10.1016/S2214-109X(17)30393-5.
3. United States Renal Data System. *2016 USRDS Annual Data Report: Epidemiology of Kidney Disease in the United States*. Bethesda, MD: National Institutes of Health, National Institute of Diabetes and Digestive and Kidney Diseases; 2016. https://www.usrds.org/2016/view/Default.aspx.
4. Narres M, Kvitkina T, Claessen H, et al. Incidence of lower extremity amputations in the diabetic compared with the non-diabetic population: A systematic review. *PLoS One*. 2017;12(8):e0182081. doi:10.1371/journal.pone.0182081.
5. Centers for Disease Control and Prevention. *National Diabetes Statistics Report, 2017*. Atlanta, GA: Centers for Disease Control and Prevention, U.S. Dept of Health and Human Services; 2017.
6. Centers for Disease Control and Prevention. *Long-Term Trends in Diabetes*. Atlanta, GA: Centers for Disease Control and Prevention, U.S. Dept of Health and Human Services; 2017.
7. NCD Risk Factor Collaboration (NCD-RisC). Worldwide trends in diabetes since 1980: A pooled analysis of 751 population-based studies with 4.4 million participants. *Lancet*. 2016;387(10027):1513-1530. doi:10.1016/S0140-6736(16)00618-8.
8. American Diabetes Association. Economic costs of diabetes in the U.S. in 2012. *Diabetes Care*. 2013;36(4):1033-1046. doi:10.2337/dc12-2625.
9. Zhuo X, Zhang P, Barker L, Albright A, Thompson TJ, Gregg E. The lifetime cost of diabetes and its implications for diabetes prevention. *Diabetes Care*. 2014;37(9):2557-2564. doi:10.2337/dc13-2484.
10. WHO, International Diabetes Foundation. *Definition and Diagnosis of Diabetes Mellitus and Intermediate Hyperglycaemia: Report of a WHO/IDF Consultation*. Geneva: Geneva: World Health Organization; 2006.
11. The International Expert Committee. International Expert Committee Report on the Role of the A1C Assay in the Diagnosis of Diabetes. *Diabetes Care*. 2009;32(7):1327-1334. doi:10.2337/dc09-9033.
12. American Diabetes Association. Standards of Medical Care in Diabetes - 2017. *Diabetes Care*. 2017;40(Supplement 1):S33-S43. doi:10.2337/dc16-S003.
13. Gerstein HC, Santaguida P, Raina P, et al. Annual incidence and relative risk of diabetes in people with various categories of dysglycemia: A systematic overview and meta-analysis of prospective studies. *Diabetes Res Clin Pract*. 2007;78(3):305-312. doi:10.1016/j.diabres.2007.05.004.
14. Huang Y, Cai X, Mai W, Li M, Hu Y. Association between prediabetes and risk of cardiovascular disease and all cause mortality: systematic review and meta-analysis. *BMJ*. 2016;355:i5953. doi:10.1136/bmj.i5953.
15. Tabák AG, Herder C, Rathmann W, Brunner EJ, Kivimäki M. Prediabetes: A high-risk state for diabetes development. *Lancet*. 2012;379(9833):2279-2290. doi:10.1016/S0140-

6736(12)60283-9.

16. Diabetes Prevention Program Research Group. 10-year follow-up of diabetes incidence and weight loss in the Diabetes Prevention Program Outcomes Study. *Lancet*. 2009;374(9702):1677-1686. doi:10.1016/S0140-6736(09)61457-4.
17. The DREAM (Diabetes REduction Assessment with ramipril and rosiglitazone Medication) Trial Investigators. Effect of rosiglitazone on the frequency of diabetes in patients with impaired glucose tolerance or impaired fasting glucose: a randomised controlled trial. *Lancet*. 2006;368(9541):1096-1105. doi:10.1016/S0140-6736(06)69420-8.
18. Perreault L, Pan Q, Mather KJ, Watson KE, Hamman RF, Kahn SE. Effect of regression from prediabetes to normal glucose regulation on long-term reduction in diabetes risk: Results from the Diabetes Prevention Program Outcomes Study. *Lancet*. 2012;379(9833):2243-2251. doi:10.1016/S0140-6736(12)60525-X.
19. Perreault L, Temprosa M, Mather KJ, et al. Regression from prediabetes to normal glucose regulation is associated with reduction in cardiovascular risk: Results from the diabetes prevention program outcomes study. *Diabetes Care*. 2014;37(9):2622-2631. doi:10.2337/dc14-0656.
20. Popkin BM. Nutrition Transition and the Global Diabetes Epidemic. *Curr Diab Rep*. 2015;15(9). doi:10.1007/s11892-015-0631-4.
21. Vazquez G, Duval S, Jacobs DRJ, Silventoinen K. Comparison of body mass index, waist circumference, and waist/hip ratio in predicting incident diabetes: a meta-analysis. *Epidemiol Rev*. 2007;29:115-128. doi:10.1093/epirev/mxm008.
22. Knowler WC, Barrett-Connor E, Fowler SE, et al. Reduction in the incidence of type 2 diabetes with lifestyle intervention or metformin. *N Engl J Med*. 2002;346(6):393-403. doi:10.1056/NEJMoa012512.
23. Pan XR, Li GW, Hu YH, et al. Effects of diet and exercise in preventing NIDDM in people with impaired glucose tolerance: The Da Qing IGT and diabetes study. *Diabetes Care*. 1997;20(4):537-544. doi:10.2337/diacare.20.4.537.
24. Tuomilehto J, Indstrom J, Eriksson J, Valle T, Hamalainine E, Uusitupa M. Prevention of Type 2 Diabetes Mellitus By Changes in Lifestyle Among Subjects With Impaired Glucose Tolerance. *N Engl J Med*. 2001;344(18):1343-1350. doi:10.1056/NEJM200105033441801.
25. Ramachandran A, Snehalatha C, Mary S, Mukesh B, Bhaskar AD, Vijay V. The Indian Diabetes Prevention Programme shows that lifestyle modification and metformin prevent type 2 diabetes in Asian Indian subjects with impaired glucose tolerance (IDPP-1). *Diabetologia*. 2006;49(2):289-297. doi:10.1007/s00125-005-0097-z.
26. The Diabetes Preventio Program Research Group. The Diabetes Prevention Program. Design and methods for a clinical trial in the prevention of type 2 diabetes. *Diabetes Care*. 1999;22(4):623-634. doi:10.2337/diacare.22.4.623 10.2337/diabetes.48.4.699.
27. Evert AB, Boucher JL, Cypress M, et al. Nutrition therapy recommendations for the management of adults with diabetes. *Diabetes Care*. 2013;36(11):3821-3842. doi:10.2337/dc13-2042.
28. Hu T, Mills KT, Yao L, et al. Effects of low-carbohydrate diets versus low-fat diets on metabolic risk factors: A meta-analysis of randomized controlled clinical trials. *Am J Epidemiol*. 2012;176(SUPPL. 7). doi:10.1093/aje/kws264.
29. Sackner-Bernstein J, Kanter D, Kaul S. Dietary Intervention for Overweight and Obese Adults: Comparison of Low-Carbohydrate and Low-Fat Diets. A Meta-Analysis. *PLoS*

One. 2015;10(10):e0139817. doi:10.1371/journal.pone.0139817.

30. Bazzano LA, Hu T, Reynolds K, et al. Effects of low-carbohydrate and low-fat diets: A randomized trial. *Ann Intern Med*. 2014;161(5):309-318. doi:10.7326/M14-0180.
31. Sawyer L, Gale EAM. Diet, delusion and diabetes. *Diabetologia*. 2009;52(1):1-7. doi:10.1007/s00125-008-1203-9.
32. Snorgaard O, Poulsen GM, Andersen HK, Astrup A. Systematic review and meta-analysis of dietary carbohydrate restriction in patients with type 2 diabetes. *BMJ Open Diabetes Res Care*. 2017;5(1):e000354. doi:10.1136/bmjdrc-2016-000354.
33. Larsen RN, Mann NJ, Maclean E, Shaw JE. The effect of high-protein, low-carbohydrate diets in the treatment of type 2 diabetes: A 12 month randomised controlled trial. *Diabetologia*. 2011;54(4):731-740. doi:10.1007/s00125-010-2027-y.
34. Saslow LR, Kim S, Daubenmier JJ, et al. A randomized pilot trial of a moderate carbohydrate diet compared to a very low carbohydrate diet in overweight or obese individuals with type 2 diabetes mellitus or prediabetes. *PLoS One*. 2014;9(4). doi:10.1371/journal.pone.0091027.
35. Tay J, Luscombe-Marsh ND, Thompson CH, et al. A very low-carbohydrate, low-saturated fat diet for type 2 diabetes management: A randomized trial. *Diabetes Care*. 2014;37(11):2909-2918. doi:10.2337/dc14-0845.
36. Davis NJ, Tomuta N, Schechter C, et al. Comparative Study of the Effects of a 1-Year Dietary Intervention of a Low- Carbohydrate Diet Versus a Low-Fat Diet on Weight and Glycemic Control in Type 2. *Diabetes Care*. 2009;32(7):1147-1152. doi:10.2337/dc08-2108.Clinical.
37. Saslow LR, Daubenmier JJ, Moskowitz JT, et al. Twelve-month outcomes of a randomized trial of a moderate-carbohydrate versus very low-carbohydrate diet in overweight adults with type 2 diabetes mellitus or prediabetes. *Nutr Diabetes*. 2017;7(12):304. doi:10.1038/s41387-017-0006-9.
38. Tay J, Thompson CH, Luscombe-Marsh ND, et al. Effects of an energy-restricted low-carbohydrate, high unsaturated fat/low saturated fat diet versus a high carbohydrate, low fat diet in type 2 diabetes: a 2 year randomized clinical trial. *Diabetes, Obes Metab*. 2017:n/a-n/a. doi:10.1111/dom.13164.
39. Yamada Y, Uchida J, Izumi H, et al. A non-calorie-restricted low-carbohydrate diet is effective as an alternative therapy for patients with type 2 diabetes. *Intern Med*. 2014;53(1):13-19. doi:http://dx.doi.org/10.2169/internalmedicine.53.0861.
40. Mayer SB, Jeffreys AS, Olsen MK, Mcduffie JR, Feinglos MN, Yancy WS. Two diets with different haemoglobin A1c and antiglycaemic medication effects despite similar weight loss in type 2 diabetes. *Diabetes, Obes Metab*. 2014;16(1):90-93. doi:10.1111/dom.12191.
41. Maekawa S, Kawahara T, Nomura R, et al. Retrospective study on the efficacy of a low-carbohydrate diet for impaired glucose tolerance. *Diabetes Metab Syndr Obes*. 2014;7:195-201. doi:10.2147/DMSO.S62681.
42. Davis C, Bryan J, Hodgson J, Murphy K. Definition of the mediterranean diet: A literature review. *Nutrients*. 2015;7(11):9139-9153. doi:10.3390/nu7115459.
43. Sofi F, Macchi C, Abbate R, Gensini GF, Casini A. Mediterranean diet and health status: an updated meta-analysis and a proposal for a literature-based adherence score. *Public Health Nutr*. 2014;17(12):2769-2782. doi:10.1017/S1368980013003169.
44. Jannasch F, Kröger J, Schulze MB. Dietary Patterns and Type 2 Diabetes: A Systematic Literature Review and Meta-Analysis of Prospective Studies. *J Nutr*. 2017;147(6):1174-

1182. doi:10.3945/jn.116.242552.

45. Schwingshackl L, Missbach B, König J, Hoffmann G. Adherence to a Mediterranean diet and risk of diabetes: a systematic review and meta-analysis. *Public Health Nutr*. 2014;18(7):1292–1299. doi:10.1017/S1368980014001542.
46. de Lorgeril M, Salen P, Martin J-L, Monjaud I, Delaye J, Mamelle N. Mediterranean Diet, Traditional Risk Factors, and the Rate of Cardiovascular Complications After Myocardial Infarction : Final Report of the Lyon Diet Heart Study. *Circulation*. 1999;99(6):779-785. doi:10.1161/01.CIR.99.6.779.
47. Estruch R, Ros E, Salas-Salvadó J, et al. Primary Prevention of Cardiovascular Disease with a Mediterranean Diet. *N Engl J Med*. 2013;368(14):1279-1290. doi:10.1056/NEJMoa1200303.
48. Salas-Salvadó J, Bulló M, Estruch R, et al. Prevention of diabetes with mediterranean diets: A subgroup analysis of a randomized trial. *Ann Intern Med*. 2014;160(1):1-10. doi:10.7326/M13-1725.
49. Esposito K, Marfella R, Ciotola M, et al. Effect of a Mediterranean-Style Diet on Endothelial Dysfunction and Markers of Vascular Inflammation in the Metabolic Syndrome. *JAMA*. 2004;292(12):1440. doi:10.1001/jama.292.12.1440.
50. Babio N, Toledo E, Estruch R, et al. Mediterranean diets and metabolic syndrome status in the PREDIMED randomized trial. *CMAJ*. 2014;186(17):E649-E657. doi:10.1503/cmaj.140764.
51. Esposito K, Maiorino MI, Bellastella G, Chiodini P, Panagiotakos D, Giugliano D. A journey into a Mediterranean diet and type 2 diabetes: a systematic review with meta-analyses. *BMJ Open*. 2015;5(8):e008222. doi:10.1136/bmjopen-2015-008222.
52. Micha R, Michas G, Mozaffarian D. Unprocessed red and processed meats and risk of coronary artery disease and type 2 diabetes--an updated review of the evidence. *Curr Atheroscler Rep*. 2012;14(6):515-524. doi:10.1007/s11883-012-0282-8.
53. Esposito K, Maiorino MI, Ciotola M, et al. Effects of a Mediterranean-style diet on the need for antihyperglycemic drug therapy in patients with newly diagnosed type 2 diabetes: A randomized trial. *Ann Intern Med*. 2009;151(5):306-314. doi:10.1097/OGX.0b013e3181e5a159.
54. Esposito K, Maiorino MI, Petrizzo M, Bellastella G, Giugliano D. The effects of a Mediterranean diet on the need for diabetes drugs and remission of newly diagnosed type 2 diabetes: Follow-up of a randomized trial. *Diabetes Care*. 2014;37(7):1824-1830. doi:10.2337/dc13-2899.
55. Elhayany A, Lustman A, Abel R, Attal-Singer J, Vinker S. A low carbohydrate Mediterranean diet improves cardiovascular risk factors and diabetes control among overweight patients with type 2 diabetes mellitus: A 1-year prospective randomized intervention study. *Diabetes, Obes Metab*. 2010;12(3):204-209. doi:10.1111/j.1463-1326.2009.01151.x.
56. Shai I, Schwarzfuchs D, Henkin Y, et al. Weight Loss with a Low-Carbohydrate, Mediterranean, or Low-Fat Diet. *N Engl J Med*. 2008;359(3):229-241. doi:10.1056/NEJMoa0708681.
57. Schwarzfuchs D, Golan R, Shai I. Four-Year Follow-up after Two-Year Dietary Interventions. *N Engl J Med*. 2012;367(14):1373-1374. doi:10.1056/NEJMc1204792.
58. Pan A, Sun Q, Bernstein AM, et al. Red meat consumption and risk of type 2 diabetes: 3 Cohorts of US adults and an updated meta-analysis. *Am J Clin Nutr*. 2011;94(4):1088-1096. doi:10.3945/ajcn.111.018978.

59. Hu EA, Pan A, Malik V, Sun Q. White rice consumption and risk of type 2 diabetes: meta-analysis and systematic review. *BMJ*. 2012;344:e1454. doi:10.1136/bmj.e1454.
60. Wang M, Yu M, Fang L, Hu R-Y. Association between sugar-sweetened beverages and type 2 diabetes: A meta-analysis. *J Diabetes Investig*. 2015;6(3):360-366. doi:10.1111/jdi.12309.
61. Imamura F, O'Connor L, Ye Z, et al. Consumption of sugar sweetened beverages, artificially sweetened beverages, and fruit juice and incidence of type 2 diabetes: systematic review, meta-analysis, and estimation of population attributable fraction. *BMJ*. 2015:h3576. doi:10.1136/bmj.h3576.
62. Carter P, Gray LJ, Troughton J, Khunti K, Davies MJ. Fruit and vegetable intake and incidence of type 2 diabetes mellitus: systematic review and meta-analysis. *BMJ*. 2010;341:c4229. doi:10.1136/bmj.c4229.
63. Cooper AJ, Forouhi NG, Ye Z, et al. Fruit and vegetable intake and type 2 diabetes: EPIC-InterAct prospective study and meta-analysis. *Eur J Clin Nutr*. 2012;66(10):1082-1092. doi:10.1038/ejcn.2012.85.
64. Chen M, Sun Q, Giovannucci E, et al. Dairy consumption and risk of type 2 diabetes: 3 cohorts of US adults and an updated meta-analysis. *BMC Med*. 2014;12(1):215. doi:10.1186/s12916-014-0215-1.
65. Aune D, Norat T, Romundstad P, Vatten LJ. Whole grain and refined grain consumption and the risk of type 2 diabetes: A systematic review and dose-response meta-analysis of cohort studies. *Eur J Epidemiol*. 2013;28(11):845-858. doi:10.1007/s10654-013-9852-5.
66. Ding M, Bhupathiraju SN, Chen M, van Dam RM, Hu FB. Caffeinated and decaffeinated coffee consumption and risk of type 2 diabetes: a systematic review and a dose-response meta-analysis. *Diabetes Care*. 2014;37(2):569-586. doi:10.2337/dc13-1203.
67. Li X-H, Yu F-F, Zhou Y-H, He J. Association between alcohol consumption and the risk of incident type 2 diabetes: a systematic review and dose-response meta-analysis. *Am J Clin Nutr*. 2016;103(3):818-829. doi:10.3945/ajcn.115.114389.
68. Muraki I, Imamura F, Manson JE, et al. Fruit consumption and risk of type 2 diabetes: results from three prospective longitudinal cohort studies. *BMJ*. 2013;347(aug28 1):f5001-f5001. doi:10.1136/bmj.f5001.
69. Upadhyaya S, Banerjee G. Type 2 diabetes and gut microbiome: At the intersection of known and unknown. *Gut Microbes*. 2015;6(2):85-92. doi:10.1080/19490976.2015.1024918.
70. David LA, Maurice CF, Carmody RN, et al. Diet rapidly and reproducibly alters the human gut microbiome. *Nature*. 2014;505(7484):559-563. doi:10.1038/nature12820.
71. Duncan SH, Belenguer A, Holtrop G, Johnstone AM, Flint HJ, Lobley GE. Reduced dietary intake of carbohydrates by obese subjects results in decreased concentrations of butyrate and butyrate-producing bacteria in feces. *Appl Environ Microbiol*. 2007;73(4):1073-1078. doi:10.1128/AEM.02340-06.
72. Dorans KS, Mostofsky E, Levitan EB, Håkansson N, Wolk A, Mittleman MA. Alcohol and Incident Heart Failure among Middle-Aged and Elderly Men: Cohort of Swedish Men. *Circ Hear Fail*. 2015;8(3):422-427. doi:10.1161/CIRCHEARTFAILURE.114.001787.
73. Steinhaus DA, Mostofsky E, Levitan EB, et al. Chocolate intake and incidence of heart failure: Findings from the Cohort of Swedish Men. *Am Heart J*. 2017;183:18-23. doi:10.1016/j.ahj.2016.10.002.
74. Dorans KS, Massa J, Chitnis T, Ascherio A, Munger KL. Physical activity and the incidence of multiple sclerosis. *Neurology*. 2016;87(17):1770-1776.

doi:10.1212/WNL.0000000000003260.

75. Mills KT, Chen J, Yang W, et al. Sodium Excretion and the Risk of Cardiovascular Disease in Patients With Chronic Kidney Disease. *JAMA*. 2016;315(20):2200. doi:10.1001/jama.2016.4447.
76. Chen J, Gu D, Huang J, et al. Metabolic syndrome and salt sensitivity of blood pressure in non-diabetic people in China: a dietary intervention study. *Lancet*. 2009;373(9666):829-835. doi:10.1016/S0140-6736(09)60144-6.
77. Li C, He J, Chen J, et al. Genome-Wide Gene-Sodium Interaction Analyses on Blood Pressure: The Genetic Epidemiology Network of Salt-Sensitivity Study. *Hypertension*. 2016;68(2):348-355. doi:10.1161/HYPERTENSIONAHA.115.06765.
78. He J, Streiffer RH, Muntner P, Krousel-Wood MA, Whelton PK. Effect of dietary fiber intake on blood pressure: a randomized, double-blind, placebo-controlled trial. *J Hypertens*. 2004;22(1):73-80. doi:10.1097/01.hjh.0000098164.70956.54.
79. He J, Ogden LG, Vupputuri S, Bazzano LA, Loria C, Whelton PK. Dietary sodium intake and subsequent risk of cardiovascular disease in overweight adults. *JAMA*. 1999;282(21):2027-2034. doi:10.1001/jama.282.21.2027.
80. He J, Wofford MR, Reynolds K, et al. Effect of dietary protein supplementation on blood pressure a randomized, controlled trial. *Circulation*. 2011;124(5):589-595. doi:10.1161/CIRCULATIONAHA.110.009159.
81. Thompson AM, Zhang Y, Tong W, et al. Association of inflammation and endothelial dysfunction with metabolic syndrome, prediabetes and diabetes in adults from Inner Mongolia, China. *BMC Endocr Disord*. 2011;11(1):16. doi:10.1186/1472-6823-11-16.
82. Irazola V, Rubinstein A, Bazzano L, et al. Prevalence, awareness, treatment and control of diabetes and impaired fasting glucose in the Southern Cone of Latin America. *PLoS One*. 2017;12(9). doi:10.1371/journal.pone.0183953.
83. Bazzano LA, Li TY, Joshipura KJ, Hu FB. Intake of fruit, vegetables, and fruit juices and risk of diabetes in women. *Diabetes Care*. 2008;31(7):1311-1317. doi:10.2337/dc08-0080.
84. Bazzano LA, Serdula M, Liu S. Prevention of Type 2 Diabetes by Diet and Lifestyle Modification. *J Am Coll Nutr*. 2005;24(5):310-319. doi:10.1080/07315724.2005.10719479.
85. Pollock BD, Chen W, Harville EW, et al. Differential sex effects of systolic blood pressure and low-density lipoprotein cholesterol on type 2 diabetes: Life course data from the Bogalusa Heart Study. *Journal of Diabetes*. 2017.
86. Zhang T, Li Y, Zhang H, et al. Insulin-sensitive adiposity is associated with a relatively lower risk of diabetes than insulin-resistant adiposity: the Bogalusa Heart Study. *Endocrine*. 2016;54(1):93-100. doi:10.1007/s12020-016-0948-z.
87. Pollock BD, Hu T, Chen W, et al. Utility of existing diabetes risk prediction tools for young black and white adults: Evidence from the Bogalusa Heart Study. *J Diabetes Complications*. 2016;31(1):86-93. doi:10.1016/j.jdiacomp.2016.07.025.
88. Hu T, Yao L, Reynolds K, et al. The effects of a low-carbohydrate diet vs. a low-fat diet on novel cardiovascular risk factors: A randomized controlled trial. *Nutrients*. 2015;7(9):7978-7994. doi:10.3390/nu7095377.
89. Hu T, Yao L, Reynolds K, et al. The effects of a low-carbohydrate diet on appetite: A randomized controlled trial. *Nutr Metab Cardiovasc Dis*. 2016;26(6):476-488. doi:10.1016/j.numecd.2015.11.011.

90. Rebholz CM, Reynolds K, Wofford MR, et al. Effect of soybean protein on novel cardiovascular disease risk factors: A randomized controlled trial. *Eur J Clin Nutr*. 2013;67(1):58-63. doi:10.1038/ejcn.2012.186.

91. Wofford MR, Rebholz CM, Reynolds K, et al. Effect of soy and milk protein supplementation on serum lipid levels: A randomized controlled trial. *Eur J Clin Nutr*. 2012;66(4):419-425. doi:10.1038/ejcn.2011.168.

92. Chen J, He J, Wildman RP, Reynolds K, Streiffer RH, Whelton PK. A randomized controlled trial of dietary fiber intake on serum lipids. *Eur J Clin Nutr*. 2006;60(1):62-68. doi:10.1038/sj.ejcn.1602268.

93. Overweight and Obesity Expert Panel. *Managing Overweight and Obesity in Adults: Systematic Evidence Review From the Obesity Expert Panel, 2013*.; 2013.

94. The Data Center. *Who Lives in New Orleans and Metro Parishes Now?*; 2017. https://www.datacenterresearch.org/data-resources/who-lives-in-new-orleans-now/.

95. Wetzels JFM, Kiemeney LALM, Swinkels DW, Willems HL, Heijer M Den. Age- and gender-specific reference values of estimated GFR in Caucasians: The Nijmegen Biomedical Study. *Kidney Int*. 2007;72(5):632-637. doi:10.1038/sj.ki.5002374.

96. Radin MS. Pitfalls in hemoglobin A1c measurement: When results may be misleading. *J Gen Intern Med*. 2014;29(2):388-394. doi:10.1007/s11606-013-2595-x.

97. Kim PS, Woods C, Georgoff P, et al. A1C underestimates glycemia in HIV infection. *Diabetes Care*. 2009;32(9):1591-1593. doi:10.2337/dc09-0177.

98. Qaseem A, Wilt TJ, Kansagara D, Horwitch C, Barry MJ, Forciea MA. Hemoglobin A1c Targets for Glycemic Control With Pharmacologic Therapy for Nonpregnant Adults With Type 2 Diabetes Mellitus: A Guidance Statement Update From the American College of Physicians. *Ann Intern Med*. 2018;168(8):569-576. doi:10.7326/M17-0939.

99. Sumithran P, Prendergast LA, Delbridge E, et al. Ketosis and appetite-mediating nutrients and hormones after weight loss. *Eur J Clin Nutr*. 2013;67(7):759-764. doi:10.1038/ejcn.2013.90.

100. Gardner CD, Trepanowski JF, Gobbo LCD, et al. Effect of low-fat VS low-carbohydrate diet on 12-month weight loss in overweight adults and the association with genotype pattern or insulin secretion the DIETFITS randomized clinical trial. *JAMA - J Am Med Assoc*. 2018;319(7):667-679. doi:10.1001/jama.2018.0245.

101. Brenner BM, Meyer TW, Hostetter TH. Dietary protein intake and the progressive nature of kidney disease: the role of hemodynamically mediated glomerular injury in the pathogenesis of progressive glomerular sclerosis in aging, renal ablation, and intrinsic renal disease. *N Engl J Med*. 1982;307(11):652-659. doi:10.1056/NEJM198209093071104.

102. Sato J, Kanazawa A, Makita S, et al. A randomized controlled trial of 130 g/day low-carbohydrate diet in type 2 diabetes with poor glycemic control. *Clin Nutr*. 2017;36(4):992-1000. doi:10.1016/j.clnu.2016.07.003.

103. Yancy WS, Foy M, Chalecki AM, et al. A low-carbohydrate, ketogenic diet to treat type 2 diabetes. *Nutr Metab (Lond)*. 2005;2(1):34. doi:10.1186/1743-7075-2-34.

104. Boden G, Sargrad K, Homko C, Mozzoli M, Stein TP. Effect of a low-carbohydrate diet on appetite, blood glucose levels, and insulin resistance in obese patients with type 2 diabetes. *Ann Intern Med*. 2005;142(6). doi:10.1016/S0084-3741(08)70336-6.

105. Stern L, Iqbal N, Seshadri P. The Effects of Low-Carbohydrate versus Conventional Weight Loss Diets in Severely Obese Adults : One-Year Follow-up of a randomized control trial. *Ann Intern …*. 2004;140(May 2001):769-777.

doi:10.1016/j.accreview.2004.07.103.

106. Westman EC, Yancy WS, Edman JS, Tomlin KF, Perkins CE. Effect of 6-month adherence to a very low carbohydrate diet program. *Am J Med*. 2002;113(1):30-36. doi:10.1016/S0002-9343(02)01129-4.

107. Moosheer SM, Waldschütz W, Itariu BK, Brath H, Stulnig TM. A protein-enriched low glycemic index diet with omega-3 polyunsaturated fatty acid supplementation exerts beneficial effects on metabolic control in type 2 diabetes. *Prim Care Diabetes*. 2014;8(4):308-314. doi:10.1016/j.pcd.2014.02.004.

108. Aagaard K, Petrosino J, Keitel W, et al. The Human Microbiome Project strategy for comprehensive sampling of the human microbiome and why it matters. *FASEB J*. 2013;27(3):1012-1022. doi:10.1096/fj.12-220806.

109. McInnes P, Cutting M. Manual of Procedures for Human Microbiome Project. http://www.hmpdacc.org/doc/HMP_MOP_Version12_0_072910.pdf.

110. Marrero DG, Palmer KNB, Phillips EO, Miller-Kovach K, Foster GD, Saha CK. Comparison of commercial and self-initiated weight loss programs in people with prediabetes: A randomized control trial. *Am J Public Health*. 2016;106(5):949-956. doi:10.2105/AJPH.2015.303035.

111. Barengolts E, Manickam B, Eisenberg Y, Akbar A, Kukreja S, Ciubotaru I. EFFECT OF HIGH-DOSE VITAMIN D REPLETION ON GLYCEMIC CONTROL IN AFRICAN-AMERICAN MALES WITH PREDIABETES AND HYPOVITAMINOSIS D. *Endocr Pract*. 2015. doi:10.4158/EP14548.OR.

112. Block G, Azar KMJ, Romanelli RJ, et al. Diabetes prevention and weight loss with a fully automated behavioral intervention by email, web, and mobile phone: A randomized controlled trial among persons with prediabetes. *J Med Internet Res*. 2015. doi:10.2196/jmir.4897.

113. Kramer MK, Molenaar DM, Arena VC, et al. Improving employee health: Evaluation of a worksite lifestyle change program to decrease risk factors for diabetes and cardiovascular disease. *J Occup Environ Med*. 2015. doi:10.1097/JOM.0000000000000350.

114. Parker AR, Byham-Gray L, Denmark R, Winkle PJ. The Effect of Medical Nutrition Therapy by a Registered Dietitian Nutritionist in Patients with Prediabetes Participating in a Randomized Controlled Clinical Research Trial. *J Acad Nutr Diet*. 2014;114(11):1739-1748. doi:10.1016/j.jand.2014.07.020.

115. Maruthur NM, Ma Y, Delahanty LM, et al. Early response to preventive strategies in the diabetes prevention program. *J Gen Intern Med*. 2013;28(12):1629-1636. doi:10.1007/s11606-013-2548-4.

116. Leong A, Daya N, Porneala B, et al. Prediction of Type 2 Diabetes by Hemoglobin A1c in Two Community-Based Cohorts. *Diabetes Care*. 2017;41(1):dc170607. doi:10.2337/dc17-0607.

117. Khaw K-T, Wareham N, Bingham S, Luben R, Welch A, Day N. Association of hemoglobin A1c with cardiovascular disease and mortality in adults: the European prospective investigation into cancer in Norfolk. *Ann Intern Med*. 2004;141(6):413-420. doi:10.7326/0003-4819-141-6-200409210-00006.

118. Cole RE, Boyer KM, Spanbauer SM, Sprague D, Bingham M. Effectiveness of Prediabetes Nutrition Shared Medical Appointments. *Diabetes Educ*. 2013;39(3):344-353. doi:10.1177/0145721713484812.

Low-Carbohydrate Dietary Pattern on Glycemic Outcomes Trial

PROTOCOL

PROTOCOL VERSION 2.0

Dated: February 10, 2020

New Orleans, LA

Table of Contents

1. Overview 5
2. Background and Significance 6
2.A. Type 2 Diabetes Mellitus is a major global public health challenge 6
2.B. Prediabetes is a major risk factor for cardiometabolic diseases 6
2.C. Role of lifestyle in preventing diabetes 6
2.D. Low-to-moderate carbohydrate diets, weight loss, and cardiometabolic diseases 7
2.E. Mediterranean-style diet is associated with reduced risk of T2D and improved glycemic control 8
2.F. Evidence for low-carbohydrate diets that emphasize Mediterranean-style dietary components 8
2.G. Evidence for specific food groups and risk of T2D 8
2.H. Gut microbiota, diet and T2D 9
2.I. Continuous Glucose Monitoring 9
2.I. Clinical and public health significance of the proposed study 9
2.J. Innovation 10
3. Preliminary Studies 10
3.A. Summary of aggregate experience of the research team 10
3.B. Low-carbohydrate behavioral dietary intervention trial experience 11
3.C. Other dietary intervention trial experience 11
4. Study Design and Organization 12
4.A. Overview of trial design 12
4.B. Study outcomes 13
4.B.1. Primary outcome 13
4.B.2. Secondary outcomes 13
4.C Study Timeline 13
6. Study Participants 13
6.A. Eligibility criteria 13
7. Recruitment and Randomization 14
7.A. Recruitment 14
7.A.1. Mass mailing, placement of brochures/flyers in community, TV/radio/billboard advertisements 14
7.A.2. Database searches and targeted mailing 15
7.A.3. Referrals and Local Partnerships 15
7.B. Randomization 15

7.C. Masking 15
7.D. Safety Considerations 16
8. Dietary Intervention Program 16
8.A. Low-carbohydrate dietary intervention group 16
8.B. Usual Diet Group 17
8.C. Physical Activity 17
8.D. Dietary Adherence 17
9. Safety of Dietary Intervention 18
10. Data Collection 18
10.A. Study visits 18
10.A.1. Screening/baseline visits 19
10.A.2. Follow-up visits and final visit 20
10.A.3. CGM 6-month visit 20
10.B Study measurements 21
10.B.1. Questionnaires 21
10.B.2. Dietary measurements 21
10.B.3. Assessment and management of adverse effects 21
10.B.4. BP and anthropometric measures 21
10.B.5. Blood samples 21
10.B.6. Urine collection 22
10.B.7. Stool Specimen Collection 22
10.B.8. CGM at 6 months: 22
11. Quality Assurance and Quality Control 23
11.A. Manual of procedures 23
11.B. Training and certification 23
12. Sample Size and Statistical Power 23
13. Data Management and Analysis 24
13.A. Data management 24
13.B. Data analysis plan 24
13.B.1. Exploratory data analysis 24
13.B.2. Primary, secondary, and exploratory outcomes 24
15. Anticipated Challenges 25
16. Protection of Human Subjects 25

16.A. Protection of human subjects from research risks 25
16.A.1. Risks to the subjects 25
16.A.2. Adequacy of protection against risks 27
16.A.3. Potential benefits of the proposed research to human subjects and others ... 28
16.A.4. Importance of knowledge to be gained 28
16.A.5. Remuneration 28
16.B. Data safety and monitoring plan 29
Bibliography and References Cited 31

1. Overview

The increasing prevalence of type 2 diabetes mellitus (T2D), a major cause of morbidity and mortality worldwide, is a critical public health concern. In the United States, an estimated 33.9% of adults have prediabetes (impaired fasting glucose, elevated hemoglobin A1c (HbA1c), or impaired glucose tolerance). Individuals with prediabetes are at elevated risk of T2D and cardiovascular disease (CVD), with 5–10% developing clinical T2D each year.

In the short-term, among patients with T2D, low-to-moderate carbohydrate diets (energy percentage below 45) have a greater glucose-lowering effect than do high-carbohydrate diets, with larger glucose-lowering effect the greater the carbohydrate reduction. Additionally, compared with low-fat diets, Mediterranean diets that are high in unsaturated fats (particularly *cis*-monounsaturated fats) reduce risk of T2D and CVD and improve glycemic control among diabetics. However, compared with usual diets, the effect of a behavioral intervention promoting a low-carbohydrate/high-unsaturated fat and protein dietary pattern that emphasizes consumption of non-starchy vegetables, plant-based oils, fish, poultry, and mixed nuts on glucose homeostasis, and other metabolic risk factors among individuals with prediabetes or early-stage untreated T2D is not well understood.

Our *long-term goal* is to develop and implement dietary intervention(s) that reduce risk of T2D. The *overall goal of this randomized controlled trial is to study the effect of a behavioral intervention promoting a low-carbohydrate/high-unsaturated fat and high-protein dietary pattern (initial target <40 g digestible carbohydrates, final target <60 g digestible carbohydrates) compared with a usual diet on metabolic risk factors among individuals with prediabetes or early-stage untreated diabetes* (HbA1c 6.0–6.9%). Specifically, we will test the following hypotheses:

Primary hypothesis: Compared with usual diet, over a 6-month period, a healthy low-carbohydrate/high unsaturated fat and high-protein dietary pattern will lead to *a larger decrease of HbA1c, by at least 0.15%.*

Secondary hypothesis: Over a 6-month period, a healthy low-carbohydrate/high-unsaturated fat and high-protein dietary pattern will lead to *larger improvements in the following major secondary outcomes*: fasting glucose, systolic blood pressure (BP), total-to-HDL-cholesterol ratio, and body weight.

Exploratory outcomes: We will also study the effect of the intervention on the 6-month change in: insulin and homeostasis model assessment of insulin resistance (HOMA-IR); diastolic BP; waist circumference; and estimated CVD risk.

To achieve these study aims, we will recruit 126 adults with prediabetes or untreated diabetes (HbA1c 6.0–6.9%) in the Greater New Orleans, LA area. Using a stratified, blocked randomization scheme, participants will be assigned to either a behavioral modification intervention aimed at promoting a healthy low-carbohydrate dietary pattern or to usual diet. The intervention group will have an intensive phase for 3 months, followed by a maintenance phase in months 4 through 6. The *primary outcome will be difference between the active intervention and control groups in the change in HbA1c from baseline to 6 months of follow-up. We expect 80% power to detect a difference of at least 0.15% in change of HbA1c.* Changes in other metabolic risk factors will be studied concurrently. Metabolic risk factors will be assessed at baseline, 3 months, and 6 months of follow-up.

There is much interest regarding dietary approaches to prevent T2D and its complications. The results from this study will help to determine whether, compared with usual diet, a behavioral intervention aimed at promoting a healthy low-carbohydrate dietary pattern improves metabolic risk factors.

2. Background and Significance

2.A. Type 2 Diabetes Mellitus is a major global public health challenge

Diabetes is a strong and independent risk factor for CVD morbidity and mortality. A meta-analysis of 102 prospective studies (698,782 participants) estimated diabetes leads to an approximately 2-fold increased risk in vascular diseases, including coronary heart disease (CHD) and major stroke subtypes, and deaths due to other vascular causes.[1] Among participants with diabetes, CHD risk is about 50% higher among those with fasting blood glucose ≥7 mmol/L compared with those with fasting blood glucose <7 mmol/L.[1] In addition to increasing CVD risk, diabetes can cause diabetic retinopathy,[2] chronic kidney disease,[3] lower limb amputation,[4] and other complications. Most individuals with diabetes have T2D.[5] The US Centers for Disease Control and Prevention (CDC) estimated that 30.2 million adults ≥18 years (12.2%) had diabetes in 2015, with 7.2 million (23.8%) not being aware of or reporting diabetes.[5] Diagnosed diabetes prevalence among US adults has increased substantially over time, from 2.54% in 1980 to 7.40% in 2015.[6] Diabetes prevalence is also high globally, with an estimated 2014 age-standardized prevalence of 9.0% in men and 7.9% in women.[7] Diabetes accounts for an increasing portion of US healthcare expenditures. Total cost of diagnosed diabetes in the US was estimated to be $245 billion in 2012, 41% higher than in 2007.[8] Diabetes is associated with much higher lifetime medical expenses, with higher expenses for those diagnosed at younger ages.[9]

2.B. Prediabetes is a major risk factor for cardiometabolic diseases

Prediabetes, defined by the American Diabetes Association (ADA) as impaired fasting glucose (IFG), impaired glucose tolerance (IGT), or elevated HbA1c, is also referred to as "intermediate hyperglycemia"[10] and "high-risk state of developing diabetes".[11] The ADA defines prediabetes as having: fasting plasma glucose of 100–125 mg/dL (IFG); 2-hour post-load glucose in the 75-g oral glucose tolerance test (OGTT) of 140–199 mg/dL (IGT); or HbA1c 5.7–6.4%.[12] Prediabetes itself is not a disease; for all three tests, "risk is continuous, extending below the lower limit of the range and becoming disproportionately greater at the higher end of the range".[12] The CDC estimated that in 2015, 33.9% of US adults had prediabetes based on fasting glucose or HbA1c.[5] Among those with prediabetes, only 11.9% reported having being told this by a medical professional.[5] Approximately 5–10% of individuals with prediabetes develop diabetes within one year, depending on how prediabetes is defined.[13] In a meta-analysis, annual diabetes incidence was 15–19% among individuals with both IFG and IGT, while it was 4–6% in those with isolated IFG and 6–9% in those with isolated IGT.[13]

A recent meta-analysis (1,611,339 participants from 53 studies) reported that prediabetes as defined by the ADA is associated with higher risk of composite CVD events and CHD.[14] IFG and IGT were also positively associated with increased risk of stroke and all-cause mortality.[14] Prediabetes is linked with increased risk of other conditions typically thought of as diabetes complications, such as neuropathies and nephropathies.[15] Importantly, it is possible for individuals with prediabetes to revert to normoglycemia, as observed in observational studies[13] and lifestyle or drug intervention trials.[16,17] In the Diabetes Prevention Program (DPP), reversion to normoglycemia was associated with lower long-term T2D risk[18] and lower predicted CVD risk.[19]

2.C. Role of lifestyle in preventing diabetes

Much evidence from observational studies and clinical trials supports the critical role of diet and other lifestyle factors in preventing T2D. Globally, lifestyle transitions towards reduced physical activity, increased consumption of refined carbohydrates, coupled with resulting changes in body composition, has led to increases in diabetes prevalence and severity.[20] Body

mass index (BMI), waist circumference, and waist-to-height ratio are predictors of T2D.[21] The DPP, which combined calorie restriction and exercise to promote weight loss, substantially reduced risk of T2D (by 58%) among individuals with elevated FPG and IGT;[22] a benefit that persisted in 10-year follow-up analyses.[16] Other trials of lifestyle modification focused on diet and exercise have also shown benefits in study populations in China,[23] Finland,[24] and India.[25]

2.D. Low-to-moderate carbohydrate diets, weight loss, and cardiometabolic diseases

Low-to-moderate carbohydrate diets (<45% of energy from carbohydrates) are popular dietary approaches for losing weight. Weight loss is a key component of the DPP lifestyle intervention[26] and ADA lifestyle recommendations.[12,27] Meta-analyses from our group[28] and others support that low-to-moderate carbohydrate diets are at least as effective as low-fat diets at promoting weight loss. For instance, a meta-analysis reported a 2-kg (95% CI, 3.1–0.9) larger weight loss in low-carbohydrate diet (<120 g carbohydrates/day) arms compared with low-fat diet (<30% energy from fat) arms.[29] Estimated 10-year CVD risk was lower in low-carbohydrate than low-fat arms.[29] This aligns with research published by our team; in a 12-month trial of participants with obesity, a low-carbohydrate diet (target <40 g carbohydrates/day) was more effective than a low-fat diet (target <30% energy from fat, <7% saturated fat) for weight loss and CVD risk factor reduction.[30]

Low-to-moderate carbohydrate diets may also be beneficial among patients with T2D. Low-carbohydrate diets have come in and out of favor as treatment of diabetes.[31] In a meta-analysis (1376 patients;10 trials), after 3 or 6 months of the intervention, low-to-moderate carbohydrate diets (<45% energy from carbohydrates) led to a 0.34% lower HbA1c (95% CI, 0.06–0.63%) compared with high-carbohydrate diets.[32] Compared with high-carbohydrate diets, there was a greater reduction in HbA1c the greater the reported carbohydrate restriction.[32] There was no benefit of the low-to-moderate carbohydrate diets on HbA1c at 12 months or longer, and no difference between interventions on BMI, body weight, low-density lipoprotein (LDL) cholesterol or quality of life.[32] However, results may have been affected by dietary adherence or glucose medication. There was evidence of diabetes medication reduction in the low-carbohydrate arms in some studies,[33–37] with evidence of medication reduction and improved blood glucose stability persisting for up to 2 years.[38] Hypoglycemia (though not after medication adjustment) was observed in three subjects taking insulin or a sulfonylurea out of twelve subjects in the low-carbohydrate arm of another study.[39] In a subset of participants with T2D (n=46) from a large weight loss trial, compared with those randomized to a low-fat diet and orlistat, those randomized to a low-carbohydrate diet for 48 weeks had improved HbA1c (0.8% lower, 95% CI, -1.5, -0.02%) and a reduction in antiglycemic medications, despite similar weight loss effects of the interventions.[40]

Though low-to-moderate carbohydrate diets have been studied among overweight and obese participants (primarily focused on weight loss), less is understood about the effects of such diets on glycemic outcomes specifically among individuals with prediabetes. A study among participants with IGT observed a reduction at 12 months in weight, HbA1c, and other glycemic outcomes among individuals who participated in a 7-day in-hospital education program, though this was not randomized.[41] A randomized pilot trial of overweight or obese individuals with T2D or prediabetes found that, over 3 months and at 12 months, a very low-carbohydrate diet improved HbA1c, led to higher medication discontinuation, and more weight loss compared with a low-fat diet.[34,37] However, only 4 individuals had prediabetes. Given benefits of low-to-moderate carbohydrate diets for weight loss in general populations and for glycemic control among patients with T2D, further study of these diets are warranted among individuals with prediabetes who are at elevated cardiometabolic disease risk.

2.E. Mediterranean-style diet is associated with reduced risk of T2D and improved glycemic control

Though there is no one “Mediterranean diet”, the Mediterranean dietary pattern tends to have the following characteristics: high consumption of vegetables, fruits, breads and cereals, legumes, nuts and seeds; low-to-moderate consumption of dairy products, fish, poultry and eggs; little consumption of red meat and sweets; and red wine in moderation.[42] Olive oil is an important source of fat. In many observational studies, adherence to the Mediterranean-style dietary pattern is associated with reduced risk of CVD[43] and T2D.[44,45] Evidence from randomized trials also supports the benefits of this dietary pattern. In several trials, participants randomized to Mediterranean diets had substantially lower risk of CVD than those randomized to low-fat control diets.[46,47] In the three-arm randomized PREDIMED trial of participants at high risk of CVD, after 4.1 years of follow-up, those randomized to a traditional Mediterranean diet supplemented with either olive oil or nuts had a 30% reduced risk of T2D compared to those in the low-fat control diet group.[48] In all PREDIMED arms, average carbohydrate intake was relatively low (39.7–43.7% at end of trial).[47] Two trials have found a higher rate of remission from metabolic syndrome among individuals randomized to a Mediterranean diet compared with those randomized to control diets (prudent or low-fat) during 2–5 years of follow-up.[49,50] The Mediterranean pattern may also be beneficial among individuals with T2D. Meta-analyses have reported that, among patients with T2D, those randomized to a Mediterranean diet had lower HbA1c levels and CVD risk factors, and more significant weight loss, compared with those randomized to control diets.[51]

2.F. Evidence for low-carbohydrate diets that emphasize Mediterranean-style dietary components

Many low-carbohydrate diets involve increased consumption of food items, such as red meat,[52] that are associated with increased cardiometabolic disease risk. However, low-carbohydrate dietary approaches can focus on substituting carbohydrates with foods common in Mediterranean-style diets, such as olive oil and nuts, which may improve cardiometabolic health. In a 4-year Italian study of individuals with newly diagnosed T2D, those randomized to a low-carbohydrate Mediterranean diet (<50% carbohydrates; consumed ~44%) had more favorable changes in glycemic control, higher rates of T2D remission, and delayed need for antiglycemic drugs compared with participants randomized to a low-fat diet (<30% fat, <10% saturated fat).[53,54] A 12-month Israeli study assessed low-carbohydrate Mediterranean (35% carbohydrates), traditional Mediterranean, and ADA (both 50–55% carbohydrates) diets in 259 overweight participants with T2D.[55] Most CVD risk factors improved in all groups; there was only HDL improvement in the low-carbohydrate Mediterranean diet and this diet was superior to the other diets in improving glycemic control.[55] In a 2-year weight-loss study of moderately obese participants, low-carbohydrate and Mediterranean diets were superior to low-fat diet for weight loss.[56] Participants in the low-carbohydrate arm were counseled to choose vegetarian sources of fat and protein and to avoid trans fat. At 6 years after study commencement, despite weight regain, there were still long-term benefits, particularly for the low-carbohydrate and Mediterranean diets.[57] In whole, evidence supports that low-carbohydrate Mediterranean-style diets are feasible and may have metabolic benefits.

2.G. Evidence for specific food groups and risk of T2D

Prospective cohort studies have found consumption of specific foods and beverages is associated with diabetes risk. Meta-analyses report higher consumption of processed and unprocessed red meat,[58] white rice (particularly in Asian populations),[59] and sugar-sweetened beverages[60,61] are associated with higher T2D risk. Consumption of green leafy vegetables,[62,63] yogurt,[64] whole grains,[65] decaffeinated and total coffee,[66] and light-to-moderate alcohol

consumption[67] are associated with reduced risk. Consumption of fruits, particularly blueberries, grapes, and apples, are associated with reduced risk; fruit juice is associated with higher risk.[68]

2.H. Gut microbiota, diet and T2D

There is increasing evidence for the importance of gut microbiota in risk of type 2 diabetes and other cardiometabolic diseases.[69] Additionally, dietary modifications, including changes in carbohydrate consumption, can lead to substantial changes in gut microbiome over short periods of time.[70,71] Less is known about longer-term changes to the gut microbiota during the adoption of a low-carbohydrate diet and how these changes may influence cardiometabolic risk. We plan to collect stool specimens at baseline, 3 months, and at 6 months, for potential future studies on how this intervention affects the gut microbiome.

2.I. Continuous Glucose Monitoring

As HbA1c is a marker of weighted average glucose over the past 8 to 12 weeks, it does not explicitly capture short-term glycemic excursions or variability. It has been theorized that such fluctuations may have an independent role in the development of diabetes complications.[3] Though more evidence is needed, research has found relationships of higher glycemic variability with coronary artery disease (CAD) and microvascular outcomes in type 2 diabetes (T2D).[4,5] This suggests that improved control of extreme glucose excursions, particularly postprandially, may help to reduce risks of such complications.[5]

In one study of a low-calorie, low-carbohydrate diet compared with a low-calorie, low-fat behavioral dietary intervention among individuals with T2D, there was evidence that some markers of glycemic variability were further reduced in the low-carbohydrate arm compared with the low-fat arm (assessed by 48-hour continuous glucose monitoring) at 6 months, 1 year, and 2 years.[6–8] However, there is not prior work assessing how a behavioral intervention aimed at reducing carbohydrate consumption affects mean glucose, glucose exposure above the set-point of 100 mg/dL, glucose excursions, or glycemic variability as measured by continuous glucose monitoring among individuals with untreated T2D or at high risk of T2D.

Given this background, we will use continuous glucose monitors (CGM) to collect data for up to 14 days on glucose levels at the end of the 6-months study and will compare mean 24-h glucose among those in the intervention group compared with mean 24-h glucose among those in the control group. We will also measure other markers of glycemic exposure, such as glycemic variability (mean amplitude of glycemic excursions (MAGE), standard deviation of glucose, coefficient of variation) and mean 24-hour glucose during wake-time (6:00 AM to 12:00 AM), and during sleep-time (12:00 AM to 6:00 AM).

2.I. Clinical and public health significance of the proposed study

Prediabetes, or intermediate hyperglycemia, is a marker of high risk of developing T2D and other complications. Due to increasing prevalence of T2D and associated morbidity and mortality globally, a better understanding of dietary approaches to delay progression to T2D or to promote reversion to normoglycemia is a high public health priority. This study proposes to assess the effect of a behavioral intervention promoting a low-carbohydrate/high-unsaturated fat dietary pattern (initial target <40 g digestible carbohydrates, final target <60 g digestible carbohydrates) on HbA1c, a marker of glycemic control, and other metabolic risk factors. Building from prior findings from clinical and observational studies, we will develop a dietary intervention that promotes the consumption of non-starchy vegetables, plant-based oils, fish, poultry, and mixed nuts, that are thought to reduced risk of T2D or CVD. Participants assigned

to the intervention will be trained in behavioral techniques that will provide capacity to maintain dietary changes long after study completion. Our outcome measures will assess the effect of this dietary intervention in a "real-life" setting as opposed to dietary modifications achieved in metabolic ward studies or through food prepared in research kitchens. Findings from this study may provide evidence in support of promoting alternative dietary styles among individuals with high T2D and CVD risk and could potentially be used to inform dietary interventions used in programs like DPP.

2.J. Innovation

The status quo as it pertains to dietary approaches for reducing risk of T2D is focused on reducing caloric intake and total fat intake. However, recent evidence suggests that the types of fat, rather than the quantity of fats may be more important, with evidence supporting that a Mediterranean-style diet that does not restrict fat intake reduces risk of developing both T2D[48] and CVD[47]. Moreover, in the short-term, low-carbohydrate diets, which are popular weight loss diets, have a greater glucose-lowering effect than do high-carbohydrate diets among patients with T2D.[32] However, the effect of low-carbohydrate diets that emphasize consumption of non-starchy vegetables, plant-based oils, fish, poultry, and mixed nuts on T2D prevention is unclear, particularly compared with higher-carbohydrate diets more commonly consumed in the US. *The proposed research is innovative, in our opinion, because it represents a substantial departure from the status quo by assessing the combined effect of a low-carbohydrate, high unsaturated fat and protein diet on cardiometabolic risk factors among adults with prediabetes or early-stage untreated diabetes.* We expect the results from this study have the potential to lead to new horizons for developing and implementing dietary approaches (other than the most commonly used reduced fat diet) that will substantially reduce risk of cardiometabolic disease among adults with T2D or at high risk of T2D. The proposed intervention dietary approach promotes consumption of dietary components thought to reduce risk of cardiometabolic disease and the behavior change intervention has expected applicability in clinical practice.

3. Preliminary Studies

3.A. Summary of aggregate experience of the research team

Dr. Kirsten Dorans is trained in cardiovascular disease epidemiology, with strong training in epidemiologic methods and biostatistics. Her publication record includes studies of behavioral risk factors and risk of chronic disease. She has published on the relationships between dietary intake and chronic heart failure[72,73] and on physical activity and incident multiple sclerosis[74]. She served as lead author, conducting data management, analysis, drafting, and revision of two of these manuscripts. In graduate school, she focused on studying links between outdoor air pollution and chronic diseases and has published several papers in this field. The COBRE program will provide an excellent opportunity for the JFI to transition from air pollution research to clinical and translational research focused on nutrition and diabetes. Building off of her training in observational epidemiology, this program will involve training and practical experience that will allow her to broaden her expertise and to develop practical skills for carrying out clinical research.

The mentorship team has much experience and prior successful collaborations in key areas related to this study. Dr. Jiang He is an internationally renowned expert in cardiometabolic disease clinical and epidemiological research. He has served as PI for many epidemiological studies and clinical trials, including clinical and observational studies focused on dietary consumption and chronic diseases[75–80] and has conducted extensive global research on diabetes.[81,82] Dr. Bazzano has collaborated extensively with Dr. He for more than 15 years in research related to diet and chronic disease. She is a clinical investigator and has expertise in

nutritional epidemiology and clinical research. Dr. Bazzano was the PI of a highly successful clinical trial comparing the effects of low-carbohydrate and low-fat diets on weight and cardiovascular risk factors among individuals with obesity, which was a subproject within the Tulane COBRE in Hypertension and Renal Biology.[30] Dr. He served as Dr. Bazzano's mentor for this clinical trial. They have collaborated on much other work related to dietary and chronic disease, including a meta-analysis on low-carbohydrate diets.[28] Dr. Bazzano has also published many articles on diet and risk of diabetes[83,84] and on diabetes in the Bogalusa Heart Study.[85–87]

3.B. Low-carbohydrate behavioral dietary intervention trial experience

In the study on macronutrient composition of diet and risk factors for cardiovascular disease (MACRO) study, 148 individuals without clinical cardiovascular disease or diabetes and with obesity were randomly assigned to a low-carbohydrate or a low-fat behavioral dietary intervention for 12 months. In the low-carbohydrate arm, daily dietary intake of carbohydrates decreased from an average of 242 g at baseline to 97 g, 93 g, and 127 g at 3, 6, and 12 months, respectively. At 12 months, participants in the low-carbohydrate intervention group had greater decreases in weight (-3.5 kg; 95% CI, -5.6 to -1.4 kg) and fat mass (-1.5%; 95% CI, -2.6% to -0.4%) and greater improvement in several cardiovascular risk factors.[30] This suggests that restricting carbohydrates may be an option for weight loss and reduction of cardiovascular risk factors. Further analyses found that the low-carbohydrate dietary intervention led to similar or greater improvement in inflammation, adipocyte dysfunction, and endothelial dysfunction compared with the low-fat dietary intervention[88] and that the low-fat diet reduced peptide YY more than the low-carbohydrate diet, suggesting satiety may be better preserved on low-carbohydrate diets.[89] No serious adverse events were reported in this study and the number of participants with symptoms were similar between the two diets, with the exception of more participants having headaches on the low-fat diet at 3 months (18 vs 6 participants, $p = 0.03$).[30] Dr. Bazzano served as PI for this study and Dr. He served as her mentor. Dr. Bazzano mentored one of her PhD students in conducting analyses and writing and editing manuscripts related to this project.

3.C. Other dietary intervention trial experience

Dr. He has also led or been involved with many dietary intervention trials, including both behavioral change and feeding study interventions. In the Protein and Blood Pressure (ProBP) study, 352 adults with prehypertension or stage 1 hypertension in New Orleans, LA and Jackson, MS were assigned to take, in a random order, 40 g/d soy protein, milk protein, or carbohydrate supplementation each for 8 weeks. Compared with the high-glycemic index refined carbohydrate, both soy protein and milk protein supplementation were associated with a -2.0 mm Hg (95% CI -3.2 to -0.7 mm Hg) and -2.3 mm Hg (-3.7 to -1.0 mm Hg) net change in systolic blood pressure.[80] These findings suggest that partial replacement of carbohydrate with soy or milk protein might be an important part of nutrition intervention strategies to prevent and treat hypertension. Additionally, soy protein supplementation led to reduction in plasma E-selectin compared with carbohydrate and milk protein and in plasma leptin compared with carbohydrate.[90] Soy protein also led to improvement in lipid profile compared with carbohydrate and milk protein.[91] Dr. He was PI for this project.

In the Fiber Intervention on Blood Pressure (FIBRE) study, 110 participants ages 30–65 years with untreated pre-hypertension or stage-1 hypertension and serum cholesterol level < 240 mg/dl were randomly assigned to 8-g/day of water soluble fiber from oat bran or a control intervention (double-blind RCT). Over the 12-week study period, net changes in systolic and diastolic blood pressure were -1.8 mm Hg (-4.3 to 0.8) and -1.2 mm Hg (-3.0 to 0.5 mm Hg), respectively.[78] Net changes in total, HDL-, and LDL-cholesterol were -2.40 mg/dL (-10.60 to

5.81 mg/d:), -1.66 mg/dL (-4.55 to 1.22 mg/dL), and -1.33 mg/dl (-8.33 to 5.68 mg/dL), respectively.[92] Dr He was PI for this study.

Dr. He is currently serving as a mentor for Dr. Katherine Mills' COBRE project (Effect of Dietary Sodium Reduction in Kidney Disease Patients with Albuminuria; SUPER). In this project, participants with chronic kidney disease and albuminuria are being randomized to a behavioral modification program designed to reduce dietary sodium intake to ≤2,300 mg/day or to usual care and test the effect of this intervention on change in albumin-to-creatinine ratio from baseline to 24-weeks.

4. Study Design and Organization

4.A. Overview of trial design

Figure 1. Study Design

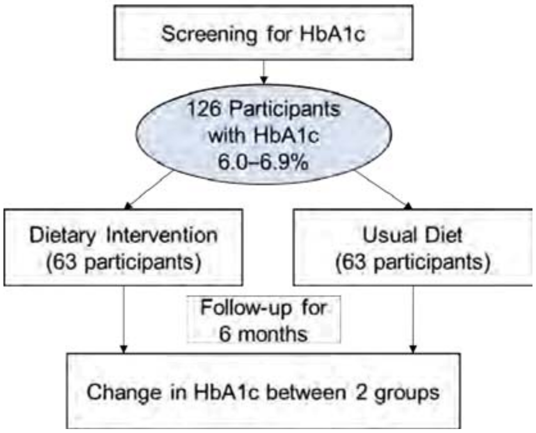

We propose to conduct a *randomized controlled trial* aimed at testing the effect of a behavioral intervention that promotes *reduced carbohydrate intake and increased consumption of heart-healthy unsaturated fats and protein* in patients with HbA1c 6.0–6.9% (*Figure 1*). We plan to recruit 126 individuals with elevated HbA1c and randomly assign them to a moderately intensive intervention on diet pattern,[93] using well-established methods for behavioral modification, or to usual diet. We will compare the difference between the active intervention and control groups for change in HbA1c from baseline to 6 months of follow-up (primary outcome). We will assess four major secondary outcomes: fasting plasma glucose, systolic BP, total-to-HDL cholesterol ratio, and body weight. In addition, we will look at several other exploratory outcomes: insulin and HOMA-IR, diastolic BP, waist circumference, and estimated CVD risk, as estimated by the 2013 ACC/AHA risk equation. The main trial components are summarized in *Table 1*.

Table 1. Study Overview

Study Design	Randomized controlled trial
Study Participants	126 participants with elevated HbA1c (6.0–6.9%)
Intervention Group (n=63)	Behavioral modification to reduce carbohydrate consumption and promote consumption of foods such as non-starchy vegetables, plant-based unsaturated oils, nuts and seeds, avocados, eggs, seafood, poultry, lean meat and tofu. We will recommend moderate consumption of cheese, unsweetened Greek yogurt, and low-carb milk. If consuming higher-carbohydrate items, recommend legumes, fruits, (limited) whole grains. Target <40 g net carbohydrates per day for first 3 months and will have a goal of <40 net g carbohydrates per day; <60 g net carbohydrates per day for months 4 onwards.
Usual Diet Group (n=63)	No dietary intervention. To encourage continued participation in the trial, we will offer optional monthly educational sessions on unrelated topics, such as retirement planning or health insurance
Outcome Measures	Primary: HbA1c; Secondary: fasting plasma glucose, systolic BP, total-to-HDL-cholesterol ratio, and body weight

4.B. Study outcomes

4.B.1. Primary outcome: The *primary outcome will be the difference between the intervention group and the usual diet group for change in HbA1c* from baseline to 6 months. Changes in HbA1c will be calculated from blood samples taken at baseline and 6 months.
4.B.2. Secondary outcomes: Secondary outcomes will include changes from baseline to 6 months of *fasting plasma glucose, systolic BP, total-to-HDL cholesterol ratio, and body weight.,*
4.B.3. Other outcomes: We will also study the effect of the intervention on the following outcomes: insulin and homeostasis model assessment of insulin resistance (HOMA-IR); diastolic BP; waist circumference; total-to-HDL-cholesterol ratio; and estimated CVD risk.

4.C Study Timeline

Table 2 shows the timeline, which will be followed in the implementation of study-related activities, such as publications and submission of an R01 grant application.

Table 2. Study Timeline

	Year 1		Year 2		Year 3	
Develop MOP, intervention program, study forms, and pilot testing	X	X				
Certification and training of staff	X					
Recruitment		X	X			
Intervention and data collection, measurement, entry, cleaning		X	X	X	X	
Data quality control and analysis						X
Publications	X	X	X	X	X	X
R01 grant submissions, resubmissions				X	X	X

The first 6 months will focus on development of manual of operations (MOP), intervention program, study forms, study protocol, Institutional Review Board (IRB) approval, and pilot testing of the intervention program. It will also involve staff training and certification. During the last half of year 1, recruitment, intervention, and data collection will commence. During the last 3 months of year 3, data quality control and analysis will be carried out. Throughout the study period, the JFI will publish peer-reviewed manuscripts from ongoing Tulane University research projects.

6. Study Participants

6.A. Eligibility criteria

Inclusion criteria

- Men or women ages 40 to 70 years: aim to recruit 50% women
- Any race or ethnicity: aim to recruit 40% ethnic minorities (i.e., African American and Hispanic), close to population in the greater New Orleans area[94]
- HbA1c 6.0–6.9%
- Willing and able to provide informed consent

Exclusion criteria

- Diagnosed type 1 diabetes mellitus based on patient self-report
- Use of agents affecting glycemic control (medications for diabetes, oral glucocorticoids) within the past three months prior to enrollment based on patient self-report
- Medical condition in which low-carbohydrate diet may not be advised
    - Estimated glomerular filtration rate (eGFR) ≤45 mL/min/1.73 m^2, which is close to the 5th percentile of eGFR among non-diseased individuals of 70 years of age[95]

- Self-report of liver disease due to hepatitis or alcohol; osteoporosis; untreated thyroid disease; gout; or cancer (other than non-melanoma skin cancer) requiring treatment in the past year, unless prognosis is excellent
- Factors that may affect HbA1c:
  - Hemoglobin <11 mg/dL (cutpoint for moderate-to-severe anemia, which could lead to falsely elevated or lowered HbA1c)[96]
  - Recent blood donation or blood transfusion (self-report, past 4 months)
  - Human Immunodeficiency Virus (self-report)[97]
- Allergies to nuts
- For women, current pregnancy, breastfeeding, or plans to become pregnant during the study
- Consumption of ≥21 alcoholic drinks per week or consumption of ≥6 drinks per occasion
- For the CGM collection at the end of the study period only: known allergy to adhesives or other products involved with CGM use (e.g., skin disinfectants), current pregnancy, currently on hemodialysis or peritoneal dialysis, or people with other implanted medical devices (e.g., a pacemaker)
- Current or planned residence making it difficult to meet trial requirements (due to distance from study site and/or challenges regularly traveling to site)
- Current participation in another lifestyle intervention trial or a pharmaceutical trial
- Participation of another household member in the study; employees or persons living with employees of the study
- Other concerns regarding ability to meet trial requirements, at the discretion of the principal investigator or study coordinator

7. Recruitment and Randomization

7.A. Recruitment

We will recruit study participants from the greater New Orleans area. The initial focus of our recruitment will be mass mailing of individuals in the greater New Orleans area and recruitment through placing flyers and brochures in the community. If community-based recruitment is not successful or cost-effective, we will recruit out-patients from the Ochsner Health System and Tulane Medical Center (TMC), from which our group has prior experience recruiting patients. For this recruitment, we will focus on electronic searches of medical records and laboratory databases. We will also carry out targeted solicitation of referrals from physicians who care for patients likely to meet our inclusion and exclusion criteria.

In addition to this recruitment plan, we have several contingency plans: (1) extending recruitment to other medical centers in the New Orleans area, including the Veterans Affairs Medical Center (VAMC), East Jefferson General Hospital, and West Jefferson Medical Center, with whom Tulane has well-established relationships; (2) TV/radio advertisements; (3) arrange a news story about the study in local TV newscasts, radio talk programs, and/or print media; and (4) conduct outreach at local community events and churches. We have ample experience with each of these approaches.

Based on U.S. Census Bureau 2016 population estimates, the metro New Orleans area population was approximately 35% African American or black and 9% Hispanic.[94] Given our prior experience and population distribution in this region, we plan to recruit a distribution of study participants with 40% ethnic minorities.

7.A.1. Mass mailing, placement of brochures/flyers in community, TV/radio/billboard advertisements: Community recruitment approaches include: mass mailing individuals who live in the New Orleans area; brochures/flyers in the community; emailing the brochure; TV, radio,

poster, or billboard advertisements in the New Orleans area. Interested individuals will be pre-screened; those at elevated risk for prediabetes or T2D, i.e. individuals who are obese or overweight and/or have a sufficiently high score from the American Diabetes Association's Diabetes Risk Assessment Questionnaire will be invited into the clinic for a screening visit.

7.A.2. Database searches and targeted mailing: We will request IRB approval for a waiver for HIPAA authorization to conduct database searching and hospital chart reviews for identification of potentially eligible study participants. After identifying a potentially eligible patient, we will send this individual an invitation letter signed by the investigators and/or the individual's personal physician and brochure via mail or email. Individuals who express interest in the study will have a pre-screening telephone interview for confirmation of potential eligibility. Those still interested and potentially eligible for enrollment will be scheduled for a visit at the COBRE study clinic.

7.A.3. Referrals and Local Partnerships: In addition to local physician referrals, we will explore holding outreach community events in the New Orleans area. Local community partners may post information about the study on their social media platforms. We may also identify potentially interested volunteers from a Tulane volunteer recruitment registry. We will also consider recruiting potentially eligible individuals who were screened or participated in other clinical studies in our clinic and have expressed interest in being contacted about future research projects. Potential participants referred by their physician or community partners will have a pre-screening telephone interview to confirm potential eligibility. Those who are still interested and potentially eligible for the study will be scheduled for a screening visit.

7.B. Randomization

Once eligibility has been confirmed, the study coordinator will ensure that all key variables have been entered into the trial-specific database. Additionally, the study coordinator will ensure any outliers have been recognized and reconciled, and will ensure receipt of informed consent prior to enrolling the volunteer in the study as a trial participant. Eligible participants will be *randomly assigned to the behavioral intervention or the usual diet group in a 1:1 allocation ratio*. Randomization will be stratified by sex, *using a random block group size (4, 6) to maintain balance among groups over time*. A statistician from the Methodology/Biostatistics Unit within the COBRE Clinical Research Core, who is not directly involved in the study and is not located at the clinic, will conduct the randomization. After ascertaining eligibility, clinical personnel will telephone the Methodology/Biostatistics Unit to obtain a randomization assignment. All randomization assignments will be created by a computer program and only accessible to the Staff at the Methodology/Biostatistics Unit. Once randomization assignment has been made, trial participants will be considered to be officially randomized into the study and every effort will be made to obtain complete follow-up information.

7.C. Masking

Clinical and laboratory staff members who assess outcomes will be blinded to the intervention assignment of participants. As this is a dietary intervention trial, study participants, and study staff members who conduct the intervention cannot be blinded to the intervention assignment. Participants will be instructed to avoid sharing knowledge of their treatment assignment with data collection staff members. We will have intervention visits and data collection study visits on different days and in separate locations. Data collectors will also use verbal cues and visual props to remind participants not to disclose treatment assignment. Investigators will be blinded to intervention assignment.

7.D. Safety Considerations

Participants with HbA1c >7.9% will be referred to a clinician for follow-up[98], though we will continue to collect data on these participants.

8. Dietary Intervention Program

8.A. Low-carbohydrate dietary intervention group

Participants randomized to the low-carbohydrate intervention group will receive counseling

aimed at reducing carbohydrate consumption. Phase I (*Go Low*) will occur for the first 3 months, with a goal of <40 g digestible carbohydrates per day. Key recommended foods will include: non-starchy vegetables, fish, poultry, lean meat, eggs, olive oil and other plant-based unsaturated oils, unsweetened/unsalted nuts and seeds, nut butters, and avocados. We will recommend moderate consumption of cheese and unsweetened Greek yogurt and low-carbohydrate milk. During Phase I, we will recommend limiting or avoiding other dairy, fruits, legumes, beans, and grains. Phase II (*Keep it Low*) will run from months 4 through 6. The net carbohydrate goal for this phase will be <40 g to <60 g, with the objective to choose a target that is as low as possible (e.g., as close to <40 g as possible). At 3 months, we expect different participants will have different levels of intake, with some consuming <40 g of net carbohydrates and others consuming levels higher than 60 g net carbohydrates per day. We have designed the intervention to accommodate various scenarios, with the goal of achieving as low of an intake as possible. For participants who consume >60 g per day, we will recommend they aim to either reduce or maintain carbohydrate intake.

At a brief check-in at 3-months, if participants are satisfied with their current intake, we will instruct them to stay at this intake level (though will recommend they try to reduce intake, if possible, if they are eating >60 net grams of carbohydrates or have not yet reached the study goal of <40 net grams of carbohydrates). If they prefer to make modifications, we will provide guidance on how to modify carbohydrate intake (either decrease or increase). If they choose to add back carbohydrates, we will recommend that participants add 5 grams of carbohydrates for two weeks at a time (e.g., from 40 to 45 g for 2 weeks), for a maximum total of <60 g net carbohydrates. If they choose to further reduce carbohydrate intake, we will provide guidance on this as well. As they modify carbohydrate intake, we will have them monitor their satisfaction with their diet and energy levels. If adding back carbohydrates, we will recommend participants add back in foods like fruit (preferably berries), legumes, beans, and dairy, while still aiming to keep total carbohydrate intake as low as possible (e.g., as close to <40 net grams as able).

Materials: We will develop a handbook that will be given to participants. This will contain diet guidelines, recipes, meal planners, shopping lists, and guides for counting carbohydrates and reading nutrition labels.

Meals: Participants will select their own menus and prepare or buy their meals according to the guidelines. We will provide participants with supplemental low-carbohydrate foods, such as nuts, olive oil, and other products, to help facilitate their compliance.

Counseling: Sessions and materials will focus on instructing participants to reduce digestible carbohydrate consumption by increasing consumption of protein and fat, with a particular emphasis on increased consumption of heart-healthy unsaturated fats. After randomization, participants will begin the 3-month *Go Low* phase consisting of individual counseling sessions each week for the first four weeks, followed by 4 group sessions held every other week with phone follow-ups occurring in between group sessions. During the *Keep it Low* phase, months 4 through 6, participants will attend 3 monthly group sessions and have 3 telephone follow-ups. There will be a total of 11 in-person sessions and 7 telephone follow-ups over the full study

period. Sessions will be approximately one hour and phone calls will be no longer than thirty minutes. These sessions will consist of diet instruction and supportive counseling, addressing results from 24-hour dietary recalls, teaching self-monitoring and individual diet goal changes, and provide suggestions for replacing usual foods containing carbohydrates. Study materials will be modified from materials used in the successful MACRO study. The JFI will work closely with Drs. He and Bazzano to develop the intervention based on the MACRO study.
Carbohydrate goal: In the MACRO study, carried out among individuals from the same study region, a behavioral intervention aimed at reducing digestible carbohydrate intake to <40 g/day led to reductions in weight and improvements in cardiovascular risk factors among individuals with obesity.[30] Additionally, restriction of carbohydrate intake to <50g/day is sufficient to induce ketosis.[99] Our rationale for having a multi-phased intervention is to allow participants to see how they feel on the more restrictive diet, and see how they respond to small increases in carbohydrate consumption. A similar technique was applied in the DIETFITS published study.[100] The higher target of 60 net grams may be more sustainable long-term and also allows for consumption of healthy higher-carbohydrate foods, such as berries, legumes, beans, and limited whole grains.

8.B. Usual Diet Group

We will provide participants randomized to the usual diet group written information with standard dietary advice. Participants randomized to the usual diet group will not receive ongoing dietary recommendations. Moreover, they will continue to receive clinical care recommendations from their physician(s). To encourage continued participation in the study and minimize the difference in participant contact with study staff, participants will be offered optional monthly educational sessions on topics unrelated to the study intervention, such as retirement planning or health insurance options.

In another effort to encourage continued participation, patients randomized to the usual diet group will be offered a copy of all available intervention materials, as well as an optional one-hour counseling session with the interventionist following their final study visit.

8.C. Physical Activity

At baseline, both groups will receive written information in study materials with standard physical activity recommendations.

8.D. Dietary Adherence

Strategies for measuring compliance: For the low-carbohydrate diet, adherence will be assessed through measurement of urinary ketones at 3-month visit and 6-month TV. Study staff will make all reasonable effort to maintain adherence of participants to the visit schedule and diet intervention.

Strategies for improving compliance:

- At the screening visit, we will ask participants who does most of the cooking in their households and how often they eat outside of the home. Results from the screening visit will help to select a group of cooperative study participants.
- Visits will be scheduled at the participants' convenience and to minimize waiting time and trips to the clinic; reminder calls, texts or emails (chosen based on participant preference) will also be made prior to clinic visits.
- We will hire enthusiastic staff who have good interpersonal skills.
- Participants will receive $40 gift cards for use at food stores or retailers when they complete each study visit, for a maximum of $120 over the study period.
- Participants will receive $25 gift cards when they return the stool specimens, for a maximum of $75 over the study period.

- Participants will receive a $25 gift card when they return the continuous glucose monitor, for a maximum of $25 over the study period.
- Participants will be offered small gifts (such as pens or magnets with study logo) to encourage compliance and adherence to the study protocol.
- Participants will be provided with recipes, shopping lists, and food samples for the intervention diet.

9. Safety of Dietary Intervention

On the low-carbohydrate diet, participants will be encouraged to primarily focus on replacing carbohydrates (particularly highly refined carbohydrates) with foods such as non-starchy vegetables, olive oil and other plant-based unsaturated oils, nuts and seeds, avocados, eggs, seafood, tofu, poultry, and lean meat. Consumption of more unsaturated fats, protein, and fiber will be recommended. Participants will not be encouraged to increase consumption of saturated fats and will be advised to avoid consumption of trans fats.

In animal studies, a high-protein diet has decreased renal function[101] In the MACRO study, on average, participants tended to increase consumption of fats, rather than protein.[30] The safety and tolerability of low-carbohydrate diets have previously been assessed in many studies, including MACRO.[30] A number of studies, including some in individuals with T2D, have found no clinically significant increases in estimated glomerular filtration rates,[33,38–40] microalbuminuria,[102] albumin excretion rate,[33,38] urinary albumin,[38] serum creatinine,[38,40,102–107] albumin,[40] or uric acid levels.[103–106] A small study of a very low-carbohydrate diet found significant increase in uric acid excretion, but no significant change in 24-hour urine creatinine clearance.[106]

Study staff will carefully monitor participants' BP, and HbA1c more often than what would occur in routine healthcare visits. A Data Safety and Monitoring Board (DSMB) consisting of an independent group of experts in areas such as biostatistics, nutrition medicine, general internal medicine, and endocrinology has been formed. The board will regularly review trial progress, including unblinded interim results, and can recommend that the trial prematurely end if participants are at risk or if it becomes clear that the trial will not be able to yield statistically significant results.

10. Data Collection

10.A. Study visits

After the initial pre-screening interview, in which data on medications and medical conditions will be collected, there will be one screening visit, one randomization visit, a follow-up study visit at 3 months and a termination visit (TV) at 6 months. *Table 3* summarizes study procedures and data collected at each study visit.

Table 3. Study visit schedule and data collection

	Pre-screen	SV	RZ	3 month	6 month TV
Pre-screen eligibility	X				
Informed consent		X			
Compliance form		X			
Eligibility confirmation			X		
Randomization			X		
Demographics		X			
Medical history questionnaire			X	X	X
Medications		X	X	X	X
Lifestyle (alcohol, cigarette smoking)			X	X	X
Physical activity			X	X	X
24-hour diet recall*			X	X	X
3 BP measurements		X	X	X	X
Body weight			X	X	X
Height			X		
Waist circumference			X	X	X
Spot urine test			X	X	X
Blood samples		X	X	X	X
Stool specimen			X	X	X
Side-effects assessment			X	X	X
Nut Consumption					X
CGM**					X

*Two 24-hour dietary recalls will be collected; one should cover a weekend and the other a weekday.
**The CGM will be worn for up to 14 days prior to the 6-month TV and returned at the TV or by mail. Participants will come in for a 6-month CGM visit (prior to the 6-month TV) to have the CGM inserted.

10.A.1. Screening/baseline visits:

Participants will be pre-screened by phone or in-person by study staff to confirm eligibility for the Screening Visit. An approved questionnaire will be used to ensure the patient is eligible to attend the in-clinic screening visit, and the patient will be scheduled thereafter.

At the screening visit (SV), participants will provide informed consent for the screening and main study. Study staff will schedule the SV and randomization (RZ) visit at least 2 days apart. If a participant is eligible based on the screening visit, he or she should be scheduled for the RZ once a sufficient group of eligible individuals has been recruited. Participants should be scheduled for the RZ at least 2 days and no more than two weeks after the screening visit.

At the screening visit, we will collect information on inclusion/exclusion criteria, medication use, and demographics. At this visit, we will also have participants complete a compliance form,

to assess their ability to complete the study and attend all intervention and study visits. We will also measure blood pressure, and take blood samples. Women will be asked if they are postmenopausal or if they had surgical sterilization (such as a tubal ligation). If not, they will be tested for pregnancy through a spot urine test; pregnant women will be excluded from the study. Blood samples will be collected for measurement of HbA1c, and, if not available from medical records in the past 3 months, serum creatinine and hemoglobin. These blood samples will be used for excluding individuals with HbA1c <6.0 or >6.9, eGFR ≤45 mL/min/1.73 m^2, or hemoglobin <11 mg/dL.

Before the start of the intervention, participants will complete two 24-hour dietary recalls. At least one (and potentially both) 24-h recalls will be collected by phone. At the RZ, participants will learn about the randomization process. After requirements for eligibility have been satisfied, participants will formally be enrolled into the study and randomized to either the active intervention or control group. We will collect fasting blood samples for later measurement of glucose, insulin and lipids and other potential future measurements. At this visit, we will measure blood pressure, body weight, height, and waist circumference. We will collect information on medical history, lifestyle risk factors, and physical activity. A spot urine sample will be collected to measure ketones and other markers and participants will be asked to collect a stool specimen for potential use in future microbiome studies.

10.A.2. Follow-up visits and final visit: A follow-up visit will occur at 3 months after randomization. A final visit will occur at 6-months post-randomization. These visits should be scheduled as close as possible to the 3-, or 6-months after randomization. At each visit, study staff will conduct dietary recalls and anthropometric measures, take 3 BP measurements, collect a fasting blood sample to measure HbA1c. At least one (and potentially both) 24-h recalls will be collected by phone. We will also collect samples for later measurement of glucose, insulin, lipid profile, and other potential future measurements. A spot urine sample will be collected to measure ketones and other markers. Participants will be asked to collect a stool specimen for potential use in future microbiome studies. Information about participants' nut consumption over the past 6 months will be collected at the 6-month TV. At study termination, we will mail participants and/or their physicians a summary of trial results and a record of participant BP, anthropometric measures, and laboratory data from the trial period. Throughout the study period, we will provide participants with their blood pressure and weight. We will request that, if possible, a participant who is prescribed a diabetes medication during the course of the study comes into the clinic for an additional separate supplemental visit for a fasting blood sample collection prior to starting the medication. Maintaining follow-up of all randomized participants is of utmost importance to ensure data integrity. In the event that a participant moves during the study we may request that the participant provides a fasting blood sample at a local LabCorp facility (this will be described on the informed consent form).

10.A.3. CGM 6-month visit: Individuals who are willing to wear the CGM prior to their 6-month termination visit will come to the research clinic approximately 2 weeks before their 6-month termination visit for their CGM visit. At this time, they will have a factory-calibrated CGM inserted just under the skin of the upper arm and activated.[27] If the CGM falls off of a participant prior to 14 days of wear, we may request that the participant (if willing) returns to the clinic for the CGM to be re-inserted. The CGM will be returned at the 6-month TV, or by mail.

10.B Study measurements

Measurements obtained for this study will be based on those used in other clinical trials and epidemiologic studies. We are familiar with strengths and limitations of such measurements and the importance of staff training and certification and use of stringent quality control procedures.

10.B.1. Questionnaires: Study staff will administer a *medical history questionnaire* at the RZ, 3-month visit and 6-month TV to assess medical conditions that would necessitate changes in therapy or medical management beyond this study's scope. At the SV, *demographic factors* will be collected. At the SV, RZ, 3-month and 6-month TV, we will also administer *medication tracking questionnaires*. At RZ, 3-month, and 6-month TV, we will collect health behavior and physical activity data.

10.B.2. Dietary measurements: Two 24-hour dietary recalls will be obtained from participants at baseline. At or close to both the 3-month and 6-month TV, two more 24-hour dietary recalls will be collected. The second sets of two recalls will be collected as closely as possible to 3- or 6-month dates after randomization, with a goal of being within 2 weeks of the respective follow-up period. For each time point, one recall should reflect week day consumption, while the other should reflect weekend day consumption and the two recalls should be at least 2 days apart from each other. We will use computer software (Nutrition Data System for Research [NDS-R]) and food models used in previous and ongoing studies. Recalls will be conducted by a study staff member who has been certified in the use of NDS-R. Food composition tables of the NDS-R will be used to calculate dietary nutrient intakes. The baseline dietary assessment will be done to characterize individual dietary intake of carbohydrates and other macronutrients. During the intervention, repeated measurements will be used to monitor short-term changes in mean dietary nutrient intake between the intervention and usual diet groups.

10.B.3. Assessment and management of adverse effects: At RZ, 3-month visit, and 6-month TV, we will administer *symptom questionnaires*, which will consist of a checklist to identify participant complaints. Prior studies of low-carbohydrate dietary interventions suggest that participants may experience a range of adverse effects, including constipation, halitosis, fatigue, headache, thirst, polyuria, and nausea. Severe adverse events will be promptly reported to the DSMB. Management of adverse effects will be based on the protection and safety of the participant. If severe adverse effects are reported by a participant, carbohydrate intake will be examined and altered in order to mitigate the adverse effect. In any extreme cases, participants will be instructed to stop the intervention. As this is a behavioral intervention, adverse effects should be rare.

10.B.4. BP and anthropometric measures: BP will be measured 3 times at the screening visit and at each follow-up visit. To minimize random error, averages of the measurements at the screening and randomization visits will be taken. BP will be measured by a standard automated blood pressure measurement device (OMRON HEM-907 XL Professional Digital Blood Pressure Monitor) and performed by experienced staff, trained and certified in BP measurement. Prior to BP measurements, participants will be asked to rest quietly in a seated position with their feet supported for at least 5 minutes; staff members will use an appropriate cuff size over a bare arm. Trained and certified staff will measure weight using a calibrated balance beam scale at the screening visits, and follow-up visits. Weight and height will be measured at the RZ, and weight will also be measured at the subsequent visits.

10.B.5. Blood samples: For SV, participants will not need to fast. For RZ, 3-month visit, and 6-month TV, participants will be instructed to fast for 12 hours prior to blood sample collection. At SV, blood samples will be collected to measure HbA1c, hemoglobin, and serum creatinine. At RZ, fasting blood samples will be collected and stored as serum/plasma, whole blood aliquots and buffy coats at -80°C for future analyses, including measurement of glucose, insulin and lipids, and potential future metabolomics or genetic analyses. At 3-month visit and 6-month TV,

we will collect fasting blood samples to measure HbA1c and will also store additional serum/plasma, whole blood aliquots and buffy coats at -80°C for future analyses, including measurement of glucose, insulin and lipids, and potential future metabolomics or genetic analyses. Details of the blood sample collection are shown in *Table 4*. In the event that a participant moves, we may request that they collect a fasting blood sample at a local LabCorp and we will measure HbA1c, glucose, insulin, and lipids at the 3 and 6-month visits. HOMA-IR will be calculated from fasting glucose and insulin.

Table 4. Study visit schedule for specimen collection

	SV	RZ	3 month	6 month TV
HbA1c	X		X	X
Serum creatinine	X			
Hemoglobin	X			
Storage of aliquots and buffy coats (for measurement of glucose, insulin, lipids, other potential future measurements)		X	X	X
Urine sample for pregnancy	X			
Urine sample for ketones and other markers		X	X	X
Stool sample		X	X	X
CGM				X

10.B.6. Urine collection: Study staff will collect spot urine specimens at RZ, interim visit, and the final visit to measure ketones.

10.B.7. Stool Specimen Collection: Stool samples will be collected from all eligible and willing trial participants during the RZ, 3-month, and 6-month TV, following the protocol set forth by the Human Microbiome Project.[108,109] Participants will be given a stool collection kit, which includes the specimen collection container, a shipper for home collection, and a form to record the timing and pertinent information related to specimen collection. Participants will be instructed to return the kit to the clinic within two days of collection (in-person or via shipping), at which time stool samples are stored at -80°C until analyses. Kits and instructions for collection will be reviewed by study staff with the participant at the required visits.

10.B.8. CGM at 6 months: Approximately two weeks before their 6-month termination visit, participants willing to wear the CGM will come to the clinic to have their CGM inserted. This CGM has been found to be accurate compared with capillary blood glucose reference values over 14 days of wear.[27] This CGM records interstitial glucose every 15 minutes throughout the day for a period of up to 14 days. The sensor uses a thin, flexible filament inserted just under the skin to measure glucose and is read through near-field communication by touching the reader to the sensor. For quality control of the CGM data, a subset of participants who wear the CGM will be asked to wear two CGMs at the same time, one on each arm. Prior to insertion, one arm will be designated as the arm used for data collection and the other arm as a duplicate.

11. Quality Assurance and Quality Control

Throughout the study, we will implement rigorous quality assurance (QA) and quality control (QC) measures. We describe some of these approaches here.

11.A. Manual of procedures

We will develop a manual of procedures (MOP) based on the MOP successfully used in the MACRO behavior change clinical trial. The MOP will provide instructions and describe procedures for staff training and certification, participant recruitment, intervention counseling, study form completion and procedures, data entry, safety measures (e.g., methods for emergency participant/primary care physician contact).

11.B. Training and certification

Trial personnel will all be required to participate in a training session before the start of any trial procedures or measurements. Training will include particular attention to core trial elements, such as recruitment, retention, intervention, completeness and quality of data acquisition (e.g., blinding procedures for the data collectors), and data entry. Separately, intervention staff will meet and receive detailed instruction on trial approaches to group and individual behavior change counseling and procedures that ensure data collectors are blinded to treatment assignment. Throughout the trial, periodic re-training/certification sessions will be conducted to ensure all trial-related procedures are being applied in a standard manner. Investigators will also monitor all aspects of study performance to ensure study goal achievement, timeliness and completeness of study visits, completeness and quality of data collection, intervention adherence, and adequacy of the procedures separating data collection from intervention. Study staff and investigators will pay particularly careful attention to the quality of all laboratory measurements, particularly key exposure, outcome, and intervention monitoring variables.

12. Sample Size and Statistical Power

The primary outcome will be the difference in the change of HbA1c between the intervention and usual diet groups from baseline to 6 months of follow-up. Preliminary data from ADEPT suggests that the SD of the 6-month change in HbA1c is 0.28%, with preliminary 6-month follow-up >90%. Based on an estimated standard deviation of change in HbA1c of 0.28% and 90% follow-up, we would need 112 participants to have 80% power to detect a difference in the change in HbA1c from baseline to 6 months of 0.15% between the intervention and usual diet groups. Assuming 90% follow-up, we would need 126 total participants.

In a follow-up analysis of the DPP, a 0.15% decrement in HbA1c at 6 months was associated with a 16% lower risk of incident T2D, adjusting for treatment arm, age, sex, race/ethnicity, household income, and baseline HbA1c.[115] In a pooled analysis from the Atherosclerosis Risk in Communities and Framingham Heart Studies, a 0.15% higher HbA1c was associated with an 16% higher risk of T2D over 20 years, a 30% higher risk over 8 years, and a 13% higher risk after 8 years, after adjusting for age, sex, fasting glucose, triglycerides, HDL, family history of T2D, SBP, and BMI.[116] Additionally, in EPIC-Norfork, a difference of HbA1c of 0.15% was associated with approximately a 5% higher risk of CHD among those free of CHD, stroke, diabetes, and with HbA1c <7.0%.[117]

In the DPP study, HbA1c was 0.15% lower among those in the lifestyle intervention arm than in the control arm at 6-months follow-up; HbA1c at study commencement was 5.9% and levels increased in the control arm and decreased in the behavioral intervention arm.[22] In a small behavioral intervention study (n=34) of a very low-carbohydrate ketogenic diet compared with a moderate carbohydrate, calorie-restricted, low-fat diet among individuals with type 2 diabetes or prediabetes (HbA1c >6.0%) and elevated body weight (BMI >25), mean HbA1c

decreased from 6.6% to 6.1% in the very low-carbohydrate group and from 6.9% to 6.7% in the moderate carbohydrate group over a 12-month period; additionally, the very low-carbohydrate group had greater reductions in glucose-lowering medications.[37]

Detectable differences are listed for HbA1c and secondary outcomes in *Table 5*, based on data from prior studies.[30,53,56,118] These were calculated for unpaired t-tests, power of 85%, two-sided alpha of 0.05, and assuming 90% follow-up of 126 participants, which would yield an estimated 112 participants.

Table 5. Minimum detectable differences

Change from baseline to 6 months in	SD	Detectable Difference
HbA1c, %	0.28	0.15
Fasting glucose, mg/dL	12.5	6.7
Fasting insulin, mU/mL	7.0	3.8
HOMA-IR	2.0	1.1
SBP (mmHg)	4.2	2.3
DBP (mmHg)	3.0	1.7
Total/HDL ratio	1.2	0.65
Body weight (kg)	6.6	3.6
Waist circumference (cm)	8.0	4.3
Estimated CVD risk (%)*	6.7	4.0

*10-y CVD risk, among estimated 85% or 95 participants free of CVD

13. Data Management and Analysis

13.A. Data management

Personnel will double-enter all study data. Prior to entering data, the study coordinator will conduct quality control on all study forms, study measures, and laboratory data. Data will be cleaned and logically checked.

13.B. Data analysis plan

The data will be analyzed using *intention to treat*; this involves comparing the primary and secondary outcomes between participants based on randomization assignment.

13.B.1. Exploratory data analysis: We will express group data for variables with a normal distribution as mean ± standard deviation and for variables not normally distributed as median and interquartile ranges (or will log-transform the variables to obtain a normal distribution). We will compare means and percentages of baseline variables across the intervention and usual diet group to assess similarity of these groups at randomization.

13.B.2. Primary, secondary, and exploratory outcomes: We will test the primary study hypothesis that there is a greater reduction in HbA1c from baseline to 6 months in the intervention group than in the usual diet group. To do this, we will use a mixed-effects linear regression model, to test the null hypothesis that there is no difference in change between baseline and 6-month HbA1c for the intervention group compared with the usual diet group (Specific Aim 1). To do this, we will use the following model:

$$Y_{i,j,k}\,T = b_0 \quad b_1G_i \quad b_2T_{3\text{-months}} \quad b_3T_{6\text{-months}} \quad b_4G_iT_{3\text{-months}} \quad b_5G_iT_{6\text{-months}} \quad a_{0i,j} \quad e_{i,j,k}$$

where $Y_{i,j,k}$ is HbA1c for the jth participant (P) nested in the ith intervention group (G) at time T_k (baseline, 3 months, and 6 months). This model includes a random intercept ($a_{0i,j}$) and error term $e_{i,j,k}$. The interaction term ($b_5G_iT_{6\text{-months}}$) allows for an interaction between group and $T_{6\text{-months}}$, which is equal to 0 for baseline and 3-months and 1 for 6-months. Rejection of the null hypothesis (b_5=0) indicates there is a significant difference in change of HbA1c from baseline to 6 months between the intervention and control groups. In this model, participants are assumed to be random effects and intervention group, time, and the interaction terms are fixed effects.

We will use a similar approach to test the difference in the change between baseline and 6-month values of secondary outcomes (Specific Aim 2) and exploratory outcomes. In exploratory analyses, we will also test the difference in the change between baseline and 3-month values of outcomes (HbA1c, fasting glucose, systolic blood pressure, total-to-HDL cholesterol, body weight, insulin, HOMA-IR, diastolic blood pressure, waist circumference, and estimated CVD risk). We will also explore the use of mixed effects models to assess overall net change in HbA1c (by treating time as a linear variable) and other outcomes over the full study period. For each outcome, we will test modeling assumptions and will consider transformation of outcomes, if needed, to meet modeling assumptions. As the mixed-effects model assumes missing at random, we will also perform sensitivity analyses using Markov-chain Monte Carlo techniques to impute missing values of the outcomes, using covariates for this imputation and will compare results from this analysis with our primary findings.

15. Anticipated Challenges

Recruitment: Many clinical trials have challenges recruiting sufficient numbers of volunteers who meet the trial inclusion and exclusion criteria and demonstrate a high level of adherence to trial protocol requirements. Fortunately, the investigators are very experienced with clinical trial recruitment, and have several contingency options that can be used to meet unanticipated shortfall in recruitment.

Intervention: The investigators are aware of and understand challenges related to achieving and maintaining compliance with a behavioral intervention. The investigators have much experience and success in behavioral interventions, with recent success of a behavioral intervention aimed at reducing carbohydrate intake among participants from the New Orleans area. The JFI will collaborate with Drs. He and Bazzano to develop the intervention program based on the MACRO study and will assess potential barriers to implementation and intervention adherence (such as financial concerns and range of dietary patterns of study participants) during the planning and pilot phase of the study.

Adherence: Though adherence and follow-up are also main challenges related to clinical trials, the investigators have an outstanding track record for studies that have recruited participants for both clinical and epidemiology studies in the Greater New Orleans area. To encourage adherence, small gifts (such as pens or magnets with study logo) will be offered to all study participants.

16. Protection of Human Subjects

This Human Subjects Research meets the definition of a clinical trial. All investigators will be required to complete the CITI (Collaborative Institutional Training Initiative) program in the ethical use of human participants in research.

16.A. Protection of human subjects from research risks

16.A.1. Risks to the subjects

Human subject involvement and characteristics: We expect to enroll 126 participants, with an equal male to female ratio. We expect to enroll approximately 30 participants who are African-Americans, and another 10 with mixed racial backgrounds, and a few Asian participants given the demographics of the greater New Orleans area. Overall, given the demographics of greater New Orleans, we estimate approximately 10% participants of Hispanic or Latino ethnicity, 50% of them with African-American racial background.

Inclusion criteria

- Men or women ages 40 to 70 years: aim to recruit 50% women
- Any race or ethnicity: aim to recruit 40% ethnic minorities (i.e., African American and Hispanic), close to population in the greater New Orleans area[94]
- HbA1c 6.0–6.9%
- Willing and able to provide informed consent

Exclusion criteria

- Diagnosed type 1 diabetes mellitus based on patient self-report
- Use of agents affecting glycemic control (medications for diabetes, oral glucocorticoids) within the past three months prior to enrollment based on patient self-report
- Medical condition in which low-carbohydrate diet may not be advised
    - Estimated glomerular filtration rate (eGFR) ≤45 mL/min/1.73 m², which is close to the 5th percentile of eGFR among non-diseased individuals of 70 years of age[95]
    - Self-report of liver disease due to hepatitis or alcohol; osteoporosis; untreated thyroid disease; gout; or cancer (other than non-melanoma skin cancer) requiring treatment in the past year, unless prognosis is excellent
- Factors that may affect HbA1c:
    - Hemoglobin <11 mg/dL (cutpoint for moderate-to-severe anemia, which could lead to falsely elevated or lowered HbA1c)[96]
    - Recent blood donation or blood transfusion (self-report, past 4 months)
    - Human Immunodeficiency Virus (self-report)[97]
- Allergies to nuts
- For women, current pregnancy, breastfeeding, or plans to become pregnant during the study
- Consumption of ≥21 alcoholic drinks per week or consumption of ≥6 drinks per occasion
- For the CGM collection at the end of the study period only: known allergy to adhesives or other products involved with CGM use (e.g., skin disinfectants), current pregnancy, currently on hemodialysis or peritoneal dialysis, or people with other implanted medical devices (e.g., a pacemaker)
- Current or planned residence making it difficult to meet trial requirements (due to distance from study site and/or challenges regularly traveling to site)
- Current participation in another lifestyle intervention trial or a pharmaceutical trial
- Participation of another household member in the study; employees or persons living with employees of the study
- Other concerns regarding ability to meet trial requirements, at the discretion of the principal investigator or study coordinator

Sources of materials: Medical information data will be collected from the participant and, after written authorization for the disclosure of protected health information, from participants' medical records. Blood and urine samples will be collected to measure HbA1c, glucose, insulin, lipids, creatinine, ketones, and test for pregnancy. Blood pressure, height, weight, waist circumference and other physical measurements will be obtained.

Potential Risks: Risks associated with venipuncture, the diet intervention, CGM insertion, and collection of confidential medical information are the main risks associated with this study. There is a small risk of bruising and a rare chance of local infection associated with standard

venipuncture to collect blood samples. The risks of wearing the CGM are minimal. There is a very low risk for local infection at the skin site where the sensor is inserted into the skin. Possible symptoms associated with the CGM sensor application or the adhesive used to keep the sensor in place include skin irritation or redness, skin inflammation, pain or discomfort, bleeding, bruising, swelling, skin rash, itching, scarring or skin discoloration, or allergic reactions to the sensor needle or adhesives. Sensors may fracture in situ on rare occasions. The dietary intervention has minimal risks. Reduced carbohydrate consumption may lead to adverse effects, including constipation, halitosis, fatigue, headache, thirst, polyuria, and nausea. At RZ, 3- , and 6-month visits, we will administer *symptom questionnaires*, which will consist of a checklist to identify participant complaints. Adverse effects of the low-carbohydrate diet will be assessed at counseling sessions. Severe adverse events will be promptly reported to the DSMB. Management of adverse effects will be based on the protection and safety of the participant. If severe adverse effects are reported by a participant, carbohydrate intake will be examined and altered in order to mitigate the adverse effect. In any extreme cases, participants will be instructed to stop the intervention. Furthermore, there is a potential risk of unauthorized disclosure of medical information. This risk will be minimized by using a strict protocol for data processing and by the appropriate training of all study personnel. Protection against risks is described in more detail below.

16.A.2. Adequacy of protection against risks

Recruitment and Informed Consent: The proposed study will be conducted following strict guidelines for the protection of the rights of human volunteers. The informed consent document will be signed by all participants at the screening/baseline visit. The informed consent form will be developed in the first year of the study and approved by the IRB. The consent form will clearly state the purpose, eligibility criteria, and protocol of the study, the potential benefits and risks of participation, and the subject's right to refuse to participate or withdraw. Prior to signing the informed consent, research staff will review the details of the consent form orally with the participant, and answer any questions that the participant has concerning participation in the study. In the case of potential participants with low or no literacy, the informed consent form will be read to the participant in its entirety and any questions will be answered prior to the participant signing or marking the form. Specifically, the following must be accomplished during the informed consent process:

- The participant must be informed that participation in the study is voluntary and that refusal to participate will involve no penalty or loss of benefits.
- The participant must be informed of the purpose of the study and that it involves research.
- The participant must be informed of any alternative procedures, if applicable.
- The participant must be informed of any reasonably foreseeable risks.
- The participant must be informed of any benefits from the research.
- An outline of safeguards to protect participant confidentiality will be included, as well as an indication of the participant’s right to withdraw without penalty. This should be balanced with a discussion of the effect that withdrawals will have on the study, and the responsibility a participant has, within limits, to continue in the study if he or she decides to enroll.
- The participant must be informed of his or her right to have questions answered at any time and whom to contact for answers or in the event of research-related injury.
- The participant must be informed as to whether or not compensation is offered for participation in the study and/or in the event of a medical injury.

- The participant must be informed that he/she will be notified of any significant changes in the protocol that might affect their willingness to continue in the study.

Written authorization for the disclosure of protected health information will be obtained during the consent process on a separate form (HIPPA authorization), if applicable.

Protection against Risk: The protocol and informed consent will be approved by the Institutional Review Boards at Tulane University Health Sciences Center and any other participating institution for recruitment. The study participants will be given contact information for the Study Coordinator and Study PI to answer questions or to report complaints. Patients can withdraw from the study at any time without any effect on their medical care. Standard techniques will be used by trained, certified phlebotomists or study nurses for blood sample collection at the study clinics where emergency medical equipment is available. Confidentiality of all data collected during the study will be maintained through the use of unique, encrypted study identification (ID numbers, rather than patient names, in the study database). These encrypted ID numbers will not be mathematical derivation of the subject's medical record number or any other patient identifier. No information will be disclosed in an individually identifiable form in any type of presentation or publication. There will be password-required access to the computerized file linking the study numbers to patient identifiers at the study clinics. Password-access will be restricted to pertinent research staff only. All study data will be kept in locked file cabinets in locked rooms, accessible only by study staff with permission. All data transmissions will use HIPPA-compliant password protected system and will be secured through a virtual private network.

16.A.3. Potential benefits of the proposed research to human subjects and others

Study participants may have improved glycemic outcomes, blood pressure, lipid levels, may lose weight and reduce waist circumference, and may have improvement in other risk factors for CVD and development and progression of T2D. Participants will receive multiple blood pressure, weight, waist circumference, and blood tests. If vital signs or lab values are clinically abnormal, participants will be referred to their primary care physician, or if they do not have a primary care physician, one will be assigned through the Tulane University Community Health Center, which is a part of the core clinical facilities. At the end of the study, we will provide the participants' primary care physician with a copy of all the blood pressure, weight, laboratory results, and (if applicable) CGM results.

16.A.4. Importance of knowledge to be gained

Information from this study may contribute to reducing the onset of T2D and the burden of T2D and cardiovascular disease in Louisiana and the general US population. The study has potential for public health impact; results from this study may provide evidence in support of promoting alternative dietary styles (than the most commonly used low-fat diet) among individuals with high T2D risk or with T2D.

16.A.5. Remuneration

For screening visit 2, the two follow-up visits, and the final visits, participants will receive $40 gift cards for use at food stores or retailers (after completing each study visit), for a maximum of $120 over the study period. Participants who return a stool sample will receive $25 gift cards for each stool sample that they return, for a total of $75 for three stool samples. For the CGM 6-month study, participants will receive $25 gift cards for returning the CGM, for a maximum of $25. Participants will also receive free parking for each intervention and study visit.

16.B. Data safety and monitoring plan

A DSMB, which consists of 3 experts who are not otherwise affiliated with the COBRE program, has been formed. Study investigators for this study will prepare accurate and timely data tables and reports for the DSMB meetings. The members of the DSMB will serve in an individual capacity and provide their expertise and recommendations. The primary responsibilities of the DSMB are: 1) periodically review and evaluate the accumulated study data for participant safety, study conduct and progress, and, when appropriate, efficacy, and 2) make recommendations to the NIH concerning the continuation, modification, or termination of the trial. The DSMB considers study-specific data, as well as relevant background knowledge, about the disease, test agent, and patient population under study.

The DSMB is responsible for defining its deliberative processes, including event triggers that would call for an unscheduled review, stopping guidelines, unmasking (unblinding) and voting procedures prior to initiating any data review. The DSMB is also responsible for maintaining the confidentiality of its internal discussions and activities, as well as the contents of reports provided to it.

The DSMB will review each protocol for any major concerns prior to implementation. During the trial, the DSMB should review cumulative study data to evaluate safety, study conduct, and scientific validity and integrity of the trial. As part of this responsibility, DSMB members must be satisfied that the timeliness, completeness, and accuracy of the data submitted to them for review are sufficient for evaluation of the safety and welfare of study participants. The DSMB should also assess the performance of overall study operations and any other relevant issues, as necessary.

Items reviewed by the DSMB include:

- Interim/cumulative data for evidence of study-related adverse events;
- Interim/cumulative data for evidence of efficacy according to pre-established statistical guidelines, if appropriate;
- Data quality, completeness, and timeliness;
- Adequacy of compliance with goals for recruitment and retention, including those related to the participation of women and minorities;
- Adherence to the protocol;
- Factors that might affect the study outcome or compromise the confidentiality of the trial data (such as protocol violations, unmasking, etc.); and,
- Factors external to the study, such as scientific or therapeutic developments, that may impact participant safety or the ethics of the study.

The DSMB will conclude each review with their recommendations to the COBRE program and NIH/NIGMS as to whether the study should continue without change or be modified or terminated. Recommendations regarding modification of the design and conduct of the study could include:

- Modifications of the study protocol based upon review of the safety data;
- Suspension or early termination of the study or of one or more study arms because of serious concerns about subjects' safety, inadequate performance or rate of enrollment;
- Suspension or early termination of the study or of one or more study arms because study objectives have not been obtained according to pre-established statistical guidelines; and,
- Corrective actions regarding a study when the performance appears unsatisfactory or suspicious.

Confidentiality must always be maintained during all phases of DSMB review and deliberation. Usually, only voting members of the DSMB should have access to interim analyses of outcome data by treatment group. Exceptions may be made when the DSMB deems it appropriate. The reason and to whom the exceptions for access to interim analyses is granted will be documented in the Closed Session Report. DSMB members must maintain strict confidentiality concerning all privileged trial results ever provided to them. The DSMB should review data only by masked study group (such as X vs. Y rather than experimental vs. control) unless or until the DSMB determines that the identities of the groups are necessary for their decision-making. Whenever masked data are presented to the DSMB, the key to the group coding must be available for immediate unmasking.

The DSMB will meet twice per year. The junior faculty investigator will attend these meetings and will be responsible for preparing and presenting up-to-date statistical reports on the progress of their trials. These reports will include data on recruitment, randomization, adherence, as well as statistical tests and special analyses requested by the DSMB. The Administrative Core will be responsible for organizing the annual in-person meeting and semiannual conference calls. They will put together data and materials submitted by junior faculty investigators and deliver them to DSMB members. The COBRE investigators will meet with the DSMB in the Open Session and not be present for the Closed Session.

Bibliography and References Cited

1. The Emerging Risk Factors Collaboration. Diabetes mellitus, fasting blood glucose concentration, and risk of vascular disease: a collaborative meta-analysis of 102 prospective studies. *Lancet*. 2010;375(9733):2215-2222. doi:10.1016/S0140-6736(10)60484-9.
2. Flaxman SR, Bourne RRA, Resnikoff S, et al. Global causes of blindness and distance vision impairment 1990-2020: a systematic review and meta-analysis. *Lancet Glob Heal*. October 2017. doi:10.1016/S2214-109X(17)30393-5.
3. United States Renal Data System. *2016 USRDS Annual Data Report: Epidemiology of Kidney Disease in the United States*. Bethesda, MD: National Institutes of Health, National Institute of Diabetes and Digestive and Kidney Diseases; 2016. https://www.usrds.org/2016/view/Default.aspx.
4. Narres M, Kvitkina T, Claessen H, et al. Incidence of lower extremity amputations in the diabetic compared with the non-diabetic population: A systematic review. *PLoS One*. 2017;12(8):e0182081. doi:10.1371/journal.pone.0182081.
5. Centers for Disease Control and Prevention. *National Diabetes Statistics Report, 2017*. Atlanta, GA: Centers for Disease Control and Prevention, U.S. Dept of Health and Human Services; 2017.
6. Centers for Disease Control and Prevention. *Long-Term Trends in Diabetes*. Atlanta, GA: Centers for Disease Control and Prevention, U.S. Dept of Health and Human Services; 2017.
7. NCD Risk Factor Collaboration (NCD-RisC). Worldwide trends in diabetes since 1980: A pooled analysis of 751 population-based studies with 4.4 million participants. *Lancet*. 2016;387(10027):1513-1530. doi:10.1016/S0140-6736(16)00618-8.
8. American Diabetes Association. Economic costs of diabetes in the U.S. in 2012. *Diabetes Care*. 2013;36(4):1033-1046. doi:10.2337/dc12-2625.
9. Zhuo X, Zhang P, Barker L, Albright A, Thompson TJ, Gregg E. The lifetime cost of diabetes and its implications for diabetes prevention. *Diabetes Care*. 2014;37(9):2557-2564. doi:10.2337/dc13-2484.
10. WHO, International Diabetes Foundation. *Definition and Diagnosis of Diabetes Mellitus and Intermediate Hyperglycaemia: Report of a WHO/IDF Consultation*. Geneva: Geneva: World Health Organization; 2006.
11. The International Expert Committee. International Expert Committee Report on the Role of the A1C Assay in the Diagnosis of Diabetes. *Diabetes Care*. 2009;32(7):1327-1334. doi:10.2337/dc09-9033.
12. American Diabetes Association. Standards of Medical Care in Diabetes - 2017. *Diabetes Care*. 2017;40(Supplement 1):S33-S43. doi:10.2337/dc16-S003.
13. Gerstein HC, Santaguida P, Raina P, et al. Annual incidence and relative risk of diabetes in people with various categories of dysglycemia: A systematic overview and meta-analysis of prospective studies. *Diabetes Res Clin Pract*. 2007;78(3):305-312. doi:10.1016/j.diabres.2007.05.004.
14. Huang Y, Cai X, Mai W, Li M, Hu Y. Association between prediabetes and risk of cardiovascular disease and all cause mortality: systematic review and meta-analysis. *BMJ*. 2016;355:i5953. doi:10.1136/bmj.i5953.
15. Tabák AG, Herder C, Rathmann W, Brunner EJ, Kivimäki M. Prediabetes: A high-risk state for diabetes development. *Lancet*. 2012;379(9833):2279-2290. doi:10.1016/S0140-

6736(12)60283-9.

16. Diabetes Prevention Program Research Group. 10-year follow-up of diabetes incidence and weight loss in the Diabetes Prevention Program Outcomes Study. *Lancet*. 2009;374(9702):1677-1686. doi:10.1016/S0140-6736(09)61457-4.

17. The DREAM (Diabetes REduction Assessment with ramipril and rosiglitazone Medication) Trial Investigators. Effect of rosiglitazone on the frequency of diabetes in patients with impaired glucose tolerance or impaired fasting glucose: a randomised controlled trial. *Lancet*. 2006;368(9541):1096-1105. doi:10.1016/S0140-6736(06)69420-8.

18. Perreault L, Pan Q, Mather KJ, Watson KE, Hamman RF, Kahn SE. Effect of regression from prediabetes to normal glucose regulation on long-term reduction in diabetes risk: Results from the Diabetes Prevention Program Outcomes Study. *Lancet*. 2012;379(9833):2243-2251. doi:10.1016/S0140-6736(12)60525-X.

19. Perreault L, Temprosa M, Mather KJ, et al. Regression from prediabetes to normal glucose regulation is associated with reduction in cardiovascular risk: Results from the diabetes prevention program outcomes study. *Diabetes Care*. 2014;37(9):2622-2631. doi:10.2337/dc14-0656.

20. Popkin BM. Nutrition Transition and the Global Diabetes Epidemic. *Curr Diab Rep*. 2015;15(9). doi:10.1007/s11892-015-0631-4.

21. Vazquez G, Duval S, Jacobs DRJ, Silventoinen K. Comparison of body mass index, waist circumference, and waist/hip ratio in predicting incident diabetes: a meta-analysis. *Epidemiol Rev*. 2007;29:115-128. doi:10.1093/epirev/mxm008.

22. Knowler WC, Barrett-Connor E, Fowler SE, et al. Reduction in the incidence of type 2 diabetes with lifestyle intervention or metformin. *N Engl J Med*. 2002;346(6):393-403. doi:10.1056/NEJMoa012512.

23. Pan XR, Li GW, Hu YH, et al. Effects of diet and exercise in preventing NIDDM in people with impaired glucose tolerance: The Da Qing IGT and diabetes study. *Diabetes Care*. 1997;20(4):537-544. doi:10.2337/diacare.20.4.537.

24. Tuomilehto J, Indstrom J, Eriksson J, Valle T, Hamalainine E, Uusitupa M. Prevention of Type 2 Diabetes Mellitus By Changes in Lifestyle Among Subjects With Impaired Glucose Tolerance. *N Engl J Med*. 2001;344(18):1343-1350. doi:10.1056/NEJM200105033441801.

25. Ramachandran A, Snehalatha C, Mary S, Mukesh B, Bhaskar AD, Vijay V. The Indian Diabetes Prevention Programme shows that lifestyle modification and metformin prevent type 2 diabetes in Asian Indian subjects with impaired glucose tolerance (IDPP-1). *Diabetologia*. 2006;49(2):289-297. doi:10.1007/s00125-005-0097-z.

26. The Diabetes Preventio Program Research Group. The Diabetes Prevention Program. Design and methods for a clinical trial in the prevention of type 2 diabetes. *Diabetes Care*. 1999;22(4):623-634. doi:10.2337/diacare.22.4.623 10.2337/diabetes.48.4.699.

27. Evert AB, Boucher JL, Cypress M, et al. Nutrition therapy recommendations for the management of adults with diabetes. *Diabetes Care*. 2013;36(11):3821-3842. doi:10.2337/dc13-2042.

28. Hu T, Mills KT, Yao L, et al. Effects of low-carbohydrate diets versus low-fat diets on metabolic risk factors: A meta-analysis of randomized controlled clinical trials. *Am J Epidemiol*. 2012;176(SUPPL. 7). doi:10.1093/aje/kws264.

29. Sackner-Bernstein J, Kanter D, Kaul S. Dietary Intervention for Overweight and Obese Adults: Comparison of Low-Carbohydrate and Low-Fat Diets. A Meta-Analysis. *PLoS*

One. 2015;10(10):e0139817. doi:10.1371/journal.pone.0139817.

30. Bazzano LA, Hu T, Reynolds K, et al. Effects of low-carbohydrate and low-fat diets: A randomized trial. *Ann Intern Med*. 2014;161(5):309-318. doi:10.7326/M14-0180.
31. Sawyer L, Gale EAM. Diet, delusion and diabetes. *Diabetologia*. 2009;52(1):1-7. doi:10.1007/s00125-008-1203-9.
32. Snorgaard O, Poulsen GM, Andersen HK, Astrup A. Systematic review and meta-analysis of dietary carbohydrate restriction in patients with type 2 diabetes. *BMJ Open Diabetes Res Care*. 2017;5(1):e000354. doi:10.1136/bmjdrc-2016-000354.
33. Larsen RN, Mann NJ, Maclean E, Shaw JE. The effect of high-protein, low-carbohydrate diets in the treatment of type 2 diabetes: A 12 month randomised controlled trial. *Diabetologia*. 2011;54(4):731-740. doi:10.1007/s00125-010-2027-y.
34. Saslow LR, Kim S, Daubenmier JJ, et al. A randomized pilot trial of a moderate carbohydrate diet compared to a very low carbohydrate diet in overweight or obese individuals with type 2 diabetes mellitus or prediabetes. *PLoS One*. 2014;9(4). doi:10.1371/journal.pone.0091027.
35. Tay J, Luscombe-Marsh ND, Thompson CH, et al. A very low-carbohydrate, low-saturated fat diet for type 2 diabetes management: A randomized trial. *Diabetes Care*. 2014;37(11):2909-2918. doi:10.2337/dc14-0845.
36. Davis NJ, Tomuta N, Schechter C, et al. Comparative Study of the Effects of a 1-Year Dietary Intervention of a Low- Carbohydrate Diet Versus a Low-Fat Diet on Weight and Glycemic Control in Type 2. *Diabetes Care*. 2009;32(7):1147-1152. doi:10.2337/dc08-2108.Clinical.
37. Saslow LR, Daubenmier JJ, Moskowitz JT, et al. Twelve-month outcomes of a randomized trial of a moderate-carbohydrate versus very low-carbohydrate diet in overweight adults with type 2 diabetes mellitus or prediabetes. *Nutr Diabetes*. 2017;7(12):304. doi:10.1038/s41387-017-0006-9.
38. Tay J, Thompson CH, Luscombe-Marsh ND, et al. Effects of an energy-restricted low-carbohydrate, high unsaturated fat/low saturated fat diet versus a high carbohydrate, low fat diet in type 2 diabetes: a 2 year randomized clinical trial. *Diabetes, Obes Metab*. 2017:n/a-n/a. doi:10.1111/dom.13164.
39. Yamada Y, Uchida J, Izumi H, et al. A non-calorie-restricted low-carbohydrate diet is effective as an alternative therapy for patients with type 2 diabetes. *Intern Med*. 2014;53(1):13-19. doi:http://dx.doi.org/10.2169/internalmedicine.53.0861.
40. Mayer SB, Jeffreys AS, Olsen MK, Mcduffie JR, Feinglos MN, Yancy WS. Two diets with different haemoglobin A1c and antiglycaemic medication effects despite similar weight loss in type 2 diabetes. *Diabetes, Obes Metab*. 2014;16(1):90-93. doi:10.1111/dom.12191.
41. Maekawa S, Kawahara T, Nomura R, et al. Retrospective study on the efficacy of a low-carbohydrate diet for impaired glucose tolerance. *Diabetes Metab Syndr Obes*. 2014;7:195-201. doi:10.2147/DMSO.S62681.
42. Davis C, Bryan J, Hodgson J, Murphy K. Definition of the mediterranean diet: A literature review. *Nutrients*. 2015;7(11):9139-9153. doi:10.3390/nu7115459.
43. Sofi F, Macchi C, Abbate R, Gensini GF, Casini A. Mediterranean diet and health status: an updated meta-analysis and a proposal for a literature-based adherence score. *Public Health Nutr*. 2014;17(12):2769-2782. doi:10.1017/S1368980013003169.
44. Jannasch F, Kröger J, Schulze MB. Dietary Patterns and Type 2 Diabetes: A Systematic Literature Review and Meta-Analysis of Prospective Studies. *J Nutr*. 2017;147(6):1174-

1182. doi:10.3945/jn.116.242552.

45. Schwingshackl L, Missbach B, König J, Hoffmann G. Adherence to a Mediterranean diet and risk of diabetes: a systematic review and meta-analysis. *Public Health Nutr*. 2014;18(7):1292–1299. doi:10.1017/S1368980014001542.
46. de Lorgeril M, Salen P, Martin J-L, Monjaud I, Delaye J, Mamelle N. Mediterranean Diet, Traditional Risk Factors, and the Rate of Cardiovascular Complications After Myocardial Infarction : Final Report of the Lyon Diet Heart Study. *Circulation*. 1999;99(6):779-785. doi:10.1161/01.CIR.99.6.779.
47. Estruch R, Ros E, Salas-Salvadó J, et al. Primary Prevention of Cardiovascular Disease with a Mediterranean Diet. *N Engl J Med*. 2013;368(14):1279-1290. doi:10.1056/NEJMoa1200303.
48. Salas-Salvadó J, Bulló M, Estruch R, et al. Prevention of diabetes with mediterranean diets: A subgroup analysis of a randomized trial. *Ann Intern Med*. 2014;160(1):1-10. doi:10.7326/M13-1725.
49. Esposito K, Marfella R, Ciotola M, et al. Effect of a Mediterranean-Style Diet on Endothelial Dysfunction and Markers of Vascular Inflammation in the Metabolic Syndrome. *JAMA*. 2004;292(12):1440. doi:10.1001/jama.292.12.1440.
50. Babio N, Toledo E, Estruch R, et al. Mediterranean diets and metabolic syndrome status in the PREDIMED randomized trial. *CMAJ*. 2014;186(17):E649-E657. doi:10.1503/cmaj.140764.
51. Esposito K, Maiorino MI, Bellastella G, Chiodini P, Panagiotakos D, Giugliano D. A journey into a Mediterranean diet and type 2 diabetes: a systematic review with meta-analyses. *BMJ Open*. 2015;5(8):e008222. doi:10.1136/bmjopen-2015-008222.
52. Micha R, Michas G, Mozaffarian D. Unprocessed red and processed meats and risk of coronary artery disease and type 2 diabetes--an updated review of the evidence. *Curr Atheroscler Rep*. 2012;14(6):515-524. doi:10.1007/s11883-012-0282-8.
53. Esposito K, Maiorino MI, Ciotola M, et al. Effects of a Mediterranean-style diet on the need for antihyperglycemic drug therapy in patients with newly diagnosed type 2 diabetes: A randomized trial. *Ann Intern Med*. 2009;151(5):306-314. doi:10.1097/OGX.0b013e3181e5a159.
54. Esposito K, Maiorino MI, Petrizzo M, Bellastella G, Giugliano D. The effects of a Mediterranean diet on the need for diabetes drugs and remission of newly diagnosed type 2 diabetes: Follow-up of a randomized trial. *Diabetes Care*. 2014;37(7):1824-1830. doi:10.2337/dc13-2899.
55. Elhayany A, Lustman A, Abel R, Attal-Singer J, Vinker S. A low carbohydrate Mediterranean diet improves cardiovascular risk factors and diabetes control among overweight patients with type 2 diabetes mellitus: A 1-year prospective randomized intervention study. *Diabetes, Obes Metab*. 2010;12(3):204-209. doi:10.1111/j.1463-1326.2009.01151.x.
56. Shai I, Schwarzfuchs D, Henkin Y, et al. Weight Loss with a Low-Carbohydrate, Mediterranean, or Low-Fat Diet. *N Engl J Med*. 2008;359(3):229-241. doi:10.1056/NEJMoa0708681.
57. Schwarzfuchs D, Golan R, Shai I. Four-Year Follow-up after Two-Year Dietary Interventions. *N Engl J Med*. 2012;367(14):1373-1374. doi:10.1056/NEJMc1204792.
58. Pan A, Sun Q, Bernstein AM, et al. Red meat consumption and risk of type 2 diabetes: 3 Cohorts of US adults and an updated meta-analysis. *Am J Clin Nutr*. 2011;94(4):1088-1096. doi:10.3945/ajcn.111.018978.

59. Hu EA, Pan A, Malik V, Sun Q. White rice consumption and risk of type 2 diabetes: meta-analysis and systematic review. *BMJ*. 2012;344:e1454. doi:10.1136/bmj.e1454.
60. Wang M, Yu M, Fang L, Hu R-Y. Association between sugar-sweetened beverages and type 2 diabetes: A meta-analysis. *J Diabetes Investig*. 2015;6(3):360-366. doi:10.1111/jdi.12309.
61. Imamura F, O'Connor L, Ye Z, et al. Consumption of sugar sweetened beverages, artificially sweetened beverages, and fruit juice and incidence of type 2 diabetes: systematic review, meta-analysis, and estimation of population attributable fraction. *BMJ*. 2015:h3576. doi:10.1136/bmj.h3576.
62. Carter P, Gray LJ, Troughton J, Khunti K, Davies MJ. Fruit and vegetable intake and incidence of type 2 diabetes mellitus: systematic review and meta-analysis. *BMJ*. 2010;341:c4229. doi:10.1136/bmj.c4229.
63. Cooper AJ, Forouhi NG, Ye Z, et al. Fruit and vegetable intake and type 2 diabetes: EPIC-InterAct prospective study and meta-analysis. *Eur J Clin Nutr*. 2012;66(10):1082-1092. doi:10.1038/ejcn.2012.85.
64. Chen M, Sun Q, Giovannucci E, et al. Dairy consumption and risk of type 2 diabetes: 3 cohorts of US adults and an updated meta-analysis. *BMC Med*. 2014;12(1):215. doi:10.1186/s12916-014-0215-1.
65. Aune D, Norat T, Romundstad P, Vatten LJ. Whole grain and refined grain consumption and the risk of type 2 diabetes: A systematic review and dose-response meta-analysis of cohort studies. *Eur J Epidemiol*. 2013;28(11):845-858. doi:10.1007/s10654-013-9852-5.
66. Ding M, Bhupathiraju SN, Chen M, van Dam RM, Hu FB. Caffeinated and decaffeinated coffee consumption and risk of type 2 diabetes: a systematic review and a dose-response meta-analysis. *Diabetes Care*. 2014;37(2):569-586. doi:10.2337/dc13-1203.
67. Li X-H, Yu F-F, Zhou Y-H, He J. Association between alcohol consumption and the risk of incident type 2 diabetes: a systematic review and dose-response meta-analysis. *Am J Clin Nutr*. 2016;103(3):818-829. doi:10.3945/ajcn.115.114389.
68. Muraki I, Imamura F, Manson JE, et al. Fruit consumption and risk of type 2 diabetes: results from three prospective longitudinal cohort studies. *BMJ*. 2013;347(aug28 1):f5001-f5001. doi:10.1136/bmj.f5001.
69. Upadhyaya S, Banerjee G. Type 2 diabetes and gut microbiome: At the intersection of known and unknown. *Gut Microbes*. 2015;6(2):85-92. doi:10.1080/19490976.2015.1024918.
70. David LA, Maurice CF, Carmody RN, et al. Diet rapidly and reproducibly alters the human gut microbiome. *Nature*. 2014;505(7484):559-563. doi:10.1038/nature12820.
71. Duncan SH, Belenguer A, Holtrop G, Johnstone AM, Flint HJ, Lobley GE. Reduced dietary intake of carbohydrates by obese subjects results in decreased concentrations of butyrate and butyrate-producing bacteria in feces. *Appl Environ Microbiol*. 2007;73(4):1073-1078. doi:10.1128/AEM.02340-06.
72. Dorans KS, Mostofsky E, Levitan EB, Håkansson N, Wolk A, Mittleman MA. Alcohol and Incident Heart Failure among Middle-Aged and Elderly Men: Cohort of Swedish Men. *Circ Hear Fail*. 2015;8(3):422-427. doi:10.1161/CIRCHEARTFAILURE.114.001787.
73. Steinhaus DA, Mostofsky E, Levitan EB, et al. Chocolate intake and incidence of heart failure: Findings from the Cohort of Swedish Men. *Am Heart J*. 2017;183:18-23. doi:10.1016/j.ahj.2016.10.002.
74. Dorans KS, Massa J, Chitnis T, Ascherio A, Munger KL. Physical activity and the incidence of multiple sclerosis. *Neurology*. 2016;87(17):1770-1776.

doi:10.1212/WNL.0000000000003260.

75. Mills KT, Chen J, Yang W, et al. Sodium Excretion and the Risk of Cardiovascular Disease in Patients With Chronic Kidney Disease. *JAMA*. 2016;315(20):2200. doi:10.1001/jama.2016.4447.

76. Chen J, Gu D, Huang J, et al. Metabolic syndrome and salt sensitivity of blood pressure in non-diabetic people in China: a dietary intervention study. *Lancet*. 2009;373(9666):829-835. doi:10.1016/S0140-6736(09)60144-6.

77. Li C, He J, Chen J, et al. Genome-Wide Gene-Sodium Interaction Analyses on Blood Pressure: The Genetic Epidemiology Network of Salt-Sensitivity Study. *Hypertension*. 2016;68(2):348-355. doi:10.1161/HYPERTENSIONAHA.115.06765.

78. He J, Streiffer RH, Muntner P, Krousel-Wood MA, Whelton PK. Effect of dietary fiber intake on blood pressure: a randomized, double-blind, placebo-controlled trial. *J Hypertens*. 2004;22(1):73-80. doi:10.1097/01.hjh.0000098164.70956.54.

79. He J, Ogden LG, Vupputuri S, Bazzano LA, Loria C, Whelton PK. Dietary sodium intake and subsequent risk of cardiovascular disease in overweight adults. *JAMA*. 1999;282(21):2027-2034. doi:10.1001/jama.282.21.2027.

80. He J, Wofford MR, Reynolds K, et al. Effect of dietary protein supplementation on blood pressure a randomized, controlled trial. *Circulation*. 2011;124(5):589-595. doi:10.1161/CIRCULATIONAHA.110.009159.

81. Thompson AM, Zhang Y, Tong W, et al. Association of inflammation and endothelial dysfunction with metabolic syndrome, prediabetes and diabetes in adults from Inner Mongolia, China. *BMC Endocr Disord*. 2011;11(1):16. doi:10.1186/1472-6823-11-16.

82. Irazola V, Rubinstein A, Bazzano L, et al. Prevalence, awareness, treatment and control of diabetes and impaired fasting glucose in the Southern Cone of Latin America. *PLoS One*. 2017;12(9). doi:10.1371/journal.pone.0183953.

83. Bazzano LA, Li TY, Joshipura KJ, Hu FB. Intake of fruit, vegetables, and fruit juices and risk of diabetes in women. *Diabetes Care*. 2008;31(7):1311-1317. doi:10.2337/dc08-0080.

84. Bazzano LA, Serdula M, Liu S. Prevention of Type 2 Diabetes by Diet and Lifestyle Modification. *J Am Coll Nutr*. 2005;24(5):310-319. doi:10.1080/07315724.2005.10719479.

85. Pollock BD, Chen W, Harville EW, et al. Differential sex effects of systolic blood pressure and low-density lipoprotein cholesterol on type 2 diabetes: Life course data from the Bogalusa Heart Study. *Journal of Diabetes*. 2017.

86. Zhang T, Li Y, Zhang H, et al. Insulin-sensitive adiposity is associated with a relatively lower risk of diabetes than insulin-resistant adiposity: the Bogalusa Heart Study. *Endocrine*. 2016;54(1):93-100. doi:10.1007/s12020-016-0948-z.

87. Pollock BD, Hu T, Chen W, et al. Utility of existing diabetes risk prediction tools for young black and white adults: Evidence from the Bogalusa Heart Study. *J Diabetes Complications*. 2016;31(1):86-93. doi:10.1016/j.jdiacomp.2016.07.025.

88. Hu T, Yao L, Reynolds K, et al. The effects of a low-carbohydrate diet vs. a low-fat diet on novel cardiovascular risk factors: A randomized controlled trial. *Nutrients*. 2015;7(9):7978-7994. doi:10.3390/nu7095377.

89. Hu T, Yao L, Reynolds K, et al. The effects of a low-carbohydrate diet on appetite: A randomized controlled trial. *Nutr Metab Cardiovasc Dis*. 2016;26(6):476-488. doi:10.1016/j.numecd.2015.11.011.

90. Rebholz CM, Reynolds K, Wofford MR, et al. Effect of soybean protein on novel cardiovascular disease risk factors: A randomized controlled trial. *Eur J Clin Nutr*. 2013;67(1):58-63. doi:10.1038/ejcn.2012.186.
91. Wofford MR, Rebholz CM, Reynolds K, et al. Effect of soy and milk protein supplementation on serum lipid levels: A randomized controlled trial. *Eur J Clin Nutr*. 2012;66(4):419-425. doi:10.1038/ejcn.2011.168.
92. Chen J, He J, Wildman RP, Reynolds K, Streiffer RH, Whelton PK. A randomized controlled trial of dietary fiber intake on serum lipids. *Eur J Clin Nutr*. 2006;60(1):62-68. doi:10.1038/sj.ejcn.1602268.
93. Overweight and Obesity Expert Panel. *Managing Overweight and Obesity in Adults: Systematic Evidence Review From the Obesity Expert Panel, 2013*.; 2013.
94. The Data Center. *Who Lives in New Orleans and Metro Parishes Now?*; 2017. https://www.datacenterresearch.org/data-resources/who-lives-in-new-orleans-now/.
95. Wetzels JFM, Kiemeney LALM, Swinkels DW, Willems HL, Heijer M Den. Age- and gender-specific reference values of estimated GFR in Caucasians: The Nijmegen Biomedical Study. *Kidney Int*. 2007;72(5):632-637. doi:10.1038/sj.ki.5002374.
96. Radin MS. Pitfalls in hemoglobin A1c measurement: When results may be misleading. *J Gen Intern Med*. 2014;29(2):388-394. doi:10.1007/s11606-013-2595-x.
97. Kim PS, Woods C, Georgoff P, et al. A1C underestimates glycemia in HIV infection. *Diabetes Care*. 2009;32(9):1591-1593. doi:10.2337/dc09-0177.
98. Qaseem A, Wilt TJ, Kansagara D, Horwitch C, Barry MJ, Forciea MA. Hemoglobin A1c Targets for Glycemic Control With Pharmacologic Therapy for Nonpregnant Adults With Type 2 Diabetes Mellitus: A Guidance Statement Update From the American College of Physicians. *Ann Intern Med*. 2018;168(8):569-576. doi:10.7326/M17-0939.
99. Sumithran P, Prendergast LA, Delbridge E, et al. Ketosis and appetite-mediating nutrients and hormones after weight loss. *Eur J Clin Nutr*. 2013;67(7):759-764. doi:10.1038/ejcn.2013.90.
100. Gardner CD, Trepanowski JF, Gobbo LCD, et al. Effect of low-fat VS low-carbohydrate diet on 12-month weight loss in overweight adults and the association with genotype pattern or insulin secretion the DIETFITS randomized clinical trial. *JAMA - J Am Med Assoc*. 2018;319(7):667-679. doi:10.1001/jama.2018.0245.
101. Brenner BM, Meyer TW, Hostetter TH. Dietary protein intake and the progressive nature of kidney disease: the role of hemodynamically mediated glomerular injury in the pathogenesis of progressive glomerular sclerosis in aging, renal ablation, and intrinsic renal disease. *N Engl J Med*. 1982;307(11):652-659. doi:10.1056/NEJM198209093071104.
102. Sato J, Kanazawa A, Makita S, et al. A randomized controlled trial of 130 g/day low-carbohydrate diet in type 2 diabetes with poor glycemic control. *Clin Nutr*. 2017;36(4):992-1000. doi:10.1016/j.clnu.2016.07.003.
103. Yancy WS, Foy M, Chalecki AM, et al. A low-carbohydrate, ketogenic diet to treat type 2 diabetes. *Nutr Metab (Lond)*. 2005;2(1):34. doi:10.1186/1743-7075-2-34.
104. Boden G, Sargrad K, Homko C, Mozzoli M, Stein TP. Effect of a low-carbohydrate diet on appetite, blood glucose levels, and insulin resistance in obese patients with type 2 diabetes. *Ann Intern Med*. 2005;142(6). doi:10.1016/S0084-3741(08)70336-6.
105. Stern L, Iqbal N, Seshadri P. The Effects of Low-Carbohydrate versus Conventional Weight Loss Diets in Severely Obese Adults : One-Year Follow-up of a randomized control trial. *Ann Intern …*. 2004;140(May 2001):769-777.

doi:10.1016/j.accreview.2004.07.103.
106. Westman EC, Yancy WS, Edman JS, Tomlin KF, Perkins CE. Effect of 6-month adherence to a very low carbohydrate diet program. *Am J Med*. 2002;113(1):30-36. doi:10.1016/S0002-9343(02)01129-4.
107. Moosheer SM, Waldschütz W, Itariu BK, Brath H, Stulnig TM. A protein-enriched low glycemic index diet with omega-3 polyunsaturated fatty acid supplementation exerts beneficial effects on metabolic control in type 2 diabetes. *Prim Care Diabetes*. 2014;8(4):308-314. doi:10.1016/j.pcd.2014.02.004.
108. Aagaard K, Petrosino J, Keitel W, et al. The Human Microbiome Project strategy for comprehensive sampling of the human microbiome and why it matters. *FASEB J*. 2013;27(3):1012-1022. doi:10.1096/fj.12-220806.
109. McInnes P, Cutting M. Manual of Procedures for Human Microbiome Project. http://www.hmpdacc.org/doc/HMP_MOP_Version12_0_072910.pdf.
110. Marrero DG, Palmer KNB, Phillips EO, Miller-Kovach K, Foster GD, Saha CK. Comparison of commercial and self-initiated weight loss programs in people with prediabetes: A randomized control trial. *Am J Public Health*. 2016;106(5):949-956. doi:10.2105/AJPH.2015.303035.
111. Barengolts E, Manickam B, Eisenberg Y, Akbar A, Kukreja S, Ciubotaru I. EFFECT OF HIGH-DOSE VITAMIN D REPLETION ON GLYCEMIC CONTROL IN AFRICAN-AMERICAN MALES WITH PREDIABETES AND HYPOVITAMINOSIS D. *Endocr Pract*. 2015. doi:10.4158/EP14548.OR.
112. Block G, Azar KMJ, Romanelli RJ, et al. Diabetes prevention and weight loss with a fully automated behavioral intervention by email, web, and mobile phone: A randomized controlled trial among persons with prediabetes. *J Med Internet Res*. 2015. doi:10.2196/jmir.4897.
113. Kramer MK, Molenaar DM, Arena VC, et al. Improving employee health: Evaluation of a worksite lifestyle change program to decrease risk factors for diabetes and cardiovascular disease. *J Occup Environ Med*. 2015. doi:10.1097/JOM.0000000000000350.
114. Parker AR, Byham-Gray L, Denmark R, Winkle PJ. The Effect of Medical Nutrition Therapy by a Registered Dietitian Nutritionist in Patients with Prediabetes Participating in a Randomized Controlled Clinical Research Trial. *J Acad Nutr Diet*. 2014;114(11):1739-1748. doi:10.1016/j.jand.2014.07.020.
115. Maruthur NM, Ma Y, Delahanty LM, et al. Early response to preventive strategies in the diabetes prevention program. *J Gen Intern Med*. 2013;28(12):1629-1636. doi:10.1007/s11606-013-2548-4.
116. Leong A, Daya N, Porneala B, et al. Prediction of Type 2 Diabetes by Hemoglobin A1c in Two Community-Based Cohorts. *Diabetes Care*. 2017;41(1):dc170607. doi:10.2337/dc17-0607.
117. Khaw K-T, Wareham N, Bingham S, Luben R, Welch A, Day N. Association of hemoglobin A1c with cardiovascular disease and mortality in adults: the European prospective investigation into cancer in Norfolk. *Ann Intern Med*. 2004;141(6):413-420. doi:10.7326/0003-4819-141-6-200409210-00006.
118. Cole RE, Boyer KM, Spanbauer SM, Sprague D, Bingham M. Effectiveness of Prediabetes Nutrition Shared Medical Appointments. *Diabetes Educ*. 2013;39(3):344-353. doi:10.1177/0145721713484812.

Low-Carbohydrate Dietary Pattern on Glycemic Outcomes Trial

PROTOCOL

PROTOCOL VERSION 2.1

Dated: June 26, 2020

New Orleans, LA

Table of Contents

1. Overview 5
2. Background and Significance 6
2.A. Type 2 Diabetes Mellitus is a major global public health challenge 6
2.B. Prediabetes is a major risk factor for cardiometabolic diseases 6
2.C. Role of lifestyle in preventing diabetes 6
2.D. Low-to-moderate carbohydrate diets, weight loss, and cardiometabolic diseases 7
2.E. Mediterranean-style diet is associated with reduced risk of T2D and improved glycemic control 8
2.F. Evidence for low-carbohydrate diets that emphasize Mediterranean-style dietary components 8
2.G. Evidence for specific food groups and risk of T2D 8
2.H. Gut microbiota, diet and T2D 9
2.I. Continuous Glucose Monitoring 9
2.I. Clinical and public health significance of the proposed study 9
2.J. Innovation 10
3. Preliminary Studies 10
3.A. Summary of aggregate experience of the research team 10
3.B. Low-carbohydrate behavioral dietary intervention trial experience 11
3.C. Other dietary intervention trial experience 11
4. Study Design and Organization 12
4.A. Overview of trial design 12
4.B. Study outcomes 13
4.B.1. Primary outcome 13
4.B.2. Secondary outcomes 13
4.C Study Timeline 13
6. Study Participants 13
6.A. Eligibility criteria 13
7. Recruitment and Randomization 14
7.A. Recruitment 14
7.A.1. Mass mailing, placement of brochures/flyers in community, TV/radio/billboard advertisements 15
7.A.2. Database searches and targeted mailing 15
7.A.3. Referrals and Local Partnerships 15
7.B. Randomization 15

7.C. Masking 15
7.D. Safety Considerations 16
8. Dietary Intervention Program 16
8.A. Low-carbohydrate dietary intervention group 16
8.B. Usual Diet Group 17
8.C. Physical Activity 17
8.D. Dietary Adherence 17
9. Safety of Dietary Intervention 18
10. Data Collection 18
10.A. Study visits 18
10.A.1. Screening/baseline visits 19
10.A.2. Follow-up visits and final visit 20
10.A.3. CGM 6-month visit 20
10.B Study measurements 21
10.B.1. Questionnaires 21
10.B.2. Dietary measurements 21
10.B.3. Assessment and management of adverse effects 21
10.B.4. BP and anthropometric measures 21
10.B.5. Blood samples 21
10.B.6. Urine collection 22
10.B.7. Stool Specimen Collection 22
10.B.8. CGM at 6 months: 22
11. Quality Assurance and Quality Control 23
11.A. Manual of procedures 23
11.B. Training and certification 23
12. Sample Size and Statistical Power 23
13. Data Management and Analysis 24
13.A. Data management 24
13.B. Data analysis plan 24
13.B.1. Exploratory data analysis 24
13.B.2. Primary, secondary, and exploratory outcomes 24
15. Anticipated Challenges 25
16. Protection of Human Subjects 25

16.A. Protection of human subjects from research risks 25
16.A.1. Risks to the subjects 25
16.A.2. Adequacy of protection against risks 27
16.A.3. Potential benefits of the proposed research to human subjects and others... 28
16.A.4. Importance of knowledge to be gained 28
16.A.5. Remuneration 28
16.B. Data safety and monitoring plan 29
Bibliography and References Cited 31

1. Overview

The increasing prevalence of type 2 diabetes mellitus (T2D), a major cause of morbidity and mortality worldwide, is a critical public health concern. In the United States, an estimated 33.9% of adults have prediabetes (impaired fasting glucose, elevated hemoglobin A1c (HbA1c), or impaired glucose tolerance). Individuals with prediabetes are at elevated risk of T2D and cardiovascular disease (CVD), with 5–10% developing clinical T2D each year.

In the short-term, among patients with T2D, low-to-moderate carbohydrate diets (energy percentage below 45) have a greater glucose-lowering effect than do high-carbohydrate diets, with larger glucose-lowering effect the greater the carbohydrate reduction. Additionally, compared with low-fat diets, Mediterranean diets that are high in unsaturated fats (particularly *cis*-monounsaturated fats) reduce risk of T2D and CVD and improve glycemic control among diabetics. However, compared with usual diets, the effect of a behavioral intervention promoting a low-carbohydrate/high-unsaturated fat and protein dietary pattern that emphasizes consumption of non-starchy vegetables, plant-based oils, fish, poultry, and mixed nuts on glucose homeostasis, and other metabolic risk factors among individuals with prediabetes or early-stage untreated T2D is not well understood.

Our *long-term goal* is to develop and implement dietary intervention(s) that reduce risk of T2D. The *overall goal of this randomized controlled trial is to study the effect of a behavioral intervention promoting a low-carbohydrate/high-unsaturated fat and high-protein dietary pattern (initial target <40 g digestible carbohydrates, final target <60 g digestible carbohydrates) compared with a usual diet on metabolic risk factors among individuals with prediabetes or early-stage untreated diabetes* (HbA1c 6.0–6.9%). Specifically, we will test the following hypotheses:

Primary hypothesis: Compared with usual diet, over a 6-month period, a healthy low-carbohydrate/high unsaturated fat and high-protein dietary pattern will lead to *a larger decrease of HbA1c, by at least 0.15%.*

Secondary hypothesis: Over a 6-month period, a healthy low-carbohydrate/high-unsaturated fat and high-protein dietary pattern will lead to *larger improvements in the following major secondary outcomes*: fasting glucose, systolic blood pressure (BP), total-to-HDL-cholesterol ratio, and body weight.

Exploratory outcomes: We will also study the effect of the intervention on the 6-month change in: insulin and homeostasis model assessment of insulin resistance (HOMA-IR); diastolic BP; waist circumference; and estimated CVD risk.

To achieve these study aims, we will recruit 126 adults with prediabetes or untreated diabetes (HbA1c 6.0–6.9%) in the Greater New Orleans, LA area. Using a stratified, blocked randomization scheme, participants will be assigned to either a behavioral modification intervention aimed at promoting a healthy low-carbohydrate dietary pattern or to usual diet. The intervention group will have an intensive phase for 3 months, followed by a maintenance phase in months 4 through 6. The *primary outcome will be difference between the active intervention and control groups in the change in HbA1c from baseline to 6 months of follow-up. We expect 80% power to detect a difference of at least 0.15% in change of HbA1c.* Changes in other metabolic risk factors will be studied concurrently. Metabolic risk factors will be assessed at baseline, 3 months, and 6 months of follow-up.

There is much interest regarding dietary approaches to prevent T2D and its complications. The results from this study will help to determine whether, compared with usual diet, a behavioral intervention aimed at promoting a healthy low-carbohydrate dietary pattern improves metabolic risk factors.

2. Background and Significance

2.A. Type 2 Diabetes Mellitus is a major global public health challenge

Diabetes is a strong and independent risk factor for CVD morbidity and mortality. A meta-analysis of 102 prospective studies (698,782 participants) estimated diabetes leads to an approximately 2-fold increased risk in vascular diseases, including coronary heart disease (CHD) and major stroke subtypes, and deaths due to other vascular causes.[1] Among participants with diabetes, CHD risk is about 50% higher among those with fasting blood glucose ≥7 mmol/L compared with those with fasting blood glucose <7 mmol/L.[1] In addition to increasing CVD risk, diabetes can cause diabetic retinopathy,[2] chronic kidney disease,[3] lower limb amputation,[4] and other complications. Most individuals with diabetes have T2D.[5] The US Centers for Disease Control and Prevention (CDC) estimated that 30.2 million adults ≥18 years (12.2%) had diabetes in 2015, with 7.2 million (23.8%) not being aware of or reporting diabetes.[5] Diagnosed diabetes prevalence among US adults has increased substantially over time, from 2.54% in 1980 to 7.40% in 2015.[6] Diabetes prevalence is also high globally, with an estimated 2014 age-standardized prevalence of 9.0% in men and 7.9% in women.[7] Diabetes accounts for an increasing portion of US healthcare expenditures. Total cost of diagnosed diabetes in the US was estimated to be $245 billion in 2012, 41% higher than in 2007.[8] Diabetes is associated with much higher lifetime medical expenses, with higher expenses for those diagnosed at younger ages.[9]

2.B. Prediabetes is a major risk factor for cardiometabolic diseases

Prediabetes, defined by the American Diabetes Association (ADA) as impaired fasting glucose (IFG), impaired glucose tolerance (IGT), or elevated HbA1c, is also referred to as “intermediate hyperglycemia”[10] and “high-risk state of developing diabetes”.[11] The ADA defines prediabetes as having: fasting plasma glucose of 100–125 mg/dL (IFG); 2-hour post-load glucose in the 75-g oral glucose tolerance test (OGTT) of 140–199 mg/dL (IGT); or HbA1c 5.7–6.4%.[12] Prediabetes itself is not a disease; for all three tests, “risk is continuous, extending below the lower limit of the range and becoming disproportionately greater at the higher end of the range”.[12] The CDC estimated that in 2015, 33.9% of US adults had prediabetes based on fasting glucose or HbA1c.[5] Among those with prediabetes, only 11.9% reported having being told this by a medical professional.[5] Approximately 5–10% of individuals with prediabetes develop diabetes within one year, depending on how prediabetes is defined.[13] In a meta-analysis, annual diabetes incidence was 15–19% among individuals with both IFG and IGT, while it was 4–6% in those with isolated IFG and 6–9% in those with isolated IGT.[13]

A recent meta-analysis (1,611,339 participants from 53 studies) reported that prediabetes as defined by the ADA is associated with higher risk of composite CVD events and CHD.[14] IFG and IGT were also positively associated with increased risk of stroke and all-cause mortality.[14] Prediabetes is linked with increased risk of other conditions typically thought of as diabetes complications, such as neuropathies and nephropathies.[15] Importantly, it is possible for individuals with prediabetes to revert to normoglycemia, as observed in observational studies[13] and lifestyle or drug intervention trials.[16,17] In the Diabetes Prevention Program (DPP), reversion to normoglycemia was associated with lower long-term T2D risk[18] and lower predicted CVD risk.[19]

2.C. Role of lifestyle in preventing diabetes

Much evidence from observational studies and clinical trials supports the critical role of diet and other lifestyle factors in preventing T2D. Globally, lifestyle transitions towards reduced physical activity, increased consumption of refined carbohydrates, coupled with resulting changes in body composition, has led to increases in diabetes prevalence and severity.[20] Body

mass index (BMI), waist circumference, and waist-to-height ratio are predictors of T2D.[21] The DPP, which combined calorie restriction and exercise to promote weight loss, substantially reduced risk of T2D (by 58%) among individuals with elevated FPG and IGT;[22] a benefit that persisted in 10-year follow-up analyses.[16] Other trials of lifestyle modification focused on diet and exercise have also shown benefits in study populations in China,[23] Finland,[24] and India.[25]

2.D. Low-to-moderate carbohydrate diets, weight loss, and cardiometabolic diseases

Low-to-moderate carbohydrate diets (<45% of energy from carbohydrates) are popular dietary approaches for losing weight. Weight loss is a key component of the DPP lifestyle intervention[26] and ADA lifestyle recommendations.[12,27] Meta-analyses from our group[28] and others support that low-to-moderate carbohydrate diets are at least as effective as low-fat diets at promoting weight loss. For instance, a meta-analysis reported a 2-kg (95% CI, 3.1–0.9) larger weight loss in low-carbohydrate diet (<120 g carbohydrates/day) arms compared with low-fat diet (<30% energy from fat) arms.[29] Estimated 10-year CVD risk was lower in low-carbohydrate than low-fat arms.[29] This aligns with research published by our team; in a 12-month trial of participants with obesity, a low-carbohydrate diet (target <40 g carbohydrates/day) was more effective than a low-fat diet (target <30% energy from fat, <7% saturated fat) for weight loss and CVD risk factor reduction.[30]

Low-to-moderate carbohydrate diets may also be beneficial among patients with T2D. Low-carbohydrate diets have come in and out of favor as treatment of diabetes.[31] In a meta-analysis (1376 patients;10 trials), after 3 or 6 months of the intervention, low-to-moderate carbohydrate diets (<45% energy from carbohydrates) led to a 0.34% lower HbA1c (95% CI, 0.06–0.63%) compared with high-carbohydrate diets.[32] Compared with high-carbohydrate diets, there was a greater reduction in HbA1c the greater the reported carbohydrate restriction.[32] There was no benefit of the low-to-moderate carbohydrate diets on HbA1c at 12 months or longer, and no difference between interventions on BMI, body weight, low-density lipoprotein (LDL) cholesterol or quality of life.[32] However, results may have been affected by dietary adherence or glucose medication. There was evidence of diabetes medication reduction in the low-carbohydrate arms in some studies,[33–37] with evidence of medication reduction and improved blood glucose stability persisting for up to 2 years.[38] Hypoglycemia (though not after medication adjustment) was observed in three subjects taking insulin or a sulfonylurea out of twelve subjects in the low-carbohydrate arm of another study.[39] In a subset of participants with T2D (n=46) from a large weight loss trial, compared with those randomized to a low-fat diet and orlistat, those randomized to a low-carbohydrate diet for 48 weeks had improved HbA1c (0.8% lower, 95% CI, -1.5, -0.02%) and a reduction in antiglycemic medications, despite similar weight loss effects of the interventions.[40]

Though low-to-moderate carbohydrate diets have been studied among overweight and obese participants (primarily focused on weight loss), less is understood about the effects of such diets on glycemic outcomes specifically among individuals with prediabetes. A study among participants with IGT observed a reduction at 12 months in weight, HbA1c, and other glycemic outcomes among individuals who participated in a 7-day in-hospital education program, though this was not randomized.[41] A randomized pilot trial of overweight or obese individuals with T2D or prediabetes found that, over 3 months and at 12 months, a very low-carbohydrate diet improved HbA1c, led to higher medication discontinuation, and more weight loss compared with a low-fat diet.[34,37] However, only 4 individuals had prediabetes. Given benefits of low-to-moderate carbohydrate diets for weight loss in general populations and for glycemic control among patients with T2D, further study of these diets are warranted among individuals with prediabetes who are at elevated cardiometabolic disease risk.

2.E. Mediterranean-style diet is associated with reduced risk of T2D and improved glycemic control

Though there is no one “Mediterranean diet”, the Mediterranean dietary pattern tends to have the following characteristics: high consumption of vegetables, fruits, breads and cereals, legumes, nuts and seeds; low-to-moderate consumption of dairy products, fish, poultry and eggs; little consumption of red meat and sweets; and red wine in moderation.[42] Olive oil is an important source of fat. In many observational studies, adherence to the Mediterranean-style dietary pattern is associated with reduced risk of CVD[43] and T2D.[44,45] Evidence from randomized trials also supports the benefits of this dietary pattern. In several trials, participants randomized to Mediterranean diets had substantially lower risk of CVD than those randomized to low-fat control diets.[46,47] In the three-arm randomized PREDIMED trial of participants at high risk of CVD, after 4.1 years of follow-up, those randomized to a traditional Mediterranean diet supplemented with either olive oil or nuts had a 30% reduced risk of T2D compared to those in the low-fat control diet group.[48] In all PREDIMED arms, average carbohydrate intake was relatively low (39.7–43.7% at end of trial).[47] Two trials have found a higher rate of remission from metabolic syndrome among individuals randomized to a Mediterranean diet compared with those randomized to control diets (prudent or low-fat) during 2–5 years of follow-up.[49,50] The Mediterranean pattern may also be beneficial among individuals with T2D. Meta-analyses have reported that, among patients with T2D, those randomized to a Mediterranean diet had lower HbA1c levels and CVD risk factors, and more significant weight loss, compared with those randomized to control diets.[51]

2.F. Evidence for low-carbohydrate diets that emphasize Mediterranean-style dietary components

Many low-carbohydrate diets involve increased consumption of food items, such as red meat,[52] that are associated with increased cardiometabolic disease risk. However, low-carbohydrate dietary approaches can focus on substituting carbohydrates with foods common in Mediterranean-style diets, such as olive oil and nuts, which may improve cardiometabolic health. In a 4-year Italian study of individuals with newly diagnosed T2D, those randomized to a low-carbohydrate Mediterranean diet (<50% carbohydrates; consumed ~44%) had more favorable changes in glycemic control, higher rates of T2D remission, and delayed need for antiglycemic drugs compared with participants randomized to a low-fat diet (<30% fat, <10% saturated fat).[53,54] A 12-month Israeli study assessed low-carbohydrate Mediterranean (35% carbohydrates), traditional Mediterranean, and ADA (both 50–55% carbohydrates) diets in 259 overweight participants with T2D.[55] Most CVD risk factors improved in all groups; there was only HDL improvement in the low-carbohydrate Mediterranean diet and this diet was superior to the other diets in improving glycemic control.[55] In a 2-year weight-loss study of moderately obese participants, low-carbohydrate and Mediterranean diets were superior to low-fat diet for weight loss.[56] Participants in the low-carbohydrate arm were counseled to choose vegetarian sources of fat and protein and to avoid trans fat. At 6 years after study commencement, despite weight regain, there were still long-term benefits, particularly for the low-carbohydrate and Mediterranean diets.[57] In whole, evidence supports that low-carbohydrate Mediterranean-style diets are feasible and may have metabolic benefits.

2.G. Evidence for specific food groups and risk of T2D

Prospective cohort studies have found consumption of specific foods and beverages is associated with diabetes risk. Meta-analyses report higher consumption of processed and unprocessed red meat,[58] white rice (particularly in Asian populations),[59] and sugar-sweetened beverages[60,61] are associated with higher T2D risk. Consumption of green leafy vegetables,[62,63] yogurt,[64] whole grains,[65] decaffeinated and total coffee,[66] and light-to-moderate alcohol

consumption[67] are associated with reduced risk. Consumption of fruits, particularly blueberries, grapes, and apples, are associated with reduced risk; fruit juice is associated with higher risk.[68]

2.H. Gut microbiota, diet and T2D

There is increasing evidence for the importance of gut microbiota in risk of type 2 diabetes and other cardiometabolic diseases.[69] Additionally, dietary modifications, including changes in carbohydrate consumption, can lead to substantial changes in gut microbiome over short periods of time.[70,71] Less is known about longer-term changes to the gut microbiota during the adoption of a low-carbohydrate diet and how these changes may influence cardiometabolic risk. We plan to collect stool specimens at baseline, 3 months, and at 6 months, for potential future studies on how this intervention affects the gut microbiome.

2.I. Continuous Glucose Monitoring

As HbA1c is a marker of weighted average glucose over the past 8 to 12 weeks, it does not explicitly capture short-term glycemic excursions or variability. It has been theorized that such fluctuations may have an independent role in the development of diabetes complications.[3] Though more evidence is needed, research has found relationships of higher glycemic variability with coronary artery disease (CAD) and microvascular outcomes in type 2 diabetes (T2D).[4,5] This suggests that improved control of extreme glucose excursions, particularly postprandially, may help to reduce risks of such complications.[5]

In one study of a low-calorie, low-carbohydrate diet compared with a low-calorie, low-fat behavioral dietary intervention among individuals with T2D, there was evidence that some markers of glycemic variability were further reduced in the low-carbohydrate arm compared with the low-fat arm (assessed by 48-hour continuous glucose monitoring) at 6 months, 1 year, and 2 years.[6–8] However, there is not prior work assessing how a behavioral intervention aimed at reducing carbohydrate consumption affects mean glucose, glucose exposure above the set-point of 100 mg/dL, glucose excursions, or glycemic variability as measured by continuous glucose monitoring among individuals with untreated T2D or at high risk of T2D.

Given this background, we will use continuous glucose monitors (CGM) to collect data for up to 14 days on glucose levels at the end of the 6-months study and will compare mean 24-h glucose among those in the intervention group compared with mean 24-h glucose among those in the control group. We will also measure other markers of glycemic exposure, such as glycemic variability (mean amplitude of glycemic excursions (MAGE), standard deviation of glucose, coefficient of variation) and mean 24-hour glucose during wake-time (6:00 AM to 12:00 AM), and during sleep-time (12:00 AM to 6:00 AM).

2.I. Clinical and public health significance of the proposed study

Prediabetes, or intermediate hyperglycemia, is a marker of high risk of developing T2D and other complications. Due to increasing prevalence of T2D and associated morbidity and mortality globally, a better understanding of dietary approaches to delay progression to T2D or to promote reversion to normoglycemia is a high public health priority. This study proposes to assess the effect of a behavioral intervention promoting a low-carbohydrate/high-unsaturated fat dietary pattern (initial target <40 g digestible carbohydrates, final target <60 g digestible carbohydrates) on HbA1c, a marker of glycemic control, and other metabolic risk factors. Building from prior findings from clinical and observational studies, we will develop a dietary intervention that promotes the consumption of non-starchy vegetables, plant-based oils, fish, poultry, and mixed nuts, that are thought to reduced risk of T2D or CVD. Participants assigned

to the intervention will be trained in behavioral techniques that will provide capacity to maintain dietary changes long after study completion. Our outcome measures will assess the effect of this dietary intervention in a “real-life” setting as opposed to dietary modifications achieved in metabolic ward studies or through food prepared in research kitchens. Findings from this study may provide evidence in support of promoting alternative dietary styles among individuals with high T2D and CVD risk and could potentially be used to inform dietary interventions used in programs like DPP.

2.J. Innovation

The status quo as it pertains to dietary approaches for reducing risk of T2D is focused on reducing caloric intake and total fat intake. However, recent evidence suggests that the types of fat, rather than the quantity of fats may be more important, with evidence supporting that a Mediterranean-style diet that does not restrict fat intake reduces risk of developing both T2D[48] and CVD[47]. Moreover, in the short-term, low-carbohydrate diets, which are popular weight loss diets, have a greater glucose-lowering effect than do high-carbohydrate diets among patients with T2D.[32] However, the effect of low-carbohydrate diets that emphasize consumption of non-starchy vegetables, plant-based oils, fish, poultry, and mixed nuts on T2D prevention is unclear, particularly compared with higher-carbohydrate diets more commonly consumed in the US. *The proposed research is innovative, in our opinion, because it represents a substantial departure from the status quo by assessing the combined effect of a low-carbohydrate, high unsaturated fat and protein diet on cardiometabolic risk factors among adults with prediabetes or early-stage untreated diabetes.* We expect the results from this study have the potential to lead to new horizons for developing and implementing dietary approaches (other than the most commonly used reduced fat diet) that will substantially reduce risk of cardiometabolic disease among adults with T2D or at high risk of T2D. The proposed intervention dietary approach promotes consumption of dietary components thought to reduce risk of cardiometabolic disease and the behavior change intervention has expected applicability in clinical practice.

3. Preliminary Studies

3.A. Summary of aggregate experience of the research team

Dr. Kirsten Dorans is trained in cardiovascular disease epidemiology, with strong training in epidemiologic methods and biostatistics. Her publication record includes studies of behavioral risk factors and risk of chronic disease. She has published on the relationships between dietary intake and chronic heart failure[72,73] and on physical activity and incident multiple sclerosis[74]. She served as lead author, conducting data management, analysis, drafting, and revision of two of these manuscripts. In graduate school, she focused on studying links between outdoor air pollution and chronic diseases and has published several papers in this field. The COBRE program will provide an excellent opportunity for the JFI to transition from air pollution research to clinical and translational research focused on nutrition and diabetes. Building off of her training in observational epidemiology, this program will involve training and practical experience that will allow her to broaden her expertise and to develop practical skills for carrying out clinical research.

The mentorship team has much experience and prior successful collaborations in key areas related to this study. Dr. Jiang He is an internationally renowned expert in cardiometabolic disease clinical and epidemiological research. He has served as PI for many epidemiological studies and clinical trials, including clinical and observational studies focused on dietary consumption and chronic diseases[75–80] and has conducted extensive global research on diabetes.[81,82] Dr. Bazzano has collaborated extensively with Dr. He for more than 15 years in research related to diet and chronic disease. She is a clinical investigator and has expertise in

nutritional epidemiology and clinical research. Dr. Bazzano was the PI of a highly successful clinical trial comparing the effects of low-carbohydrate and low-fat diets on weight and cardiovascular risk factors among individuals with obesity, which was a subproject within the Tulane COBRE in Hypertension and Renal Biology.[30] Dr. He served as Dr. Bazzano's mentor for this clinical trial. They have collaborated on much other work related to dietary and chronic disease, including a meta-analysis on low-carbohydrate diets.[28] Dr. Bazzano has also published many articles on diet and risk of diabetes[83,84] and on diabetes in the Bogalusa Heart Study.[85–87]

3.B. Low-carbohydrate behavioral dietary intervention trial experience

In the study on macronutrient composition of diet and risk factors for cardiovascular disease (MACRO) study, 148 individuals without clinical cardiovascular disease or diabetes and with obesity were randomly assigned to a low-carbohydrate or a low-fat behavioral dietary intervention for 12 months. In the low-carbohydrate arm, daily dietary intake of carbohydrates decreased from an average of 242 g at baseline to 97 g, 93 g, and 127 g at 3, 6, and 12 months, respectively. At 12 months, participants in the low-carbohydrate intervention group had greater decreases in weight (-3.5 kg; 95% CI, -5.6 to -1.4 kg) and fat mass (-1.5%; 95% CI, -2.6% to -0.4%) and greater improvement in several cardiovascular risk factors.[30] This suggests that restricting carbohydrates may be an option for weight loss and reduction of cardiovascular risk factors. Further analyses found that the low-carbohydrate dietary intervention led to similar or greater improvement in inflammation, adipocyte dysfunction, and endothelial dysfunction compared with the low-fat dietary intervention[88] and that the low-fat diet reduced peptide YY more than the low-carbohydrate diet, suggesting satiety may be better preserved on low-carbohydrate diets.[89] No serious adverse events were reported in this study and the number of participants with symptoms were similar between the two diets, with the exception of more participants having headaches on the low-fat diet at 3 months (18 vs 6 participants, $p = 0.03$).[30] Dr. Bazzano served as PI for this study and Dr. He served as her mentor. Dr. Bazzano mentored one of her PhD students in conducting analyses and writing and editing manuscripts related to this project.

3.C. Other dietary intervention trial experience

Dr. He has also led or been involved with many dietary intervention trials, including both behavioral change and feeding study interventions. In the Protein and Blood Pressure (ProBP) study, 352 adults with prehypertension or stage 1 hypertension in New Orleans, LA and Jackson, MS were assigned to take, in a random order, 40 g/d soy protein, milk protein, or carbohydrate supplementation each for 8 weeks. Compared with the high-glycemic index refined carbohydrate, both soy protein and milk protein supplementation were associated with a -2.0 mm Hg (95% CI -3.2 to -0.7 mm Hg) and -2.3 mm Hg (-3.7 to -1.0 mm Hg) net change in systolic blood pressure.[80] These findings suggest that partial replacement of carbohydrate with soy or milk protein might be an important part of nutrition intervention strategies to prevent and treat hypertension. Additionally, soy protein supplementation led to reduction in plasma E-selectin compared with carbohydrate and milk protein and in plasma leptin compared with carbohydrate.[90] Soy protein also led to improvement in lipid profile compared with carbohydrate and milk protein.[91] Dr. He was PI for this project.

In the Fiber Intervention on Blood Pressure (FIBRE) study, 110 participants ages 30–65 years with untreated pre-hypertension or stage-1 hypertension and serum cholesterol level < 240 mg/dl were randomly assigned to 8-g/day of water soluble fiber from oat bran or a control intervention (double-blind RCT). Over the 12-week study period, net changes in systolic and diastolic blood pressure were -1.8 mm Hg (-4.3 to 0.8) and -1.2 mm Hg (-3.0 to 0.5 mm Hg), respectively.[78] Net changes in total, HDL-, and LDL-cholesterol were -2.40 mg/dL (-10.60 to

5.81 mg/d:), -1.66 mg/dL (-4.55 to 1.22 mg/dL), and -1.33 mg/dl (-8.33 to 5.68 mg/dL), respectively.[92] Dr He was PI for this study.

Dr. He is currently serving as a mentor for Dr. Katherine Mills' COBRE project (Effect of Dietary Sodium Reduction in Kidney Disease Patients with Albuminuria; SUPER). In this project, participants with chronic kidney disease and albuminuria are being randomized to a behavioral modification program designed to reduce dietary sodium intake to ≤2,300 mg/day or to usual care and test the effect of this intervention on change in albumin-to-creatinine ratio from baseline to 24-weeks.

4. Study Design and Organization

4.A. Overview of trial design

We propose to conduct a *randomized controlled trial* aimed at testing the effect of a behavioral intervention that promotes *reduced carbohydrate intake and increased consumption of heart-healthy unsaturated fats and protein* in patients with HbA1c 6.0–6.9% (*Figure 1*). We plan to recruit 126 individuals with elevated HbA1c and randomly assign them to a moderately intensive intervention on diet pattern,[93] using well-established methods for behavioral modification, or to usual diet. We will compare the difference between the active intervention and control groups for change in HbA1c from baseline to 6 months of follow-up (primary outcome). We will assess four major secondary outcomes: fasting plasma glucose, systolic BP, total-to-HDL cholesterol ratio, and body weight. In addition, we will look at several other exploratory outcomes: insulin and HOMA-IR, diastolic BP, waist circumference, and estimated CVD risk, as estimated by the 2013 ACC/AHA risk equation. The main trial components are summarized in *Table 1*.

Figure 1. Study Design

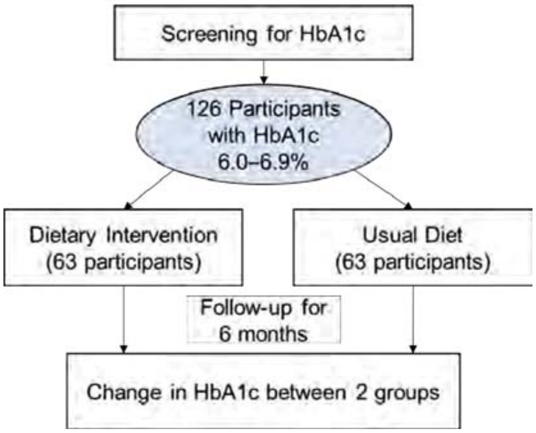

Table 1. Study Overview

Study Design	Randomized controlled trial
Study Participants	126 participants with elevated HbA1c (6.0–6.9%)
Intervention Group (n=63)	Behavioral modification to reduce carbohydrate consumption and promote consumption of foods such as non-starchy vegetables, plant-based unsaturated oils, nuts and seeds, avocados, eggs, seafood, poultry, lean meat and tofu. We will recommend moderate consumption of cheese, unsweetened Greek yogurt, and low-carb milk. If consuming higher-carbohydrate items, recommend legumes, fruits, (limited) whole grains. Target <40 g net carbohydrates per day for first 3 months and will have a goal of <40 net g carbohydrates per day; <60 g net carbohydrates per day for months 4 onwards.
Usual Diet Group (n=63)	No dietary intervention. To encourage continued participation in the trial, we will offer optional monthly educational sessions on unrelated topics, such as retirement planning or health insurance
Outcome Measures	Primary: HbA1c; Secondary: fasting plasma glucose, systolic BP, total-to-HDL-cholesterol ratio, and body weight

4.B. Study outcomes

4.B.1. Primary outcome: The *primary outcome will be the difference between the intervention group and the usual diet group for change in HbA1c* from baseline to 6 months. Changes in HbA1c will be calculated from blood samples taken at baseline and 6 months.
4.B.2. Secondary outcomes: Secondary outcomes will include changes from baseline to 6 months of *fasting plasma glucose, systolic BP, total-to-HDL cholesterol ratio, and body weight.,*
4.B.3. Other outcomes: We will also study the effect of the intervention on the following outcomes: insulin and homeostasis model assessment of insulin resistance (HOMA-IR); diastolic BP; waist circumference; total-to-HDL-cholesterol ratio; and estimated CVD risk.

4.C Study Timeline

Table 2 shows the timeline, which will be followed in the implementation of study-related activities, such as publications and submission of an R01 grant application.

Table 2. Study Timeline

	Year 1		Year 2		Year 3	
Develop MOP, intervention program, study forms, and pilot testing	X	X				
Certification and training of staff	X					
Recruitment		X	X			
Intervention and data collection, measurement, entry, cleaning		X	X	X	X	
Data quality control and analysis						X
Publications	X	X	X	X	X	X
R01 grant submissions, resubmissions				X	X	X

The first 6 months will focus on development of manual of operations (MOP), intervention program, study forms, study protocol, Institutional Review Board (IRB) approval, and pilot testing of the intervention program. It will also involve staff training and certification. During the last half of year 1, recruitment, intervention, and data collection will commence. During the last 3 months of year 3, data quality control and analysis will be carried out. Throughout the study period, the JFI will publish peer-reviewed manuscripts from ongoing Tulane University research projects.

6. Study Participants

6.A. Eligibility criteria

Inclusion criteria

- Men or women ages 40 to 70 years: aim to recruit 50% women
- Any race or ethnicity: aim to recruit 40% ethnic minorities (i.e., African American and Hispanic), close to population in the greater New Orleans area[94]
- HbA1c 6.0–6.9%
- Willing and able to provide informed consent

Exclusion criteria

- Diagnosed type 1 diabetes mellitus based on patient self-report
- Use of agents affecting glycemic control (medications for diabetes, oral glucocorticoids) within the past three months prior to enrollment based on patient self-report
- Medical condition in which low-carbohydrate diet may not be advised
    - Estimated glomerular filtration rate (eGFR) ≤45 mL/min/1.73 m^2, which is close to the 5th percentile of eGFR among non-diseased individuals of 70 years of age[95]

- Self-report of liver disease due to hepatitis or alcohol; osteoporosis; untreated thyroid disease; gout; or cancer (other than non-melanoma skin cancer) requiring treatment in the past year, unless prognosis is excellent

- Factors that may affect HbA1c:
  - Hemoglobin <11 mg/dL (cutpoint for moderate-to-severe anemia, which could lead to falsely elevated or lowered HbA1c)[96]
  - Recent blood donation or blood transfusion (self-report, past 4 months)
  - Human Immunodeficiency Virus (self-report)[97]
- Self-reported history of intensive care unit stay due to Coronavirus Disease 2019 (COVID-19) in the past three months, as severe COVID-19 may affect blood glucose levels
- Allergies to nuts
- For women, current pregnancy, breastfeeding, or plans to become pregnant during the study
- Consumption of ≥21 alcoholic drinks per week or consumption of ≥6 drinks per occasion
- For the CGM collection at the end of the study period only: known allergy to adhesives or other products involved with CGM use (e.g., skin disinfectants), current pregnancy, currently on hemodialysis or peritoneal dialysis, or people with other implanted medical devices (e.g., a pacemaker)
- Current or planned residence making it difficult to meet trial requirements (due to distance from study site and/or challenges regularly traveling to site)
- Current participation in another lifestyle intervention trial or a pharmaceutical trial
- Participation of another household member in the study; employees or persons living with employees of the study
- Other concerns regarding ability to meet trial requirements, at the discretion of the principal investigator or study coordinator

7. Recruitment and Randomization

7.A. Recruitment

We will recruit study participants from the greater New Orleans area. The initial focus of our recruitment will be mass mailing of individuals in the greater New Orleans area and recruitment through placing flyers and brochures in the community. If community-based recruitment is not successful or cost-effective, we will recruit out-patients from the Ochsner Health System and Tulane Medical Center (TMC), from which our group has prior experience recruiting patients. For this recruitment, we will focus on electronic searches of medical records and laboratory databases. We will also carry out targeted solicitation of referrals from physicians who care for patients likely to meet our inclusion and exclusion criteria.

In addition to this recruitment plan, we have several contingency plans: (1) extending recruitment to other medical centers in the New Orleans area, including the Veterans Affairs Medical Center (VAMC), East Jefferson General Hospital, and West Jefferson Medical Center, with whom Tulane has well-established relationships; (2) TV/radio advertisements; (3) arrange a news story about the study in local TV newscasts, radio talk programs, and/or print media; and (4) conduct outreach at local community events and churches. We have ample experience with each of these approaches.

Based on U.S. Census Bureau 2016 population estimates, the metro New Orleans area population was approximately 35% African American or black and 9% Hispanic.[94] Given our prior experience and population distribution in this region, we plan to recruit a distribution of study participants with 40% ethnic minorities.

7.A.1. Mass mailing, placement of brochures/flyers in community, TV/radio/billboard advertisements: Community recruitment approaches include: mass mailing individuals who live in the New Orleans area; brochures/flyers in the community; emailing the brochure; TV, radio, poster, or billboard advertisements in the New Orleans area. Interested individuals will be pre-screened; those at elevated risk for prediabetes or T2D, i.e. individuals who are obese or overweight and/or have a sufficiently high score from the American Diabetes Association's Diabetes Risk Assessment Questionnaire will be invited into the clinic for a screening visit.

7.A.2. Database searches and targeted mailing: We will request IRB approval for a waiver for HIPAA authorization to conduct database searching and hospital chart reviews for identification of potentially eligible study participants. After identifying a potentially eligible patient, we will send this individual an invitation letter signed by the investigators and/or the individual's personal physician and brochure via mail or email. Individuals who express interest in the study will have a pre-screening telephone interview for confirmation of potential eligibility. Those still interested and potentially eligible for enrollment will be scheduled for a visit at the COBRE study clinic.

7.A.3. Referrals and Local Partnerships: In addition to local physician referrals, we will explore holding outreach community events in the New Orleans area. Local community partners may post information about the study on their social media platforms. We may also identify potentially interested volunteers from a Tulane volunteer recruitment registry. We will also consider recruiting potentially eligible individuals who were screened or participated in other clinical studies in our clinic and have expressed interest in being contacted about future research projects. Potential participants referred by their physician or community partners will have a pre-screening telephone interview to confirm potential eligibility. Those who are still interested and potentially eligible for the study will be scheduled for a screening visit.

7.B. Randomization

Once eligibility has been confirmed, the study coordinator will ensure that all key variables have been entered into the trial-specific database. Additionally, the study coordinator will ensure any outliers have been recognized and reconciled, and will ensure receipt of informed consent prior to enrolling the volunteer in the study as a trial participant. Eligible participants will be *randomly assigned to the behavioral intervention or the usual diet group in a 1:1 allocation ratio*. Randomization will be stratified by sex, *using a random block group size (4, 6) to maintain balance among groups over time*. A statistician from the Methodology/Biostatistics Unit within the COBRE Clinical Research Core, who is not directly involved in the study and is not located at the clinic, will conduct the randomization. After ascertaining eligibility, clinical personnel will telephone the Methodology/Biostatistics Unit to obtain a randomization assignment. All randomization assignments will be created by a computer program and only accessible to the Staff at the Methodology/Biostatistics Unit. Once randomization assignment has been made, trial participants will be considered to be officially randomized into the study and every effort will be made to obtain complete follow-up information.

7.C. Masking

Clinical and laboratory staff members who assess outcomes will be blinded to the intervention assignment of participants. As this is a dietary intervention trial, study participants, and study staff members who conduct the intervention cannot be blinded to the intervention assignment. Participants will be instructed to avoid sharing knowledge of their treatment assignment with data collection staff members. We will have intervention visits and data collection study visits on different days and in separate locations. Data collectors will also use verbal cues and visual props to remind participants not to disclose treatment assignment. Investigators will be blinded to intervention assignment.

7.D. Safety Considerations

Participants with HbA1c >7.9% will be referred to a clinician for follow-up[98], though we will continue to collect data on these participants.

8. Dietary Intervention Program

8.A. Low-carbohydrate dietary intervention group

Participants randomized to the low-carbohydrate intervention group will receive counseling

aimed at reducing carbohydrate consumption. Phase I (*Go Low*) will occur for the first 3 months, with a goal of <40 g digestible carbohydrates per day. Key recommended foods will include: non-starchy vegetables, fish, poultry, lean meat, eggs, olive oil and other plant-based unsaturated oils, unsweetened/unsalted nuts and seeds, nut butters, and avocados. We will recommend moderate consumption of cheese and unsweetened Greek yogurt and low-carbohydrate milk. During Phase I, we will recommend limiting or avoiding other dairy, fruits, legumes, beans, and grains. Phase II (*Keep it Low*) will run from months 4 through 6. The net carbohydrate goal for this phase will be <40 g to <60 g, with the objective to choose a target that is as low as possible (e.g., as close to <40 g as possible). At 3 months, we expect different participants will have different levels of intake, with some consuming <40 g of net carbohydrates and others consuming levels higher than 60 g net carbohydrates per day. We have designed the intervention to accommodate various scenarios, with the goal of achieving as low of an intake as possible. For participants who consume >60 g per day, we will recommend they aim to either reduce or maintain carbohydrate intake.

At a brief check-in at 3-months, if participants are satisfied with their current intake, we will instruct them to stay at this intake level (though will recommend they try to reduce intake, if possible, if they are eating >60 net grams of carbohydrates or have not yet reached the study goal of <40 net grams of carbohydrates). If they prefer to make modifications, we will provide guidance on how to modify carbohydrate intake (either decrease or increase). If they choose to add back carbohydrates, we will recommend that participants add 5 grams of carbohydrates for two weeks at a time (e.g., from 40 to 45 g for 2 weeks), for a maximum total of <60 g net carbohydrates. If they choose to further reduce carbohydrate intake, we will provide guidance on this as well. As they modify carbohydrate intake, we will have them monitor their satisfaction with their diet and energy levels. If adding back carbohydrates, we will recommend participants add back in foods like fruit (preferably berries), legumes, beans, and dairy, while still aiming to keep total carbohydrate intake as low as possible (e.g., as close to <40 net grams as able).

Materials: We will develop a handbook that will be given to participants. This will contain diet guidelines, recipes, meal planners, shopping lists, and guides for counting carbohydrates and reading nutrition labels.

Meals: Participants will select their own menus and prepare or buy their meals according to the guidelines. We will provide participants with supplemental low-carbohydrate foods, such as nuts, olive oil, and other products, to help facilitate their compliance.

Counseling: Sessions and materials will focus on instructing participants to reduce digestible carbohydrate consumption by increasing consumption of protein and fat, with a particular emphasis on increased consumption of heart-healthy unsaturated fats. After randomization, participants will begin the 3-month *Go Low* phase consisting of individual counseling sessions each week for the first four weeks, followed by 4 group sessions held every other week with phone follow-ups occurring in between group sessions. During the *Keep it Low* phase, months 4 through 6, participants will attend 3 monthly group sessions and have 3 telephone follow-ups. There will be a total of 11 in-person sessions and 7 telephone follow-ups over the full study

period. Sessions will be approximately one hour and phone calls will be no longer than thirty minutes. These sessions will consist of diet instruction and supportive counseling, addressing results from 24-hour dietary recalls, teaching self-monitoring and individual diet goal changes, and provide suggestions for replacing usual foods containing carbohydrates. Study materials will be modified from materials used in the successful MACRO study. The JFI will work closely with Drs. He and Bazzano to develop the intervention based on the MACRO study.
Carbohydrate goal: In the MACRO study, carried out among individuals from the same study region, a behavioral intervention aimed at reducing digestible carbohydrate intake to <40 g/day led to reductions in weight and improvements in cardiovascular risk factors among individuals with obesity.[30] Additionally, restriction of carbohydrate intake to <50g/day is sufficient to induce ketosis.[99] Our rationale for having a multi-phased intervention is to allow participants to see how they feel on the more restrictive diet, and see how they respond to small increases in carbohydrate consumption. A similar technique was applied in the DIETFITS published study.[100] The higher target of 60 net grams may be more sustainable long-term and also allows for consumption of healthy higher-carbohydrate foods, such as berries, legumes, beans, and limited whole grains.

8.B. Usual Diet Group

We will provide participants randomized to the usual diet group written information with standard dietary advice. Participants randomized to the usual diet group will not receive ongoing dietary recommendations. Moreover, they will continue to receive clinical care recommendations from their physician(s). To encourage continued participation in the study and minimize the difference in participant contact with study staff, participants will be offered optional monthly educational sessions on topics unrelated to the study intervention, such as retirement planning or health insurance options.

In another effort to encourage continued participation, patients randomized to the usual diet group will be offered a copy of all available intervention materials, as well as an optional one-hour counseling session with the interventionist following their final study visit.

8.C. Physical Activity

At baseline, both groups will receive written information in study materials with standard physical activity recommendations.

8.D. Dietary Adherence

Strategies for measuring compliance: For the low-carbohydrate diet, adherence will be assessed through measurement of urinary ketones at 3-month visit and 6-month TV. Study staff will make all reasonable effort to maintain adherence of participants to the visit schedule and diet intervention.

Strategies for improving compliance:

- At the screening visit, we will ask participants who does most of the cooking in their households and how often they eat outside of the home. Results from the screening visit will help to select a group of cooperative study participants.
- Visits will be scheduled at the participants' convenience and to minimize waiting time and trips to the clinic; reminder calls, texts or emails (chosen based on participant preference) will also be made prior to clinic visits.
- We will hire enthusiastic staff who have good interpersonal skills.
- Participants will receive $40 gift cards for use at food stores or retailers when they complete each study visit, for a maximum of $120 over the study period.
- Participants will receive $25 gift cards when they return the stool specimens, for a maximum of $75 over the study period.

- Participants will receive a $25 gift card when they return the continuous glucose monitor, for a maximum of $25 over the study period.
- Participants will be offered small gifts (such as pens or magnets with study logo) to encourage compliance and adherence to the study protocol.
- Participants will be provided with recipes, shopping lists, and food samples for the intervention diet.

9. Safety of Dietary Intervention

On the low-carbohydrate diet, participants will be encouraged to primarily focus on replacing carbohydrates (particularly highly refined carbohydrates) with foods such as non-starchy vegetables, olive oil and other plant-based unsaturated oils, nuts and seeds, avocados, eggs, seafood, tofu, poultry, and lean meat. Consumption of more unsaturated fats, protein, and fiber will be recommended. Participants will not be encouraged to increase consumption of saturated fats and will be advised to avoid consumption of trans fats.

In animal studies, a high-protein diet has decreased renal function[101] In the MACRO study, on average, participants tended to increase consumption of fats, rather than protein.[30] The safety and tolerability of low-carbohydrate diets have previously been assessed in many studies, including MACRO.[30] A number of studies, including some in individuals with T2D, have found no clinically significant increases in estimated glomerular filtration rates,[33,38–40] microalbuminuria,[102] albumin excretion rate,[33,38] urinary albumin,[38] serum creatinine,[38,40,102–107] albumin,[40] or uric acid levels.[103–106] A small study of a very low-carbohydrate diet found significant increase in uric acid excretion, but no significant change in 24-hour urine creatinine clearance.[106]

Study staff will carefully monitor participants' BP, and HbA1c more often than what would occur in routine healthcare visits. A Data Safety and Monitoring Board (DSMB) consisting of an independent group of experts in areas such as biostatistics, nutrition medicine, general internal medicine, and endocrinology has been formed. The board will regularly review trial progress, including unblinded interim results, and can recommend that the trial prematurely end if participants are at risk or if it becomes clear that the trial will not be able to yield statistically significant results.

10. Data Collection

10.A. Study visits

After the initial pre-screening interview, in which data on medications and medical conditions will be collected, there will be one screening visit, one randomization visit, a follow-up study visit at 3 months and a termination visit (TV) at 6 months. *Table 3* summarizes study procedures and data collected at each study visit.

Table 3. Study visit schedule and data collection

	Pre-screen	SV	RZ	3 month	6 month TV
Pre-screen eligibility	X				
Informed consent		X			
Compliance form		X			
Eligibility confirmation			X		
Randomization			X		
Demographics		X			
Medical history questionnaire			X	X	X
Medications		X	X	X	X
Lifestyle (alcohol, cigarette smoking)			X	X	X
Physical activity			X	X	X
24-hour diet recall*			X	X	X
3 BP measurements		X	X	X	X
Body weight			X	X	X
Height			X		
Waist circumference			X	X	X
Spot urine test			X	X	X
Blood samples		X	X	X	X
Stool specimen			X	X	X
Side-effects assessment			X	X	X
Nut Consumption					X
CGM**					X

*Two 24-hour dietary recalls will be collected; one should cover a weekend and the other a weekday.
**The CGM will be worn for up to 14 days prior to the 6-month TV and returned at the TV or by mail. Participants will come in for a 6-month CGM visit (prior to the 6-month TV) to have the CGM inserted.

10.A.1. Screening/baseline visits:

Participants will be pre-screened by phone or in-person by study staff to confirm eligibility for the Screening Visit. An approved questionnaire will be used to ensure the patient is eligible to attend the in-clinic screening visit, and the patient will be scheduled thereafter.

At the screening visit (SV), participants will provide informed consent for the screening and main study. Study staff will schedule the SV and randomization (RZ) visit at least 2 days apart. If a participant is eligible based on the screening visit, he or she should be scheduled for the RZ once a sufficient group of eligible individuals has been recruited. Participants should be scheduled for the RZ at least 2 days and no more than two weeks after the screening visit.

At the screening visit, we will collect information on inclusion/exclusion criteria, medication use, and demographics. At this visit, we will also have participants complete a compliance form,

to assess their ability to complete the study and attend all intervention and study visits. We will also measure blood pressure, and take blood samples. Women will be asked if they are postmenopausal or if they had surgical sterilization (such as a tubal ligation). If not, they will be tested for pregnancy through a spot urine test; pregnant women will be excluded from the study. Blood samples will be collected for measurement of HbA1c, and, if not available from medical records in the past 3 months, serum creatinine and hemoglobin. These blood samples will be used for excluding individuals with HbA1c <6.0 or >6.9, eGFR ≤45 mL/min/1.73 m^2, or hemoglobin <11 mg/dL.

Before the start of the intervention, participants will complete two 24-hour dietary recalls. At least one (and potentially both) 24-h recalls will be collected by phone. At the RZ, participants will learn about the randomization process. After requirements for eligibility have been satisfied, participants will formally be enrolled into the study and randomized to either the active intervention or control group. We will collect fasting blood samples for later measurement of glucose, insulin and lipids and other potential future measurements. At this visit, we will measure blood pressure, body weight, height, and waist circumference. We will collect information on medical history, lifestyle risk factors, and physical activity. A spot urine sample will be collected to measure ketones and other markers and participants will be asked to collect a stool specimen for potential use in future microbiome studies.

10.A.2. Follow-up visits and final visit: A follow-up visit will occur at 3 months after randomization. A final visit will occur at 6-months post-randomization. These visits should be scheduled as close as possible to the 3-, or 6-months after randomization. At each visit, study staff will conduct dietary recalls and anthropometric measures, take 3 BP measurements, collect a fasting blood sample to measure HbA1c. At least one (and potentially both) 24-h recalls will be collected by phone. We will also collect samples for later measurement of glucose, insulin, lipid profile, and other potential future measurements. A spot urine sample will be collected to measure ketones and other markers. Participants will be asked to collect a stool specimen for potential use in future microbiome studies. Information about participants' nut consumption over the past 6 months will be collected at the 6-month TV. At study termination, we will mail participants and/or their physicians a summary of trial results and a record of participant BP, anthropometric measures, and laboratory data from the trial period. Throughout the study period, we will provide participants with their blood pressure and weight. We will request that, if possible, a participant who is prescribed a diabetes medication during the course of the study comes into the clinic for an additional separate supplemental visit for a fasting blood sample collection prior to starting the medication. Maintaining follow-up of all randomized participants is of utmost importance to ensure data integrity. In the event that a participant moves during the study we may request that the participant provides a fasting blood sample at a local LabCorp facility (this will be described on the informed consent form).

10.A.3. CGM 6-month visit: Individuals who are willing to wear the CGM prior to their 6-month termination visit will come to the research clinic approximately 2 weeks before their 6-month termination visit for their CGM visit. At this time, they will have a factory-calibrated CGM inserted just under the skin of the upper arm and activated.[27] If the CGM falls off of a participant prior to 14 days of wear, we may request that the participant (if willing) returns to the clinic for the CGM to be re-inserted. The CGM will be returned at the 6-month TV, or by mail.

10.B Study measurements

Measurements obtained for this study will be based on those used in other clinical trials and epidemiologic studies. We are familiar with strengths and limitations of such measurements and the importance of staff training and certification and use of stringent quality control procedures.

10.B.1. Questionnaires: Study staff will administer a *medical history questionnaire* at the RZ, 3-month visit and 6-month TV to assess medical conditions that would necessitate changes in therapy or medical management beyond this study's scope. At the SV, *demographic factors* will be collected. At the SV, RZ, 3-month and 6-month TV, we will also administer *medication tracking questionnaires*. At RZ, 3-month, and 6-month TV, we will collect health behavior and physical activity data.

10.B.2. Dietary measurements: Two 24-hour dietary recalls will be obtained from participants at baseline. At or close to both the 3-month and 6-month TV, two more 24-hour dietary recalls will be collected. The second sets of two recalls will be collected as closely as possible to 3- or 6-month dates after randomization, with a goal of being within 2 weeks of the respective follow-up period. For each time point, one recall should reflect week day consumption, while the other should reflect weekend day consumption and the two recalls should be at least 2 days apart from each other. We will use computer software (Nutrition Data System for Research [NDS-R]) and food models used in previous and ongoing studies. Recalls will be conducted by a study staff member who has been certified in the use of NDS-R. Food composition tables of the NDS-R will be used to calculate dietary nutrient intakes. The baseline dietary assessment will be done to characterize individual dietary intake of carbohydrates and other macronutrients. During the intervention, repeated measurements will be used to monitor short-term changes in mean dietary nutrient intake between the intervention and usual diet groups.

10.B.3. Assessment and management of adverse effects: At RZ, 3-month visit, and 6-month TV, we will administer *symptom questionnaires*, which will consist of a checklist to identify participant complaints. Prior studies of low-carbohydrate dietary interventions suggest that participants may experience a range of adverse effects, including constipation, halitosis, fatigue, headache, thirst, polyuria, and nausea. Severe adverse events will be promptly reported to the DSMB. Management of adverse effects will be based on the protection and safety of the participant. If severe adverse effects are reported by a participant, carbohydrate intake will be examined and altered in order to mitigate the adverse effect. In any extreme cases, participants will be instructed to stop the intervention. As this is a behavioral intervention, adverse effects should be rare.

10.B.4. BP and anthropometric measures: BP will be measured 3 times at the screening visit and at each follow-up visit. To minimize random error, averages of the measurements at the screening and randomization visits will be taken. BP will be measured by a standard automated blood pressure measurement device (OMRON HEM-907 XL Professional Digital Blood Pressure Monitor) and performed by experienced staff, trained and certified in BP measurement. Prior to BP measurements, participants will be asked to rest quietly in a seated position with their feet supported for at least 5 minutes; staff members will use an appropriate cuff size over a bare arm. Trained and certified staff will measure weight using a calibrated balance beam scale at the screening visits, and follow-up visits. Weight and height will be measured at the RZ, and weight will also be measured at the subsequent visits.

10.B.5. Blood samples: For SV, participants will not need to fast. For RZ, 3-month visit, and 6-month TV, participants will be instructed to fast for 12 hours prior to blood sample collection. At SV, blood samples will be collected to measure HbA1c, hemoglobin, and serum creatinine. At RZ, fasting blood samples will be collected and stored as serum/plasma, whole blood aliquots and buffy coats at -80°C for future analyses, including measurement of glucose, insulin and lipids, and potential future metabolomics or genetic analyses. At 3-month visit and 6-month TV,

we will collect fasting blood samples to measure HbA1c and will also store additional serum/plasma, whole blood aliquots and buffy coats at -80°C for future analyses, including measurement of glucose, insulin and lipids, and potential future metabolomics or genetic analyses. Details of the blood sample collection are shown in *Table 4*. In the event that a participant moves, we may request that they collect a fasting blood sample at a local LabCorp and we will measure HbA1c, glucose, insulin, and lipids at the 3 and 6-month visits. HOMA-IR will be calculated from fasting glucose and insulin.

Table 4. Study visit schedule for specimen collection

	SV	RZ	3 month	6 month TV
HbA1c	X		X	X
Serum creatinine	X			
Hemoglobin	X			
Storage of aliquots and buffy coats (for measurement of glucose, insulin, lipids, other potential future measurements)		X	X	X
Urine sample for pregnancy	X			
Urine sample for ketones and other markers		X	X	X
Stool sample		X	X	X
CGM				X

10.B.6. Urine collection: Study staff will collect spot urine specimens at RZ, interim visit, and the final visit to measure ketones.

10.B.7. Stool Specimen Collection: Stool samples will be collected from all eligible and willing trial participants during the RZ, 3-month, and 6-month TV, following the protocol set forth by the Human Microbiome Project.[108,109] Participants will be given a stool collection kit, which includes the specimen collection container, a shipper for home collection, and a form to record the timing and pertinent information related to specimen collection. Participants will be instructed to return the kit to the clinic within two days of collection (in-person or via shipping), at which time stool samples are stored at -80°C until analyses. Kits and instructions for collection will be reviewed by study staff with the participant at the required visits.

10.B.8. CGM at 6 months: Approximately two weeks before their 6-month termination visit, participants willing to wear the CGM will come to the clinic to have their CGM inserted. This CGM has been found to be accurate compared with capillary blood glucose reference values over 14 days of wear.[27] This CGM records interstitial glucose every 15 minutes throughout the day for a period of up to 14 days. The sensor uses a thin, flexible filament inserted just under the skin to measure glucose and is read through near-field communication by touching the reader to the sensor. For quality control of the CGM data, a subset of participants who wear the CGM will be asked to wear two CGMs at the same time, one on each arm. Prior to insertion, one arm will be designated as the arm used for data collection and the other arm as a duplicate.

11. Quality Assurance and Quality Control

Throughout the study, we will implement rigorous quality assurance (QA) and quality control (QC) measures. We describe some of these approaches here.

11.A. Manual of procedures

We will develop a manual of procedures (MOP) based on the MOP successfully used in the MACRO behavior change clinical trial. The MOP will provide instructions and describe procedures for staff training and certification, participant recruitment, intervention counseling, study form completion and procedures, data entry, safety measures (e.g., methods for emergency participant/primary care physician contact).

11.B. Training and certification

Trial personnel will all be required to participate in a training session before the start of any trial procedures or measurements. Training will include particular attention to core trial elements, such as recruitment, retention, intervention, completeness and quality of data acquisition (e.g., blinding procedures for the data collectors), and data entry. Separately, intervention staff will meet and receive detailed instruction on trial approaches to group and individual behavior change counseling and procedures that ensure data collectors are blinded to treatment assignment. Throughout the trial, periodic re-training/certification sessions will be conducted to ensure all trial-related procedures are being applied in a standard manner. Investigators will also monitor all aspects of study performance to ensure study goal achievement, timeliness and completeness of study visits, completeness and quality of data collection, intervention adherence, and adequacy of the procedures separating data collection from intervention. Study staff and investigators will pay particularly careful attention to the quality of all laboratory measurements, particularly key exposure, outcome, and intervention monitoring variables.

12. Sample Size and Statistical Power

The primary outcome will be the difference in the change of HbA1c between the intervention and usual diet groups from baseline to 6 months of follow-up. Preliminary data from ADEPT suggests that the SD of the 6-month change in HbA1c is 0.28%, with preliminary 6-month follow-up >90%. Based on an estimated standard deviation of change in HbA1c of 0.28% and 90% follow-up, we would need 112 participants to have 80% power to detect a difference in the change in HbA1c from baseline to 6 months of 0.15% between the intervention and usual diet groups. Assuming 90% follow-up, we would need 126 total participants.

In a follow-up analysis of the DPP, a 0.15% decrement in HbA1c at 6 months was associated with a 16% lower risk of incident T2D, adjusting for treatment arm, age, sex, race/ethnicity, household income, and baseline HbA1c.[115] In a pooled analysis from the Atherosclerosis Risk in Communities and Framingham Heart Studies, a 0.15% higher HbA1c was associated with an 16% higher risk of T2D over 20 years, a 30% higher risk over 8 years, and a 13% higher risk after 8 years, after adjusting for age, sex, fasting glucose, triglycerides, HDL, family history of T2D, SBP, and BMI.[116] Additionally, in EPIC-Norfork, a difference of HbA1c of 0.15% was associated with approximately a 5% higher risk of CHD among those free of CHD, stroke, diabetes, and with HbA1c <7.0%.[117]

In the DPP study, HbA1c was 0.15% lower among those in the lifestyle intervention arm than in the control arm at 6-months follow-up; HbA1c at study commencement was 5.9% and levels increased in the control arm and decreased in the behavioral intervention arm.[22] In a small behavioral intervention study (n=34) of a very low-carbohydrate ketogenic diet compared with a moderate carbohydrate, calorie-restricted, low-fat diet among individuals with type 2 diabetes or prediabetes (HbA1c >6.0%) and elevated body weight (BMI >25), mean HbA1c

decreased from 6.6% to 6.1% in the very low-carbohydrate group and from 6.9% to 6.7% in the moderate carbohydrate group over a 12-month period; additionally, the very low-carbohydrate group had greater reductions in glucose-lowering medications.[37]

Detectable differences are listed for HbA1c and secondary outcomes in *Table 5*, based on data from prior studies.[30,53,56,118] These were calculated for unpaired t-tests, power of 85%, two-sided alpha of 0.05, and assuming 90% follow-up of 126 participants, which would yield an estimated 112 participants.

Table 5. Minimum detectable differences

Change from baseline to 6 months in	SD	Detectable Difference
HbA1c, %	0.28	0.15
Fasting glucose, mg/dL	12.5	6.7
Fasting insulin, mU/mL	7.0	3.8
HOMA-IR	2.0	1.1
SBP (mmHg)	4.2	2.3
DBP (mmHg)	3.0	1.7
Total/HDL ratio	1.2	0.65
Body weight (kg)	6.6	3.6
Waist circumference (cm)	8.0	4.3
Estimated CVD risk (%)*	6.7	4.0

*10-y CVD risk, among estimated 85% or 95 participants free of CVD

13. Data Management and Analysis

13.A. Data management

Personnel will double-enter all study data. Prior to entering data, the study coordinator will conduct quality control on all study forms, study measures, and laboratory data. Data will be cleaned and logically checked.

13.B. Data analysis plan

The data will be analyzed using <u>*intention to treat*</u>; this involves comparing the primary and secondary outcomes between participants based on randomization assignment.

13.B.1. Exploratory data analysis: We will express group data for variables with a normal distribution as mean ± standard deviation and for variables not normally distributed as median and interquartile ranges (or will log-transform the variables to obtain a normal distribution). We will compare means and percentages of baseline variables across the intervention and usual diet group to assess similarity of these groups at randomization.

13.B.2. Primary, secondary, and exploratory outcomes: We will test the primary study hypothesis that there is a greater reduction in HbA1c from baseline to 6 months in the intervention group than in the usual diet group. To do this, we will use a mixed-effects linear regression model, to test the null hypothesis that there is no difference in change between baseline and 6-month HbA1c for the intervention group compared with the usual diet group (Specific Aim 1). To do this, we will use the following model:

$$Y_{i,j,k}\, T = b_0 \quad b_1G_i \quad b_2T_{3\text{-months}} \quad b_3T_{6\text{-months}} \quad b_4G_iT_{3\text{-months}} \quad b_5G_iT_{6\text{-months}} \quad a_{0i,j} \quad e_{i,j,k}$$

where $Y_{i,j,k}$ is HbA1c for the jth participant (P) nested in the ith intervention group (G) at time T_k (baseline, 3 months, and 6 months). This model includes a random intercept ($a_{0i,j}$) and error term $e_{i,j,k}$. The interaction term ($b_5G_iT_{6\text{-months}}$) allows for an interaction between group and $T_{6\text{-months}}$, which is equal to 0 for baseline and 3-months and 1 for 6-months. Rejection of the null hypothesis (b_5=0) indicates there is a significant difference in change of HbA1c from baseline to 6 months between the intervention and control groups. In this model, participants are assumed to be random effects and intervention group, time, and the interaction terms are fixed effects.

We will use a similar approach to test the difference in the change between baseline and 6-month values of secondary outcomes (Specific Aim 2) and exploratory outcomes. In exploratory analyses, we will also test the difference in the change between baseline and 3-month values of outcomes (HbA1c, fasting glucose, systolic blood pressure, total-to-HDL cholesterol, body weight, insulin, HOMA-IR, diastolic blood pressure, waist circumference, and estimated CVD risk). We will also explore the use of mixed effects models to assess overall net change in HbA1c (by treating time as a linear variable) and other outcomes over the full study period. For each outcome, we will test modeling assumptions and will consider transformation of outcomes, if needed, to meet modeling assumptions. As the mixed-effects model assumes missing at random, we will also perform sensitivity analyses using Markov-chain Monte Carlo techniques to impute missing values of the outcomes, using covariates for this imputation and will compare results from this analysis with our primary findings.

15. Anticipated Challenges

Recruitment: Many clinical trials have challenges recruiting sufficient numbers of volunteers who meet the trial inclusion and exclusion criteria and demonstrate a high level of adherence to trial protocol requirements. Fortunately, the investigators are very experienced with clinical trial recruitment, and have several contingency options that can be used to meet unanticipated shortfall in recruitment.

Intervention: The investigators are aware of and understand challenges related to achieving and maintaining compliance with a behavioral intervention. The investigators have much experience and success in behavioral interventions, with recent success of a behavioral intervention aimed at reducing carbohydrate intake among participants from the New Orleans area. The JFI will collaborate with Drs. He and Bazzano to develop the intervention program based on the MACRO study and will assess potential barriers to implementation and intervention adherence (such as financial concerns and range of dietary patterns of study participants) during the planning and pilot phase of the study.

Adherence: Though adherence and follow-up are also main challenges related to clinical trials, the investigators have an outstanding track record for studies that have recruited participants for both clinical and epidemiology studies in the Greater New Orleans area. To encourage adherence, small gifts (such as pens or magnets with study logo) will be offered to all study participants.

16. Protection of Human Subjects

This Human Subjects Research meets the definition of a clinical trial. All investigators will be required to complete the CITI (Collaborative Institutional Training Initiative) program in the ethical use of human participants in research.

16.A. Protection of human subjects from research risks

16.A.1. Risks to the subjects

Human subject involvement and characteristics: We expect to enroll 126 participants, with an equal male to female ratio. We expect to enroll approximately 30 participants who are African-Americans, and another 10 with mixed racial backgrounds, and a few Asian participants given the demographics of the greater New Orleans area. Overall, given the demographics of greater New Orleans, we estimate approximately 10% participants of Hispanic or Latino ethnicity, 50% of them with African-American racial background.

Inclusion criteria

- Men or women ages 40 to 70 years: aim to recruit 50% women
- Any race or ethnicity: aim to recruit 40% ethnic minorities (i.e., African American and Hispanic), close to population in the greater New Orleans area[94]
- HbA1c 6.0–6.9%
- Willing and able to provide informed consent

Exclusion criteria

- Diagnosed type 1 diabetes mellitus based on patient self-report
- Use of agents affecting glycemic control (medications for diabetes, oral glucocorticoids) within the past three months prior to enrollment based on patient self-report
- Medical condition in which low-carbohydrate diet may not be advised
  - Estimated glomerular filtration rate (eGFR) ≤45 mL/min/1.73 m^2, which is close to the 5th percentile of eGFR among non-diseased individuals of 70 years of age[95]
  - Self-report of liver disease due to hepatitis or alcohol; osteoporosis; untreated thyroid disease; gout; or cancer (other than non-melanoma skin cancer) requiring treatment in the past year, unless prognosis is excellent
- Factors that may affect HbA1c:
  - Hemoglobin <11 mg/dL (cutpoint for moderate-to-severe anemia, which could lead to falsely elevated or lowered HbA1c)[96]
  - Recent blood donation or blood transfusion (self-report, past 4 months)
  - Human Immunodeficiency Virus (self-report)[97]
- Self-reported history of intensive care unit stay due to Coronavirus Disease 2019 (COVID-19) in the past three months, as severe COVID-19 may affect blood glucose levels
- Allergies to nuts
- For women, current pregnancy, breastfeeding, or plans to become pregnant during the study
- Consumption of ≥21 alcoholic drinks per week or consumption of ≥6 drinks per occasion
- For the CGM collection at the end of the study period only: known allergy to adhesives or other products involved with CGM use (e.g., skin disinfectants), current pregnancy, currently on hemodialysis or peritoneal dialysis, or people with other implanted medical devices (e.g., a pacemaker)
- Current or planned residence making it difficult to meet trial requirements (due to distance from study site and/or challenges regularly traveling to site)
- Current participation in another lifestyle intervention trial or a pharmaceutical trial
- Participation of another household member in the study; employees or persons living with employees of the study
- Other concerns regarding ability to meet trial requirements, at the discretion of the principal investigator or study coordinator

Sources of materials: Medical information data will be collected from the participant and, after written authorization for the disclosure of protected health information, from participants' medical records. Blood and urine samples will be collected to measure HbA1c, glucose, insulin, lipids, creatinine, ketones, and test for pregnancy. Blood pressure, height, weight, waist circumference and other physical measurements will be obtained.

Potential Risks: Risks associated with venipuncture, the diet intervention, CGM insertion, and collection of confidential medical information are the main risks associated with this study. There is a small risk of bruising and a rare chance of local infection associated with standard venipuncture to collect blood samples. The risks of wearing the CGM are minimal. There is a very low risk for local infection at the skin site where the sensor is inserted into the skin. Possible symptoms associated with the CGM sensor application or the adhesive used to keep the sensor in place include skin irritation or redness, skin inflammation, pain or discomfort, bleeding, bruising, swelling, skin rash, itching, scarring or skin discoloration, or allergic reactions to the sensor needle or adhesives. Sensors may fracture in situ on rare occasions. The dietary intervention has minimal risks. Reduced carbohydrate consumption may lead to adverse effects, including constipation, halitosis, fatigue, headache, thirst, polyuria, and nausea. At RZ, 3- , and 6-month visits, we will administer *symptom questionnaires*, which will consist of a checklist to identify participant complaints. Adverse effects of the low-carbohydrate diet will be assessed at counseling sessions. Severe adverse events will be promptly reported to the DSMB. Management of adverse effects will be based on the protection and safety of the participant. If severe adverse effects are reported by a participant, carbohydrate intake will be examined and altered in order to mitigate the adverse effect. In any extreme cases, participants will be instructed to stop the intervention. Furthermore, there is a potential risk of unauthorized disclosure of medical information. This risk will be minimized by using a strict protocol for data processing and by the appropriate training of all study personnel. Protection against risks is described in more detail below.

16.A.2. Adequacy of protection against risks

Recruitment and Informed Consent: The proposed study will be conducted following strict guidelines for the protection of the rights of human volunteers. The informed consent document will be signed by all participants at the screening/baseline visit. The informed consent form will be developed in the first year of the study and approved by the IRB. The consent form will clearly state the purpose, eligibility criteria, and protocol of the study, the potential benefits and risks of participation, and the subject's right to refuse to participate or withdraw. Prior to signing the informed consent, research staff will review the details of the consent form orally with the participant, and answer any questions that the participant has concerning participation in the study. In the case of potential participants with low or no literacy, the informed consent form will be read to the participant in its entirety and any questions will be answered prior to the participant signing or marking the form. Specifically, the following must be accomplished during the informed consent process:

- The participant must be informed that participation in the study is voluntary and that refusal to participate will involve no penalty or loss of benefits.
- The participant must be informed of the purpose of the study and that it involves research.
- The participant must be informed of any alternative procedures, if applicable.
- The participant must be informed of any reasonably foreseeable risks.
- The participant must be informed of any benefits from the research.
- An outline of safeguards to protect participant confidentiality will be included, as well as an indication of the participant’s right to withdraw without penalty. This should be balanced with a discussion of the effect that withdrawals will have on the study, and the responsibility a participant has, within limits, to continue in the study if he or she decides to enroll.

- The participant must be informed of his or her right to have questions answered at any time and whom to contact for answers or in the event of research-related injury.
- The participant must be informed as to whether or not compensation is offered for participation in the study and/or in the event of a medical injury.
- The participant must be informed that he/she will be notified of any significant changes in the protocol that might affect their willingness to continue in the study.

Written authorization for the disclosure of protected health information will be obtained during the consent process on a separate form (HIPPA authorization), if applicable.

Protection against Risk: The protocol and informed consent will be approved by the Institutional Review Boards at Tulane University Health Sciences Center and any other participating institution for recruitment. The study participants will be given contact information for the Study Coordinator and Study PI to answer questions or to report complaints. Patients can withdraw from the study at any time without any effect on their medical care. Standard techniques will be used by trained, certified phlebotomists or study nurses for blood sample collection at the study clinics where emergency medical equipment is available. Confidentiality of all data collected during the study will be maintained through the use of unique, encrypted study identification (ID numbers, rather than patient names, in the study database). These encrypted ID numbers will not be mathematical derivation of the subject's medical record number or any other patient identifier. No information will be disclosed in an individually identifiable form in any type of presentation or publication. There will be password-required access to the computerized file linking the study numbers to patient identifiers at the study clinics. Password-access will be restricted to pertinent research staff only. All study data will be kept in locked file cabinets in locked rooms, accessible only by study staff with permission. All data transmissions will use HIPPA-compliant password protected system and will be secured through a virtual private network.

16.A.3. Potential benefits of the proposed research to human subjects and others

Study participants may have improved glycemic outcomes, blood pressure, lipid levels, may lose weight and reduce waist circumference, and may have improvement in other risk factors for CVD and development and progression of T2D. Participants will receive multiple blood pressure, weight, waist circumference, and blood tests. If vital signs or lab values are clinically abnormal, participants will be referred to their primary care physician, or if they do not have a primary care physician, one will be assigned through the Tulane University Community Health Center, which is a part of the core clinical facilities. At the end of the study, we will provide the participants' primary care physician with a copy of all the blood pressure, weight, laboratory results, and (if applicable) CGM results.

16.A.4. Importance of knowledge to be gained

Information from this study may contribute to reducing the onset of T2D and the burden of T2D and cardiovascular disease in Louisiana and the general US population. The study has potential for public health impact; results from this study may provide evidence in support of promoting alternative dietary styles (than the most commonly used low-fat diet) among individuals with high T2D risk or with T2D.

16.A.5. Remuneration

For screening visit 2, the two follow-up visits, and the final visits, participants will receive $40 gift cards for use at food stores or retailers (after completing each study visit), for a maximum of

$120 over the study period. Participants who return a stool sample will receive $25 gift cards for each stool sample that they return, for a total of $75 for three stool samples. For the CGM 6-month study, participants will receive $25 gift cards for returning the CGM, for a maximum of $25. Participants will also receive free parking for each intervention and study visit.

16.B. Data safety and monitoring plan

A DSMB, which consists of 3 experts who are not otherwise affiliated with the COBRE program, has been formed. Study investigators for this study will prepare accurate and timely data tables and reports for the DSMB meetings. The members of the DSMB will serve in an individual capacity and provide their expertise and recommendations. The primary responsibilities of the DSMB are: 1) periodically review and evaluate the accumulated study data for participant safety, study conduct and progress, and, when appropriate, efficacy, and 2) make recommendations to the NIH concerning the continuation, modification, or termination of the trial. The DSMB considers study-specific data, as well as relevant background knowledge, about the disease, test agent, and patient population under study.

The DSMB is responsible for defining its deliberative processes, including event triggers that would call for an unscheduled review, stopping guidelines, unmasking (unblinding) and voting procedures prior to initiating any data review. The DSMB is also responsible for maintaining the confidentiality of its internal discussions and activities, as well as the contents of reports provided to it.

The DSMB will review each protocol for any major concerns prior to implementation. During the trial, the DSMB should review cumulative study data to evaluate safety, study conduct, and scientific validity and integrity of the trial. As part of this responsibility, DSMB members must be satisfied that the timeliness, completeness, and accuracy of the data submitted to them for review are sufficient for evaluation of the safety and welfare of study participants. The DSMB should also assess the performance of overall study operations and any other relevant issues, as necessary.

Items reviewed by the DSMB include:

- Interim/cumulative data for evidence of study-related adverse events;
- Interim/cumulative data for evidence of efficacy according to pre-established statistical guidelines, if appropriate;
- Data quality, completeness, and timeliness;
- Adequacy of compliance with goals for recruitment and retention, including those related to the participation of women and minorities;
- Adherence to the protocol;
- Factors that might affect the study outcome or compromise the confidentiality of the trial data (such as protocol violations, unmasking, etc.); and,
- Factors external to the study, such as scientific or therapeutic developments, that may impact participant safety or the ethics of the study.

The DSMB will conclude each review with their recommendations to the COBRE program and NIH/NIGMS as to whether the study should continue without change or be modified or terminated. Recommendations regarding modification of the design and conduct of the study could include:

- Modifications of the study protocol based upon review of the safety data;
- Suspension or early termination of the study or of one or more study arms because of serious concerns about subjects' safety, inadequate performance or rate of enrollment;

- Suspension or early termination of the study or of one or more study arms because study objectives have not been obtained according to pre-established statistical guidelines; and,
- Corrective actions regarding a study when the performance appears unsatisfactory or suspicious.

Confidentiality must always be maintained during all phases of DSMB review and deliberation. Usually, only voting members of the DSMB should have access to interim analyses of outcome data by treatment group. Exceptions may be made when the DSMB deems it appropriate. The reason and to whom the exceptions for access to interim analyses is granted will be documented in the Closed Session Report. DSMB members must maintain strict confidentiality concerning all privileged trial results ever provided to them. The DSMB should review data only by masked study group (such as X vs. Y rather than experimental vs. control) unless or until the DSMB determines that the identities of the groups are necessary for their decision-making. Whenever masked data are presented to the DSMB, the key to the group coding must be available for immediate unmasking.

The DSMB will meet twice per year. The junior faculty investigator will attend these meetings and will be responsible for preparing and presenting up-to-date statistical reports on the progress of their trials. These reports will include data on recruitment, randomization, adherence, as well as statistical tests and special analyses requested by the DSMB. The Administrative Core will be responsible for organizing the annual in-person meeting and semiannual conference calls. They will put together data and materials submitted by junior faculty investigators and deliver them to DSMB members. The COBRE investigators will meet with the DSMB in the Open Session and not be present for the Closed Session.

Bibliography and References Cited

1. The Emerging Risk Factors Collaboration. Diabetes mellitus, fasting blood glucose concentration, and risk of vascular disease: a collaborative meta-analysis of 102 prospective studies. *Lancet*. 2010;375(9733):2215-2222. doi:10.1016/S0140-6736(10)60484-9.
2. Flaxman SR, Bourne RRA, Resnikoff S, et al. Global causes of blindness and distance vision impairment 1990-2020: a systematic review and meta-analysis. *Lancet Glob Heal*. October 2017. doi:10.1016/S2214-109X(17)30393-5.
3. United States Renal Data System. *2016 USRDS Annual Data Report: Epidemiology of Kidney Disease in the United States*. Bethesda, MD: National Institutes of Health, National Institute of Diabetes and Digestive and Kidney Diseases; 2016. https://www.usrds.org/2016/view/Default.aspx.
4. Narres M, Kvitkina T, Claessen H, et al. Incidence of lower extremity amputations in the diabetic compared with the non-diabetic population: A systematic review. *PLoS One*. 2017;12(8):e0182081. doi:10.1371/journal.pone.0182081.
5. Centers for Disease Control and Prevention. *National Diabetes Statistics Report, 2017*. Atlanta, GA: Centers for Disease Control and Prevention, U.S. Dept of Health and Human Services; 2017.
6. Centers for Disease Control and Prevention. *Long-Term Trends in Diabetes*. Atlanta, GA: Centers for Disease Control and Prevention, U.S. Dept of Health and Human Services; 2017.
7. NCD Risk Factor Collaboration (NCD-RisC). Worldwide trends in diabetes since 1980: A pooled analysis of 751 population-based studies with 4.4 million participants. *Lancet*. 2016;387(10027):1513-1530. doi:10.1016/S0140-6736(16)00618-8.
8. American Diabetes Association. Economic costs of diabetes in the U.S. in 2012. *Diabetes Care*. 2013;36(4):1033-1046. doi:10.2337/dc12-2625.
9. Zhuo X, Zhang P, Barker L, Albright A, Thompson TJ, Gregg E. The lifetime cost of diabetes and its implications for diabetes prevention. *Diabetes Care*. 2014;37(9):2557-2564. doi:10.2337/dc13-2484.
10. WHO, International Diabetes Foundation. *Definition and Diagnosis of Diabetes Mellitus and Intermediate Hyperglycaemia: Report of a WHO/IDF Consultation*. Geneva: Geneva: World Health Organization; 2006.
11. The International Expert Committee. International Expert Committee Report on the Role of the A1C Assay in the Diagnosis of Diabetes. *Diabetes Care*. 2009;32(7):1327-1334. doi:10.2337/dc09-9033.
12. American Diabetes Association. Standards of Medical Care in Diabetes - 2017. *Diabetes Care*. 2017;40(Supplement 1):S33-S43. doi:10.2337/dc16-S003.
13. Gerstein HC, Santaguida P, Raina P, et al. Annual incidence and relative risk of diabetes in people with various categories of dysglycemia: A systematic overview and meta-analysis of prospective studies. *Diabetes Res Clin Pract*. 2007;78(3):305-312. doi:10.1016/j.diabres.2007.05.004.
14. Huang Y, Cai X, Mai W, Li M, Hu Y. Association between prediabetes and risk of cardiovascular disease and all cause mortality: systematic review and meta-analysis. *BMJ*. 2016;355:i5953. doi:10.1136/bmj.i5953.
15. Tabák AG, Herder C, Rathmann W, Brunner EJ, Kivimäki M. Prediabetes: A high-risk state for diabetes development. *Lancet*. 2012;379(9833):2279-2290. doi:10.1016/S0140-

6736(12)60283-9.

16. Diabetes Prevention Program Research Group. 10-year follow-up of diabetes incidence and weight loss in the Diabetes Prevention Program Outcomes Study. *Lancet*. 2009;374(9702):1677-1686. doi:10.1016/S0140-6736(09)61457-4.
17. The DREAM (Diabetes REduction Assessment with ramipril and rosiglitazone Medication) Trial Investigators. Effect of rosiglitazone on the frequency of diabetes in patients with impaired glucose tolerance or impaired fasting glucose: a randomised controlled trial. *Lancet*. 2006;368(9541):1096-1105. doi:10.1016/S0140-6736(06)69420-8.
18. Perreault L, Pan Q, Mather KJ, Watson KE, Hamman RF, Kahn SE. Effect of regression from prediabetes to normal glucose regulation on long-term reduction in diabetes risk: Results from the Diabetes Prevention Program Outcomes Study. *Lancet*. 2012;379(9833):2243-2251. doi:10.1016/S0140-6736(12)60525-X.
19. Perreault L, Temprosa M, Mather KJ, et al. Regression from prediabetes to normal glucose regulation is associated with reduction in cardiovascular risk: Results from the diabetes prevention program outcomes study. *Diabetes Care*. 2014;37(9):2622-2631. doi:10.2337/dc14-0656.
20. Popkin BM. Nutrition Transition and the Global Diabetes Epidemic. *Curr Diab Rep*. 2015;15(9). doi:10.1007/s11892-015-0631-4.
21. Vazquez G, Duval S, Jacobs DRJ, Silventoinen K. Comparison of body mass index, waist circumference, and waist/hip ratio in predicting incident diabetes: a meta-analysis. *Epidemiol Rev*. 2007;29:115-128. doi:10.1093/epirev/mxm008.
22. Knowler WC, Barrett-Connor E, Fowler SE, et al. Reduction in the incidence of type 2 diabetes with lifestyle intervention or metformin. *N Engl J Med*. 2002;346(6):393-403. doi:10.1056/NEJMoa012512.
23. Pan XR, Li GW, Hu YH, et al. Effects of diet and exercise in preventing NIDDM in people with impaired glucose tolerance: The Da Qing IGT and diabetes study. *Diabetes Care*. 1997;20(4):537-544. doi:10.2337/diacare.20.4.537.
24. Tuomilehto J, Indstrom J, Eriksson J, Valle T, Hamalainine E, Uusitupa M. Prevention of Type 2 Diabetes Mellitus By Changes in Lifestyle Among Subjects With Impaired Glucose Tolerance. *N Engl J Med*. 2001;344(18):1343-1350. doi:10.1056/NEJM200105033441801.
25. Ramachandran A, Snehalatha C, Mary S, Mukesh B, Bhaskar AD, Vijay V. The Indian Diabetes Prevention Programme shows that lifestyle modification and metformin prevent type 2 diabetes in Asian Indian subjects with impaired glucose tolerance (IDPP-1). *Diabetologia*. 2006;49(2):289-297. doi:10.1007/s00125-005-0097-z.
26. The Diabetes Preventio Program Research Group. The Diabetes Prevention Program. Design and methods for a clinical trial in the prevention of type 2 diabetes. *Diabetes Care*. 1999;22(4):623-634. doi:10.2337/diacare.22.4.623 10.2337/diabetes.48.4.699.
27. Evert AB, Boucher JL, Cypress M, et al. Nutrition therapy recommendations for the management of adults with diabetes. *Diabetes Care*. 2013;36(11):3821-3842. doi:10.2337/dc13-2042.
28. Hu T, Mills KT, Yao L, et al. Effects of low-carbohydrate diets versus low-fat diets on metabolic risk factors: A meta-analysis of randomized controlled clinical trials. *Am J Epidemiol*. 2012;176(SUPPL. 7). doi:10.1093/aje/kws264.
29. Sackner-Bernstein J, Kanter D, Kaul S. Dietary Intervention for Overweight and Obese Adults: Comparison of Low-Carbohydrate and Low-Fat Diets. A Meta-Analysis. *PLoS*

One. 2015;10(10):e0139817. doi:10.1371/journal.pone.0139817.

30. Bazzano LA, Hu T, Reynolds K, et al. Effects of low-carbohydrate and low-fat diets: A randomized trial. *Ann Intern Med*. 2014;161(5):309-318. doi:10.7326/M14-0180.
31. Sawyer L, Gale EAM. Diet, delusion and diabetes. *Diabetologia*. 2009;52(1):1-7. doi:10.1007/s00125-008-1203-9.
32. Snorgaard O, Poulsen GM, Andersen HK, Astrup A. Systematic review and meta-analysis of dietary carbohydrate restriction in patients with type 2 diabetes. *BMJ Open Diabetes Res Care*. 2017;5(1):e000354. doi:10.1136/bmjdrc-2016-000354.
33. Larsen RN, Mann NJ, Maclean E, Shaw JE. The effect of high-protein, low-carbohydrate diets in the treatment of type 2 diabetes: A 12 month randomised controlled trial. *Diabetologia*. 2011;54(4):731-740. doi:10.1007/s00125-010-2027-y.
34. Saslow LR, Kim S, Daubenmier JJ, et al. A randomized pilot trial of a moderate carbohydrate diet compared to a very low carbohydrate diet in overweight or obese individuals with type 2 diabetes mellitus or prediabetes. *PLoS One*. 2014;9(4). doi:10.1371/journal.pone.0091027.
35. Tay J, Luscombe-Marsh ND, Thompson CH, et al. A very low-carbohydrate, low-saturated fat diet for type 2 diabetes management: A randomized trial. *Diabetes Care*. 2014;37(11):2909-2918. doi:10.2337/dc14-0845.
36. Davis NJ, Tomuta N, Schechter C, et al. Comparative Study of the Effects of a 1-Year Dietary Intervention of a Low- Carbohydrate Diet Versus a Low-Fat Diet on Weight and Glycemic Control in Type 2. *Diabetes Care*. 2009;32(7):1147-1152. doi:10.2337/dc08-2108.Clinical.
37. Saslow LR, Daubenmier JJ, Moskowitz JT, et al. Twelve-month outcomes of a randomized trial of a moderate-carbohydrate versus very low-carbohydrate diet in overweight adults with type 2 diabetes mellitus or prediabetes. *Nutr Diabetes*. 2017;7(12):304. doi:10.1038/s41387-017-0006-9.
38. Tay J, Thompson CH, Luscombe-Marsh ND, et al. Effects of an energy-restricted low-carbohydrate, high unsaturated fat/low saturated fat diet versus a high carbohydrate, low fat diet in type 2 diabetes: a 2 year randomized clinical trial. *Diabetes, Obes Metab*. 2017:n/a-n/a. doi:10.1111/dom.13164.
39. Yamada Y, Uchida J, Izumi H, et al. A non-calorie-restricted low-carbohydrate diet is effective as an alternative therapy for patients with type 2 diabetes. *Intern Med*. 2014;53(1):13-19. doi:http://dx.doi.org/10.2169/internalmedicine.53.0861.
40. Mayer SB, Jeffreys AS, Olsen MK, Mcduffie JR, Feinglos MN, Yancy WS. Two diets with different haemoglobin A1c and antiglycaemic medication effects despite similar weight loss in type 2 diabetes. *Diabetes, Obes Metab*. 2014;16(1):90-93. doi:10.1111/dom.12191.
41. Maekawa S, Kawahara T, Nomura R, et al. Retrospective study on the efficacy of a low-carbohydrate diet for impaired glucose tolerance. *Diabetes Metab Syndr Obes*. 2014;7:195-201. doi:10.2147/DMSO.S62681.
42. Davis C, Bryan J, Hodgson J, Murphy K. Definition of the mediterranean diet: A literature review. *Nutrients*. 2015;7(11):9139-9153. doi:10.3390/nu7115459.
43. Sofi F, Macchi C, Abbate R, Gensini GF, Casini A. Mediterranean diet and health status: an updated meta-analysis and a proposal for a literature-based adherence score. *Public Health Nutr*. 2014;17(12):2769-2782. doi:10.1017/S1368980013003169.
44. Jannasch F, Kröger J, Schulze MB. Dietary Patterns and Type 2 Diabetes: A Systematic Literature Review and Meta-Analysis of Prospective Studies. *J Nutr*. 2017;147(6):1174-

1182. doi:10.3945/jn.116.242552.

45. Schwingshackl L, Missbach B, König J, Hoffmann G. Adherence to a Mediterranean diet and risk of diabetes: a systematic review and meta-analysis. *Public Health Nutr*. 2014;18(7):1292–1299. doi:10.1017/S1368980014001542.

46. de Lorgeril M, Salen P, Martin J-L, Monjaud I, Delaye J, Mamelle N. Mediterranean Diet, Traditional Risk Factors, and the Rate of Cardiovascular Complications After Myocardial Infarction : Final Report of the Lyon Diet Heart Study. *Circulation*. 1999;99(6):779-785. doi:10.1161/01.CIR.99.6.779.

47. Estruch R, Ros E, Salas-Salvadó J, et al. Primary Prevention of Cardiovascular Disease with a Mediterranean Diet. *N Engl J Med*. 2013;368(14):1279-1290. doi:10.1056/NEJMoa1200303.

48. Salas-Salvadó J, Bulló M, Estruch R, et al. Prevention of diabetes with mediterranean diets: A subgroup analysis of a randomized trial. *Ann Intern Med*. 2014;160(1):1-10. doi:10.7326/M13-1725.

49. Esposito K, Marfella R, Ciotola M, et al. Effect of a Mediterranean-Style Diet on Endothelial Dysfunction and Markers of Vascular Inflammation in the Metabolic Syndrome. *JAMA*. 2004;292(12):1440. doi:10.1001/jama.292.12.1440.

50. Babio N, Toledo E, Estruch R, et al. Mediterranean diets and metabolic syndrome status in the PREDIMED randomized trial. *CMAJ*. 2014;186(17):E649-E657. doi:10.1503/cmaj.140764.

51. Esposito K, Maiorino MI, Bellastella G, Chiodini P, Panagiotakos D, Giugliano D. A journey into a Mediterranean diet and type 2 diabetes: a systematic review with meta-analyses. *BMJ Open*. 2015;5(8):e008222. doi:10.1136/bmjopen-2015-008222.

52. Micha R, Michas G, Mozaffarian D. Unprocessed red and processed meats and risk of coronary artery disease and type 2 diabetes--an updated review of the evidence. *Curr Atheroscler Rep*. 2012;14(6):515-524. doi:10.1007/s11883-012-0282-8.

53. Esposito K, Maiorino MI, Ciotola M, et al. Effects of a Mediterranean-style diet on the need for antihyperglycemic drug therapy in patients with newly diagnosed type 2 diabetes: A randomized trial. *Ann Intern Med*. 2009;151(5):306-314. doi:10.1097/OGX.0b013e3181e5a159.

54. Esposito K, Maiorino MI, Petrizzo M, Bellastella G, Giugliano D. The effects of a Mediterranean diet on the need for diabetes drugs and remission of newly diagnosed type 2 diabetes: Follow-up of a randomized trial. *Diabetes Care*. 2014;37(7):1824-1830. doi:10.2337/dc13-2899.

55. Elhayany A, Lustman A, Abel R, Attal-Singer J, Vinker S. A low carbohydrate Mediterranean diet improves cardiovascular risk factors and diabetes control among overweight patients with type 2 diabetes mellitus: A 1-year prospective randomized intervention study. *Diabetes, Obes Metab*. 2010;12(3):204-209. doi:10.1111/j.1463-1326.2009.01151.x.

56. Shai I, Schwarzfuchs D, Henkin Y, et al. Weight Loss with a Low-Carbohydrate, Mediterranean, or Low-Fat Diet. *N Engl J Med*. 2008;359(3):229-241. doi:10.1056/NEJMoa0708681.

57. Schwarzfuchs D, Golan R, Shai I. Four-Year Follow-up after Two-Year Dietary Interventions. *N Engl J Med*. 2012;367(14):1373-1374. doi:10.1056/NEJMc1204792.

58. Pan A, Sun Q, Bernstein AM, et al. Red meat consumption and risk of type 2 diabetes: 3 Cohorts of US adults and an updated meta-analysis. *Am J Clin Nutr*. 2011;94(4):1088-1096. doi:10.3945/ajcn.111.018978.

59. Hu EA, Pan A, Malik V, Sun Q. White rice consumption and risk of type 2 diabetes: meta-analysis and systematic review. *BMJ*. 2012;344:e1454. doi:10.1136/bmj.e1454.
60. Wang M, Yu M, Fang L, Hu R-Y. Association between sugar-sweetened beverages and type 2 diabetes: A meta-analysis. *J Diabetes Investig*. 2015;6(3):360-366. doi:10.1111/jdi.12309.
61. Imamura F, O'Connor L, Ye Z, et al. Consumption of sugar sweetened beverages, artificially sweetened beverages, and fruit juice and incidence of type 2 diabetes: systematic review, meta-analysis, and estimation of population attributable fraction. *BMJ*. 2015:h3576. doi:10.1136/bmj.h3576.
62. Carter P, Gray LJ, Troughton J, Khunti K, Davies MJ. Fruit and vegetable intake and incidence of type 2 diabetes mellitus: systematic review and meta-analysis. *BMJ*. 2010;341:c4229. doi:10.1136/bmj.c4229.
63. Cooper AJ, Forouhi NG, Ye Z, et al. Fruit and vegetable intake and type 2 diabetes: EPIC-InterAct prospective study and meta-analysis. *Eur J Clin Nutr*. 2012;66(10):1082-1092. doi:10.1038/ejcn.2012.85.
64. Chen M, Sun Q, Giovannucci E, et al. Dairy consumption and risk of type 2 diabetes: 3 cohorts of US adults and an updated meta-analysis. *BMC Med*. 2014;12(1):215. doi:10.1186/s12916-014-0215-1.
65. Aune D, Norat T, Romundstad P, Vatten LJ. Whole grain and refined grain consumption and the risk of type 2 diabetes: A systematic review and dose-response meta-analysis of cohort studies. *Eur J Epidemiol*. 2013;28(11):845-858. doi:10.1007/s10654-013-9852-5.
66. Ding M, Bhupathiraju SN, Chen M, van Dam RM, Hu FB. Caffeinated and decaffeinated coffee consumption and risk of type 2 diabetes: a systematic review and a dose-response meta-analysis. *Diabetes Care*. 2014;37(2):569-586. doi:10.2337/dc13-1203.
67. Li X-H, Yu F-F, Zhou Y-H, He J. Association between alcohol consumption and the risk of incident type 2 diabetes: a systematic review and dose-response meta-analysis. *Am J Clin Nutr*. 2016;103(3):818-829. doi:10.3945/ajcn.115.114389.
68. Muraki I, Imamura F, Manson JE, et al. Fruit consumption and risk of type 2 diabetes: results from three prospective longitudinal cohort studies. *BMJ*. 2013;347(aug28 1):f5001-f5001. doi:10.1136/bmj.f5001.
69. Upadhyaya S, Banerjee G. Type 2 diabetes and gut microbiome: At the intersection of known and unknown. *Gut Microbes*. 2015;6(2):85-92. doi:10.1080/19490976.2015.1024918.
70. David LA, Maurice CF, Carmody RN, et al. Diet rapidly and reproducibly alters the human gut microbiome. *Nature*. 2014;505(7484):559-563. doi:10.1038/nature12820.
71. Duncan SH, Belenguer A, Holtrop G, Johnstone AM, Flint HJ, Lobley GE. Reduced dietary intake of carbohydrates by obese subjects results in decreased concentrations of butyrate and butyrate-producing bacteria in feces. *Appl Environ Microbiol*. 2007;73(4):1073-1078. doi:10.1128/AEM.02340-06.
72. Dorans KS, Mostofsky E, Levitan EB, Håkansson N, Wolk A, Mittleman MA. Alcohol and Incident Heart Failure among Middle-Aged and Elderly Men: Cohort of Swedish Men. *Circ Hear Fail*. 2015;8(3):422-427. doi:10.1161/CIRCHEARTFAILURE.114.001787.
73. Steinhaus DA, Mostofsky E, Levitan EB, et al. Chocolate intake and incidence of heart failure: Findings from the Cohort of Swedish Men. *Am Heart J*. 2017;183:18-23. doi:10.1016/j.ahj.2016.10.002.
74. Dorans KS, Massa J, Chitnis T, Ascherio A, Munger KL. Physical activity and the incidence of multiple sclerosis. *Neurology*. 2016;87(17):1770-1776.

doi:10.1212/WNL.0000000000003260.

75. Mills KT, Chen J, Yang W, et al. Sodium Excretion and the Risk of Cardiovascular Disease in Patients With Chronic Kidney Disease. *JAMA*. 2016;315(20):2200. doi:10.1001/jama.2016.4447.
76. Chen J, Gu D, Huang J, et al. Metabolic syndrome and salt sensitivity of blood pressure in non-diabetic people in China: a dietary intervention study. *Lancet*. 2009;373(9666):829-835. doi:10.1016/S0140-6736(09)60144-6.
77. Li C, He J, Chen J, et al. Genome-Wide Gene-Sodium Interaction Analyses on Blood Pressure: The Genetic Epidemiology Network of Salt-Sensitivity Study. *Hypertension*. 2016;68(2):348-355. doi:10.1161/HYPERTENSIONAHA.115.06765.
78. He J, Streiffer RH, Muntner P, Krousel-Wood MA, Whelton PK. Effect of dietary fiber intake on blood pressure: a randomized, double-blind, placebo-controlled trial. *J Hypertens*. 2004;22(1):73-80. doi:10.1097/01.hjh.0000098164.70956.54.
79. He J, Ogden LG, Vupputuri S, Bazzano LA, Loria C, Whelton PK. Dietary sodium intake and subsequent risk of cardiovascular disease in overweight adults. *JAMA*. 1999;282(21):2027-2034. doi:10.1001/jama.282.21.2027.
80. He J, Wofford MR, Reynolds K, et al. Effect of dietary protein supplementation on blood pressure a randomized, controlled trial. *Circulation*. 2011;124(5):589-595. doi:10.1161/CIRCULATIONAHA.110.009159.
81. Thompson AM, Zhang Y, Tong W, et al. Association of inflammation and endothelial dysfunction with metabolic syndrome, prediabetes and diabetes in adults from Inner Mongolia, China. *BMC Endocr Disord*. 2011;11(1):16. doi:10.1186/1472-6823-11-16.
82. Irazola V, Rubinstein A, Bazzano L, et al. Prevalence, awareness, treatment and control of diabetes and impaired fasting glucose in the Southern Cone of Latin America. *PLoS One*. 2017;12(9). doi:10.1371/journal.pone.0183953.
83. Bazzano LA, Li TY, Joshipura KJ, Hu FB. Intake of fruit, vegetables, and fruit juices and risk of diabetes in women. *Diabetes Care*. 2008;31(7):1311-1317. doi:10.2337/dc08-0080.
84. Bazzano LA, Serdula M, Liu S. Prevention of Type 2 Diabetes by Diet and Lifestyle Modification. *J Am Coll Nutr*. 2005;24(5):310-319. doi:10.1080/07315724.2005.10719479.
85. Pollock BD, Chen W, Harville EW, et al. Differential sex effects of systolic blood pressure and low-density lipoprotein cholesterol on type 2 diabetes: Life course data from the Bogalusa Heart Study. *Journal of Diabetes*. 2017.
86. Zhang T, Li Y, Zhang H, et al. Insulin-sensitive adiposity is associated with a relatively lower risk of diabetes than insulin-resistant adiposity: the Bogalusa Heart Study. *Endocrine*. 2016;54(1):93-100. doi:10.1007/s12020-016-0948-z.
87. Pollock BD, Hu T, Chen W, et al. Utility of existing diabetes risk prediction tools for young black and white adults: Evidence from the Bogalusa Heart Study. *J Diabetes Complications*. 2016;31(1):86-93. doi:10.1016/j.jdiacomp.2016.07.025.
88. Hu T, Yao L, Reynolds K, et al. The effects of a low-carbohydrate diet vs. a low-fat diet on novel cardiovascular risk factors: A randomized controlled trial. *Nutrients*. 2015;7(9):7978-7994. doi:10.3390/nu7095377.
89. Hu T, Yao L, Reynolds K, et al. The effects of a low-carbohydrate diet on appetite: A randomized controlled trial. *Nutr Metab Cardiovasc Dis*. 2016;26(6):476-488. doi:10.1016/j.numecd.2015.11.011.

90. Rebholz CM, Reynolds K, Wofford MR, et al. Effect of soybean protein on novel cardiovascular disease risk factors: A randomized controlled trial. *Eur J Clin Nutr*. 2013;67(1):58-63. doi:10.1038/ejcn.2012.186.
91. Wofford MR, Rebholz CM, Reynolds K, et al. Effect of soy and milk protein supplementation on serum lipid levels: A randomized controlled trial. *Eur J Clin Nutr*. 2012;66(4):419-425. doi:10.1038/ejcn.2011.168.
92. Chen J, He J, Wildman RP, Reynolds K, Streiffer RH, Whelton PK. A randomized controlled trial of dietary fiber intake on serum lipids. *Eur J Clin Nutr*. 2006;60(1):62-68. doi:10.1038/sj.ejcn.1602268.
93. Overweight and Obesity Expert Panel. *Managing Overweight and Obesity in Adults: Systematic Evidence Review From the Obesity Expert Panel, 2013*.; 2013.
94. The Data Center. *Who Lives in New Orleans and Metro Parishes Now?*; 2017. https://www.datacenterresearch.org/data-resources/who-lives-in-new-orleans-now/.
95. Wetzels JFM, Kiemeney LALM, Swinkels DW, Willems HL, Heijer M Den. Age- and gender-specific reference values of estimated GFR in Caucasians: The Nijmegen Biomedical Study. *Kidney Int*. 2007;72(5):632-637. doi:10.1038/sj.ki.5002374.
96. Radin MS. Pitfalls in hemoglobin A1c measurement: When results may be misleading. *J Gen Intern Med*. 2014;29(2):388-394. doi:10.1007/s11606-013-2595-x.
97. Kim PS, Woods C, Georgoff P, et al. A1C underestimates glycemia in HIV infection. *Diabetes Care*. 2009;32(9):1591-1593. doi:10.2337/dc09-0177.
98. Qaseem A, Wilt TJ, Kansagara D, Horwitch C, Barry MJ, Forciea MA. Hemoglobin A1c Targets for Glycemic Control With Pharmacologic Therapy for Nonpregnant Adults With Type 2 Diabetes Mellitus: A Guidance Statement Update From the American College of Physicians. *Ann Intern Med*. 2018;168(8):569-576. doi:10.7326/M17-0939.
99. Sumithran P, Prendergast LA, Delbridge E, et al. Ketosis and appetite-mediating nutrients and hormones after weight loss. *Eur J Clin Nutr*. 2013;67(7):759-764. doi:10.1038/ejcn.2013.90.
100. Gardner CD, Trepanowski JF, Gobbo LCD, et al. Effect of low-fat VS low-carbohydrate diet on 12-month weight loss in overweight adults and the association with genotype pattern or insulin secretion the DIETFITS randomized clinical trial. *JAMA - J Am Med Assoc*. 2018;319(7):667-679. doi:10.1001/jama.2018.0245.
101. Brenner BM, Meyer TW, Hostetter TH. Dietary protein intake and the progressive nature of kidney disease: the role of hemodynamically mediated glomerular injury in the pathogenesis of progressive glomerular sclerosis in aging, renal ablation, and intrinsic renal disease. *N Engl J Med*. 1982;307(11):652-659. doi:10.1056/NEJM198209093071104.
102. Sato J, Kanazawa A, Makita S, et al. A randomized controlled trial of 130 g/day low-carbohydrate diet in type 2 diabetes with poor glycemic control. *Clin Nutr*. 2017;36(4):992-1000. doi:10.1016/j.clnu.2016.07.003.
103. Yancy WS, Foy M, Chalecki AM, et al. A low-carbohydrate, ketogenic diet to treat type 2 diabetes. *Nutr Metab (Lond)*. 2005;2(1):34. doi:10.1186/1743-7075-2-34.
104. Boden G, Sargrad K, Homko C, Mozzoli M, Stein TP. Effect of a low-carbohydrate diet on appetite, blood glucose levels, and insulin resistance in obese patients with type 2 diabetes. *Ann Intern Med*. 2005;142(6). doi:10.1016/S0084-3741(08)70336-6.
105. Stern L, Iqbal N, Seshadri P. The Effects of Low-Carbohydrate versus Conventional Weight Loss Diets in Severely Obese Adults : One-Year Follow-up of a randomized control trial. *Ann Intern …*. 2004;140(May 2001):769-777.

doi:10.1016/j.accreview.2004.07.103.

106. Westman EC, Yancy WS, Edman JS, Tomlin KF, Perkins CE. Effect of 6-month adherence to a very low carbohydrate diet program. *Am J Med*. 2002;113(1):30-36. doi:10.1016/S0002-9343(02)01129-4.

107. Moosheer SM, Waldschütz W, Itariu BK, Brath H, Stulnig TM. A protein-enriched low glycemic index diet with omega-3 polyunsaturated fatty acid supplementation exerts beneficial effects on metabolic control in type 2 diabetes. *Prim Care Diabetes*. 2014;8(4):308-314. doi:10.1016/j.pcd.2014.02.004.

108. Aagaard K, Petrosino J, Keitel W, et al. The Human Microbiome Project strategy for comprehensive sampling of the human microbiome and why it matters. *FASEB J*. 2013;27(3):1012-1022. doi:10.1096/fj.12-220806.

109. McInnes P, Cutting M. Manual of Procedures for Human Microbiome Project. http://www.hmpdacc.org/doc/HMP_MOP_Version12_0_072910.pdf.

110. Marrero DG, Palmer KNB, Phillips EO, Miller-Kovach K, Foster GD, Saha CK. Comparison of commercial and self-initiated weight loss programs in people with prediabetes: A randomized control trial. *Am J Public Health*. 2016;106(5):949-956. doi:10.2105/AJPH.2015.303035.

111. Barengolts E, Manickam B, Eisenberg Y, Akbar A, Kukreja S, Ciubotaru I. EFFECT OF HIGH-DOSE VITAMIN D REPLETION ON GLYCEMIC CONTROL IN AFRICAN-AMERICAN MALES WITH PREDIABETES AND HYPOVITAMINOSIS D. *Endocr Pract*. 2015. doi:10.4158/EP14548.OR.

112. Block G, Azar KMJ, Romanelli RJ, et al. Diabetes prevention and weight loss with a fully automated behavioral intervention by email, web, and mobile phone: A randomized controlled trial among persons with prediabetes. *J Med Internet Res*. 2015. doi:10.2196/jmir.4897.

113. Kramer MK, Molenaar DM, Arena VC, et al. Improving employee health: Evaluation of a worksite lifestyle change program to decrease risk factors for diabetes and cardiovascular disease. *J Occup Environ Med*. 2015. doi:10.1097/JOM.0000000000000350.

114. Parker AR, Byham-Gray L, Denmark R, Winkle PJ. The Effect of Medical Nutrition Therapy by a Registered Dietitian Nutritionist in Patients with Prediabetes Participating in a Randomized Controlled Clinical Research Trial. *J Acad Nutr Diet*. 2014;114(11):1739-1748. doi:10.1016/j.jand.2014.07.020.

115. Maruthur NM, Ma Y, Delahanty LM, et al. Early response to preventive strategies in the diabetes prevention program. *J Gen Intern Med*. 2013;28(12):1629-1636. doi:10.1007/s11606-013-2548-4.

116. Leong A, Daya N, Porneala B, et al. Prediction of Type 2 Diabetes by Hemoglobin A1c in Two Community-Based Cohorts. *Diabetes Care*. 2017;41(1):dc170607. doi:10.2337/dc17-0607.

117. Khaw K-T, Wareham N, Bingham S, Luben R, Welch A, Day N. Association of hemoglobin A1c with cardiovascular disease and mortality in adults: the European prospective investigation into cancer in Norfolk. *Ann Intern Med*. 2004;141(6):413-420. doi:10.7326/0003-4819-141-6-200409210-00006.

118. Cole RE, Boyer KM, Spanbauer SM, Sprague D, Bingham M. Effectiveness of Prediabetes Nutrition Shared Medical Appointments. *Diabetes Educ*. 2013;39(3):344-353. doi:10.1177/0145721713484812.

Low-Carbohydrate Dietary Pattern on Glycemic Outcomes Trial

PROTOCOL

PROTOCOL VERSION 2.2

Dated: September 14, 2020

New Orleans, LA

Table of Contents

1. Overview 5
2. Background and Significance 6
2.A. Type 2 Diabetes Mellitus is a major global public health challenge 6
2.B. Prediabetes is a major risk factor for cardiometabolic diseases 6
2.C. Role of lifestyle in preventing diabetes........ 6
2.D. Low-to-moderate carbohydrate diets, weight loss, and cardiometabolic diseases 7
2.E. Mediterranean-style diet is associated with reduced risk of T2D and improved glycemic control 8
2.F. Evidence for low-carbohydrate diets that emphasize Mediterranean-style dietary components........ 8
2.G. Evidence for specific food groups and risk of T2D........ 8
2.H. Gut microbiota, diet and T2D 9
2.I. Continuous Glucose Monitoring 9
2.I. Clinical and public health significance of the proposed study 9
2.J. Innovation........ 10
3. Preliminary Studies........ 10
3.A. Summary of aggregate experience of the research team 10
3.B. Low-carbohydrate behavioral dietary intervention trial experience........ 11
3.C. Other dietary intervention trial experience 11
4. Study Design and Organization........ 12
4.A. Overview of trial design........ 12
4.B. Study outcomes........ 13
4.B.1. Primary outcome 13
4.B.2. Secondary outcomes 13
4.C Study Timeline........ 13
6. Study Participants........ 13
6.A. Eligibility criteria........ 13
7. Recruitment and Randomization 14
7.A. Recruitment........ 14
7.A.1. Mass mailing, placement of brochures/flyers in community, TV/radio/billboard advertisements 15
7.A.2. Database searches and targeted mailing........ 15
7.A.3. Referrals and Local Partnerships 15
7.B. Randomization........ 15

7.C. Masking 15
7.D. Safety Considerations 16
8. Dietary Intervention Program 16
8.A. Low-carbohydrate dietary intervention group 16
8.B. Usual Diet Group 17
8.C. Physical Activity 17
8.D. Dietary Adherence 17
9. Safety of Dietary Intervention 18
10. Data Collection 18
10.A. Study visits 18
10.A.1. Screening/baseline visits 19
10.A.2. Follow-up visits and final visit 20
10.A.3. CGM 6-month visit 20
10.B Study measurements 21
10.B.1. Questionnaires 21
10.B.2. Dietary measurements 21
10.B.3. Assessment and management of adverse effects 21
10.B.4. BP and anthropometric measures 21
10.B.5. Blood samples 21
10.B.6. Urine collection 22
10.B.7. Stool Specimen Collection 22
10.B.8. CGM at 6 months: 23
11. Quality Assurance and Quality Control 23
11.A. Manual of procedures 23
11.B. Training and certification 23
12. Sample Size and Statistical Power 23
13. Data Management and Analysis 24
13.A. Data management 24
13.B. Data analysis plan 24
13.B.1. Exploratory data analysis 24
13.B.2. Primary, secondary, and exploratory outcomes 24
15. Anticipated Challenges 25
16. Protection of Human Subjects 25

16.A. Protection of human subjects from research risks 25
16.A.1. Risks to the subjects 26
16.A.2. Adequacy of protection against risks 27
16.A.3. Potential benefits of the proposed research to human subjects and others ... 28
16.A.4. Importance of knowledge to be gained 28
16.A.5. Remuneration 29
16.B. Data safety and monitoring plan 29
Bibliography and References Cited 31

1. Overview

The increasing prevalence of type 2 diabetes mellitus (T2D), a major cause of morbidity and mortality worldwide, is a critical public health concern. In the United States, an estimated 33.9% of adults have prediabetes (impaired fasting glucose, elevated hemoglobin A1c (HbA1c), or impaired glucose tolerance). Individuals with prediabetes are at elevated risk of T2D and cardiovascular disease (CVD), with 5–10% developing clinical T2D each year.

In the short-term, among patients with T2D, low-to-moderate carbohydrate diets (energy percentage below 45) have a greater glucose-lowering effect than do high-carbohydrate diets, with larger glucose-lowering effect the greater the carbohydrate reduction. Additionally, compared with low-fat diets, Mediterranean diets that are high in unsaturated fats (particularly *cis*-monounsaturated fats) reduce risk of T2D and CVD and improve glycemic control among diabetics. However, compared with usual diets, the effect of a behavioral intervention promoting a low-carbohydrate/high-unsaturated fat and protein dietary pattern that emphasizes consumption of non-starchy vegetables, plant-based oils, fish, poultry, and mixed nuts on glucose homeostasis, and other metabolic risk factors among individuals with prediabetes or early-stage untreated T2D is not well understood.

Our *long-term goal* is to develop and implement dietary intervention(s) that reduce risk of T2D. The *overall goal of this randomized controlled trial is to study the effect of a behavioral intervention promoting a low-carbohydrate/high-unsaturated fat and high-protein dietary pattern (initial target <40 g digestible carbohydrates, final target <60 g digestible carbohydrates) compared with a usual diet on metabolic risk factors among individuals with prediabetes or early-stage untreated diabetes* (HbA1c 6.0–6.9%). Specifically, we will test the following hypotheses:

Primary hypothesis: Compared with usual diet, over a 6-month period, a healthy low-carbohydrate/high unsaturated fat and high-protein dietary pattern will lead to *a larger decrease of HbA1c, by at least 0.15%.*

Secondary hypothesis: Over a 6-month period, a healthy low-carbohydrate/high-unsaturated fat and high-protein dietary pattern will lead to *larger improvements in the following major secondary outcomes*: fasting glucose, systolic blood pressure (BP), total-to-HDL-cholesterol ratio, and body weight.

Exploratory outcomes: We will also study the effect of the intervention on the 6-month change in: insulin and homeostasis model assessment of insulin resistance (HOMA-IR); diastolic BP; waist circumference; and estimated CVD risk.

To achieve these study aims, we will recruit 150 adults with prediabetes or untreated diabetes (HbA1c 6.0–6.9%) in the Greater New Orleans, LA area. Using a stratified, blocked randomization scheme, participants will be assigned to either a behavioral modification intervention aimed at promoting a healthy low-carbohydrate dietary pattern or to usual diet. The intervention group will have an intensive phase for 3 months, followed by a maintenance phase in months 4 through 6. The *primary outcome will be difference between the active intervention and control groups in the change in HbA1c from baseline to 6 months of follow-up. We expect 80% power to detect a difference of at least 0.15% in change of HbA1c.* Changes in other metabolic risk factors will be studied concurrently. Metabolic risk factors will be assessed at baseline, 3 months, and 6 months of follow-up.

There is much interest regarding dietary approaches to prevent T2D and its complications. The results from this study will help to determine whether, compared with usual diet, a behavioral intervention aimed at promoting a healthy low-carbohydrate dietary pattern improves metabolic risk factors.

2. Background and Significance

2.A. Type 2 Diabetes Mellitus is a major global public health challenge

Diabetes is a strong and independent risk factor for CVD morbidity and mortality. A meta-analysis of 102 prospective studies (698,782 participants) estimated diabetes leads to an approximately 2-fold increased risk in vascular diseases, including coronary heart disease (CHD) and major stroke subtypes, and deaths due to other vascular causes.[1] Among participants with diabetes, CHD risk is about 50% higher among those with fasting blood glucose ≥7 mmol/L compared with those with fasting blood glucose <7 mmol/L.[1] In addition to increasing CVD risk, diabetes can cause diabetic retinopathy,[2] chronic kidney disease,[3] lower limb amputation,[4] and other complications. Most individuals with diabetes have T2D.[5] The US Centers for Disease Control and Prevention (CDC) estimated that 30.2 million adults ≥18 years (12.2%) had diabetes in 2015, with 7.2 million (23.8%) not being aware of or reporting diabetes.[5] Diagnosed diabetes prevalence among US adults has increased substantially over time, from 2.54% in 1980 to 7.40% in 2015.[6] Diabetes prevalence is also high globally, with an estimated 2014 age-standardized prevalence of 9.0% in men and 7.9% in women.[7] Diabetes accounts for an increasing portion of US healthcare expenditures. Total cost of diagnosed diabetes in the US was estimated to be $245 billion in 2012, 41% higher than in 2007.[8] Diabetes is associated with much higher lifetime medical expenses, with higher expenses for those diagnosed at younger ages.[9]

2.B. Prediabetes is a major risk factor for cardiometabolic diseases

Prediabetes, defined by the American Diabetes Association (ADA) as impaired fasting glucose (IFG), impaired glucose tolerance (IGT), or elevated HbA1c, is also referred to as “intermediate hyperglycemia”[10] and “high-risk state of developing diabetes”.[11] The ADA defines prediabetes as having: fasting plasma glucose of 100–125 mg/dL (IFG); 2-hour post-load glucose in the 75-g oral glucose tolerance test (OGTT) of 140–199 mg/dL (IGT); or HbA1c 5.7–6.4%.[12] Prediabetes itself is not a disease; for all three tests, “risk is continuous, extending below the lower limit of the range and becoming disproportionately greater at the higher end of the range”.[12] The CDC estimated that in 2015, 33.9% of US adults had prediabetes based on fasting glucose or HbA1c.[5] Among those with prediabetes, only 11.9% reported having being told this by a medical professional.[5] Approximately 5–10% of individuals with prediabetes develop diabetes within one year, depending on how prediabetes is defined.[13] In a meta-analysis, annual diabetes incidence was 15–19% among individuals with both IFG and IGT, while it was 4–6% in those with isolated IFG and 6–9% in those with isolated IGT.[13]

A recent meta-analysis (1,611,339 participants from 53 studies) reported that prediabetes as defined by the ADA is associated with higher risk of composite CVD events and CHD.[14] IFG and IGT were also positively associated with increased risk of stroke and all-cause mortality.[14] Prediabetes is linked with increased risk of other conditions typically thought of as diabetes complications, such as neuropathies and nephropathies.[15] Importantly, it is possible for individuals with prediabetes to revert to normoglycemia, as observed in observational studies[13] and lifestyle or drug intervention trials.[16,17] In the Diabetes Prevention Program (DPP), reversion to normoglycemia was associated with lower long-term T2D risk[18] and lower predicted CVD risk.[19]

2.C. Role of lifestyle in preventing diabetes

Much evidence from observational studies and clinical trials supports the critical role of diet and other lifestyle factors in preventing T2D. Globally, lifestyle transitions towards reduced physical activity, increased consumption of refined carbohydrates, coupled with resulting changes in body composition, has led to increases in diabetes prevalence and severity.[20] Body

mass index (BMI), waist circumference, and waist-to-height ratio are predictors of T2D.[21] The DPP, which combined calorie restriction and exercise to promote weight loss, substantially reduced risk of T2D (by 58%) among individuals with elevated FPG and IGT;[22] a benefit that persisted in 10-year follow-up analyses.[16] Other trials of lifestyle modification focused on diet and exercise have also shown benefits in study populations in China,[23] Finland,[24] and India.[25]

2.D. Low-to-moderate carbohydrate diets, weight loss, and cardiometabolic diseases

Low-to-moderate carbohydrate diets (<45% of energy from carbohydrates) are popular dietary approaches for losing weight. Weight loss is a key component of the DPP lifestyle intervention[26] and ADA lifestyle recommendations.[12,27] Meta-analyses from our group[28] and others support that low-to-moderate carbohydrate diets are at least as effective as low-fat diets at promoting weight loss. For instance, a meta-analysis reported a 2-kg (95% CI, 3.1–0.9) larger weight loss in low-carbohydrate diet (<120 g carbohydrates/day) arms compared with low-fat diet (<30% energy from fat) arms.[29] Estimated 10-year CVD risk was lower in low-carbohydrate than low-fat arms.[29] This aligns with research published by our team; in a 12-month trial of participants with obesity, a low-carbohydrate diet (target <40 g carbohydrates/day) was more effective than a low-fat diet (target <30% energy from fat, <7% saturated fat) for weight loss and CVD risk factor reduction.[30]

Low-to-moderate carbohydrate diets may also be beneficial among patients with T2D. Low-carbohydrate diets have come in and out of favor as treatment of diabetes.[31] In a meta-analysis (1376 patients;10 trials), after 3 or 6 months of the intervention, low-to-moderate carbohydrate diets (<45% energy from carbohydrates) led to a 0.34% lower HbA1c (95% CI, 0.06–0.63%) compared with high-carbohydrate diets.[32] Compared with high-carbohydrate diets, there was a greater reduction in HbA1c the greater the reported carbohydrate restriction.[32] There was no benefit of the low-to-moderate carbohydrate diets on HbA1c at 12 months or longer, and no difference between interventions on BMI, body weight, low-density lipoprotein (LDL) cholesterol or quality of life.[32] However, results may have been affected by dietary adherence or glucose medication. There was evidence of diabetes medication reduction in the low-carbohydrate arms in some studies,[33–37] with evidence of medication reduction and improved blood glucose stability persisting for up to 2 years.[38] Hypoglycemia (though not after medication adjustment) was observed in three subjects taking insulin or a sulfonylurea out of twelve subjects in the low-carbohydrate arm of another study.[39] In a subset of participants with T2D (n=46) from a large weight loss trial, compared with those randomized to a low-fat diet and orlistat, those randomized to a low-carbohydrate diet for 48 weeks had improved HbA1c (0.8% lower, 95% CI, -1.5, -0.02%) and a reduction in antiglycemic medications, despite similar weight loss effects of the interventions.[40]

Though low-to-moderate carbohydrate diets have been studied among overweight and obese participants (primarily focused on weight loss), less is understood about the effects of such diets on glycemic outcomes specifically among individuals with prediabetes. A study among participants with IGT observed a reduction at 12 months in weight, HbA1c, and other glycemic outcomes among individuals who participated in a 7-day in-hospital education program, though this was not randomized.[41] A randomized pilot trial of overweight or obese individuals with T2D or prediabetes found that, over 3 months and at 12 months, a very low-carbohydrate diet improved HbA1c, led to higher medication discontinuation, and more weight loss compared with a low-fat diet.[34,37] However, only 4 individuals had prediabetes. Given benefits of low-to-moderate carbohydrate diets for weight loss in general populations and for glycemic control among patients with T2D, further study of these diets are warranted among individuals with prediabetes who are at elevated cardiometabolic disease risk.

2.E. Mediterranean-style diet is associated with reduced risk of T2D and improved glycemic control

Though there is no one "Mediterranean diet", the Mediterranean dietary pattern tends to have the following characteristics: high consumption of vegetables, fruits, breads and cereals, legumes, nuts and seeds; low-to-moderate consumption of dairy products, fish, poultry and eggs; little consumption of red meat and sweets; and red wine in moderation.[42] Olive oil is an important source of fat. In many observational studies, adherence to the Mediterranean-style dietary pattern is associated with reduced risk of CVD[43] and T2D.[44,45] Evidence from randomized trials also supports the benefits of this dietary pattern. In several trials, participants randomized to Mediterranean diets had substantially lower risk of CVD than those randomized to low-fat control diets.[46,47] In the three-arm randomized PREDIMED trial of participants at high risk of CVD, after 4.1 years of follow-up, those randomized to a traditional Mediterranean diet supplemented with either olive oil or nuts had a 30% reduced risk of T2D compared to those in the low-fat control diet group.[48] In all PREDIMED arms, average carbohydrate intake was relatively low (39.7–43.7% at end of trial).[47] Two trials have found a higher rate of remission from metabolic syndrome among individuals randomized to a Mediterranean diet compared with those randomized to control diets (prudent or low-fat) during 2–5 years of follow-up.[49,50] The Mediterranean pattern may also be beneficial among individuals with T2D. Meta-analyses have reported that, among patients with T2D, those randomized to a Mediterranean diet had lower HbA1c levels and CVD risk factors, and more significant weight loss, compared with those randomized to control diets.[51]

2.F. Evidence for low-carbohydrate diets that emphasize Mediterranean-style dietary components

Many low-carbohydrate diets involve increased consumption of food items, such as red meat,[52] that are associated with increased cardiometabolic disease risk. However, low-carbohydrate dietary approaches can focus on substituting carbohydrates with foods common in Mediterranean-style diets, such as olive oil and nuts, which may improve cardiometabolic health. In a 4-year Italian study of individuals with newly diagnosed T2D, those randomized to a low-carbohydrate Mediterranean diet (<50% carbohydrates; consumed ~44%) had more favorable changes in glycemic control, higher rates of T2D remission, and delayed need for antiglycemic drugs compared with participants randomized to a low-fat diet (<30% fat, <10% saturated fat).[53,54] A 12-month Israeli study assessed low-carbohydrate Mediterranean (35% carbohydrates), traditional Mediterranean, and ADA (both 50–55% carbohydrates) diets in 259 overweight participants with T2D.[55] Most CVD risk factors improved in all groups; there was only HDL improvement in the low-carbohydrate Mediterranean diet and this diet was superior to the other diets in improving glycemic control.[55] In a 2-year weight-loss study of moderately obese participants, low-carbohydrate and Mediterranean diets were superior to low-fat diet for weight loss.[56] Participants in the low-carbohydrate arm were counseled to choose vegetarian sources of fat and protein and to avoid trans fat. At 6 years after study commencement, despite weight regain, there were still long-term benefits, particularly for the low-carbohydrate and Mediterranean diets.[57] In whole, evidence supports that low-carbohydrate Mediterranean-style diets are feasible and may have metabolic benefits.

2.G. Evidence for specific food groups and risk of T2D

Prospective cohort studies have found consumption of specific foods and beverages is associated with diabetes risk. Meta-analyses report higher consumption of processed and unprocessed red meat,[58] white rice (particularly in Asian populations),[59] and sugar-sweetened beverages[60,61] are associated with higher T2D risk. Consumption of green leafy vegetables,[62,63] yogurt,[64] whole grains,[65] decaffeinated and total coffee,[66] and light-to-moderate alcohol

consumption[67] are associated with reduced risk. Consumption of fruits, particularly blueberries, grapes, and apples, are associated with reduced risk; fruit juice is associated with higher risk.[68]

2.H. Gut microbiota, diet and T2D

There is increasing evidence for the importance of gut microbiota in risk of type 2 diabetes and other cardiometabolic diseases.[69] Additionally, dietary modifications, including changes in carbohydrate consumption, can lead to substantial changes in gut microbiome over short periods of time.[70,71] Less is known about longer-term changes to the gut microbiota during the adoption of a low-carbohydrate diet and how these changes may influence cardiometabolic risk. We plan to collect stool specimens at baseline, 3 months, and at 6 months, for potential future studies on how this intervention affects the gut microbiome.

2.I. Continuous Glucose Monitoring

As HbA1c is a marker of weighted average glucose over the past 8 to 12 weeks, it does not explicitly capture short-term glycemic excursions or variability. It has been theorized that such fluctuations may have an independent role in the development of diabetes complications.[72] Though more evidence is needed, research has found relationships of higher glycemic variability with coronary artery disease (CAD) and microvascular outcomes in type 2 diabetes (T2D).[73,74] This suggests that improved control of extreme glucose excursions, particularly postprandially, may help to reduce risks of such complications.[74]

In one study of a low-calorie, low-carbohydrate diet compared with a low-calorie, low-fat behavioral dietary intervention among individuals with T2D, there was evidence that some markers of glycemic variability were further reduced in the low-carbohydrate arm compared with the low-fat arm (assessed by 48-hour continuous glucose monitoring) at 6 months, 1 year, and 2 years.[35,38,75] However, there is not prior work assessing how a behavioral intervention aimed at reducing carbohydrate consumption affects mean glucose, glucose exposure above the set-point of 100 mg/dL, glucose excursions, or glycemic variability as measured by continuous glucose monitoring among individuals with untreated T2D or at high risk of T2D.

Given this background, we will use continuous glucose monitors (CGM) to collect data for up to 14 days on glucose levels at the end of the 6-months study and will compare mean 24-h glucose among those in the intervention group compared with mean 24-h glucose among those in the control group. We will also measure other markers of glycemic exposure, such as glycemic variability (mean amplitude of glycemic excursions (MAGE), standard deviation of glucose, coefficient of variation) and mean 24-hour glucose during wake-time (6:00 AM to 12:00 AM), and during sleep-time (12:00 AM to 6:00 AM).

2.I. Clinical and public health significance of the proposed study

Prediabetes, or intermediate hyperglycemia, is a marker of high risk of developing T2D and other complications. Due to increasing prevalence of T2D and associated morbidity and mortality globally, a better understanding of dietary approaches to delay progression to T2D or to promote reversion to normoglycemia is a high public health priority. This study proposes to assess the effect of a behavioral intervention promoting a low-carbohydrate/high-unsaturated fat dietary pattern (initial target <40 g digestible carbohydrates, final target <60 g digestible carbohydrates) on HbA1c, a marker of glycemic control, and other metabolic risk factors. Building from prior findings from clinical and observational studies, we will develop a dietary intervention that promotes the consumption of non-starchy vegetables, plant-based oils, fish, poultry, and mixed nuts, that are thought to reduced risk of T2D or CVD. Participants assigned

to the intervention will be trained in behavioral techniques that will provide capacity to maintain dietary changes long after study completion. Our outcome measures will assess the effect of this dietary intervention in a “real-life” setting as opposed to dietary modifications achieved in metabolic ward studies or through food prepared in research kitchens. Findings from this study may provide evidence in support of promoting alternative dietary styles among individuals with high T2D and CVD risk and could potentially be used to inform dietary interventions used in programs like DPP.

2.J. Innovation

The status quo as it pertains to dietary approaches for reducing risk of T2D is focused on reducing caloric intake and total fat intake. However, recent evidence suggests that the types of fat, rather than the quantity of fats may be more important, with evidence supporting that a Mediterranean-style diet that does not restrict fat intake reduces risk of developing both T2D[48] and CVD[47]. Moreover, in the short-term, low-carbohydrate diets, which are popular weight loss diets, have a greater glucose-lowering effect than do high-carbohydrate diets among patients with T2D.[32] However, the effect of low-carbohydrate diets that emphasize consumption of non-starchy vegetables, plant-based oils, fish, poultry, and mixed nuts on T2D prevention is unclear, particularly compared with higher-carbohydrate diets more commonly consumed in the US. *The proposed research is innovative, in our opinion, because it represents a substantial departure from the status quo by assessing the combined effect of a low-carbohydrate, high unsaturated fat and protein diet on cardiometabolic risk factors among adults with prediabetes or early-stage untreated diabetes.* We expect the results from this study have the potential to lead to new horizons for developing and implementing dietary approaches (other than the most commonly used reduced fat diet) that will substantially reduce risk of cardiometabolic disease among adults with T2D or at high risk of T2D. The proposed intervention dietary approach promotes consumption of dietary components thought to reduce risk of cardiometabolic disease and the behavior change intervention has expected applicability in clinical practice.

3. Preliminary Studies

3.A. Summary of aggregate experience of the research team

Dr. Kirsten Dorans is trained in cardiovascular disease epidemiology, with strong training in epidemiologic methods and biostatistics. Her publication record includes studies of behavioral risk factors and risk of chronic disease. She has published on the relationships between dietary intake and chronic heart failure[76,77] and on physical activity and incident multiple sclerosis[78]. She served as lead author, conducting data management, analysis, drafting, and revision of two of these manuscripts. In graduate school, she focused on studying links between outdoor air pollution and chronic diseases and has published several papers in this field. The COBRE program will provide an excellent opportunity for the JFI to transition from air pollution research to clinical and translational research focused on nutrition and diabetes. Building off of her training in observational epidemiology, this program will involve training and practical experience that will allow her to broaden her expertise and to develop practical skills for carrying out clinical research.

The mentorship team has much experience and prior successful collaborations in key areas related to this study. Dr. Jiang He is an internationally renowned expert in cardiometabolic disease clinical and epidemiological research. He has served as PI for many epidemiological studies and clinical trials, including clinical and observational studies focused on dietary consumption and chronic diseases[79–84] and has conducted extensive global research on diabetes.[85,86] Dr. Bazzano has collaborated extensively with Dr. He for more than 15 years in research related to diet and chronic disease. She is a clinical investigator and has expertise in

nutritional epidemiology and clinical research. Dr. Bazzano was the PI of a highly successful clinical trial comparing the effects of low-carbohydrate and low-fat diets on weight and cardiovascular risk factors among individuals with obesity, which was a subproject within the Tulane COBRE in Hypertension and Renal Biology.[30] Dr. He served as Dr. Bazzano's mentor for this clinical trial. They have collaborated on much other work related to dietary and chronic disease, including a meta-analysis on low-carbohydrate diets.[28] Dr. Bazzano has also published many articles on diet and risk of diabetes[87,88] and on diabetes in the Bogalusa Heart Study.[89–91]

3.B. Low-carbohydrate behavioral dietary intervention trial experience

In the study on macronutrient composition of diet and risk factors for cardiovascular disease (MACRO) study, 148 individuals without clinical cardiovascular disease or diabetes and with obesity were randomly assigned to a low-carbohydrate or a low-fat behavioral dietary intervention for 12 months. In the low-carbohydrate arm, daily dietary intake of carbohydrates decreased from an average of 242 g at baseline to 97 g, 93 g, and 127 g at 3, 6, and 12 months, respectively. At 12 months, participants in the low-carbohydrate intervention group had greater decreases in weight (-3.5 kg; 95% CI, -5.6 to -1.4 kg) and fat mass (-1.5%; 95% CI, -2.6% to -0.4%) and greater improvement in several cardiovascular risk factors.[30] This suggests that restricting carbohydrates may be an option for weight loss and reduction of cardiovascular risk factors. Further analyses found that the low-carbohydrate dietary intervention led to similar or greater improvement in inflammation, adipocyte dysfunction, and endothelial dysfunction compared with the low-fat dietary intervention[92] and that the low-fat diet reduced peptide YY more than the low-carbohydrate diet, suggesting satiety may be better preserved on low-carbohydrate diets.[93] No serious adverse events were reported in this study and the number of participants with symptoms were similar between the two diets, with the exception of more participants having headaches on the low-fat diet at 3 months (18 vs 6 participants, $p = 0.03$).[30] Dr. Bazzano served as PI for this study and Dr. He served as her mentor. Dr. Bazzano mentored one of her PhD students in conducting analyses and writing and editing manuscripts related to this project.

3.C. Other dietary intervention trial experience

Dr. He has also led or been involved with many dietary intervention trials, including both behavioral change and feeding study interventions. In the Protein and Blood Pressure (ProBP) study, 352 adults with prehypertension or stage 1 hypertension in New Orleans, LA and Jackson, MS were assigned to take, in a random order, 40 g/d soy protein, milk protein, or carbohydrate supplementation each for 8 weeks. Compared with the high-glycemic index refined carbohydrate, both soy protein and milk protein supplementation were associated with a -2.0 mm Hg (95% CI -3.2 to -0.7 mm Hg) and -2.3 mm Hg (-3.7 to -1.0 mm Hg) net change in systolic blood pressure.[84] These findings suggest that partial replacement of carbohydrate with soy or milk protein might be an important part of nutrition intervention strategies to prevent and treat hypertension. Additionally, soy protein supplementation led to reduction in plasma E-selectin compared with carbohydrate and milk protein and in plasma leptin compared with carbohydrate.[94] Soy protein also led to improvement in lipid profile compared with carbohydrate and milk protein.[95] Dr. He was PI for this project.

In the Fiber Intervention on Blood Pressure (FIBRE) study, 110 participants ages 30–65 years with untreated pre-hypertension or stage-1 hypertension and serum cholesterol level < 240 mg/dl were randomly assigned to 8-g/day of water soluble fiber from oat bran or a control intervention (double-blind RCT). Over the 12-week study period, net changes in systolic and diastolic blood pressure were -1.8 mm Hg (-4.3 to 0.8) and -1.2 mm Hg (-3.0 to 0.5 mm Hg), respectively.[82] Net changes in total, HDL-, and LDL-cholesterol were -2.40 mg/dL (-10.60 to

5.81 mg/d:), -1.66 mg/dL (-4.55 to 1.22 mg/dL), and -1.33 mg/dl (-8.33 to 5.68 mg/dL), respectively.[96] Dr He was PI for this study.

Dr. He is currently serving as a mentor for Dr. Katherine Mills' COBRE project (Effect of Dietary Sodium Reduction in Kidney Disease Patients with Albuminuria; SUPER). In this project, participants with chronic kidney disease and albuminuria are being randomized to a behavioral modification program designed to reduce dietary sodium intake to ≤2,300 mg/day or to usual care and test the effect of this intervention on change in albumin-to-creatinine ratio from baseline to 24-weeks.

4. Study Design and Organization

4.A. Overview of trial design

We propose to conduct a *randomized controlled trial* aimed at testing the effect of a behavioral intervention that promotes *reduced carbohydrate intake and increased consumption of heart-healthy unsaturated fats and protein* in patients with HbA1c 6.0–6.9% (*Figure 1*). We plan to recruit 150 individuals with elevated HbA1c and randomly assign them to a moderately intensive intervention on diet pattern,[97] using well-established methods for behavioral modification, or to usual diet. We will compare the difference between the active intervention and control groups for change in HbA1c from baseline to 6 months of follow-up (primary outcome). We will assess four major secondary outcomes: fasting plasma glucose, systolic BP, total-to-HDL cholesterol ratio, and body weight. In addition, we will look at several other exploratory outcomes: insulin and HOMA-IR, diastolic BP, waist circumference, and estimated CVD risk, as estimated by the 2013 ACC/AHA risk equation. The main trial components are summarized in *Table 1*.

Figure 1. Study Design

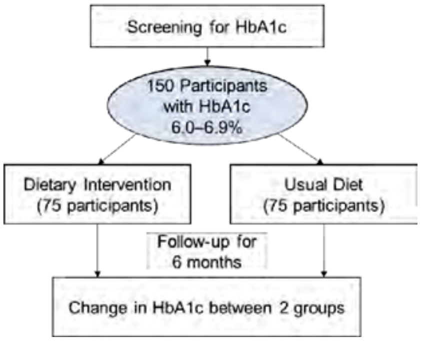

Table 1. Study Overview

Study Design	Randomized controlled trial
Study Participants	150 participants with elevated HbA1c (6.0–6.9%)
Intervention Group (n=75)	Behavioral modification to reduce carbohydrate consumption and promote consumption of foods such as non-starchy vegetables, plant-based unsaturated oils, nuts and seeds, avocados, eggs, seafood, poultry, lean meat and tofu. We will recommend moderate consumption of cheese, unsweetened Greek yogurt, and low-carb milk. If consuming higher-carbohydrate items, recommend legumes, fruits, (limited) whole grains. Target <40 g net carbohydrates per day for first 3 months and will have a goal of <40 net g carbohydrates per day; <60 g net carbohydrates per day for months 4 onwards.
Usual Diet Group (n=75)	No dietary intervention. To encourage continued participation in the trial, we will offer optional monthly educational sessions on unrelated topics, such as retirement planning or health insurance
Outcome Measures	Primary: HbA1c; Secondary: fasting plasma glucose, systolic BP, total-to-HDL-cholesterol ratio, and body weight

4.B. Study outcomes

4.B.1. Primary outcome: The *primary outcome will be the difference between the intervention group and the usual diet group for change in HbA1c* from baseline to 6 months. Changes in HbA1c will be calculated from blood samples taken at baseline and 6 months.
4.B.2. Secondary outcomes: Secondary outcomes will include changes from baseline to 6 months of *fasting plasma glucose, systolic BP, total-to-HDL cholesterol ratio, and body weight.,*
4.B.3. Other outcomes: We will also study the effect of the intervention on the following outcomes: insulin and homeostasis model assessment of insulin resistance (HOMA-IR); diastolic BP; waist circumference; total-to-HDL-cholesterol ratio; and estimated CVD risk.

4.C Study Timeline

Table 2 shows the timeline, which will be followed in the implementation of study-related activities, such as publications and submission of an R01 grant application.

Table 2. Study Timeline

	Year 1		Year 2		Year 3	
Develop MOP, intervention program, study forms, and pilot testing	X	X				
Certification and training of staff	X					
Recruitment		X	X			
Intervention and data collection, measurement, entry, cleaning		X	X	X	X	
Data quality control and analysis						X
Publications	X	X	X	X	X	X
R01 grant submissions, resubmissions				X	X	X

The first 6 months will focus on development of manual of operations (MOP), intervention program, study forms, study protocol, Institutional Review Board (IRB) approval, and pilot testing of the intervention program. It will also involve staff training and certification. During the last half of year 1, recruitment, intervention, and data collection will commence. During the last 3 months of year 3, data quality control and analysis will be carried out. Throughout the study period, the JFI will publish peer-reviewed manuscripts from ongoing Tulane University research projects.

6. Study Participants

6.A. Eligibility criteria

Inclusion criteria

- Men or women ages 40 to 70 years: aim to recruit 50% women
- Any race or ethnicity: aim to recruit 40% ethnic minorities (i.e., African American and Hispanic), close to population in the greater New Orleans area[98]
- HbA1c 6.0–6.9%
- Willing and able to provide informed consent

Exclusion criteria

- Diagnosed type 1 diabetes mellitus based on patient self-report
- Use of agents affecting glycemic control (medications for diabetes, oral glucocorticoids) within the past three months prior to enrollment based on patient self-report
- Medical condition in which low-carbohydrate diet may not be advised
    - Estimated glomerular filtration rate (eGFR) ≤45 mL/min/1.73 m^2, which is close to the 5th percentile of eGFR among non-diseased individuals of 70 years of age[99]

- Self-report of liver disease due to hepatitis or alcohol; osteoporosis; untreated thyroid disease; gout; or cancer (other than non-melanoma skin cancer) requiring treatment in the past year, unless prognosis is excellent

- Factors that may affect HbA1c:
  - Hemoglobin <11 mg/dL (cutpoint for moderate-to-severe anemia, which could lead to falsely elevated or lowered HbA1c)[100]
  - Recent blood donation or blood transfusion (self-report, past 4 months)
  - Human Immunodeficiency Virus (self-report)[101]
- Self-reported history of intensive care unit stay due to Coronavirus Disease 2019 (COVID-19) in the past three months, as severe COVID-19 may affect blood glucose levels
- Allergies to nuts
- For women, current pregnancy, breastfeeding, or plans to become pregnant during the study
- Consumption of ≥21 alcoholic drinks per week or consumption of ≥6 drinks per occasion
- For the CGM collection at the end of the study period only: known allergy to adhesives or other products involved with CGM use (e.g., skin disinfectants), current pregnancy, currently on hemodialysis or peritoneal dialysis, or people with other implanted medical devices (e.g., a pacemaker)
- Current or planned residence making it difficult to meet trial requirements (due to distance from study site and/or challenges regularly traveling to site)
- Current participation in another lifestyle intervention trial or a pharmaceutical trial
- Participation of another household member in the study; employees or persons living with employees of the study
- Other concerns regarding ability to meet trial requirements, at the discretion of the principal investigator or study coordinator

7. Recruitment and Randomization

7.A. Recruitment

We will recruit study participants from the greater New Orleans area. The initial focus of our recruitment will be mass mailing of individuals in the greater New Orleans area and recruitment through placing flyers and brochures in the community. If community-based recruitment is not successful or cost-effective, we will recruit out-patients from the Ochsner Health System and Tulane Medical Center (TMC), from which our group has prior experience recruiting patients. For this recruitment, we will focus on electronic searches of medical records and laboratory databases. We will also carry out targeted solicitation of referrals from physicians who care for patients likely to meet our inclusion and exclusion criteria.

In addition to this recruitment plan, we have several contingency plans: (1) extending recruitment to other medical centers in the New Orleans area, including the Veterans Affairs Medical Center (VAMC), East Jefferson General Hospital, and West Jefferson Medical Center, with whom Tulane has well-established relationships; (2) TV/radio advertisements; (3) arrange a news story about the study in local TV newscasts, radio talk programs, and/or print media; and (4) conduct outreach at local community events and churches. We have ample experience with each of these approaches.

Based on U.S. Census Bureau 2016 population estimates, the metro New Orleans area population was approximately 35% African American or black and 9% Hispanic.[98] Given our prior experience and population distribution in this region, we plan to recruit a distribution of study participants with 40% ethnic minorities.

7.A.1. Mass mailing, placement of brochures/flyers in community, TV/radio/billboard advertisements: Community recruitment approaches include: mass mailing individuals who live in the New Orleans area; brochures/flyers in the community; emailing the brochure; TV, radio, poster, or billboard advertisements in the New Orleans area. Interested individuals will be pre-screened; those at elevated risk for prediabetes or T2D, i.e. individuals who are obese or overweight and/or have a sufficiently high score from the American Diabetes Association's Diabetes Risk Assessment Questionnaire will be invited into the clinic for a screening visit.

7.A.2. Database searches and targeted mailing: We will request IRB approval for a waiver for HIPAA authorization to conduct database searching and hospital chart reviews for identification of potentially eligible study participants. After identifying a potentially eligible patient, we will send this individual an invitation letter signed by the investigators and/or the individual's personal physician and brochure via mail or email. Individuals who express interest in the study will have a pre-screening telephone interview for confirmation of potential eligibility. Those still interested and potentially eligible for enrollment will be scheduled for a visit at the COBRE study clinic.

7.A.3. Referrals and Local Partnerships: In addition to local physician referrals, we will explore holding outreach community events in the New Orleans area. Local community partners may post information about the study on their social media platforms. We may also identify potentially interested volunteers from a Tulane volunteer recruitment registry. We will also consider recruiting potentially eligible individuals who were screened or participated in other clinical studies in our clinic and have expressed interest in being contacted about future research projects. Potential participants referred by their physician or community partners will have a pre-screening telephone interview to confirm potential eligibility. Those who are still interested and potentially eligible for the study will be scheduled for a screening visit.

7.B. Randomization

Once eligibility has been confirmed, the study coordinator will ensure that all key variables have been entered into the trial-specific database. Additionally, the study coordinator will ensure any outliers have been recognized and reconciled, and will ensure receipt of informed consent prior to enrolling the volunteer in the study as a trial participant. Eligible participants will be *randomly assigned to the behavioral intervention or the usual diet group in a 1:1 allocation ratio*. Randomization will be stratified by sex, *using a random block group size (4, 6) to maintain balance among groups over time*. A statistician from the Methodology/Biostatistics Unit within the COBRE Clinical Research Core, who is not directly involved in the study and is not located at the clinic, will conduct the randomization. After ascertaining eligibility, clinical personnel will telephone the Methodology/Biostatistics Unit to obtain a randomization assignment. All randomization assignments will be created by a computer program and only accessible to the Staff at the Methodology/Biostatistics Unit. Once randomization assignment has been made, trial participants will be considered to be officially randomized into the study and every effort will be made to obtain complete follow-up information.

7.C. Masking

Clinical and laboratory staff members who assess outcomes will be blinded to the intervention assignment of participants. As this is a dietary intervention trial, study participants, and study staff members who conduct the intervention cannot be blinded to the intervention assignment. Participants will be instructed to avoid sharing knowledge of their treatment assignment with data collection staff members. We will have intervention visits and data collection study visits on different days and in separate locations. Data collectors will also use verbal cues and visual props to remind participants not to disclose treatment assignment. Investigators will be blinded to intervention assignment.

7.D. Safety Considerations

Participants with HbA1c >7.9% will be referred to a clinician for follow-up[102], though we will continue to collect data on these participants.

8. Dietary Intervention Program

8.A. Low-carbohydrate dietary intervention group

Participants randomized to the low-carbohydrate intervention group will receive counseling

aimed at reducing carbohydrate consumption. Phase I (*Go Low*) will occur for the first 3 months, with a goal of <40 g digestible carbohydrates per day. Key recommended foods will include: non-starchy vegetables, fish, poultry, lean meat, eggs, olive oil and other plant-based unsaturated oils, unsweetened/unsalted nuts and seeds, nut butters, and avocados. We will recommend moderate consumption of cheese and unsweetened Greek yogurt and low-carbohydrate milk. During Phase I, we will recommend limiting or avoiding other dairy, fruits, legumes, beans, and grains. Phase II (*Keep it Low*) will run from months 4 through 6. The net carbohydrate goal for this phase will be <40 g to <60 g, with the objective to choose a target that is as low as possible (e.g., as close to <40 g as possible). At 3 months, we expect different participants will have different levels of intake, with some consuming <40 g of net carbohydrates and others consuming levels higher than 60 g net carbohydrates per day. We have designed the intervention to accommodate various scenarios, with the goal of achieving as low of an intake as possible. For participants who consume >60 g per day, we will recommend they aim to either reduce or maintain carbohydrate intake.

At a brief check-in at 3-months, if participants are satisfied with their current intake, we will instruct them to stay at this intake level (though will recommend they try to reduce intake, if possible, if they are eating >60 net grams of carbohydrates or have not yet reached the study goal of <40 net grams of carbohydrates). If they prefer to make modifications, we will provide guidance on how to modify carbohydrate intake (either decrease or increase). If they choose to add back carbohydrates, we will recommend that participants add 5 grams of carbohydrates for two weeks at a time (e.g., from 40 to 45 g for 2 weeks), for a maximum total of <60 g net carbohydrates. If they choose to further reduce carbohydrate intake, we will provide guidance on this as well. As they modify carbohydrate intake, we will have them monitor their satisfaction with their diet and energy levels. If adding back carbohydrates, we will recommend participants add back in foods like fruit (preferably berries), legumes, beans, and dairy, while still aiming to keep total carbohydrate intake as low as possible (e.g., as close to <40 net grams as able).

Materials: We will develop a handbook that will be given to participants. This will contain diet guidelines, recipes, meal planners, shopping lists, and guides for counting carbohydrates and reading nutrition labels.

Meals: Participants will select their own menus and prepare or buy their meals according to the guidelines. We will provide participants with supplemental low-carbohydrate foods, such as nuts, olive oil, and other products, to help facilitate their compliance.

Counseling: Sessions and materials will focus on instructing participants to reduce digestible carbohydrate consumption by increasing consumption of protein and fat, with a particular emphasis on increased consumption of heart-healthy unsaturated fats. After randomization, participants will begin the 3-month *Go Low* phase consisting of individual counseling sessions each week for the first four weeks, followed by 4 group sessions held every other week with phone follow-ups occurring in between group sessions. During the *Keep it Low* phase, months 4 through 6, participants will attend 3 monthly group sessions and have 3 telephone follow-ups. There will be a total of 11 in-person sessions and 7 telephone follow-ups over the full study

period. Sessions will be approximately one hour and phone calls will be no longer than thirty minutes. These sessions will consist of diet instruction and supportive counseling, addressing results from 24-hour dietary recalls, teaching self-monitoring and individual diet goal changes, and provide suggestions for replacing usual foods containing carbohydrates. Study materials will be modified from materials used in the successful MACRO study. The JFI will work closely with Drs. He and Bazzano to develop the intervention based on the MACRO study.
Carbohydrate goal: In the MACRO study, carried out among individuals from the same study region, a behavioral intervention aimed at reducing digestible carbohydrate intake to <40 g/day led to reductions in weight and improvements in cardiovascular risk factors among individuals with obesity.[30] Additionally, restriction of carbohydrate intake to <50g/day is sufficient to induce ketosis.[103] Our rationale for having a multi-phased intervention is to allow participants to see how they feel on the more restrictive diet, and see how they respond to small increases in carbohydrate consumption. A similar technique was applied in the DIETFITS published study.[104] The higher target of 60 net grams may be more sustainable long-term and also allows for consumption of healthy higher-carbohydrate foods, such as berries, legumes, beans, and limited whole grains.

8.B. Usual Diet Group

We will provide participants randomized to the usual diet group written information with standard dietary advice. Participants randomized to the usual diet group will not receive ongoing dietary recommendations. Moreover, they will continue to receive clinical care recommendations from their physician(s). To encourage continued participation in the study and minimize the difference in participant contact with study staff, participants will be offered optional monthly educational sessions on topics unrelated to the study intervention, such as retirement planning or health insurance options.

In another effort to encourage continued participation, patients randomized to the usual diet group will be offered a copy of all available intervention materials, as well as an optional one-hour counseling session with the interventionist following their final study visit.

8.C. Physical Activity

At baseline, both groups will receive written information in study materials with standard physical activity recommendations.

8.D. Dietary Adherence

Strategies for measuring compliance: For the low-carbohydrate diet, adherence will be assessed through measurement of urinary ketones at 3-month visit and 6-month TV. Study staff will make all reasonable effort to maintain adherence of participants to the visit schedule and diet intervention.

Strategies for improving compliance:

- At the screening visit, we will ask participants who does most of the cooking in their households and how often they eat outside of the home. Results from the screening visit will help to select a group of cooperative study participants.
- Visits will be scheduled at the participants' convenience and to minimize waiting time and trips to the clinic; reminder calls, texts or emails (chosen based on participant preference) will also be made prior to clinic visits.
- We will hire enthusiastic staff who have good interpersonal skills.
- Participants will receive $40 gift cards for use at food stores or retailers when they complete each study visit, for a maximum of $120 over the study period.
- Participants will receive $25 gift cards when they return the stool specimens, for a maximum of $75 over the study period.

- Participants will receive a $25 gift card when they return the continuous glucose monitor, for a maximum of $25 over the study period.
- Participants will be offered small gifts (such as pens or magnets with study logo) to encourage compliance and adherence to the study protocol.
- Participants will be provided with recipes, shopping lists, and food samples for the intervention diet.

9. Safety of Dietary Intervention

On the low-carbohydrate diet, participants will be encouraged to primarily focus on replacing carbohydrates (particularly highly refined carbohydrates) with foods such as non-starchy vegetables, olive oil and other plant-based unsaturated oils, nuts and seeds, avocados, eggs, seafood, tofu, poultry, and lean meat. Consumption of more unsaturated fats, protein, and fiber will be recommended. Participants will not be encouraged to increase consumption of saturated fats and will be advised to avoid consumption of trans fats.

In animal studies, a high-protein diet has decreased renal function[105] In the MACRO study, on average, participants tended to increase consumption of fats, rather than protein.[30] The safety and tolerability of low-carbohydrate diets have previously been assessed in many studies, including MACRO.[30] A number of studies, including some in individuals with T2D, have found no clinically significant increases in estimated glomerular filtration rates,[33,38–40] microalbuminuria,[106] albumin excretion rate,[33,38] urinary albumin,[38] serum creatinine,[38,40,106–111] albumin,[40] or uric acid levels.[107–110] A small study of a very low-carbohydrate diet found significant increase in uric acid excretion, but no significant change in 24-hour urine creatinine clearance.[110]

Study staff will carefully monitor participants' BP, and HbA1c more often than what would occur in routine healthcare visits. A Data Safety and Monitoring Board (DSMB) consisting of an independent group of experts in areas such as biostatistics, nutrition medicine, general internal medicine, and endocrinology has been formed. The board will regularly review trial progress, including unblinded interim results, and can recommend that the trial prematurely end if participants are at risk or if it becomes clear that the trial will not be able to yield statistically significant results.

10. Data Collection

10.A. Study visits

After the initial pre-screening interview, in which data on medications and medical conditions will be collected, there will be one screening visit, one randomization visit, a follow-up study visit at 3 months and a termination visit (TV) at 6 months. *Table 3* summarizes study procedures and data collected at each study visit.

Table 3. Study visit schedule and data collection

	Pre-screen	SV	RZ	3 month	6 month TV
Pre-screen eligibility	X				
Informed consent		X			
Compliance form		X			
Eligibility confirmation			X		
Randomization			X		
Demographics		X			
Medical history questionnaire			X	X	X
Medications		X	X	X	X
Lifestyle (alcohol, cigarette smoking)			X	X	X
Physical activity			X	X	X
24-hour diet recall*			X	X	X
3 BP measurements		X	X	X	X
Body weight			X	X	X
Height			X		
Waist circumference			X	X	X
Spot urine test			X	X	X
Blood samples		X	X	X	X
Stool specimen			X	X	X
Side-effects assessment			X	X	X
Nut Consumption					X
CGM**					X

*Two 24-hour dietary recalls will be collected; one should cover a weekend and the other a weekday.
**The CGM will be worn for up to 14 days prior to the 6-month TV and returned at the TV or by mail. Participants will come in for a 6-month CGM visit (prior to the 6-month TV) to have the CGM inserted.

10.A.1. Screening/baseline visits:

Participants will be pre-screened by phone or in-person by study staff to confirm eligibility for the Screening Visit. An approved questionnaire will be used to ensure the patient is eligible to attend the in-clinic screening visit, and the patient will be scheduled thereafter.

At the screening visit (SV), participants will provide informed consent for the screening and main study. Study staff will schedule the SV and randomization (RZ) visit at least 2 days apart. If a participant is eligible based on the screening visit, he or she should be scheduled for the RZ once a sufficient group of eligible individuals has been recruited. Participants should be scheduled for the RZ at least 2 days and no more than two weeks after the screening visit.

At the screening visit, we will collect information on inclusion/exclusion criteria, medication use, and demographics. At this visit, we will also have participants complete a compliance form,

to assess their ability to complete the study and attend all intervention and study visits. We will also measure blood pressure, and take blood samples. Women will be asked if they are postmenopausal or if they had surgical sterilization (such as a tubal ligation). If not, they will be tested for pregnancy through a spot urine test; pregnant women will be excluded from the study. Blood samples will be collected for measurement of HbA1c, and, if not available from medical records in the past 3 months, serum creatinine and hemoglobin. These blood samples will be used for excluding individuals with HbA1c <6.0 or >6.9, eGFR ≤45 mL/min/1.73 m^2, or hemoglobin <11 mg/dL.

Before the start of the intervention, participants will complete two 24-hour dietary recalls. At least one (and potentially both) 24-h recalls will be collected by phone. At the RZ, participants will learn about the randomization process. After requirements for eligibility have been satisfied, participants will formally be enrolled into the study and randomized to either the active intervention or control group. We will collect fasting blood samples for later measurement of glucose, insulin and lipids and other potential future measurements. At this visit, we will measure blood pressure, body weight, height, and waist circumference. We will collect information on medical history, lifestyle risk factors, and physical activity. A spot urine sample will be collected to measure ketones and other markers and participants will be asked to collect a stool specimen for potential use in future microbiome studies.

10.A.2. Follow-up visits and final visit: A follow-up visit will occur at 3 months after randomization. A final visit will occur at 6-months post-randomization. These visits should be scheduled as close as possible to the 3-, or 6-months after randomization. At each visit, study staff will conduct dietary recalls and anthropometric measures, take 3 BP measurements, collect a fasting blood sample to measure HbA1c. At least one (and potentially both) 24-h recalls will be collected by phone. We will also collect samples for later measurement of glucose, insulin, lipid profile, and other potential future measurements. A spot urine sample will be collected to measure ketones and other markers. Participants will be asked to collect a stool specimen for potential use in future microbiome studies. Information about participants' nut consumption over the past 6 months will be collected at the 6-month TV. At study termination, we will mail participants and/or their physicians a summary of trial results and a record of participant BP, anthropometric measures, and laboratory data from the trial period. Throughout the study period, we will provide participants with their blood pressure and weight. We will request that, if possible, a participant who is prescribed a diabetes medication during the course of the study comes into the clinic for an additional separate supplemental visit for a fasting blood sample collection prior to starting the medication. Maintaining follow-up of all randomized participants is of utmost importance to ensure data integrity. In the event that a participant moves during the study we may request that the participant provides a fasting blood sample at a local LabCorp facility (this will be described on the informed consent form).

10.A.3. CGM 6-month visit: Individuals who are willing to wear the CGM prior to their 6-month termination visit will come to the research clinic approximately 2 weeks before their 6-month termination visit for their CGM visit. At this time, they will have a factory-calibrated CGM inserted just under the skin of the upper arm and activated.[112] If the CGM falls off of a participant prior to 14 days of wear, we may request that the participant (if willing) returns to the clinic for the CGM to be re-inserted. The CGM will be returned at the 6-month TV, or by mail.

10.B Study measurements

Measurements obtained for this study will be based on those used in other clinical trials and epidemiologic studies. We are familiar with strengths and limitations of such measurements and the importance of staff training and certification and use of stringent quality control procedures.

10.B.1. Questionnaires: Study staff will administer a *medical history questionnaire* at the RZ, 3-month visit and 6-month TV to assess medical conditions that would necessitate changes in therapy or medical management beyond this study's scope. At the SV, *demographic factors* will be collected. At the SV, RZ, 3-month and 6-month TV, we will also administer *medication tracking questionnaires*. At RZ, 3-month, and 6-month TV, we will collect health behavior and physical activity data.

10.B.2. Dietary measurements: Two 24-hour dietary recalls will be obtained from participants at baseline. At or close to both the 3-month and 6-month TV, two more 24-hour dietary recalls will be collected. The second sets of two recalls will be collected as closely as possible to 3- or 6-month dates after randomization, with a goal of being within 2 weeks of the respective follow-up period. For each time point, one recall should reflect week day consumption, while the other should reflect weekend day consumption and the two recalls should be at least 2 days apart from each other. We will use computer software (Nutrition Data System for Research [NDS-R]) and food models used in previous and ongoing studies. Recalls will be conducted by a study staff member who has been certified in the use of NDS-R. Food composition tables of the NDS-R will be used to calculate dietary nutrient intakes. The baseline dietary assessment will be done to characterize individual dietary intake of carbohydrates and other macronutrients. During the intervention, repeated measurements will be used to monitor short-term changes in mean dietary nutrient intake between the intervention and usual diet groups.

10.B.3. Assessment and management of adverse effects: At RZ, 3-month visit, and 6-month TV, we will administer *symptom questionnaires*, which will consist of a checklist to identify participant complaints. Prior studies of low-carbohydrate dietary interventions suggest that participants may experience a range of adverse effects, including constipation, halitosis, fatigue, headache, thirst, polyuria, and nausea. Severe adverse events will be promptly reported to the DSMB. Management of adverse effects will be based on the protection and safety of the participant. If severe adverse effects are reported by a participant, carbohydrate intake will be examined and altered in order to mitigate the adverse effect. In any extreme cases, participants will be instructed to stop the intervention. As this is a behavioral intervention, adverse effects should be rare.

10.B.4. BP and anthropometric measures: BP will be measured 3 times at the screening visit and at each follow-up visit. To minimize random error, averages of the measurements at the screening and randomization visits will be taken. BP will be measured by a standard automated blood pressure measurement device (OMRON HEM-907 XL Professional Digital Blood Pressure Monitor) and performed by experienced staff, trained and certified in BP measurement. Prior to BP measurements, participants will be asked to rest quietly in a seated position with their feet supported for at least 5 minutes; staff members will use an appropriate cuff size over a bare arm. Trained and certified staff will measure weight using a calibrated balance beam scale at the screening visits, and follow-up visits. Weight and height will be measured at the RZ, and weight will also be measured at the subsequent visits.

10.B.5. Blood samples: For SV, participants will not need to fast. For RZ, 3-month visit, and 6-month TV, participants will be instructed to fast for 12 hours prior to blood sample collection. At SV, blood samples will be collected to measure HbA1c, hemoglobin, and serum creatinine. At RZ, fasting blood samples will be collected and stored as serum/plasma, whole blood aliquots and buffy coats at -80°C for future analyses, including measurement of glucose, insulin and lipids, and potential future metabolomics or genetic analyses. At 3-month visit and 6-month TV,

we will collect fasting blood samples to measure HbA1c and will also store additional serum/plasma, whole blood aliquots and buffy coats at -80°C for future analyses, including measurement of glucose, insulin and lipids, and potential future metabolomics or genetic analyses. Details of the blood sample collection are shown in *Table 4*. In the event that a participant moves, we may request that they collect a fasting blood sample at a local LabCorp and we will measure HbA1c, glucose, insulin, and lipids at the 3 and 6-month visits. HOMA-IR will be calculated from fasting glucose and insulin.

HbA1c will be measured at LabCorp (Roche Tina Quant; fresh samples) from whole blood collected at screening, 3-month, and 6-month visits. Following trial completion, to improve precision of HbA1c estimates, HbA1c will also be measured at the Diabetes Diagnostic Laboratory (ion-exchange method; Tosoh G8 HPLC Analyzer; frozen samples) from whole blood collected at randomization, 3-month, and 6-month visits. Samples with Hb variants (C, D, E, S) that are measured at the Diabetes Diagnostic Laboratory will be reflexed for measurement by boronate affinity HPLC (Trinity Premier Hb9210).[113]

Table 4. Study visit schedule for specimen collection

	SV	RZ	3 month	6 month TV
HbA1c	X		X	X
Serum creatinine	X			
Hemoglobin	X			
Storage of aliquots and buffy coats (for measurement of glucose, insulin, lipids, other potential future measurements)		X	X	X
Urine sample for pregnancy	X			
Urine sample for ketones and other markers		X	X	X
Stool sample		X	X	X
CGM				X

10.B.6. Urine collection: Study staff will collect spot urine specimens at RZ, interim visit, and the final visit to measure ketones.

10.B.7. Stool Specimen Collection: Stool samples will be collected from all eligible and willing trial participants during the RZ, 3-month, and 6-month TV, following the protocol set forth by the Human Microbiome Project.[114,115] Participants will be given a stool collection kit, which includes the specimen collection container, a shipper for home collection, and a form to record the timing and pertinent information related to specimen collection. Participants will be instructed to return the kit to the clinic within two days of collection (in-person or via shipping), at which time stool samples are stored at -80°C until analyses. Kits and instructions for collection will be reviewed by study staff with the participant at the required visits.

10.B.8. CGM at 6 months: Approximately two weeks before their 6-month termination visit, participants willing to wear the CGM will come to the clinic to have their CGM inserted. This CGM has been found to be accurate compared with capillary blood glucose reference values over 14 days of wear.[112] This CGM records interstitial glucose every 15 minutes throughout the day for a period of up to 14 days. The sensor uses a thin, flexible filament inserted just under the skin to measure glucose and is read through near-field communication by touching the reader to the sensor. For quality control of the CGM data, a subset of participants who wear the CGM will be asked to wear two CGMs at the same time, one on each arm. Prior to insertion, one arm will be designated as the arm used for data collection and the other arm as a duplicate.

11. Quality Assurance and Quality Control

Throughout the study, we will implement rigorous quality assurance (QA) and quality control (QC) measures. We describe some of these approaches here.

11.A. Manual of procedures

We will develop a manual of procedures (MOP) based on the MOP successfully used in the MACRO behavior change clinical trial. The MOP will provide instructions and describe procedures for staff training and certification, participant recruitment, intervention counseling, study form completion and procedures, data entry, safety measures (e.g., methods for emergency participant/primary care physician contact).

11.B. Training and certification

Trial personnel will all be required to participate in a training session before the start of any trial procedures or measurements. Training will include particular attention to core trial elements, such as recruitment, retention, intervention, completeness and quality of data acquisition (e.g., blinding procedures for the data collectors), and data entry. Separately, intervention staff will meet and receive detailed instruction on trial approaches to group and individual behavior change counseling and procedures that ensure data collectors are blinded to treatment assignment. Throughout the trial, periodic re-training/certification sessions will be conducted to ensure all trial-related procedures are being applied in a standard manner. Investigators will also monitor all aspects of study performance to ensure study goal achievement, timeliness and completeness of study visits, completeness and quality of data collection, intervention adherence, and adequacy of the procedures separating data collection from intervention. Study staff and investigators will pay particularly careful attention to the quality of all laboratory measurements, particularly key exposure, outcome, and intervention monitoring variables.

12. Sample Size and Statistical Power

The primary outcome will be the difference in the change of HbA1c between the intervention and usual diet groups from baseline to 6 months of follow-up. Preliminary data from ADEPT suggests that the SD of the 6-month change in HbA1c is 0.28%, with preliminary 6-month follow-up >90%. Based on an estimated standard deviation of change in HbA1c of 0.35% and 95% follow-up, we would need 142 participants to have 80% power to detect a difference in the change in HbA1c from baseline to 6 months of 0.17% between the intervention and usual diet groups. Assuming 95% follow-up, we would need 150 total participants.

In a follow-up analysis of the DPP, a 0.15% decrement in HbA1c at 6 months was associated with a 16% lower risk of incident T2D, adjusting for treatment arm, age, sex, race/ethnicity, household income, and baseline HbA1c.[116] In a pooled analysis from the Atherosclerosis Risk in Communities and Framingham Heart Studies, a 0.15% higher HbA1c was associated with an 16% higher risk of T2D over 20 years, a 30% higher risk over 8 years, and a 13% higher risk after 8 years, after adjusting for age, sex, fasting glucose, triglycerides,

HDL, family history of T2D, SBP, and BMI.[117] Additionally, in EPIC-Norfork, a difference of HbA1c of 0.15% was associated with approximately a 5% higher risk of CHD among those free of CHD, stroke, diabetes, and with HbA1c <7.0%.[118]

In the DPP study, HbA1c was 0.15% lower among those in the lifestyle intervention arm than in the control arm at 6-months follow-up; HbA1c at study commencement was 5.9% and levels increased in the control arm and decreased in the behavioral intervention arm.[22] In a small behavioral intervention study (n=34) of a very low-carbohydrate ketogenic diet compared with a moderate carbohydrate, calorie-restricted, low-fat diet among individuals with type 2 diabetes or prediabetes (HbA1c >6.0%) and elevated body weight (BMI >25), mean HbA1c decreased from 6.6% to 6.1% in the very low-carbohydrate group and from 6.9% to 6.7% in the moderate carbohydrate group over a 12-month period; additionally, the very low-carbohydrate group had greater reductions in glucose-lowering medications.[37]

Detectable differences are listed for HbA1c and secondary outcomes in *Table 5*, based on data from prior studies.[30,53,56,119] These were calculated for unpaired t-tests, power of 80%, two-sided alpha of 0.05, and assuming 95% follow-up of 150 participants, which would yield an estimated 142 participants.

Table 5. Minimum detectable differences

Change from baseline to 6 months in	SD	Detectable Difference
HbA1c, %	0.35	0.17
Fasting glucose, mg/dL	12.5	5.9
Fasting insulin, mU/mL	7.0	3.3
HOMA-IR	2.0	0.95
SBP (mmHg)	4.2	2.0
DBP (mmHg)	3.0	1.4
Total/HDL ratio	1.2	0.57
Body weight (kg)	6.6	3.1
Waist circumference (cm)	8.0	3.8
Estimated CVD risk (%)*	6.7	3.4

*10-y CVD risk, among estimated 85% or 126 participants free of CVD

13. Data Management and Analysis

13.A. Data management

Personnel will double-enter all study data. Prior to entering data, the study coordinator will conduct quality control on all study forms, study measures, and laboratory data. Data will be cleaned and logically checked.

13.B. Data analysis plan

The data will be analyzed using *intention to treat*; this involves comparing the primary and secondary outcomes between participants based on randomization assignment.

13.B.1. Exploratory data analysis: We will express group data for variables with a normal distribution as mean ± standard deviation and for variables not normally distributed as median and interquartile ranges (or will log-transform the variables to obtain a normal distribution). We will compare means and percentages of baseline variables across the intervention and usual diet group to assess similarity of these groups at randomization.

13.B.2. Primary, secondary, and exploratory outcomes: We will test the primary study hypothesis that there is a greater reduction in HbA1c from baseline to 6 months in the intervention group than in the usual diet group. To do this, we will use linear regression with change in HbA1c from baseline to 6 months as the dependent variable

We will use Bland-Altman plots to explore whether there is a systematic difference in HbA1c measured by LabCorp compared with DDL. If there is not a systematic difference in HbA1c measured at these two laboratories, to improve precision in the estimate of the primary outcome (difference in change in HbA1c between the intervention and control groups), we will use the weighted average of change in HbA1c measures from the two labs as the outcome (weights defined as the ratio of the variance for each lab over the sum of the variance) (50). If we observe a systematic difference between HbA1c measured at LabCorp and DDL, we will use HbA1c measured at DDL.

We will use a similar approach to test the difference in the change between baseline and 6-month values of secondary outcomes (Specific Aim 2) and exploratory outcomes. In exploratory analyses, we will also test the difference in the change between baseline and 3-month values of outcomes (HbA1c, fasting glucose, systolic blood pressure, total-to-HDL cholesterol, body weight, insulin, HOMA-IR, diastolic blood pressure, waist circumference, and estimated CVD risk). We will also explore the use of mixed effects models to assess overall net change in HbA1c (by treating time as a linear variable) and other outcomes over the full study period. For each outcome, we will test modeling assumptions and will consider transformation of outcomes, if needed, to meet modeling assumptions. We will also perform sensitivity analyses using Markov-chain Monte Carlo techniques to impute missing values of the outcomes, using covariates for this imputation and will compare results from this analysis with our primary findings.

15. Anticipated Challenges

Recruitment: Many clinical trials have challenges recruiting sufficient numbers of volunteers who meet the trial inclusion and exclusion criteria and demonstrate a high level of adherence to trial protocol requirements. Fortunately, the investigators are very experienced with clinical trial recruitment, and have several contingency options that can be used to meet unanticipated shortfall in recruitment.

Intervention: The investigators are aware of and understand challenges related to achieving and maintaining compliance with a behavioral intervention. The investigators have much experience and success in behavioral interventions, with recent success of a behavioral intervention aimed at reducing carbohydrate intake among participants from the New Orleans area. The JFI will collaborate with Drs. He and Bazzano to develop the intervention program based on the MACRO study and will assess potential barriers to implementation and intervention adherence (such as financial concerns and range of dietary patterns of study participants) during the planning and pilot phase of the study.

Adherence: Though adherence and follow-up are also main challenges related to clinical trials, the investigators have an outstanding track record for studies that have recruited participants for both clinical and epidemiology studies in the Greater New Orleans area. To encourage adherence, small gifts (such as pens or magnets with study logo) will be offered to all study participants.

16. Protection of Human Subjects

This Human Subjects Research meets the definition of a clinical trial. All investigators will be required to complete the CITI (Collaborative Institutional Training Initiative) program in the ethical use of human participants in research.

16.A. Protection of human subjects from research risks

16.A.1. Risks to the subjects

Human subject involvement and characteristics: We expect to enroll 150 participants, with an equal male to female ratio. We expect to enroll approximately 35 participants who are African-Americans, and another 12 with mixed racial backgrounds, and a few Asian participants given the demographics of the greater New Orleans area. Overall, given the demographics of greater New Orleans, we estimate approximately 10% participants of Hispanic or Latino ethnicity, 50% of them with African-American racial background.

Inclusion criteria

- Men or women ages 40 to 70 years: aim to recruit 50% women
- Any race or ethnicity: aim to recruit 40% ethnic minorities (i.e., African American and Hispanic), close to population in the greater New Orleans area[98]
- HbA1c 6.0–6.9%
- Willing and able to provide informed consent

Exclusion criteria

- Diagnosed type 1 diabetes mellitus based on patient self-report
- Use of agents affecting glycemic control (medications for diabetes, oral glucocorticoids) within the past three months prior to enrollment based on patient self-report
- Medical condition in which low-carbohydrate diet may not be advised
    - Estimated glomerular filtration rate (eGFR) ≤45 mL/min/1.73 m², which is close to the 5th percentile of eGFR among non-diseased individuals of 70 years of age[99]
    - Self-report of liver disease due to hepatitis or alcohol; osteoporosis; untreated thyroid disease; gout; or cancer (other than non-melanoma skin cancer) requiring treatment in the past year, unless prognosis is excellent
- Factors that may affect HbA1c:
    - Hemoglobin <11 mg/dL (cutpoint for moderate-to-severe anemia, which could lead to falsely elevated or lowered HbA1c)[100]
    - Recent blood donation or blood transfusion (self-report, past 4 months)
    - Human Immunodeficiency Virus (self-report)[101]
- Self-reported history of intensive care unit stay due to Coronavirus Disease 2019 (COVID-19) in the past three months, as severe COVID-19 may affect blood glucose levels
- Allergies to nuts
- For women, current pregnancy, breastfeeding, or plans to become pregnant during the study
- Consumption of ≥21 alcoholic drinks per week or consumption of ≥6 drinks per occasion
- For the CGM collection at the end of the study period only: known allergy to adhesives or other products involved with CGM use (e.g., skin disinfectants), current pregnancy, currently on hemodialysis or peritoneal dialysis, or people with other implanted medical devices (e.g., a pacemaker)
- Current or planned residence making it difficult to meet trial requirements (due to distance from study site and/or challenges regularly traveling to site)
- Current participation in another lifestyle intervention trial or a pharmaceutical trial
- Participation of another household member in the study; employees or persons living with employees of the study
- Other concerns regarding ability to meet trial requirements, at the discretion of the principal investigator or study coordinator

Sources of materials: Medical information data will be collected from the participant and, after written authorization for the disclosure of protected health information, from participants' medical records. Blood and urine samples will be collected to measure HbA1c, glucose, insulin, lipids, creatinine, ketones, and test for pregnancy. Blood pressure, height, weight, waist circumference and other physical measurements will be obtained.

Potential Risks: Risks associated with venipuncture, the diet intervention, CGM insertion, and collection of confidential medical information are the main risks associated with this study. There is a small risk of bruising and a rare chance of local infection associated with standard venipuncture to collect blood samples. The risks of wearing the CGM are minimal. There is a very low risk for local infection at the skin site where the sensor is inserted into the skin. Possible symptoms associated with the CGM sensor application or the adhesive used to keep the sensor in place include skin irritation or redness, skin inflammation, pain or discomfort, bleeding, bruising, swelling, skin rash, itching, scarring or skin discoloration, or allergic reactions to the sensor needle or adhesives. Sensors may fracture in situ on rare occasions. The dietary intervention has minimal risks. Reduced carbohydrate consumption may lead to adverse effects, including constipation, halitosis, fatigue, headache, thirst, polyuria, and nausea. At RZ, 3- , and 6-month visits, we will administer *symptom questionnaires*, which will consist of a checklist to identify participant complaints. Adverse effects of the low-carbohydrate diet will be assessed at counseling sessions. Severe adverse events will be promptly reported to the DSMB. Management of adverse effects will be based on the protection and safety of the participant. If severe adverse effects are reported by a participant, carbohydrate intake will be examined and altered in order to mitigate the adverse effect. In any extreme cases, participants will be instructed to stop the intervention. Furthermore, there is a potential risk of unauthorized disclosure of medical information. This risk will be minimized by using a strict protocol for data processing and by the appropriate training of all study personnel. Protection against risks is described in more detail below.

16.A.2. Adequacy of protection against risks

Recruitment and Informed Consent: The proposed study will be conducted following strict guidelines for the protection of the rights of human volunteers. The informed consent document will be signed by all participants at the screening/baseline visit. The informed consent form will be developed in the first year of the study and approved by the IRB. The consent form will clearly state the purpose, eligibility criteria, and protocol of the study, the potential benefits and risks of participation, and the subject's right to refuse to participate or withdraw. Prior to signing the informed consent, research staff will review the details of the consent form orally with the participant, and answer any questions that the participant has concerning participation in the study. In the case of potential participants with low or no literacy, the informed consent form will be read to the participant in its entirety and any questions will be answered prior to the participant signing or marking the form. Specifically, the following must be accomplished during the informed consent process:

- The participant must be informed that participation in the study is voluntary and that refusal to participate will involve no penalty or loss of benefits.
- The participant must be informed of the purpose of the study and that it involves research.
- The participant must be informed of any alternative procedures, if applicable.

- The participant must be informed of any reasonably foreseeable risks.
- The participant must be informed of any benefits from the research.
- An outline of safeguards to protect participant confidentiality will be included, as well as an indication of the participant's right to withdraw without penalty. This should be balanced with a discussion of the effect that withdrawals will have on the study, and the responsibility a participant has, within limits, to continue in the study if he or she decides to enroll.
- The participant must be informed of his or her right to have questions answered at any time and whom to contact for answers or in the event of research-related injury.
- The participant must be informed as to whether or not compensation is offered for participation in the study and/or in the event of a medical injury.
- The participant must be informed that he/she will be notified of any significant changes in the protocol that might affect their willingness to continue in the study.

Written authorization for the disclosure of protected health information will be obtained during the consent process on a separate form (HIPPA authorization), if applicable.

Protection against Risk: The protocol and informed consent will be approved by the Institutional Review Boards at Tulane University Health Sciences Center and any other participating institution for recruitment. The study participants will be given contact information for the Study Coordinator and Study PI to answer questions or to report complaints. Patients can withdraw from the study at any time without any effect on their medical care. Standard techniques will be used by trained, certified phlebotomists or study nurses for blood sample collection at the study clinics where emergency medical equipment is available. Confidentiality of all data collected during the study will be maintained through the use of unique, encrypted study identification (ID numbers, rather than patient names, in the study database). These encrypted ID numbers will not be mathematical derivation of the subject's medical record number or any other patient identifier. No information will be disclosed in an individually identifiable form in any type of presentation or publication. There will be password-required access to the computerized file linking the study numbers to patient identifiers at the study clinics. Password-access will be restricted to pertinent research staff only. All study data will be kept in locked file cabinets in locked rooms, accessible only by study staff with permission. All data transmissions will use HIPPA-compliant password protected system and will be secured through a virtual private network.

16.A.3. Potential benefits of the proposed research to human subjects and others

Study participants may have improved glycemic outcomes, blood pressure, lipid levels, may lose weight and reduce waist circumference, and may have improvement in other risk factors for CVD and development and progression of T2D. Participants will receive multiple blood pressure, weight, waist circumference, and blood tests. If vital signs or lab values are clinically abnormal, participants will be referred to their primary care physician, or if they do not have a primary care physician, one will be assigned through the Tulane University Community Health Center, which is a part of the core clinical facilities. At the end of the study, we will provide the participants' primary care physician with a copy of all the blood pressure, weight, laboratory results, and (if applicable) CGM results.

16.A.4. Importance of knowledge to be gained

Information from this study may contribute to reducing the onset of T2D and the burden of T2D and cardiovascular disease in Louisiana and the general US population. The study has

potential for public health impact; results from this study may provide evidence in support of promoting alternative dietary styles (than the most commonly used low-fat diet) among individuals with high T2D risk or with T2D.

16.A.5. Remuneration

For screening visit 2, the two follow-up visits, and the final visits, participants will receive $40 gift cards for use at food stores or retailers (after completing each study visit), for a maximum of $120 over the study period. Participants who return a stool sample will receive $25 gift cards for each stool sample that they return, for a total of $75 for three stool samples. For the CGM 6-month study, participants will receive $25 gift cards for returning the CGM, for a maximum of $25. Participants will also receive free parking for each intervention and study visit.

16.B. Data safety and monitoring plan

A DSMB, which consists of 3 experts who are not otherwise affiliated with the COBRE program, has been formed. Study investigators for this study will prepare accurate and timely data tables and reports for the DSMB meetings. The members of the DSMB will serve in an individual capacity and provide their expertise and recommendations. The primary responsibilities of the DSMB are: 1) periodically review and evaluate the accumulated study data for participant safety, study conduct and progress, and, when appropriate, efficacy, and 2) make recommendations to the NIH concerning the continuation, modification, or termination of the trial. The DSMB considers study-specific data, as well as relevant background knowledge, about the disease, test agent, and patient population under study.

The DSMB is responsible for defining its deliberative processes, including event triggers that would call for an unscheduled review, stopping guidelines, unmasking (unblinding) and voting procedures prior to initiating any data review. The DSMB is also responsible for maintaining the confidentiality of its internal discussions and activities, as well as the contents of reports provided to it.

The DSMB will review each protocol for any major concerns prior to implementation. During the trial, the DSMB should review cumulative study data to evaluate safety, study conduct, and scientific validity and integrity of the trial. As part of this responsibility, DSMB members must be satisfied that the timeliness, completeness, and accuracy of the data submitted to them for review are sufficient for evaluation of the safety and welfare of study participants. The DSMB should also assess the performance of overall study operations and any other relevant issues, as necessary.

Items reviewed by the DSMB include:

- Interim/cumulative data for evidence of study-related adverse events;
- Interim/cumulative data for evidence of efficacy according to pre-established statistical guidelines, if appropriate;
- Data quality, completeness, and timeliness;
- Adequacy of compliance with goals for recruitment and retention, including those related to the participation of women and minorities;
- Adherence to the protocol;
- Factors that might affect the study outcome or compromise the confidentiality of the trial data (such as protocol violations, unmasking, etc.); and,
- Factors external to the study, such as scientific or therapeutic developments, that may impact participant safety or the ethics of the study.

The DSMB will conclude each review with their recommendations to the COBRE program and NIH/NIGMS as to whether the study should continue without change or be modified or terminated. Recommendations regarding modification of the design and conduct of the study could include:

- Modifications of the study protocol based upon review of the safety data;
- Suspension or early termination of the study or of one or more study arms because of serious concerns about subjects' safety, inadequate performance or rate of enrollment;
- Suspension or early termination of the study or of one or more study arms because study objectives have not been obtained according to pre-established statistical guidelines; and,
- Corrective actions regarding a study when the performance appears unsatisfactory or suspicious.

Confidentiality must always be maintained during all phases of DSMB review and deliberation. Usually, only voting members of the DSMB should have access to interim analyses of outcome data by treatment group. Exceptions may be made when the DSMB deems it appropriate. The reason and to whom the exceptions for access to interim analyses is granted will be documented in the Closed Session Report. DSMB members must maintain strict confidentiality concerning all privileged trial results ever provided to them. The DSMB should review data only by masked study group (such as X vs. Y rather than experimental vs. control) unless or until the DSMB determines that the identities of the groups are necessary for their decision-making. Whenever masked data are presented to the DSMB, the key to the group coding must be available for immediate unmasking.

The DSMB will meet twice per year. The junior faculty investigator will attend these meetings and will be responsible for preparing and presenting up-to-date statistical reports on the progress of their trials. These reports will include data on recruitment, randomization, adherence, as well as statistical tests and special analyses requested by the DSMB. The Administrative Core will be responsible for organizing the annual in-person meeting and semiannual conference calls. They will put together data and materials submitted by junior faculty investigators and deliver them to DSMB members. The COBRE investigators will meet with the DSMB in the Open Session and not be present for the Closed Session.

Bibliography and References Cited

1. The Emerging Risk Factors Collaboration. Diabetes mellitus, fasting blood glucose concentration, and risk of vascular disease: a collaborative meta-analysis of 102 prospective studies. *Lancet*. 2010;375(9733):2215-2222. doi:10.1016/S0140-6736(10)60484-9.
2. Flaxman SR, Bourne RRA, Resnikoff S, et al. Global causes of blindness and distance vision impairment 1990-2020: a systematic review and meta-analysis. *Lancet Glob Heal*. October 2017. doi:10.1016/S2214-109X(17)30393-5.
3. United States Renal Data System. *2016 USRDS Annual Data Report: Epidemiology of Kidney Disease in the United States*. Bethesda, MD: National Institutes of Health, National Institute of Diabetes and Digestive and Kidney Diseases; 2016. https://www.usrds.org/2016/view/Default.aspx.
4. Narres M, Kvitkina T, Claessen H, et al. Incidence of lower extremity amputations in the diabetic compared with the non-diabetic population: A systematic review. *PLoS One*. 2017;12(8):e0182081. doi:10.1371/journal.pone.0182081.
5. Centers for Disease Control and Prevention. *National Diabetes Statistics Report, 2017*. Atlanta, GA: Centers for Disease Control and Prevention, U.S. Dept of Health and Human Services; 2017.
6. Centers for Disease Control and Prevention. *Long-Term Trends in Diabetes*. Atlanta, GA: Centers for Disease Control and Prevention, U.S. Dept of Health and Human Services; 2017.
7. NCD Risk Factor Collaboration (NCD-RisC). Worldwide trends in diabetes since 1980: A pooled analysis of 751 population-based studies with 4.4 million participants. *Lancet*. 2016;387(10027):1513-1530. doi:10.1016/S0140-6736(16)00618-8.
8. American Diabetes Association. Economic costs of diabetes in the U.S. in 2012. *Diabetes Care*. 2013;36(4):1033-1046. doi:10.2337/dc12-2625.
9. Zhuo X, Zhang P, Barker L, Albright A, Thompson TJ, Gregg E. The lifetime cost of diabetes and its implications for diabetes prevention. *Diabetes Care*. 2014;37(9):2557-2564. doi:10.2337/dc13-2484.
10. WHO, International Diabetes Foundation. *Definition and Diagnosis of Diabetes Mellitus and Intermediate Hyperglycaemia: Report of a WHO/IDF Consultation*. Geneva: Geneva: World Health Organization; 2006.
11. The International Expert Committee. International Expert Committee Report on the Role of the A1C Assay in the Diagnosis of Diabetes. *Diabetes Care*. 2009;32(7):1327-1334. doi:10.2337/dc09-9033.
12. American Diabetes Association. Standards of Medical Care in Diabetes - 2017. *Diabetes Care*. 2017;40(Supplement 1):S33-S43. doi:10.2337/dc16-S003.
13. Gerstein HC, Santaguida P, Raina P, et al. Annual incidence and relative risk of diabetes in people with various categories of dysglycemia: A systematic overview and meta-analysis of prospective studies. *Diabetes Res Clin Pract*. 2007;78(3):305-312. doi:10.1016/j.diabres.2007.05.004.
14. Huang Y, Cai X, Mai W, Li M, Hu Y. Association between prediabetes and risk of cardiovascular disease and all cause mortality: systematic review and meta-analysis. *BMJ*. 2016;355:i5953. doi:10.1136/bmj.i5953.
15. Tabák AG, Herder C, Rathmann W, Brunner EJ, Kivimäki M. Prediabetes: A high-risk state for diabetes development. *Lancet*. 2012;379(9833):2279-2290. doi:10.1016/S0140-

6736(12)60283-9.

16. Diabetes Prevention Program Research Group. 10-year follow-up of diabetes incidence and weight loss in the Diabetes Prevention Program Outcomes Study. *Lancet*. 2009;374(9702):1677-1686. doi:10.1016/S0140-6736(09)61457-4.
17. The DREAM (Diabetes REduction Assessment with ramipril and rosiglitazone Medication) Trial Investigators. Effect of rosiglitazone on the frequency of diabetes in patients with impaired glucose tolerance or impaired fasting glucose: a randomised controlled trial. *Lancet*. 2006;368(9541):1096-1105. doi:10.1016/S0140-6736(06)69420-8.
18. Perreault L, Pan Q, Mather KJ, Watson KE, Hamman RF, Kahn SE. Effect of regression from prediabetes to normal glucose regulation on long-term reduction in diabetes risk: Results from the Diabetes Prevention Program Outcomes Study. *Lancet*. 2012;379(9833):2243-2251. doi:10.1016/S0140-6736(12)60525-X.
19. Perreault L, Temprosa M, Mather KJ, et al. Regression from prediabetes to normal glucose regulation is associated with reduction in cardiovascular risk: Results from the diabetes prevention program outcomes study. *Diabetes Care*. 2014;37(9):2622-2631. doi:10.2337/dc14-0656.
20. Popkin BM. Nutrition Transition and the Global Diabetes Epidemic. *Curr Diab Rep*. 2015;15(9). doi:10.1007/s11892-015-0631-4.
21. Vazquez G, Duval S, Jacobs DRJ, Silventoinen K. Comparison of body mass index, waist circumference, and waist/hip ratio in predicting incident diabetes: a meta-analysis. *Epidemiol Rev*. 2007;29:115-128. doi:10.1093/epirev/mxm008.
22. Knowler WC, Barrett-Connor E, Fowler SE, et al. Reduction in the incidence of type 2 diabetes with lifestyle intervention or metformin. *N Engl J Med*. 2002;346(6):393-403. doi:10.1056/NEJMoa012512.
23. Pan XR, Li GW, Hu YH, et al. Effects of diet and exercise in preventing NIDDM in people with impaired glucose tolerance: The Da Qing IGT and diabetes study. *Diabetes Care*. 1997;20(4):537-544. doi:10.2337/diacare.20.4.537.
24. Tuomilehto J, Indstrom J, Eriksson J, Valle T, Hamalainine E, Uusitupa M. Prevention of Type 2 Diabetes Mellitus By Changes in Lifestyle Among Subjects With Impaired Glucose Tolerance. *N Engl J Med*. 2001;344(18):1343-1350. doi:10.1056/NEJM200105033441801.
25. Ramachandran A, Snehalatha C, Mary S, Mukesh B, Bhaskar AD, Vijay V. The Indian Diabetes Prevention Programme shows that lifestyle modification and metformin prevent type 2 diabetes in Asian Indian subjects with impaired glucose tolerance (IDPP-1). *Diabetologia*. 2006;49(2):289-297. doi:10.1007/s00125-005-0097-z.
26. The Diabetes Preventio Program Research Group. The Diabetes Prevention Program. Design and methods for a clinical trial in the prevention of type 2 diabetes. *Diabetes Care*. 1999;22(4):623-634. doi:10.2337/diacare.22.4.623 10.2337/diabetes.48.4.699.
27. Evert AB, Boucher JL, Cypress M, et al. Nutrition therapy recommendations for the management of adults with diabetes. *Diabetes Care*. 2013;36(11):3821-3842. doi:10.2337/dc13-2042.
28. Hu T, Mills KT, Yao L, et al. Effects of low-carbohydrate diets versus low-fat diets on metabolic risk factors: A meta-analysis of randomized controlled clinical trials. *Am J Epidemiol*. 2012;176(SUPPL. 7). doi:10.1093/aje/kws264.
29. Sackner-Bernstein J, Kanter D, Kaul S. Dietary Intervention for Overweight and Obese Adults: Comparison of Low-Carbohydrate and Low-Fat Diets. A Meta-Analysis. *PLoS*

One. 2015;10(10):e0139817. doi:10.1371/journal.pone.0139817.
30. Bazzano LA, Hu T, Reynolds K, et al. Effects of low-carbohydrate and low-fat diets: A randomized trial. *Ann Intern Med*. 2014;161(5):309-318. doi:10.7326/M14-0180.
31. Sawyer L, Gale EAM. Diet, delusion and diabetes. *Diabetologia*. 2009;52(1):1-7. doi:10.1007/s00125-008-1203-9.
32. Snorgaard O, Poulsen GM, Andersen HK, Astrup A. Systematic review and meta-analysis of dietary carbohydrate restriction in patients with type 2 diabetes. *BMJ Open Diabetes Res Care*. 2017;5(1):e000354. doi:10.1136/bmjdrc-2016-000354.
33. Larsen RN, Mann NJ, Maclean E, Shaw JE. The effect of high-protein, low-carbohydrate diets in the treatment of type 2 diabetes: A 12 month randomised controlled trial. *Diabetologia*. 2011;54(4):731-740. doi:10.1007/s00125-010-2027-y.
34. Saslow LR, Kim S, Daubenmier JJ, et al. A randomized pilot trial of a moderate carbohydrate diet compared to a very low carbohydrate diet in overweight or obese individuals with type 2 diabetes mellitus or prediabetes. *PLoS One*. 2014;9(4). doi:10.1371/journal.pone.0091027.
35. Tay J, Luscombe-Marsh ND, Thompson CH, et al. A very low-carbohydrate, low-saturated fat diet for type 2 diabetes management: A randomized trial. *Diabetes Care*. 2014;37(11):2909-2918. doi:10.2337/dc14-0845.
36. Davis NJ, Tomuta N, Schechter C, et al. Comparative Study of the Effects of a 1-Year Dietary Intervention of a Low- Carbohydrate Diet Versus a Low-Fat Diet on Weight and Glycemic Control in Type 2. *Diabetes Care*. 2009;32(7):1147-1152. doi:10.2337/dc08-2108.Clinical.
37. Saslow LR, Daubenmier JJ, Moskowitz JT, et al. Twelve-month outcomes of a randomized trial of a moderate-carbohydrate versus very low-carbohydrate diet in overweight adults with type 2 diabetes mellitus or prediabetes. *Nutr Diabetes*. 2017;7(12):304. doi:10.1038/s41387-017-0006-9.
38. Tay J, Thompson CH, Luscombe-Marsh ND, et al. Effects of an energy-restricted low-carbohydrate, high unsaturated fat/low saturated fat diet versus a high carbohydrate, low fat diet in type 2 diabetes: a 2 year randomized clinical trial. *Diabetes, Obes Metab*. 2017:n/a-n/a. doi:10.1111/dom.13164.
39. Yamada Y, Uchida J, Izumi H, et al. A non-calorie-restricted low-carbohydrate diet is effective as an alternative therapy for patients with type 2 diabetes. *Intern Med*. 2014;53(1):13-19. doi:http://dx.doi.org/10.2169/internalmedicine.53.0861.
40. Mayer SB, Jeffreys AS, Olsen MK, Mcduffie JR, Feinglos MN, Yancy WS. Two diets with different haemoglobin A1c and antiglycaemic medication effects despite similar weight loss in type 2 diabetes. *Diabetes, Obes Metab*. 2014;16(1):90-93. doi:10.1111/dom.12191.
41. Maekawa S, Kawahara T, Nomura R, et al. Retrospective study on the efficacy of a low-carbohydrate diet for impaired glucose tolerance. *Diabetes Metab Syndr Obes*. 2014;7:195-201. doi:10.2147/DMSO.S62681.
42. Davis C, Bryan J, Hodgson J, Murphy K. Definition of the mediterranean diet: A literature review. *Nutrients*. 2015;7(11):9139-9153. doi:10.3390/nu7115459.
43. Sofi F, Macchi C, Abbate R, Gensini GF, Casini A. Mediterranean diet and health status: an updated meta-analysis and a proposal for a literature-based adherence score. *Public Health Nutr*. 2014;17(12):2769-2782. doi:10.1017/S1368980013003169.
44. Jannasch F, Kröger J, Schulze MB. Dietary Patterns and Type 2 Diabetes: A Systematic Literature Review and Meta-Analysis of Prospective Studies. *J Nutr*. 2017;147(6):1174-

1182. doi:10.3945/jn.116.242552.

45. Schwingshackl L, Missbach B, König J, Hoffmann G. Adherence to a Mediterranean diet and risk of diabetes: a systematic review and meta-analysis. *Public Health Nutr*. 2014;18(7):1292–1299. doi:10.1017/S1368980014001542.

46. de Lorgeril M, Salen P, Martin J-L, Monjaud I, Delaye J, Mamelle N. Mediterranean Diet, Traditional Risk Factors, and the Rate of Cardiovascular Complications After Myocardial Infarction : Final Report of the Lyon Diet Heart Study. *Circulation*. 1999;99(6):779-785. doi:10.1161/01.CIR.99.6.779.

47. Estruch R, Ros E, Salas-Salvadó J, et al. Primary Prevention of Cardiovascular Disease with a Mediterranean Diet. *N Engl J Med*. 2013;368(14):1279-1290. doi:10.1056/NEJMoa1200303.

48. Salas-Salvadó J, Bulló M, Estruch R, et al. Prevention of diabetes with mediterranean diets: A subgroup analysis of a randomized trial. *Ann Intern Med*. 2014;160(1):1-10. doi:10.7326/M13-1725.

49. Esposito K, Marfella R, Ciotola M, et al. Effect of a Mediterranean-Style Diet on Endothelial Dysfunction and Markers of Vascular Inflammation in the Metabolic Syndrome. *JAMA*. 2004;292(12):1440. doi:10.1001/jama.292.12.1440.

50. Babio N, Toledo E, Estruch R, et al. Mediterranean diets and metabolic syndrome status in the PREDIMED randomized trial. *CMAJ*. 2014;186(17):E649-E657. doi:10.1503/cmaj.140764.

51. Esposito K, Maiorino MI, Bellastella G, Chiodini P, Panagiotakos D, Giugliano D. A journey into a Mediterranean diet and type 2 diabetes: a systematic review with meta-analyses. *BMJ Open*. 2015;5(8):e008222. doi:10.1136/bmjopen-2015-008222.

52. Micha R, Michas G, Mozaffarian D. Unprocessed red and processed meats and risk of coronary artery disease and type 2 diabetes--an updated review of the evidence. *Curr Atheroscler Rep*. 2012;14(6):515-524. doi:10.1007/s11883-012-0282-8.

53. Esposito K, Maiorino MI, Ciotola M, et al. Effects of a Mediterranean-style diet on the need for antihyperglycemic drug therapy in patients with newly diagnosed type 2 diabetes: A randomized trial. *Ann Intern Med*. 2009;151(5):306-314. doi:10.1097/OGX.0b013e3181e5a159.

54. Esposito K, Maiorino MI, Petrizzo M, Bellastella G, Giugliano D. The effects of a Mediterranean diet on the need for diabetes drugs and remission of newly diagnosed type 2 diabetes: Follow-up of a randomized trial. *Diabetes Care*. 2014;37(7):1824-1830. doi:10.2337/dc13-2899.

55. Elhayany A, Lustman A, Abel R, Attal-Singer J, Vinker S. A low carbohydrate Mediterranean diet improves cardiovascular risk factors and diabetes control among overweight patients with type 2 diabetes mellitus: A 1-year prospective randomized intervention study. *Diabetes, Obes Metab*. 2010;12(3):204-209. doi:10.1111/j.1463-1326.2009.01151.x.

56. Shai I, Schwarzfuchs D, Henkin Y, et al. Weight Loss with a Low-Carbohydrate, Mediterranean, or Low-Fat Diet. *N Engl J Med*. 2008;359(3):229-241. doi:10.1056/NEJMoa0708681.

57. Schwarzfuchs D, Golan R, Shai I. Four-Year Follow-up after Two-Year Dietary Interventions. *N Engl J Med*. 2012;367(14):1373-1374. doi:10.1056/NEJMc1204792.

58. Pan A, Sun Q, Bernstein AM, et al. Red meat consumption and risk of type 2 diabetes: 3 Cohorts of US adults and an updated meta-analysis. *Am J Clin Nutr*. 2011;94(4):1088-1096. doi:10.3945/ajcn.111.018978.

59. Hu EA, Pan A, Malik V, Sun Q. White rice consumption and risk of type 2 diabetes: meta-analysis and systematic review. *BMJ*. 2012;344:e1454. doi:10.1136/bmj.e1454.
60. Wang M, Yu M, Fang L, Hu R-Y. Association between sugar-sweetened beverages and type 2 diabetes: A meta-analysis. *J Diabetes Investig*. 2015;6(3):360-366. doi:10.1111/jdi.12309.
61. Imamura F, O’Connor L, Ye Z, et al. Consumption of sugar sweetened beverages, artificially sweetened beverages, and fruit juice and incidence of type 2 diabetes: systematic review, meta-analysis, and estimation of population attributable fraction. *BMJ*. 2015:h3576. doi:10.1136/bmj.h3576.
62. Carter P, Gray LJ, Troughton J, Khunti K, Davies MJ. Fruit and vegetable intake and incidence of type 2 diabetes mellitus: systematic review and meta-analysis. *BMJ*. 2010;341:c4229. doi:10.1136/bmj.c4229.
63. Cooper AJ, Forouhi NG, Ye Z, et al. Fruit and vegetable intake and type 2 diabetes: EPIC-InterAct prospective study and meta-analysis. *Eur J Clin Nutr*. 2012;66(10):1082-1092. doi:10.1038/ejcn.2012.85.
64. Chen M, Sun Q, Giovannucci E, et al. Dairy consumption and risk of type 2 diabetes: 3 cohorts of US adults and an updated meta-analysis. *BMC Med*. 2014;12(1):215. doi:10.1186/s12916-014-0215-1.
65. Aune D, Norat T, Romundstad P, Vatten LJ. Whole grain and refined grain consumption and the risk of type 2 diabetes: A systematic review and dose-response meta-analysis of cohort studies. *Eur J Epidemiol*. 2013;28(11):845-858. doi:10.1007/s10654-013-9852-5.
66. Ding M, Bhupathiraju SN, Chen M, van Dam RM, Hu FB. Caffeinated and decaffeinated coffee consumption and risk of type 2 diabetes: a systematic review and a dose-response meta-analysis. *Diabetes Care*. 2014;37(2):569-586. doi:10.2337/dc13-1203.
67. Li X-H, Yu F-F, Zhou Y-H, He J. Association between alcohol consumption and the risk of incident type 2 diabetes: a systematic review and dose-response meta-analysis. *Am J Clin Nutr*. 2016;103(3):818-829. doi:10.3945/ajcn.115.114389.
68. Muraki I, Imamura F, Manson JE, et al. Fruit consumption and risk of type 2 diabetes: results from three prospective longitudinal cohort studies. *BMJ*. 2013;347(aug28 1):f5001-f5001. doi:10.1136/bmj.f5001.
69. Upadhyaya S, Banerjee G. Type 2 diabetes and gut microbiome: At the intersection of known and unknown. *Gut Microbes*. 2015;6(2):85-92. doi:10.1080/19490976.2015.1024918.
70. David LA, Maurice CF, Carmody RN, et al. Diet rapidly and reproducibly alters the human gut microbiome. *Nature*. 2014;505(7484):559-563. doi:10.1038/nature12820.
71. Duncan SH, Belenguer A, Holtrop G, Johnstone AM, Flint HJ, Lobley GE. Reduced dietary intake of carbohydrates by obese subjects results in decreased concentrations of butyrate and butyrate-producing bacteria in feces. *Appl Environ Microbiol*. 2007;73(4):1073-1078. doi:10.1128/AEM.02340-06.
72. Brownlee M, Hirsch IB. Glycemic variability: a hemoglobin A1c-independent risk factor for diabetic complications. *JAMA*. 2006;295(14):1707-1708. doi:10.1001/jama.295.14.1707.
73. Smith-Palmer J, Brändle M, Trevisan R, Orsini Federici M, Liabat S, Valentine W. Assessment of the association between glycemic variability and diabetes-related complications in type 1 and type 2 diabetes. *Diabetes Res Clin Pract*. 2014;105(3):273-284. doi:10.1016/j.diabres.2014.06.007.
74. Nalysnyk L, Hernandez-Medina M, Krishnarajah G. Glycaemic variability and complications in patients with diabetes mellitus: Evidence from a systematic review of the

literature. *Diabetes, Obes Metab*. 2010;12(4):288-298. doi:10.1111/j.1463-1326.2009.01160.x.

75. J. T, N.D. L-M, C.H. T, et al. Comparison of low- and high-carbohydrate diets for type 2 diabetes management: A randomized trial. *Am J Clin Nutr*. 2015;102(4):780-790. doi:10.3945/ajcn.115.112581.1.
76. Dorans KS, Mostofsky E, Levitan EB, Håkansson N, Wolk A, Mittleman MA. Alcohol and Incident Heart Failure among Middle-Aged and Elderly Men: Cohort of Swedish Men. *Circ Hear Fail*. 2015;8(3):422-427. doi:10.1161/CIRCHEARTFAILURE.114.001787.
77. Steinhaus DA, Mostofsky E, Levitan EB, et al. Chocolate intake and incidence of heart failure: Findings from the Cohort of Swedish Men. *Am Heart J*. 2017;183:18-23. doi:10.1016/j.ahj.2016.10.002.
78. Dorans KS, Massa J, Chitnis T, Ascherio A, Munger KL. Physical activity and the incidence of multiple sclerosis. *Neurology*. 2016;87(17):1770-1776. doi:10.1212/WNL.0000000000003260.
79. Mills KT, Chen J, Yang W, et al. Sodium Excretion and the Risk of Cardiovascular Disease in Patients With Chronic Kidney Disease. *JAMA*. 2016;315(20):2200. doi:10.1001/jama.2016.4447.
80. Chen J, Gu D, Huang J, et al. Metabolic syndrome and salt sensitivity of blood pressure in non-diabetic people in China: a dietary intervention study. *Lancet*. 2009;373(9666):829-835. doi:10.1016/S0140-6736(09)60144-6.
81. Li C, He J, Chen J, et al. Genome-Wide Gene-Sodium Interaction Analyses on Blood Pressure: The Genetic Epidemiology Network of Salt-Sensitivity Study. *Hypertension*. 2016;68(2):348-355. doi:10.1161/HYPERTENSIONAHA.115.06765.
82. He J, Streiffer RH, Muntner P, Krousel-Wood MA, Whelton PK. Effect of dietary fiber intake on blood pressure: a randomized, double-blind, placebo-controlled trial. *J Hypertens*. 2004;22(1):73-80. doi:10.1097/01.hjh.0000098164.70956.54.
83. He J, Ogden LG, Vupputuri S, Bazzano LA, Loria C, Whelton PK. Dietary sodium intake and subsequent risk of cardiovascular disease in overweight adults. *JAMA*. 1999;282(21):2027-2034. doi:10.1001/jama.282.21.2027.
84. He J, Wofford MR, Reynolds K, et al. Effect of dietary protein supplementation on blood pressure a randomized, controlled trial. *Circulation*. 2011;124(5):589-595. doi:10.1161/CIRCULATIONAHA.110.009159.
85. Thompson AM, Zhang Y, Tong W, et al. Association of inflammation and endothelial dysfunction with metabolic syndrome, prediabetes and diabetes in adults from Inner Mongolia, China. *BMC Endocr Disord*. 2011;11(1):16. doi:10.1186/1472-6823-11-16.
86. Irazola V, Rubinstein A, Bazzano L, et al. Prevalence, awareness, treatment and control of diabetes and impaired fasting glucose in the Southern Cone of Latin America. *PLoS One*. 2017;12(9). doi:10.1371/journal.pone.0183953.
87. Bazzano LA, Li TY, Joshipura KJ, Hu FB. Intake of fruit, vegetables, and fruit juices and risk of diabetes in women. *Diabetes Care*. 2008;31(7):1311-1317. doi:10.2337/dc08-0080.
88. Bazzano LA, Serdula M, Liu S. Prevention of Type 2 Diabetes by Diet and Lifestyle Modification. *J Am Coll Nutr*. 2005;24(5):310-319. doi:10.1080/07315724.2005.10719479.
89. Pollock BD, Chen W, Harville EW, et al. Differential sex effects of systolic blood pressure and low-density lipoprotein cholesterol on type 2 diabetes: Life course data from the Bogalusa Heart Study. *Journal of Diabetes*. 2017.

90. Zhang T, Li Y, Zhang H, et al. Insulin-sensitive adiposity is associated with a relatively lower risk of diabetes than insulin-resistant adiposity: the Bogalusa Heart Study. *Endocrine*. 2016;54(1):93-100. doi:10.1007/s12020-016-0948-z.

91. Pollock BD, Hu T, Chen W, et al. Utility of existing diabetes risk prediction tools for young black and white adults: Evidence from the Bogalusa Heart Study. *J Diabetes Complications*. 2016;31(1):86-93. doi:10.1016/j.jdiacomp.2016.07.025.

92. Hu T, Yao L, Reynolds K, et al. The effects of a low-carbohydrate diet vs. a low-fat diet on novel cardiovascular risk factors: A randomized controlled trial. *Nutrients*. 2015;7(9):7978-7994. doi:10.3390/nu7095377.

93. Hu T, Yao L, Reynolds K, et al. The effects of a low-carbohydrate diet on appetite: A randomized controlled trial. *Nutr Metab Cardiovasc Dis*. 2016;26(6):476-488. doi:10.1016/j.numecd.2015.11.011.

94. Rebholz CM, Reynolds K, Wofford MR, et al. Effect of soybean protein on novel cardiovascular disease risk factors: A randomized controlled trial. *Eur J Clin Nutr*. 2013;67(1):58-63. doi:10.1038/ejcn.2012.186.

95. Wofford MR, Rebholz CM, Reynolds K, et al. Effect of soy and milk protein supplementation on serum lipid levels: A randomized controlled trial. *Eur J Clin Nutr*. 2012;66(4):419-425. doi:10.1038/ejcn.2011.168.

96. Chen J, He J, Wildman RP, Reynolds K, Streiffer RH, Whelton PK. A randomized controlled trial of dietary fiber intake on serum lipids. *Eur J Clin Nutr*. 2006;60(1):62-68. doi:10.1038/sj.ejcn.1602268.

97. Overweight and Obesity Expert Panel. *Managing Overweight and Obesity in Adults: Systematic Evidence Review From the Obesity Expert Panel, 2013*.; 2013.

98. Shrinath N, Mack V, Plyer A. *Who Lives in New Orleans and Metro Parishes Now ?*; 2013. https://www.datacenterresearch.org/data-resources/who-lives-in-new-orleans-now/.

99. Wetzels JFM, Kiemeney LALM, Swinkels DW, Willems HL, Heijer M Den. Age- and gender-specific reference values of estimated GFR in Caucasians: The Nijmegen Biomedical Study. *Kidney Int*. 2007;72(5):632-637. doi:10.1038/sj.ki.5002374.

100. Radin MS. Pitfalls in hemoglobin A1c measurement: When results may be misleading. *J Gen Intern Med*. 2014;29(2):388-394. doi:10.1007/s11606-013-2595-x.

101. Kim PS, Woods C, Georgoff P, et al. A1C underestimates glycemia in HIV infection. *Diabetes Care*. 2009;32(9):1591-1593. doi:10.2337/dc09-0177.

102. Qaseem A, Wilt TJ, Kansagara D, Horwitch C, Barry MJ, Forciea MA. Hemoglobin A1c Targets for Glycemic Control With Pharmacologic Therapy for Nonpregnant Adults With Type 2 Diabetes Mellitus: A Guidance Statement Update From the American College of Physicians. *Ann Intern Med*. 2018;168(8):569-576. doi:10.7326/M17-0939.

103. Sumithran P, Prendergast LA, Delbridge E, et al. Ketosis and appetite-mediating nutrients and hormones after weight loss. *Eur J Clin Nutr*. 2013;67(7):759-764. doi:10.1038/ejcn.2013.90.

104. Gardner CD, Trepanowski JF, Gobbo LCD, et al. Effect of low-fat VS low-carbohydrate diet on 12-month weight loss in overweight adults and the association with genotype pattern or insulin secretion the DIETFITS randomized clinical trial. *JAMA - J Am Med Assoc*. 2018;319(7):667-679. doi:10.1001/jama.2018.0245.

105. Brenner BM, Meyer TW, Hostetter TH. Dietary protein intake and the progressive nature of kidney disease: the role of hemodynamically mediated glomerular injury in the pathogenesis of progressive glomerular sclerosis in aging, renal ablation, and intrinsic renal disease. *N Engl J Med*. 1982;307(11):652-659.

doi:10.1056/NEJM198209093071104.

106. Sato J, Kanazawa A, Makita S, et al. A randomized controlled trial of 130 g/day low-carbohydrate diet in type 2 diabetes with poor glycemic control. *Clin Nutr*. 2017;36(4):992-1000. doi:10.1016/j.clnu.2016.07.003.

107. Yancy WS, Foy M, Chalecki AM, et al. A low-carbohydrate, ketogenic diet to treat type 2 diabetes. *Nutr Metab (Lond)*. 2005;2(1):34. doi:10.1186/1743-7075-2-34.

108. Boden G, Sargrad K, Homko C, Mozzoli M, Stein TP. Effect of a low-carbohydrate diet on appetite, blood glucose levels, and insulin resistance in obese patients with type 2 diabetes. *Ann Intern Med*. 2005;142(6). doi:10.1016/S0084-3741(08)70336-6.

109. Stern L, Iqbal N, Seshadri P. The Effects of Low-Carbohydrate versus Conventional Weight Loss Diets in Severely Obese Adults : One-Year Follow-up of a randomized control trial. *Ann Intern …*. 2004;140(May 2001):769-777. doi:10.1016/j.accreview.2004.07.103.

110. Westman EC, Yancy WS, Edman JS, Tomlin KF, Perkins CE. Effect of 6-month adherence to a very low carbohydrate diet program. *Am J Med*. 2002;113(1):30-36. doi:10.1016/S0002-9343(02)01129-4.

111. Moosheer SM, Waldschütz W, Itariu BK, Brath H, Stulnig TM. A protein-enriched low glycemic index diet with omega-3 polyunsaturated fatty acid supplementation exerts beneficial effects on metabolic control in type 2 diabetes. *Prim Care Diabetes*. 2014;8(4):308-314. doi:10.1016/j.pcd.2014.02.004.

112. Bailey T, Bode BW, Christiansen MP, Klaff LJ, Alva S. The Performance and Usability of a Factory-Calibrated Flash Glucose Monitoring System. *Diabetes Technol Ther*. 2015;17(11):787-794. doi:10.1089/dia.2014.0378.

113. Rohlfing C, Hanson S, Weykamp C, et al. Effects of hemoglobin C, D, E and S traits on measurements of hemoglobin A1c by twelve methods. *Clin Chim Acta*. 2016. doi:10.1016/j.cca.2016.01.031.

114. Aagaard K, Petrosino J, Keitel W, et al. The Human Microbiome Project strategy for comprehensive sampling of the human microbiome and why it matters. *FASEB J*. 2013;27(3):1012-1022. doi:10.1096/fj.12-220806.

115. McInnes P, Cutting M. Manual of Procedures for Human Microbiome Project. http://www.hmpdacc.org/doc/HMP_MOP_Version12_0_072910.pdf.

116. Maruthur NM, Ma Y, Delahanty LM, et al. Early response to preventive strategies in the diabetes prevention program. *J Gen Intern Med*. 2013;28(12):1629-1636. doi:10.1007/s11606-013-2548-4.

117. Leong A, Daya N, Porneala B, et al. Prediction of Type 2 Diabetes by Hemoglobin A1c in Two Community-Based Cohorts. *Diabetes Care*. 2017;41(1):dc170607. doi:10.2337/dc17-0607.

118. Khaw K-T, Wareham N, Bingham S, Luben R, Welch A, Day N. Association of hemoglobin A1c with cardiovascular disease and mortality in adults: the European prospective investigation into cancer in Norfolk. *Ann Intern Med*. 2004;141(6):413-420. doi:10.7326/0003-4819-141-6-200409210-00006.

119. Cole RE, Boyer KM, Spanbauer SM, Sprague D, Bingham M. Effectiveness of Prediabetes Nutrition Shared Medical Appointments. *Diabetes Educ*. 2013;39(3):344-353. doi:10.1177/0145721713484812.
