# Supplementary material for: Effects of a Low-Carbohydrate Dietary Intervention on Hemoglobin A1c: A Randomized Clinical Trial
Source: JAMA Netw Open. 2022 Oct 26;5(10):e2238645. doi: 10.1001/jamanetworkopen.2022.38645 (PMC9606840; doi:10.1001/jamanetworkopen.2022.38645)
Supplement: Supplement 2. — eAppendix. Supplemental Methods eReferences eTable 1. Self-Reported Median Physical Activity Levels Over Time by Assigned Arm eTable 2. Mean Differences in Continuous Glucose Monitor Outcomes at 6 Months, by Assigned Arm, Among Those With at Least 10 Days of Full 24-h Data eTable 3. Change in Metabolic Risk Factors From Baseline, Within and Between Arms, After Adjusting for Baseline Level of Outcome, Race and Baseline Weight, Waist Circumference, Fasting Glucose, LDL Cholesterol, Total Cholesterol, and Physical Activity eTable 4. Change in Metabolic Risk Factors From Baseline, Within and Between Arms, by Assigned Arm After Multiple Imputation eTable 5. Subgroup Analyses of Change in Metabolic Risk Factors From Baseline, Within and Between Arms eTable 6. Total Number of Times Any Adverse Event Was Reported eTable 7. Symptoms Reported by Participants: Percent of Participants in Each Arm (95% CI) Reporting Symptoms at 3 Months and 6 Months [file jamanetwopen-e2238645-s002.pdf]

Supplemental Online Content

Dorans KS, Bazzano LA, Qi L, et al. Effects of a low-carbohydrate dietary intervention on hemoglobin A_{1c}: a randomized clinical trial. *JAMA Netw Open*. 2022;5(10):e2238645. doi:10.1001/jamanetworkopen.2022.38645

eAppendix. Supplemental Methods
eReferences
eTable 1. Self-Reported Median Physical Activity Levels Over Time by Assigned Arm
eTable 2. Mean Differences in Continuous Glucose Monitor Outcomes at 6 Months, by Assigned Arm, Among Those With at Least 10 Days of Full 24-h Data
eTable 3. Change in Metabolic Risk Factors From Baseline, Within and Between Arms, After Adjusting for Baseline Level of Outcome, Race and Baseline Weight, Waist Circumference, Fasting Glucose, LDL Cholesterol, Total Cholesterol, and Physical Activity
eTable 4. Change in Metabolic Risk Factors From Baseline, Within and Between Arms, by Assigned Arm After Multiple Imputation
eTable 5. Subgroup Analyses of Change in Metabolic Risk Factors From Baseline, Within and Between Arms
eTable 6. Total Number of Times Any Adverse Event Was Reported
eTable 7. Symptoms Reported by Participants: Percent of Participants in Each Arm (95% CI) Reporting Symptoms at 3 Months and 6 Months

This supplemental material has been provided by the authors to give readers additional information about their work.

eAppendix. Supplemental Methods

Study Design and Intervention

Low-carbohydrate dietary intervention arm additional details. Interventionists were registered dietitians or had undergraduate-level education in human performance/health promotion. The intervention was focused on helping participants develop a realistic plan of small yet effective changes. Key recommended foods included non-starchy vegetables, fish, poultry, meat trimmed of fat, eggs, olive oil and other plant-based unsaturated oils, unsweetened/unsalted nuts and seeds, nut butters, avocados, and moderate consumption of cheese, unsweetened Greek yogurt and low-carbohydrate milk. We recommended limiting or avoiding other dairy, fruits, legumes, beans, and grains.

To facilitate dietary modifications, participants received supplemental food at baseline (one 1-L container of olive oil, 3 cans of green beans, 3 cans of tomatoes, and samples of non-sugar sweetener) and throughout the study (4 ounces of walnuts, 1 ounce of almonds, 2 low-carbohydrate bars, 2 low-carbohydrate shakes, and 1 can of tuna per week and samples of non-sugar sweetener).

Statistical Analysis

Sensitivity analyses. We adjusted for baseline levels of the outcome of interest, race, and baseline covariates: weight, waist circumference, fasting glucose, LDL cholesterol, total cholesterol, and physical activity. We assessed whether results were similar when multiple imputation (Markov-chain Monte Carlo technique) was used to impute missing values.[1] Given the small amount of missingness, we followed our protocol to use multiple imputation as a sensitivity analyses, rather than in the main analysis.[2]

Subgroup analyses. We conducted post hoc subgroup analyses examining heterogeneity of the effect on hemoglobin A1c change by race (Black and White participants) and sex, using statistical tests of interaction between treatment and subgroup within mixed effects models. We conducted subgroup analyses by period (enrollment from October 2018 to August 2019 vs. from July to December 2020), representing participants who completed the study before lockdowns related to COVID-19 and those enrolled after start of the pandemic.

Continuous glucose monitor analyses. Continuous glucose monitor analyses were restricted to participants with ≥10 days of full 24-h data, per guidelines.[3] Per manufacturer, we excluded data from the first 24 hours. We calculated mean 24-h, day-time (6am-10pm), and night-time (10pm-6am) glucose. In addition to mean glucose measurements, we chose several other core continuous glucose monitor metrics based on the Advanced Technologies & Treatments for Diabetes (ATTD) Congress consensus recommendations.[3,4] To assess glycemic variability, we estimated the coefficient of variation and standard deviation. The coefficient of variation is calculated as the ratio of the standard deviation of glucose to mean glucose. For investigating glucose target control within specific ranges, we chose three glucose ranges: Time in Range (TIR) 70-120, 70-140, and 70-180 mg/dL. The TIR of 70-180 mg/dL is recommended by ATTD for nonpregnant adults with type 2 diabetes, given its relationship with diabetes complications and hemoglobin A1c.[4] As there are not guidelines for TIR reporting for people without diabetes, we chose additional upper bound cut-points. We chose the range of 70 to 120 mg/dL, as people without diabetes or prediabetes would be expected to have blood sugar levels between 70 to 120 mg/dL most of the time. We chose the upper bound of 140 mg/dL, as post-meal hyperglycemia is considered to be glucose >140 mg/dL 1-2 hours after eating food and 140 mg/dL is the cut-point for impaired glucose tolerance on a 75-g 2-hour oral glucose tolerance test.[5,6] We calculated these measures using the R package cgmanalysis.[7]

© 2022 Dorans KS et al. *JAMA Network Open.*

eReferences

1. Sterne JAC, White IR, Carlin JB, et al. Multiple imputation for missing data in epidemiological and clinical research: Potential and pitfalls. *BMJ*. 2009;338:b2393. doi:10.1136/bmj.b2393
2. Dorans KS, Bazzano LA, Qi L, et al. Low-carbohydrate dietary pattern on glycemic outcomes trial (ADEPT) among individuals with elevated hemoglobin A1c: study protocol for a randomized controlled trial. *Trials*. 2021;22(1):108. doi:10.1186/s13063-020-05001-x
3. Danne T, Nimri R, Battelino T, et al. International consensus on use of continuous glucose monitoring. *Diabetes Care*. 2017;40(12):1631-1640. doi:10.2337/dc17-1600
4. Battelino T, Danne T, Bergenstal RM, et al. Clinical targets for continuous glucose monitoring data interpretation: Recommendations from the international consensus on time in range. *Diabetes Care*. Published online 2019. doi:10.2337/dci19-0028
5. International Diabetes Federation Guideline Development Group. Guideline for management of postmeal glucose in diabetes. *Diabetes Res Clin Pract*. 2014;103(2):256-268. doi:10.1016/j.diabres.2012.08.002
6. American Diabetes Association. Standards of Medical Care in Diabetes-2021. *Diabetes Care*. 2021;44(Suppl 1):S1-S232.
7. Vigers T, Chan CL, Snell-Bergeon J, et al. Cgmanalysis: An R package for descriptive analysis of continuous glucose monitor data. *PLoS One*. 2019;14(10):e0216851. doi:10.1371/journal.pone.0216851

eTable 1. Self-Reported Median Physical Activity Levels Over Time by Assigned Arm

	Median physical activity level (IQR), MET-h/wk				
	**Low-Carbohydrate (n=75)**		**Usual Diet (n=74)**		**P value[a]**
Baseline	19.6	(8.3 to 39.0)	14.9	(8.3 to 37.2)	0.4
3 mo	19.3	(8.6 to 37.7)	23.4	(7.7 to 39.1)	0.8
6 mo	27.0	(9.8 to 71.9)	15.7	(7.7 to 32.3)	0.05

[a]Mann-Whitney U test
Physical activity data missing from one participant at baseline; missing from 10 participants at 3 mo and 9 participants at 6 mo.

eTable 2. Mean Differences in Continuous Glucose Monitor Outcomes at 6 Months, by Assigned Arm, Among Those With at Least 10 Days of Full 24-h Data

	**Low-Carbohydrate Diet (N=35)**	**Usual Diet (N=24)**	**Difference**	**P value**
Mean 24-hour glucose, mg/dL	96.7 (93.0 to 100.3)	103.7 (97.0 to 110.3)	-7.0 (-13.8 to -0.1)	0.05
Mean day-time glucose, mg/dL[a]	98.3 (94.6 to 101.9)	104.1 (97.7 to 110.5)	-5.9 (-12.5 to 0.8)	0.08
Mean night-time glucose, mg/dL[a]	92.8 (88.6 to 97.0)	102.5 (94.7 to 110.3)	-9.7 (-17.7 to -1.7)	0.02
CV	0.18 (0.17 to 0.20)	0.19 (0.17 to 0.20)	-0.01 (-0.03 to 0.02)	0.54
SD, mg/dL	17.7 (16.0 to 19.4)	19.8 (17.7 to 21.9)	-2.1 (-4.8 to 0.6)	0.13
TIR 70–120 mg/dL, %	83.2 (78.9 to 87.4)	73.4 (65.3 to 81.4)	9.8 (1.6 to 18.0)	0.02
TIR 70–140 mg/dL, %	90.5 (87.9 to 93.1)	86.0 (80.9 to 91.2)	4.4 (0.7 to 9.6)	0.09
TIR 70–180 mg/dL, %	95.1 (93.1 to 97.0)	94.8 (91.6 to 98.0)	0.2 (-3.2 to 3.7)	0.89

CV = coefficient of variation; SD = standard deviation; TIR = percent of specified time-in-range.
[a]Day-time was considered to be 6am to 10pm and night-time from 10pm to 6am.
SI conversion factors: To convert glucose to mmol/L, multiply by 0.0555.

© 2022 Dorans KS et al. *JAMA Network Open.*

eTable 3. Change in Metabolic Risk Factors From Baseline, Within and Between Arms, After Adjusting for Baseline Level of Outcome, Race and Baseline Weight, Waist Circumference, Fasting Glucose, LDL Cholesterol, Total Cholesterol, and Physical Activity

	**Change from Baseline (95% CI)**		**Difference in Change from Baseline (95% CI)**	
**Variable**	**Low-Carbohydrate Diet (N=73)**	**Usual Diet (N=69)**	**Difference in Change**	**P value**
Hemoglobin A1c, %				
3 mo	-0.21 (-0.28 to -0.15)	-0.11 (-0.16 to -0.05)	-0.11 (-0.19 to -0.02)	0.01
6 mo	-0.25 (-0.30 to -0.19)	-0.08 (-0.14 to -0.01)	-0.17 (-0.25 to -0.09)	<0.001
Fasting plasma glucose, mg/dL				
3 mo	0 (-2.9 to 3.0)	3.3 (-0.7 to 7.4)	-3.3 (-8.4 to 1.8)	0.20
6 mo	-5.9 (-8.6 to -3.2)	-0.5 (-4.0 to 3.0)	-5.4 (-9.9 to -0.9)	0.02
Systolic blood pressure, mmHg				
3 mo	-4.2 (-6.5 to -2.0)	-0.1 (-2.4 to 2.2)	-4.1 (-7.4 to -0.8)	0.01
6 mo	-4.9 (-7.6 to -2.2)	-1.7 (-4.4 to 1.1)	-3.2 (-7.1 to 0.7)	0.10
Total-to-HDL cholesterol, mg/dL				
3 mo	-0.46 (-0.55 to -0.37)	-0.43 (-0.53 to -0.33)	-0.03 (-0.17 to 0.11)	0.68
6 mo	-0.54 (-0.63 to -0.44)	-0.42 (-0.51 to -0.33)	-0.12 (-0.25 to 0.02)	0.09
Body weight, kg				
3 mo	-4.3 (-5.1 to -3.5)	-0.7 (-1.4 to -0.1)	-3.5 (-4.6 to -2.5)	<0.001
6 mo	-6.2 (-7.3 to -5.0)	-0.8 (-1.7 to 0)	-5.3 (-6.8 to -3.9)	<0.001
Exploratory				
Fasting insulin, µIU/L				
3 mo	-3.7 (-6.3 to -1.1)	1.1 (-1.5 to 3.7)	-4.7 (-8.7 to -0.8)	0.02
6 mo	-3.6 (-6.6 to -0.6)	2 (-1.1 to 5.1)	-5.6 (-10.1 to -1.1)	0.02
HOMA-IR				
3 mo	-0.9 (-1.7 to 0)	0.6 (-0.4 to 1.5)	-1.4 (-2.8 to -0.1)	0.04
6 mo	-1.3 (-2.2 to -0.5)	0.5 (-0.5 to 1.5)	-1.8 (-3.2 to -0.4)	0.01
Diastolic blood pressure, mmHg				
3 mo	-3.4 (-4.8 to -2.0)	0.4 (-1.1 to 2.0)	-3.8 (-5.9 to -1.7)	<0.001
6 mo	-3.3 (-5.1 to -1.5)	0 (-1.7 to 1.6)	-3.3 (-5.8 to -0.8)	0.009
Waist circumference, cm				
3 mo	-2.9 (-4.0 to -1.8)	-0.5 (-1.3 to 0.4)	-2.4 (-3.8 to -1.1)	<0.001
6 mo	-5.2 (-6.7 to -3.7)	-0.5 (-1.6 to 0.6)	-4.7 (-6.5 to -2.8)	<0.001
10-year ASCVD score, %				
3 mo	-1.2 (-1.9 to -0.4)	-0.6 (-1.2 to 0)	-0.6 (-1.6 to 0.5)	0.29
6 mo	-1.6 (-2.3 to -1.0)	-1.1 (-1.8 to -0.4)	-0.5 (-1.5 to 0.5)	0.29
HDL, mg/dL				
3 mo	3.1 (1.5 to 4.8)	2.8 (1.3 to 4.3)	0.3 (-2.0 to 2.6)	0.79
6 mo	5.3 (3.6 to 7.0)	3.3 (1.6 to 5.0)	2.0 (-0.5 to 4.4)	0.12
LDL, mg/dL				
3 mo	5.3 (1.5 to 9.0)	3.2 (-1.4 to 7.8)	2 (-3.9 to 8.0)	0.50
6 mo	2.6 (-1.2 to 6.5)	4.6 (-0.1 to 9.4)	-2 (-8.2 to 4.1)	0.52

© 2022 Dorans KS et al. *JAMA Network Open.*

HDL = high-density lipoprotein; HOMA-IR = homeostasis model assessment of insulin resistance; ASCVD= atherosclerotic cardiovascular disease; LDL = low-density lipoprotein
SI conversion factors: To convert glucose to mmol/L, multiply by 0.0555; insulin to pmol/L, multiply by 6.945; total cholesterol to mmol/L, multiply by 0.0259; HDL cholesterol to mmol/L, multiply by 0.0259; LDL cholesterol to mmol/L, multiply by 0.0259; triglycerides to mmol/L, multiply by 0.0113.
Adjusted for race (black vs other), baseline level of outcome, baseline physical activity (met-h/week, continuous), baseline weight (continuous), baseline waist circumference (continuous), baseline fasting glucose (continuous), baseline LDL (continuous), baseline total cholesterol (continuous).

© 2022 Dorans KS et al. *JAMA Network Open.*

eTable 4. Change in Metabolic Risk Factors From Baseline, Within and Between Arms, by Assigned Arm After Multiple Imputation

	Change from Baseline (95% CI)		Difference in Change from Baseline (95% CI)	
**Variable**	**Low-Carbohydrate Diet (N=75)**	**Usual Diet (N=75)**	**Difference in Change**	**P value**
Hemoglobin A1c, %				
3 mo	-0.22 (-0.30 to -0.14)	-0.07 (-0.12 to -0.03)	-0.15 (-0.24 to -0.06)	0.001
6 mo	-0.26 (-0.33 to -0.19)	-0.03 (-0.09 to 0.02)	-0.22 (-0.32 to -0.13)	<0.001
Fasting plasma glucose, mg/dL				
3 mo	-2.4 (-6.3 to 1.5)	5.5 (1.2 to 9.7)	-7.8 (-13.5 to -2.1)	0.007
6 mo	-8.7 (-12.6 to -4.7)	1.8 (-1.9 to 5.5)	-10.5 (-15.9 to -5.1)	<0.001
Systolic blood pressure, mmHg				
3 mo	-4.3 (-6.8 to -1.8)	-0.7 (-3.2 to 1.9)	-3.6 (-7.2 to 0)	0.05
6 mo	-4.8 (-7.9 to -1.8)	-2.0 (-5.0 to 0.9)	-2.8 (-7.0 to 1.4)	0.20
Total-to-HDL cholesterol, mg/dL				
3 mo	-0.45 (-0.58 to -0.32)	-0.42 (-0.53 to -0.30)	-0.03 (-0.20 to 0.14)	0.71
6 mo	-0.53 (-0.66 to -0.40)	-0.42 (-0.55 to -0.30)	-0.11 (-0.29 to 0.08)	0.26
Body weight, kg				
3 mo	-4.5 (-5.5 to -3.6)	-0.6 (-1.2 to 0)	-3.9 (-5.0 to -2.8)	<0.001
6 mo	-6.4 (-7.7 to -5.0)	-0.9 (-1.8 to 0)	-5.5 (-7.1 to -3.9)	<0.001
Exploratory				
Fasting insulin, µIU/L				
3 mo	-3.9 (-6.7 to -1.1)	1.5 (-1.0 to 4.0)	-5.4 (-9.2 to -1.6)	0.005
6 mo	-4.1 (7.3 to -0.8)	2.3 (-0.6 to 5.1)	-6.3 (-10.6 to -2.0)	0.004
HOMA-IR				
3 mo	-1.1 (-2.0 to -0.2)	0.9 (0 to 1.8)	-2.0 (-3.3 to -0.7)	0.002
6 mo	-1.7 (-2.6 to -0.7)	0.8 (-0.1 to 1.8)	-2.5 (-3.8 to -1.1)	<0.001
Diastolic blood pressure, mmHg				
3 mo	-3.2 (-4.8 to -1.7)	-0.3 (-2.2 to 1.6)	-3.0 (-5.4 to -0.5)	0.02
6 mo	-3.2 (-5.1 to -1.2)	-0.7 (-2.5 to 1.1)	-2.4 (-5.1 to 0.3)	0.08
Waist circumference, cm				
3 mo	-2.9 (-4.2 to -1.6)	-0.5 (-1.3 to 0.4)	-2.4 (-4.0 to -0.9)	0.002
6 mo	-5.0 (-6.8 to -3.3)	-0.7 (-1.9 to 0.4)	-4.3 (-6.4 to -2.3)	<0.001
10-year ASCVD score, %				
3 mo	-1.2 (-2.1 to -0.4)	-0.5 (-1.1 to 0.1)	-0.7 (-1.8 to 0.3)	0.15
6 mo	-1.6 (-2.4 to -0.9)	-1.0 (-1.6 to -0.3)	-0.7 (-1.7 to 0.4)	0.20
HDL, mg/dL				
3 mo	3.0 (1.2 to 4.9)	2.9 (1.5 to 4.3)	0.1 (-2.3 to 2.5)	0.93
6 mo	5.2 (3.5 to 6.9)	3.4 (1.7 to 5.1)	1.9 (-0.5 to 4.3)	0.13
LDL, mg/dL				
3 mo	5.8 (2.3 to 9.2)	3.3 (-1.3 to 7.9)	2.4 (-3.3 to 8.2)	0.41
6 mo	2.9 (-1.2 to 7.0)	3.5 (-1.3 to 8.3)	-0.6 (-6.9 to 5.7)	0.85

© 2022 Dorans KS et al. *JAMA Network Open.*

HDL = high-density lipoprotein; HOMA-IR = homeostasis model assessment of insulin resistance; ASCVD= atherosclerotic cardiovascular disease; LDL = low-density lipoprotein.
SI conversion factors: To convert glucose to mmol/L, multiply by 0.0555; insulin to pmol/L, multiply by 6.945; total cholesterol to mmol/L, multiply by 0.0259; HDL cholesterol to mmol/L, multiply by 0.0259; LDL cholesterol to mmol/L, multiply by 0.0259; triglycerides to mmol/L, multiply by 0.0113.

© 2022 Dorans KS et al. *JAMA Network Open.*

eTable 5. Subgroup Analyses of Change in Metabolic Risk Factors From Baseline, Within and Between Arms

	Change from Baseline (95% CI)		Difference in Change from Baseline (95% CI)	
	**Low-Carbohydrate Diet**	**Usual Diet**	**Intervention vs. Control Arm**	**P-value for interaction**
**Race**				
Black	-0.16 (-0.24 to -0.08)	-0.06 (-0.13 to 0.01)	-0.10 (-0.2 to 0.01)	0.003
White	-0.38 (-0.46 to -0.29)	-0.01 (-0.12 to 0.08)	-0.36 (-0.49 to -0.23)	
**Sex**				
Men	-0.42 (-0.53 to -0.31)	-0.01 (-0.13 to 0.10)	-0.41 (-0.57 to -0.25)	
Women	-0.2 (-0.27 to -0.13)	-0.06 (-0.13 to 0.01)	-0.14 (-0.24 to -0.04)	0.006
**Enrollment**				
Before September 2019	-0.23 (-0.31 to -0.14)	0.02 (-0.07 to 0.1)	-0.24 (-0.37 to -0.12)	0.24
July 2020 or later	-0.28 (-0.38 to -0.18)	-0.06 (-0.16 to 0.04)	-0.22 (-0.36 to -0.08)	

Adjusted for baseline level of hemoglobin A1c due to imbalance in baseline hemoglobin A1c in some subgroups.

© 2022 Dorans KS et al. *JAMA Network Open.*

eTable 6. Total Number of Times Any Adverse Event Was Reported

	Overall	Trial Assignment	
		Low-Carbohydrate	Usual Diet
**Adverse events**	11	6	5
**Serious adverse events**	7	4	3

No reported adverse events or serious adverse events were study related.

© 2022 Dorans KS et al. *JAMA Network Open.*

eTable 7. Symptoms Reported by Participants: Percent of Participants in Each Arm (95% CI) Reporting Symptoms at 3 Months and 6 Months

Symptom[a,*]	Trial assignment		*P* value[b]
	Low-Carbohydrate (N=73)	Usual Diet (N=69)	
Constipation			
3 mo	26 (8, to 37)	14 (8 to 25)	0.10
6 mo	22 (11 to 33)	19 (11 to 30)	0.64
Fatigue			
3 mo	39 (29 to 50)	40 (29 to 52)	0.86
6 mo	42 (26 to 54)	37 (26 to 49)	0.48
W eakness			
3 mo	14 (5 to 24)	10 (5, 20)	0.49
6 mo	14 (4 to 24)	9 (4 to 18)	0.34
Headache			
3 mo	36 (40 to 48)	51 (40 to 63)	0.06
6 mo	36 (31 to 47)	42 (31 to 54)	0.41
Excess thirst			
3 mo	21 (7 to 32)	13 (7 to 23)	0.21
6 mo	21 (13 to 31)	21 (13 to 32)	0.98
Polyuria, or producing excess urine			
3 mo	18 (7 to 29)	13 (7 to 23)	0.40
6 mo	15 (5 to 25)	10 (5 to 20)	0.38
Muscle cramps			
3 mo	35 (11 to 46)	19 (11 to 29)	0.03
6 mo	34 (11 to 46)	19 (11 to 30)	0.04
Loss of appetite			
3 mo	15 (2 to 25)	6 (2 to 14)	0.07
6 mo	11 (1 to 20)	4 (1 to 13)	0.15
Nausea or being sick to stomach			
3 mo	18 (5 to 29)	10 (5 to 20)	0.18
6 mo	15 (9 to 25)	16 (9 to 26)	0.90
Vomiting			
3 mo	3 (0 to 10)	1 (0 to 9)	0.59
6 mo	3 (1 to 10)	4 (1 to 13)	0.61
Diarrhea			
3 mo	12 (7 to 22)	13 (7 to 23)	0.94
6 mo	12 (7 to 22)	13 (7 to 23)	0.91
Heartburn			
3 mo	21 (16 to 32)	24 (16 to 36)	0.61
6 mo	16 (12 to 27)	20 (12 to 31)	0.57
Abdominal bloating or gas			
3 mo	31 (16 to 43)	24 (16 to 36)	0.37
6 mo	36 (22 to 47)	32 (22 to 43)	0.62
Bad breath			
3 mo	10 (7 to 19)	13 (7 to 23)	0.55
6 mo	7 (4 to 15)	9 (4 to 19)	0.59
Itching			
3 mo	16 (9 to 26)	16 (9 to 26)	1.0
6 mo	21 (11 to 31)	19 (11 to 30)	0.79
Hives or another type of rash			
3 mo	6 (6 to 14)	11 (6 to 21)	0.22
6 mo	4 (2 to 12)	6 (2 to 15)	0.59
Easy bruising or bleeding			
3 mo	8 (1 to 17)	3 (1 to 11)	0.18
6 mo	7 (1 to 15)	3 (1 to 11)	0.29

© 2022 Dorans KS et al. *JAMA Network Open.*

Symptom*	Low-Carbohydrate (N=73)	Usual Diet (N=69)	*P* value[b]
Numbness or tingling in hands and feet			
3 mo	22 (15 to 33)	23 (15 to 34)	0.91
6 mo	22 (18 to 33)	27 (18 to 39)	0.45
Feeling faint when standing up			
3 mo	4 (7 to 12)	13 (7 to 23)	0.08
6 mo	12 (5 to 22)	10 (5 to 20)	0.67
Generalized warmth			
3 mo	19 (9 to 30)	16 (9 to 26)	0.57
6 mo	15 (6 to 25)	12 (6 to 22)	0.58
Stuffy nose			
3 mo	33 (27 to 45)	37 (27 to 49)	0.62
6 mo	25 (27 to 36)	37 (27 to 49)	0.10
Dry mouth			
3 mo	23 (15 to 34)	23 (15 to 34)	0.94
6 mo	21 (12 to 31)	20 (12 to 31)	0.89
Wheezing			
3 mo	10 (3 to 19)	7 (3 to 16)	0.59
6 mo	5 (2 to 14)	6 (2 to 14)	0.95
Stomach pain			
3 mo	8 (8 to 17)	14 (8 to 25)	0.26
6 mo	8 (7 to 17)	13 (7 to 23)	0.36
Blurred vision			
3 mo	7 (3 to 16)	7 (3 to 16)	0.95
6 mo	7 (8 to 15)	14 (8 to 24)	0.17
Chest pain			
3 mo	1 (1 to 9)	3 (1 to 11)	0.55
6 mo	5 (2 to 14)	6 (2 to 14)	0.94
Palpitations			
3 mo	6 (2 to 14)	6 (2 to 14)	0.96
6 mo	8 (2 to 17)	6 (2 to 14)	0.57
Chest pressure			
3 mo	0	0	NA
6 mo	3 (1 to 10)	3 (1 to 11)	0.95

[a]Self-reported symptoms in the past month to close-ended questions
[b]For the between-arm difference at each time point.

© 2022 Dorans KS et al. *JAMA Network Open.*
