# Supplementary material for: Effects of a Low-Carbohydrate Dietary Intervention on Hemoglobin A1c: A Randomized Clinical Trial
Source: JAMA Netw Open. 2022 Oct 26;5(10):e2238645. doi: 10.1001/jamanetworkopen.2022.38645 (PMC9606840; doi:10.1001/jamanetworkopen.2022.38645)
Supplement: Supplement 3. — Data Sharing Statement [file jamanetwopen-e2238645-s003.pdf]

Data Sharing Statement

Dorans. Effects of a Low-Carbohydrate Dietary Intervention on Hemoglobin A_{1c}. *JAMA Netw Open*. Published October 26, 2022. doi:10.1001/jamanetworkopen.2022.38645

Data
Data available: Yes
Data types: Deidentified participant data
How to access data: kdorans@tulane.edu
When available: With publication

Supporting Documents
Document types: Statistical/analytic code
How to access documents: kdorans@tulane.edu
When available: With publication

Additional Information
Who can access the data: Researchers whose proposed use of the data has been approved
Types of analyses: Research purpose
Mechanisms of data availability: With approval of a proposal and investigator
